# Hepatoprotective Effect of *Citrus aurantium L.* Against APAP-induced Liver Injury by Regulating Liver Lipid Metabolism and Apoptosis

**DOI:** 10.7150/ijbs.40612

**Published:** 2020-01-14

**Authors:** Yisong Shu, Dan He, Wen Li, Menglei Wang, Siyu Zhao, Linlin Liu, Zhiwen Cao, Rui Liu, Yujuan Huang, Hui Li, Xueqing Yang, Cheng Lu, Yuanyan Liu

**Affiliations:** 1 Beijing University of Chinese Medicine, Beijing, 100029, China; 2 Patent Examination Cooperation (Tianjin) Center of the Patent Office, Tianjin, 300304, China; 3 Institute of Basic Research in Clinical Medicine, China Academy of Chinese Medical Sciences, Beijing, 100700, China.

**Keywords:** liver injury, apoptosis, hepatoprotective effect, liver metabolomics

## Abstract

Acetaminophen (APAP) refers to a medication used to manage pain and fever symptoms, but it always causes liver injury when overdosed. Zhishi, dried young fruit of *Citrus aurantium L.*, is a famous *Citrus* herbal medicine in Asian countries which is rich in dietary phenolic substances. In this study, the mechanism of Zhishi protected against APAP-induced liver injury was studied more deeply by metabolomic strategy and pharmacological study. The metabolomics results demonstrated that Zhishi can prevent the APAP-induced liver injury model by regulating liver metabolic disorders in glycerophospholipid metabolism, fatty acid biosynthesis and glycerolipid metabolism. Moreover, it is confirmed that Zhishi blocked apoptosis of APAP-induced BRL-3A cell by simultaneously regulating p53 up-regulated apoptosis regulator (PUMA), AMPK-SIRT1 and JNK1 signaling pathways. Our findings indicated that Zhishi exhibited a hepaprotective effect against APAP-induced liver necrosis by inhibiting the PUMA and reversing disorder of liver lipid metabolism which could assist in improving the clinical outcomes of chemical-induced liver injury.

## Introduction

In Asian, American and European countries, most of the causes of acute liver failure are acute liver damage caused by drugs. This failure is primarily attributable to acetaminophen (APAP) overdose 1. Excess APAP causes the accumulation of its toxic metabolite N-acetyl-p-benzoquinone (NAPQI), resulting in severe hepatotoxicity and hepatocellular necrosis 2. It is widely recognized that cytochrome P450 (especially CYP2E1, CYP3A4 and CYP1A2) activates APAP as a key initiation event for hepatotoxicity with NAPQI 3. Hepatocyte proteins and DNA are covalently attached after excess NAPQI has consumed intracellular glutathione (GSH). This will trigger mitochondrial oxidative stress damage and the body's lipid metabolism disorder, eventually leading to apoptosis and liver injury 4. Taken together, these findings indicate that inhibiting APAP bioactivation, regulating lipid metabolism and promoting liver regeneration are crucial steps in the prevention of APAP toxicity in liver. Thus, any compound that can affect these cascading linkages has the positive effects on hepatotoxicity induced by APAP.

Herbal medicines, which receive increasing attention in many medical areas today, have been widely used worldwide since ancient times due to their low toxicity and high efficiency 5. In recent years, herbs with homology of medicine and food have been widely studied all over the world. The fruit of *Citrus* plants is one of them, which contains a large amount of dietary phenolic substances that benefit to human health, and is highly praised around the world 6. In our previous study, the global constituents in Zhishi have been characterized (Fig. S2) using UPLC-Q-TOF-MS based metabolomics 5. Meanwhile, 32 phenolic compounds were quantified (Fig. S1) and validated using HPLC-DAD/UV and RRLC-QqQ-MS instruments 7-9. The established metabolomics-guided chemotaxonomic classification strategy was successfully applied for discrimination of four closely-related Citrus TCMs 5. Among them, Zhishi, the fruit of *Citrus aurantium L.*, a critical medicine in Asia since ancient times, is now included in the Pharmacopoeia of China with its actual clinical applications by regulating the balance and homeostasis of the body in a holistic fashion 9. In recent years, Zhishi exhibited many biological activities and pharmacological functions, such as free radical scavenging activity, anti-inflammatory activity, antioxidation activity and antiallergic activity 10. Our previous study has determined that Zhishi exhibited the strongest activity among the four closely-related *Citrus* TCMs 11, and can regulate liver metabolism via CYP, indicating the inhibition of Zhishi in APAP metabolic activation 9.

Therefore, for continuous and in-depth research, the purpose of current research was to use model animals and cell experiments to explore the effects of Zhishi in hepatotoxicity of APAP. We also investigated the hepatoprotective mechanism of lipid metabolism and hepatocyte necrosis involved in Zhishi-treated therapeutic activity against liver injury. Emerging hepatic metabolomics provides a new perspective for the study of lipid metabolism disorders during disease development and treatment. Metabolomics research is a comprehensive quantitative and qualitative analysis study that targets multiple metabolites and their interactions in vivo with relevant environmental variables, such as diet, disease, or drug intervention 12. As an aspect of hepatocyte apoptosis and necrosis, p53-upregulated modulator of apoptosis (PUMA) is of great important to a number of key activities associated with alleviating APAP-induced toxicity, and thus p53 is considered to be a protective target that could resist liver damage 13. P53 would be activated to inhibit cell proliferation and induce apoptosis when APAP overdosed, so low expression of p53 in liver injury promotes hepatocyte proliferation and liver repair 14-16. Resveratrol is a polygonal polyphenolic compound found in fruits and vegetables. It has recently attracted more and more attention because it has been proven to have a variety of medicinal activities, including anti-aging, anti-cancer, anti-inflammatory and prevent cardiovascular diseases, and to have a significant role in inflammation, oxidative stress, apoptosis, mitochondria Functional and angiogenic signaling pathways 17, particularly to protect APAP-induced acute liver injury in rats and to reduce apoptosis through the SIRT1 signaling pathway^18^-^20^. Therefore, RES is an ideal positive control drug for liver damage caused by oxidative stress. At present, liver metabolomics have been widely used in biomarker seeking for diagnosis and prognosis of diseases, drug toxicity and efficacy, genetic polymorphism and drug metabolism 13. Accordingly, liver metabolomics can be used to monitor the effect of Zhishi on liver lipid metabolism disorders caused by overdosed APAP at a systemic level and to identify potential intervention targets related to hepatic toxicity. Then, a p53 involved pathway was employed to explore the effect of Zhishi on hepatocyte apoptosis induced by APAP. Finally, in this study, the effect of Zhishi in APAP- related hepatotoxicity was observed both in vitro and in vivo.

## Materials and Methods

### Chemicals and materials

APAP and resveratrol (RES) were purchased from Shanghai Source Leaf Biological Technology Co., Ltd. HPLC-grade acetonitrile, methanol and formic acid were obtained from Dikma Technologies Inc. (Beijing, China). Deionized water (18.2 MΩ) was obtained from a Milli-Q water purification system (Millipore, Bedford, MA, USA). Assay design enzyme-linked immunosorbent assay kit (ELISA) was obtained from Nanjing Jiancheng Bioengineering Institute. All the reference standards were obtained from Chengdu Mansite Bio-technology Co., Ltd. The Zhishi sample was collected from Jiangxi province of China, and its species, local name, collection location, year of collection and growing environment are listed in the complementary materials. Its dried specimens were deposited at the Institute of Basic Theory, China Academy of Chinese Medical Sciences, Beijing, P. R. China.

### Preparation of Zhishi extract

In the previous study, the extraction solvent, extraction step and time were optimized overall. 21. In this study, Zhishi powder (400 g) was first immersed in 1 L ethanol for 30 minutes and then pulverized into a powder. The mixture was boiled for 1 hour and the process was repeated three times. The aqueous extracts were combined and concentrated in a vacuum rotary evaporator and dried in a vacuum oven to yield 132 g of dry extract. The extract (1 g) was diluted to 100 mL with 50% methanol and filtered through a 0.22-μm microporous membrane before experiment 11. Then, the specific oral daily dose of tamping in rats was diluted with deionized water in a concentration of 6g/kg extract (18g/kg crude drug) 9, 11.

### Animals

On the choice of subject, it's obvious that rats are resistant to APAP toxicity compared with mice. However, According to the reported literature 22-24, rats also have significant oxidative stress, even apoptosis and liver damage response at high doses (>1 g/kg) of APAP even under resistant state. Additionally, the use of rats for metabolomics and organ damage studies exhibit obvious advantages over mice 25, 26. Thus, forty male Sprague-Dawley rats (180 ± 20 g) at age of six weeks old were purchased from Beijing Vital River Laboratory Animal Technology Co., Ltd (SCXK 2016-0011). Experiments were conducted in a laboratory without specific pathogens (SPF) according to the “Guidelines for the Care and Use of Laboratory Animals” adopted and promulgated by the Chinese Ministry of Health.

Every 5 animals are kept in an independent cage in a room of a temperature of 22-25 ° C, fixed humidity, free food and water, and every 12 hours of light/dark cycle (lighting from 8:00 to 20:00) condition. Animal experiment program approved by A the Animal Center of the Institute of Basic Theory, China Academy of Chinese Medical Sciences.

### Rat liver injury model

The APAP suspension was prepared by dissolving acetaminophen in warm phosphate buffered saline (PBS, pH 7.2). APAP-induced acute liver injury was established by intragastric administration of a toxic dose of 2 g / kg body weight of APAP suspension. The newly prepared APAP suspension was used in each experiment 27. After all animals were sacrificed, AST, ALT and GSH were measured in rat using the assay kit to determine the toxicity of APAP-administered rats.

### Experimental design

Forty rats were randomly divided into 4 groups, 10 in each group, after adjusting for one week in laboratory conditions. The experiment is grouped as follows: Rats of RES treatment group and Zhishi treatment group were treated with Zhishi and RES respectively, and APAP was administered for seven consecutive days from day 7. The APAP-induced model group was not given Zhishi or RES, and the control group was given the same amount of normal saline for 7 consecutive days 27. Administration time have evaluated in our previous study. Seven days of zhishi administration is to obtain stable plasma concentrations of various components and their effects on endogenous metabolites 9, 11. The APAP-induced rats received 15mg/kg of RES by oral gavage once a day 28. Zhishi extract (6g/kg extract) was administered by gavage once a day.

### Pre-processing of plasma and liver sample

Blood samples were collected using heparin anticoagulated blood vessels, and the supernatant was separated by centrifugation at 10,000 rpm for 15 minutes and stored at -80 °C for subsequent analysis. Each 1 mL of plasma sample was mixed with 3 mL of acetonitrile and vortexed for pre-analytical treatment. After centrifugation at 10,000 rpm for 10 minutes, the supernatant was taken and concentrated to dryness at 37 °C. The dried concentrate was redissolved in 1 mL of methanol under the action of an ultrasonic extractor prior to analysis. Finally, the precipitate was filtered through a 0.22-μm polytetrafluoroethylene filter after centrifuging again at 10,000 rpm for 10 minutes, and the supernatant was taken for instrumental analysis and stored at 4 °C 9.

For liver sample, approximately 0.3g of liver was homogenized in 10 volumes of chloroform/ methanol (3:1, v/v). Then liver was ultrasonically processed for 1h at 60°C. The extract of chloroform was dried under a stream of nitrogen. The supernatant was taken after the dried sample was redissolved in 100 μL of isopropanol/acetonitrile (1:1, v / v), and filtered to obtain an analytical sample by filtration through a 0.22-μm polytetrafluoroethylene filter. Each sample was extracted in parallel three times, stored at 4 ° C, and finally analyzed by UPLC-Q-TOF-MS.

### Detection of liver activity-related serum markers

After experiment period, blood was collected and all animals were sacrificed. The blood was centrifuged at 3500 rpm for 10 minutes to obtain a serum sample. Determination of ALT, AST and GSH activity in serum was using enzyme-linked immuno sorbent assay (Elisa) kit (Nanjing Jiancheng Bioengineering Institute).

### Liver histology

Histopathology of liver injury was studied. Liver tissue was fixed in a formalin solution containing 10% neutral buffer and dehydrated in graded alcohol. The tissue fixed in the formalin solution was embedded in paraffin and cut into 4 μm thick sections. It was then dewaxed in xylene on a conventional glass slide, rehydrated in low concentrations of ethanol, and finally stained with hematoxylin and eosin (H&E). The pathological changes of liver tissue were determined according to histopathological methods.

### UHPLC-QE Orbitrap HRMS instrumentation and conditions

Metabolite analysis was performed on an Acquity UPLC HSS T3 column (1.8 μm 100 x 2.1 mm) using an UltiMate 3000 high performance LC system coupled to Q Exactive MS. Mobile phase A (water contained 2 mmol/L ammonium formate, v/v, 0.1 % formic acid) and mobile phase B (methanol) were utilized at a flow rate of 0.3 mL/min. From 0 to 2 min, A was decreased to 80 % from 70 %. From 2 to 5 min, A was decreased to 55 %. From 5 to 6.5 min, A was decreased to 40 %. From 6.5 to 12 min, A was decreased to 35 %. From 12 to 18 min, A was decreased to 0. The mobile phase gradient was as follows: The temperature of the column oven was set to 40 °C and the temperature of the autosampler was set at 4 °C. The injection volume was 1 μL.

The mass spectrometry ESI source was run in positive ion mode. The parameters are as follows: capillary temperature, 320 °C; source voltage and spray voltage, 3.5 kV; sheath gas (nitrogen) flow, 30 arb; and aux gas flow, 10 arb. Data were acquired using full MS scan (resolution, 70,000; AGC target, 1 × 106; maximum IT, 120 ms; scan range, m/z 120-1800).

### Reliability assessment of LC-MS-based metabolomic methods

Quality control (QC) samples were used to evaluated the stability and repeatability of LC-MS based metabolomics method. Each QC sample was prepared by sequentially combining 5 samples of the same volume (100 μL) in the order of analysis. To ensure stable operation of the system method, all of the combined QC samples for running all QC samples made by every five samples before analysis. During the analysis run, after five samples were tested, the combined QC samples of the five samples were taken.

### Optimization of UHPLC-QE Orbitrap HRMS system conditions

In order to obtain the best peak shape and resolution in the LC chromatogram, several different mobile phases were screened in the experiment. Acetonitrile contain 0.1% formic acid (v / v) as the organic phase and water containing 2 mmol / L ammonium formate as the aqueous phase were selected to obtain sufficient separation efficiency. Water and acetonitrile containing 2 mmol / L ammonium formate and 0.1% formic acid (v / v) were finally selected as the mobile phase to obtain sufficient separation efficiency and good peak symmetry for the metabolite.

To improve sensitivity of the QE Orbitrap HRMS system when capturing precursor, daughter and fragment ions of most compounds, mass spectrometer ion source parameters were optimized in the experiment. The best conditions for the QE Orbitrap HRMS were as follows: capillary temperature, 350 °C; source voltage and spray voltage, 3.7 kV; sheath gas (nitrogen) flow, 28 arb and auxiliary gas flow, 8 arb.

### Cell culture

The BRL-3A cell line was purchased from the Shanghai Institute of Biochemistry and Cell Biology, Chinese Academy of Sciences (Shanghai, China). Place the cells in DMEM medium (GIBCO) containing 10% (v / v) FBS (GIBCO), penicillin (100 U / mL) and streptomycin (100 U / mL) in a 37 ° C, 5% CO 2 cell incubator to cultivate.

### Cell viability assay

The cells were first plated to a 96-well cell culture plate at a density of 1.0 × 104 cells/well. Then, Zhishi was added to a 96-well cell culture plate (Sigma, St. Louis, MO, USA) of BRL-3A entering the logarithmic growth phase, and incubated at 37°C for 48 hours in a 5% CO 2 incubator. The cells were washed twice with PBS, and then the new medium, Zhishi and APAP, were added to a 96-well cell culture plate. The cells were incubated with Zhishi, APAP in a cell incubator at 37 ° C, 5% CO 2 for 24 hours. Next, add 10 μl of cck8 reagent (Dojindo, Tokyo, Japan) to each well, then incubate for 3 hours in a cell culture incubator. Finally, the absorbance of each well was measured by microplate reader at 450nm.

### Quantitative Rea-ltime-PCR analysis

First let the cells fully lyse with Trizol for 5 min at room temperature, centrifuge to get total RNA in the supernatant. Under the effect of M-MLV reverse transcriptase (Promega) in oligo-dT primers (QIAGEN), first strand cDNA synthesis by 3 micrograms of total RNA. The synthesized cDNA was amplified based on PCR. Semi-quantitative detection was performed by Taq polymerase (Bioneer, Daejeon, South Korea) by RT-PCR. Using SYBR Green PCR Master Mix (Applied Biosystems, Foster City, CA) and ABI 7500 Fast Real-Time PCR System (Applied Biosystems) for real-time PCR. Synthesized by Invitrogen gene-specific primers, the primer sequences are as follows: P53: AGCTACACCGTGGCCTCTGTC (forward) and CCAGGTGGAGGTGTGGAGGTG (reverse), Casp 3: ACGAACGGACCTGTGGACCTG (forward) and TTCCAGCTTGTGCGCGTACAG (reverse), Bax: GCCCACCAGCTCTGAACAGA (forward) and TCAGCTGCCACACGGAAGAA (reverse), Sirt1: CCGGACAGTTCCAGCCATCT (forward) and TGGCAAGTGGCTCATCAGCT (reverse), GAPDH: TGGAGTCTACTGGCGTCTT (forward) and TGTCATATTTCTCGTGGTTCA (reverse). Standardize mRNA expression data with GAPDH as an internal reference.

### Western blot analysis

PMSF and phosphatase inhibitor (Amresco, Houston, TX, USA) were first added to the cell sample. After that, cells were lysis using RIPA lysis buffer (Beyotime, Shanghai, China). The cell sample was centrifuged at 13,000 rpm and 4 °C for 20 minutes, and Take the supernatant for later use. The protein concentration in the supernatant was determined using a BCA Protein Assay Kit (Pierce Biotechnology, Rockford, IL) and diluted to obtain the same total concentration of protein. Add β-mercaptoethanol sample buffer to the lysate and boil for 5 minutes. The protein sample was then subjected to an SDS-PAGE experiment and finally electrotransferred to a polyvinylidene fluoride membrane (GE Healthcare). After blocking in PBS containing 5% skim milk, the membrane was immersed in an appropriate dilution of antibody solution and incubated overnight at 4 °C, as follows: anti-SIRT1 (Millipore, Temecula, CA. 1:3000 diluted), anti-p53, anti-BAX, anti-caspase-3, anti-p-MEKK7, anti-MEKK7, anti-p-JNK1, anti-JNK1 (Cell Signaling Technology, 1:1000 dilution), anti-AMPK and anti-GAPDH (Abcam, 1:1000 dilution), PBS-Tween 20 washing membrane with 0.05 %, and the dilution of HRP in 1/5000 of the second incubation for 1 hour at room temperature, resistance to detect immune reactivity use ECL detection system (GE Healthcare), exposure film in more than one point in time to ensure that the image is not saturated, normalizing changes in protein expression to GAPDH expression levels.

### Statistical analysis

Data for each of the three parallel experiments is expressed as mean ± standard deviation. Differences between groups were assessed by one-way analysis of variance (ANOVA), and then Tukey's multiple comparison analysis was performed in Graphpad Prism 6. Differences were considered statistically significant at *indicates a significant difference (p < 0.05), **indicates a highly significant difference (p < 0.01), ***indicates an extremely significant difference (p < 0.001).

## Results

### Chemical constituents of Zhishi extract

RRLC-QqQ-MS was performed to quantify the chemical constituents of Zhishi extract. The corresponding method validations are summarized in previous studies 21. The total percent of 32 chemical constituents is 64.6 % in the Zhishi extract. As shown in Supplementary Table S2, 32 phenolic constituents were determined. In addition, 75 chemical constituents in the extract were characterized based on their fragmentation behaviors, accurate mass and retention times in UPLC-Q-TOF-MS system. The 75 identified chemical constituents were briefly identified, including 29 coumarins, 25 flavones and flavones glycoside, 14 flavanones and flavanone glycoside, 3 flavonols, 2 limonoids and glycosides, 1 anthocyanin and 1 abscisic acid (Table S3) 5.

### Zhishi prevent liver injury caused by APAP in rats

**Fig 1:**
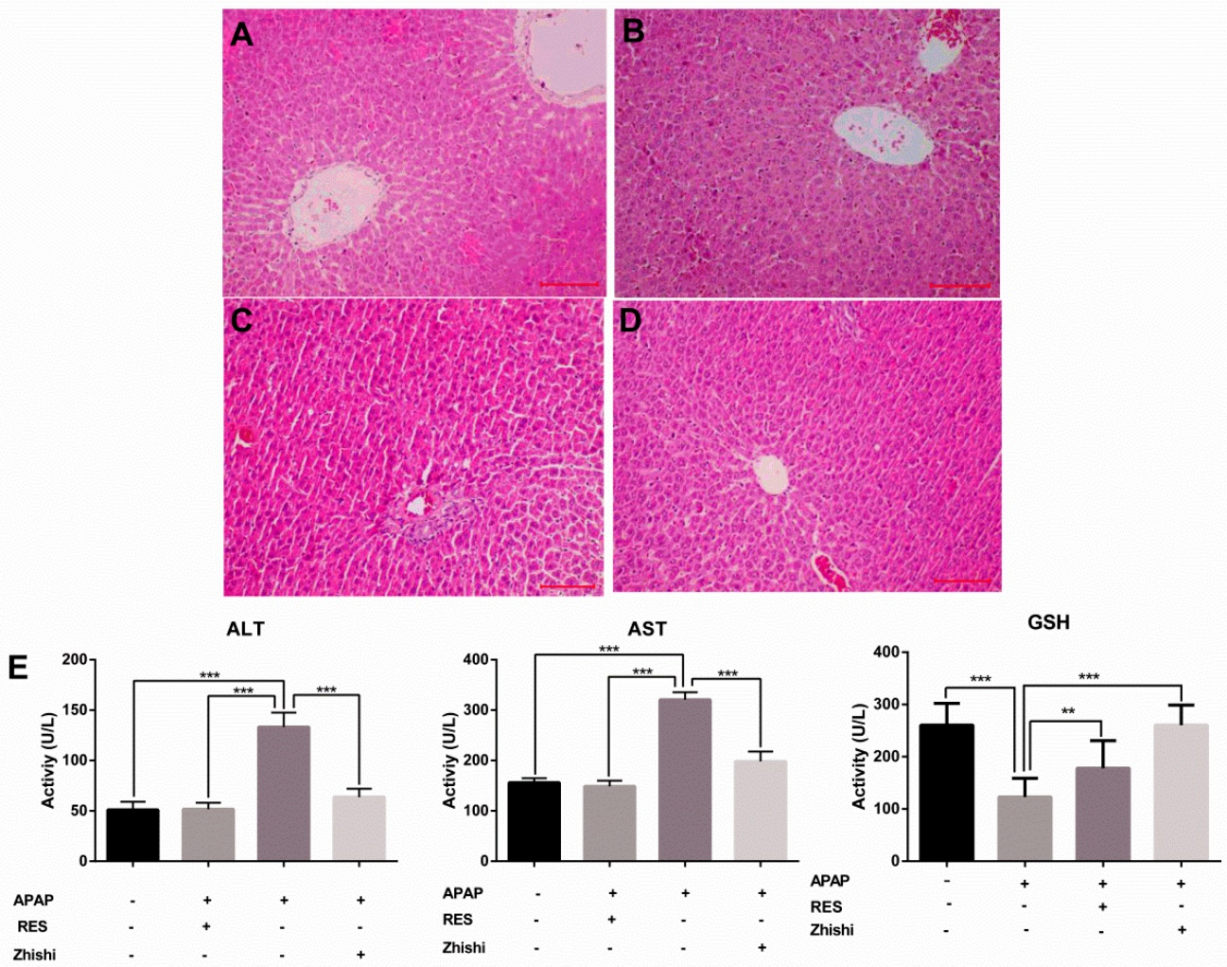
The biochemical markers in APAP-induced liver injury rat. The serum levels of ALT, AST, GSH and the histological changes in the liver of rats in different groups were shown. (A) Control group; (B) APAP induced model group; (C) Zhishi treatment group; (D) Resveratrol treatment group; (E) The activity of ALT, AST and GSH in different groups. The results are presented as mean ± S.D. of experiments. (n=10 per group, 200x magnification, scale bars=100μm)), *p < 0.05, **p < 0.01, ***p < 0.001.

In this study, we evaluated the effects of Zhishi liver injury induced by excess APAP in rats. The circulating levels of hepatic enzymes AST, ALT, GSH and the histopathological studies are widely used for evaluating hepatic injury. In Figure 1(E), ALT and AST levels increased after the administration of APAP to 133.31 ± 14.269 and 320.92 ± 14.627 U/L from 51.83 ± 6.183 and148.98 ± 11.350 U/L in controls. As expected, serum ALT, AST levels were significantly decreased in the group treated with RES and Zhishi. The GSH levels in the APAP group were markedly decreased. After Zhishi or RES treatment, GSH levels were increased respectively. Likewise, in Figure 1, histopathological studies were performed to study the effect of Zhishi on rats with overdosed APAP. Hematoxylin and eosin (H&E) stained sections of Rat liver tissue showed that APAP caused remarkable liver injury compared with the control group, with the hepatocyte necrosis and irregularly arrangement. All the features suggested that Zhishi significant prevent APAP-induced liver injury.

### Hepatic metabolomics study of APAP-induced liver injury and Zhishi treatment

Metabolomics, a technique for comprehensive analysis of small molecule metabolites in cells, tissues, or whole organisms, has been increasingly used to assess the therapeutic effects of drugs 29, 30. The hepatic metabolomics in this study provides a new perspective for the study of metabolism in liver during disease development and treatment. The raw data of hepatic metabolomics obtained by the UHPLC-Q-Orbitrap in the Progenesis QI software in the positive ion mode was subjected to background subtraction, component extraction and peak alignment to obtain visualized standardized data. This approach ensured the smoothness of the multivariate modeling.

**Fig 2:**
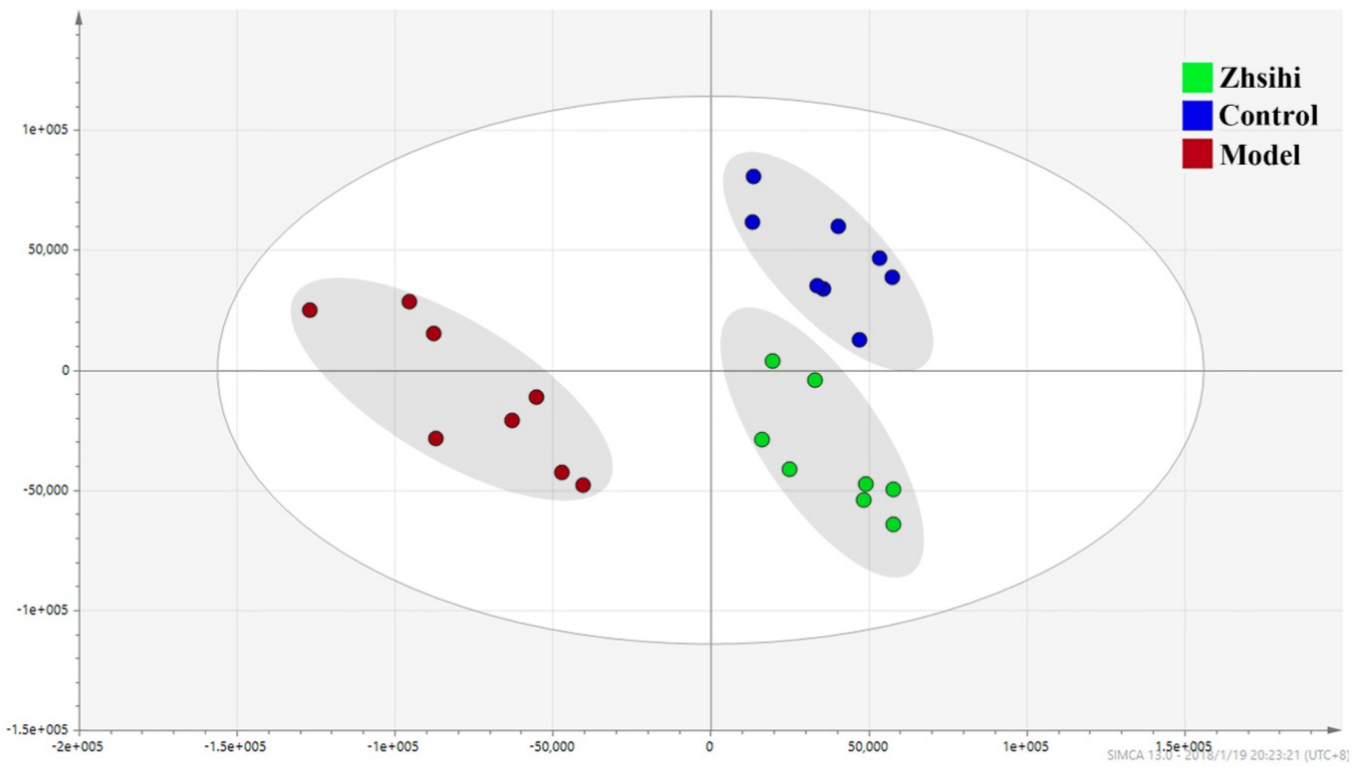
The PCA scores plots between ZS treatment group, model group and control group.

**Fig 3:**
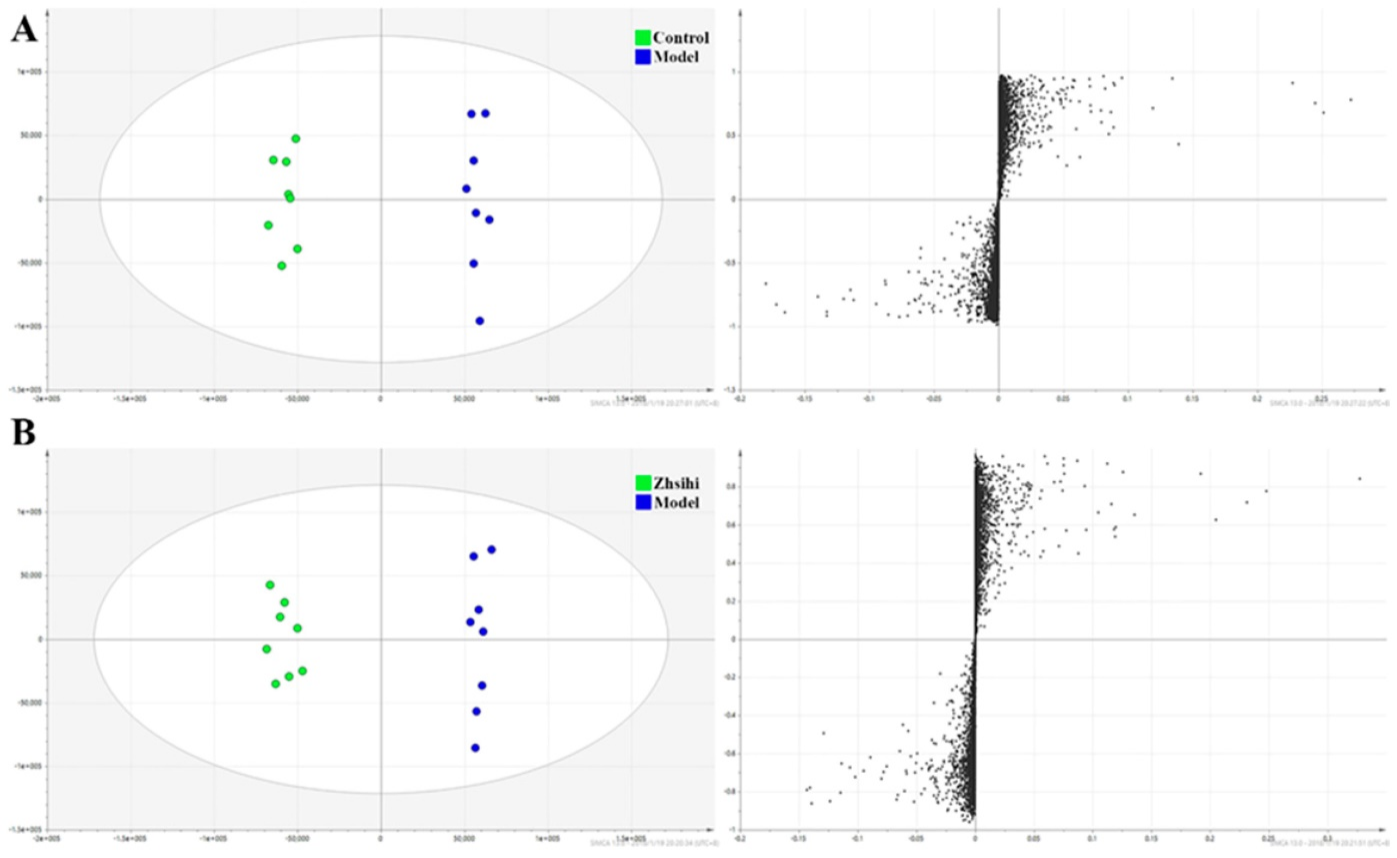
Screening and identification of potential biomarkers. (A) OPLS-DA scores plots and S-plot map between the control group and model group; (B) OPLS-DA scores plots and S-plot map between the Zhishi treated group and model group.

PCA scores plots (Figure 2) showed that the separation between ZS treatment group, model group and control group was obviously significant (R2X=0.588, Q2=0.442). Moreover, the ZS treatment group and control group were close to each other and both were separated from the model group. The OPLS-DA model (Figure 3) was constructed in the experiment to maximize the differences and highlight key variables to find potential markers. A successful distinction between ZS treatment (R2X = 0.746, R2Y = 0.992 and Q2 = 0.983) / control group (R2X = 0.705, R2Y = 0.991 and Q2 = 0.956) and the model group shown in OPLS-DA score plot. The S-map was used to identify key metabolites that contribute to ZS treatment, model group and control group differentiation. From the loading map, various metabolites (VIP values > 1) were identified responsible for the separation between each pair of groups and were considered to be potential biomarkers with common metabolites in both figures.

**Fig 4:**
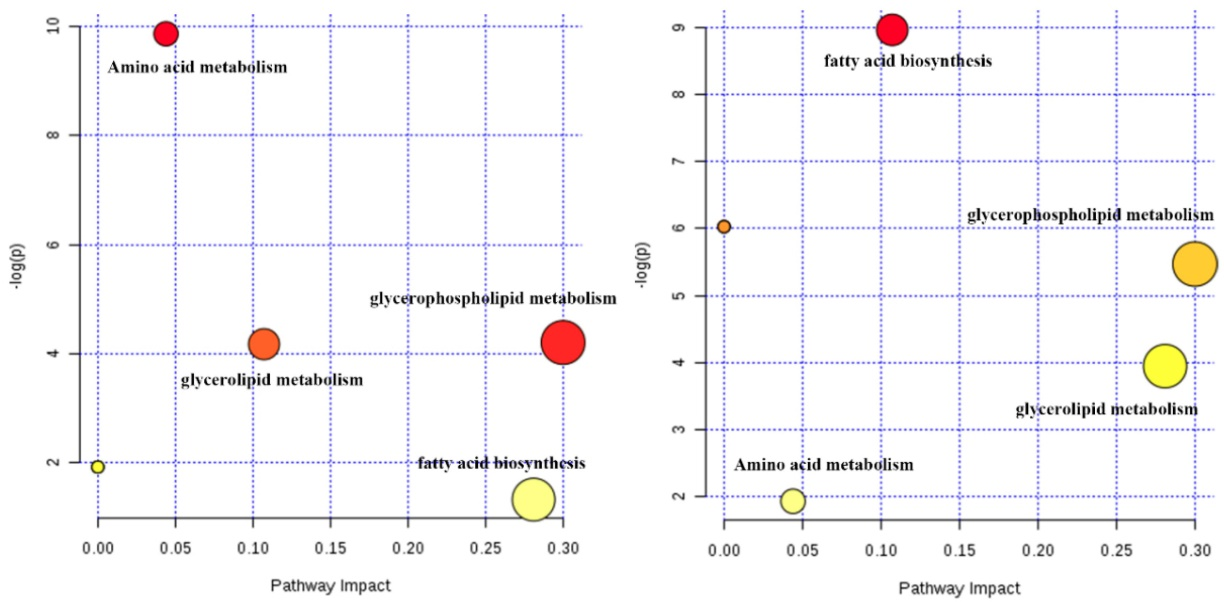
Potential metabolic network of the protective effect of Zhishi in APAP-induced liver injury (between the control group and model group (left) and between the Zhishi treated group (right)).

The potential biomarkers (shown in Table 1), included 13 amino acids, 12 phosphatidylcholines (PC), 11 phosphatidylethanolamines (PE) and 8 other compounds, were tentatively identified from the reference standards. Based on these potential markers and KEGG database, an integrated metabolic network of Zhishi acting on apap-induced liver injury was constructed (Fig. 4) with the use of MetaboAnalyst 3.0 online data analysis software. Fourteen metabolic pathways were disturbed in the ZS treatment, model and control groups. The first three metabolic pathways with the most influential value (over 0.1) in the treatment group were glycerophospholipid metabolism, fatty acid biosynthesis and glycerolipid metabolism.

### APAP-induced cytotoxity and the protective effect of Zhishi on BRL-3A hepatocytes

**Fig 5:**
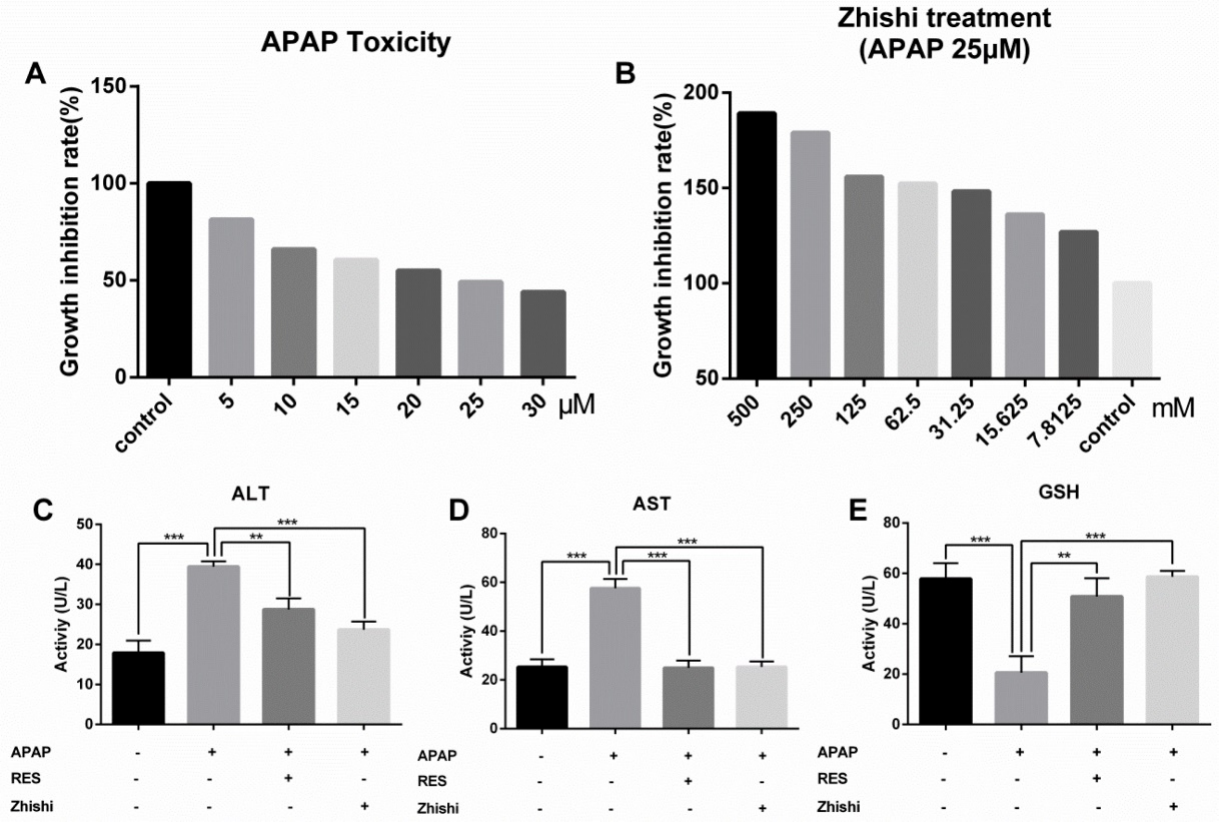
Cell proliferation and toxicity experiments of Zhishi and APAP in BRL-3A cell. (A) Effect of different doses of APAP on BRL-3A cells; (B) Inhibition of APAP-induced cell death by different doses of Zhishi; The ALT (C), AST (D) and GSH (E) levels in the BRL-3A cell injury model. The data were expressed as means ± standard deviation (S.D.) of triplicate dependent experiments. *p < 0.05, **p < 0.01, ***p < 0.001.

We established a BRL-3A cell model to examin the potential effect of Zhishi when APAP overdosed. To examine the cytotoxic effects of APAP, the cytoactive of BRL-3A hepatocytes was assessed by CCK8 assay at concentrations of 30, 25, 20, 15, 10, 5, and 0 µM APAP. As shown in Figure 5A, a reduction in cell viability from 100 % to 49.2 % was observed after incubating with 25 µM of APAP for 24 h (p< 0.01). To determine the effects of Zhishi, the BRL-3A hepatocytes induced by APAP (25 µM) were treated with Zhishi (1000, 500, 125, 62.5, 31.25, 15.625, 7.8125 mM). As is shown in Figure 5B, the mortality rate of the Zhishi (62.5 mM) -treated on APAP (25 µM) induced-BRL-3A hepatocytes was considerably lower than that of the APAP (25 µM) induced-BRL-3A hepatocytes.

According to the cell viability results, the model group was treated with 25 µM of APAP, the Zhishi group was treated with 62.5 mM of Zhishi in addition to 25 µM of APAP, the positive parallel control group was treated with 20 µM of RES 15 in addition to 25 µM of APAP and control group were given the same amount of PBS. The AST and ALT level in the supernatant fluid of cultured BRL-3A hepatocytes was detected using an assay kit. We observed that APAP (25 µM) dramatically increased the AST and ALT levels, but Zhishi reduced these increases (Figure 5C). The GSH, a key substance in detoxification in APAP metabolism, were markedly decreased (20.57±6.512 U/L) in the APAP group. After Zhishi or RES treatment, GSH levels were (58.62±2.365) and (50.86±7.202), respectively, and the Zhishi and RES groups could keep the GSH levels close to the normal level (57.86±6.191 U/L) (Figure 5C). This recommends that Zhishi has a strong prevention on APAP-induced BRL-3A hepatocytes apoptosis.

### Activation of AMPK/SIRT1 pathway by Zhishi to reduce cell damage

**Fig 6:**
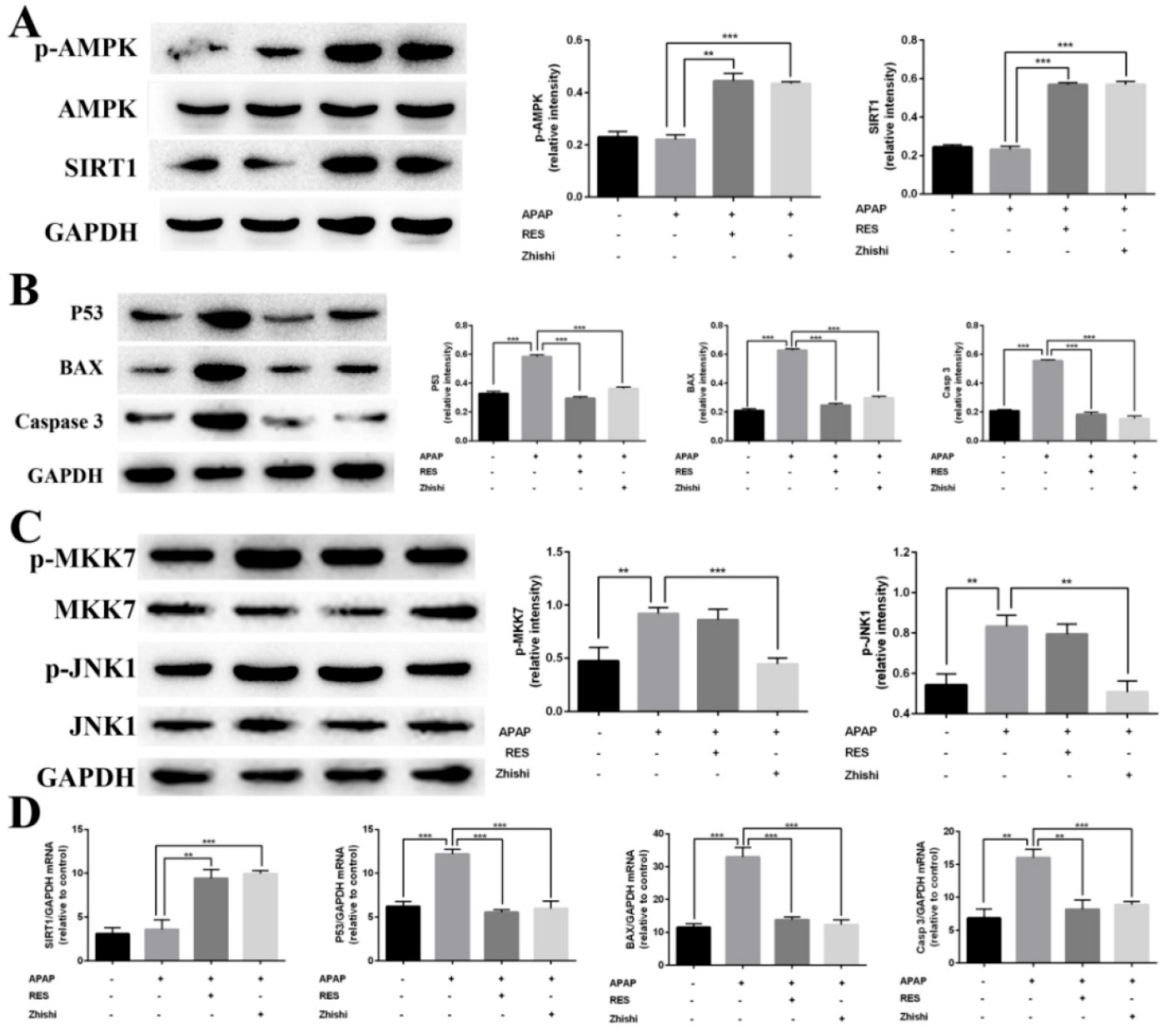
Inhibitory effects of Zhishi and resveratrol on PUMA/AMPK-SIRT1/JNK1 associated hepatocyte apoptosis in APAP-induced BRL-3A Cells. (A) The levels of protein related to AMPK signaling pathway; (B) The levels of protein related to P53 signaling pathway; (C) The levels of protein related to MAPK-JNK pathway; (D) The levels of protein mRNA expression related to SIRT1-p53 pathway. The data were expressed as means ± standard deviation (S.D.) of triplicate dependent experiments. *p < 0.05, **p < 0.01, ***p < 0.001.

Previous studies shown that SIRT1 is closely associated with apoptosis of hepatocytes induced by APAP 31. RES, a well-known antioxidant, was shown to reduce liver injury and stimulate SIRT1 activity. To investigate protective effects on APAP-induced BRL-3A hepatocytes of Zhishi, the mRNA expression levels of SIRT1 and the protein levels of AMPK, p-AMPK and SIRT1 were investigated by qPCR and western blot analysis. Compared to the model group in Fig. 6A, the expression of p-AMPK and SIRT1 were significantly elevated on protein level. The mRNA expression levels of SIRT1 (Fig. 6D) in BRL-3A hepatocytes treated with Zhishi were significantly enhanced (p < 0.001) as to the model group. As a consequence, our results indicated that Zhishi stimulates AMPK signaling and increases SIRT1 expression in an APAP-treated cell model.

### Inhibition of Zhishi on P53-associated hepatocyte apoptosis

To examine the molecular mechanism of APAP-induced apoptosis, p53 changes were evaluated in BRL-3A hepatocytes. As shown in Figure 6B and D, mRNA and protein expression of p53 was induced by APAP. Both qPCR and western blot results demonstrated that RES or Zhishi treatment significantly reversed the upregulation of p53 expression as anticipated. P53, a nuclear transcription factor that transactivates genes involved in apoptosis, can trigger apoptosis by interacting with Bax 32. Furthermore, apoptosis can be triggered via caspase-9 (Cas9) activation and might also assist in APAP-mediated hepatocyte apoptosis. The mRNA expression of pro-apoptotic markers (Bax and Cas3) were shown to be significantly downregulated in cells treated with the Zhishi or RES for 24 h (Figure 6D) compared to the model group. In Figure 6B, the protein levels of BAX and Cas3 in BRL-3A cells showed that down-regulation of these pro-apoptotic genes after treatment with Zhishi or RES. Collectively, these results demonstrated that Zhishi prevents hepatocyte apoptosis induced by APAP by regulating p53-associated hepatocyte apoptosis.

### The inhibitory effect of Zhishi on the JNK pathway

As JNK1 is involved in p53-upregulated modulator of apoptosis (PUMA), we investigated its activity in BRL-3A cellular death induced by APAP 33. Since MKK7 is an important upstream mediator in the JNK1 signaling pathway, we investigated the effect of MKK7 and JNK1 expression in APAP-induced BRL-3A hepatocyte apoptosis. As expected, the expression of phosphorylated MKK7 and JNK1 was apparently upregulated (Figure 6C) after treated with APAP for 24 h in BRL-3A. Interestingly, their protein levels in the RES group has no significant difference compared with model group. In contrast, p-MKK7 and p-JNK1 was significantly decreased after treated with Zhishi for 24 h (Figure 6C). This indicated that Zhishi could attenuate APAP-induced cell death via the JNK1 signal pathway. Although RES can resist APAP-induced hepatocyte apoptosis by down-regulating PUMA, it has no role in its regulation.

## Discussion

In the most grievous cases, drug-induced liver injury (DILI), the leading cause of acute liver failure in Western countries, can directly cause liver necrosis or lead to death. Drug hepatotoxicity can be ordinary (predictable), as with APAP, or distinctive (unpredictable) 34. Acetaminophen is one of the most commonly used antipyretic analgesic and anti-inflammatory drugs in clinical practice. A safe dose for each healthy adult is generally considered to be less than 4,000 mg for healthy adult per day 35. However, acute higher concentrations of use or long-term excessive accumulation could give rise to liver damage and possibly develop to liver failure. Recent studies implicated abnormal lipid metabolism strongly linked to hepatocyte apoptosis in rat liver injury caused by excessive APAP. In vivo studies exhibited that APAP-induced liver injury also activated the p53 signaling pathway in vivo and caused apoptosis in hepatocytes of rats treated with toxic doses of APAP 36. Consistent with this observation, our studies show a novel therapeutic mechanism of Zhishi in modulating both apoptosis pathways and host lipid metabolism during liver injury.

It is well-established that the CYP-mediated metabolic reactions convert APAP into NAPQI, a toxic metabolite which causes hepatotoxicity. Then, NAPQI consumes GSH in hepatocytes and, when depleted, binds to cellular proteins, eventually leading to liver damage and tissue necrosis 37. Toxic metabolic activation of CYP to APAP is a critical step in excess APAP-induced cell death 38. Intriguingly, we reported that Zhishi, a *Citrus* plant which is used as both medicine and food, have a wide range of research interests due to a great many dietary phenolic substances, which have important therapeutic effects on human health 5. Zhishi exhibits significant inhibition of the metabolic processes of CYP enzymes that produce toxic metabolites 9, especially for CYP2E1, CYP3A4, and CYP1A2 39. Thus, our previous studies have shown that Zhishi may have the ability to prevent APAP-induced liver injury. We further studied the effects of Zhishi on rats with liver injury caused by excessive APAP.

Our current results indicate that 2 g / kg of APAP can significantly increase the serum ALT and AST levels of rats. Besides, serious hepatic necrosis and inflammation appeared in histopathological examination, which are the common measures taken to assess APAP toxicity in the lab (Figure 1). When Zhishi was administered for seven consecutive days before a toxic dose of APAP, hepatotoxicity of rats caused by excessive APAP is alleviated, and this protection was indicated by lower serum AST and ALT levels, higher GSH activity and markedly less liver damage. The mechanisms of DILI are not always certain, but mitochondrial dysfunction often occurs in their published research. Importantly, drug-induced mitochondrial dysfunction is a term of art that usually includes structural damage to mitochondria and changes and disturbances in different metabolic pathways. Similarly, liver damage caused by excessive APAP may be related to lipid metabolism and changes in intracellular signaling proteins, such as JNK1, AMPK and p53 signaling pathways, which leading to mitochondrial permeability transition (MPT), excessive oxidative stress, Abnormal changes in mitochondrial membrane potential, mitochondrial ATP synthesis and release blocked, eventually cause tissue necrosis 40. At the time of APAP overdose, liver triglycerides accumulate in the lipid vacuoles around the nucleus of the hepatocytes, and denaturation of the microbubble fat mixture of some hepatocytes may cause lesions in the liver. Our results demonstrated the protective effect of Zhishi on the maintenance of hepatic lipid metabolite homeostasis and inhibition of apoptosis-related protein expression in APAP overdose.

**Fig 7:**
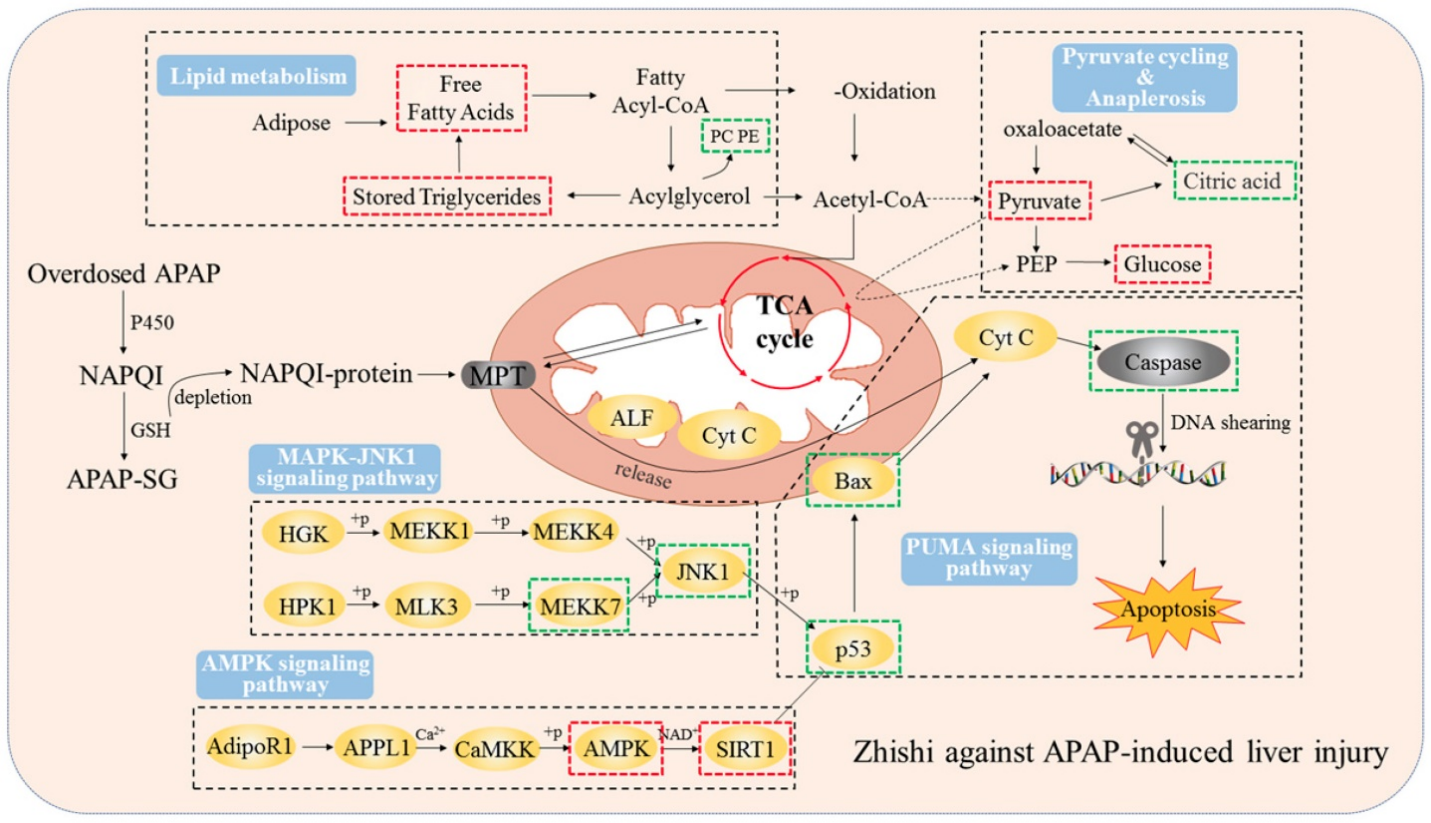
Zhishi protects against liver damage in APAP-induced liver injury. These include the regulation of liver cell lipid metabolism by mitochondrial damage and oxidative stress, maintenance of energy metabolism, and inhibition of apoptosis-associated proteins. The red box represents the factor being enhanced and the green box represents the weakened factor when pretreated by Zhishi.

The rate and amount of lipid synthesis and accumulation are critical to liver health, rest with the age, gender, eating habits and health condition of the individual. Liver lipid components are almost steady in healthy individuals, which is independent of influencing factors 35. Lipids have many important functions. They are involved in various biochemical reactions and almost all metabolic pathways in the body. Therefore, the alteration of lipids would affect cell function and reflect the state of cells and tissues 41. However, drugs such as APAP can alter the normal process of lipid metabolism in liver. The content of TG, DG, PC, PE and CL in liver tissue increased or decreased while the liver concentrations of AST and ALT increased with the APAP overdose compared to the group treated with Zhishi (shown in Figure 7). The analysis of UHPLC-QE Orbitrap HRMS data revealed the changes of liver lipids of liver tissue in response to APAP overdose and Zhishi pretreatment. Based on the results of metabolomics studies, the level of many metabolites of PC/PE in liver tissue are raised in APAP overdose, but the rats in the Zhishi treatment group demonstrated an increased tendency toward normal levels compared with the control group. This result is similar to previous research data. 41. Although the earlier studies have shown that lipid storage promotes the healing of chronic wounds and the loss of lipid compartmentalization and TG storage capacity, produces significant lipotoxicity and can exacerbate liver damage 42. This finding indicates that there is a certain benefit to the recovery of the liver TG pool. Our results attractively revealed that TG levels in the liver accumulated back to normal in Zhishi-treated group as well as in the control group, suggesting a potential role for protective effect in liver injury caused by overdosed APAP. Therefore, the disorder of lipid metabolism (fatty acid biosynthesis, glycerolipid metabolism and glycerophospholipid metabolism) in liver injury induced by APAP might be reversed due to the protective effect of Zhishi in APAP overdose. Our results suggesting that the prevention of hepatotoxicity of APAP is closely related to the regulation of lipid metabolism. The changes of the cellular signaling proteins during the development of this protective effect in the liver necrosis process requires further detailed investigation.

Consistent with previous findings 9, we observed that Zhishi exerted an important protective effect on APAP-induced liver injury by decreasing apoptosis and increasing cell viability in BRL-3A hepatocytes in vitro. This finding demonstrated Zhishi's prevent against APAP-induced hepatotoxicity in rats along with the regulation of lipid metabolism involved in the APAP toxicity. P53 is a famous tumor suppressor and closely related to the apoptotic process. Protein of Bcl-2 family is a sub-target protein of p53 43. PUMA can induce mitochondria permeability conversion process by releasing p53 and activating BAK and Bax through Bcl-XL / p53 complex, ultimately leads to apoptosis. At the same time, caspase-3 effectors that cleave many regulatory proteins and structural proteins are activated by caspase-8, leading to apoptosis. Based on these studies, mitochondrial membrane permeabilization caused by overdosed APAP may activate PUMA and Bax apoptotic factors, causing cytochrome c released to the cytoplasm from the mitochondria, triggering the caspase cascade leading to apoptosis 44. All of the above results indicate that the SIRT1-p53 pathway may of great importance to BRL-3A apoptosis and the ability of RES to prevent liver injury caused by APAP. Previous studies have shown that JNK1-mediated Bcl-2 phosphorylation can interfere with the binding of Bcl-2 to p53 protein involved in ER stress-mediated autophagy and apoptosis 45. In the results of our current study, as shown in Figure 7, these clearly showed that Zhishi prevents APAP-induced liver injury by blocking the apoptosis process and facilitating liver regeneration compared to RES's protective effect against liver injury by inducing the SIRT1-P53 pathway alone.

## Conclusions

To sum up, this study explained that Zhishi significantly prevent the liver injury induced by APAP. The reversed effect of lipid metabolism disorder in APAP-induced liver injury caused by Zhishi was confirmed the nontargeted metabolomics study of liver tissue. Moreover, down-regulation of apoptotic markers and upregulation of anti-apoptotic markers at the transcription and expression level further confirmed the effectiveness of Zhishi used to protect against APAP-induced liver injury. It was clearly demonstrated that Zhishi exerts hepatoprotective effects by regulating lipid metabolism combined with conducting multi-targeted signaling pathway, which could lead to improving the clinical outcomes of liver injury prevention.

## Supplementary Material

Supplementary figure and tables.

## Figures and Tables

**Table 1:** Key differential metabolites detected by UHPLC-QE Orbitrap HRMS

No.	Metabolites	Metabolic pathway	RT	Formula	VIP value	^a^fold change	^b^P-value
1	Leucine	Amino acid metabolism	4.81	C6H13NO2H	6.17083	1.58	1.12E-02
2	Tryptophan	Amino acid metabolism	4.89	C11H12N2O2H	5.18573	1.19	3.31E-02
3	Valine	Amino acid metabolism	4.93	C5H11NO2H	4.68174	0.58	1.17E-02
4	Isoleucine	Amino acid metabolism	4.97	C6H13NO2H	4.79804	-1.63	1.94E-02
5	L-methionine	Amino acid metabolism	5.22	C5H11NO2SH	8.14658	0.84	3.86E-03
6	Proline	Amino acid metabolism	5.36	C5H9NO2H	4.76387	1.77	3.13E-02
7	Tyrosine	Amino acid metabolism	5.48	C9H11NO3H	8.40403	-1.48	3.69E-02
8	Alanine	Amino acid metabolism	5.92	C3H7NO2H	5.26734	-0.66	3.74E-02
9	Cysteine	Amino acid metabolism	6	C3H7NO2SH	6.11844	0.45	3.4E-02
10	Homoserine	Amino acid metabolism	6.15	C4H9NO3H	5.8804	0.71	3.75E-02
11	Threonine	Amino acid metabolism	6.15	C4H9NO3H	8.08608	1.11	3.3E-02
12	Glycine	Amino acid metabolism	6.16	C2H5NO2H	5.13134	1.29	2.92E-02
13	Serine	Amino acid metabolism	6.41	C3H7NO3H	7.69839	0.65	1.97E-02
14	Dimethylglycine	Amino acid metabolism	2.92	C5H13NOH	8.12094	-1.06	1.61E-02
15	Ethanolamine	Amino acid metabolism	5.05	C2H7NOH	7.09972	0.78	3.23E-02
16	N-acetylornithine	Amino acid metabolism	5.38	C7H14N2O3H	8.83872	0.42	2.35E-02
17	Hydroxyproline	Amino acid metabolism	5.99	C5H9NO3H	5.95346	0.71	3.02E-02
18	Sarcosine	Energy metabolism	5.73	C3H7NO2H	4.90247	1.43	3.26E-02
19	Creatine	Energy metabolism	5.79	C4H9N3O2H	5.69281	1	1.15E-02
20	Glucosamine 6-phosphate	Energy metabolism	6	C6H14NO8PH	8.28821	-1.17	1.17E-02
21	Glucose 6-phosphate	Energy metabolism	6	C6H13O9PH	7.45167	1.68	2.35E-02
22	PC(0:0/22:6(4Z,7Z,10Z,13Z,16Z,19Z))	Lipid metabolism	4.73	C30H51NO7P	5.05299	0.96	3.45E-02
23	PC(16:0/14:0)	Lipid metabolism	8.87	C38H77NO8P	8.74988	1.73	4.21E-03
24	PC(16:0/16:0)	Lipid metabolism	9.47	C40H81NO8P	4.99775	1.58	4.12E-02
25	PC(16:0/17:1(9Z))	Lipid metabolism	9.25	C41H81NO8P	5.20716	-0.97	3.64E-02
26	PC(16:0/9:0(CHO))	Lipid metabolism	6.11	C33H65NO9P	7.12949	0.68	2.18E-02
27	PC(16:1(9Z)/14:0)	Lipid metabolism	8.45	C38H75NO8P	8.84866	0.68	2.93E-02
28	PC(16:1(9Z)/16:0)	Lipid metabolism	8.98	C40H79NO8P	4.69183	0.74	2.99E-02
29	PC(20:4(5Z,8Z,11Z,14Z)/0:0)	Lipid metabolism	4.96	C28H51NO7P	7.70826	1.42	3.35E-02
30	PC(20:4(5Z,8Z,11Z,14Z)/14:0)	Lipid metabolism	8.53	C42H77NO8P	7.56503	-1.29	2.75E-02
31	PC(20:4(5Z,8Z,11Z,14Z)/15:0)	Lipid metabolism	8.87	C43H79NO8P	4.66881	1.08	4.48E-03
32	PC(20:4(5Z,8Z,11Z,14Z)/16:1(9Z))	Lipid metabolism	8.82	C44H79NO8P	8.42831	0.99	3.13E-02
33	PC(20:4(5Z,8Z,11Z,14Z)/17:0)	Lipid metabolism	9.34	C45H83NO8P	5.89928	1.62	4E-02
34	PE(16:0/16:0)	Lipid metabolism	9.61	C37H75NO8P	5.87224	1.64	1.4E-02
35	PE(16:0/18:1(9Z))	Lipid metabolism	9.57	C39H77NO8P	5.97014	1.67	4.48E-04
36	PE(17:0/17:0)	Lipid metabolism	10.21	C39H79NO8P	6.44635	0.5	2.69E-02
37	PE(18:0/0:0)	Lipid metabolism	6.38	C23H49NO7P	6.0993	-0.83	1.65E-02
38	PE(18:0/18:1(9Z))	Lipid metabolism	9.32	C41H81NO8P	6.19751	0.55	2.12E-03
39	PE(18:2(9Z,12Z)/16:0)	Lipid metabolism	9.16	C39H75NO8P	5.79491	0.41	2.49E-02
40	PE(22:4(7Z,10Z,13Z,16Z)/0:0)	Lipid metabolism	5.82	C27H49NO7P	8.34833	-0.91	4.5E-03
41	PE(22:6(4Z,7Z,10Z,13Z,16Z,19Z)/0:0)	Lipid metabolism	4.99	C27H45NO7P	5.45747	-0.76	4.62E-04
42	PE(22:6(4Z,7Z,10Z,13Z,16Z,19Z)/16:0)	Lipid metabolism	8.97	C43H75NO8P	7.88728	1.54	4.23E-02
43	PE(P-16:0/0:0)	Lipid metabolism	5.89	C21H45NO6P	8.90852	1.55	4.11E-02
44	PE(P-16:0/22:4(7Z,10Z,13Z,16Z))	Lipid metabolism	9.99	C43H79NO7P	6.90864	0.78	1.98E-02

a Only metabolites with variable influence on projection (VIP) values of greater than 1.0 and p-values of less than 0.05 were deemed statistically significant. b Fold change was calculated as the logarithm of the average mass response (area) ratio between the two classes (i.e., Fold change = lg[Zhishi/model]).

## Abbreviations

APAP: Acetaminophen
PUMA: p53-upregulated modulator of apoptosis
NAPQI: N-acetyl-p-benzoquinone imine
GSH: glutathione
TCMs: traditional Chinese medicines
RES: resveratrol
ELISA: enzyme-linked immunosorbent assay kit
ALT: aminotransferase
AST: aspartate transaminase
QC: quality control
DILI: drug-induced liver injury
MPT: mitochondrial permeability transition

## References

[1] Bernal W, Auzinger G, Dhawan A, Wendon J (2010). Acute liver failure. Lancet.

[2] Cohen SD, Khairallah EA (1997). Selective protein arylation and acetaminophen-induced hepatotoxicity. Drug metabolism reviews.

[3] Mbimba T, Awale P, Bhatia D, Geldenhuys WJ, Darvesh AS, Carroll RT (2012). Alteration of hepatic proinflammatory cytokines is involved in the resveratrol-mediated chemoprevention of chemically-induced hepatocarcinogenesis. Current pharmaceutical biotechnology.

[4] Saito C, Lemasters JJ, Jaeschke H (2010). c-Jun N-terminal kinase modulates oxidant stress and peroxynitrite formation independent of inducible nitric oxide synthase in acetaminophen hepatotoxicity. Toxicology and applied pharmacology.

[5] Zhao SY, Liu ZL, Shu YS, Wang ML, He D, Song ZQ (2017). Chemotaxonomic Classification Applied to the Identification of Two Closely-Related Citrus TCMs Using UPLC-Q-TOF-MS-Based Metabolomics.

[6] Da Pozzo E, De Leo M, Faraone I, Milella L, Cavallini C, Piragine E (2018). Antioxidant and Antisenescence Effects of Bergamot Juice. Oxidative medicine and cellular longevity.

[7] Zeng HL, Liu ZL, Song ZQ, Wang C, Dong YZ, Ning ZC (2016). [Study on HPLC fingerprint and chemical constituent difference of different species of Aurantii Fructus Immaturus]. Zhongguo Zhong Yao Za Zhi.

[8] Liu Y, Liu Z, Wang C, Zha Q, Lu C, Song Z (2014). Study on essential oils from four species of Zhishi with gas chromatography-mass spectrometry. Chem Cent J.

[9] Shu Y, Liu Z, Zhao S, Song Z, He D, Wang M (2017). Integrated and global pseudotargeted metabolomics strategy applied to screening for quality control markers of Citrus TCMs. Analytical and bioanalytical chemistry.

[10] Suntar I, Khan H, Patel S, Celano R, Rastrelli L (2018). An Overview on Citrus aurantium L.: Its Functions as Food Ingredient and Therapeutic Agent. Oxidative medicine and cellular longevity.

[11] Zhao S, Liu Z, Wang M, He D, Liu L, Shu Y (2018). Anti-inflammatory effects of Zhishi and Zhiqiao revealed by network pharmacology integrated with molecular mechanism and metabolomics studies. Phytomedicine: international journal of phytotherapy and phytopharmacology.

[12] Pujos-Guillot E, Hubert J, Martin JF, Lyan B, Quintana M, Claude S (2013). Mass spectrometry-based metabolomics for the discovery of biomarkers of fruit and vegetable intake: citrus fruit as a case study. Journal of proteome research.

[13] Gum SI, Cho MK (2013). Recent updates on acetaminophen hepatotoxicity: the role of nrf2 in hepatoprotection. Toxicological research.

[14] Sun J, Wen Y, Zhou Y, Jiang Y, Chen Y, Zhang H (2018). p53 attenuates acetaminophen-induced hepatotoxicity by regulating drug-metabolizing enzymes and transporter expression. Cell death & disease.

[15] Zhao X, Cong X, Zheng L, Xu L, Yin L, Peng J (2012). Dioscin, a natural steroid saponin, shows remarkable protective effect against acetaminophen-induced liver damage in vitro and in vivo. Toxicology letters.

[16] Fan X, Jiang Y, Wang Y, Tan H, Zeng H, Wang Y (2014). Wuzhi tablet (Schisandra Sphenanthera extract) protects against acetaminophen-induced hepatotoxicity by inhibition of CYP-mediated bioactivation and regulation of NRF2-ARE and p53/p21 pathways. Drug metabolism and disposition: the biological fate of chemicals.

[17] Rauf A, Imran M, Suleria HAR, Ahmad B, Peters DG, Mubarak MS (2017). A comprehensive review of the health perspectives of resveratrol. Food Funct.

[18] Al Humayed S, Al-Ani B, El Karib AO, Shatoor AS, Eid RA, Aziz S (2019). Suppression of acetaminophen-induced hepatocyte ultrastructural alterations in rats using a combination of resveratrol and quercetin.

[19] Faghihzadeh F, Hekmatdoost A, Adibi P (2015). Resveratrol and liver: A systematic review. J Res Med Sci.

[20] Yu S, Zhou X, Xiang H, Wang S, Cui Z, Zhou J (2019). Resveratrol Reduced Liver Damage After Liver Resection in a Rat Model by Upregulating Sirtuin 1 (SIRT1) and Inhibiting the Acetylation of High Mobility Group Box 1 (HMGB1). Med Sci Monit.

[21] Zeng H, Liu Z, Zhao S, Shu Y, Song Z, Wang C (2016). Preparation and quantification of the total phenolic products in Citrus fruit using solid-phase extraction coupled with high-performance liquid chromatography with diode array and UV detection. Journal of separation science.

[22] Mohammadi A, Kazemi S, Hosseini M, Najafzadeh Varzi H, Feyzi F, Morakabati P (2019). The Chrysin effect in prevention of acetaminophen-induced hepatotoxicity in rat.

[23] Tripathi SS, Singh S, Garg G, Kumar R, Verma AK, Singh AK (2019). Metformin ameliorates acetaminophen-induced sub-acute toxicity via antioxidant property.

[24] Al Humayed S, Al-Hashem F, Haidara MA, El Karib AO, Kamar SS, Amin SN (2019). Resveratrol Pretreatment Ameliorates p53-Bax Axis and Augments the Survival Biomarker B-Cell Lymphoma 2 Modulated by Paracetamol Overdose in a Rat Model of Acute Liver Injury.

[25] Aragon-Herrera A, Feijoo-Bandin S, Otero Santiago M, Barral L, Campos-Toimil M, Gil-Longo J (2019). Empagliflozin Reduces The Levels Of CD36 And Cardiotoxic Lipids While Improving Autophagy In The Hearts Of Zucker Diabetic Fatty Rats.

[26] Kumar P, Agarwal A, Singh AK, Gautam AK, Chakraborti S, Kumar U (2019). Antineoplastic properties of zafirlukast against hepatocellular carcinoma via activation of mitochondrial mediated apoptosis. Regul Toxicol Pharmacol.

[27] Cao P, Sun J, Sullivan MA, Huang X, Wang H, Zhang Y (2018). Angelica sinensis polysaccharide protects against acetaminophen-induced acute liver injury and cell death by suppressing oxidative stress and hepatic apoptosis in vivo and in vitro. International journal of biological macromolecules.

[28] Tian Y, Wu B, Li X, Jin X, Zhang F, Jiang C (2019). The Resveratrol Alleviates the Hepatic Toxicity of CuSO4 in the Rat. Biological trace element research.

[29] Schrimpe-Rutledge AC, Codreanu SG, Sherrod SD, McLean JA (2016). Untargeted Metabolomics Strategies-Challenges and Emerging Directions. J Am Soc Mass Spectrom.

[30] Li B, He X, Jia W, Li H (2017). Novel Applications of Metabolomics in Personalized Medicine: A Mini-Review.

[31] Guo Y, Xu A, Wang Y (2016). SIRT1 in Endothelial Cells as a Novel Target for the Prevention of Early Vascular Aging. Journal of cardiovascular pharmacology.

[32] Kim GJ, Jo HJ, Lee KJ, Choi JW, An JH (2018). Oleanolic acid induces p53-dependent apoptosis via the ERK/JNK/AKT pathway in cancer cell lines in prostatic cancer xenografts in mice. Oncotarget.

[33] Wu F, Zheng Y, Gao J, Chen S, Wang Z (2014). Induction of oxidative stress and the transcription of genes related to apoptosis in rare minnow (Gobiocypris rarus) larvae with Aroclor 1254 exposure. Ecotoxicology and environmental safety.

[34] Leise MD, Poterucha JJ, Talwalkar JA (2014). Drug-induced liver injury. Mayo Clinic proceedings.

[35] Suciu M, Gruia AT, Nica DV, Azghadi SM, Mic AA, Mic FA (2015). Acetaminophen-induced liver injury: Implications for temporal homeostasis of lipid metabolism and eicosanoid signaling pathway. Chemico-biological interactions.

[36] Huo Y, Yin S, Yan M, Win S, Aung Than T, Aghajan M (2017). Protective role of p53 in acetaminophen hepatotoxicity. Free radical biology & medicine.

[37] Bi H, Li F, Krausz KW, Qu A, Johnson CH, Gonzalez FJ (2013). Targeted Metabolomics of Serum Acylcarnitines Evaluates Hepatoprotective Effect of Wuzhi Tablet (Schisandra sphenanthera Extract) against Acute Acetaminophen Toxicity. Evid Based Complement Alternat Med.

[38] Jiang Y, Fan X, Wang Y, Tan H, Chen P, Zeng H (2015). Hepato-protective effects of six schisandra lignans on acetaminophen-induced liver injury are partially associated with the inhibition of CYP-mediated bioactivation. Chemico-biological interactions.

[39] Gill P, Bhattacharyya S, McCullough S, Letzig L, Mishra PJ, Luo C (2017). MicroRNA regulation of CYP 1A2, CYP3A4 and CYP2E1 expression in acetaminophen toxicity. Scientific reports.

[40] Jaeschke H, McGill MR, Williams CD, Ramachandran A (2011). Current issues with acetaminophen hepatotoxicity-a clinically relevant model to test the efficacy of natural products. Life sciences.

[41] Ming YN, Zhang JY, Wang XL, Li CM, Ma SC, Wang ZY (2017). Liquid chromatography mass spectrometry-based profiling of phosphatidylcholine and phosphatidylethanolamine in the plasma and liver of acetaminophen-induced liver injured mice. Lipids in health and disease.

[42] Moustafa T, Fickert P, Magnes C, Guelly C, Thueringer A, Frank S (2012). Alterations in lipid metabolism mediate inflammation, fibrosis, and proliferation in a mouse model of chronic cholestatic liver injury. Gastroenterology.

[43] Chen C, Qincao L, Xu J, Du S, Huang E, Liu C (2016). Role of PUMA in methamphetamine-induced neuronal apoptosis. Toxicology letters.

[44] Chen D, Ni HM, Wang L, Ma X, Yu J, Ding WX (2019). p53 Up-regulated Modulator of Apoptosis Induction Mediates Acetaminophen-Induced Necrosis and Liver Injury in Mice. Hepatology.

[45] Jakhar R, Paul S, Bhardwaj M, Kang SC (2016). Astemizole-Histamine induces Beclin-1-independent autophagy by targeting p53-dependent crosstalk between autophagy and apoptosis. Cancer letters.

