# Supplementary material for: Hepatoprotective Effect of Citrus aurantium L. Against APAP-induced Liver Injury by Regulating Liver Lipid Metabolism and Apoptosis
Source: Int J Biol Sci. 2020 Jan 14;16(5):752–65. doi: 10.7150/ijbs.40612 (PMC7019131; doi:10.7150/ijbs.40612)
Supplement: Supplementary file 1 — Supplementary figure and tables. [file ijbsv16p0752s1.pdf]

Supplementary data

Table S1 The origins of the Zhishi samples.

Name	Species	Local name	Location and collection time	Growing enviroment
Zhishi	*Citrus aurantium* L.	Sour orange	Jiangxi, 2016.05	Hillsides (N 29° E 106°; Alt.229 m)

Table S2. The content of 32 phenolic compounds.

Name	Content (ng/ml)
Acacetin	770.4
Apigenin	1490.1
Apigenin-7-O-Glucoside	19076.58
Auraptene	1595.34
Bergapten	1653.6
5-Demethylnobiletin	113856.18
Diosmetin	5558.46
Diosmetin-7-O-Glucoside	34483.38
Diosmin	629279.22
Eriocitrin	2166024.54
Eriodictyol	4218.9
Hesperetin	340966.86
Hesperidin	20603969.1
Imperatorin	874.38
Isopimpinellin	3898.14
Isosakuranetin	792.48
Kaempferitrin	9062.52
Limonin	97171.56
Luteolin	2591288.1
Naringenin	261388.26
Naringin	61045773.9
Narirutin	17752304.46
Neohesperidin	97244453.52
Nobiletin	2291214.3
Nomilin	50669.22
Poncirin	520566.96
Rhoifolin	2631472.98
Rutin	852041.22
Scoparone	4124.04

Sinensetin	256528.62
Tangeretin	3802648.92
Xanthotoxol	15009

Table S3.Different chemical constituents from the Zhishi extract in positive ion mode.

No.	RT (time)	Formula	$[M+H]^+$ m/z		Mw(Da)	Identification
			Detected	Mass Erorr		
Flavones and flavones glycoside						
1	3.37	$C_{15}H_{10}O_8$	319.0446	−0.6	318.0448	Myricetin
2	3.83	$C_{21}H_{20}O_{10}$	433.0988	1.2	432.0983	Vitexin
3	3.86	$C_{22}H_{22}O_{12}$	479.1185	0.2	478.1184	Isorhamnetin-3-O-glucoside
4	3.93	$C_{22}H_{22}O_{11}$	463.1156	-1.1	462.1161	Diosmetin-7-O-glucoside
5	4.09	$C_{27}H_{30}O_{14}$	579.1630	-1.0	578.1636	Isorhoifolin
6	4.13	$C_{21}H_{20}O_{11}$	449.1003	-0.4	448.1005	Orientin
7	4.18	$C_{26}H_{28}O_{14}$	565.1475	-0.7	563.1479	Apiin
8	4.2	$C_{27}H_{30}O_{14}$	579.1626	-1.7	578.1636	Rhoifolin
9	4.28	$C_{28}H_{32}O_{15}$	609.1745	0.7	608.1741	Diosmin
10	4.37	$C_{28}H_{32}O_{15}$	609.1745	0.7	608.1741	Neodiosmin
11	4.47	$C_{21}H_{20}O_{11}$	449.1009	0.9	448.1005	Cynaroside
12	4.47	$C_{28}H_{32}O_{14}$	593.1786	-1.0	592.1792	Linarin
13	5.21	$C_{16}H_{12}O_6$	301.0629	-1.7	300.0634	Chrysoeriol
14	5.21	$C_{30}H_{18}O_{10}$	539.0896	-0.7	538.0900	Amentoflavone
15	5.51	$C_{21}H_{20}O_{10}$	433.1040	-3.9	432.1057	Apigetrin
16	5.51	$C_{27}H_{30}O_{15}$	595.1589	0.8	594.1584	Saponarin
17	5.58	$C_{15}H_{10}O_6$	287.0476	-0.3	286.0477	Luteolin
18	6.3	$C_{15}H_{10}O_5$	271.0526	-0.7	270.0528	Apigenin
19	6.58	$C_{16}H_{12}O_6$	301.0637	1.0	300.0634	Diosmetin
20	6.95	$C_{16}H_{12}O_5$	285.0678	-2.5	284.0685	Acacetin
21	7.75	$C_{20}H_{20}O_7$	373.1212	0.8	372.1209	Sinensetin
22	8.35	$C_{21}H_{22}O_8$	403.1320	1.2	402.1315	Nobiletin
23	8.38	$C_{19}H_{18}O_6$	343.1108	1.5	342.1103	Tetramethyl-isoscutellarein
24	8.88	$C_{20}H_{20}O_7$	373.1201	-2.1	372.1209	Tangeretin
25	9.31	$C_{20}H_{20}O_8$	389.1162	1.0	388.1158	5-demethylnobiletin
Flavanones and flavanone glycoside						
26	3.71	$C_{27}H_{32}O_{15}$	597.1745	0.7	596.1741	Eriocitrin
27	3.73	$C_{21}H_{22}O_{11}$	451.1156	-1.3	450.1162	Pyracanthoside
28	3.74	$C_{28}H_{34}O_{14}$	595.1940	-1.5	594.1949	Neoponcirin
29	3.82	$C_{27}H_{32}O_{15}$	597.1746	0.8	596.1741	Neoeriocitrin
30	3.84	$C_{28}H_{34}O_{14}$	595.1935	-2.3	594.1949	Poncirin
31	4.1	$C_{27}H_{32}O_{14}$	581.1786	-1.0	580.1792	Narirutin
32	4.26	$C_{27}H_{32}O_{14}$	581.1802	1.7	580.1792	Naringin
33	4.34	$C_{28}H_{34}O_{15}$	611.1882	-2.5	610.1897	Hesperidin

34	4.49	$C_{28}H_{34}O_{15}$	611.1889	-1.3	610.1897	Neohesperidin
35	4.69	$C_{16}H_{14}O_6$	303.0792	0.7	302.0790	Homoeriodictyol
36	4.82	$C_{15}H_{12}O_6$	289.0628	-2.1	288.0634	Eriodictyol
37	5.51	$C_{16}H_{14}O_5$	287.0835	-2.1	286.0841	Sakuranetin
38	6.3	$C_{15}H_{12}O_5$	273.0673	-4.4	272.0685	Naringenin
39	6.62	$C_{16}H_{14}O_6$	303.0795	1.7	302.0790	Hesperetin
Flavonols						
40	3.39	$C_{27}H_{30}O_{14}$	579.1630	-1.0	578.1636	Kaempferitrin
41	3.67	$C_{27}H_{30}O_{16}$	611.1543	1.5	610.1534	Rutin
42	5.60	$C_{15}H_{10}O_7$	303.0425	-0.7	302.0427	Quercetin
Cyanidin						
43	2.98	$C_{21}H_{21}ClO_{11}$	485.0782	2.1	484.0772	Cyanidin-3-O-glucoside
Coumarins						
44	3.46	$C_{11}H_{10}O_4$	207.0577	-1.0	206.0579	Limettin
45	4.35	$C_9H_6O_3$	163.0312	-3.1	162.0317	Umbelliferone
46	5.34	$C_{11}H_{10}O_4$	207.0570	-4.4	206.0579	Scoparone
47	6.15	$C_{20}H_{24}O_4$	329.1681	2.1	328.1674	5-Geranyloxy-7-Methoxycoumarin
48	7.85	$C_{19}H_{22}O_4$	315.1507	-3.5	314.1518	Epoxyaurapten
49	9.60	$C_{15}H_{16}O_3$	245.1102	1.2	244.1099	Osthol
50	10.67	$C_{19}H_{22}O_3$	299.1571	0.7	298.1569	Aurapten
51	11.00	$C_{19}H_{22}O_3$	299.1565	-1.3	298.1569	Auraptene
Furanocoumarins						
52	4.56	$C_{17}H_{18}O_7$	335.1053	0.3	334.1052	Byakangelicin
53	5.31	$C_{16}H_{16}O_6$	305.09451	-2.0	304.0947	Heraclenol
54	5.51	$C_{16}H_{14}O_5$	287.0845	1.4	286.0841	Heraclenin
55	5.72	$C_{16}H_{16}O_6$	305.0940	-2.3	304.0947	Oxypeucedanin hydrate
56	6.75	$C_{11}H_6O_3$	187.0318	0.5	186.0317	Psoralen
57	7.06	$C_{12}H_8O_4$	217.0420	-0.9	216.0422	Xanthotoxin
58	7.06	C21H22O5	355.1472	1.4	354.1467	Epoxybergamottin
59	7.43	$C_{12}H_8O_4$	217.0421	-0.5	216.0422	Bergapten
60	7.48	$C_{13}H_{10}O_5$	247.0531	1.2	246.0528	Isopimpinellin
61	7.89	C11H6O4	203.0274	4.0	202.0266	Xanthotoxol
62	7.89	$C_{17}H_{16}O_5$	301.1007	3.0	300.099998	Cnidilin
63	8.22	$C_{21}H_{24}O_6$	373.1581	2.1	372.1573	6’-7’-dihydroxybergamotin
64	8.24	$C_{16}H_{14}O_5$	287.0832	-3.1	286.0841	Oxypeucedanin
65	8.45	$C_{17}H_{16}O_6$	317.0956	2.8	316.0947	Byakangelicol
66	9.27	$C_{16}H_{14}O_4$	271.0895	1.1	270.0892	Imperatorin
67	9.54	$C_{17}H_{16}O_5$	301.0997	-0.3	300.0998	Phellopterin

68	9.76	$C_{16}H_{14}O_4$	271.0892	0.0	270.0892	Isoimperatorin
69	10.37	$C_{21}H_{22}O_5$	355.1465	-0.6	354.1467	Cnidicin
70	10.87	$C_{21}H_{22}O_4$	339.1513	-1.5	338.1515	8-geranyloxypsoralen
71	11.26	$C_{11}H_6O_4$	203.0267	0.5	202.0266	Bergaptol
72	11.56	$C_{21}H_{22}O_4$	339.1522	1.2	338.1518	Berganmottin
Limonin						
73	7.82	$C_{26}H_{30}O_8$	471.2033	0.4	470.2031	Limonin
74	8.38	$C_{28}H_{34}O_9$	515.2447	-0.2	514.2448	Nomilin
Abscisic acid						
75	4.14	$C_{15}H_{20}O_4$	265.1380	2.3	264.1374	Abscisic acid

Figure S1.RRLC-QqQ-MS spectrum of 32 chemical constituents

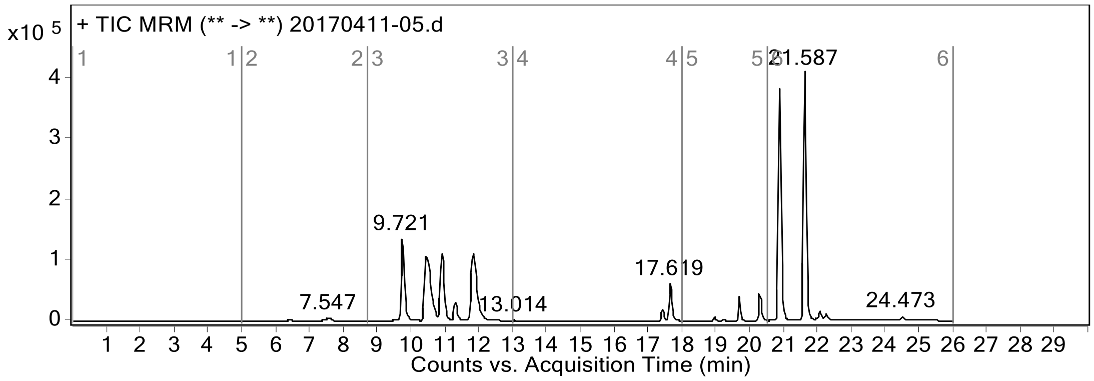

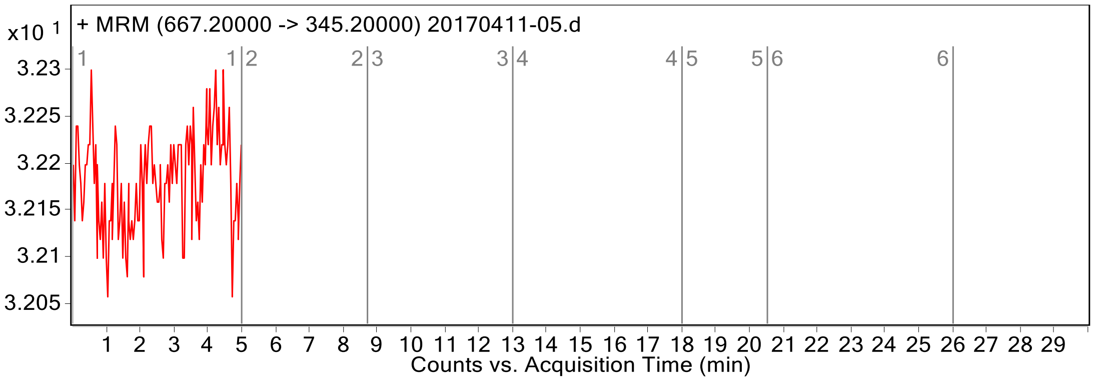

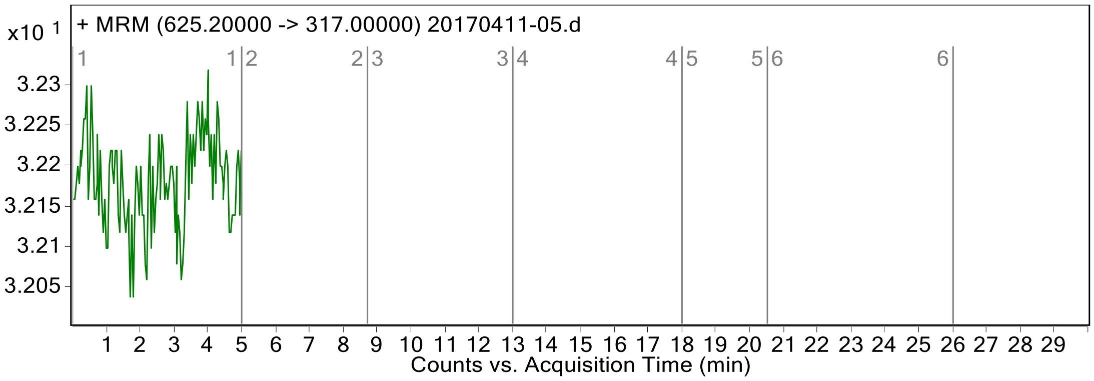

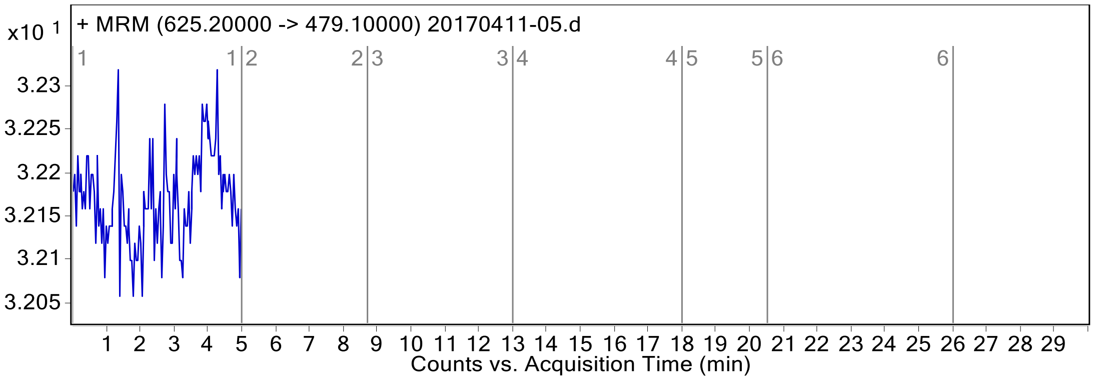
x10 1
+ MRM (625.20000 -> 479.10000) 20170411-05.d
3.23
3.225
3.22
3.215
3.21
3.205
1 2 3 4 5 6 7 8 9 10 11 12 13 14 15 16 17 18 19 20 21 22 23 24 25 26 27 28 29
Counts vs. Acquisition Time (min)

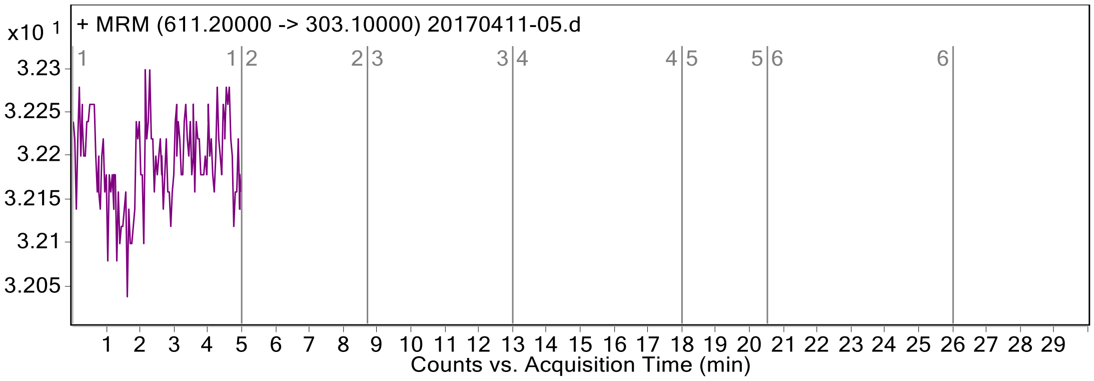
x10 1
+ MRM (611.20000 -> 303.10000) 20170411-05.d
3.23
3.225
3.22
3.215
3.21
3.205
1 2 3 4 5 6 7 8 9 10 11 12 13 14 15 16 17 18 19 20 21 22 23 24 25 26 27 28 29
Counts vs. Acquisition Time (min)

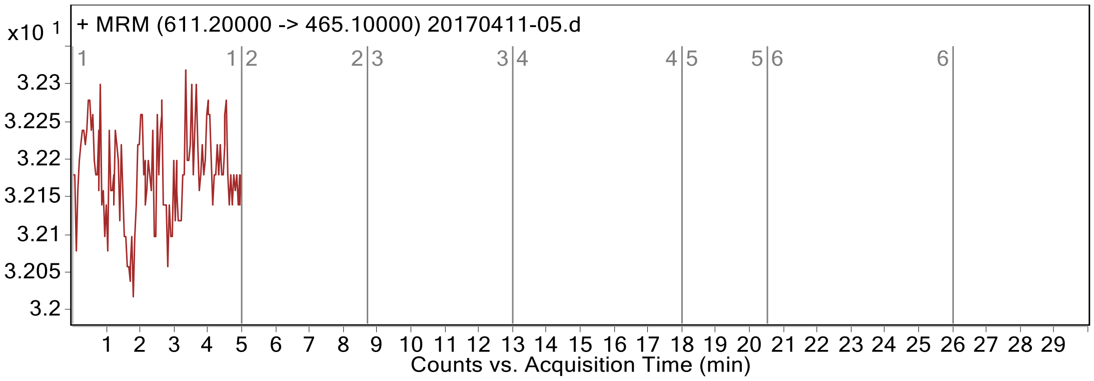
x10 1
+ MRM (611.20000 -> 465.10000) 20170411-05.d
3.23
3.225
3.22
3.215
3.21
3.205
3.2
1 2 3 4 5 6 7 8 9 10 11 12 13 14 15 16 17 18 19 20 21 22 23 24 25 26 27 28 29
Counts vs. Acquisition Time (min)

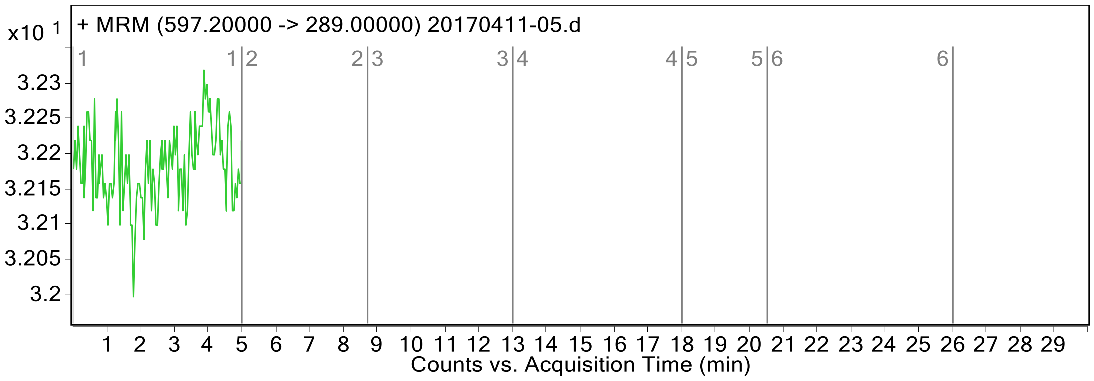
x10 1
+ MRM (597.20000 -> 289.00000) 20170411-05.d
3.23
3.225
3.22
3.215
3.21
3.205
3.2
1 2 3 4 5 6 7 8 9 10 11 12 13 14 15 16 17 18 19 20 21 22 23 24 25 26 27 28 29
Counts vs. Acquisition Time (min)

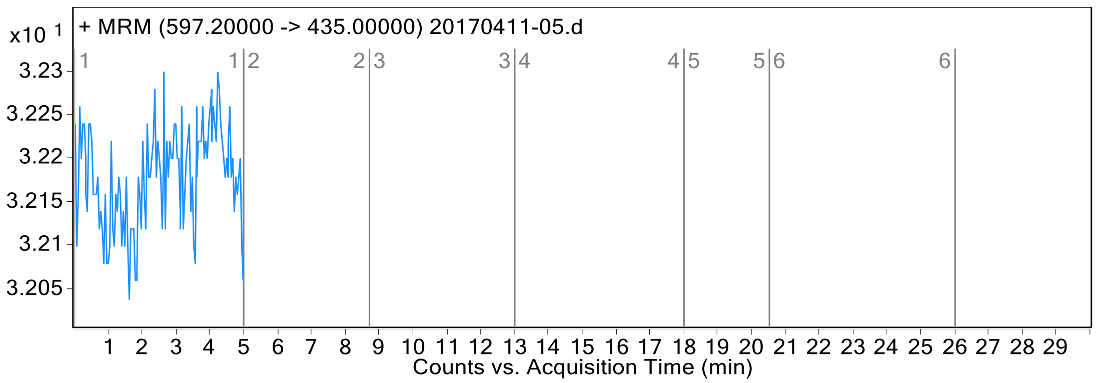
x10 1
+ MRM (597.20000 -> 435.00000) 20170411-05.d
3.23
3.225
3.22
3.215
3.21
3.205
1 2 3 4 5 6 7 8 9 10 11 12 13 14 15 16 17 18 19 20 21 22 23 24 25 26 27 28 29
Counts vs. Acquisition Time (min)

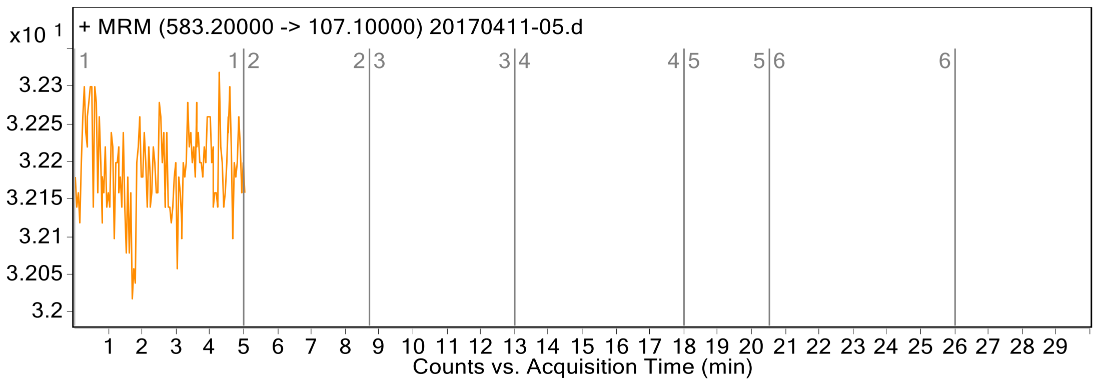
x10 1
+ MRM (583.20000 -> 107.10000) 20170411-05.d
3.23
3.225
3.22
3.215
3.21
3.205
3.2
1 2 3 4 5 6 7 8 9 10 11 12 13 14 15 16 17 18 19 20 21 22 23 24 25 26 27 28 29
Counts vs. Acquisition Time (min)

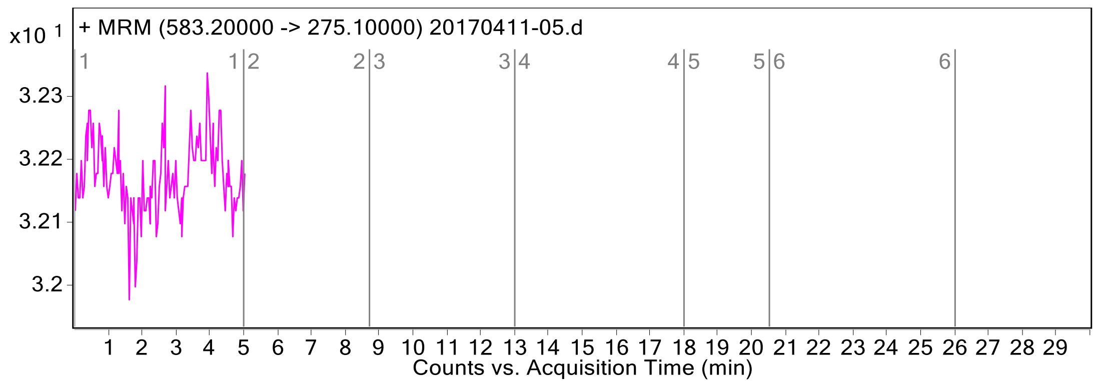
x10 1
+ MRM (583.20000 -> 275.10000) 20170411-05.d
3.23
3.22
3.21
3.2
1 2 3 4 5 6 7 8 9 10 11 12 13 14 15 16 17 18 19 20 21 22 23 24 25 26 27 28 29
Counts vs. Acquisition Time (min)

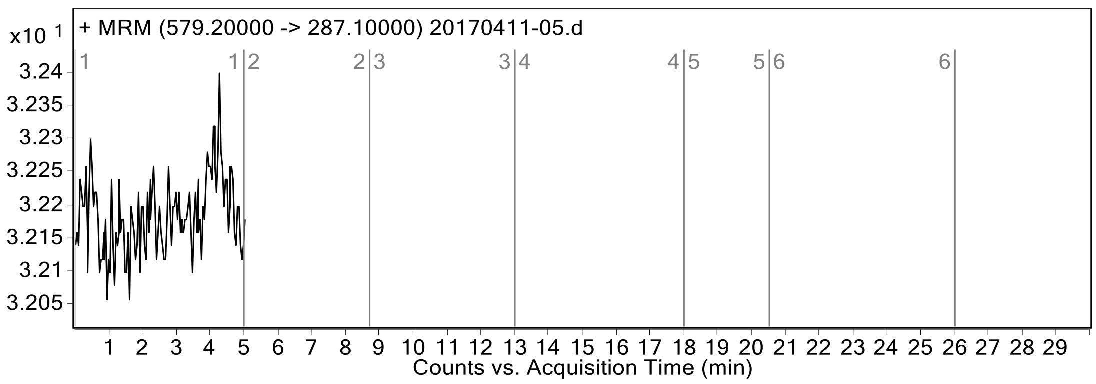
x10 1
+ MRM (579.20000 -> 287.10000) 20170411-05.d
3.24
3.235
3.23
3.225
3.22
3.215
3.21
3.205
1 2 3 4 5 6 7 8 9 10 11 12 13 14 15 16 17 18 19 20 21 22 23 24 25 26 27 28 29
Counts vs. Acquisition Time (min)

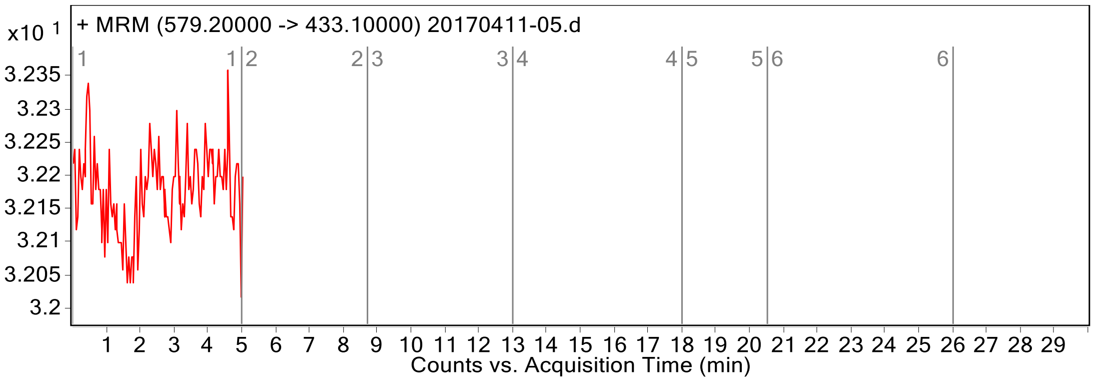
+ MRM (579.20000 -> 433.10000) 20170411-05.d
x10 1
3.235
3.23
3.225
3.22
3.215
3.21
3.205
3.2
1 2 3 4 5 6 7 8 9 10 11 12 13 14 15 16 17 18 19 20 21 22 23 24 25 26 27 28 29
Counts vs. Acquisition Time (min)

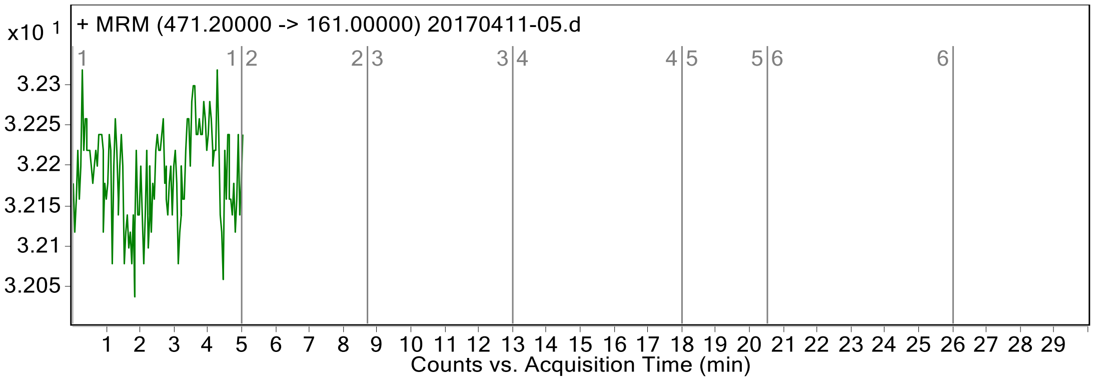
+ MRM (471.20000 -> 161.00000) 20170411-05.d
x10 1
3.23
3.225
3.22
3.215
3.21
3.205
1 2 3 4 5 6 7 8 9 10 11 12 13 14 15 16 17 18 19 20 21 22 23 24 25 26 27 28 29
Counts vs. Acquisition Time (min)

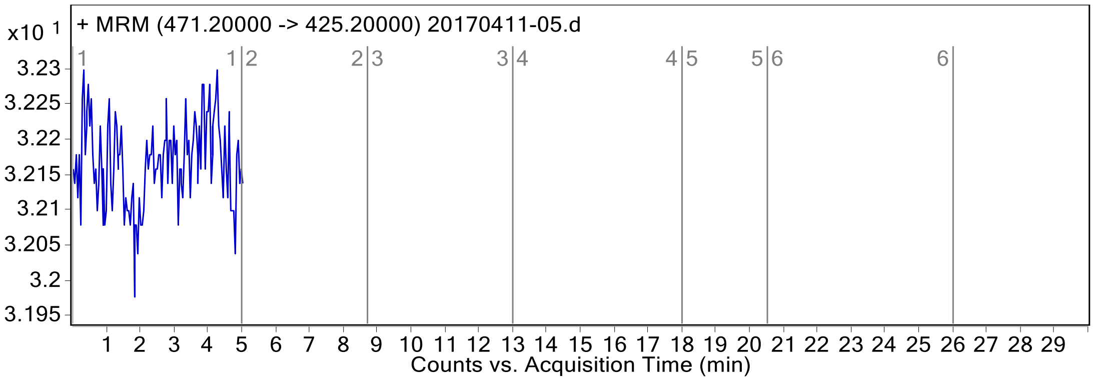
+ MRM (471.20000 -> 425.20000) 20170411-05.d
x10 1
3.23
3.225
3.22
3.215
3.21
3.205
3.2
3.195
1 2 3 4 5 6 7 8 9 10 11 12 13 14 15 16 17 18 19 20 21 22 23 24 25 26 27 28 29
Counts vs. Acquisition Time (min)

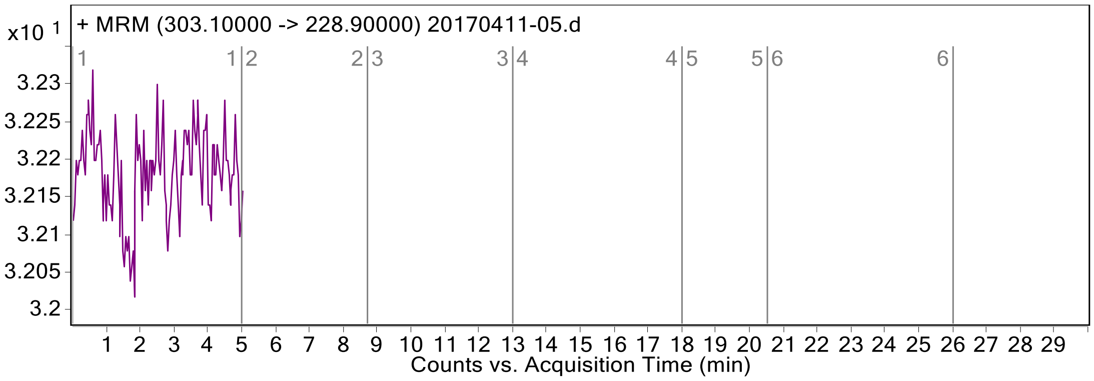
+ MRM (303.10000 -> 228.90000) 20170411-05.d
x10 1
3.23
3.225
3.22
3.215
3.21
3.205
3.2
1 2 3 4 5 6 7 8 9 10 11 12 13 14 15 16 17 18 19 20 21 22 23 24 25 26 27 28 29
Counts vs. Acquisition Time (min)

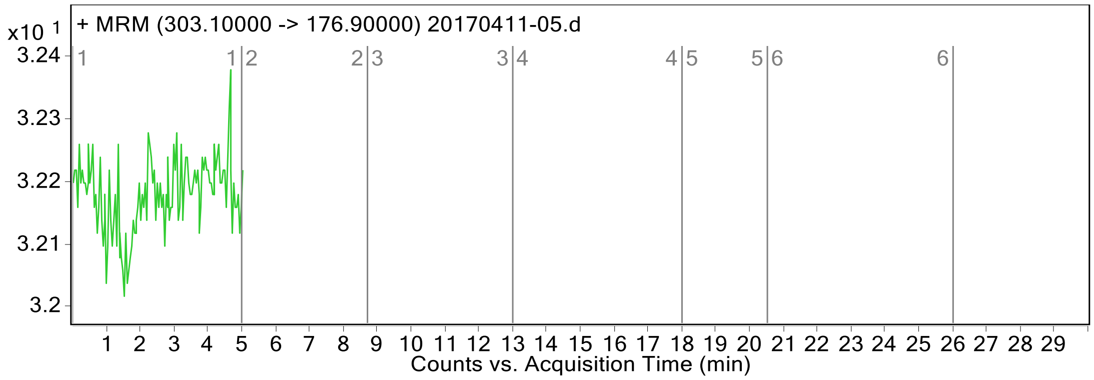
+ MRM (303.10000 -> 176.90000) 20170411-05.d
x10 1
3.24
3.23
3.22
3.21
3.2
Counts vs. Acquisition Time (min)

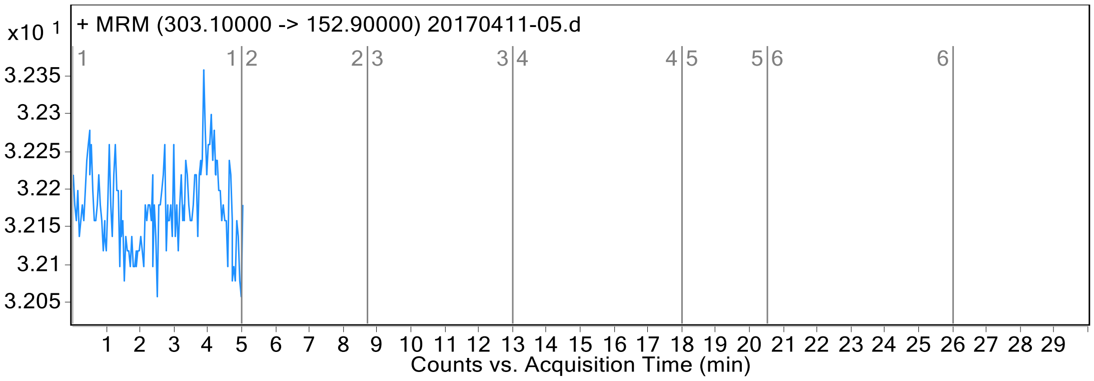
+ MRM (303.10000 -> 152.90000) 20170411-05.d
x10 1
3.235
3.23
3.225
3.22
3.215
3.21
3.205
Counts vs. Acquisition Time (min)

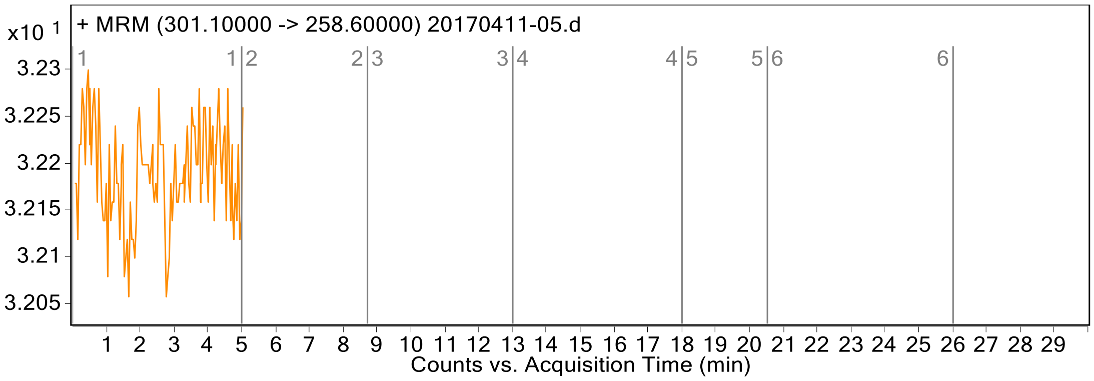
+ MRM (301.10000 -> 258.60000) 20170411-05.d
x10 1
3.23
3.225
3.22
3.215
3.21
3.205
Counts vs. Acquisition Time (min)

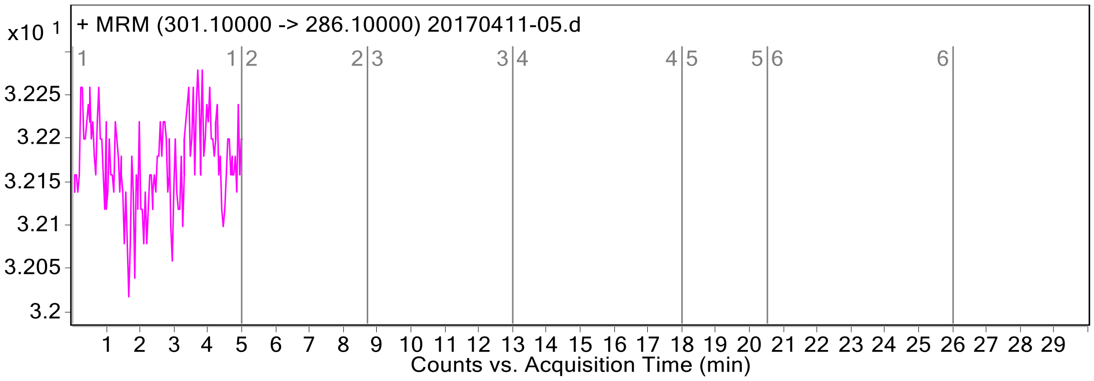
+ MRM (301.10000 -> 286.10000) 20170411-05.d
x10 1
3.225
3.22
3.215
3.21
3.205
3.2
Counts vs. Acquisition Time (min)

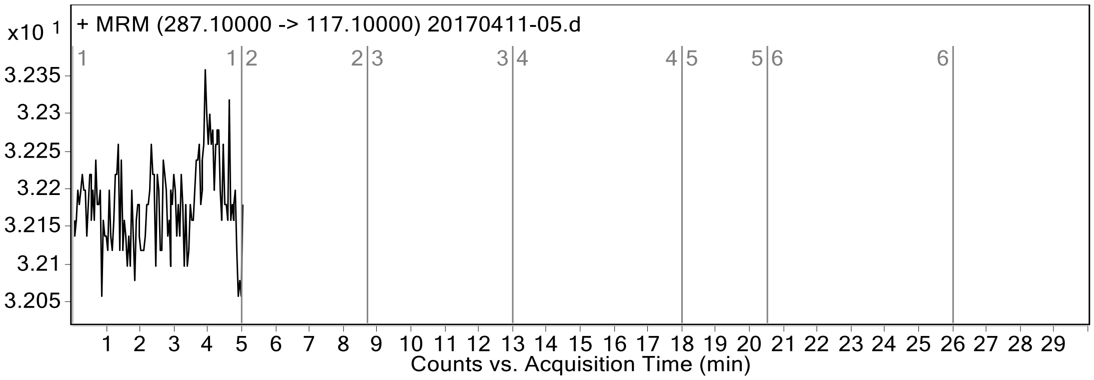
x10 1
+ MRM (287.10000 -> 117.10000) 20170411-05.d
3.235
3.23
3.225
3.22
3.215
3.21
3.205
1 2 3 4 5 6 7 8 9 10 11 12 13 14 15 16 17 18 19 20 21 22 23 24 25 26 27 28 29
Counts vs. Acquisition Time (min)

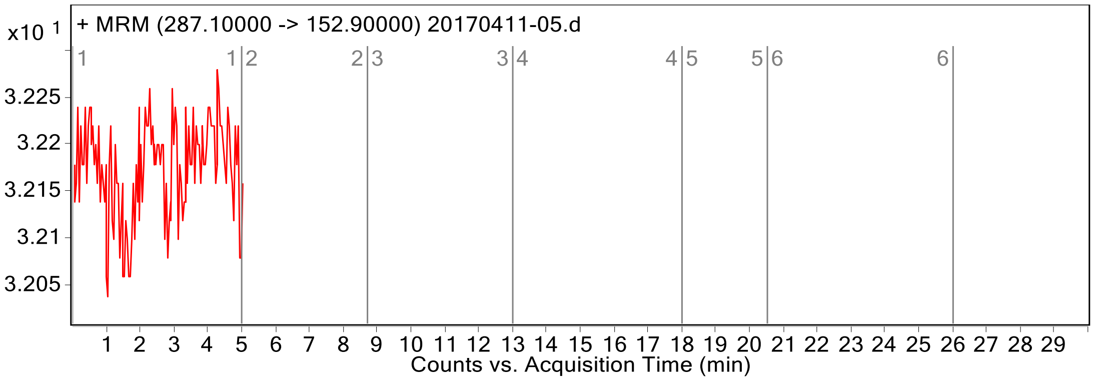
x10 1
+ MRM (287.10000 -> 152.90000) 20170411-05.d
3.225
3.22
3.215
3.21
3.205
1 2 3 4 5 6 7 8 9 10 11 12 13 14 15 16 17 18 19 20 21 22 23 24 25 26 27 28 29
Counts vs. Acquisition Time (min)

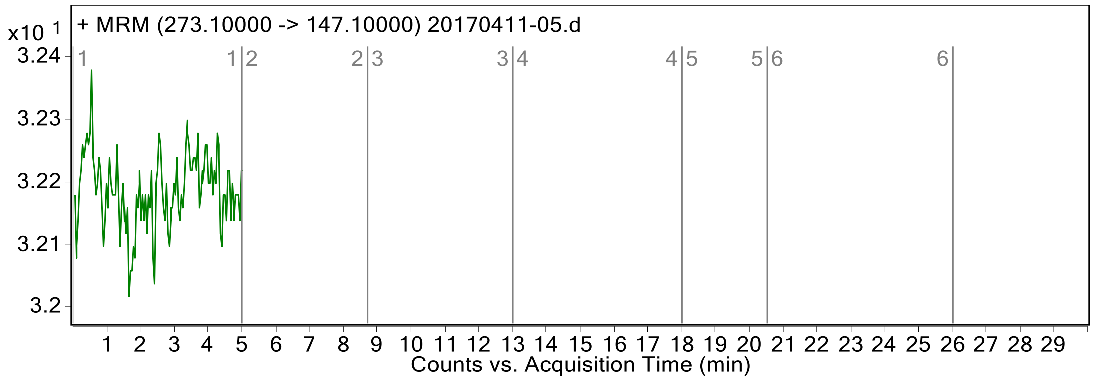
x10 1
+ MRM (273.10000 -> 147.10000) 20170411-05.d
3.24
3.23
3.22
3.21
3.2
1 2 3 4 5 6 7 8 9 10 11 12 13 14 15 16 17 18 19 20 21 22 23 24 25 26 27 28 29
Counts vs. Acquisition Time (min)

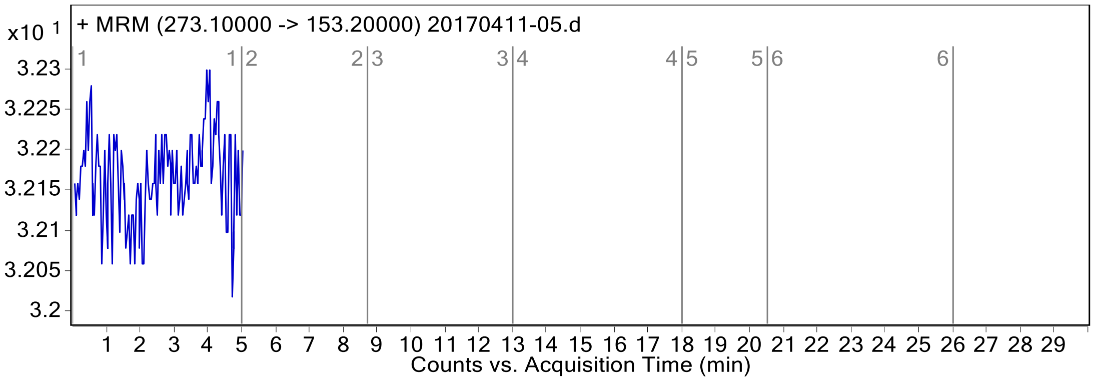
x10 1
+ MRM (273.10000 -> 153.20000) 20170411-05.d
3.23
3.225
3.22
3.215
3.21
3.205
3.2
1 2 3 4 5 6 7 8 9 10 11 12 13 14 15 16 17 18 19 20 21 22 23 24 25 26 27 28 29
Counts vs. Acquisition Time (min)

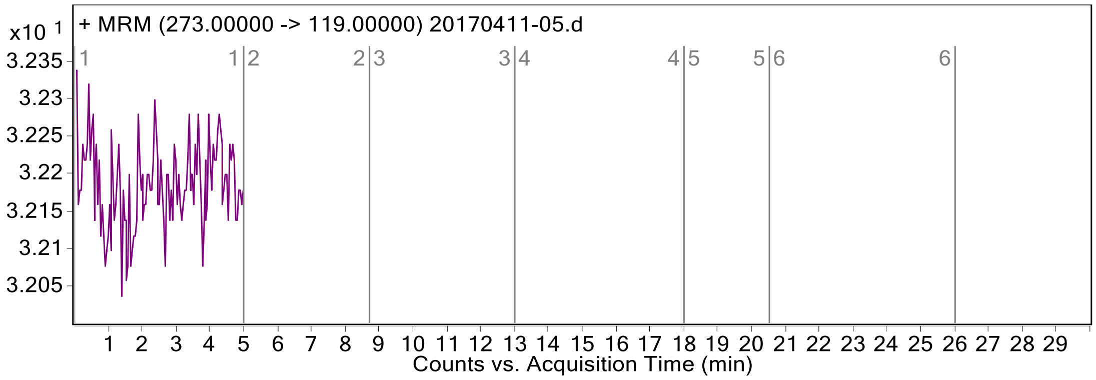

x10 1
+ MRM (273.00000 -> 119.00000) 20170411-05.d
Counts vs. Acquisition Time (min)

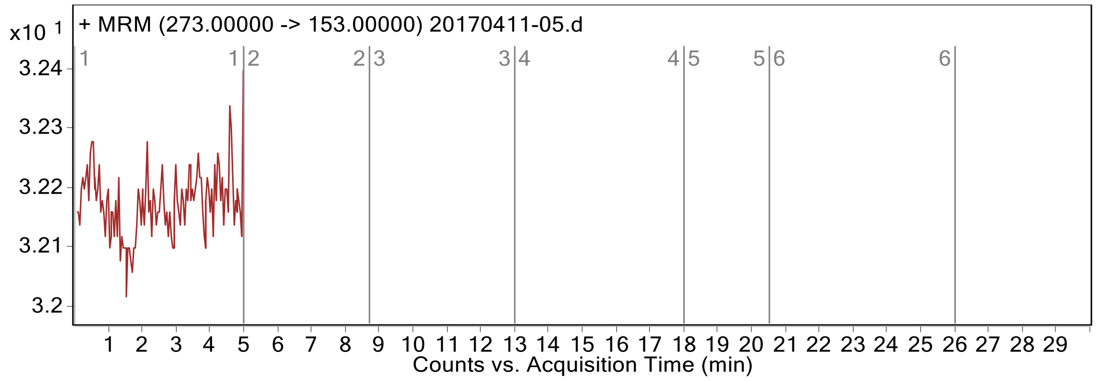

x10 1
+ MRM (273.00000 -> 153.00000) 20170411-05.d
Counts vs. Acquisition Time (min)

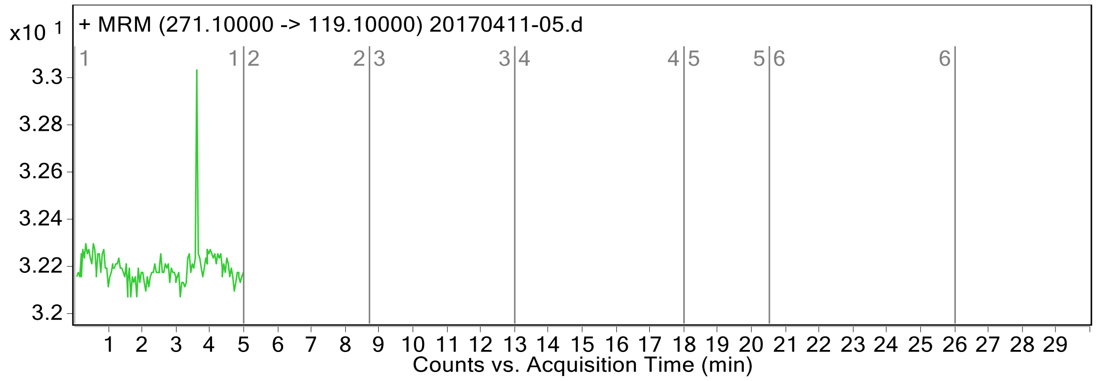

x10 1
+ MRM (271.10000 -> 119.10000) 20170411-05.d
Counts vs. Acquisition Time (min)

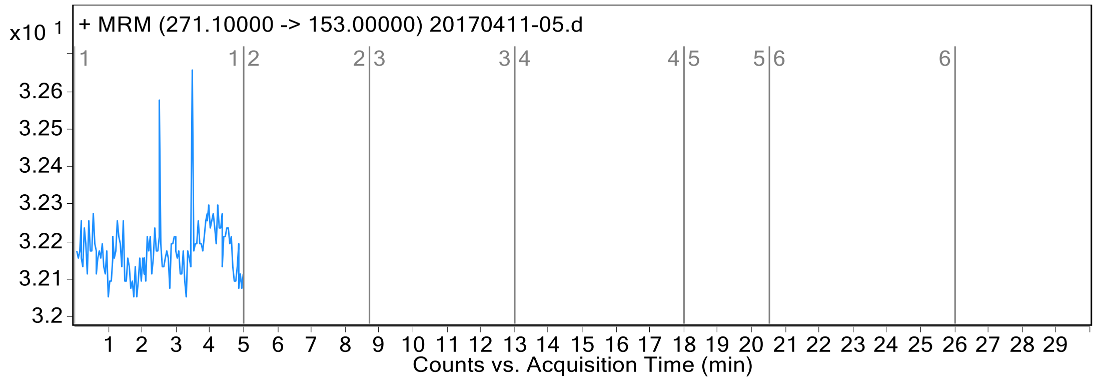

x10 1
+ MRM (271.10000 -> 153.00000) 20170411-05.d
Counts vs. Acquisition Time (min)

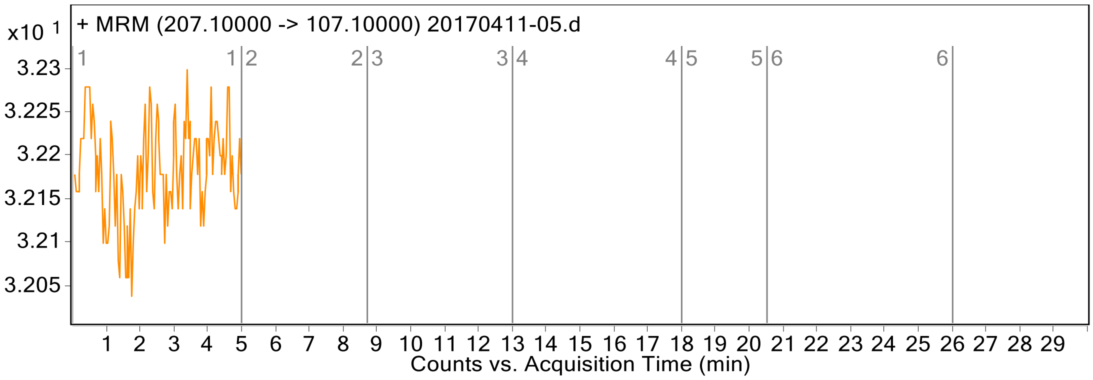
x10 1
+ MRM (207.10000 -> 107.10000) 20170411-05.d
3.23
3.225
3.22
3.215
3.21
3.205
1 2 3 4 5 6 7 8 9 10 11 12 13 14 15 16 17 18 19 20 21 22 23 24 25 26 27 28 29
Counts vs. Acquisition Time (min)

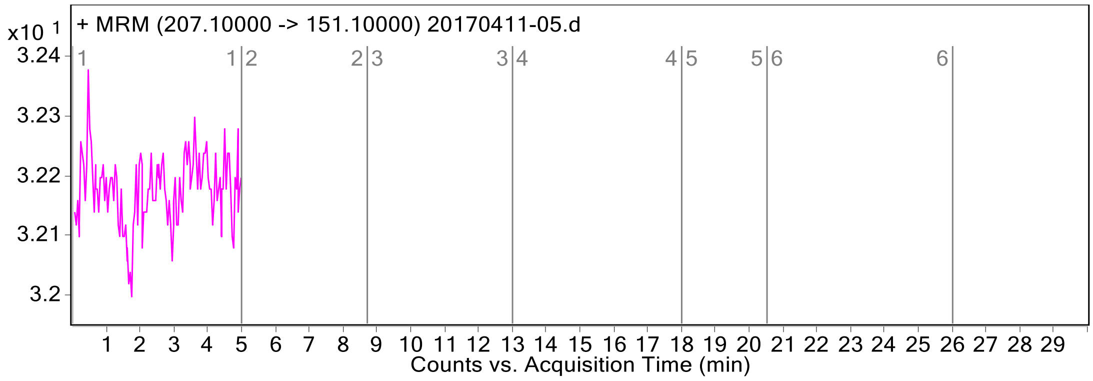
x10 1
+ MRM (207.10000 -> 151.10000) 20170411-05.d
3.24
3.23
3.22
3.21
3.2
1 2 3 4 5 6 7 8 9 10 11 12 13 14 15 16 17 18 19 20 21 22 23 24 25 26 27 28 29
Counts vs. Acquisition Time (min)

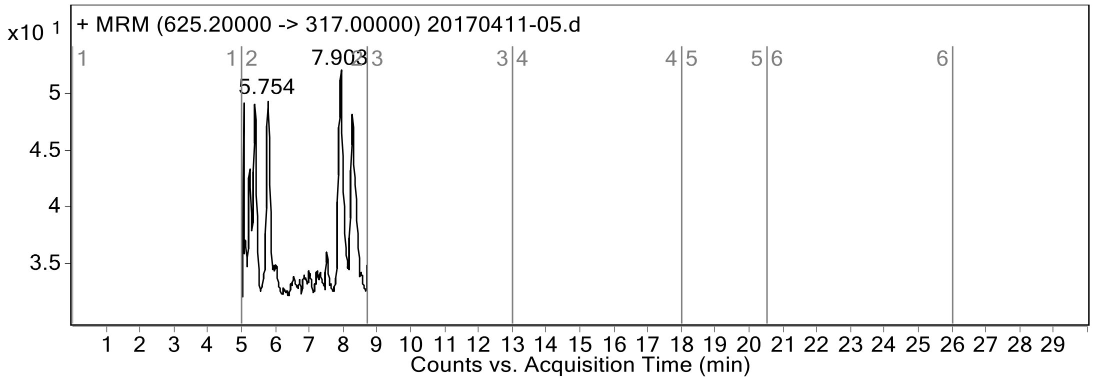
x10 1
+ MRM (625.20000 -> 317.00000) 20170411-05.d
7.903
5.754
5
4.5
4
3.5
1 2 3 4 5 6 7 8 9 10 11 12 13 14 15 16 17 18 19 20 21 22 23 24 25 26 27 28 29
Counts vs. Acquisition Time (min)

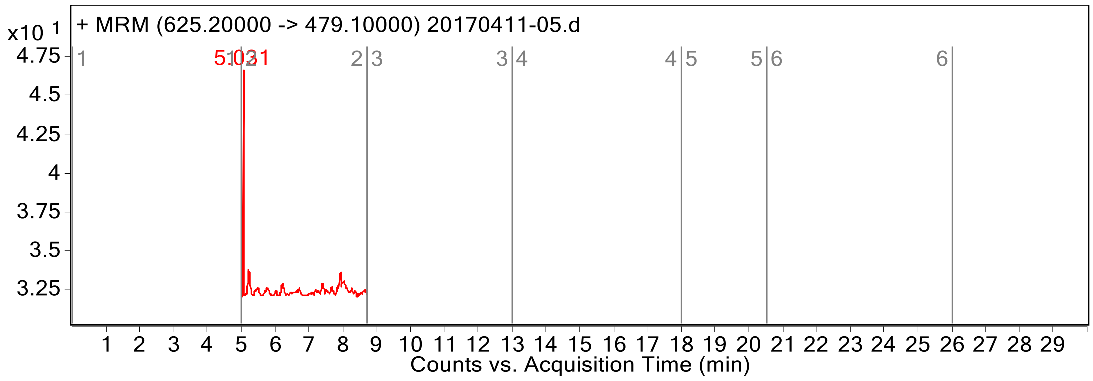
x10 1
+ MRM (625.20000 -> 479.10000) 20170411-05.d
5.031
4.75
4.5
4.25
4
3.75
3.5
3.25
1 2 3 4 5 6 7 8 9 10 11 12 13 14 15 16 17 18 19 20 21 22 23 24 25 26 27 28 29
Counts vs. Acquisition Time (min)

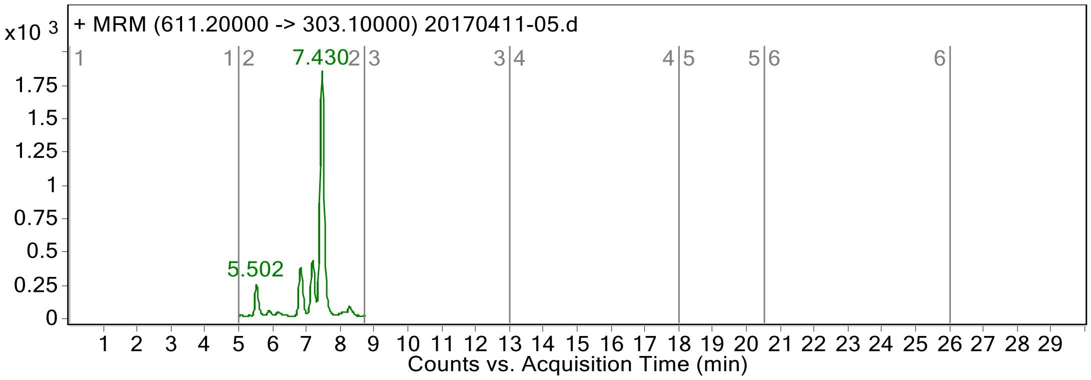
x10 3
+ MRM (611.20000 -> 303.10000) 20170411-05.d
7.430
5.502
Counts vs. Acquisition Time (min)

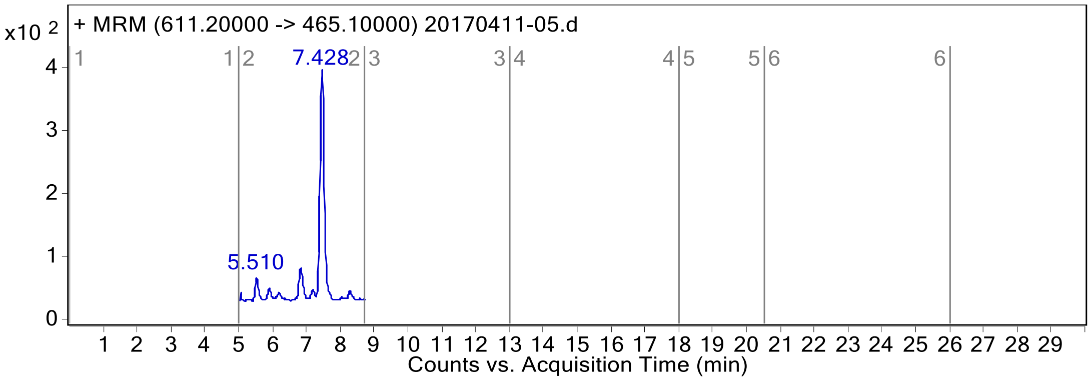
x10 2
+ MRM (611.20000 -> 465.10000) 20170411-05.d
7.428
5.510
Counts vs. Acquisition Time (min)

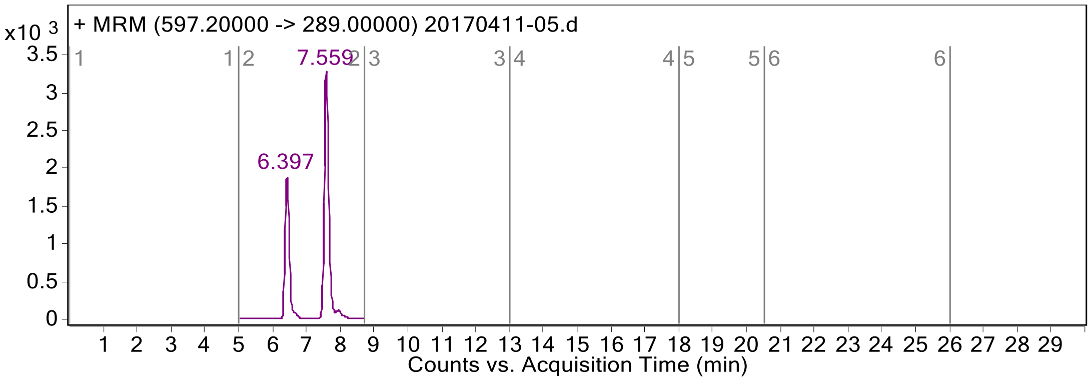
x10 3
+ MRM (597.20000 -> 289.00000) 20170411-05.d
7.559
6.397
Counts vs. Acquisition Time (min)

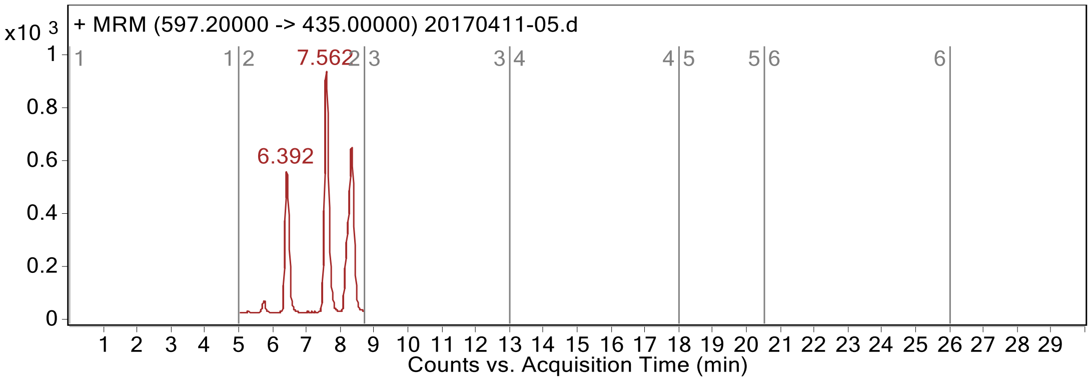
x10 3
+ MRM (597.20000 -> 435.00000) 20170411-05.d
7.562
6.392
Counts vs. Acquisition Time (min)

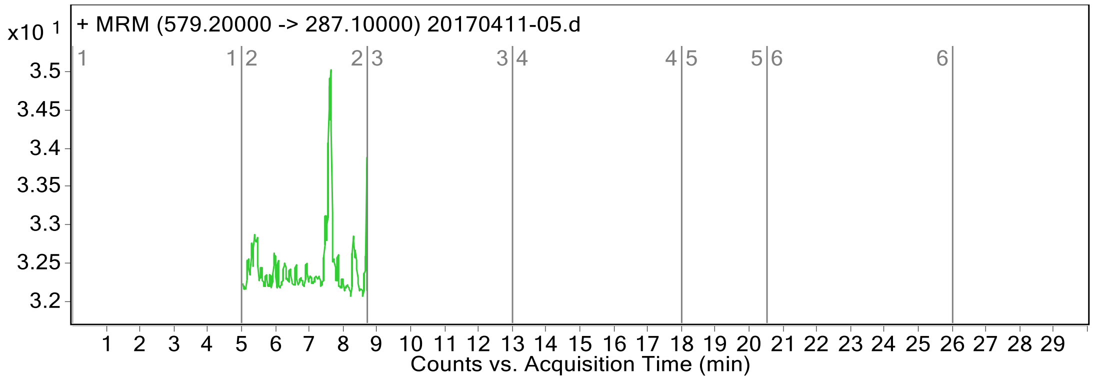
x10 1
+ MRM (579.20000 -> 287.10000) 20170411-05.d
3.5
3.45
3.4
3.35
3.3
3.25
3.2
1 2 3 4 5 6 7 8 9 10 11 12 13 14 15 16 17 18 19 20 21 22 23 24 25 26 27 28 29
Counts vs. Acquisition Time (min)

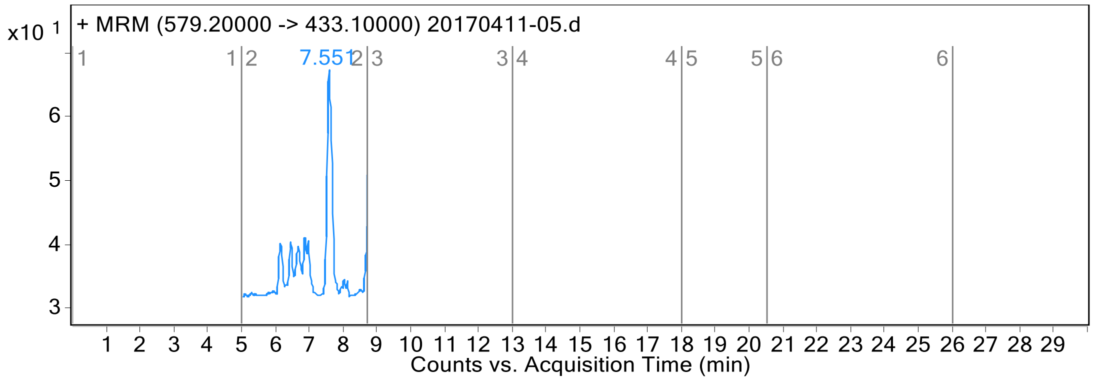
x10 1
+ MRM (579.20000 -> 433.10000) 20170411-05.d
7.551
6
5
4
3
1 2 3 4 5 6 7 8 9 10 11 12 13 14 15 16 17 18 19 20 21 22 23 24 25 26 27 28 29
Counts vs. Acquisition Time (min)

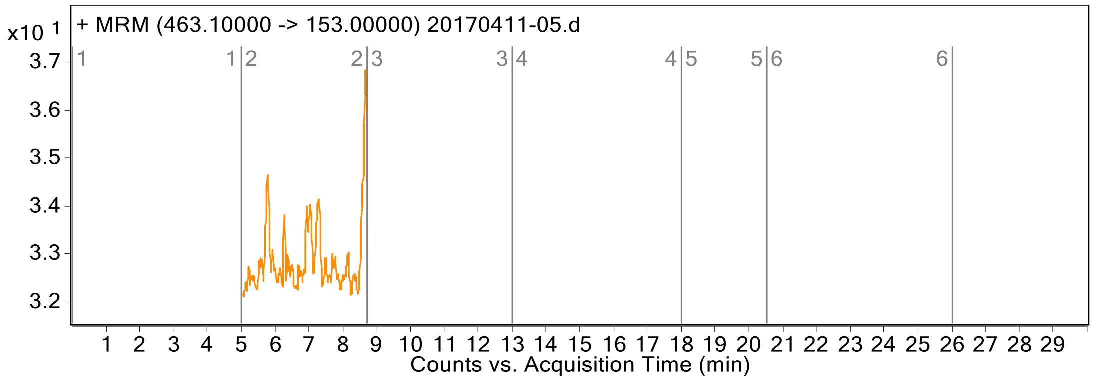
x10 1
+ MRM (463.10000 -> 153.00000) 20170411-05.d
3.7
3.6
3.5
3.4
3.3
3.2
1 2 3 4 5 6 7 8 9 10 11 12 13 14 15 16 17 18 19 20 21 22 23 24 25 26 27 28 29
Counts vs. Acquisition Time (min)

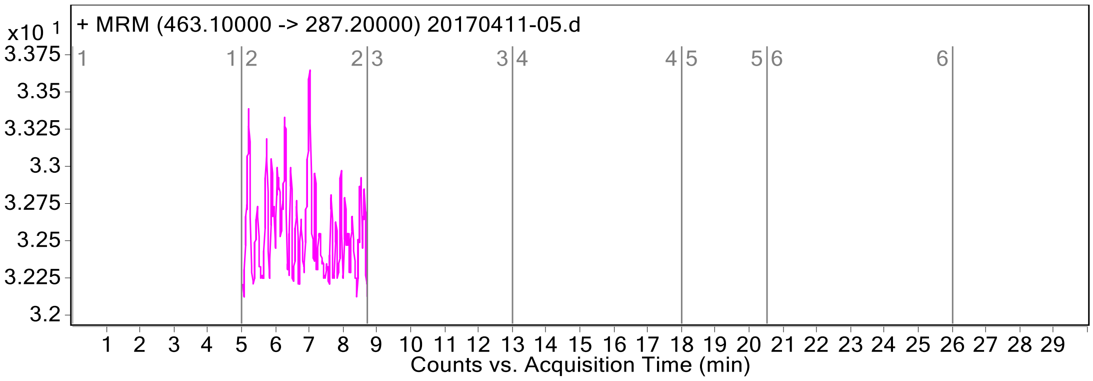
x10 1
+ MRM (463.10000 -> 287.20000) 20170411-05.d
3.375
3.35
3.325
3.3
3.275
3.25
3.225
3.2
1 2 3 4 5 6 7 8 9 10 11 12 13 14 15 16 17 18 19 20 21 22 23 24 25 26 27 28 29
Counts vs. Acquisition Time (min)

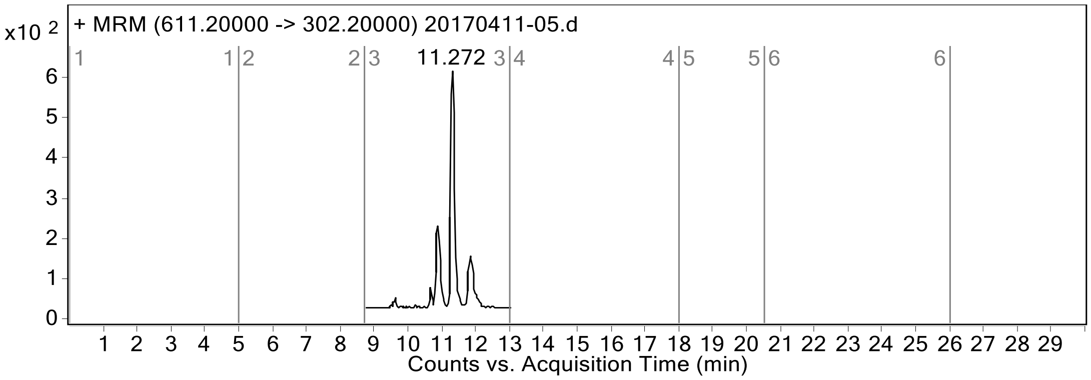
x10 2
+ MRM (611.20000 -> 302.20000) 20170411-05.d
11.272
Counts vs. Acquisition Time (min)

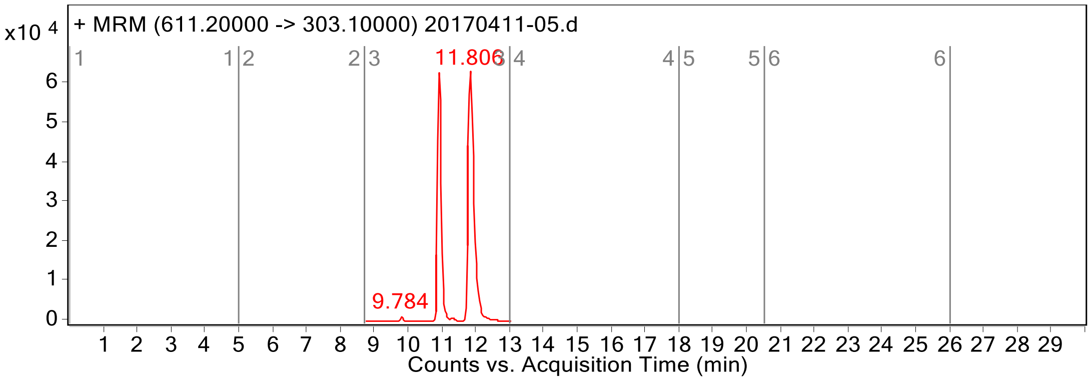
x10 4
+ MRM (611.20000 -> 303.10000) 20170411-05.d
11.806
9.784
Counts vs. Acquisition Time (min)

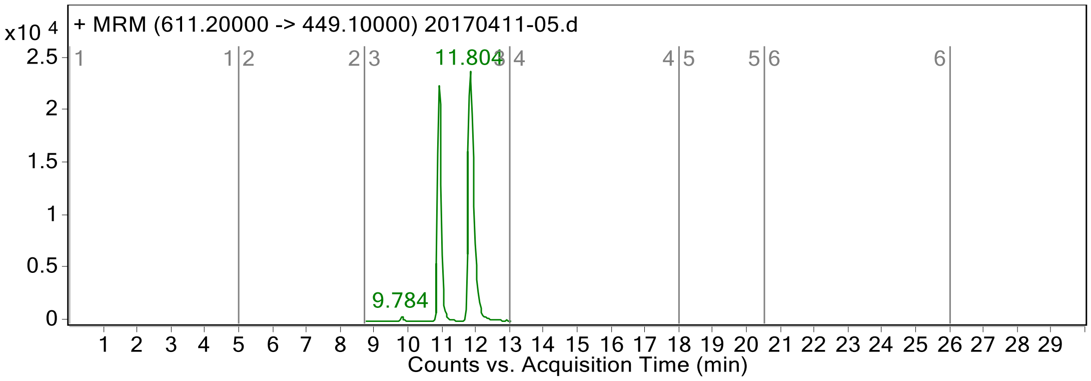
x10 4
+ MRM (611.20000 -> 449.10000) 20170411-05.d
11.804
9.784
Counts vs. Acquisition Time (min)

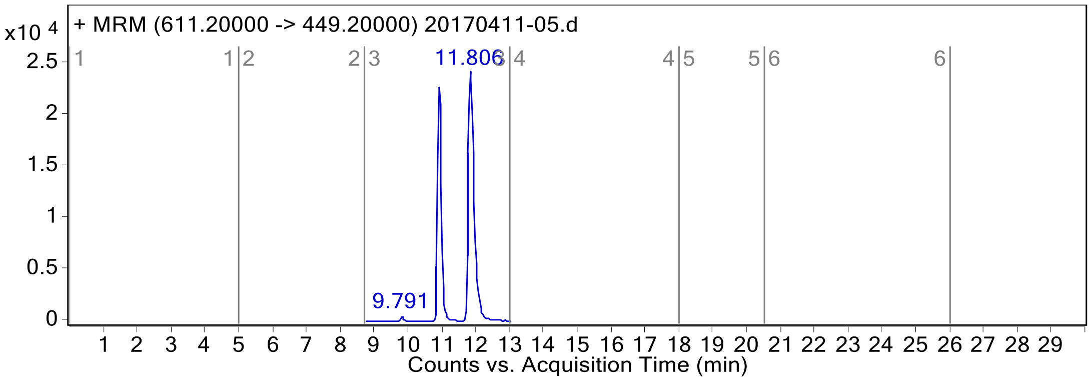
x10 4
+ MRM (611.20000 -> 449.20000) 20170411-05.d
11.806
9.791
Counts vs. Acquisition Time (min)

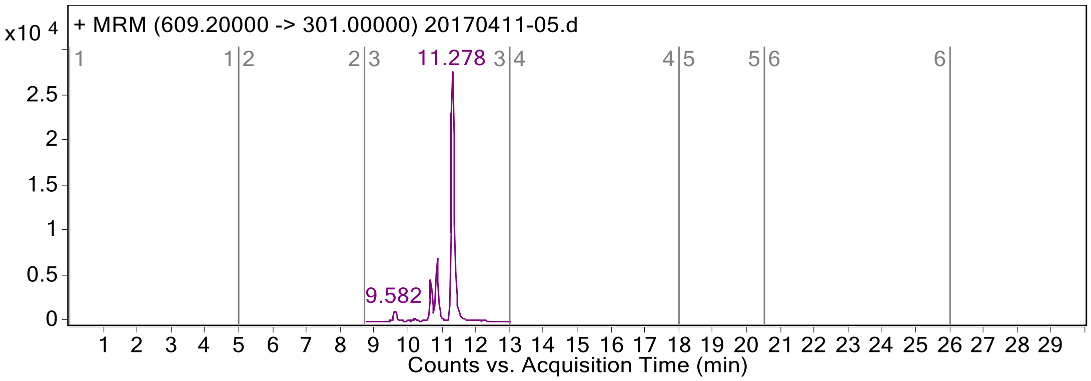
x10 4
+ MRM (609.20000 -> 301.00000) 20170411-05.d
11.278
9.582
Counts vs. Acquisition Time (min)

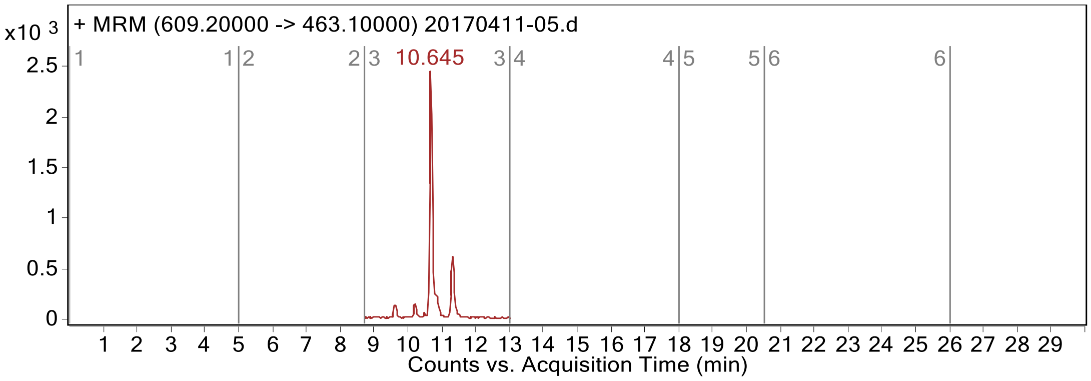
x10 3
+ MRM (609.20000 -> 463.10000) 20170411-05.d
10.645
Counts vs. Acquisition Time (min)

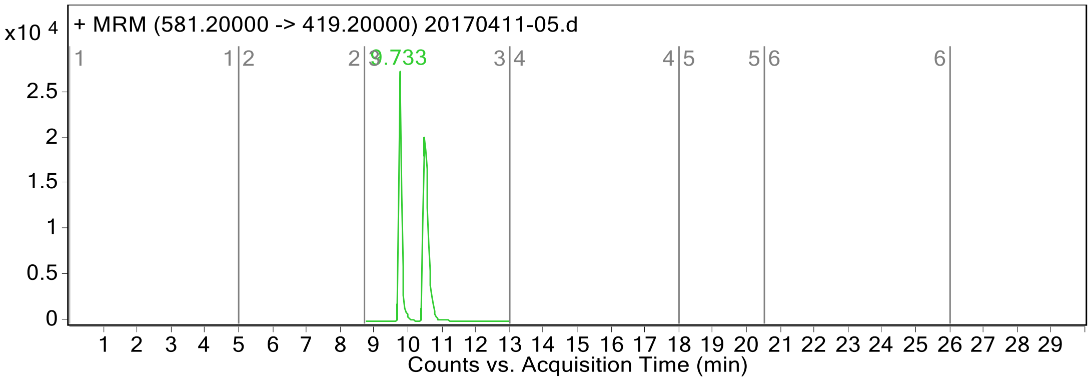
x10 4
+ MRM (581.20000 -> 419.20000) 20170411-05.d
9.733
Counts vs. Acquisition Time (min)

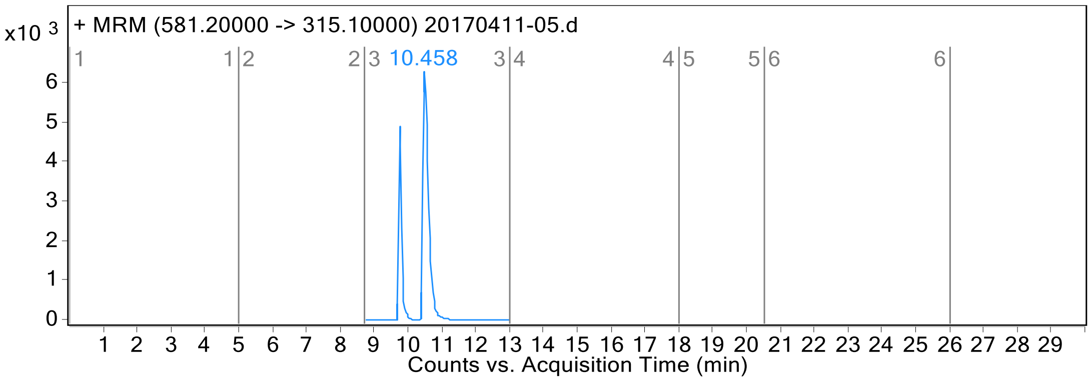
x10 3
+ MRM (581.20000 -> 315.10000) 20170411-05.d
10.458
Counts vs. Acquisition Time (min)

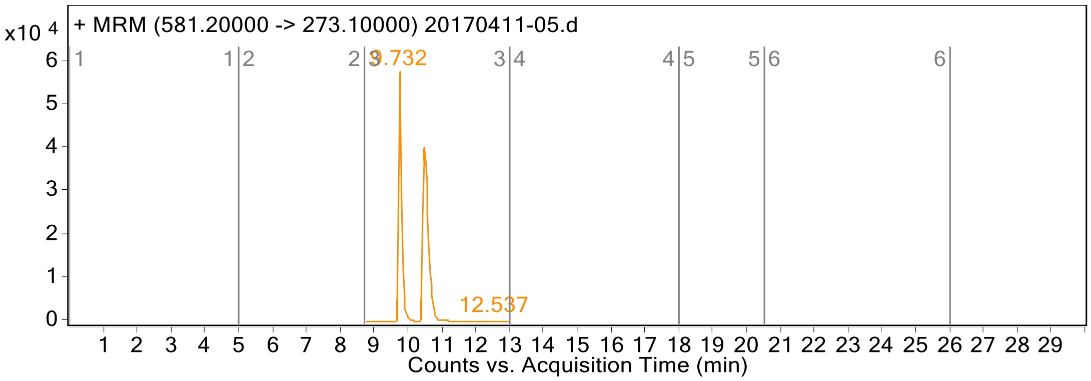
x10 4
+ MRM (581.20000 -> 273.10000) 20170411-05.d
9.732
12.537
Counts vs. Acquisition Time (min)

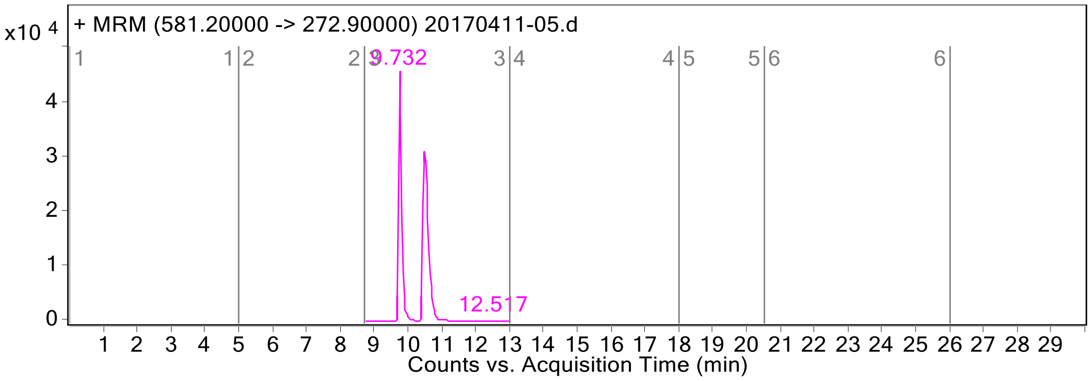
x10 4
+ MRM (581.20000 -> 272.90000) 20170411-05.d
9.732
12.517
Counts vs. Acquisition Time (min)

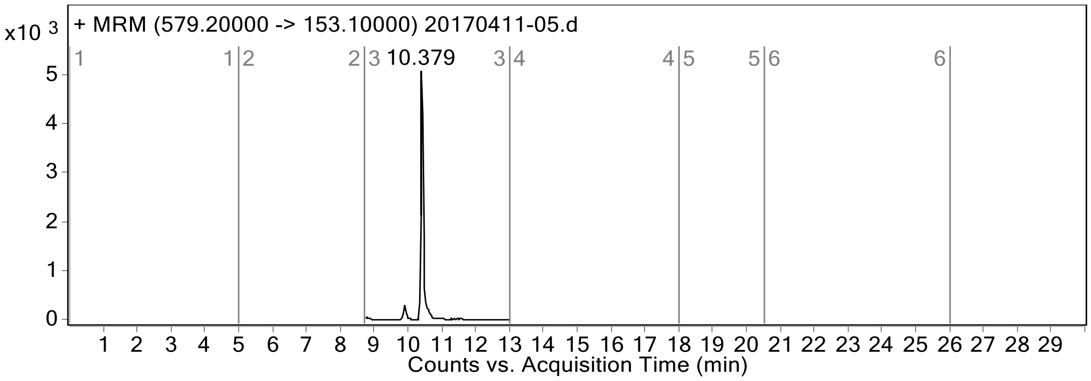
x10 3
+ MRM (579.20000 -> 153.10000) 20170411-05.d
10.379
Counts vs. Acquisition Time (min)

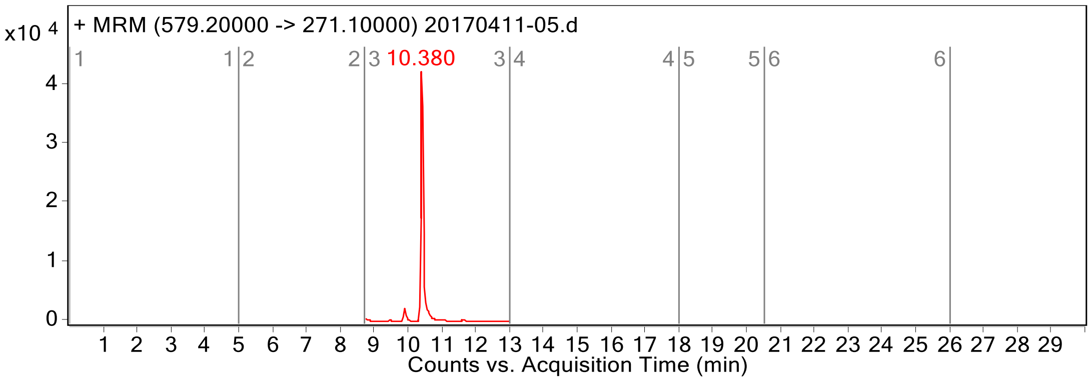
x10 4
+ MRM (579.20000 -> 271.10000) 20170411-05.d
10.380
Counts vs. Acquisition Time (min)

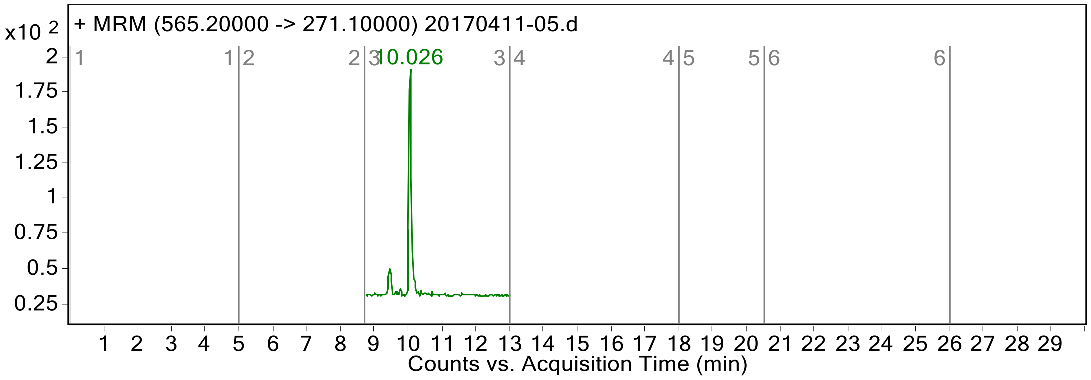

+ MRM (565.20000 -> 271.10000) 20170411-05.d
x10 2
10.026
Counts vs. Acquisition Time (min)

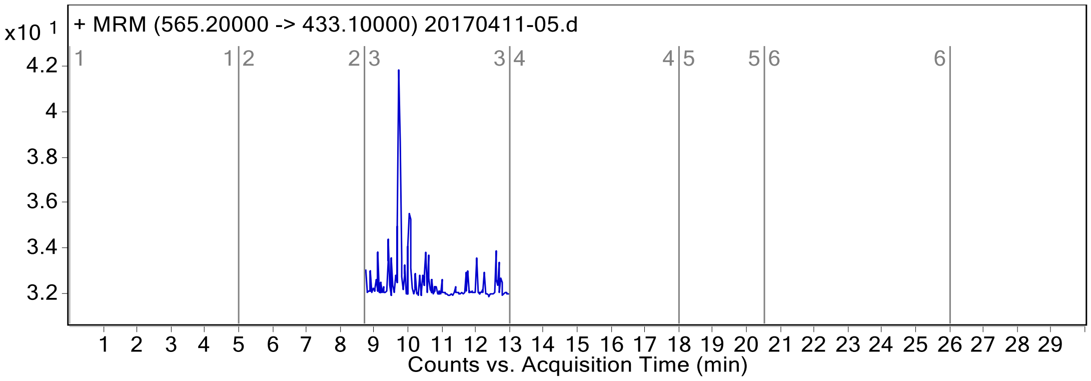

+ MRM (565.20000 -> 433.10000) 20170411-05.d
x10 1
Counts vs. Acquisition Time (min)

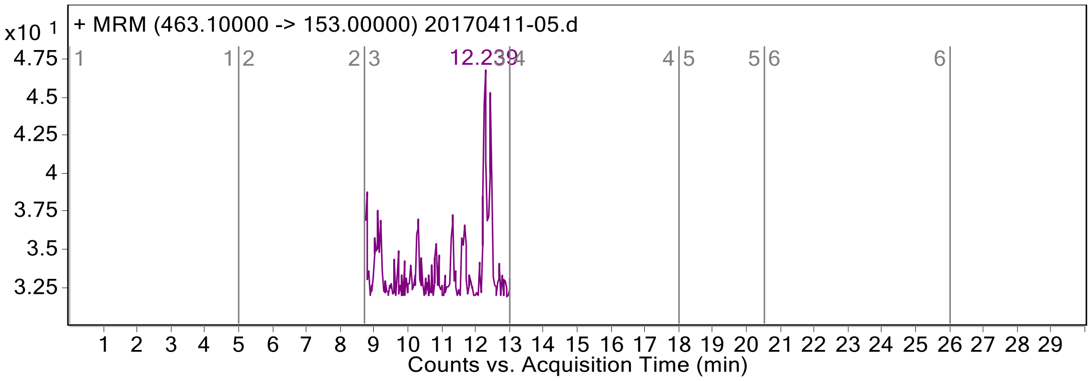

+ MRM (463.10000 -> 153.00000) 20170411-05.d
x10 1
12.239
Counts vs. Acquisition Time (min)

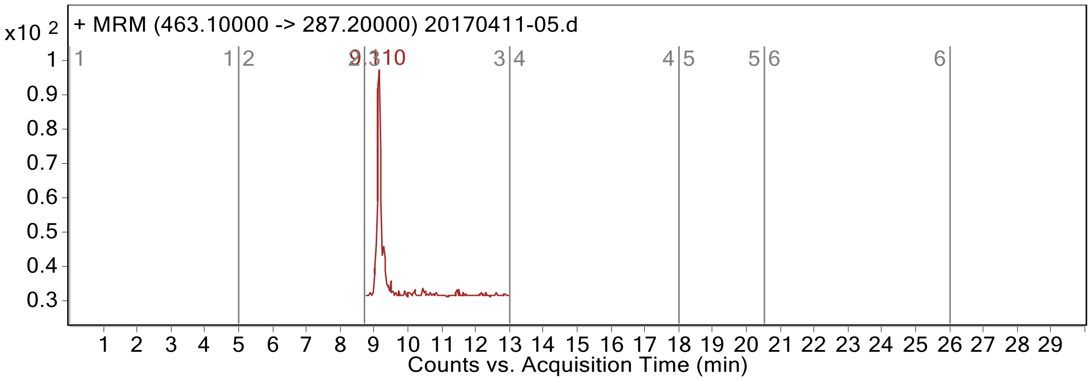

+ MRM (463.10000 -> 287.20000) 20170411-05.d
x10 2
9.310
Counts vs. Acquisition Time (min)

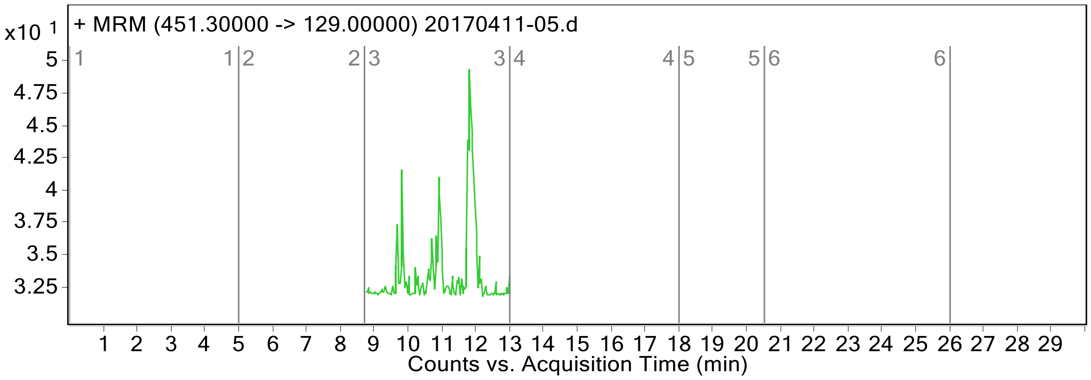
+ MRM (451.30000 -> 129.00000) 20170411-05.d
x10 1
Counts vs. Acquisition Time (min)

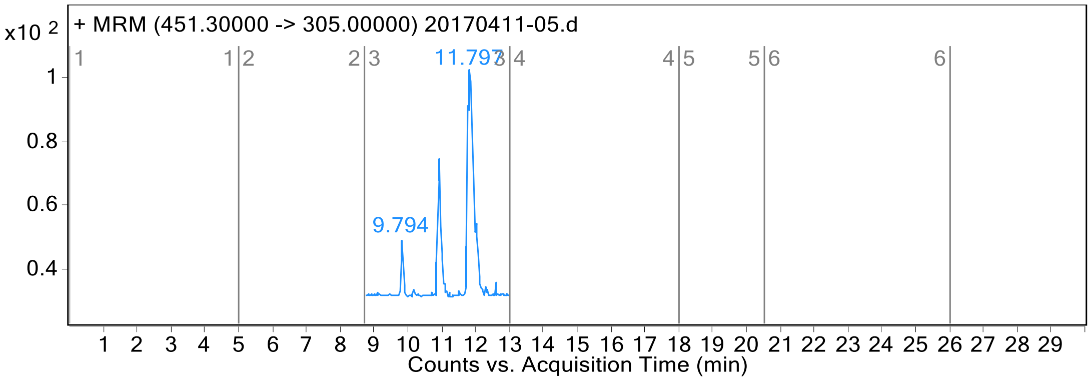
+ MRM (451.30000 -> 305.00000) 20170411-05.d
x10 2
11.797
9.794
Counts vs. Acquisition Time (min)

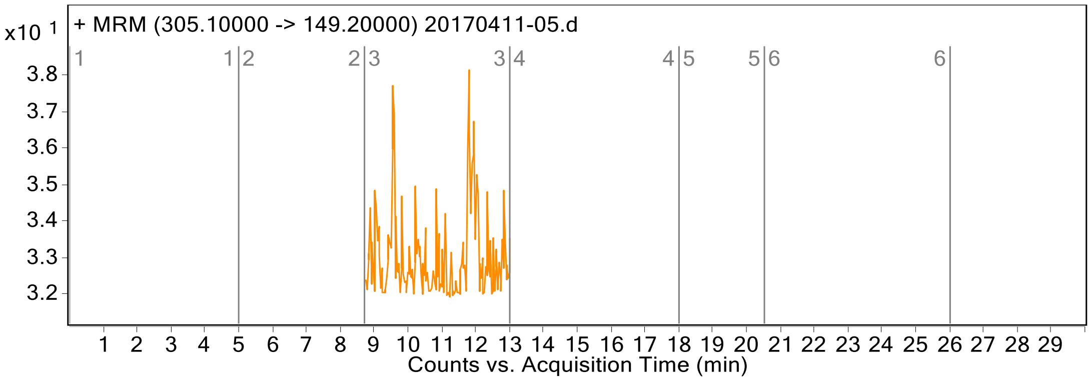
+ MRM (305.10000 -> 149.20000) 20170411-05.d
x10 1
Counts vs. Acquisition Time (min)

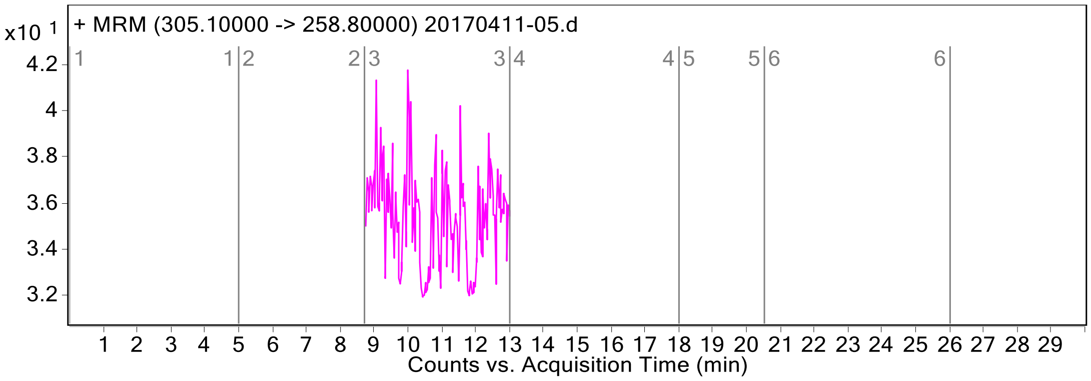
+ MRM (305.10000 -> 258.80000) 20170411-05.d
x10 1
Counts vs. Acquisition Time (min)

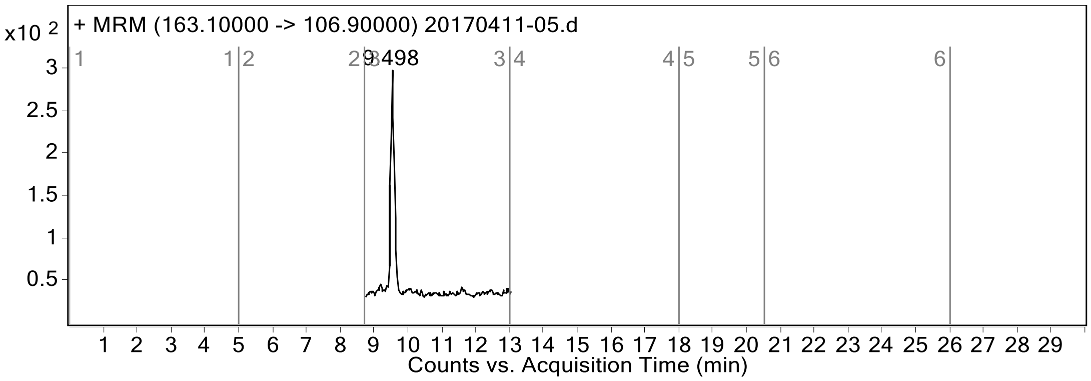

x10 2
+ MRM (163.10000 -> 106.90000) 20170411-05.d
9.498
Counts vs. Acquisition Time (min)

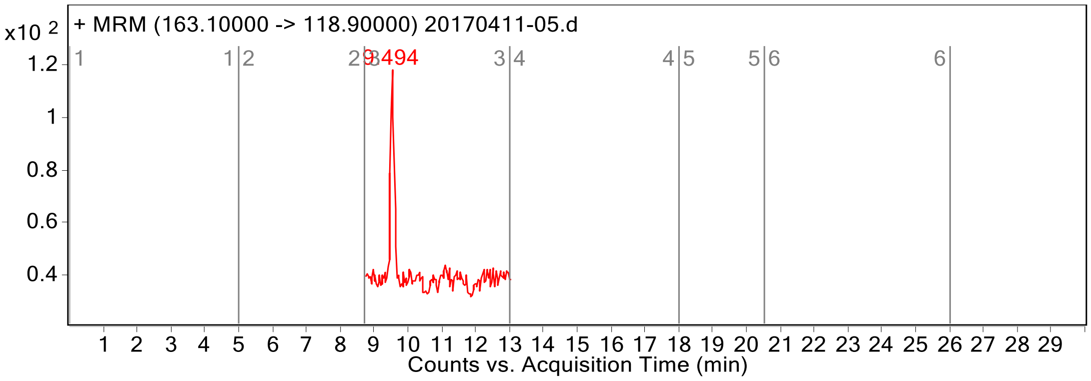

x10 2
+ MRM (163.10000 -> 118.90000) 20170411-05.d
9.494
Counts vs. Acquisition Time (min)

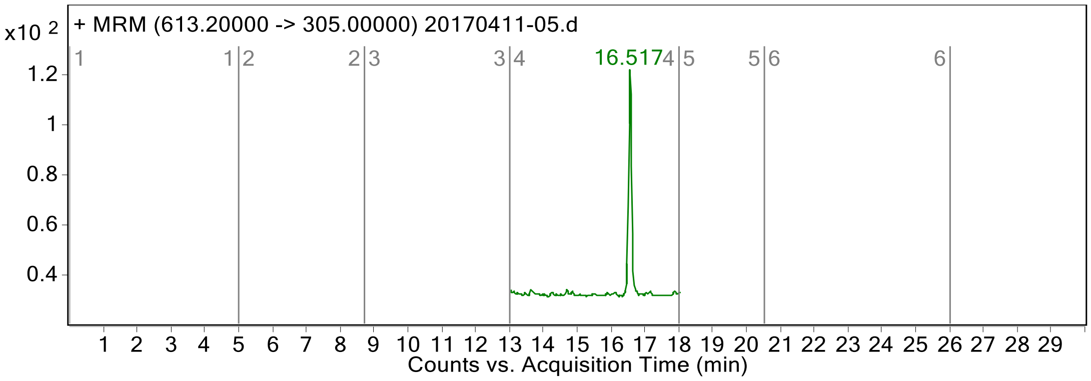

x10 2
+ MRM (613.20000 -> 305.00000) 20170411-05.d
16.517
Counts vs. Acquisition Time (min)

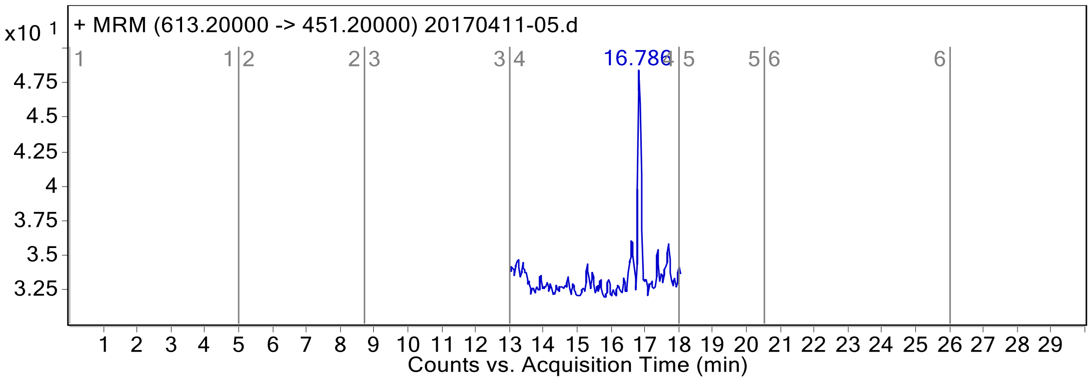

x10 1
+ MRM (613.20000 -> 451.20000) 20170411-05.d
16.786
Counts vs. Acquisition Time (min)

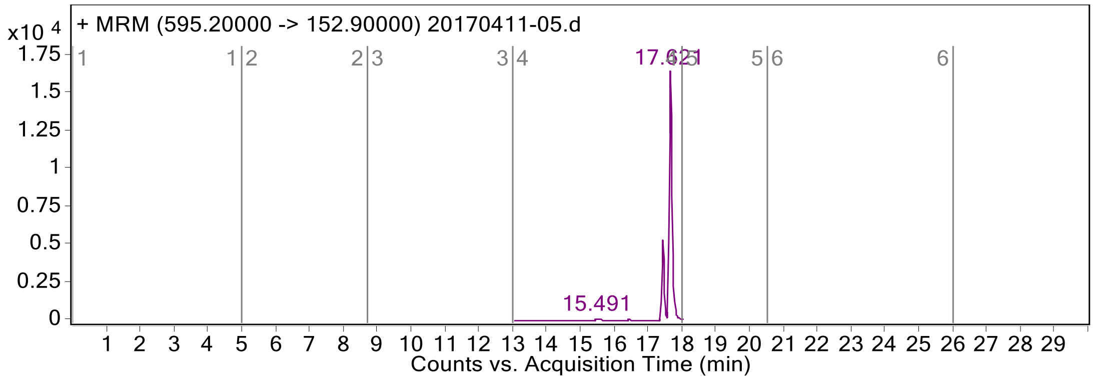

x10 4
+ MRM (595.20000 -> 152.90000) 20170411-05.d
15.491
17.621
Counts vs. Acquisition Time (min)

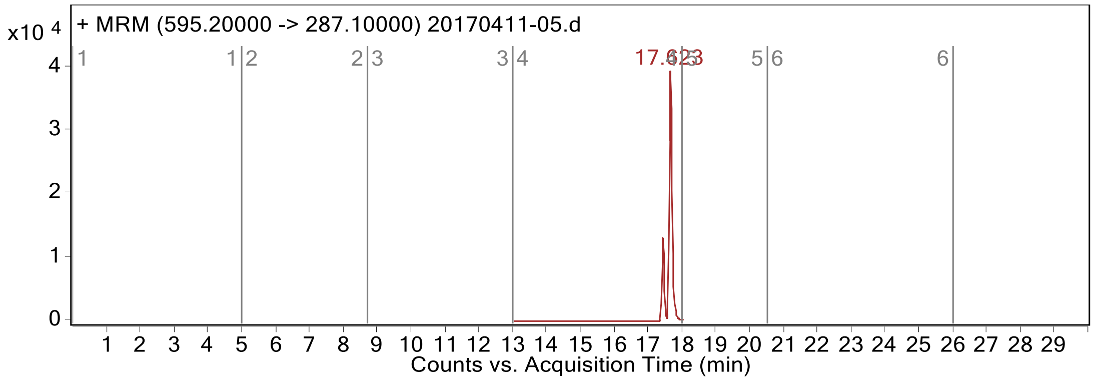

x10 4
+ MRM (595.20000 -> 287.10000) 20170411-05.d
17.623
Counts vs. Acquisition Time (min)

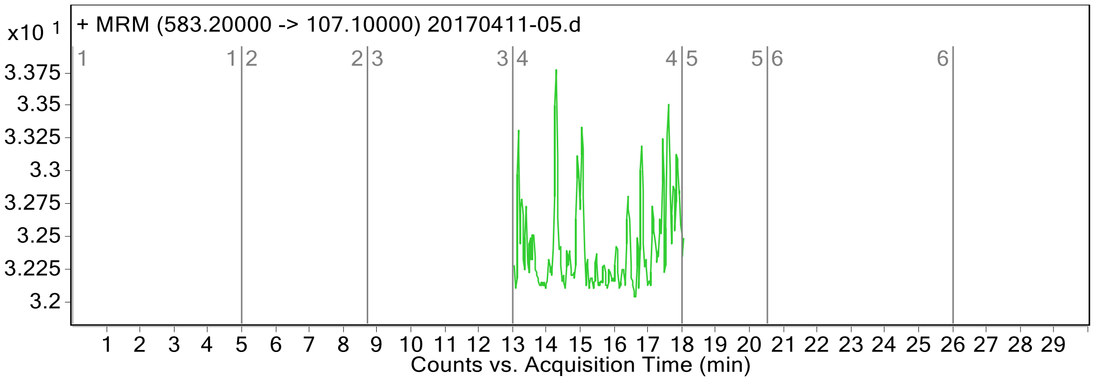

x10 1
+ MRM (583.20000 -> 107.10000) 20170411-05.d
Counts vs. Acquisition Time (min)

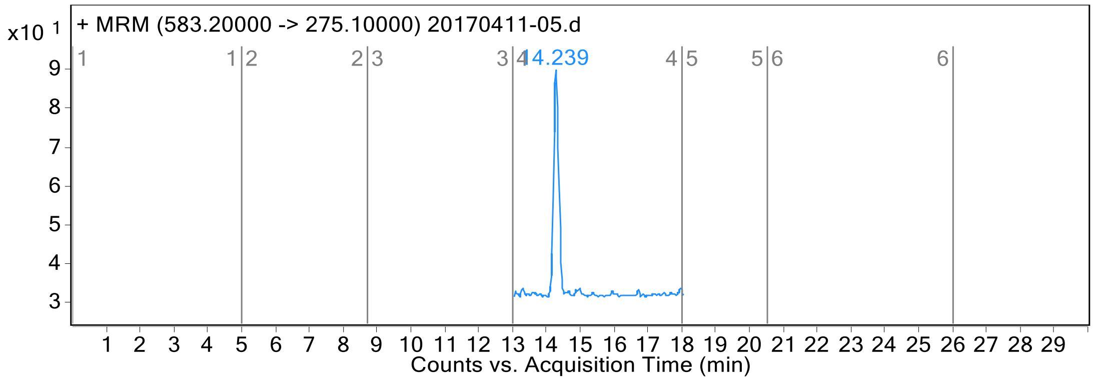

x10 1
+ MRM (583.20000 -> 275.10000) 20170411-05.d
14.239
Counts vs. Acquisition Time (min)

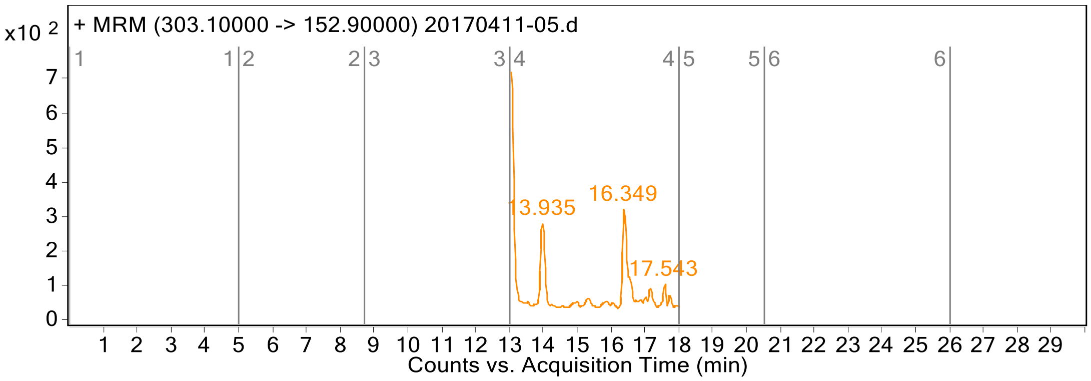
x10 2
+ MRM (303.10000 -> 152.90000) 20170411-05.d
13.935
16.349
17.543
Counts vs. Acquisition Time (min)

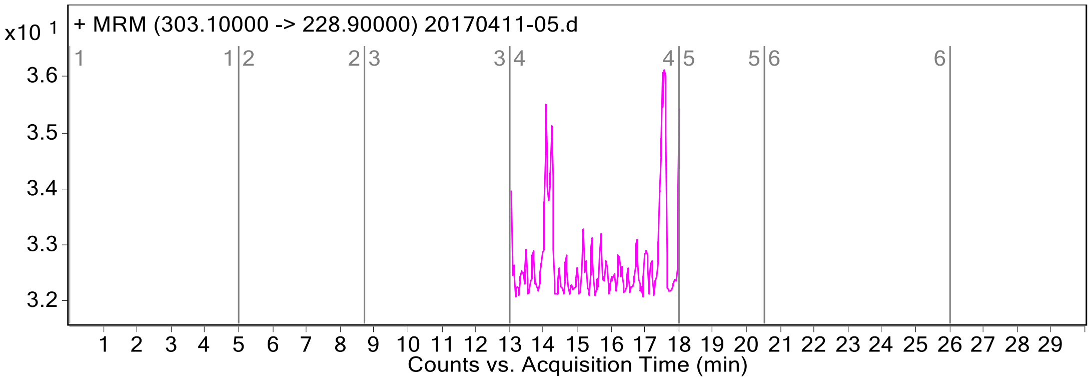
x10 1
+ MRM (303.10000 -> 228.90000) 20170411-05.d
Counts vs. Acquisition Time (min)

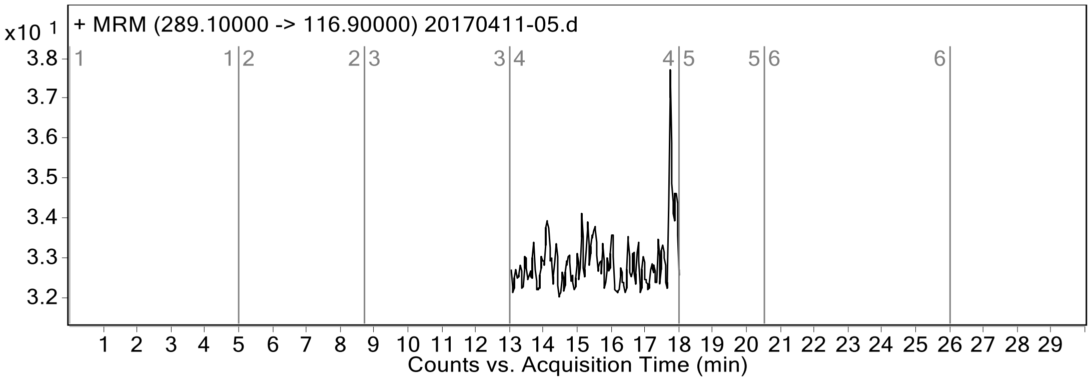
x10 1
+ MRM (289.10000 -> 116.90000) 20170411-05.d
Counts vs. Acquisition Time (min)

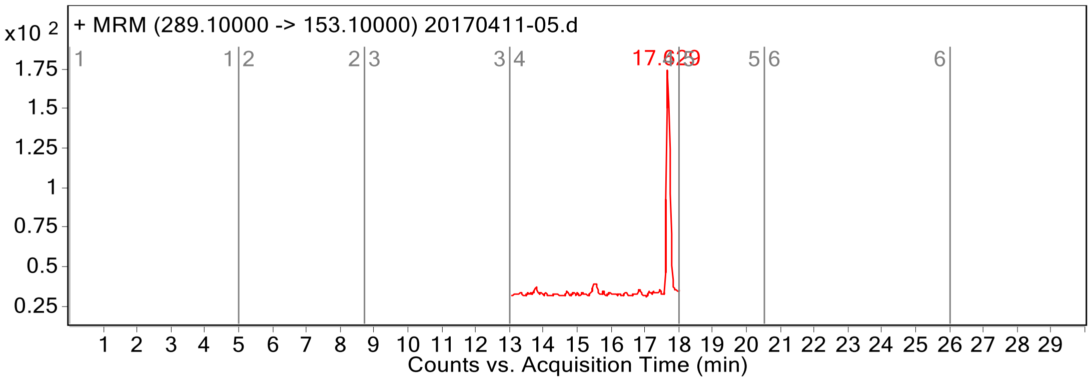
x10 2
+ MRM (289.10000 -> 153.10000) 20170411-05.d
17.629
Counts vs. Acquisition Time (min)

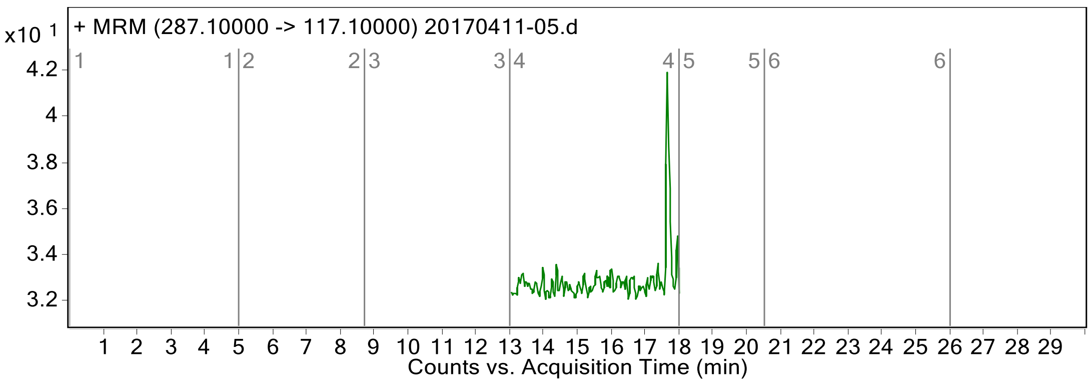
+ MRM (287.10000 -> 117.10000) 20170411-05.d
x10 1
Counts vs. Acquisition Time (min)

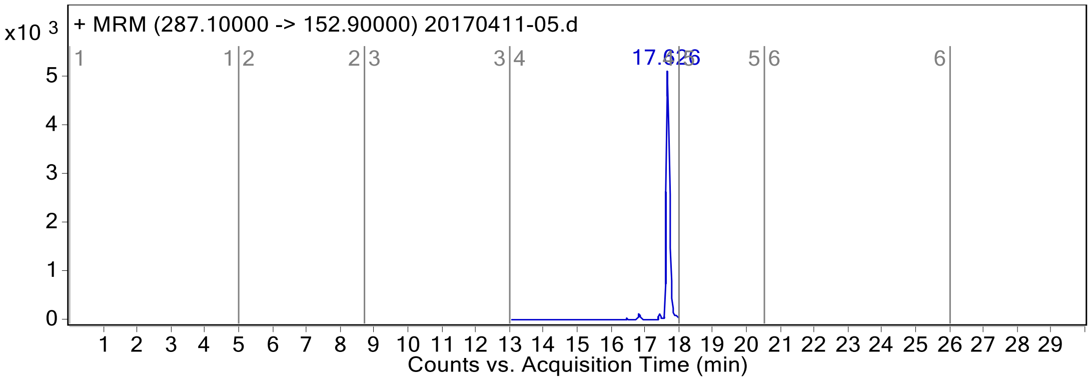
+ MRM (287.10000 -> 152.90000) 20170411-05.d
x10 3
Counts vs. Acquisition Time (min)

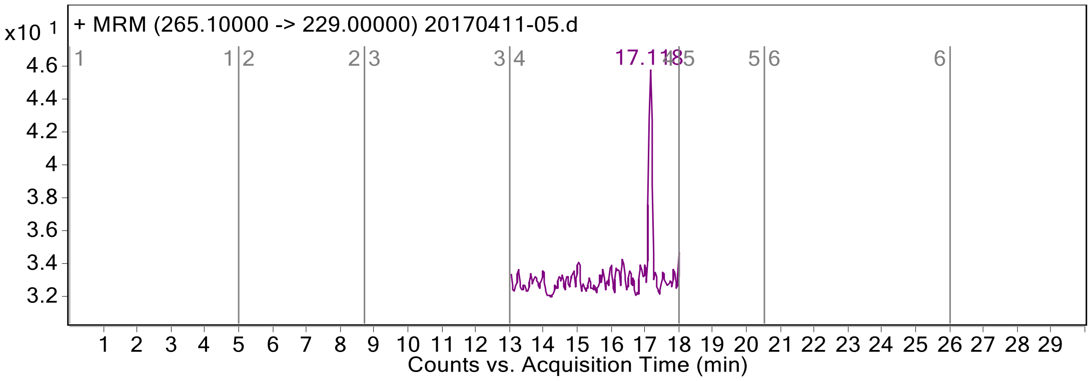
+ MRM (265.10000 -> 229.00000) 20170411-05.d
x10 1
Counts vs. Acquisition Time (min)

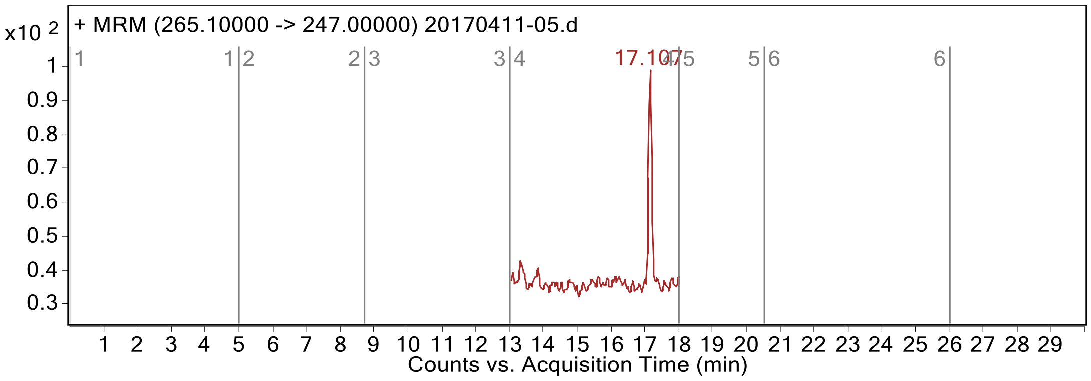
+ MRM (265.10000 -> 247.00000) 20170411-05.d
x10 2
Counts vs. Acquisition Time (min)

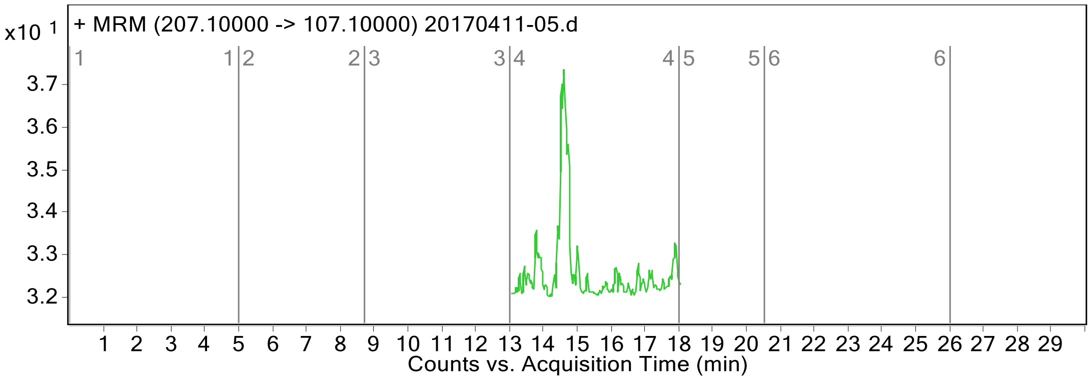
x10 1
+ MRM (207.10000 -> 107.10000) 20170411-05.d
3.7
3.6
3.5
3.4
3.3
3.2
1 2 3 4 5 6 7 8 9 10 11 12 13 14 15 16 17 18 19 20 21 22 23 24 25 26 27 28 29
Counts vs. Acquisition Time (min)

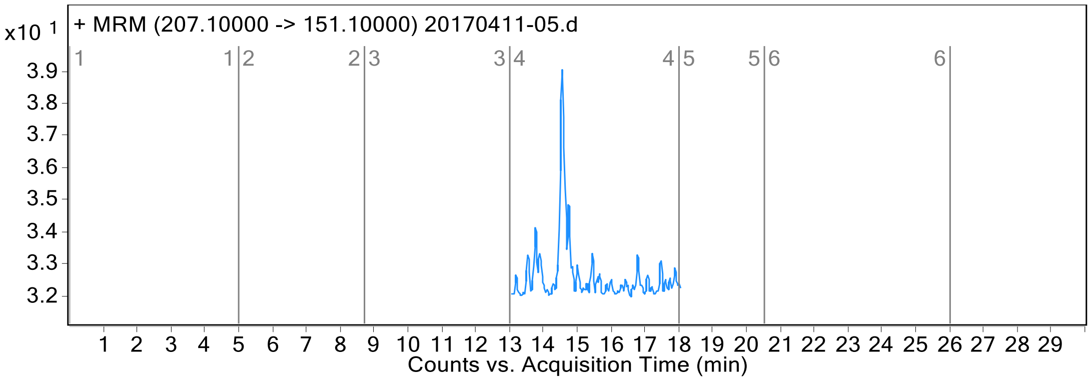
x10 1
+ MRM (207.10000 -> 151.10000) 20170411-05.d
3.9
3.8
3.7
3.6
3.5
3.4
3.3
3.2
1 2 3 4 5 6 7 8 9 10 11 12 13 14 15 16 17 18 19 20 21 22 23 24 25 26 27 28 29
Counts vs. Acquisition Time (min)

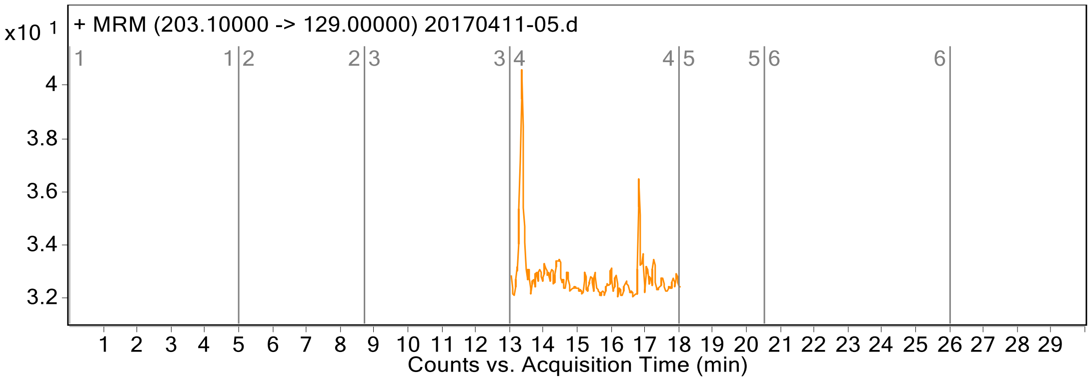
x10 1
+ MRM (203.10000 -> 129.00000) 20170411-05.d
4
3.8
3.6
3.4
3.2
1 2 3 4 5 6 7 8 9 10 11 12 13 14 15 16 17 18 19 20 21 22 23 24 25 26 27 28 29
Counts vs. Acquisition Time (min)

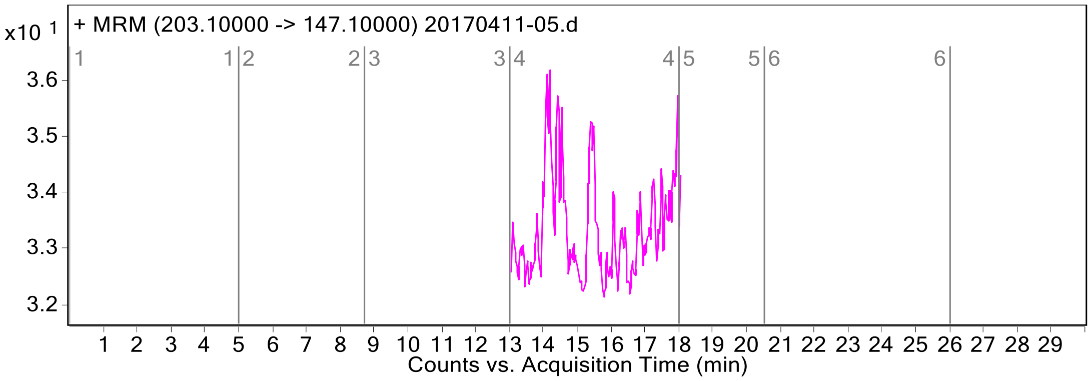
x10 1
+ MRM (203.10000 -> 147.10000) 20170411-05.d
3.6
3.5
3.4
3.3
3.2
1 2 3 4 5 6 7 8 9 10 11 12 13 14 15 16 17 18 19 20 21 22 23 24 25 26 27 28 29
Counts vs. Acquisition Time (min)

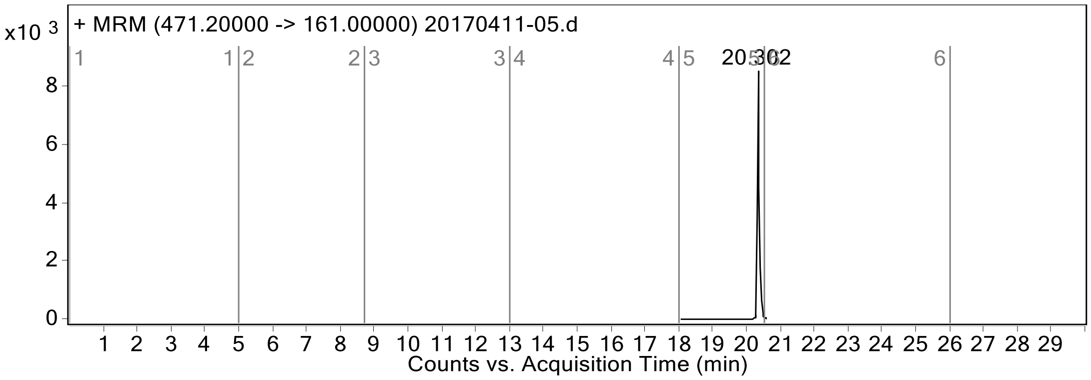
+ MRM (471.20000 -> 161.00000) 20170411-05.d
x10 3
20.302
Counts vs. Acquisition Time (min)

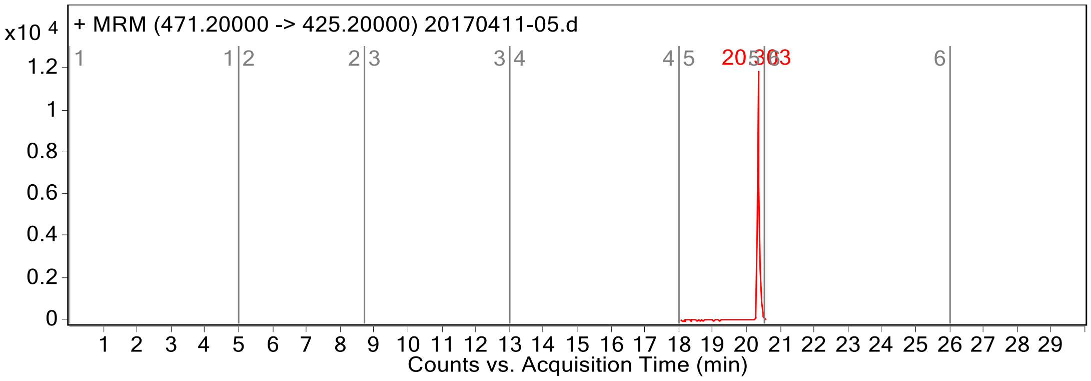
+ MRM (471.20000 -> 425.20000) 20170411-05.d
x10 4
20.303
Counts vs. Acquisition Time (min)

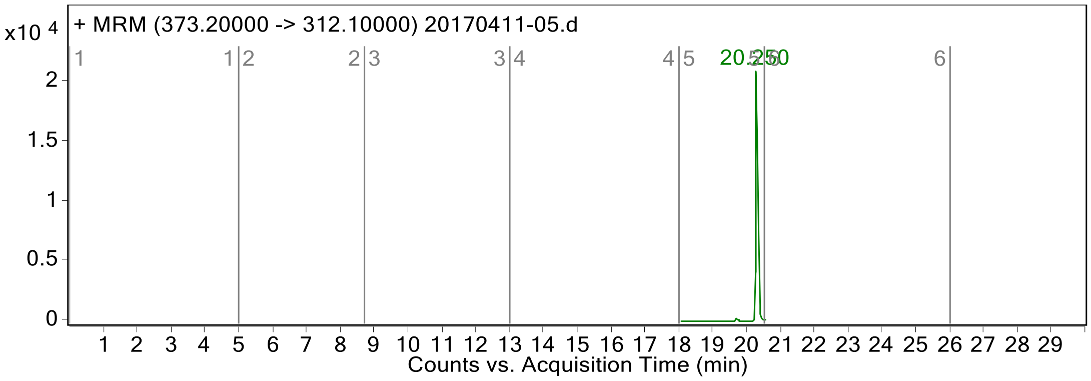
+ MRM (373.20000 -> 312.10000) 20170411-05.d
x10 4
20.250
Counts vs. Acquisition Time (min)

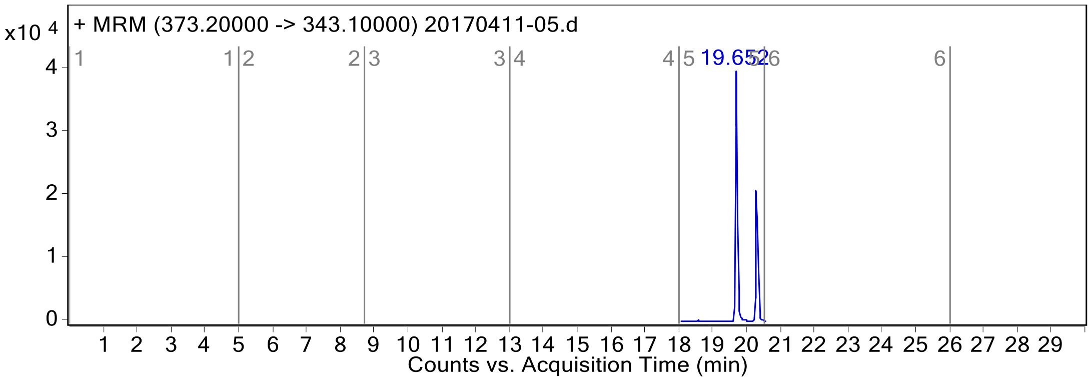
+ MRM (373.20000 -> 343.10000) 20170411-05.d
x10 4
19.652
Counts vs. Acquisition Time (min)

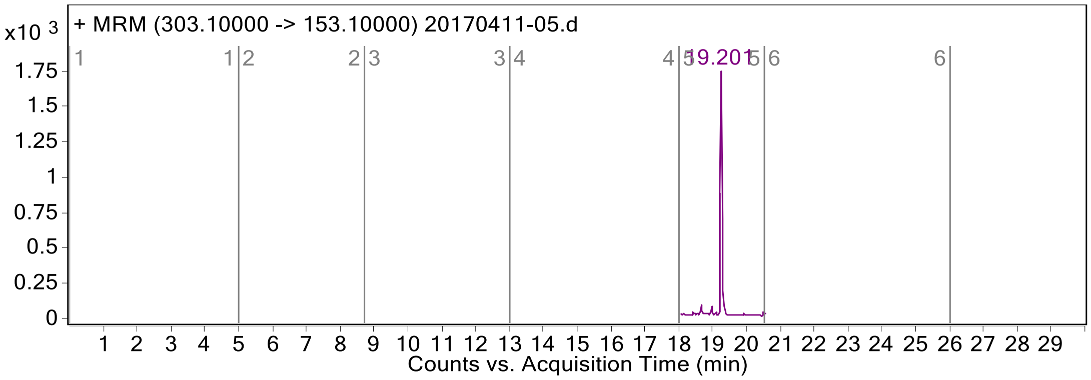
x10 3
+ MRM (303.10000 -> 153.10000) 20170411-05.d
19.201
Counts vs. Acquisition Time (min)

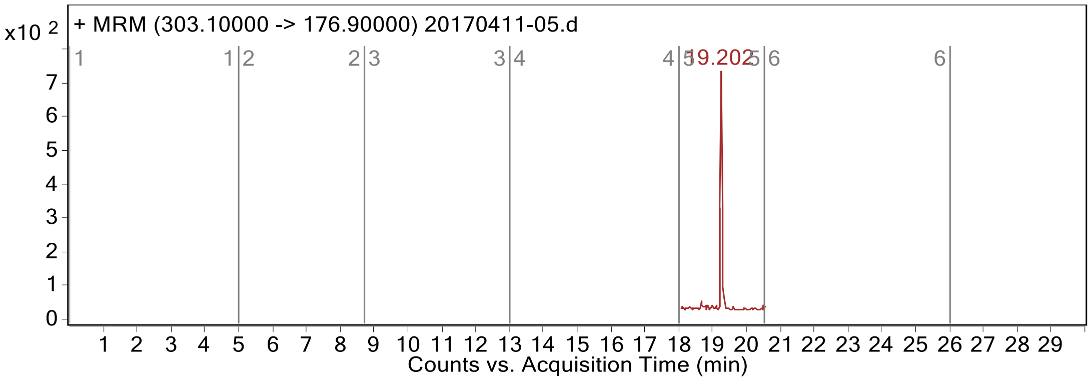
x10 2
+ MRM (303.10000 -> 176.90000) 20170411-05.d
19.202
Counts vs. Acquisition Time (min)

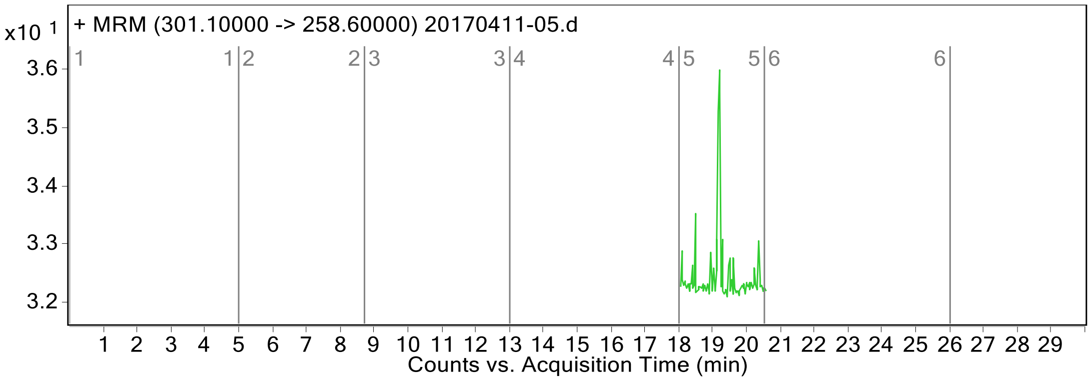
x10 1
+ MRM (301.10000 -> 258.60000) 20170411-05.d
Counts vs. Acquisition Time (min)

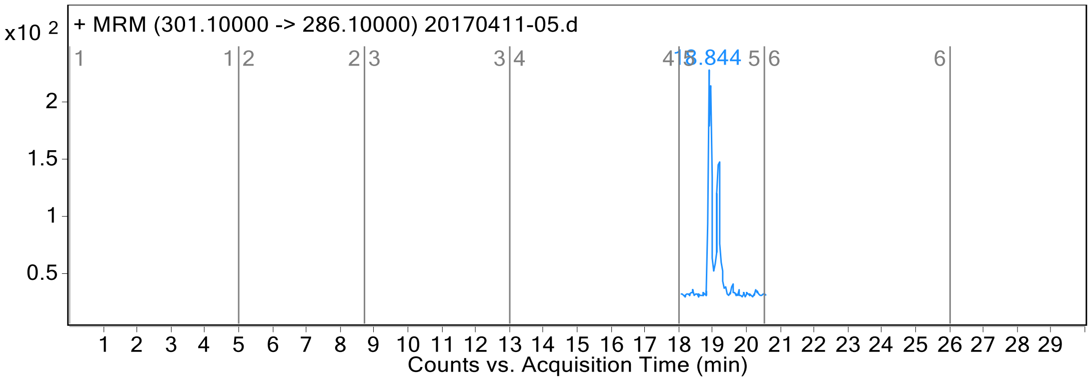
x10 2
+ MRM (301.10000 -> 286.10000) 20170411-05.d
18.844
Counts vs. Acquisition Time (min)

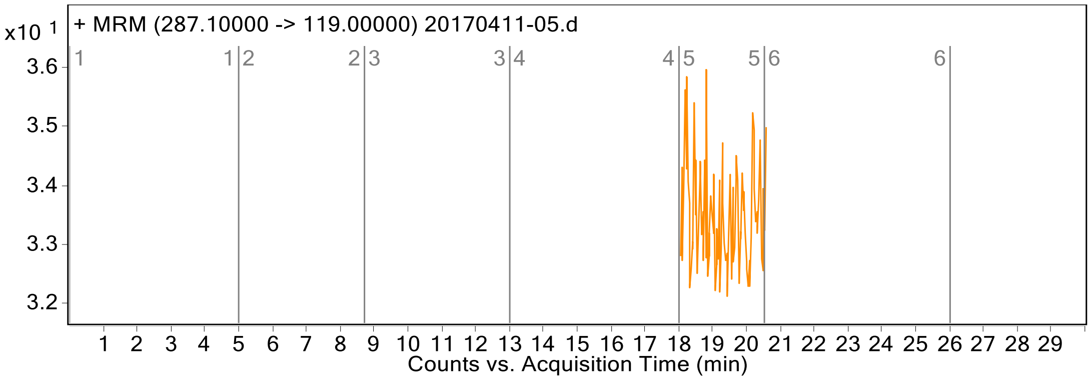
x10 1
+ MRM (287.10000 -> 119.00000) 20170411-05.d
Counts vs. Acquisition Time (min)

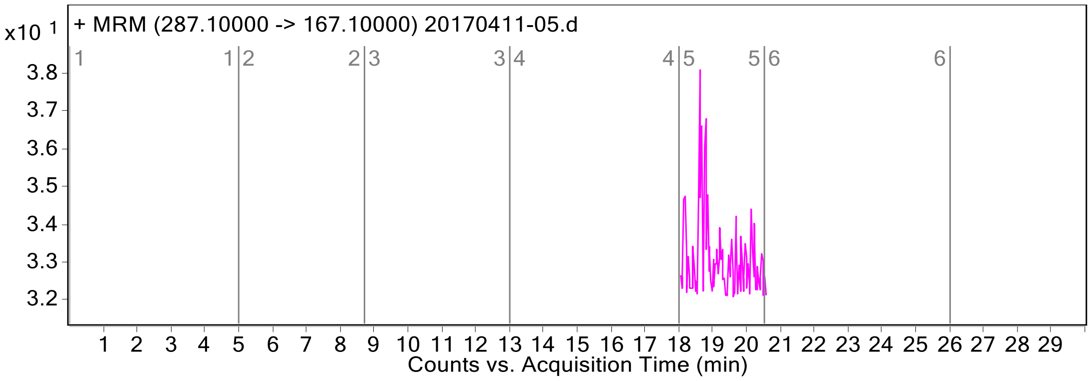
x10 1
+ MRM (287.10000 -> 167.10000) 20170411-05.d
Counts vs. Acquisition Time (min)

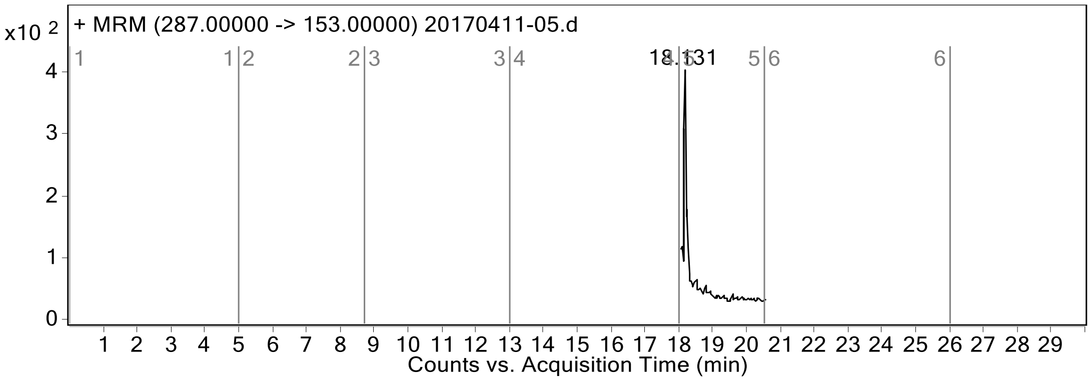
x10 2
+ MRM (287.00000 -> 153.00000) 20170411-05.d
18.131
Counts vs. Acquisition Time (min)

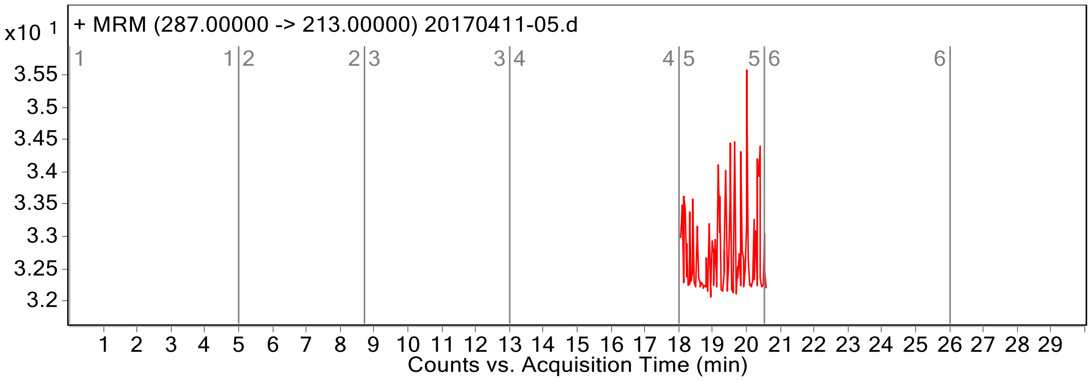
x10 1
+ MRM (287.00000 -> 213.00000) 20170411-05.d
Counts vs. Acquisition Time (min)

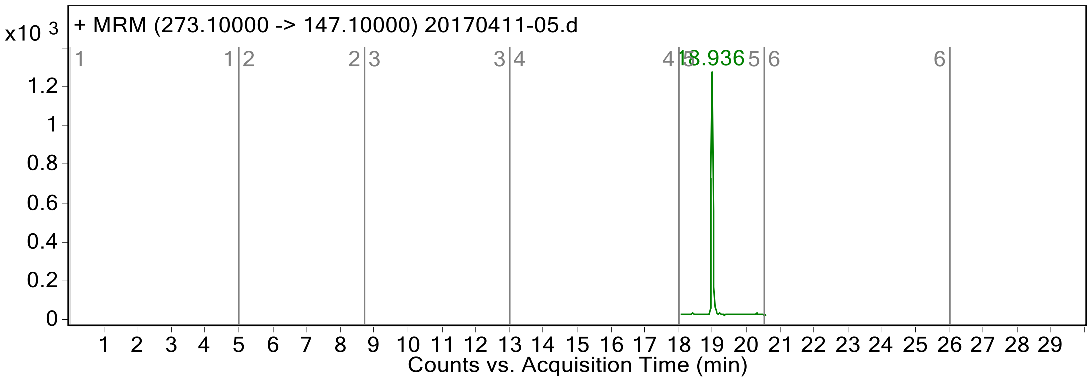
x10 3
+ MRM (273.10000 -> 147.10000) 20170411-05.d
18.936
Counts vs. Acquisition Time (min)

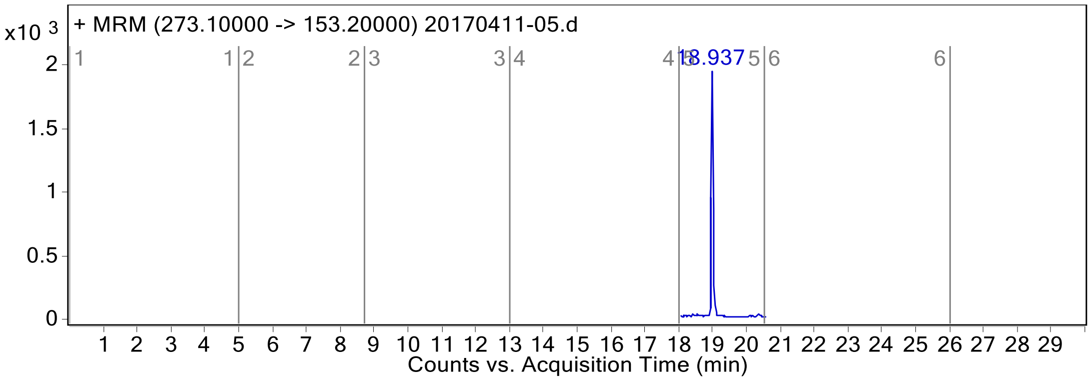
x10 3
+ MRM (273.10000 -> 153.20000) 20170411-05.d
18.937
Counts vs. Acquisition Time (min)

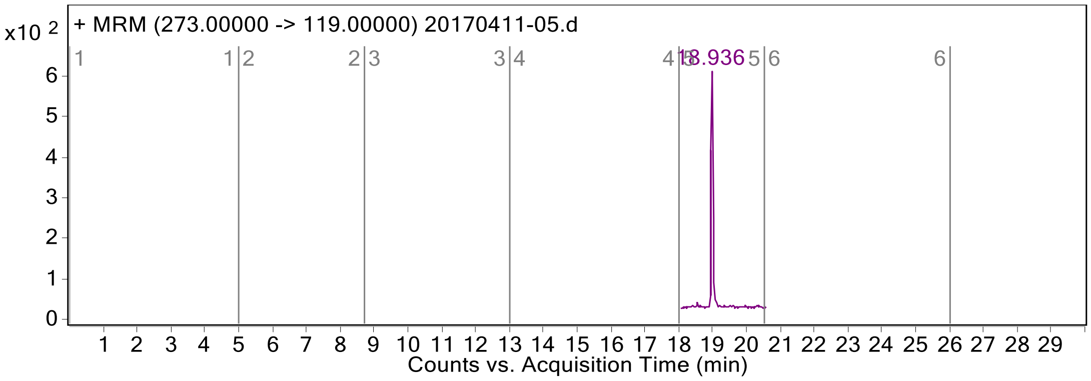
x10 2
+ MRM (273.00000 -> 119.00000) 20170411-05.d
18.936
Counts vs. Acquisition Time (min)

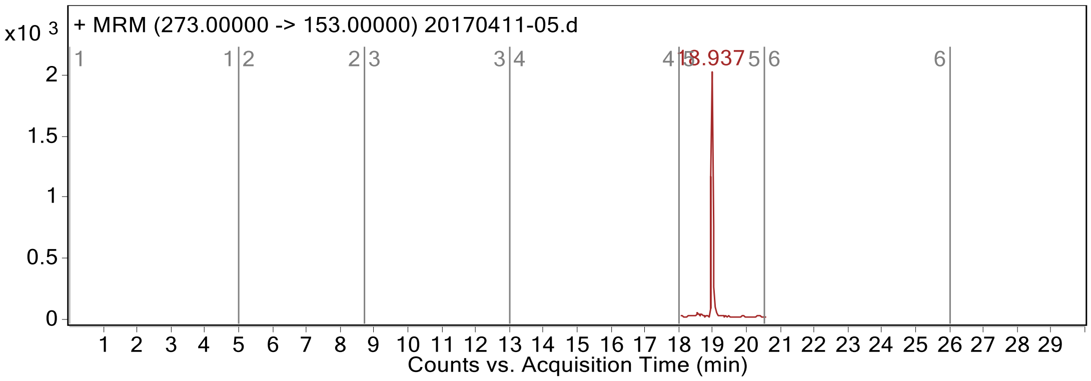
x10 3
+ MRM (273.00000 -> 153.00000) 20170411-05.d
18.937
Counts vs. Acquisition Time (min)

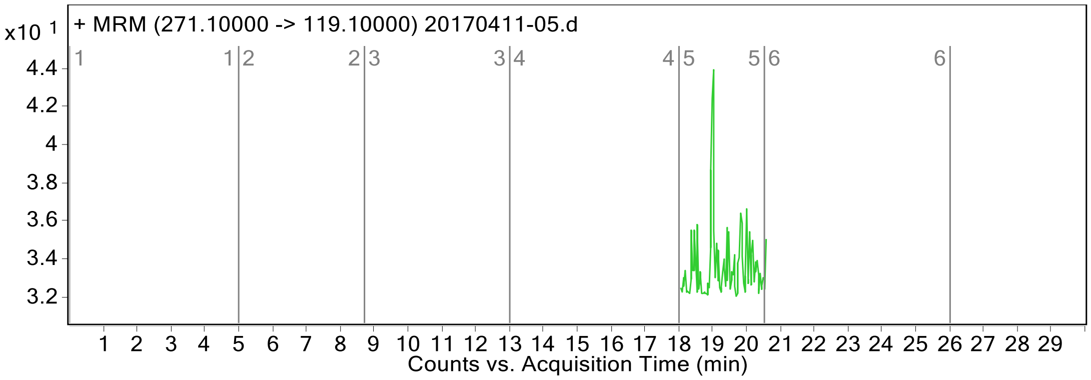
x10 1
+ MRM (271.10000 -> 119.10000) 20170411-05.d
Counts vs. Acquisition Time (min)

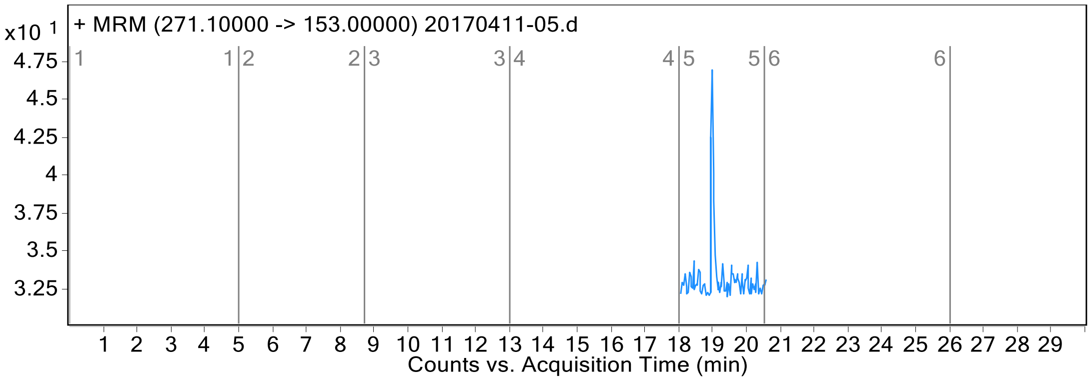
x10 1
+ MRM (271.10000 -> 153.00000) 20170411-05.d
Counts vs. Acquisition Time (min)

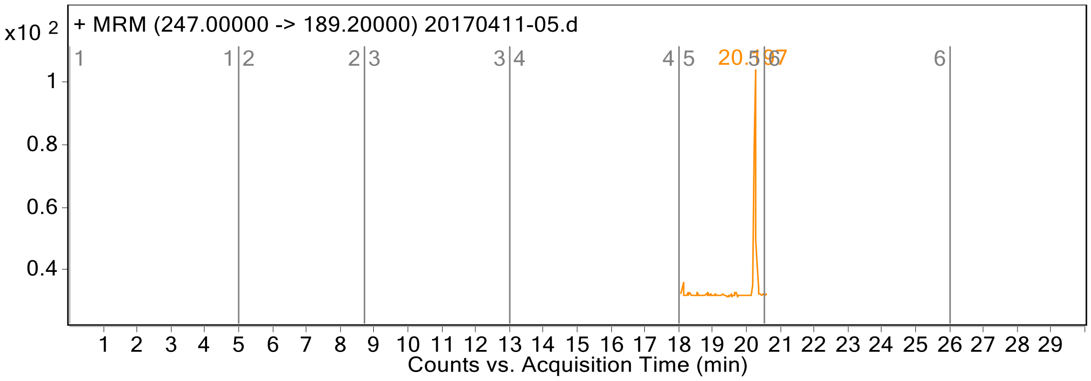
x10 2
+ MRM (247.00000 -> 189.20000) 20170411-05.d
Counts vs. Acquisition Time (min)

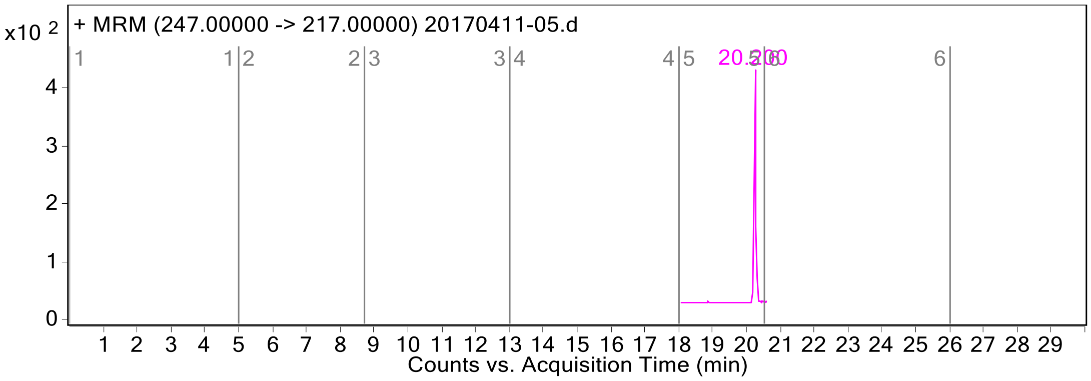
x10 2
+ MRM (247.00000 -> 217.00000) 20170411-05.d
Counts vs. Acquisition Time (min)

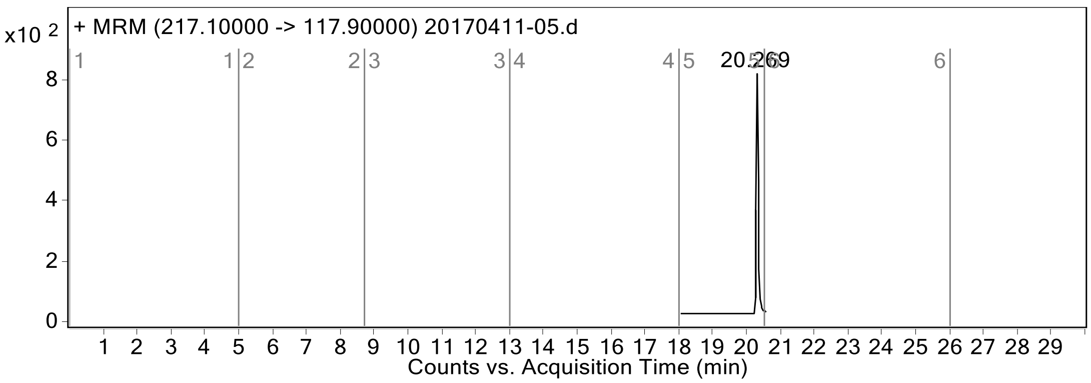
+ MRM (217.10000 -> 117.90000) 20170411-05.d
20.269
x10 2
Counts vs. Acquisition Time (min)

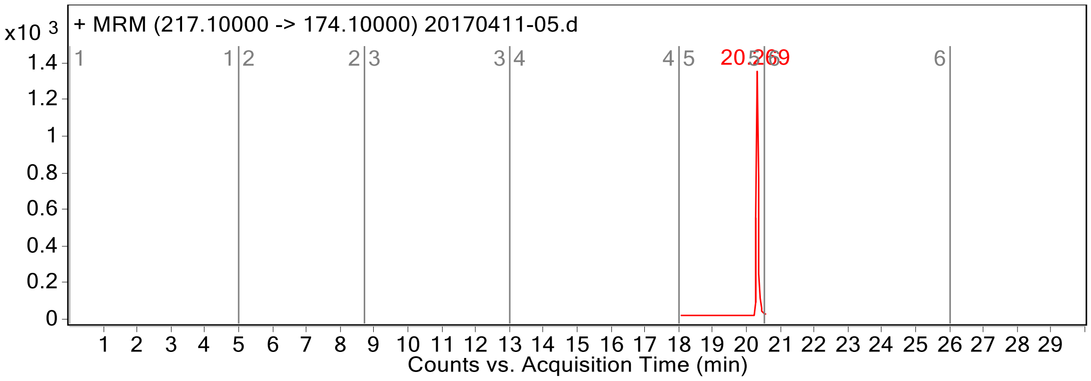
+ MRM (217.10000 -> 174.10000) 20170411-05.d
20.269
x10 3
Counts vs. Acquisition Time (min)

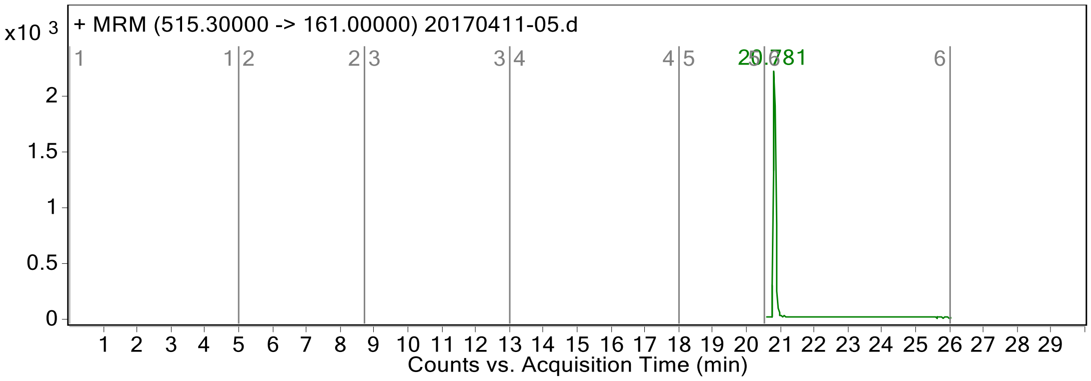
+ MRM (515.30000 -> 161.00000) 20170411-05.d
20.781
x10 3
Counts vs. Acquisition Time (min)

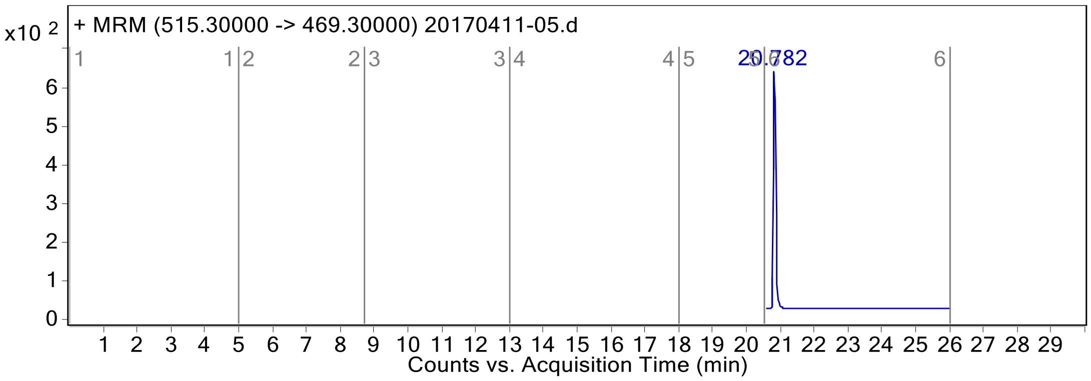
+ MRM (515.30000 -> 469.30000) 20170411-05.d
20.782
x10 2
Counts vs. Acquisition Time (min)

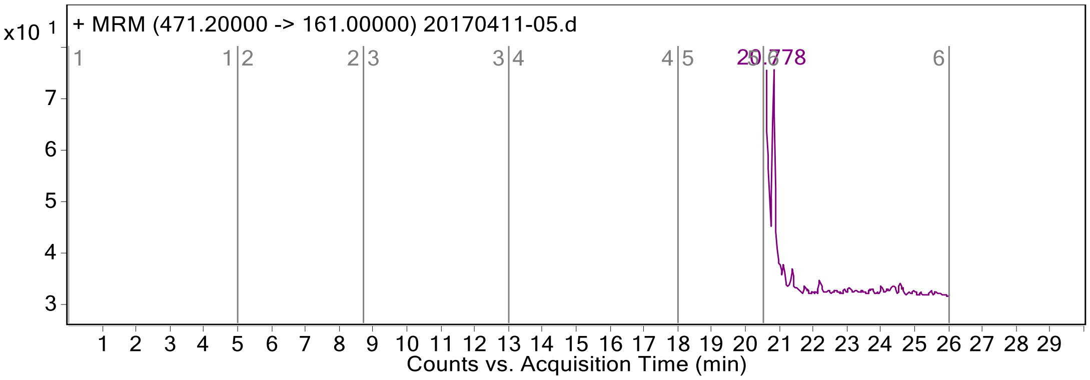
x10 1
+ MRM (471.20000 -> 161.00000) 20170411-05.d
20.778
Counts vs. Acquisition Time (min)

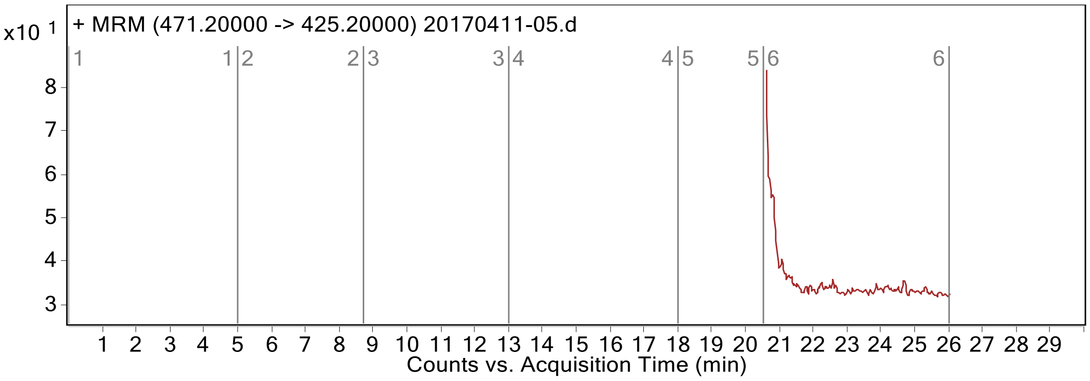
x10 1
+ MRM (471.20000 -> 425.20000) 20170411-05.d
Counts vs. Acquisition Time (min)

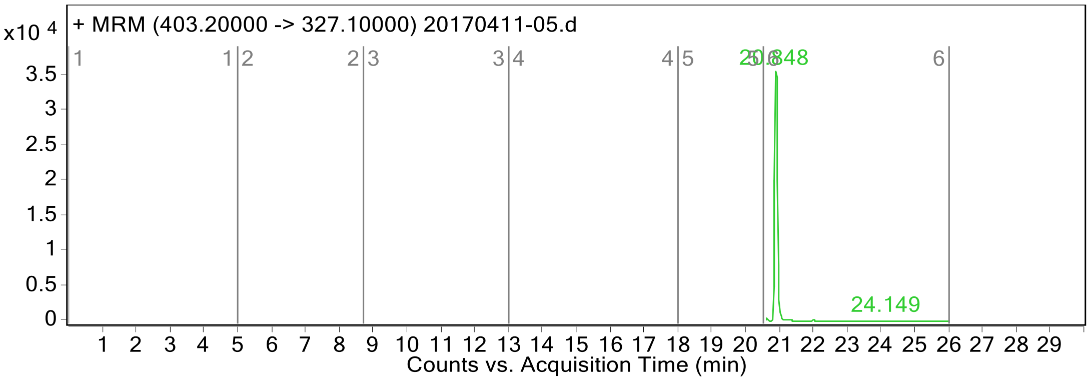
x10 4
+ MRM (403.20000 -> 327.10000) 20170411-05.d
20.848
24.149
Counts vs. Acquisition Time (min)

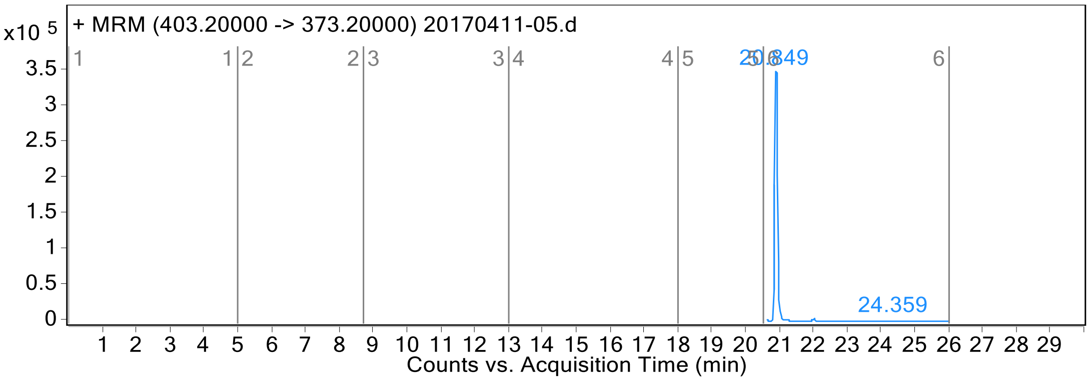
x10 5
+ MRM (403.20000 -> 373.20000) 20170411-05.d
20.849
24.359
Counts vs. Acquisition Time (min)

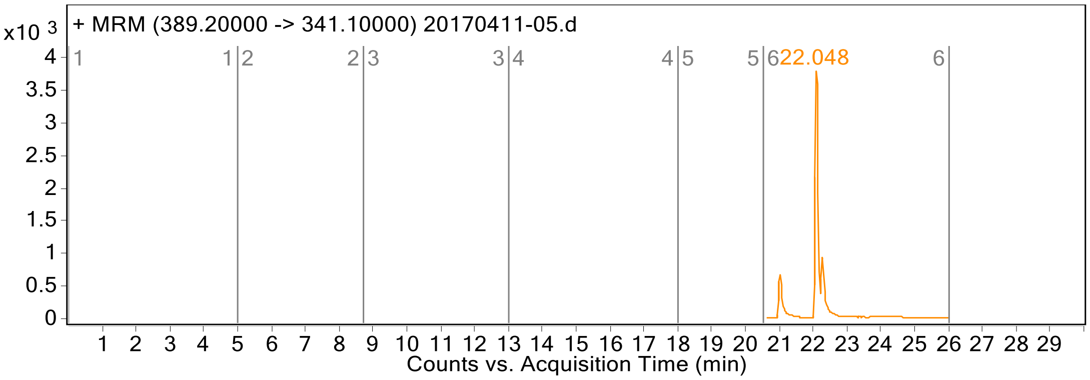
+ MRM (389.20000 -> 341.10000) 20170411-05.d
x10 3
22.048
Counts vs. Acquisition Time (min)

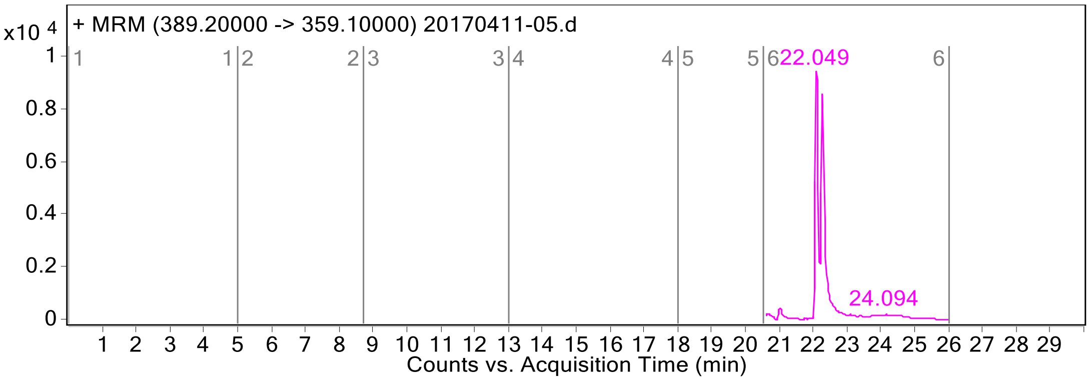
+ MRM (389.20000 -> 359.10000) 20170411-05.d
x10 4
22.049
24.094
Counts vs. Acquisition Time (min)

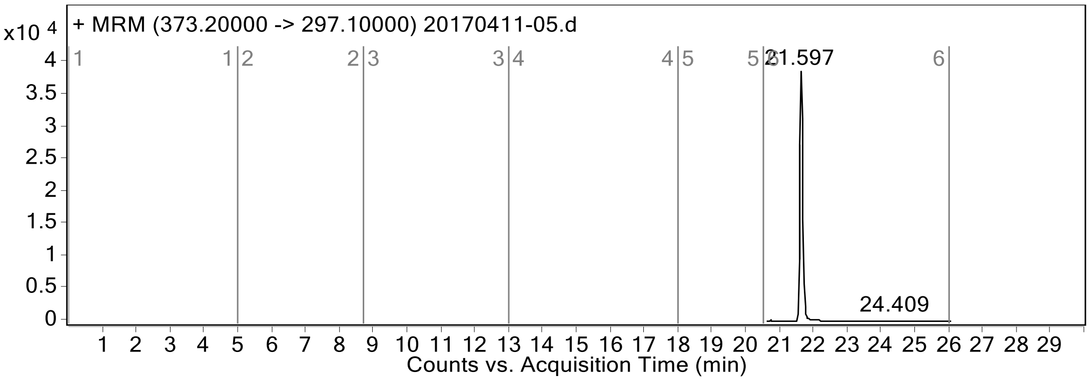
+ MRM (373.20000 -> 297.10000) 20170411-05.d
x10 4
21.597
24.409
Counts vs. Acquisition Time (min)

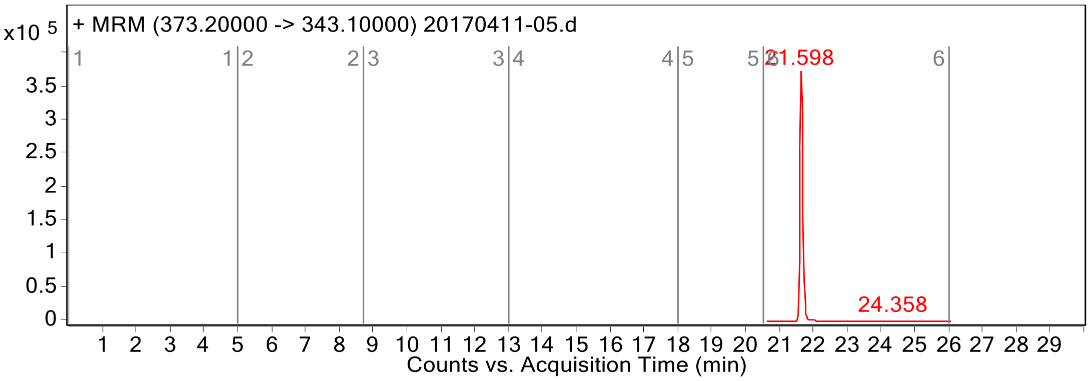
+ MRM (373.20000 -> 343.10000) 20170411-05.d
x10 5
21.598
24.358
Counts vs. Acquisition Time (min)

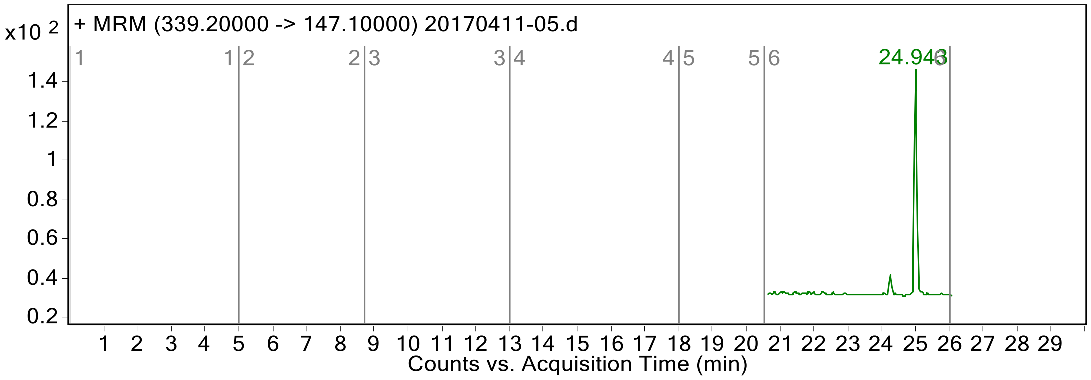
x10 2
+ MRM (339.20000 -> 147.10000) 20170411-05.d
24.943
Counts vs. Acquisition Time (min)

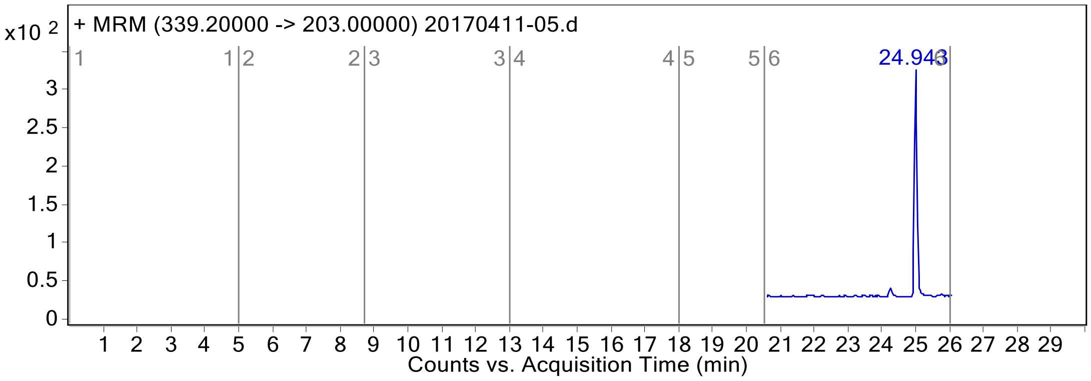
x10 2
+ MRM (339.20000 -> 203.00000) 20170411-05.d
24.943
Counts vs. Acquisition Time (min)

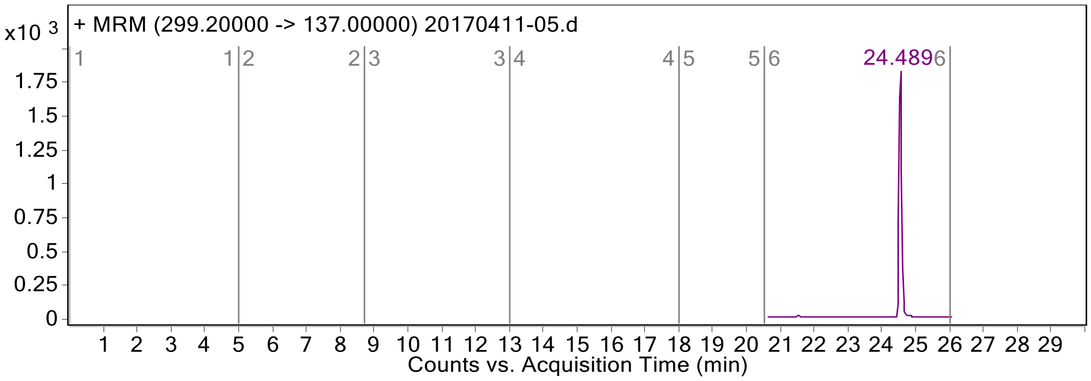
x10 3
+ MRM (299.20000 -> 137.00000) 20170411-05.d
24.489
Counts vs. Acquisition Time (min)

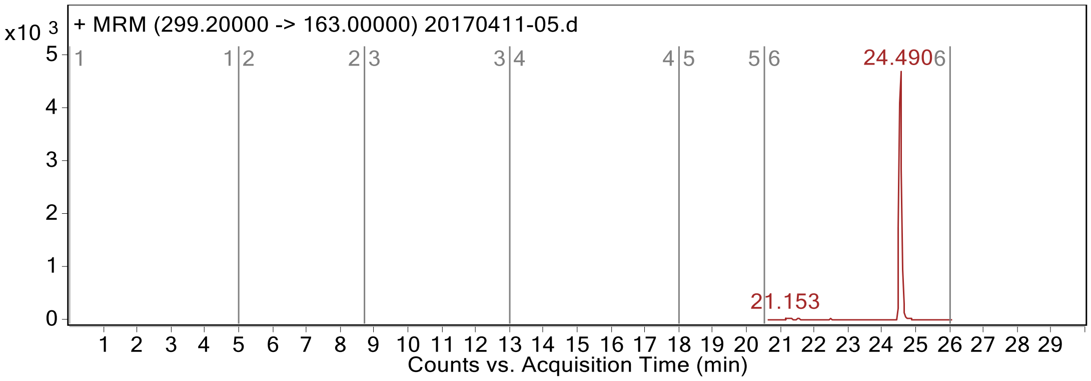
x10 3
+ MRM (299.20000 -> 163.00000) 20170411-05.d
24.490
21.153
Counts vs. Acquisition Time (min)

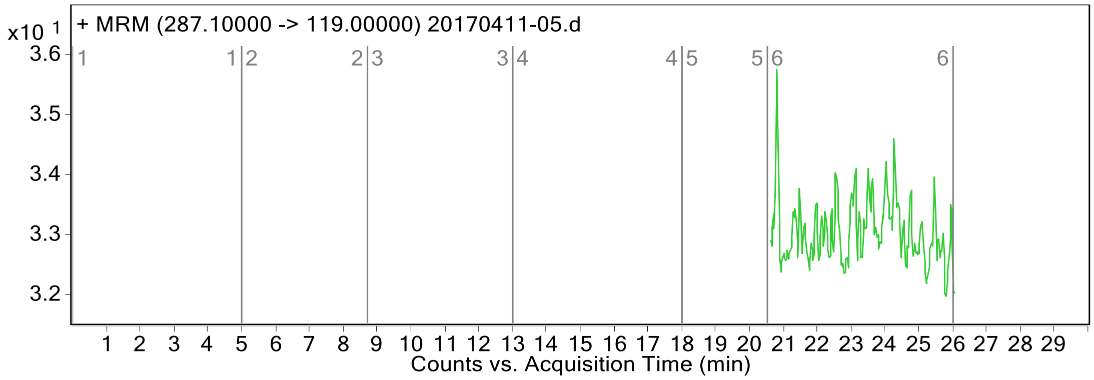
+ MRM (287.10000 -> 119.00000) 20170411-05.d
x10 1
3.6
3.5
3.4
3.3
3.2
1 2 3 4 5 6 7 8 9 10 11 12 13 14 15 16 17 18 19 20 21 22 23 24 25 26 27 28 29
Counts vs. Acquisition Time (min)

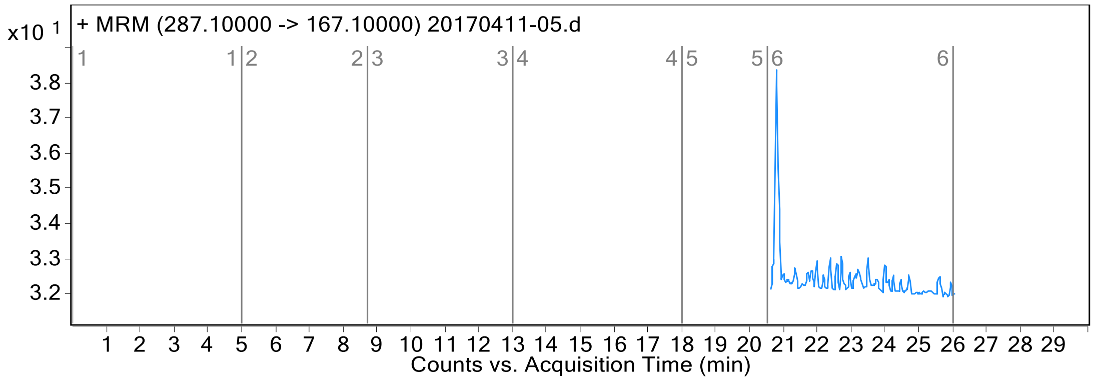
+ MRM (287.10000 -> 167.10000) 20170411-05.d
x10 1
3.8
3.7
3.6
3.5
3.4
3.3
3.2
1 2 3 4 5 6 7 8 9 10 11 12 13 14 15 16 17 18 19 20 21 22 23 24 25 26 27 28 29
Counts vs. Acquisition Time (min)

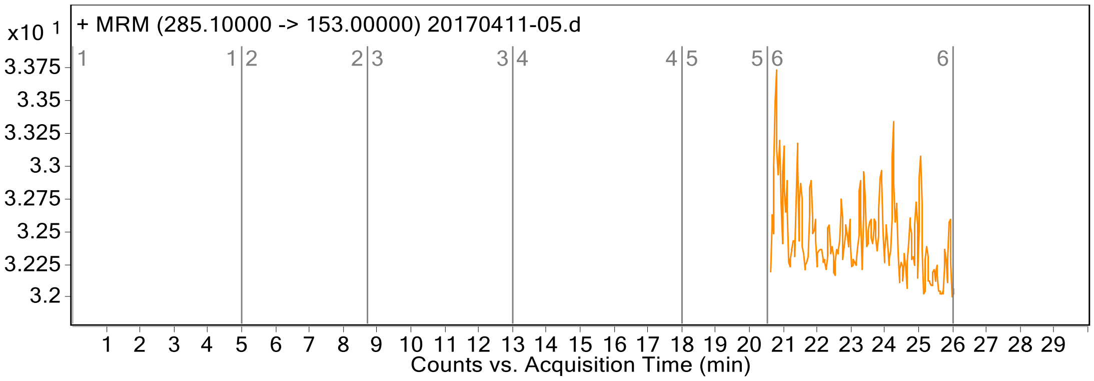
+ MRM (285.10000 -> 153.00000) 20170411-05.d
x10 1
3.375
3.35
3.325
3.3
3.275
3.25
3.225
3.2
1 2 3 4 5 6 7 8 9 10 11 12 13 14 15 16 17 18 19 20 21 22 23 24 25 26 27 28 29
Counts vs. Acquisition Time (min)

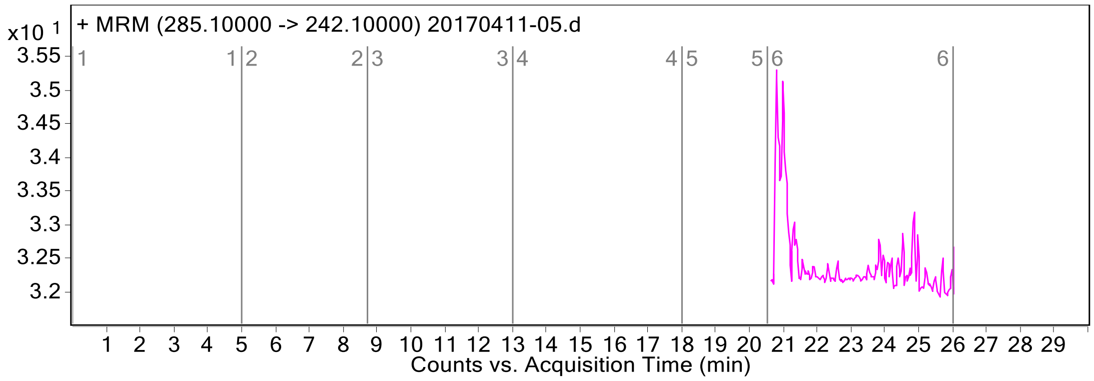
+ MRM (285.10000 -> 242.10000) 20170411-05.d
x10 1
3.55
3.5
3.45
3.4
3.35
3.3
3.25
3.2
1 2 3 4 5 6 7 8 9 10 11 12 13 14 15 16 17 18 19 20 21 22 23 24 25 26 27 28 29
Counts vs. Acquisition Time (min)

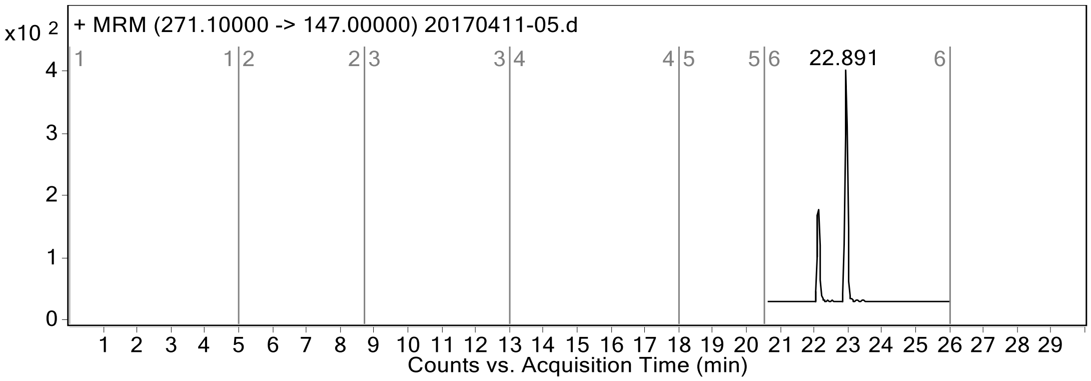

x10 2
+ MRM (271.10000 -> 147.00000) 20170411-05.d
22.891
Counts vs. Acquisition Time (min)

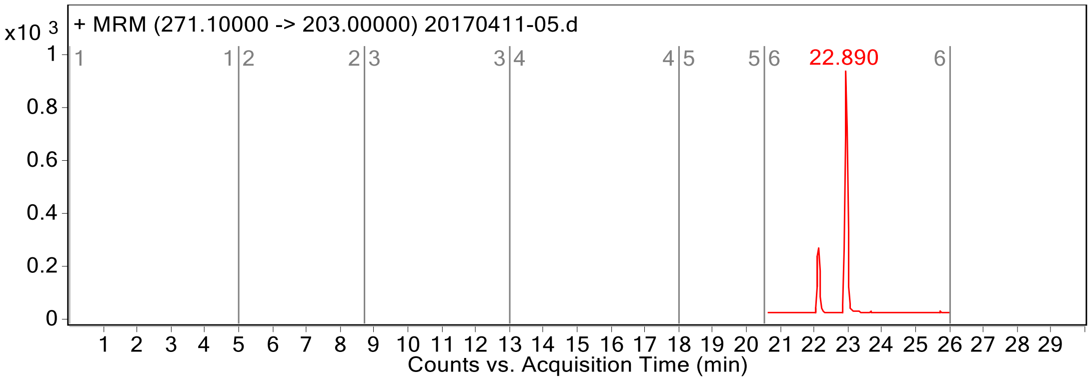

x10 3
+ MRM (271.10000 -> 203.00000) 20170411-05.d
22.890
Counts vs. Acquisition Time (min)

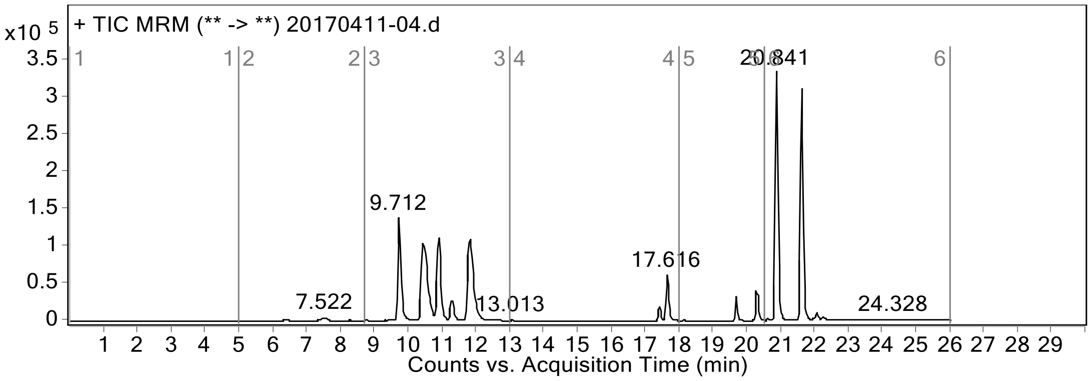

x10 5
+ TIC MRM (** -> **) 20170411-04.d
9.712
7.522
13.013
17.616
20.841
24.328
Counts vs. Acquisition Time (min)

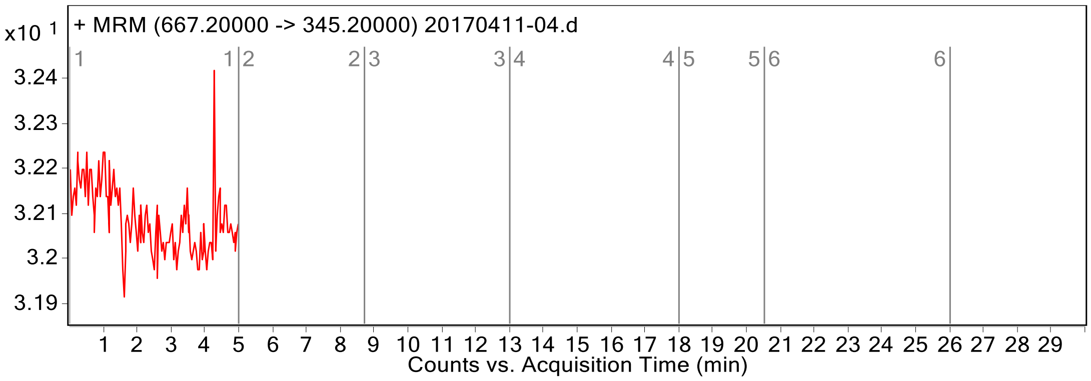

x10 1
+ MRM (667.20000 -> 345.20000) 20170411-04.d
Counts vs. Acquisition Time (min)

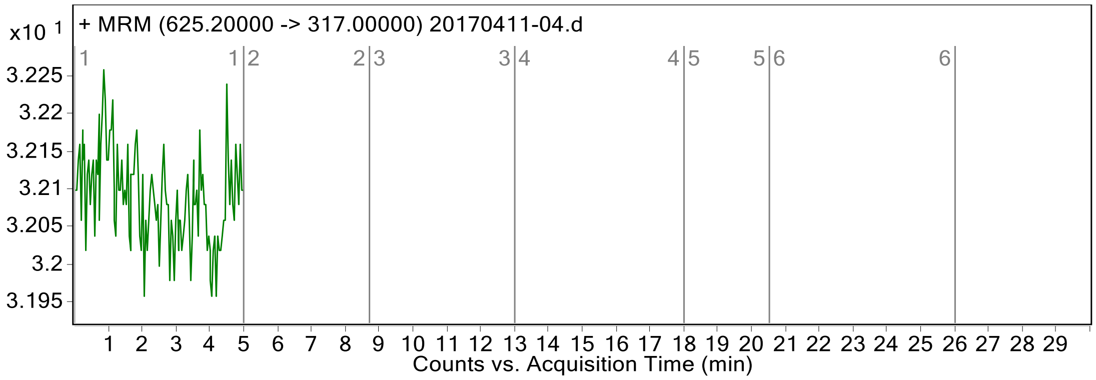
+ MRM (625.20000 -> 317.00000) 20170411-04.d
x10 1
3.225
3.22
3.215
3.21
3.205
3.2
3.195
1 | 1 2 | 2 3 | 3 4 | 4 5 | 5 6 | 6
1 2 3 4 5 6 7 8 9 10 11 12 13 14 15 16 17 18 19 20 21 22 23 24 25 26 27 28 29
Counts vs. Acquisition Time (min)

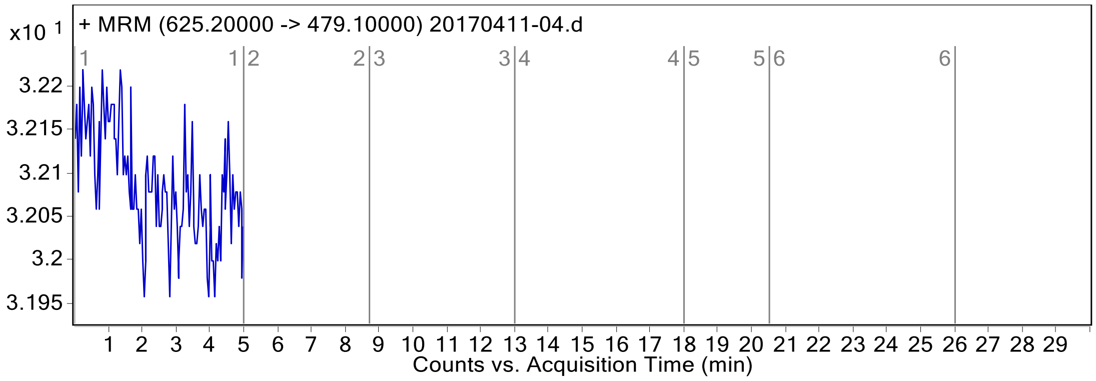
+ MRM (625.20000 -> 479.10000) 20170411-04.d
x10 1
3.22
3.215
3.21
3.205
3.2
3.195
1 | 1 2 | 2 3 | 3 4 | 4 5 | 5 6 | 6
1 2 3 4 5 6 7 8 9 10 11 12 13 14 15 16 17 18 19 20 21 22 23 24 25 26 27 28 29
Counts vs. Acquisition Time (min)

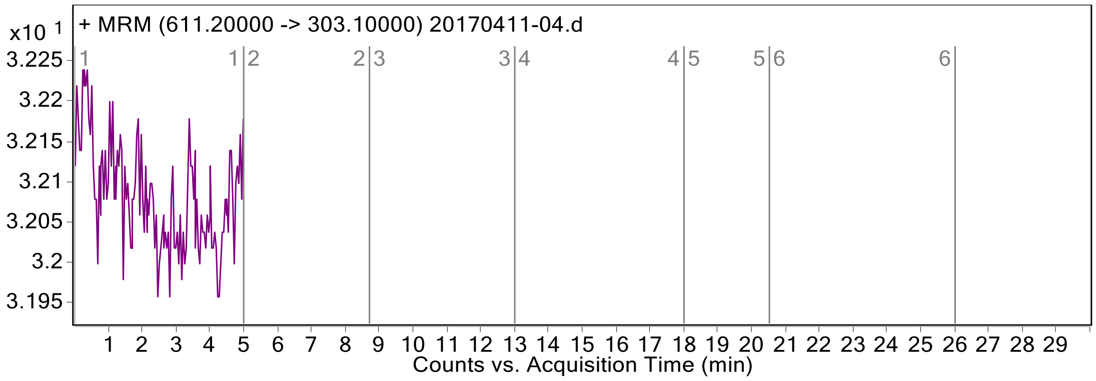
+ MRM (611.20000 -> 303.10000) 20170411-04.d
x10 1
3.225
3.22
3.215
3.21
3.205
3.2
3.195
1 | 1 2 | 2 3 | 3 4 | 4 5 | 5 6 | 6
1 2 3 4 5 6 7 8 9 10 11 12 13 14 15 16 17 18 19 20 21 22 23 24 25 26 27 28 29
Counts vs. Acquisition Time (min)

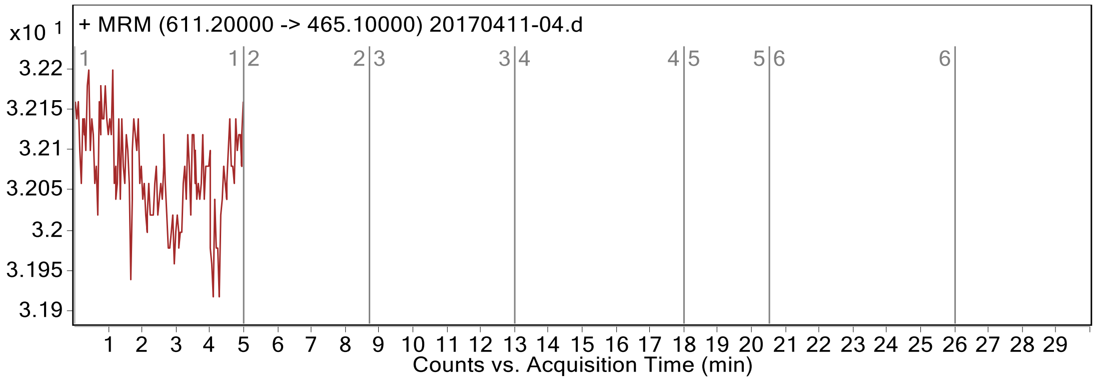
+ MRM (611.20000 -> 465.10000) 20170411-04.d
x10 1
3.22
3.215
3.21
3.205
3.2
3.195
3.19
1 | 1 2 | 2 3 | 3 4 | 4 5 | 5 6 | 6
1 2 3 4 5 6 7 8 9 10 11 12 13 14 15 16 17 18 19 20 21 22 23 24 25 26 27 28 29
Counts vs. Acquisition Time (min)

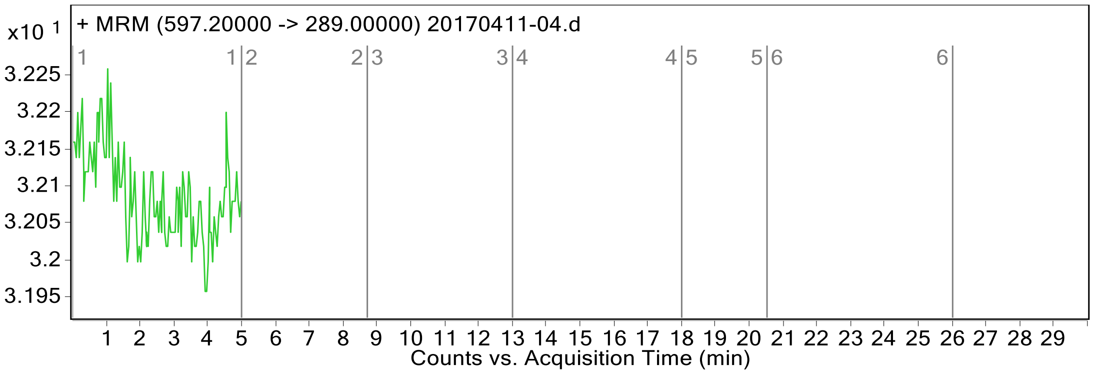
x10 1
+ MRM (597.20000 -> 289.00000) 20170411-04.d
3.225
3.22
3.215
3.21
3.205
3.2
3.195
1 2 3 4 5 6 7 8 9 10 11 12 13 14 15 16 17 18 19 20 21 22 23 24 25 26 27 28 29
Counts vs. Acquisition Time (min)

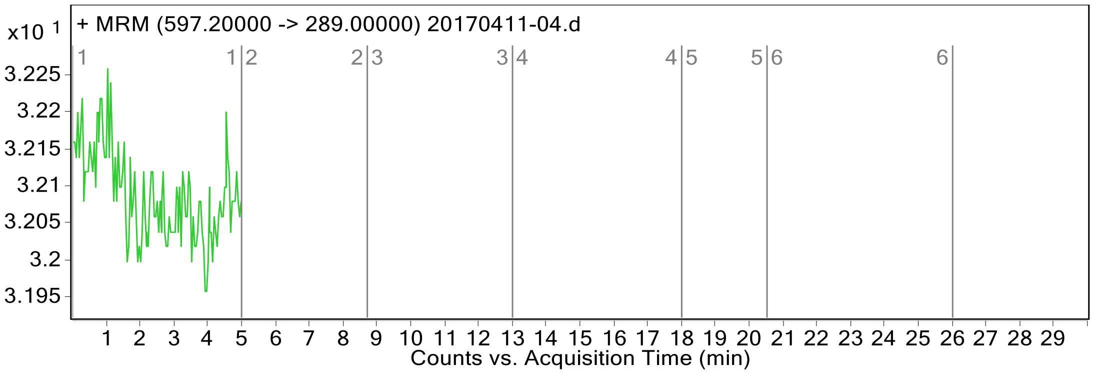
x10 1
+ MRM (597.20000 -> 289.00000) 20170411-04.d
3.225
3.22
3.215
3.21
3.205
3.2
3.195
1 2 3 4 5 6 7 8 9 10 11 12 13 14 15 16 17 18 19 20 21 22 23 24 25 26 27 28 29
Counts vs. Acquisition Time (min)

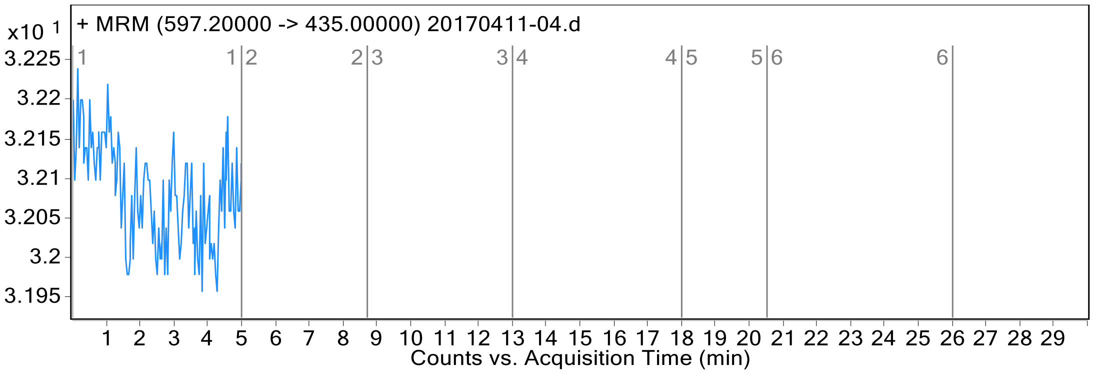
x10 1
+ MRM (597.20000 -> 435.00000) 20170411-04.d
3.225
3.22
3.215
3.21
3.205
3.2
3.195
1 2 3 4 5 6 7 8 9 10 11 12 13 14 15 16 17 18 19 20 21 22 23 24 25 26 27 28 29
Counts vs. Acquisition Time (min)

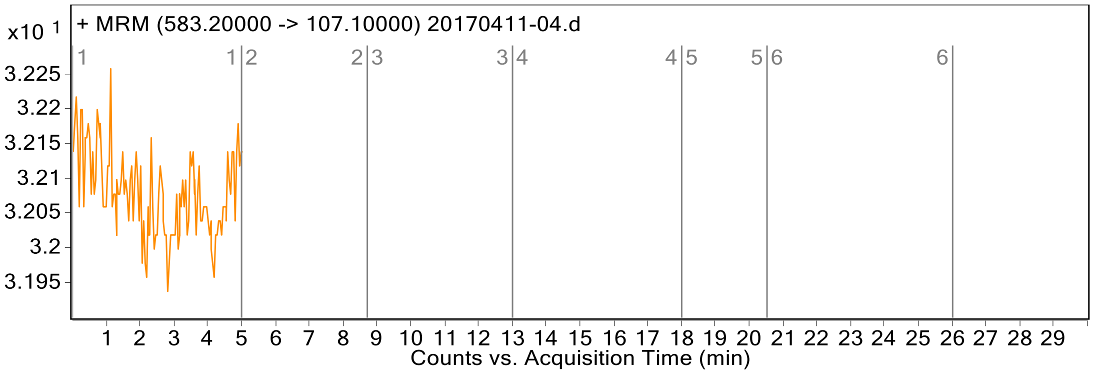
x10 1
+ MRM (583.20000 -> 107.10000) 20170411-04.d
3.225
3.22
3.215
3.21
3.205
3.2
3.195
1 2 3 4 5 6 7 8 9 10 11 12 13 14 15 16 17 18 19 20 21 22 23 24 25 26 27 28 29
Counts vs. Acquisition Time (min)

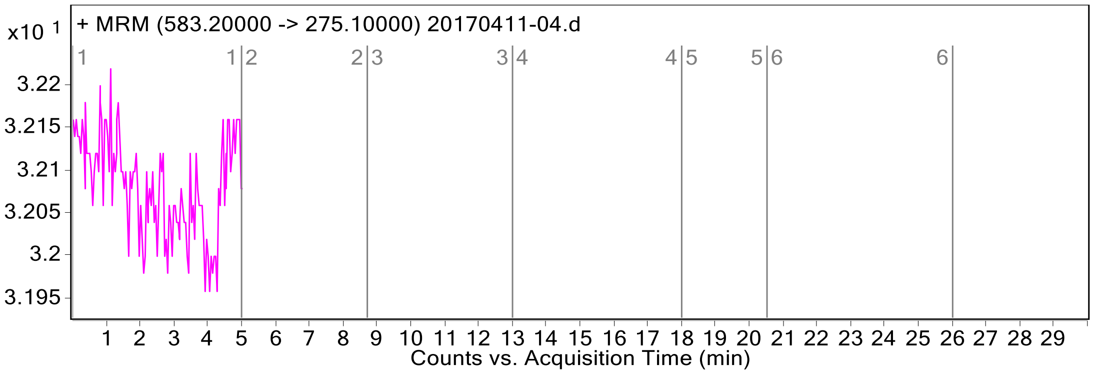
x10 1
+ MRM (583.20000 -> 275.10000) 20170411-04.d
3.22
3.215
3.21
3.205
3.2
3.195
1 2 3 4 5 6 7 8 9 10 11 12 13 14 15 16 17 18 19 20 21 22 23 24 25 26 27 28 29
Counts vs. Acquisition Time (min)

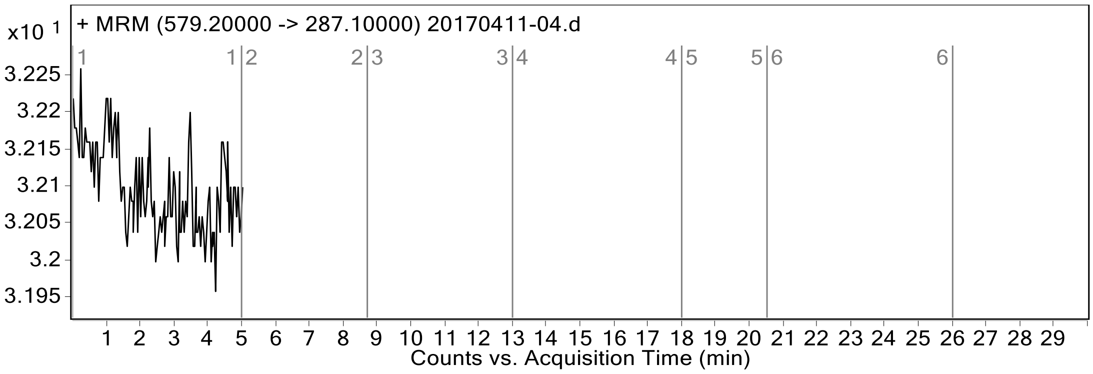
x10 1
+ MRM (579.20000 -> 287.10000) 20170411-04.d
3.225
3.22
3.215
3.21
3.205
3.2
3.195
1 2 3 4 5 6 7 8 9 10 11 12 13 14 15 16 17 18 19 20 21 22 23 24 25 26 27 28 29
Counts vs. Acquisition Time (min)

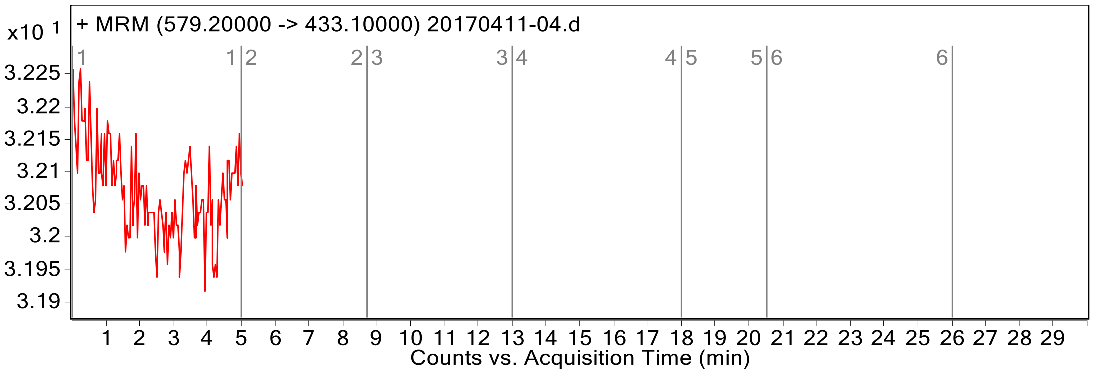
x10 1
+ MRM (579.20000 -> 433.10000) 20170411-04.d
3.225
3.22
3.215
3.21
3.205
3.2
3.195
3.19
1 2 3 4 5 6 7 8 9 10 11 12 13 14 15 16 17 18 19 20 21 22 23 24 25 26 27 28 29
Counts vs. Acquisition Time (min)

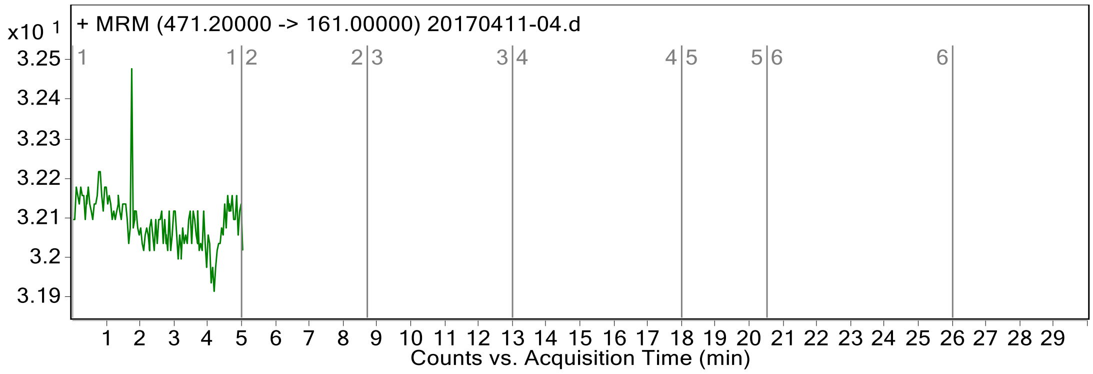
x10 1
+ MRM (471.20000 -> 161.00000) 20170411-04.d
3.25
3.24
3.23
3.22
3.21
3.2
3.19
1 2 3 4 5 6 7 8 9 10 11 12 13 14 15 16 17 18 19 20 21 22 23 24 25 26 27 28 29
Counts vs. Acquisition Time (min)

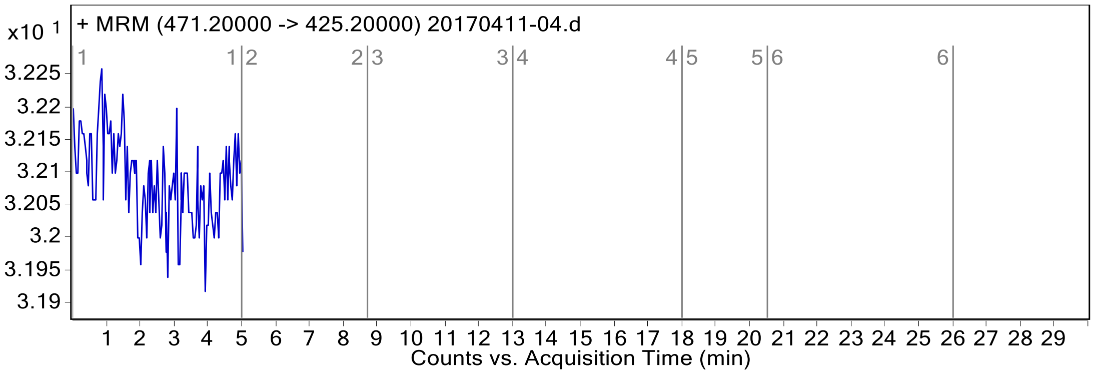
x10 1
+ MRM (471.20000 -> 425.20000) 20170411-04.d
3.225
3.22
3.215
3.21
3.205
3.2
3.195
3.19
1 2 3 4 5 6 7 8 9 10 11 12 13 14 15 16 17 18 19 20 21 22 23 24 25 26 27 28 29
Counts vs. Acquisition Time (min)

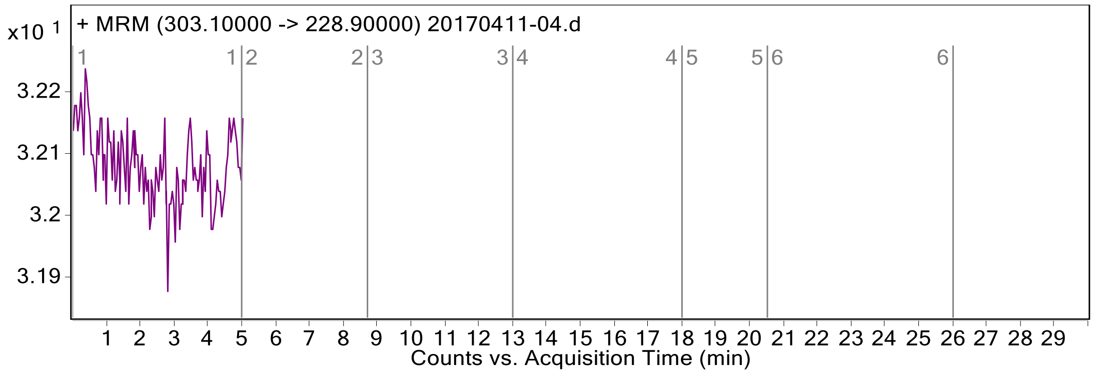
x10 1
+ MRM (303.10000 -> 228.90000) 20170411-04.d
3.22
3.21
3.2
3.19
1 2 3 4 5 6 7 8 9 10 11 12 13 14 15 16 17 18 19 20 21 22 23 24 25 26 27 28 29
Counts vs. Acquisition Time (min)

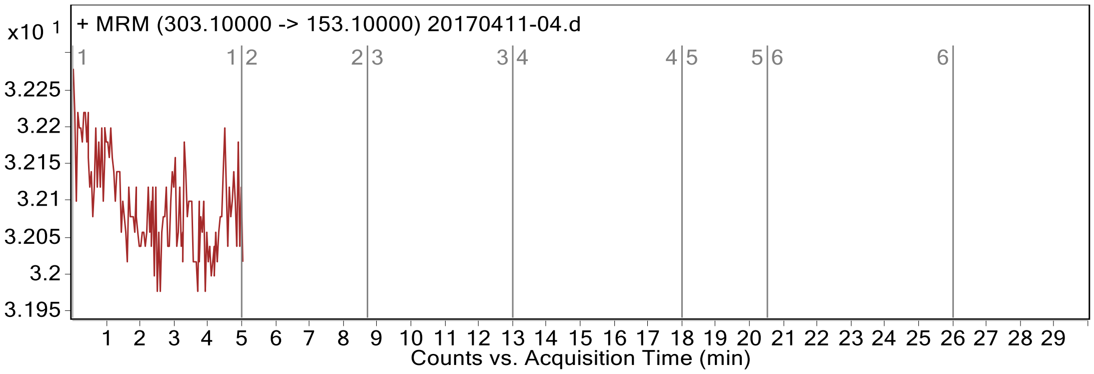
x10 1
+ MRM (303.10000 -> 153.10000) 20170411-04.d
3.225
3.22
3.215
3.21
3.205
3.2
3.195
1 2 3 4 5 6 7 8 9 10 11 12 13 14 15 16 17 18 19 20 21 22 23 24 25 26 27 28 29
Counts vs. Acquisition Time (min)

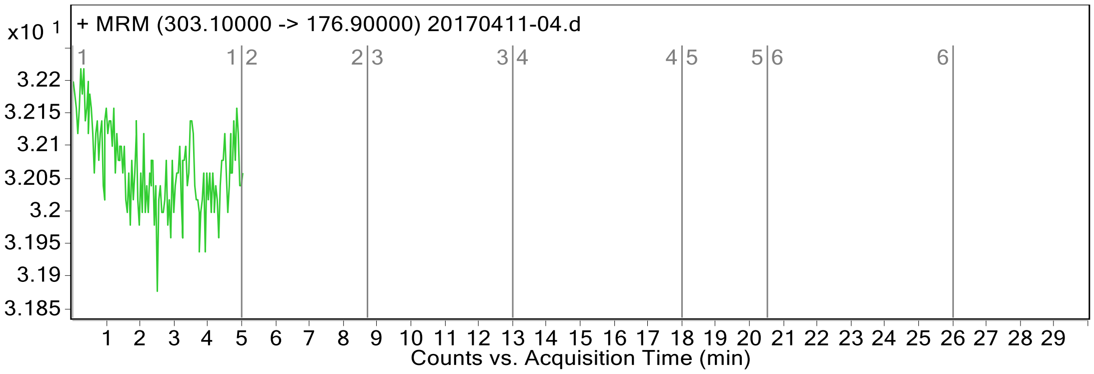
x10 1
+ MRM (303.10000 -> 176.90000) 20170411-04.d
3.22
3.215
3.21
3.205
3.2
3.195
3.19
3.185
1 2 3 4 5 6 7 8 9 10 11 12 13 14 15 16 17 18 19 20 21 22 23 24 25 26 27 28 29
Counts vs. Acquisition Time (min)

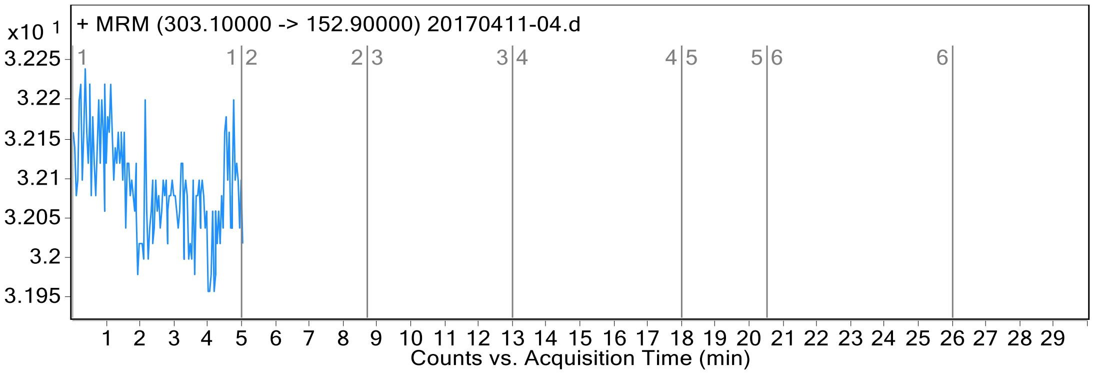

x10 1
+ MRM (303.10000 -> 152.90000) 20170411-04.d
3.225
3.22
3.215
3.21
3.205
3.2
3.195
1 2 3 4 5 6 7 8 9 10 11 12 13 14 15 16 17 18 19 20 21 22 23 24 25 26 27 28 29
Counts vs. Acquisition Time (min)

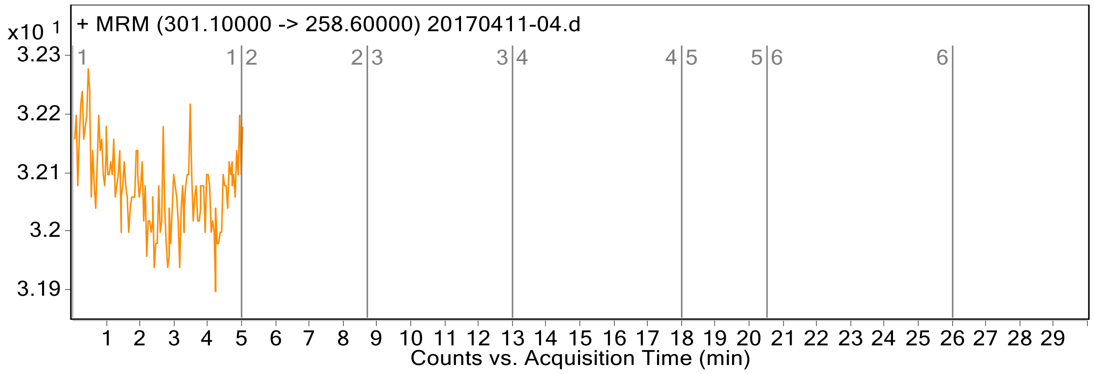

x10 1
+ MRM (301.10000 -> 258.60000) 20170411-04.d
3.23
3.22
3.21
3.2
3.19
1 2 3 4 5 6 7 8 9 10 11 12 13 14 15 16 17 18 19 20 21 22 23 24 25 26 27 28 29
Counts vs. Acquisition Time (min)

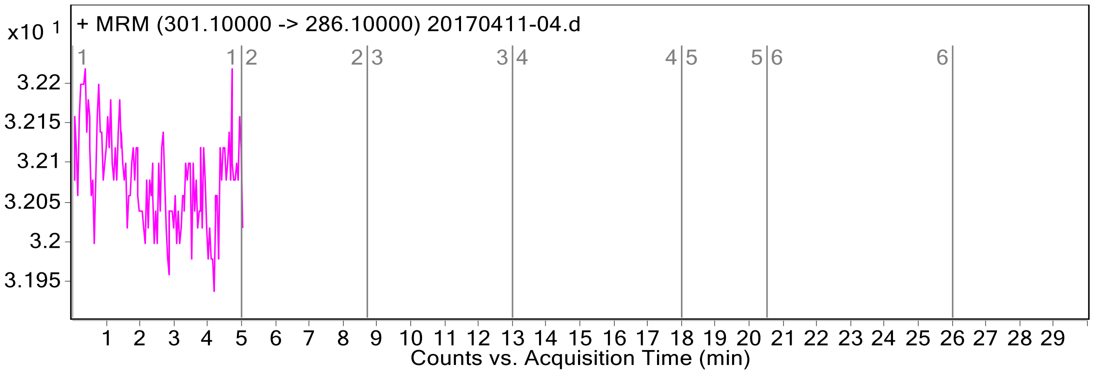

x10 1
+ MRM (301.10000 -> 286.10000) 20170411-04.d
3.22
3.215
3.21
3.205
3.2
3.195
1 2 3 4 5 6 7 8 9 10 11 12 13 14 15 16 17 18 19 20 21 22 23 24 25 26 27 28 29
Counts vs. Acquisition Time (min)

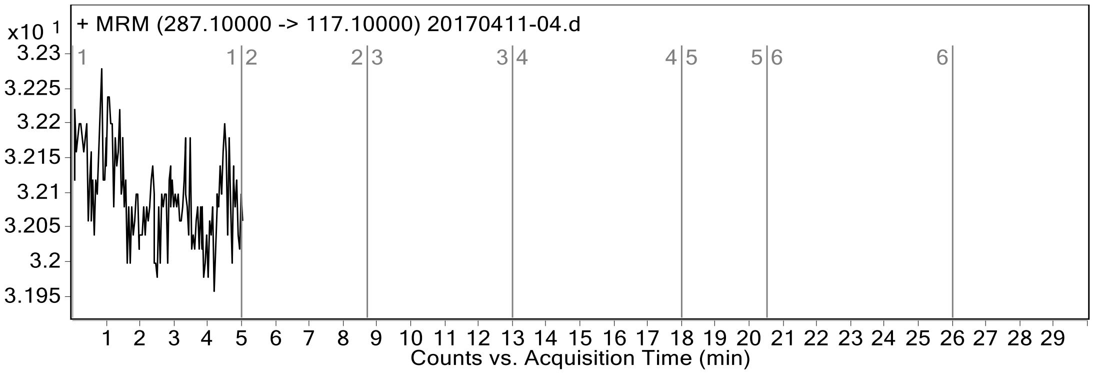

x10 1
+ MRM (287.10000 -> 117.10000) 20170411-04.d
3.23
3.225
3.22
3.215
3.21
3.205
3.2
3.195
1 2 3 4 5 6 7 8 9 10 11 12 13 14 15 16 17 18 19 20 21 22 23 24 25 26 27 28 29
Counts vs. Acquisition Time (min)

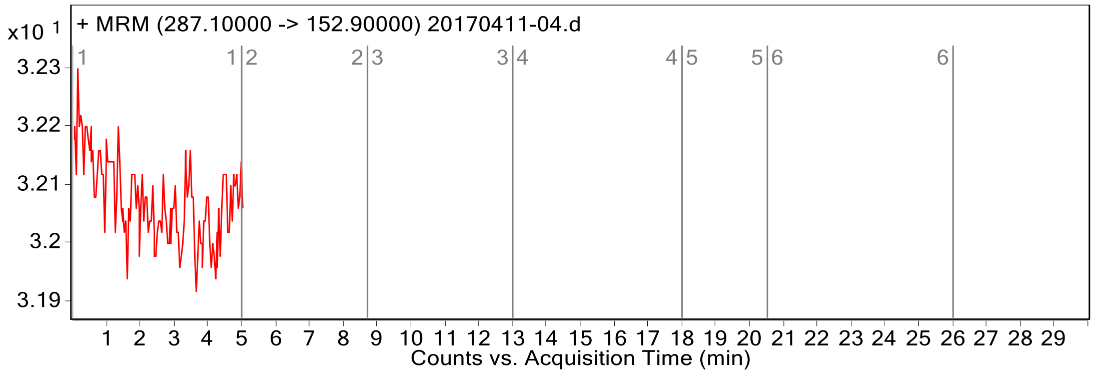

x10 1
+ MRM (287.10000 -> 152.90000) 20170411-04.d
3.23
3.22
3.21
3.2
3.19
Counts vs. Acquisition Time (min)

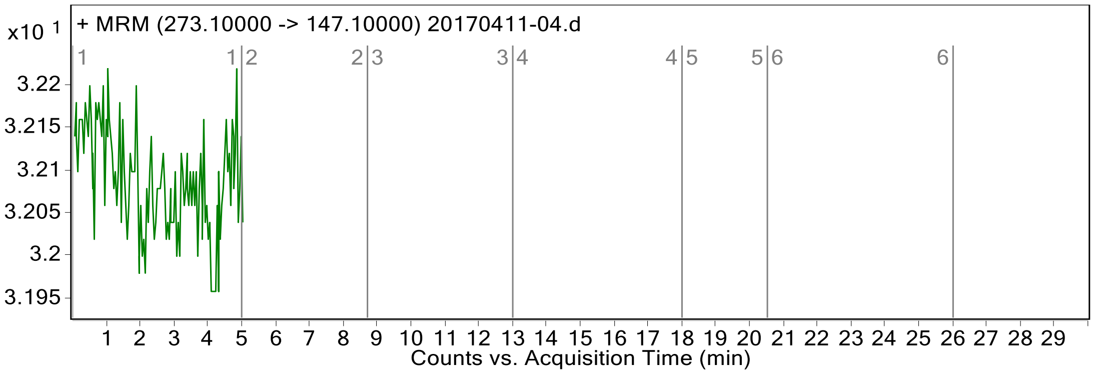

x10 1
+ MRM (273.10000 -> 147.10000) 20170411-04.d
3.22
3.215
3.21
3.205
3.2
3.195
Counts vs. Acquisition Time (min)

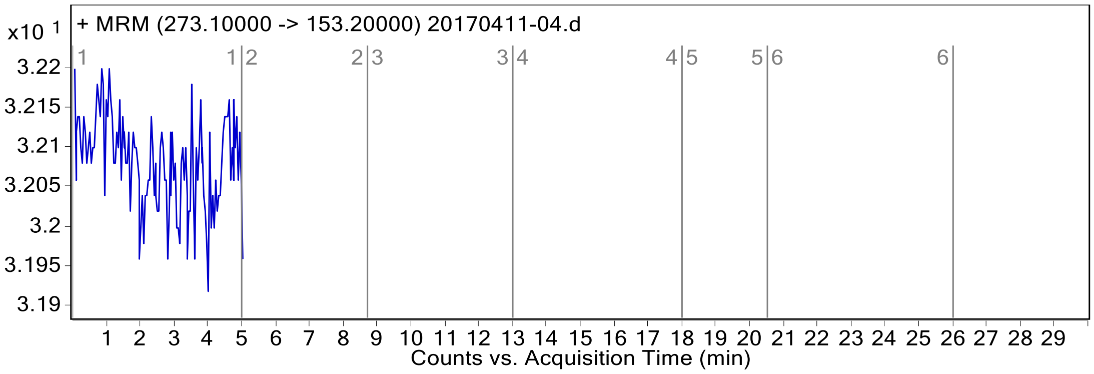

x10 1
+ MRM (273.10000 -> 153.20000) 20170411-04.d
3.22
3.215
3.21
3.205
3.2
3.195
3.19
Counts vs. Acquisition Time (min)

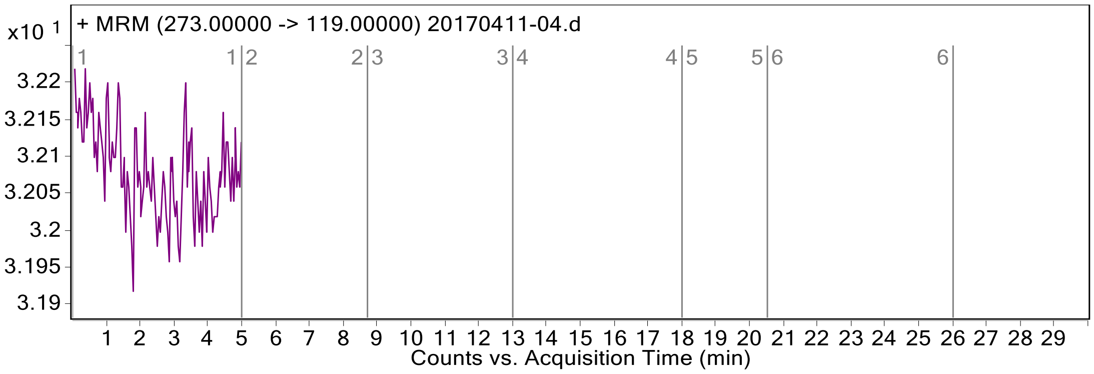

x10 1
+ MRM (273.00000 -> 119.00000) 20170411-04.d
3.22
3.215
3.21
3.205
3.2
3.195
3.19
Counts vs. Acquisition Time (min)

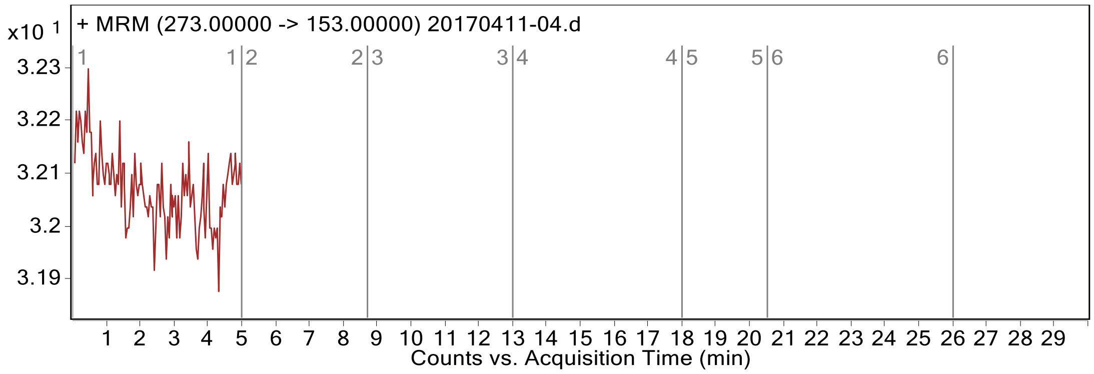
x10 1
+ MRM (273.00000 -> 153.00000) 20170411-04.d
3.23
3.22
3.21
3.2
3.19
Counts vs. Acquisition Time (min)

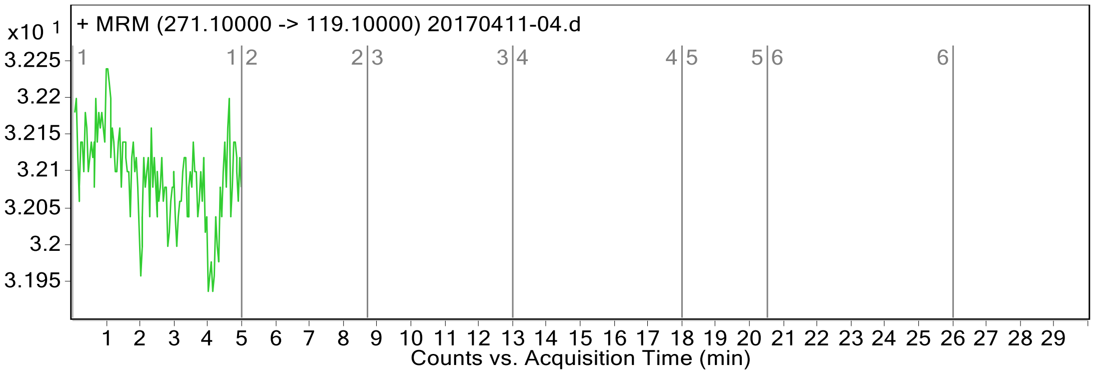
x10 1
+ MRM (271.10000 -> 119.10000) 20170411-04.d
3.225
3.22
3.215
3.21
3.205
3.2
3.195
Counts vs. Acquisition Time (min)

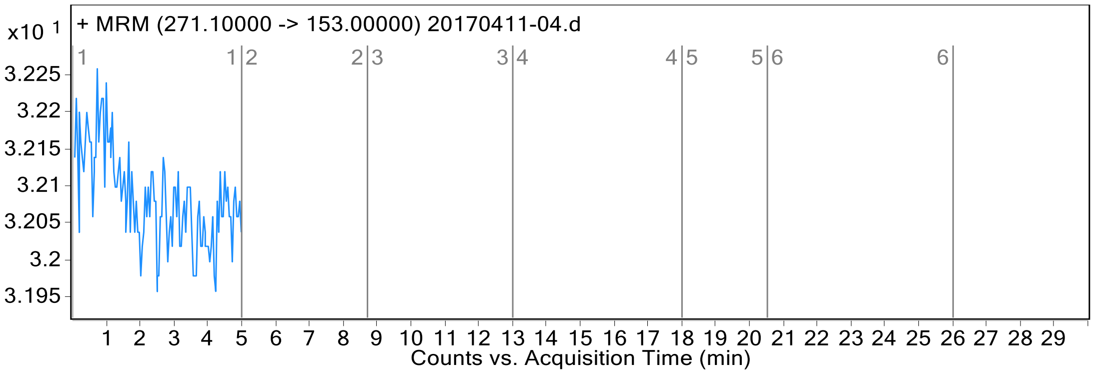
x10 1
+ MRM (271.10000 -> 153.00000) 20170411-04.d
3.225
3.22
3.215
3.21
3.205
3.2
3.195
Counts vs. Acquisition Time (min)

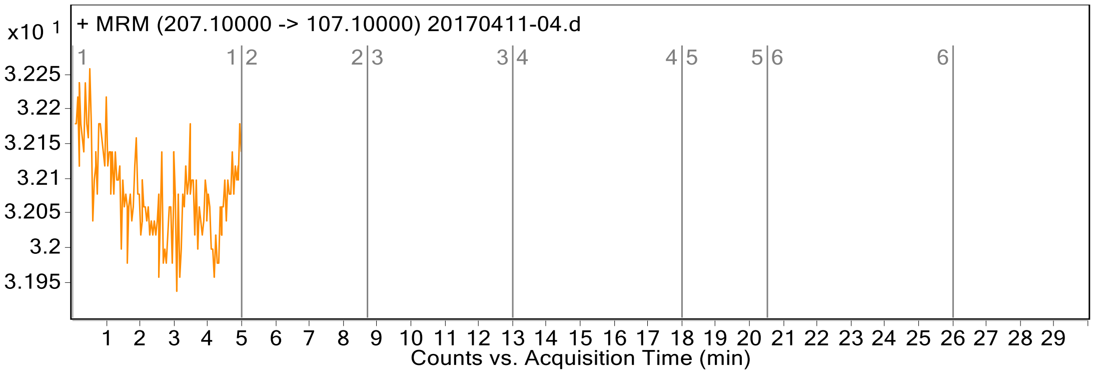
x10 1
+ MRM (207.10000 -> 107.10000) 20170411-04.d
3.225
3.22
3.215
3.21
3.205
3.2
3.195
Counts vs. Acquisition Time (min)

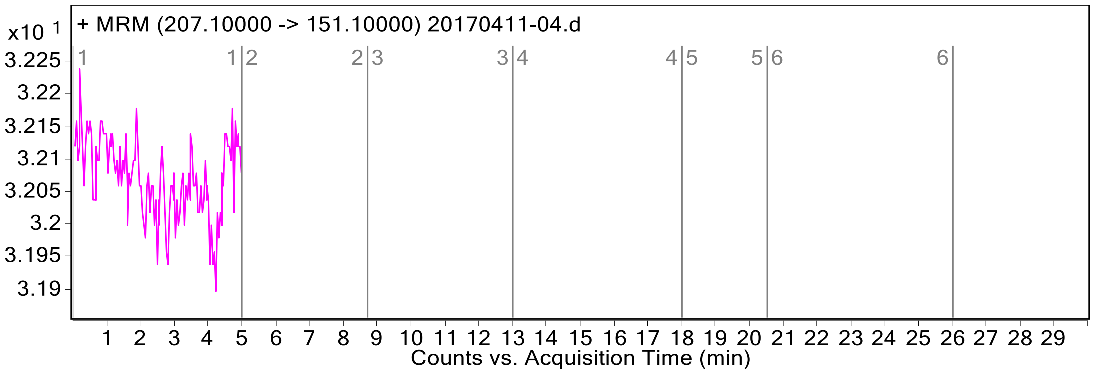
x10 1
+ MRM (207.10000 -> 151.10000) 20170411-04.d
Counts vs. Acquisition Time (min)

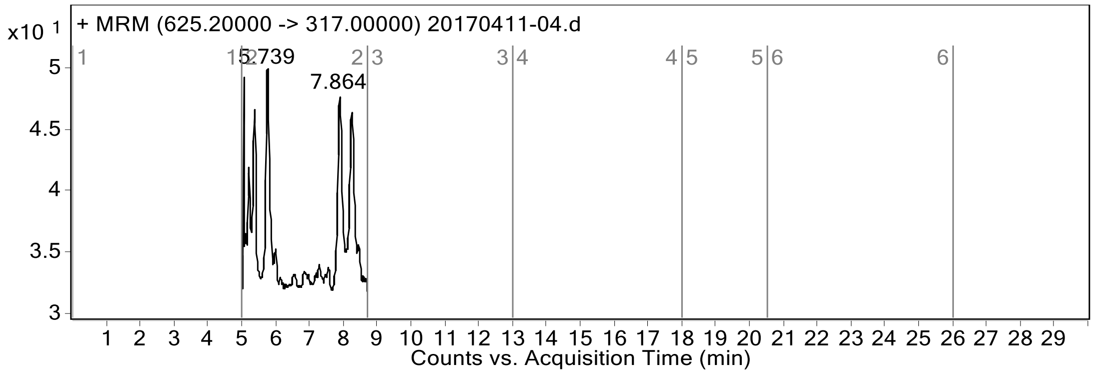
x10 1
+ MRM (625.20000 -> 317.00000) 20170411-04.d
5.739
7.864
Counts vs. Acquisition Time (min)

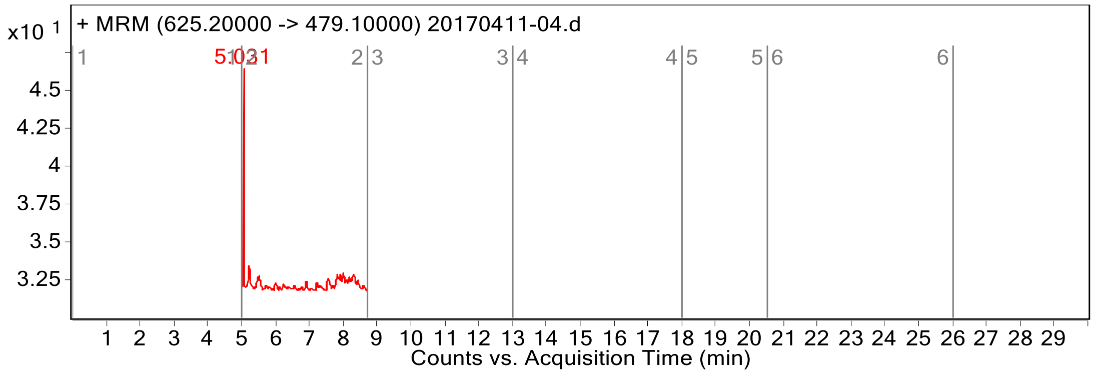
x10 1
+ MRM (625.20000 -> 479.10000) 20170411-04.d
5.031
Counts vs. Acquisition Time (min)

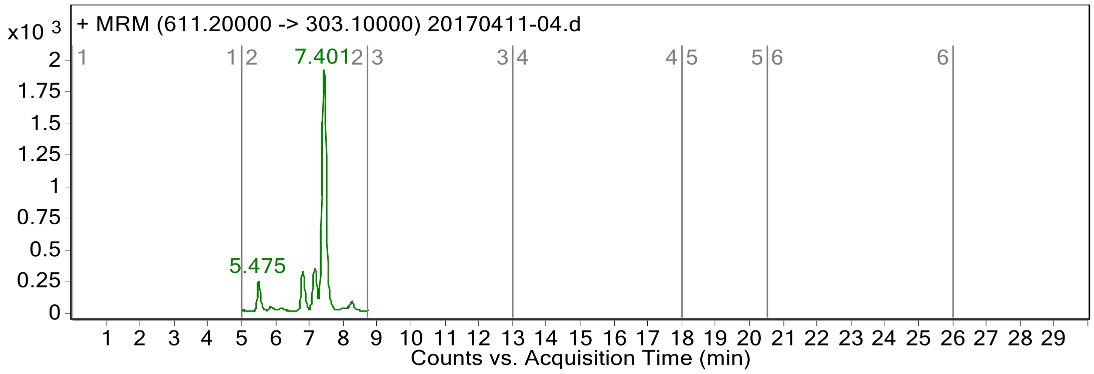
x10 3
+ MRM (611.20000 -> 303.10000) 20170411-04.d
7.401
5.475
Counts vs. Acquisition Time (min)

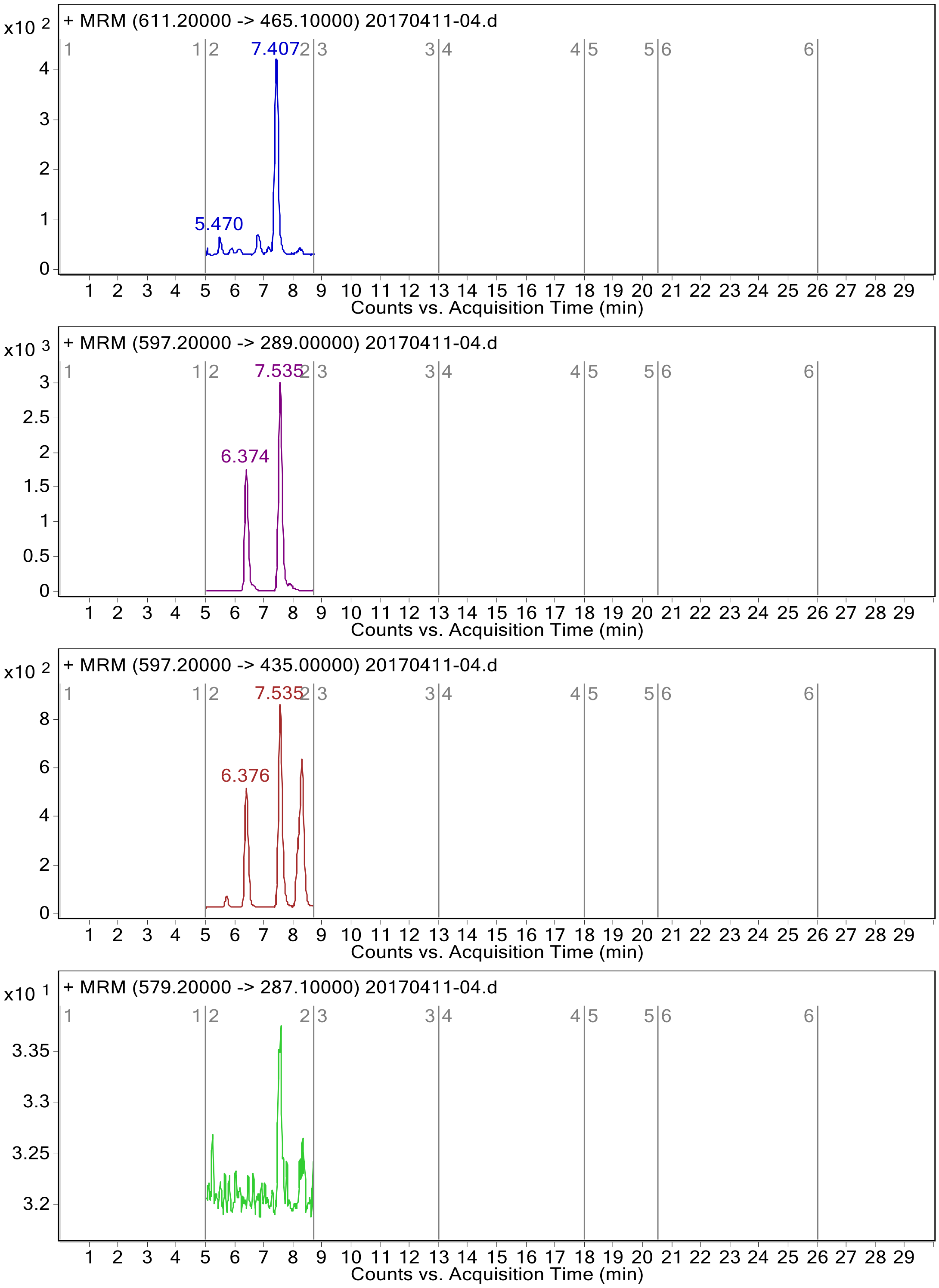
x10 2
+ MRM (611.20000 -> 465.10000) 20170411-04.d
7.407
5.470
Counts vs. Acquisition Time (min)
x10 3
+ MRM (597.20000 -> 289.00000) 20170411-04.d
7.535
6.374
Counts vs. Acquisition Time (min)
x10 2
+ MRM (597.20000 -> 435.00000) 20170411-04.d
7.535
6.376
Counts vs. Acquisition Time (min)
x10 1
+ MRM (579.20000 -> 287.10000) 20170411-04.d
Counts vs. Acquisition Time (min)

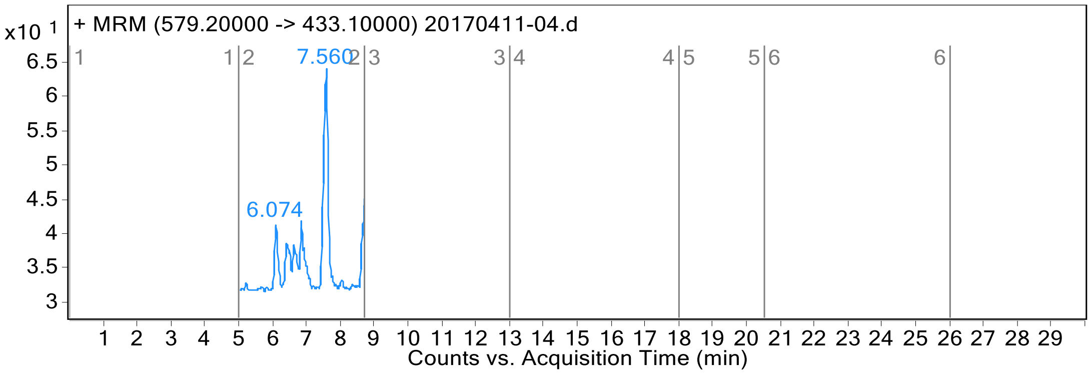
x10 1
+ MRM (579.20000 -> 433.10000) 20170411-04.d
7.560
6.074
Counts vs. Acquisition Time (min)

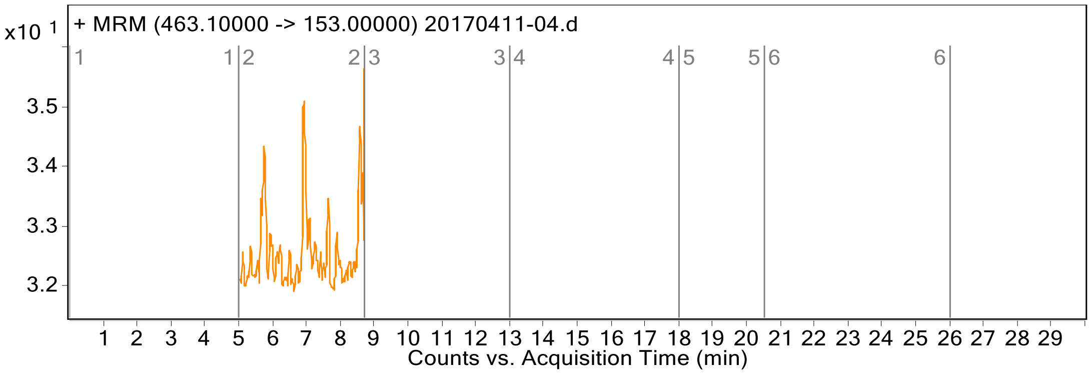
x10 1
+ MRM (463.10000 -> 153.00000) 20170411-04.d
Counts vs. Acquisition Time (min)

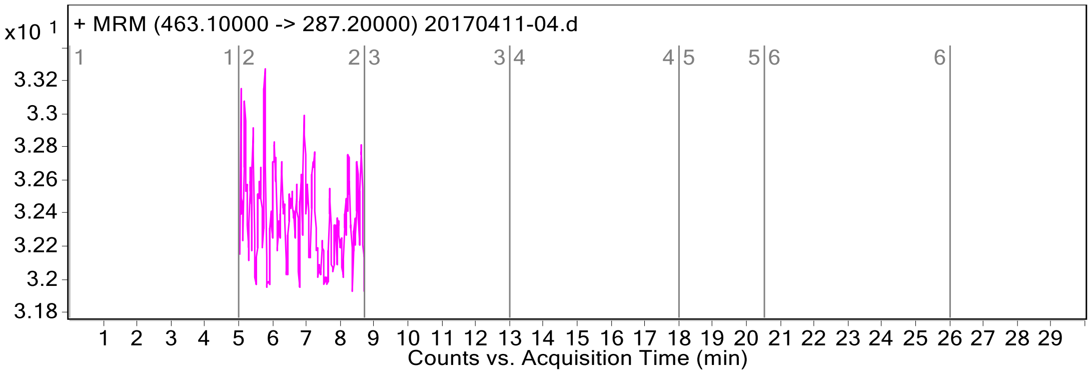
x10 1
+ MRM (463.10000 -> 287.20000) 20170411-04.d
Counts vs. Acquisition Time (min)

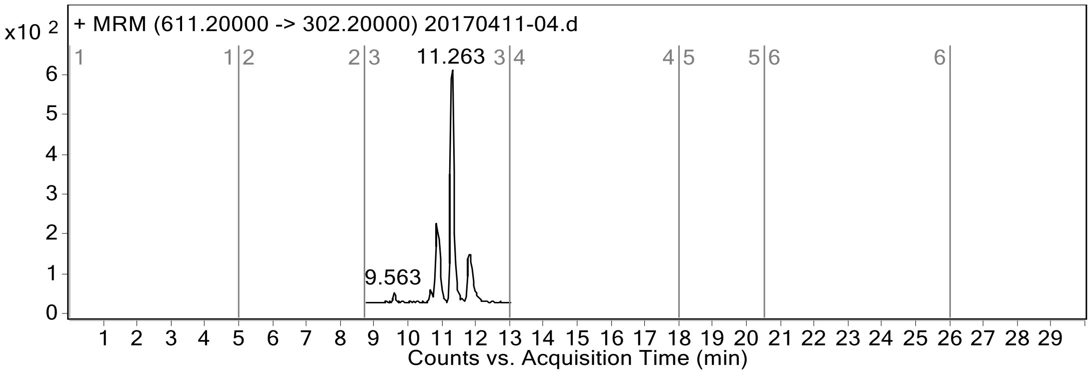
x10 2
+ MRM (611.20000 -> 302.20000) 20170411-04.d
11.263
9.563
Counts vs. Acquisition Time (min)

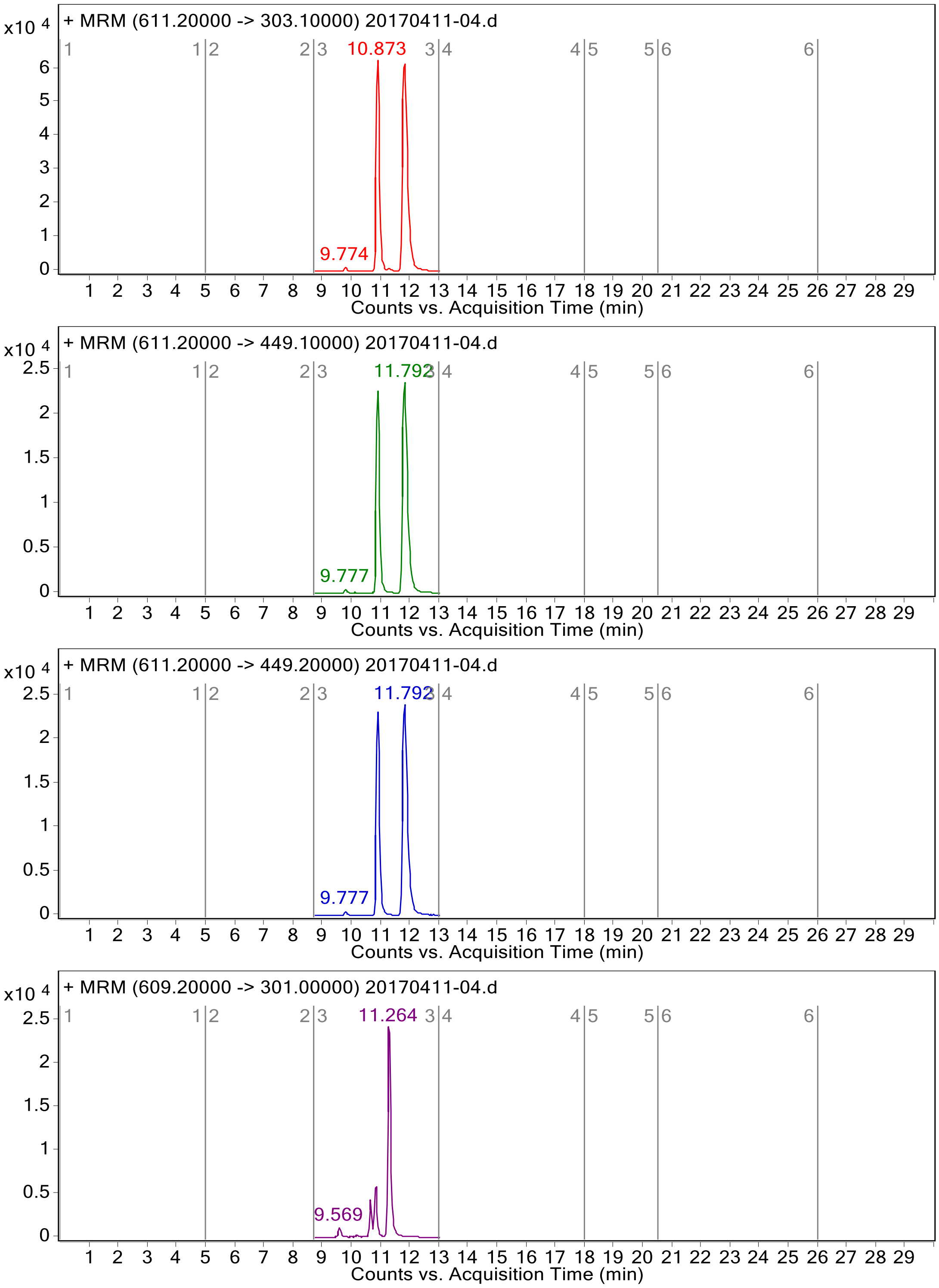

+ MRM (611.20000 -> 303.10000) 20170411-04.d
10.873
9.774
Counts vs. Acquisition Time (min)
+ MRM (611.20000 -> 449.10000) 20170411-04.d
11.792
9.777
Counts vs. Acquisition Time (min)
+ MRM (611.20000 -> 449.20000) 20170411-04.d
11.792
9.777
Counts vs. Acquisition Time (min)
+ MRM (609.20000 -> 301.00000) 20170411-04.d
11.264
9.569
Counts vs. Acquisition Time (min)

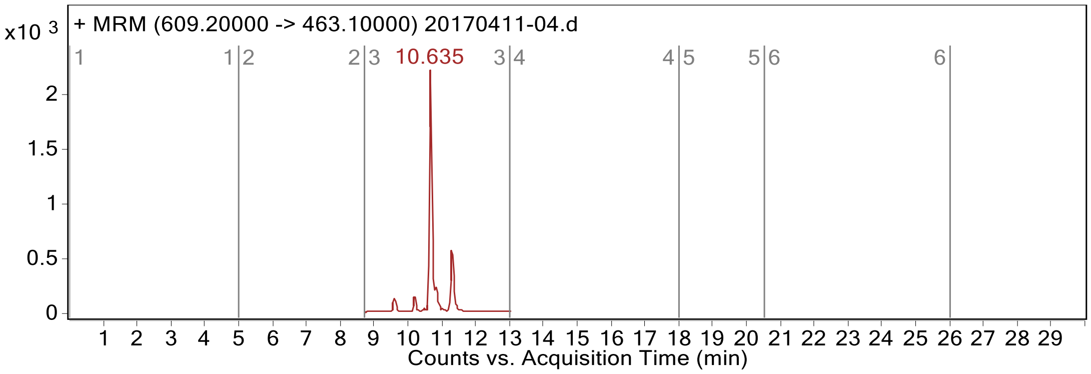
x10 3
+ MRM (609.20000 -> 463.10000) 20170411-04.d
10.635
Counts vs. Acquisition Time (min)

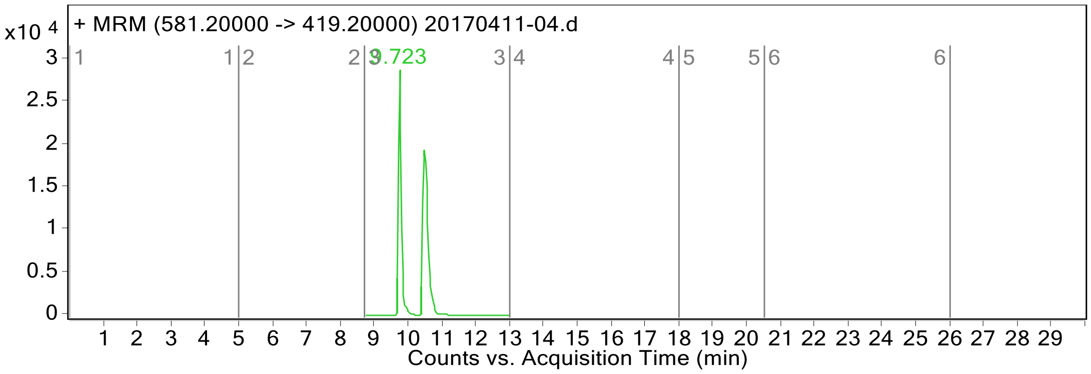
x10 4
+ MRM (581.20000 -> 419.20000) 20170411-04.d
9.723
Counts vs. Acquisition Time (min)

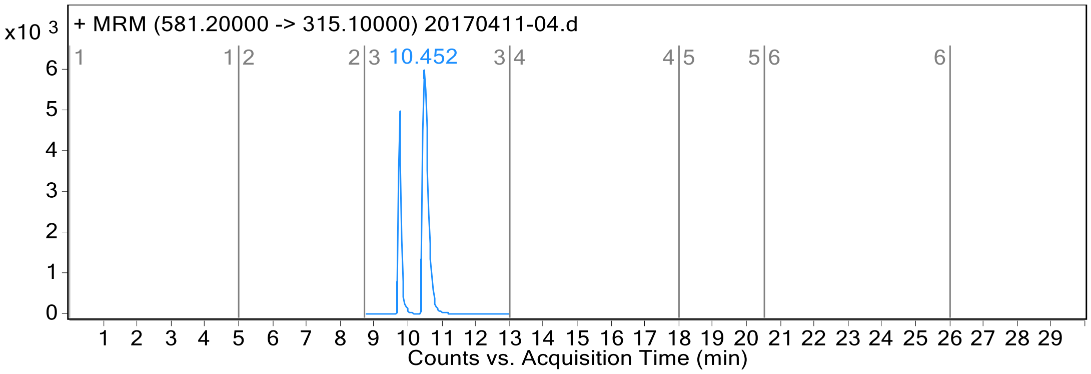
x10 3
+ MRM (581.20000 -> 315.10000) 20170411-04.d
10.452
Counts vs. Acquisition Time (min)

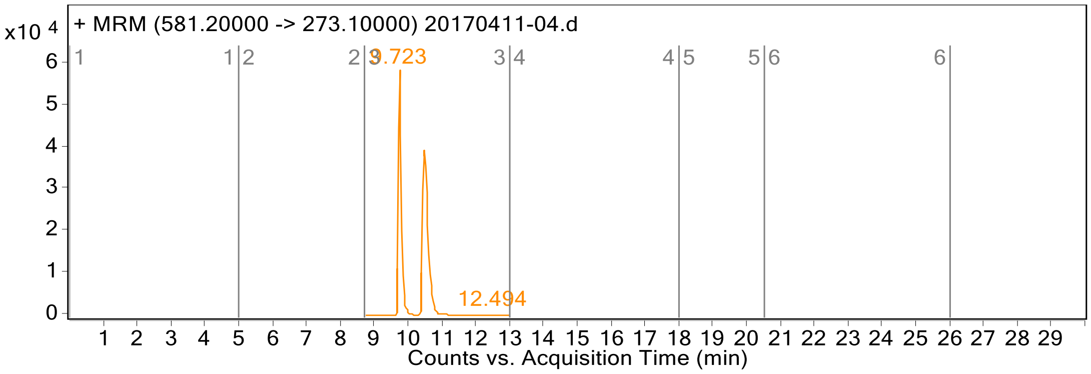
x10 4
+ MRM (581.20000 -> 273.10000) 20170411-04.d
9.723
12.494
Counts vs. Acquisition Time (min)

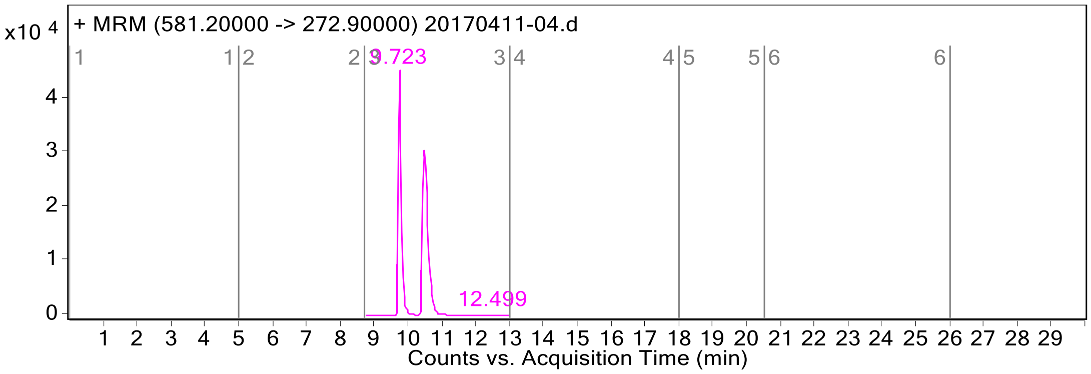
x10 4
+ MRM (581.20000 -> 272.90000) 20170411-04.d
9.723
12.499
Counts vs. Acquisition Time (min)

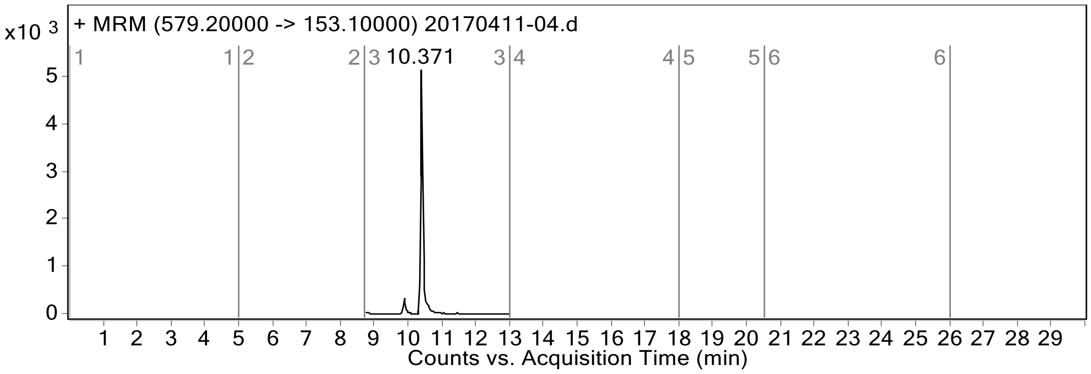
x10 3
+ MRM (579.20000 -> 153.10000) 20170411-04.d
10.371
Counts vs. Acquisition Time (min)

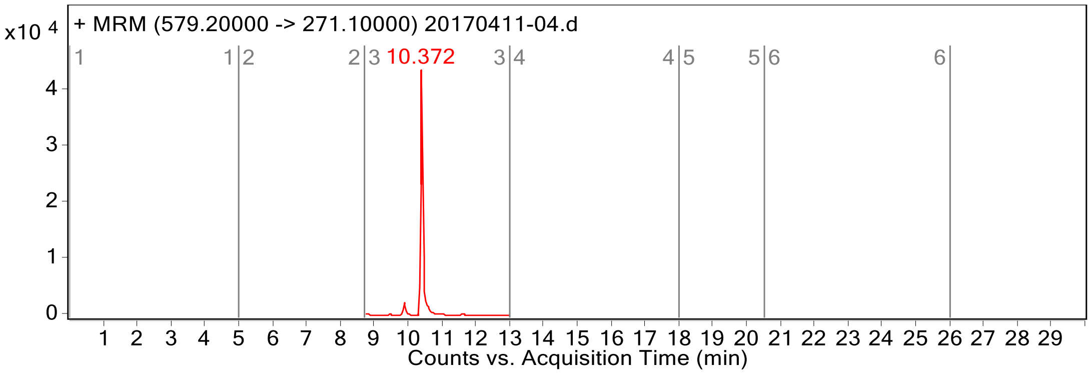
x10 4
+ MRM (579.20000 -> 271.10000) 20170411-04.d
10.372
Counts vs. Acquisition Time (min)

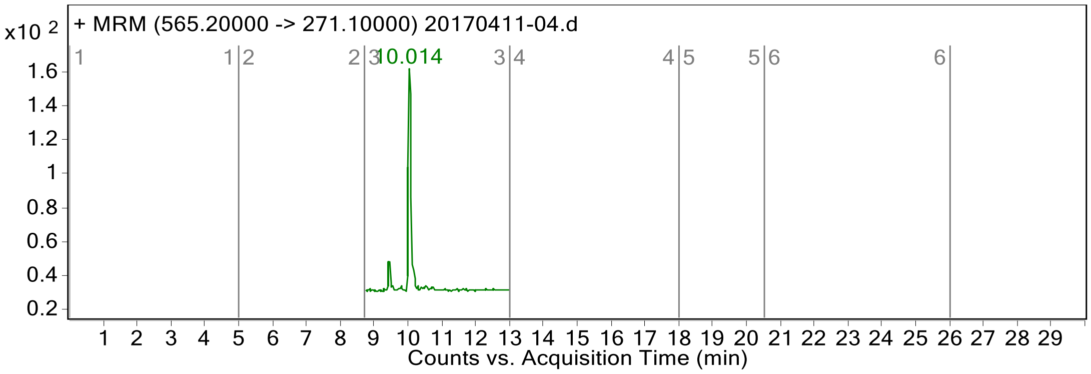
x10 2
+ MRM (565.20000 -> 271.10000) 20170411-04.d
10.014
Counts vs. Acquisition Time (min)

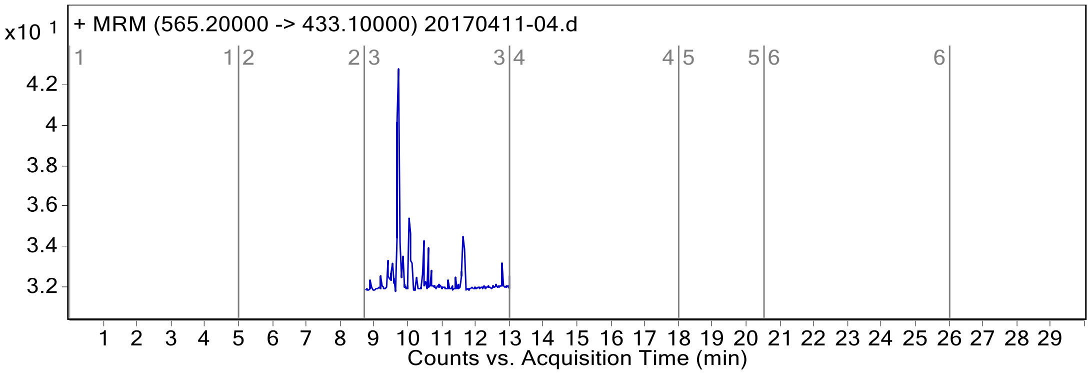

x10 1
+ MRM (565.20000 -> 433.10000) 20170411-04.d
Counts vs. Acquisition Time (min)

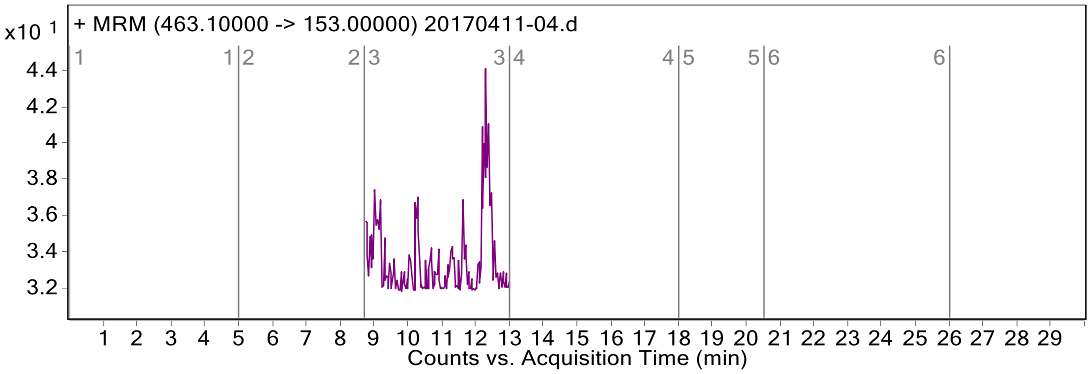

x10 1
+ MRM (463.10000 -> 153.00000) 20170411-04.d
Counts vs. Acquisition Time (min)

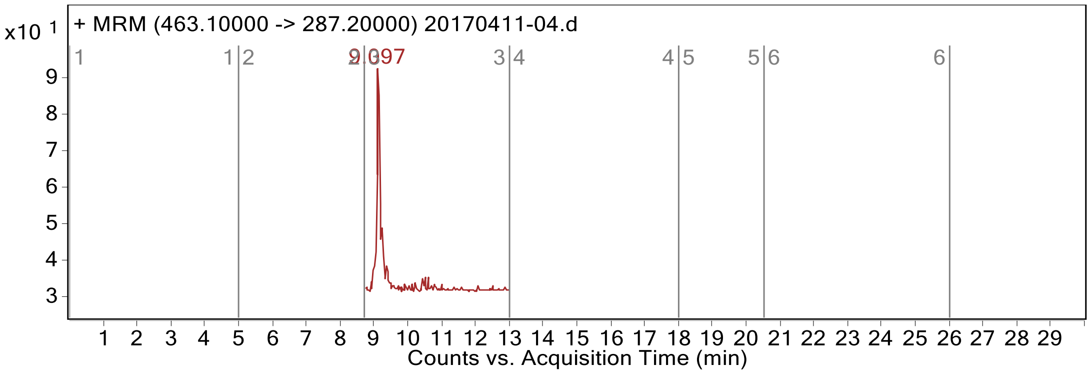

x10 1
+ MRM (463.10000 -> 287.20000) 20170411-04.d
9.097
Counts vs. Acquisition Time (min)

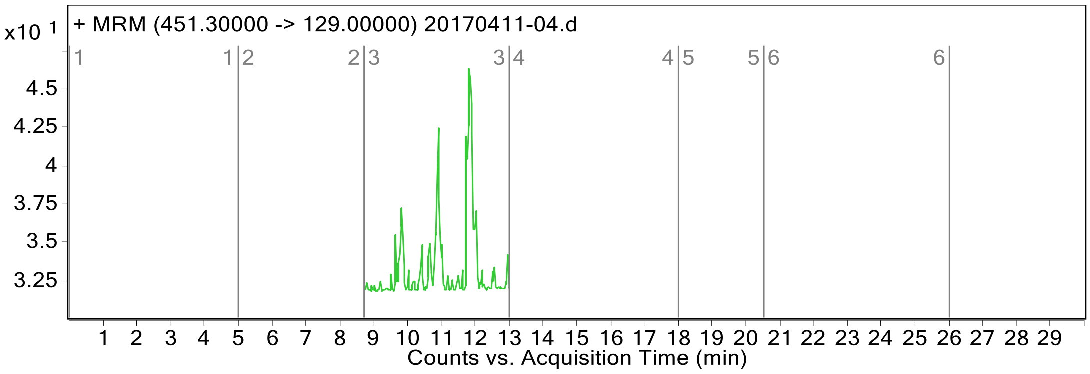

x10 1
+ MRM (451.30000 -> 129.00000) 20170411-04.d
Counts vs. Acquisition Time (min)

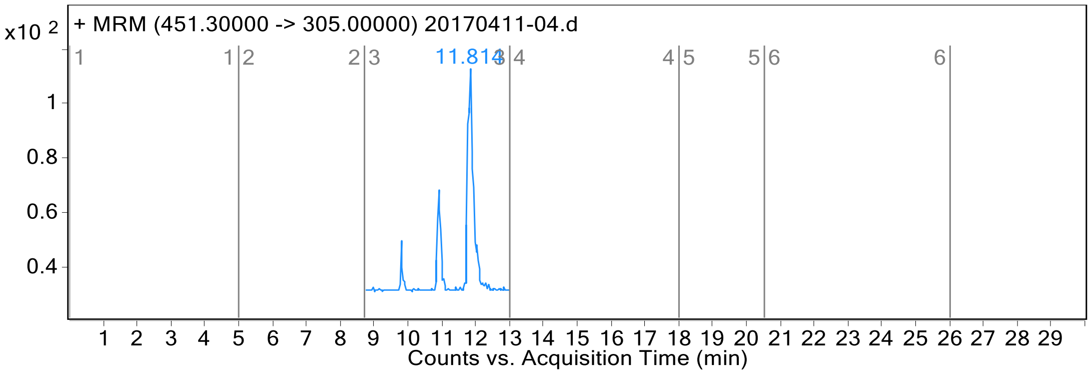
x10 2
+ MRM (451.30000 -> 305.00000) 20170411-04.d
11.814
Counts vs. Acquisition Time (min)

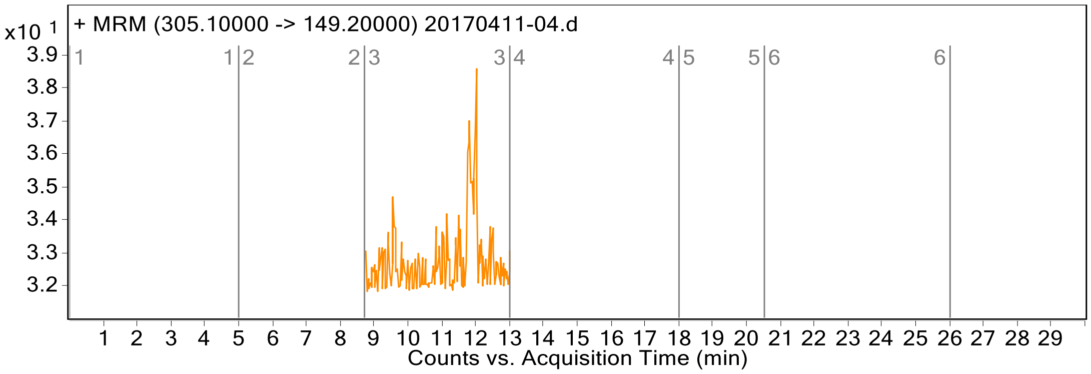
x10 1
+ MRM (305.10000 -> 149.20000) 20170411-04.d
Counts vs. Acquisition Time (min)

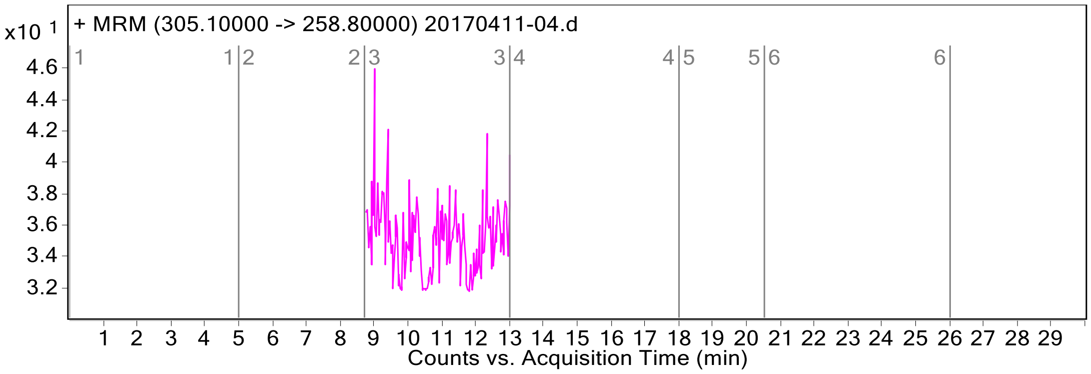
x10 1
+ MRM (305.10000 -> 258.80000) 20170411-04.d
Counts vs. Acquisition Time (min)

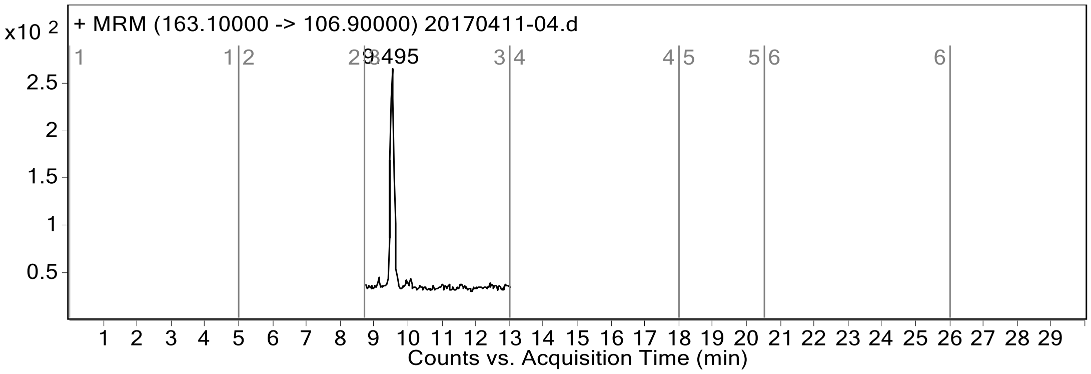
x10 2
+ MRM (163.10000 -> 106.90000) 20170411-04.d
9.495
Counts vs. Acquisition Time (min)

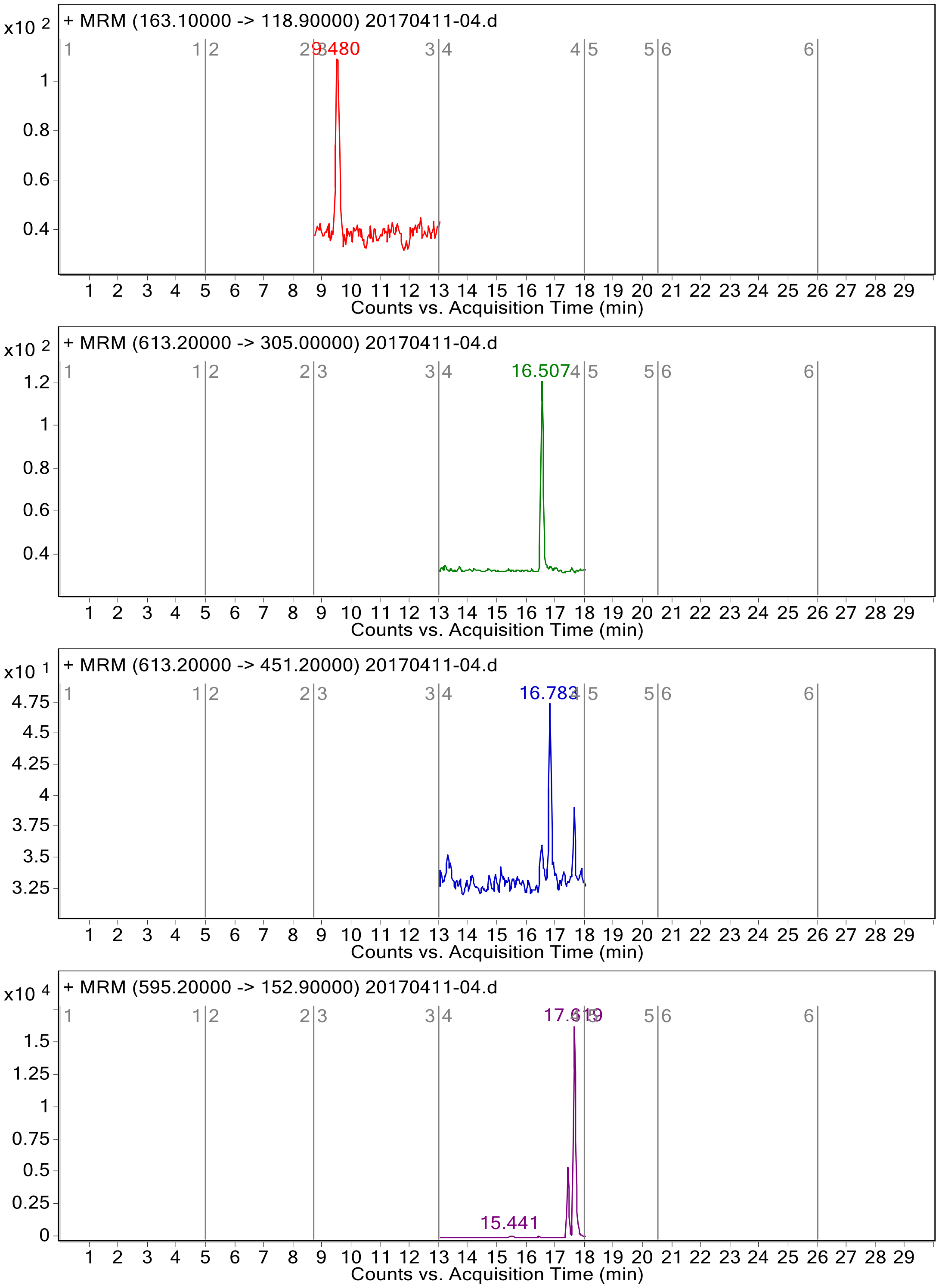

+ MRM (163.10000 -> 118.90000) 20170411-04.d
x10 2
9.480
Counts vs. Acquisition Time (min)
+ MRM (613.20000 -> 305.00000) 20170411-04.d
x10 2
16.507
Counts vs. Acquisition Time (min)
+ MRM (613.20000 -> 451.20000) 20170411-04.d
x10 1
16.783
Counts vs. Acquisition Time (min)
+ MRM (595.20000 -> 152.90000) 20170411-04.d
x10 4
17.619
15.441
Counts vs. Acquisition Time (min)

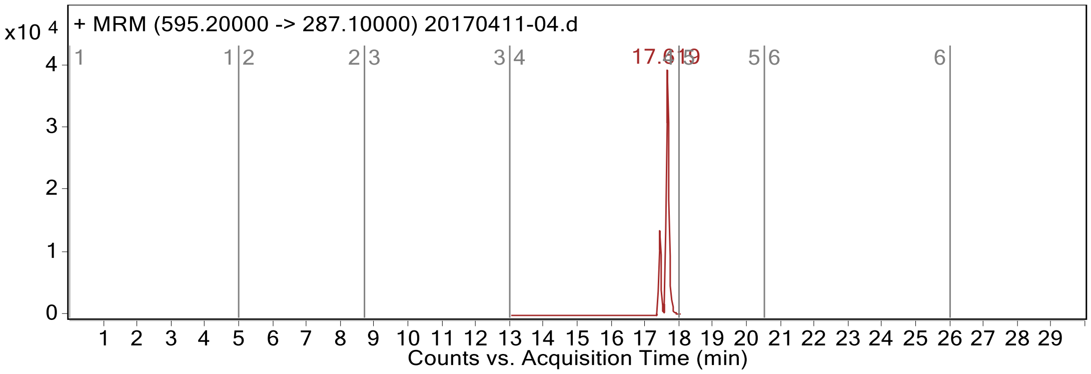
x10 4
+ MRM (595.20000 -> 287.10000) 20170411-04.d
Counts vs. Acquisition Time (min)

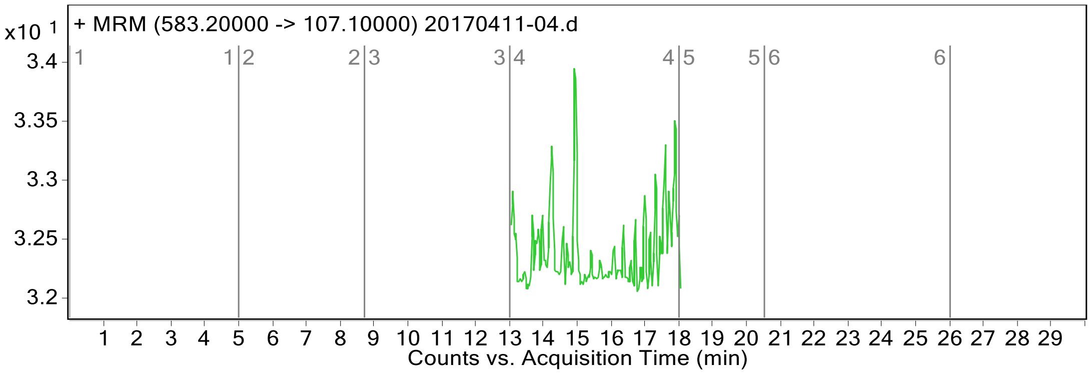
x10 1
+ MRM (583.20000 -> 107.10000) 20170411-04.d
Counts vs. Acquisition Time (min)

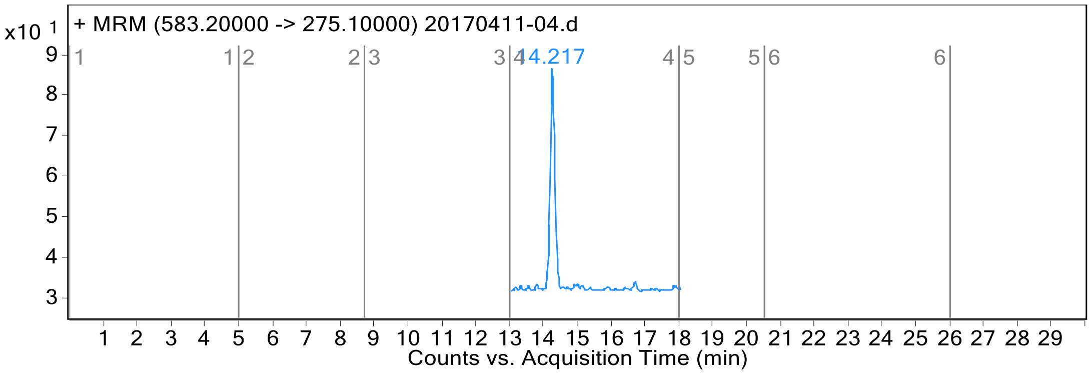
x10 1
+ MRM (583.20000 -> 275.10000) 20170411-04.d
14.217
Counts vs. Acquisition Time (min)

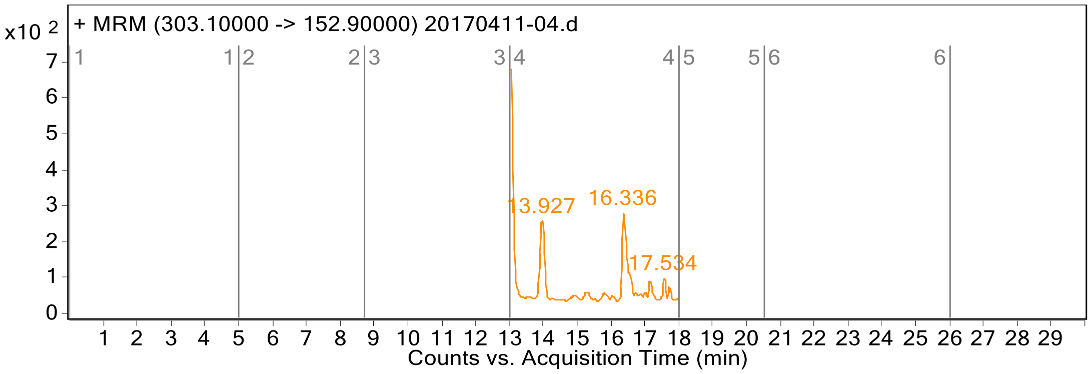
x10 2
+ MRM (303.10000 -> 152.90000) 20170411-04.d
13.927
16.336
17.534
Counts vs. Acquisition Time (min)

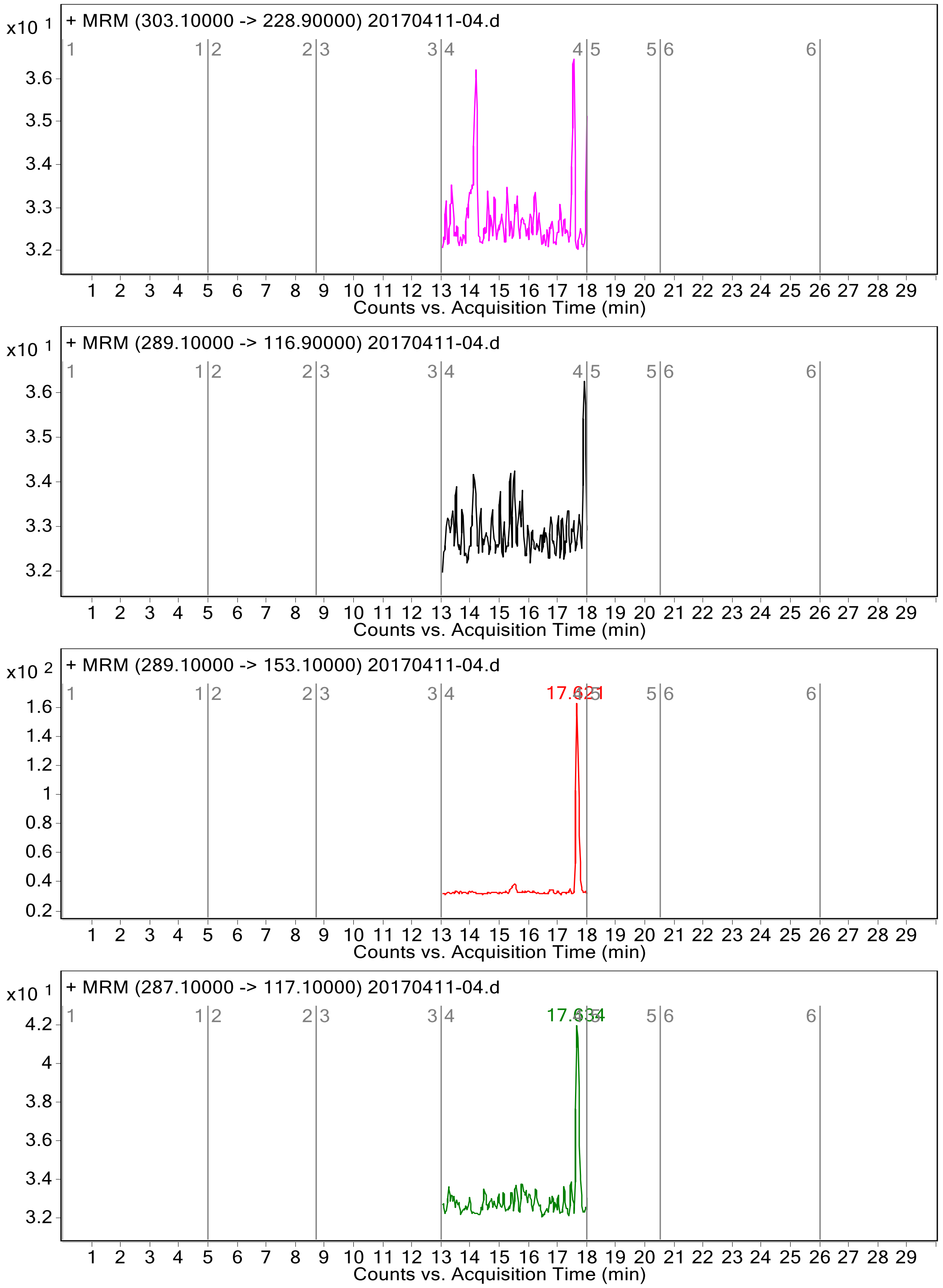
x10 1
+ MRM (303.10000 -> 228.90000) 20170411-04.d
Counts vs. Acquisition Time (min)
x10 1
+ MRM (289.10000 -> 116.90000) 20170411-04.d
Counts vs. Acquisition Time (min)
x10 2
+ MRM (289.10000 -> 153.10000) 20170411-04.d
Counts vs. Acquisition Time (min)
x10 1
+ MRM (287.10000 -> 117.10000) 20170411-04.d
Counts vs. Acquisition Time (min)

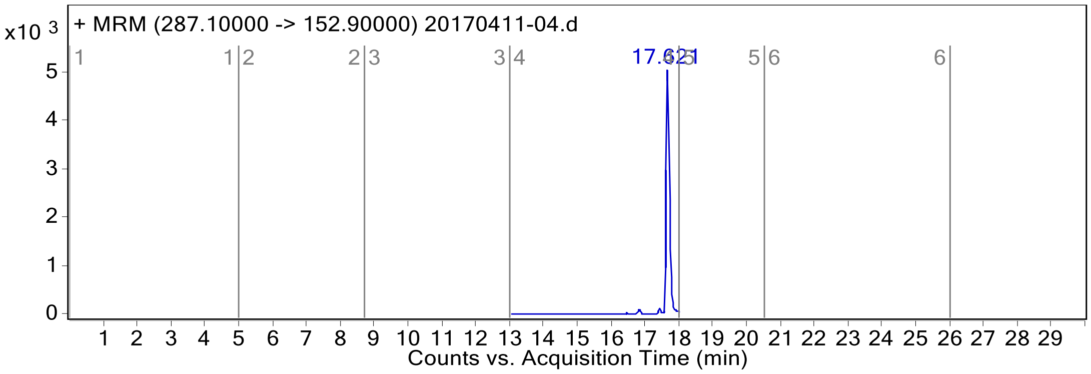
x10 3
+ MRM (287.10000 -> 152.90000) 20170411-04.d
17.621
Counts vs. Acquisition Time (min)

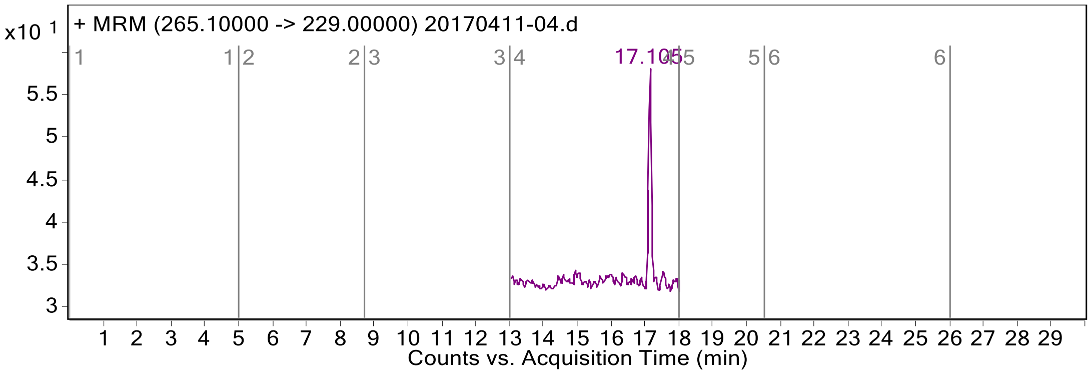
x10 1
+ MRM (265.10000 -> 229.00000) 20170411-04.d
17.105
Counts vs. Acquisition Time (min)

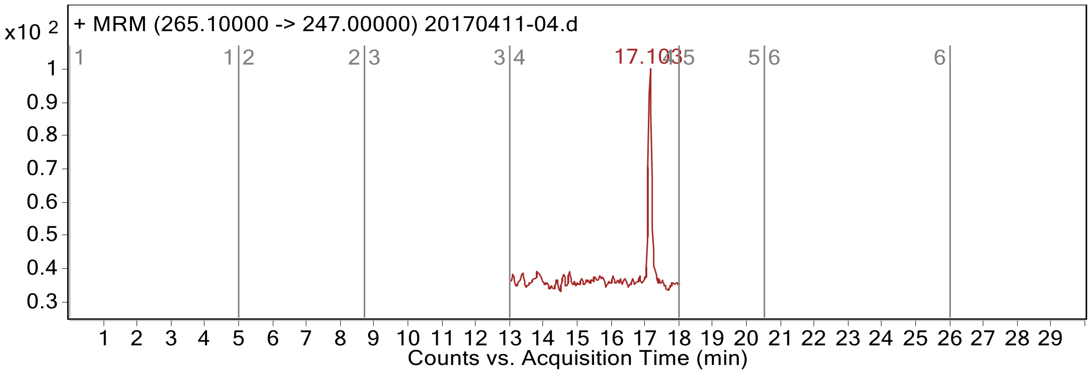
x10 2
+ MRM (265.10000 -> 247.00000) 20170411-04.d
17.103
Counts vs. Acquisition Time (min)

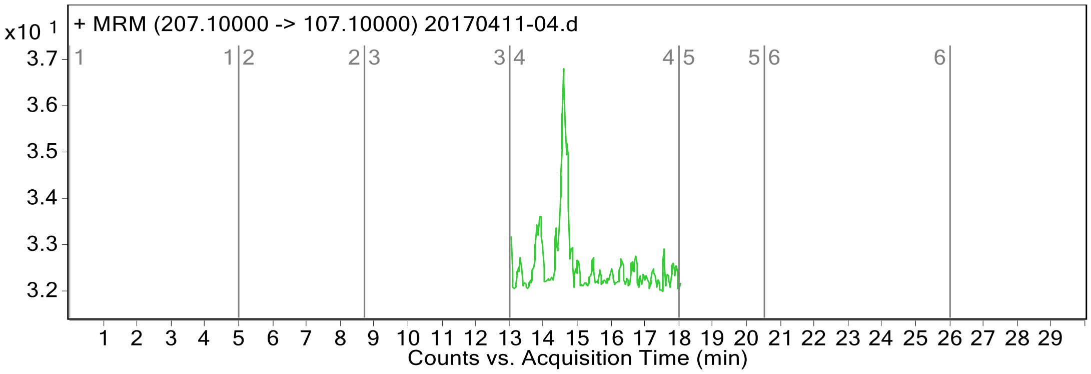
x10 1
+ MRM (207.10000 -> 107.10000) 20170411-04.d
Counts vs. Acquisition Time (min)

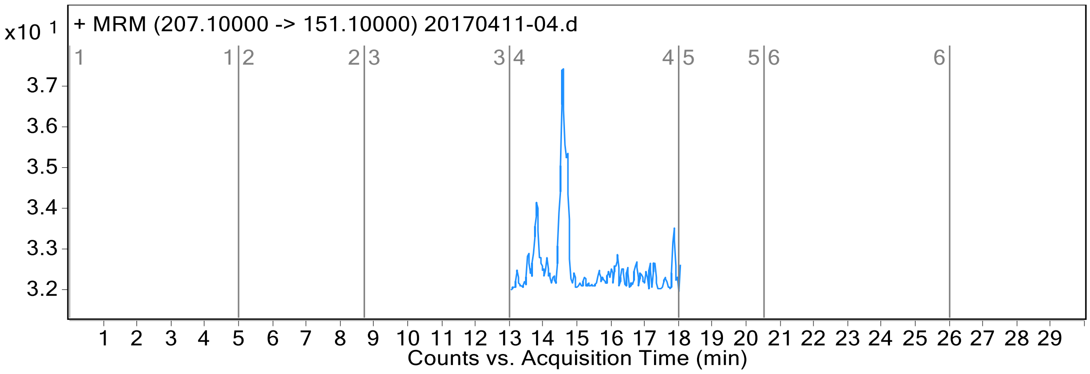
x10 1
+ MRM (207.10000 -> 151.10000) 20170411-04.d
3.7
3.6
3.5
3.4
3.3
3.2
1 2 3 4 5 6 7 8 9 10 11 12 13 14 15 16 17 18 19 20 21 22 23 24 25 26 27 28 29
Counts vs. Acquisition Time (min)

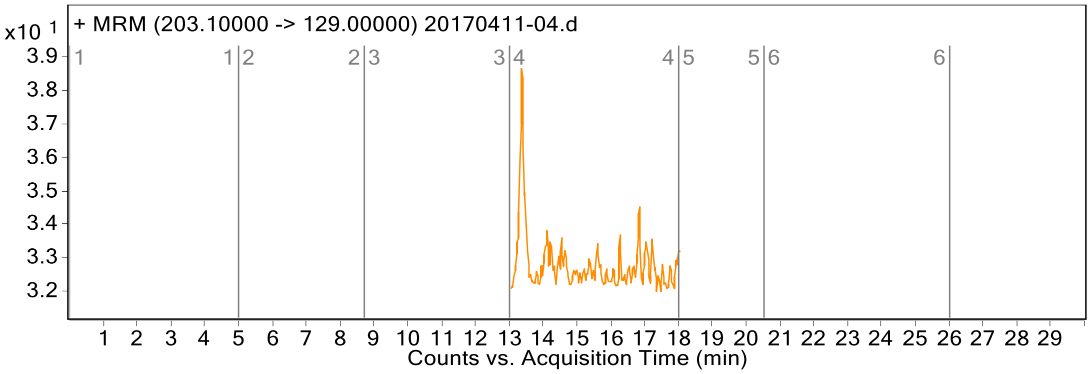
x10 1
+ MRM (203.10000 -> 129.00000) 20170411-04.d
3.9
3.8
3.7
3.6
3.5
3.4
3.3
3.2
1 2 3 4 5 6 7 8 9 10 11 12 13 14 15 16 17 18 19 20 21 22 23 24 25 26 27 28 29
Counts vs. Acquisition Time (min)

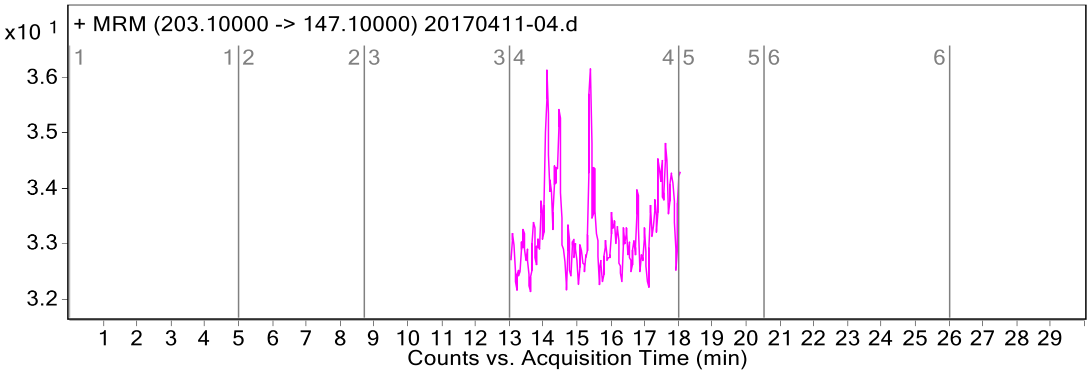
x10 1
+ MRM (203.10000 -> 147.10000) 20170411-04.d
3.6
3.5
3.4
3.3
3.2
1 2 3 4 5 6 7 8 9 10 11 12 13 14 15 16 17 18 19 20 21 22 23 24 25 26 27 28 29
Counts vs. Acquisition Time (min)

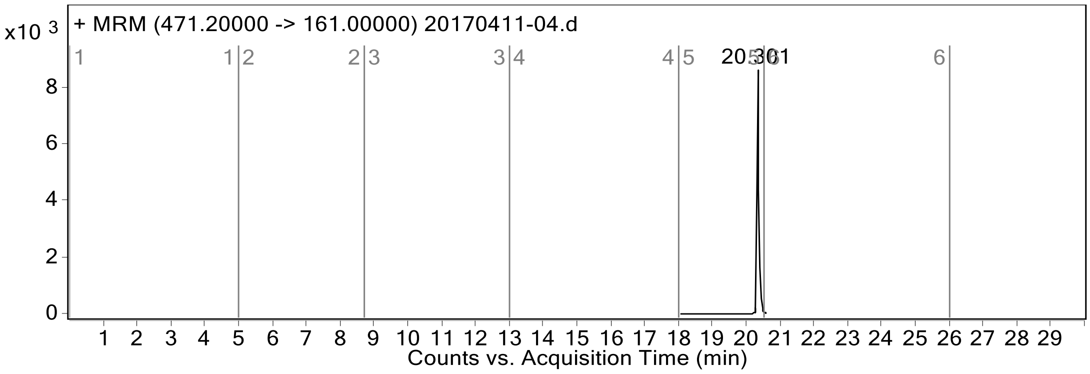
x10 3
+ MRM (471.20000 -> 161.00000) 20170411-04.d
8
6
4
2
0
1 2 3 4 5 6 7 8 9 10 11 12 13 14 15 16 17 18 19 20 21 22 23 24 25 26 27 28 29
Counts vs. Acquisition Time (min)

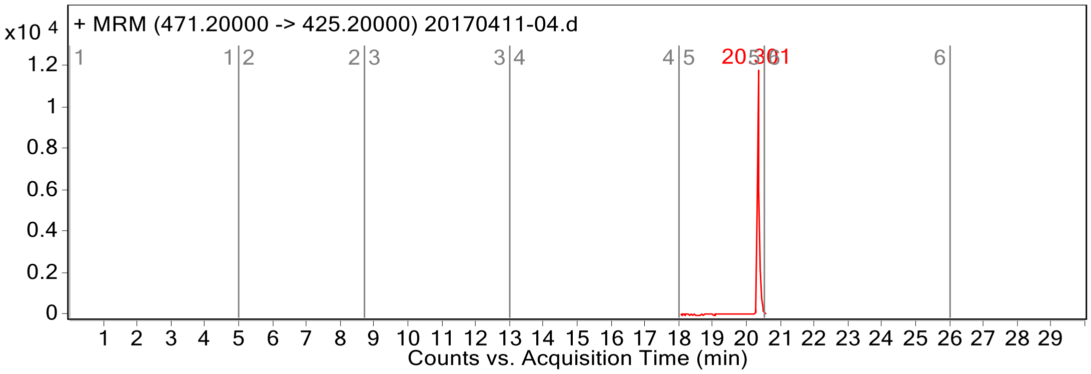
+ MRM (471.20000 -> 425.20000) 20170411-04.d
20.301
Counts vs. Acquisition Time (min)

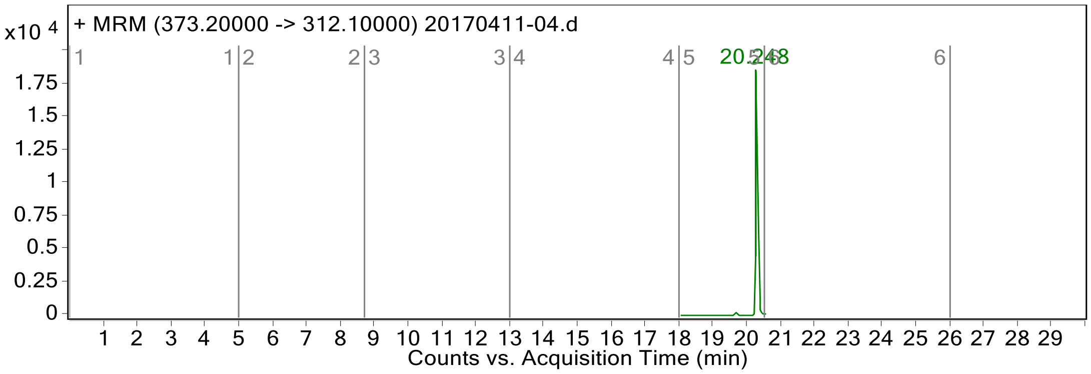
+ MRM (373.20000 -> 312.10000) 20170411-04.d
20.248
Counts vs. Acquisition Time (min)

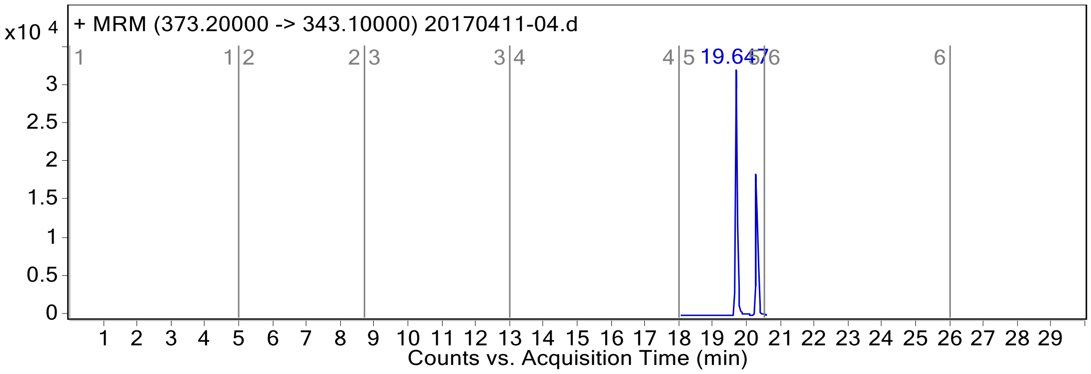
+ MRM (373.20000 -> 343.10000) 20170411-04.d
19.647
Counts vs. Acquisition Time (min)

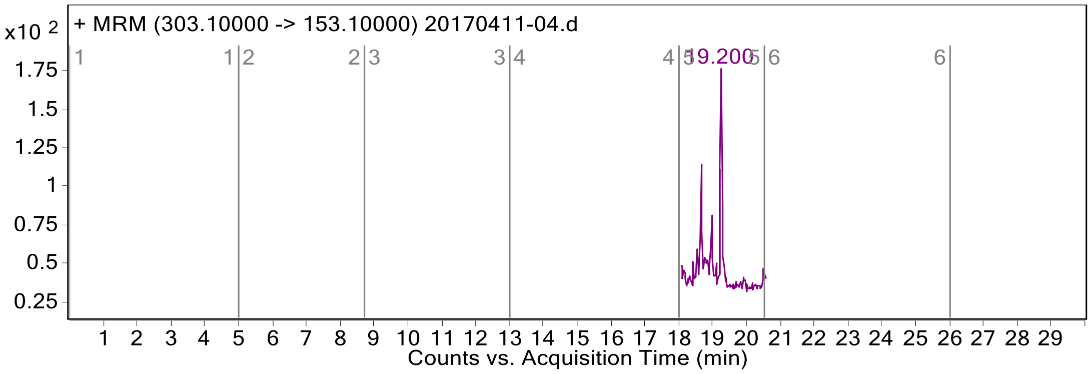
+ MRM (303.10000 -> 153.10000) 20170411-04.d
19.200
Counts vs. Acquisition Time (min)

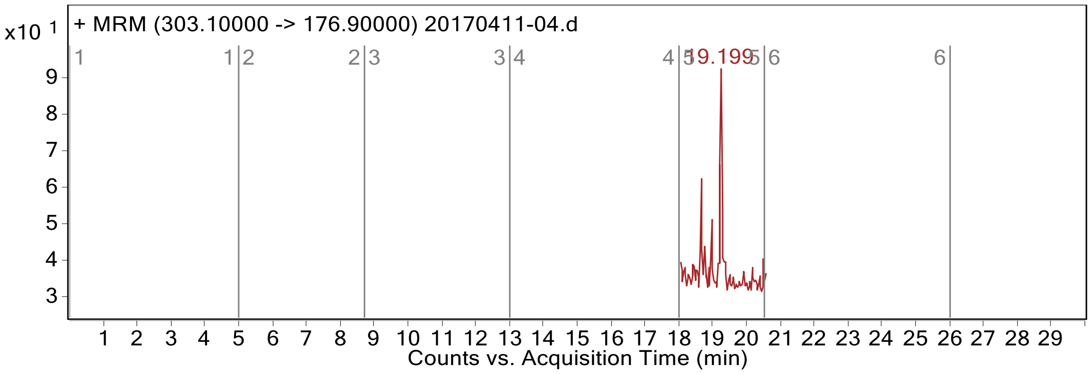
x10 1
+ MRM (303.10000 -> 176.90000) 20170411-04.d
19.199
Counts vs. Acquisition Time (min)

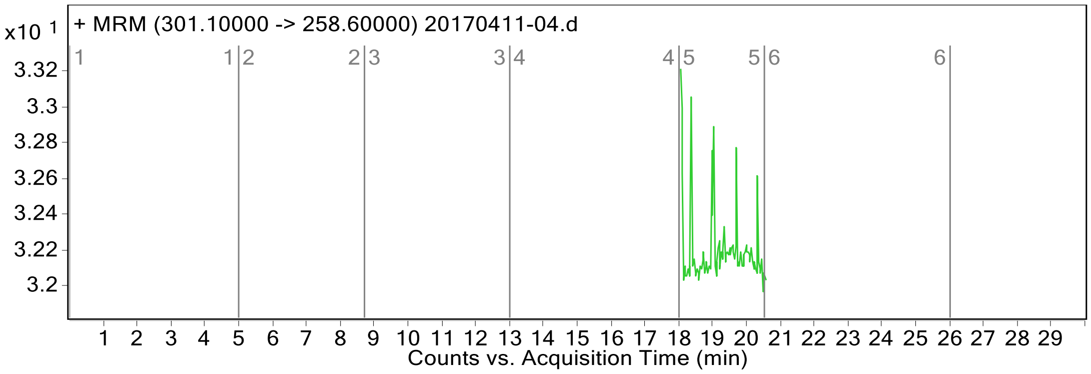
x10 1
+ MRM (301.10000 -> 258.60000) 20170411-04.d
Counts vs. Acquisition Time (min)

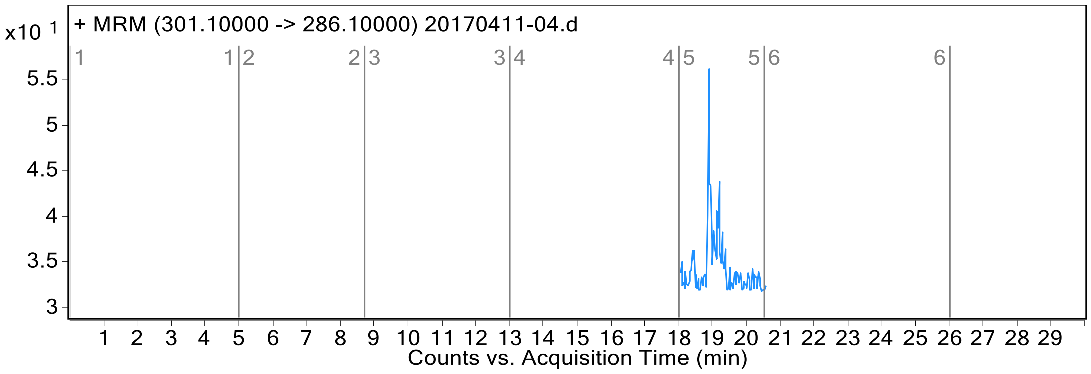
x10 1
+ MRM (301.10000 -> 286.10000) 20170411-04.d
Counts vs. Acquisition Time (min)

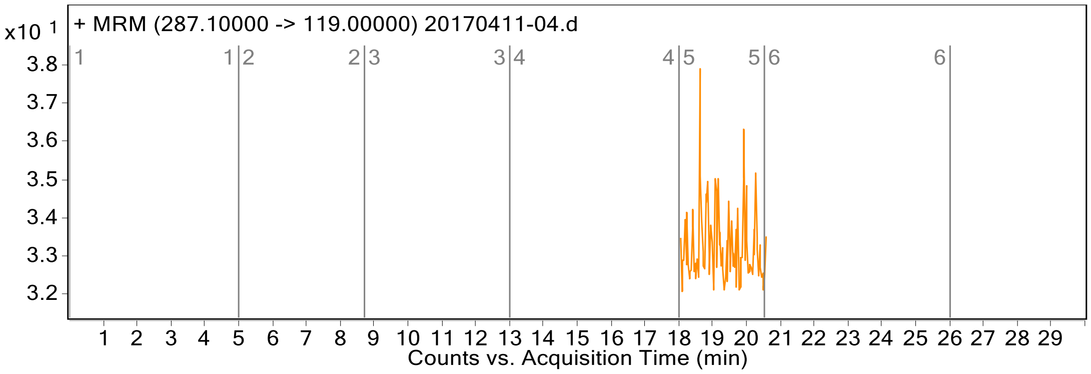
x10 1
+ MRM (287.10000 -> 119.00000) 20170411-04.d
Counts vs. Acquisition Time (min)

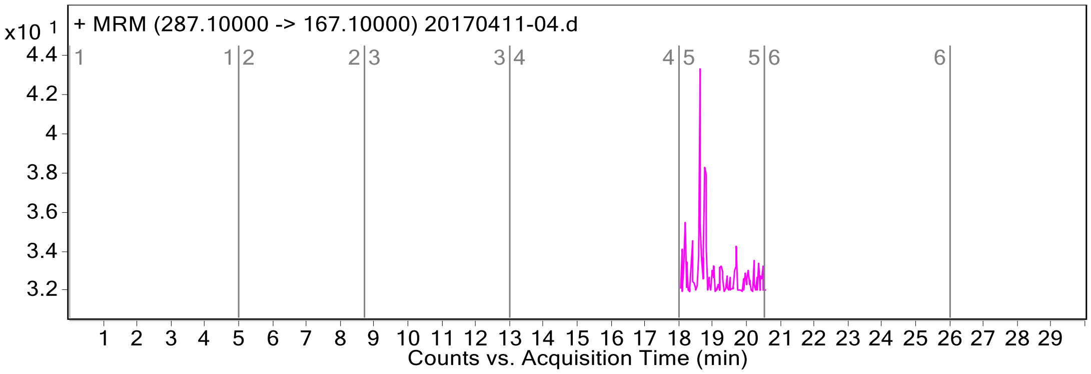
+ MRM (287.10000 -> 167.10000) 20170411-04.d
x10 1
Counts vs. Acquisition Time (min)

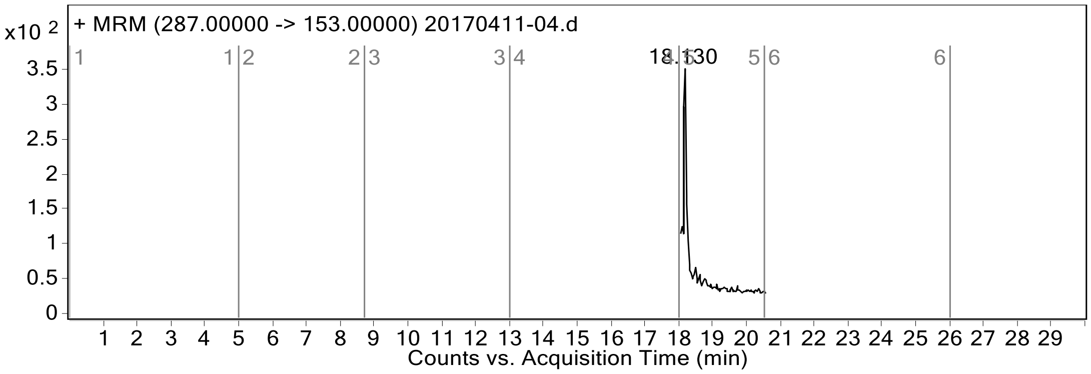
+ MRM (287.00000 -> 153.00000) 20170411-04.d
x10 2
18.130
Counts vs. Acquisition Time (min)

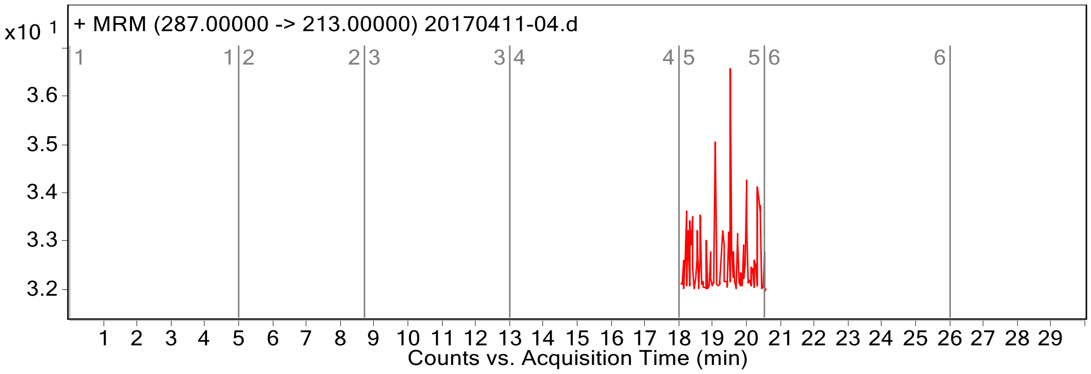
+ MRM (287.00000 -> 213.00000) 20170411-04.d
x10 1
Counts vs. Acquisition Time (min)

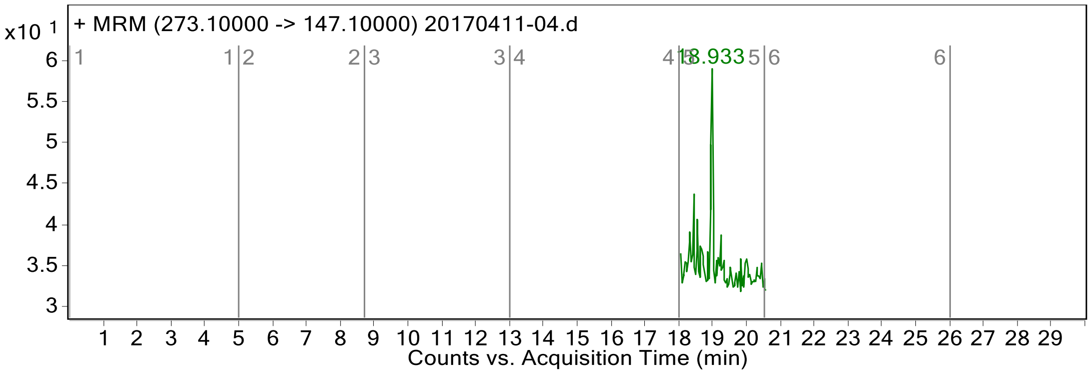
+ MRM (273.10000 -> 147.10000) 20170411-04.d
x10 1
18.933
Counts vs. Acquisition Time (min)

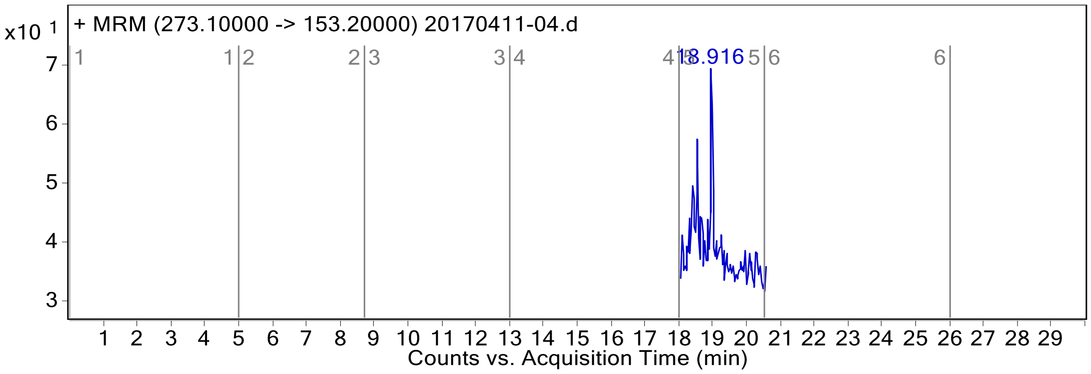
+ MRM (273.10000 -> 153.20000) 20170411-04.d
x10 1
18.916
Counts vs. Acquisition Time (min)

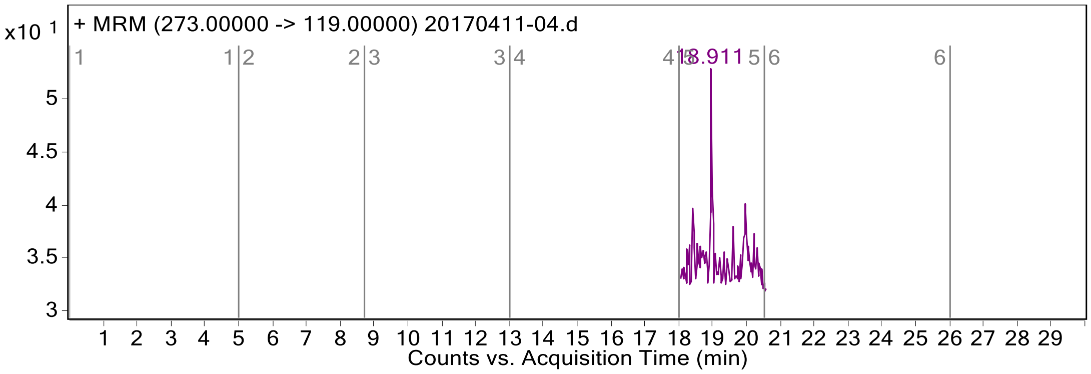
+ MRM (273.00000 -> 119.00000) 20170411-04.d
x10 1
18.911
Counts vs. Acquisition Time (min)

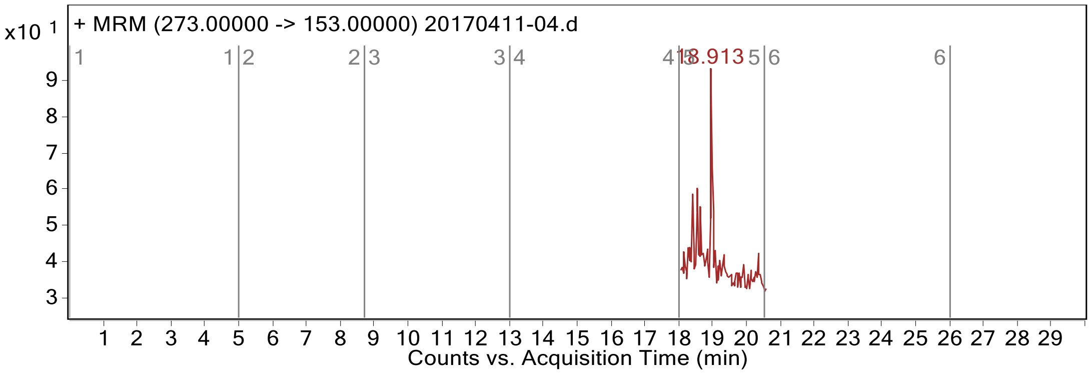
+ MRM (273.00000 -> 153.00000) 20170411-04.d
x10 1
18.913
Counts vs. Acquisition Time (min)

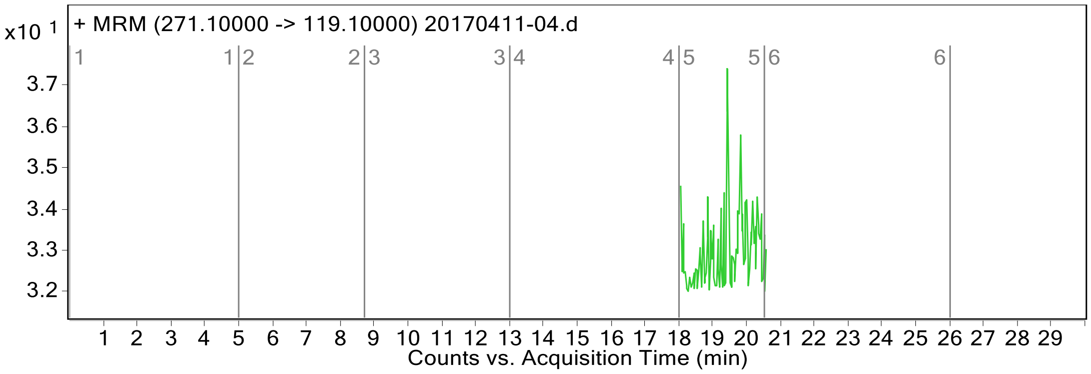
+ MRM (271.10000 -> 119.10000) 20170411-04.d
x10 1
Counts vs. Acquisition Time (min)

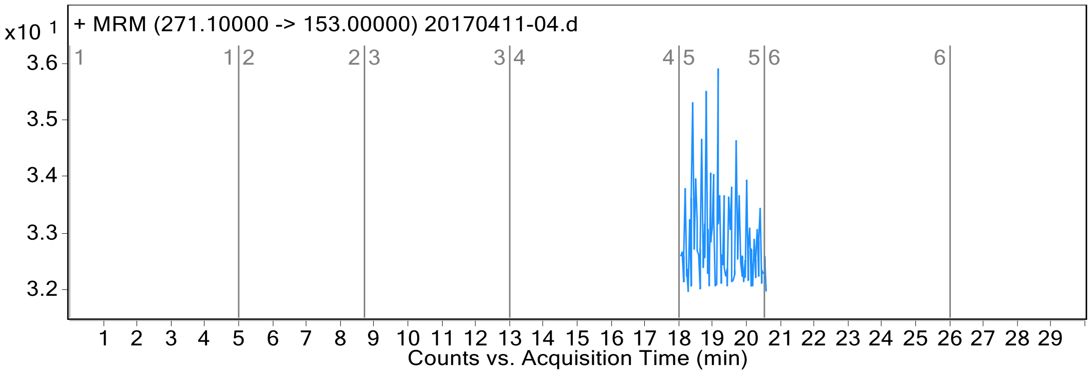
+ MRM (271.10000 -> 153.00000) 20170411-04.d
x10 1
Counts vs. Acquisition Time (min)

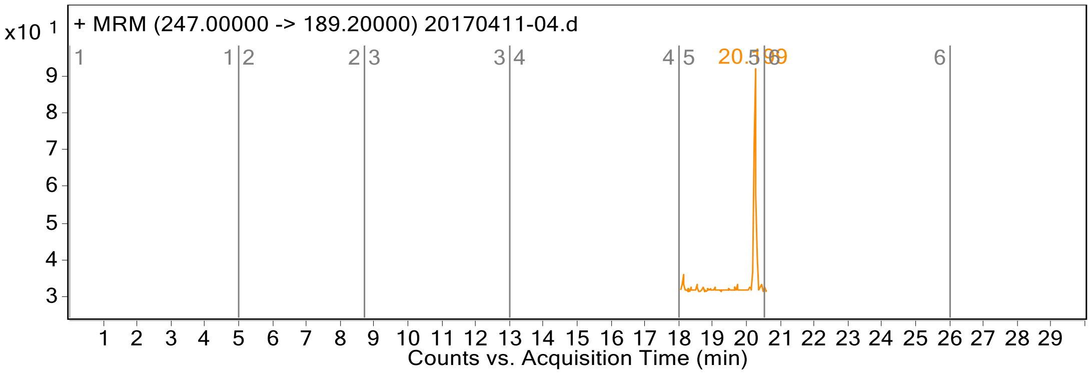
+ MRM (247.00000 -> 189.20000) 20170411-04.d
x10 1
Counts vs. Acquisition Time (min)

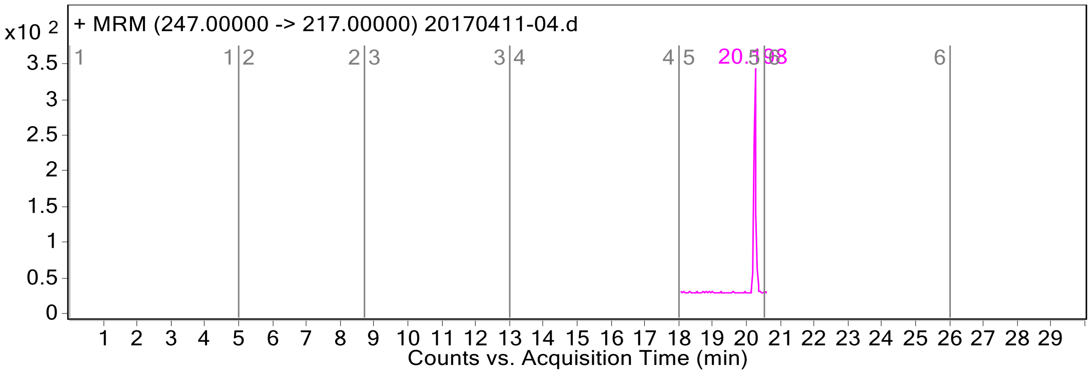
+ MRM (247.00000 -> 217.00000) 20170411-04.d
x10 2
Counts vs. Acquisition Time (min)

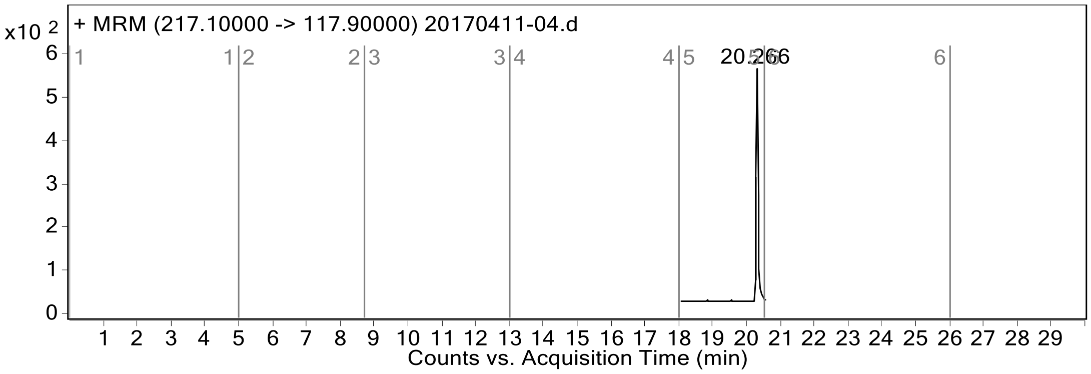
+ MRM (217.10000 -> 117.90000) 20170411-04.d
x10 2
Counts vs. Acquisition Time (min)

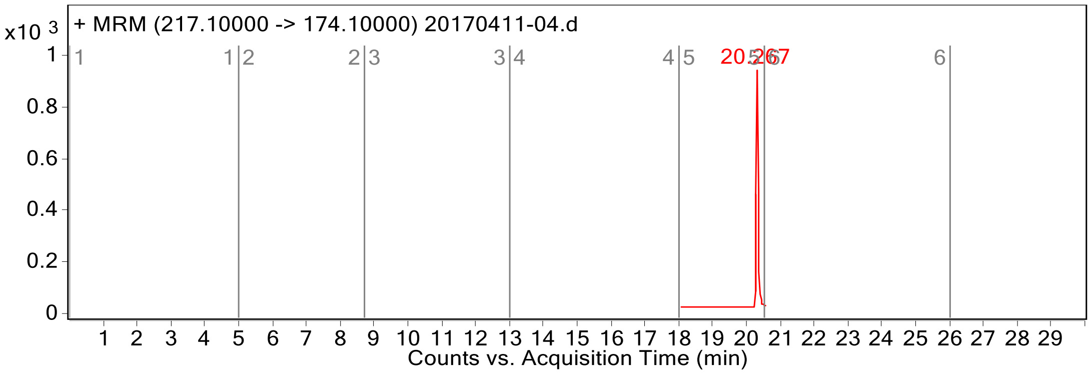
+ MRM (217.10000 -> 174.10000) 20170411-04.d
x10 3
20.267
Counts vs. Acquisition Time (min)

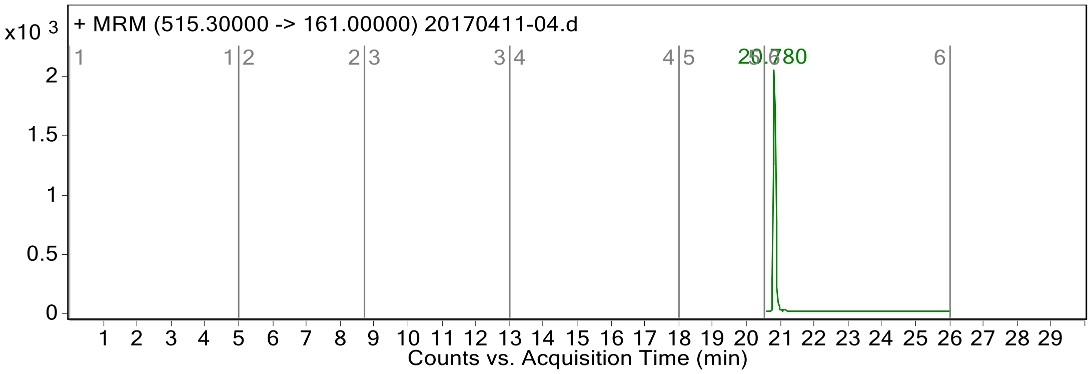
+ MRM (515.30000 -> 161.00000) 20170411-04.d
x10 3
20.780
Counts vs. Acquisition Time (min)

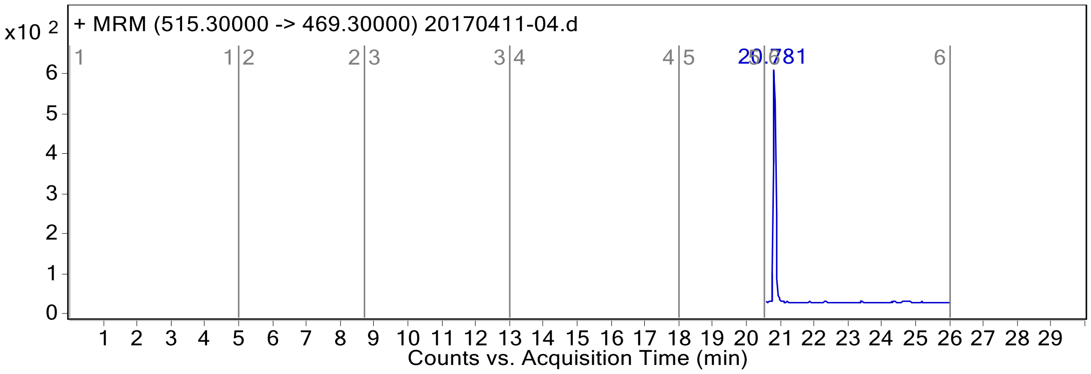
+ MRM (515.30000 -> 469.30000) 20170411-04.d
x10 2
20.781
Counts vs. Acquisition Time (min)

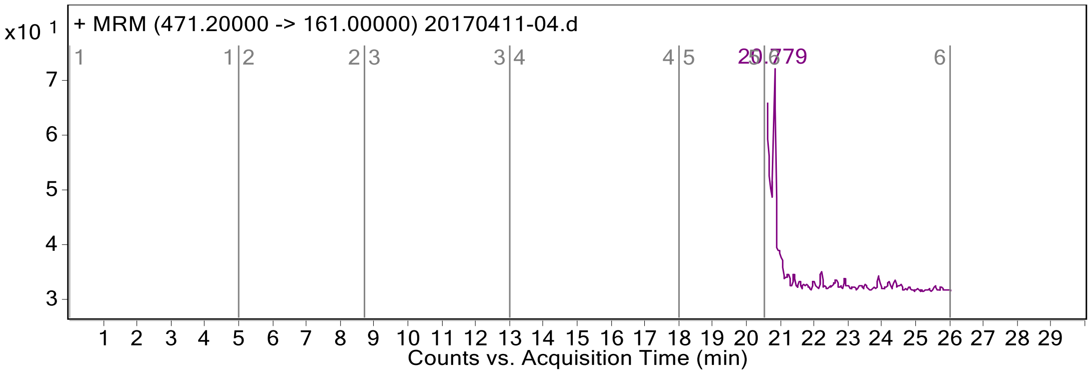
+ MRM (471.20000 -> 161.00000) 20170411-04.d
x10 1
20.779
Counts vs. Acquisition Time (min)

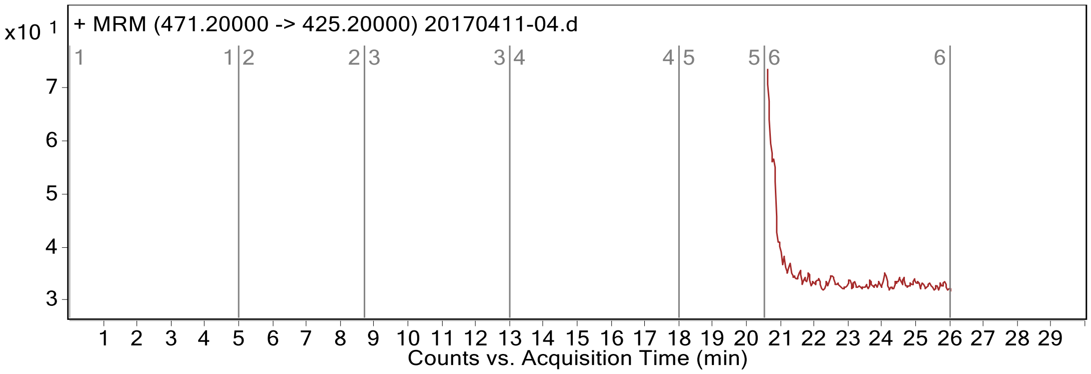
x10 1
+ MRM (471.20000 -> 425.20000) 20170411-04.d
Counts vs. Acquisition Time (min)

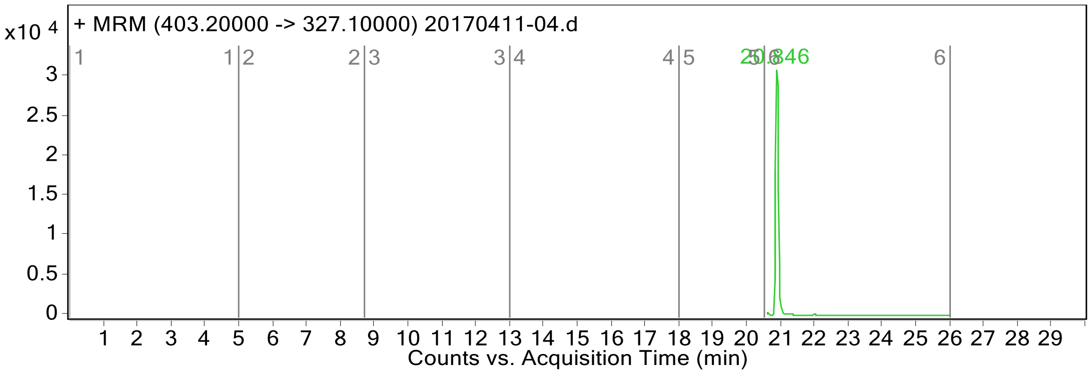
x10 4
+ MRM (403.20000 -> 327.10000) 20170411-04.d
20.846
Counts vs. Acquisition Time (min)

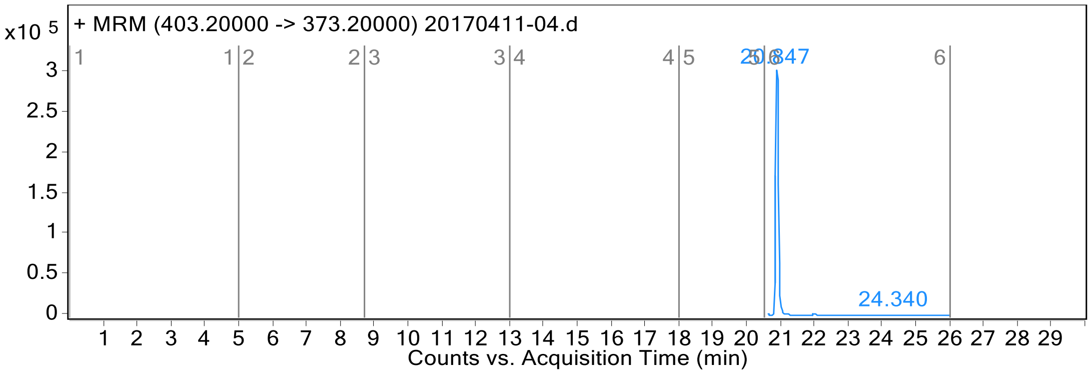
x10 5
+ MRM (403.20000 -> 373.20000) 20170411-04.d
20.847
24.340
Counts vs. Acquisition Time (min)

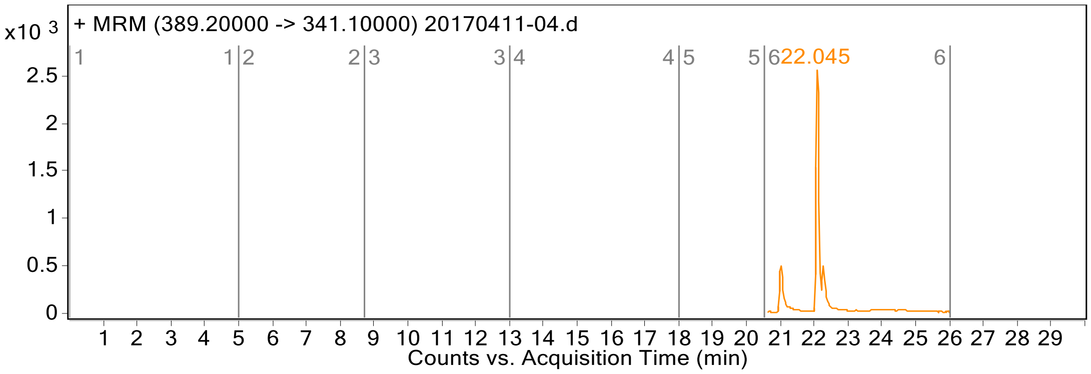
x10 3
+ MRM (389.20000 -> 341.10000) 20170411-04.d
22.045
Counts vs. Acquisition Time (min)

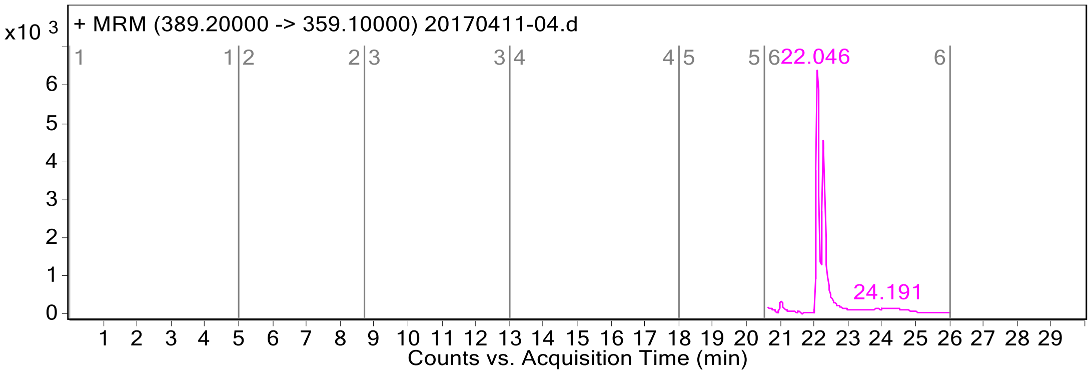

x10 3
+ MRM (389.20000 -> 359.10000) 20170411-04.d
22.046
24.191
Counts vs. Acquisition Time (min)

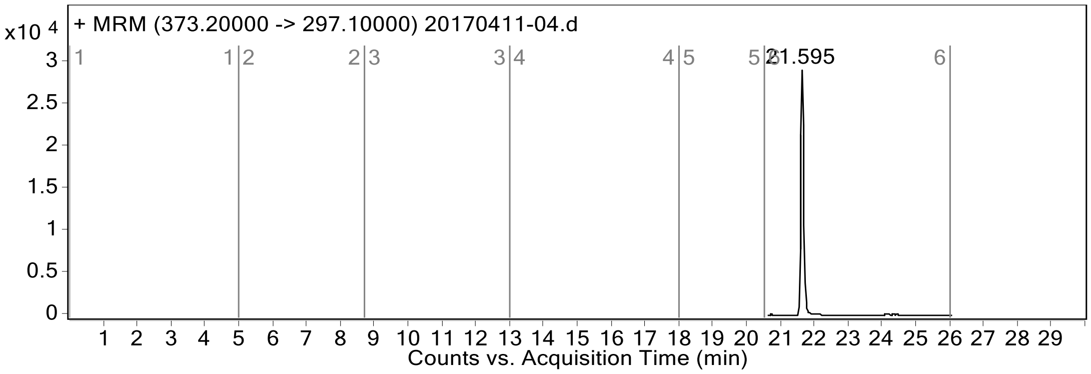

x10 4
+ MRM (373.20000 -> 297.10000) 20170411-04.d
21.595
Counts vs. Acquisition Time (min)

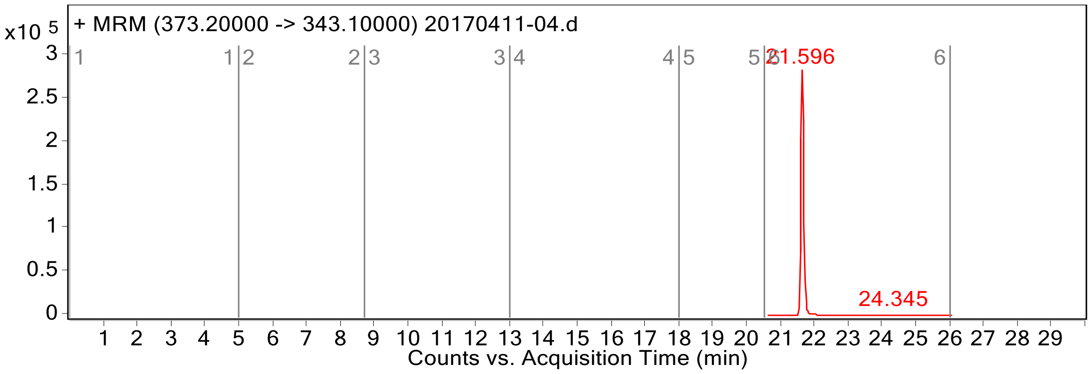

x10 5
+ MRM (373.20000 -> 343.10000) 20170411-04.d
21.596
24.345
Counts vs. Acquisition Time (min)

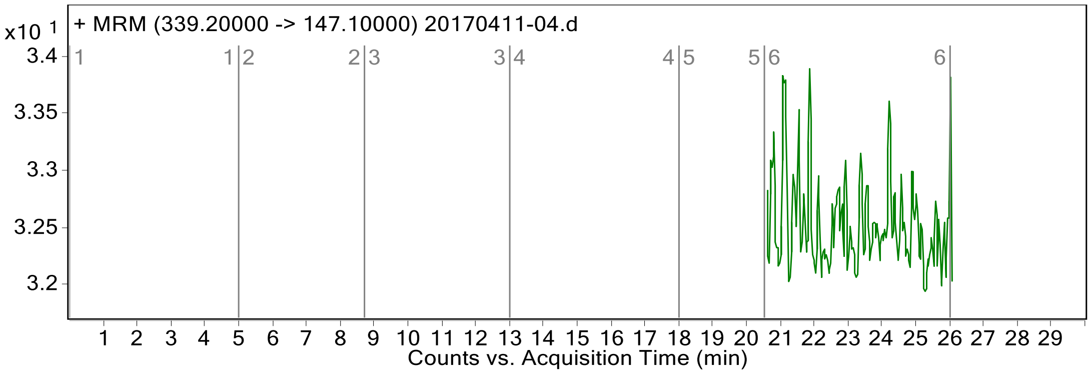

x10 1
+ MRM (339.20000 -> 147.10000) 20170411-04.d
Counts vs. Acquisition Time (min)

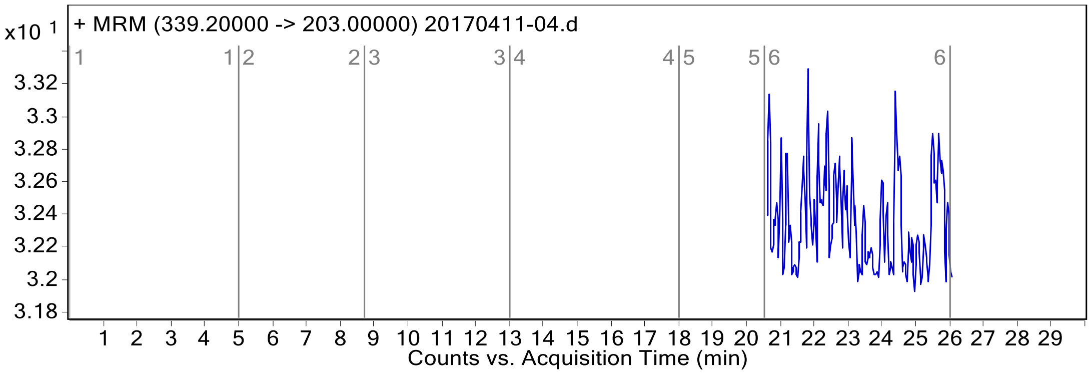
+ MRM (339.20000 -> 203.00000) 20170411-04.d
x10 1
Counts vs. Acquisition Time (min)

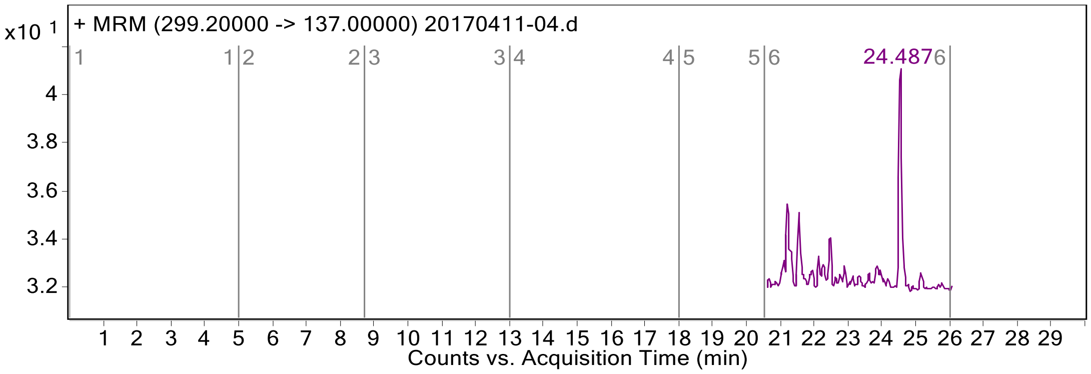
+ MRM (299.20000 -> 137.00000) 20170411-04.d
x10 1
24.4876
Counts vs. Acquisition Time (min)

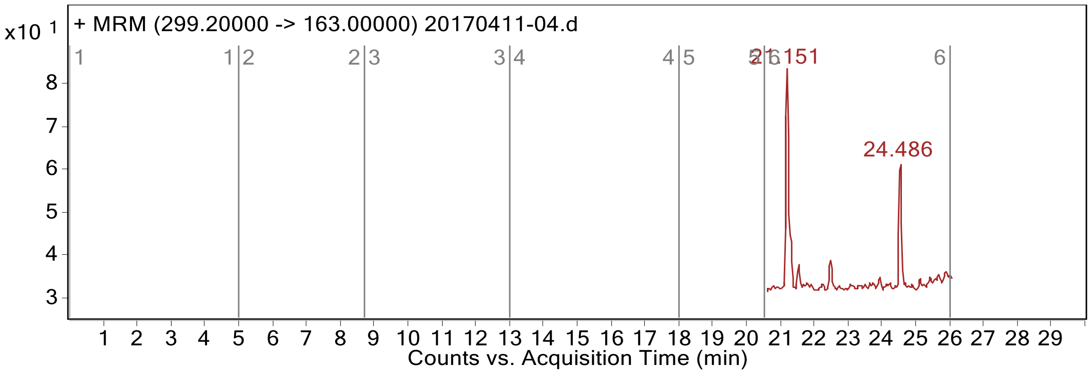
+ MRM (299.20000 -> 163.00000) 20170411-04.d
x10 1
21.151
24.486
Counts vs. Acquisition Time (min)

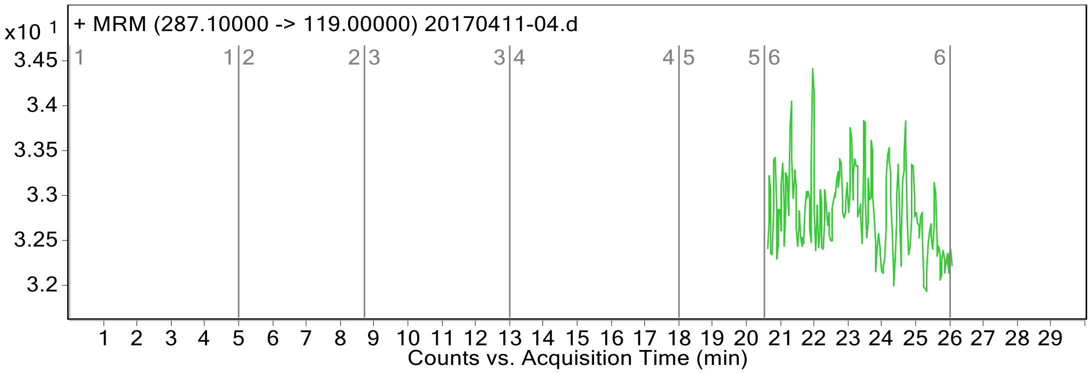
+ MRM (287.10000 -> 119.00000) 20170411-04.d
x10 1
Counts vs. Acquisition Time (min)

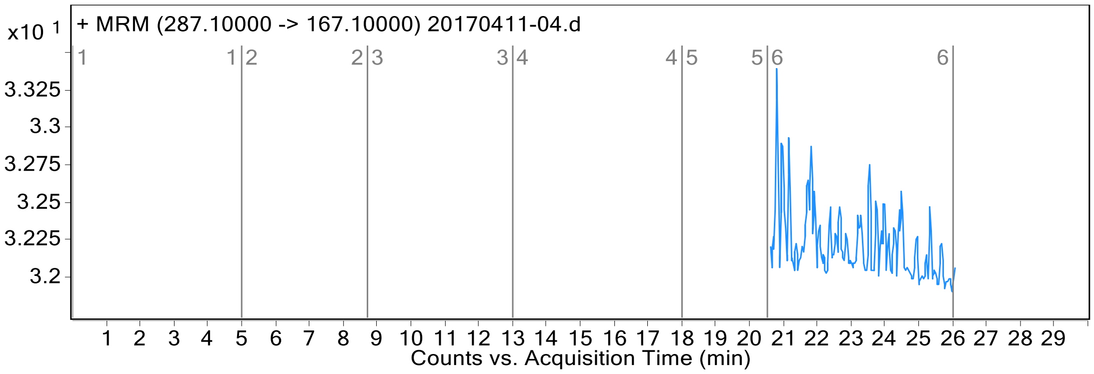

x10 1
+ MRM (287.10000 -> 167.10000) 20170411-04.d
Counts vs. Acquisition Time (min)

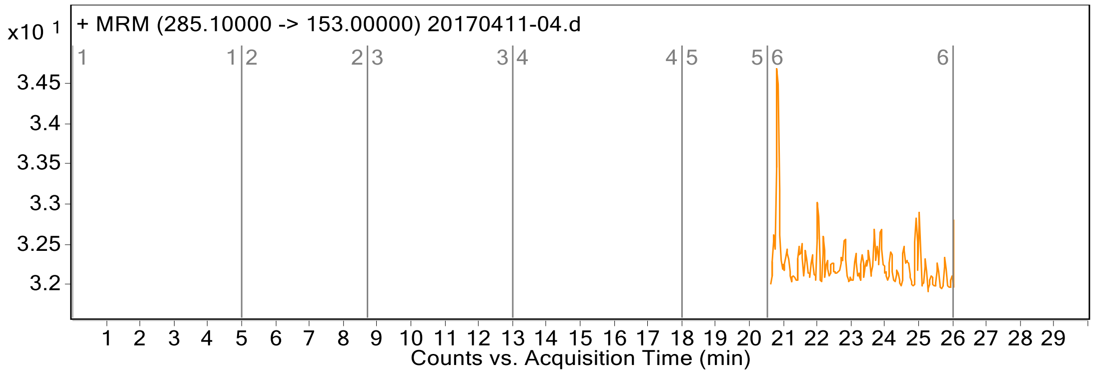

x10 1
+ MRM (285.10000 -> 153.00000) 20170411-04.d
Counts vs. Acquisition Time (min)

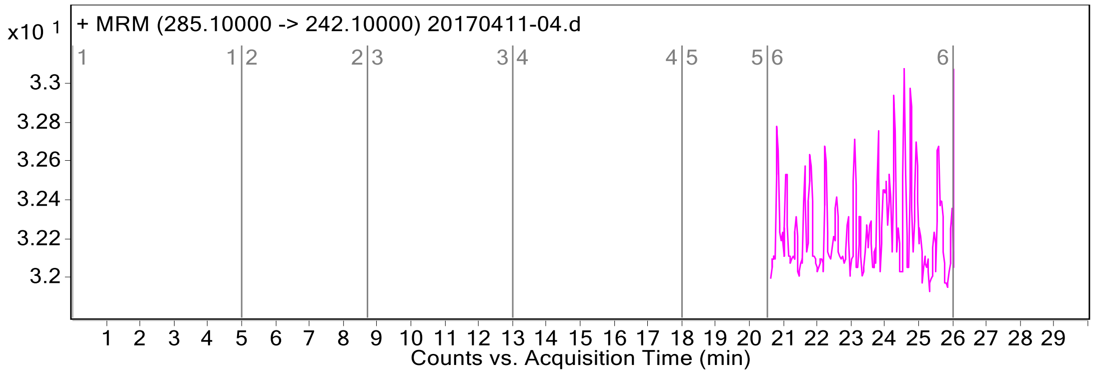

x10 1
+ MRM (285.10000 -> 242.10000) 20170411-04.d
Counts vs. Acquisition Time (min)

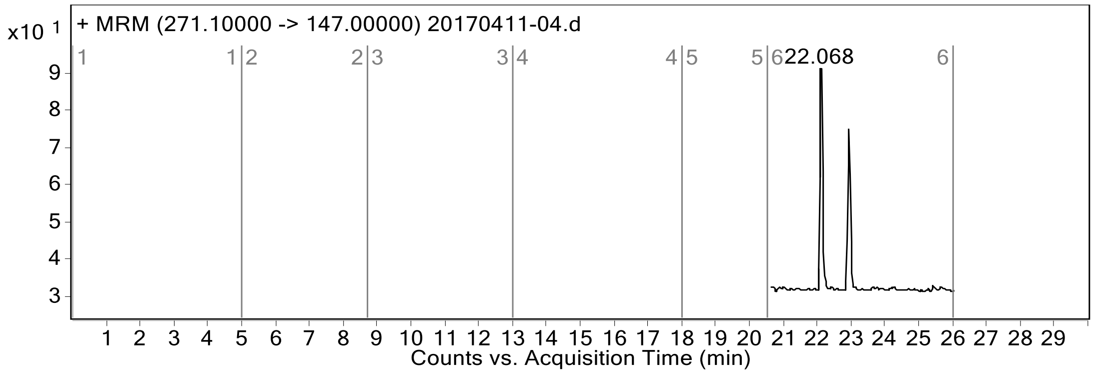

x10 1
+ MRM (271.10000 -> 147.00000) 20170411-04.d
22.068
Counts vs. Acquisition Time (min)

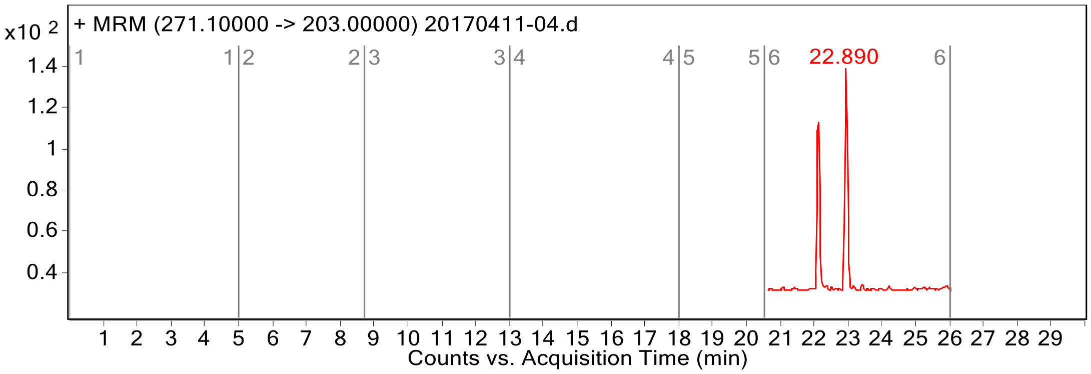
+ MRM (271.10000 -> 203.00000) 20170411-04.d
x10 2
22.890
Counts vs. Acquisition Time (min)

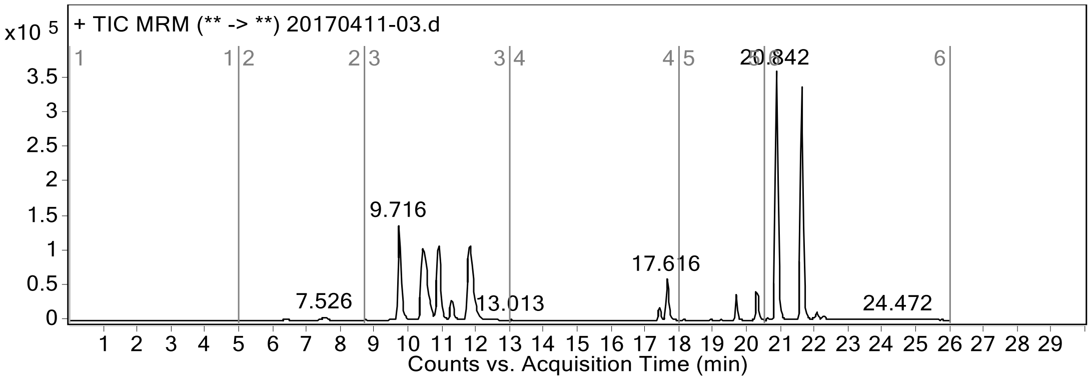
+ TIC MRM (** -> **) 20170411-03.d
x10 5
9.716
7.526
13.013
17.616
20.842
24.472
Counts vs. Acquisition Time (min)

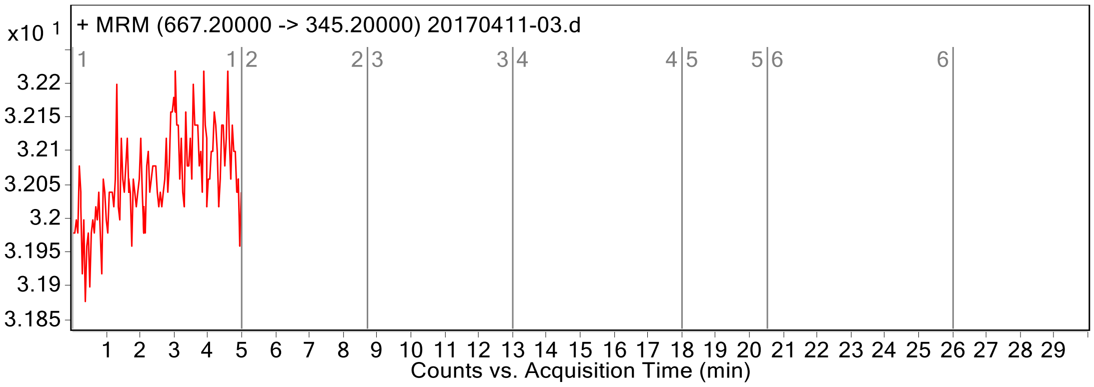
+ MRM (667.20000 -> 345.20000) 20170411-03.d
x10 1
Counts vs. Acquisition Time (min)

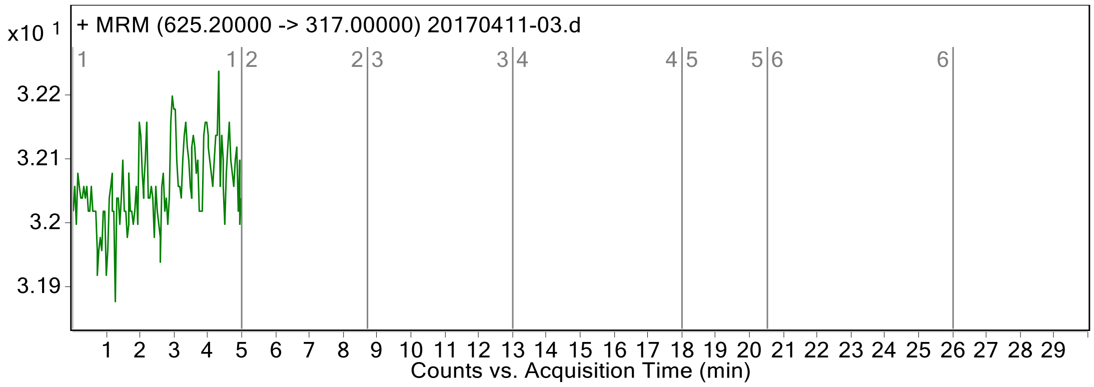
+ MRM (625.20000 -> 317.00000) 20170411-03.d
x10 1
Counts vs. Acquisition Time (min)

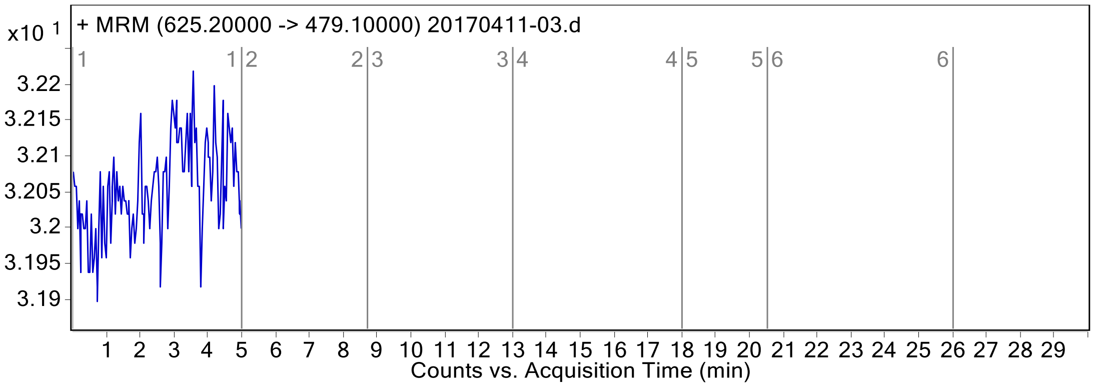
x10 1
+ MRM (625.20000 -> 479.10000) 20170411-03.d
3.22
3.215
3.21
3.205
3.2
3.195
3.19
1 2 3 4 5 6 7 8 9 10 11 12 13 14 15 16 17 18 19 20 21 22 23 24 25 26 27 28 29
Counts vs. Acquisition Time (min)

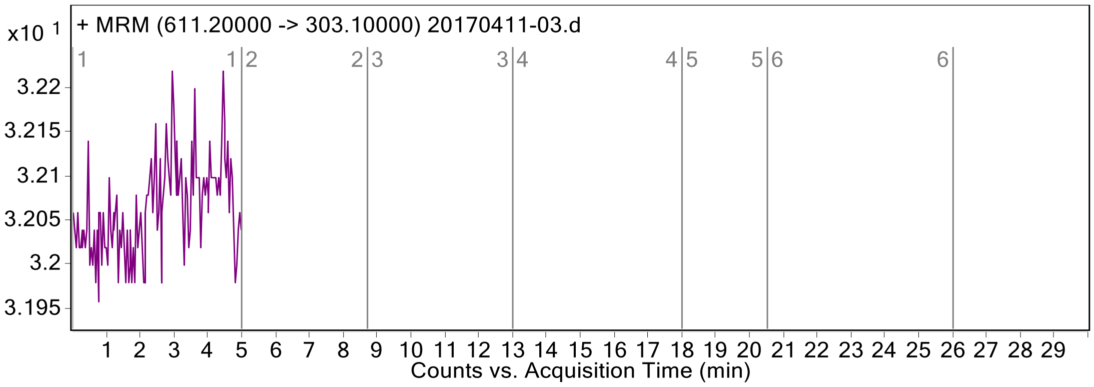
x10 1
+ MRM (611.20000 -> 303.10000) 20170411-03.d
3.22
3.215
3.21
3.205
3.2
3.195
1 2 3 4 5 6 7 8 9 10 11 12 13 14 15 16 17 18 19 20 21 22 23 24 25 26 27 28 29
Counts vs. Acquisition Time (min)

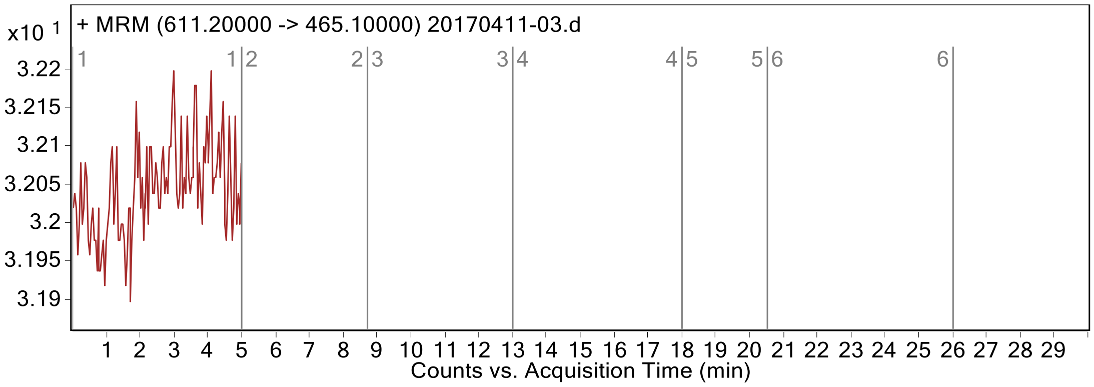
x10 1
+ MRM (611.20000 -> 465.10000) 20170411-03.d
3.22
3.215
3.21
3.205
3.2
3.195
3.19
1 2 3 4 5 6 7 8 9 10 11 12 13 14 15 16 17 18 19 20 21 22 23 24 25 26 27 28 29
Counts vs. Acquisition Time (min)

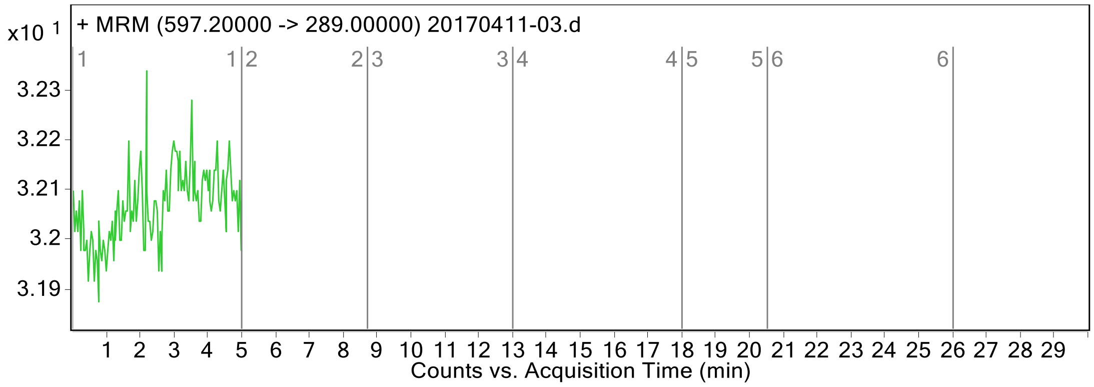
x10 1
+ MRM (597.20000 -> 289.00000) 20170411-03.d
3.23
3.22
3.21
3.2
3.19
1 2 3 4 5 6 7 8 9 10 11 12 13 14 15 16 17 18 19 20 21 22 23 24 25 26 27 28 29
Counts vs. Acquisition Time (min)

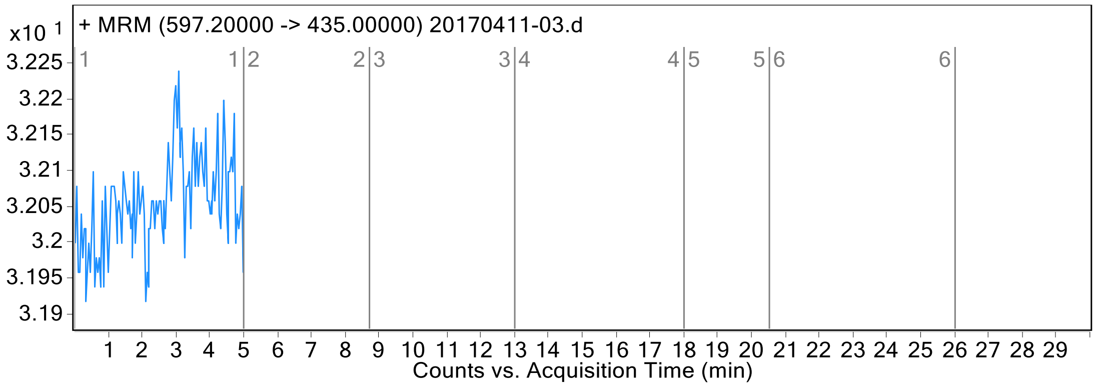
x10 1
+ MRM (597.20000 -> 435.00000) 20170411-03.d
3.225
3.22
3.215
3.21
3.205
3.2
3.195
3.19
1 2 3 4 5 6 7 8 9 10 11 12 13 14 15 16 17 18 19 20 21 22 23 24 25 26 27 28 29
Counts vs. Acquisition Time (min)

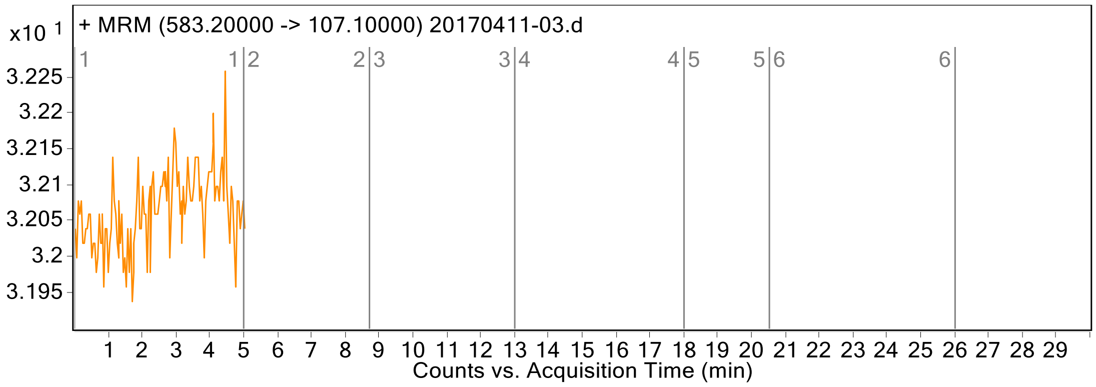
x10 1
+ MRM (583.20000 -> 107.10000) 20170411-03.d
3.225
3.22
3.215
3.21
3.205
3.2
3.195
1 2 3 4 5 6 7 8 9 10 11 12 13 14 15 16 17 18 19 20 21 22 23 24 25 26 27 28 29
Counts vs. Acquisition Time (min)

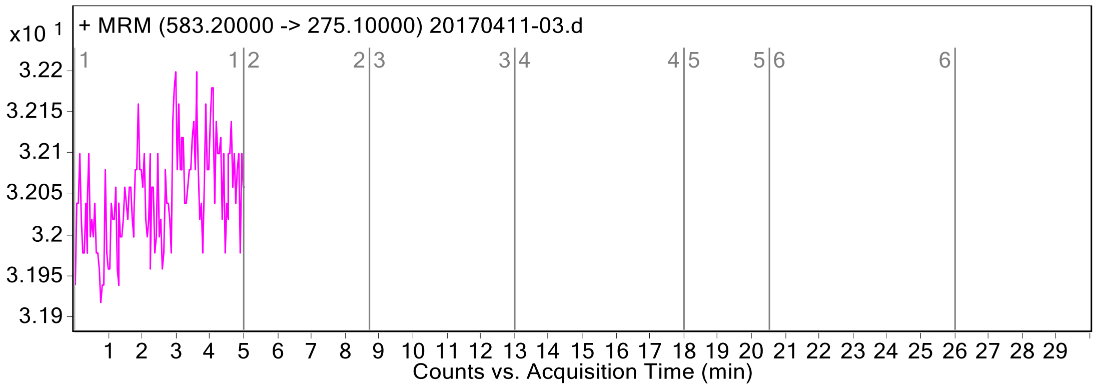
x10 1
+ MRM (583.20000 -> 275.10000) 20170411-03.d
3.22
3.215
3.21
3.205
3.2
3.195
3.19
1 2 3 4 5 6 7 8 9 10 11 12 13 14 15 16 17 18 19 20 21 22 23 24 25 26 27 28 29
Counts vs. Acquisition Time (min)

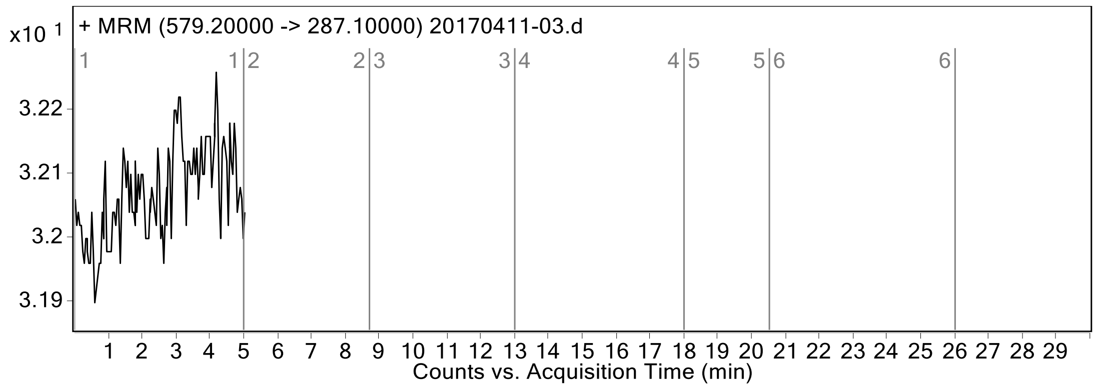
x10 1
+ MRM (579.20000 -> 287.10000) 20170411-03.d
3.22
3.21
3.2
3.19
1 2 3 4 5 6 7 8 9 10 11 12 13 14 15 16 17 18 19 20 21 22 23 24 25 26 27 28 29
Counts vs. Acquisition Time (min)

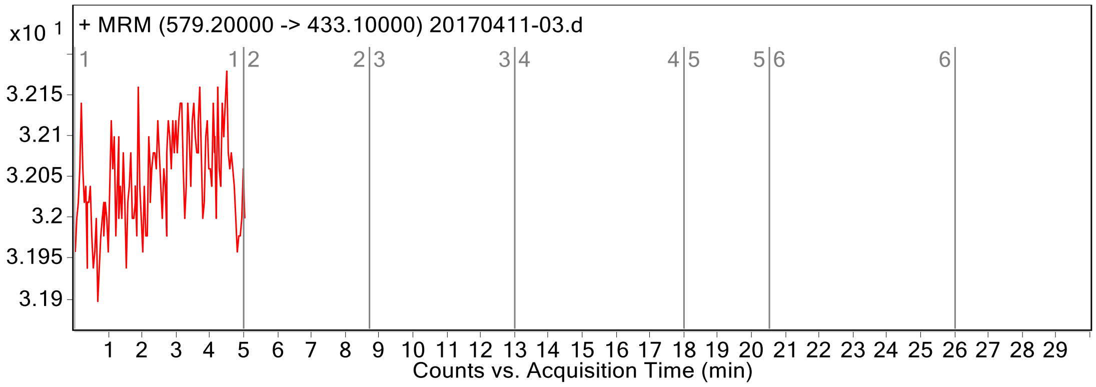
x10 1
+ MRM (579.20000 -> 433.10000) 20170411-03.d
3.215
3.21
3.205
3.2
3.195
3.19
1 2 3 4 5 6 7 8 9 10 11 12 13 14 15 16 17 18 19 20 21 22 23 24 25 26 27 28 29
Counts vs. Acquisition Time (min)

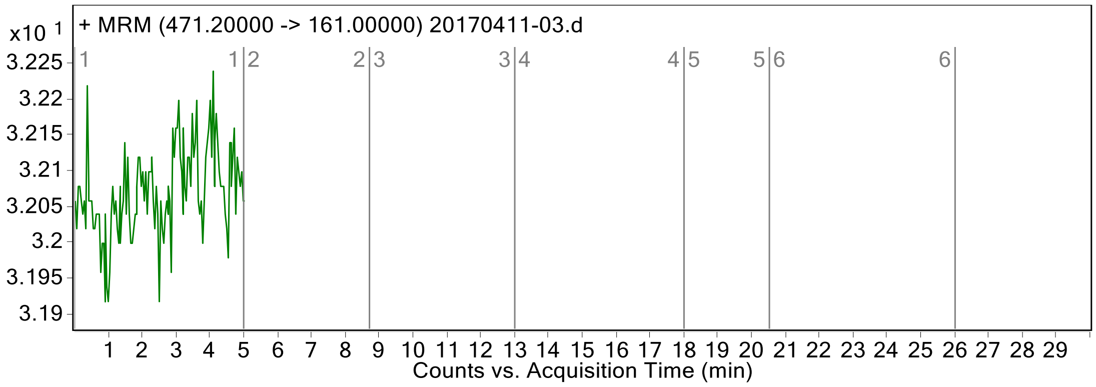
x10 1
+ MRM (471.20000 -> 161.00000) 20170411-03.d
3.225
3.22
3.215
3.21
3.205
3.2
3.195
3.19
1 2 3 4 5 6 7 8 9 10 11 12 13 14 15 16 17 18 19 20 21 22 23 24 25 26 27 28 29
Counts vs. Acquisition Time (min)

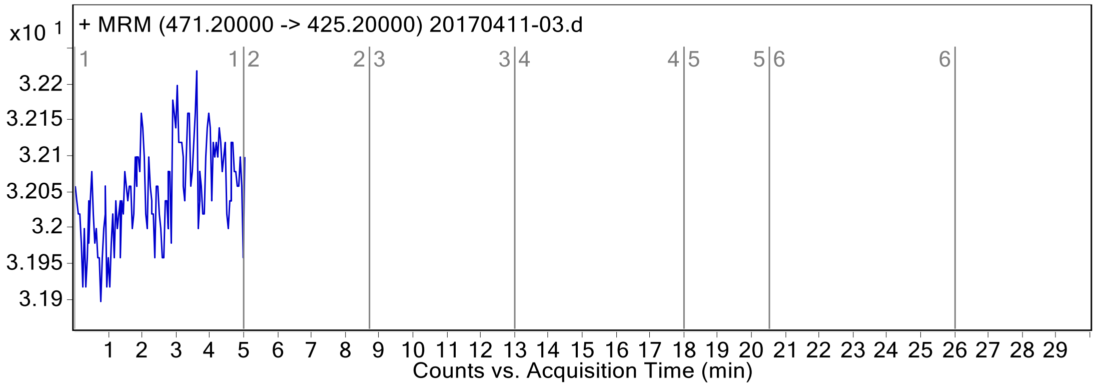
x10 1
+ MRM (471.20000 -> 425.20000) 20170411-03.d
3.22
3.215
3.21
3.205
3.2
3.195
3.19
1 2 3 4 5 6 7 8 9 10 11 12 13 14 15 16 17 18 19 20 21 22 23 24 25 26 27 28 29
Counts vs. Acquisition Time (min)

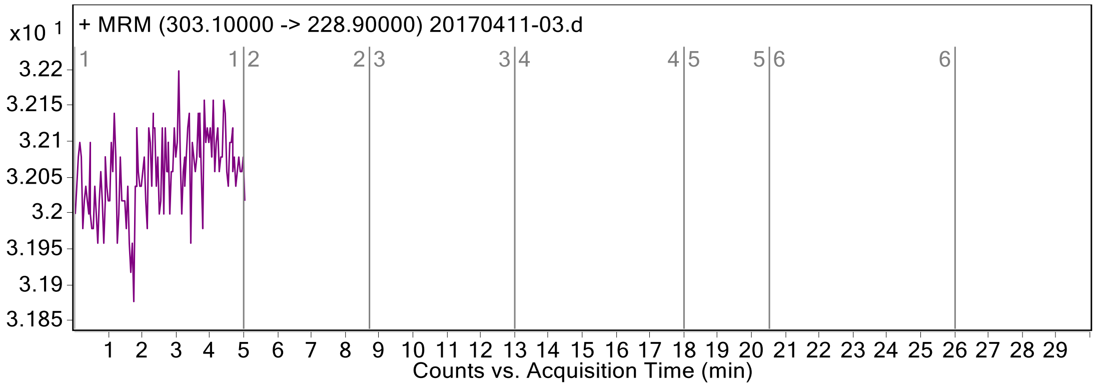
x10 1
+ MRM (303.10000 -> 228.90000) 20170411-03.d
3.22
3.215
3.21
3.205
3.2
3.195
3.19
3.185
1 2 3 4 5 6 7 8 9 10 11 12 13 14 15 16 17 18 19 20 21 22 23 24 25 26 27 28 29
Counts vs. Acquisition Time (min)

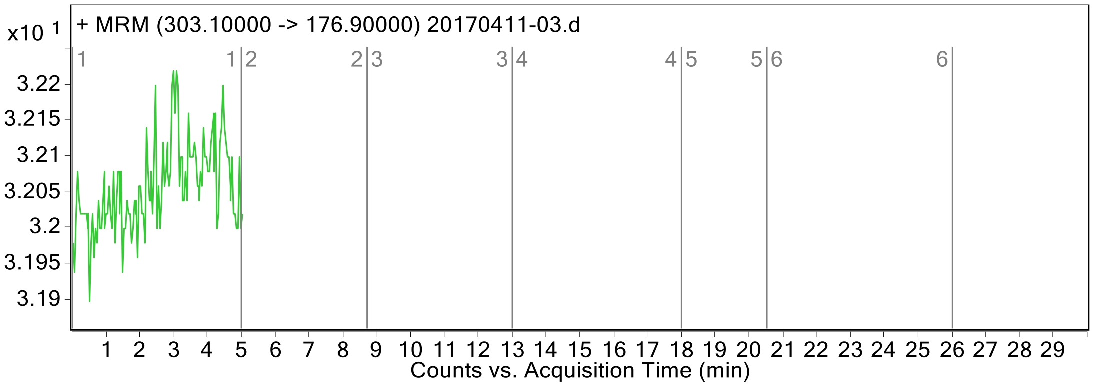
+ MRM (303.10000 -> 176.90000) 20170411-03.d
x10 1
Counts vs. Acquisition Time (min)

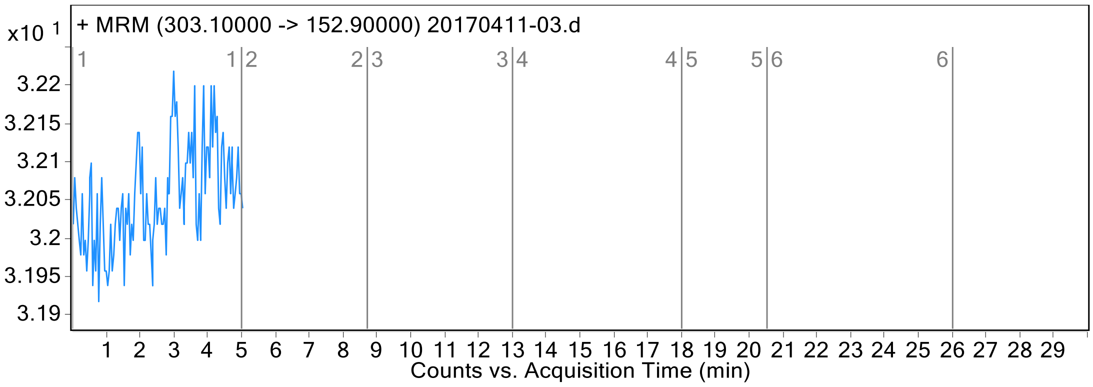
+ MRM (303.10000 -> 152.90000) 20170411-03.d
x10 1
Counts vs. Acquisition Time (min)

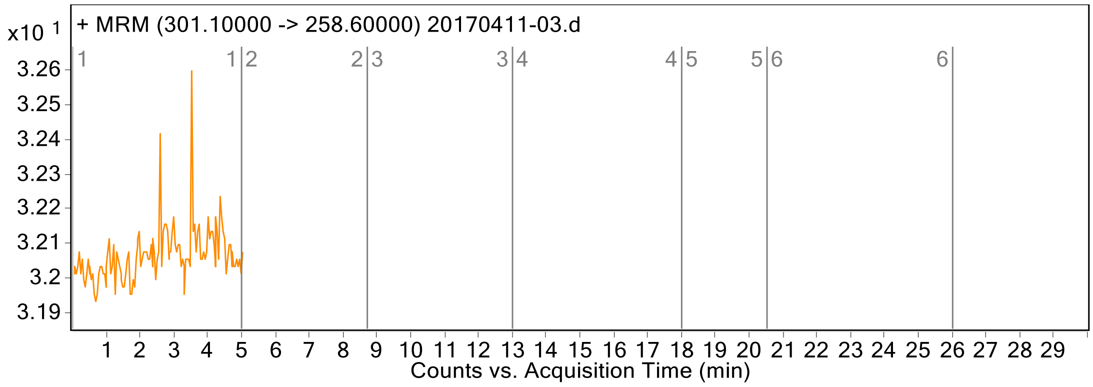
+ MRM (301.10000 -> 258.60000) 20170411-03.d
x10 1
Counts vs. Acquisition Time (min)

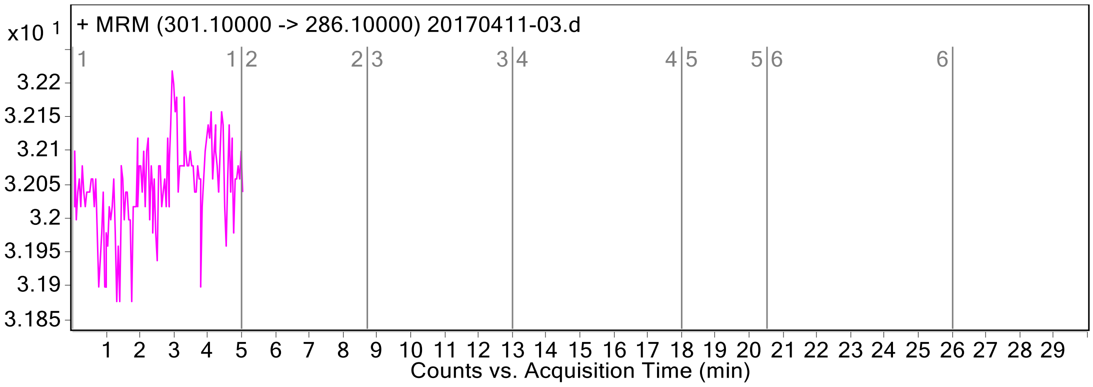
+ MRM (301.10000 -> 286.10000) 20170411-03.d
x10 1
Counts vs. Acquisition Time (min)

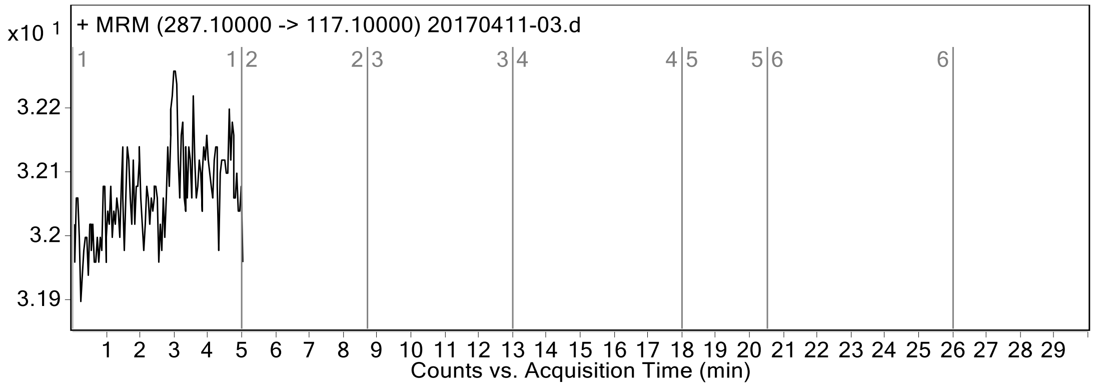
x10 1
+ MRM (287.10000 -> 117.10000) 20170411-03.d
Counts vs. Acquisition Time (min)

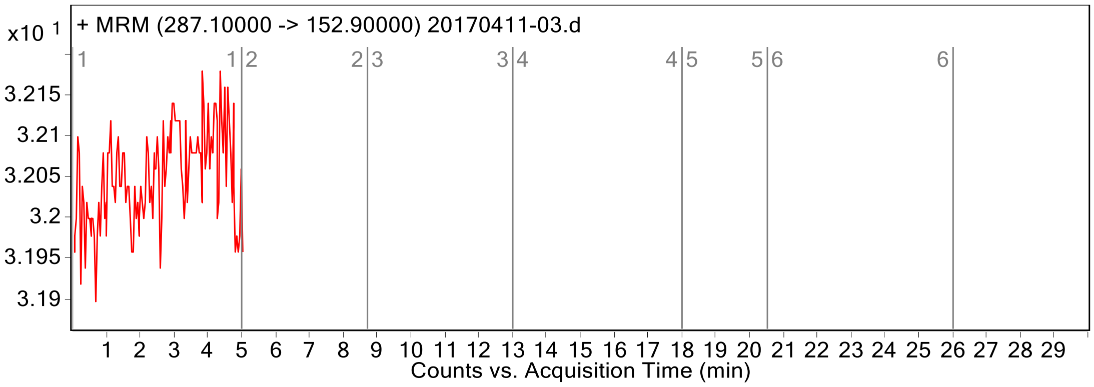
x10 1
+ MRM (287.10000 -> 152.90000) 20170411-03.d
Counts vs. Acquisition Time (min)

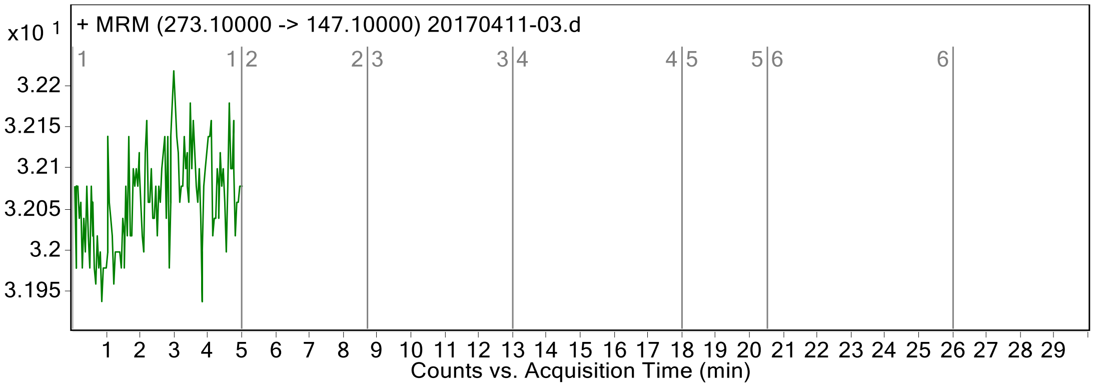
x10 1
+ MRM (273.10000 -> 147.10000) 20170411-03.d
Counts vs. Acquisition Time (min)

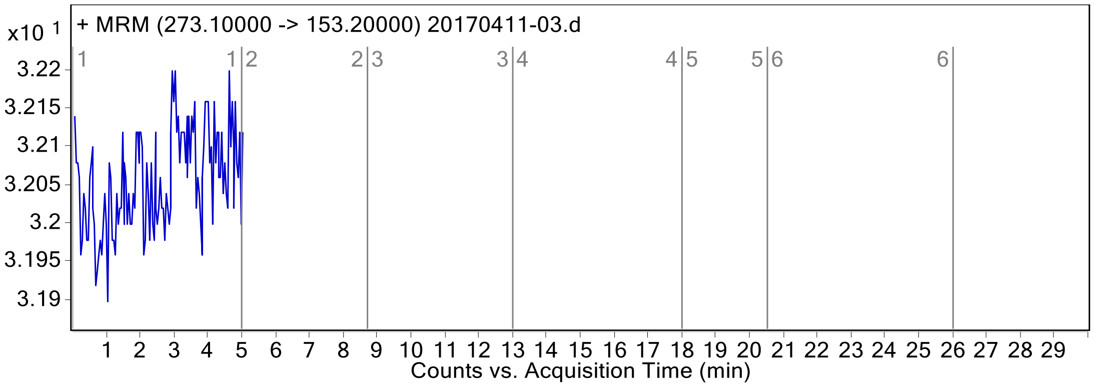
x10 1
+ MRM (273.10000 -> 153.20000) 20170411-03.d
Counts vs. Acquisition Time (min)

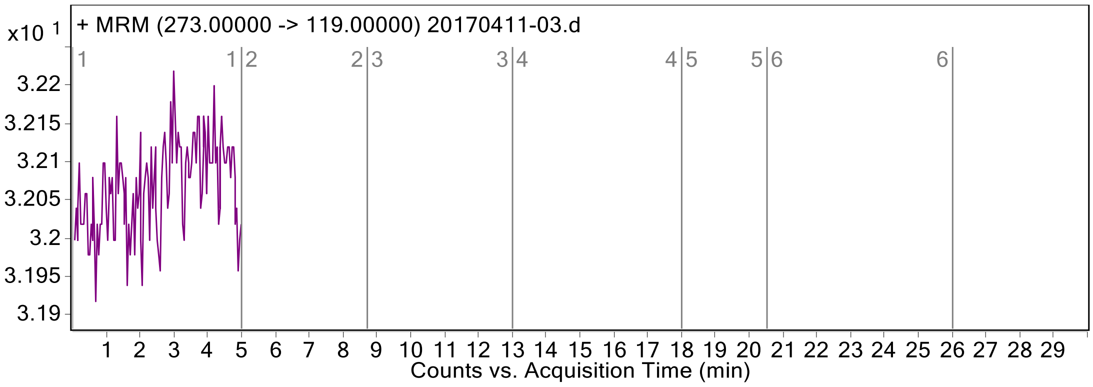
x10 1
+ MRM (273.00000 -> 119.00000) 20170411-03.d
3.22
3.215
3.21
3.205
3.2
3.195
3.19
1 2 3 4 5 6 7 8 9 10 11 12 13 14 15 16 17 18 19 20 21 22 23 24 25 26 27 28 29
Counts vs. Acquisition Time (min)

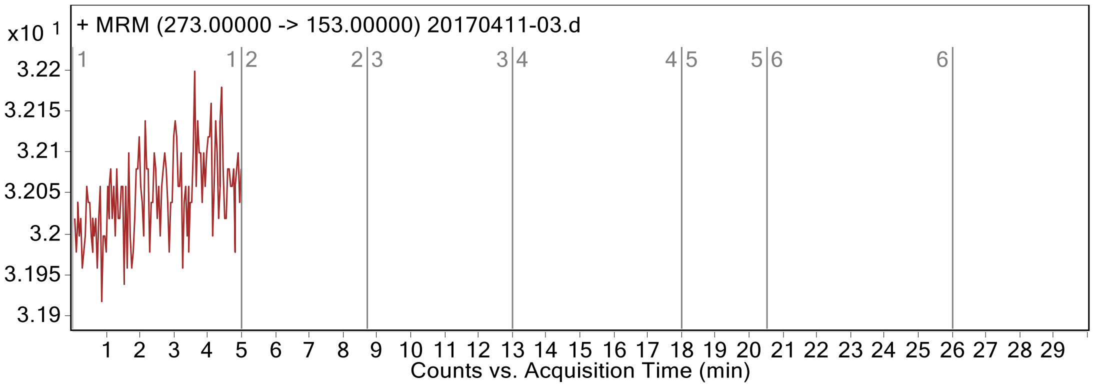
x10 1
+ MRM (273.00000 -> 153.00000) 20170411-03.d
3.22
3.215
3.21
3.205
3.2
3.195
3.19
1 2 3 4 5 6 7 8 9 10 11 12 13 14 15 16 17 18 19 20 21 22 23 24 25 26 27 28 29
Counts vs. Acquisition Time (min)

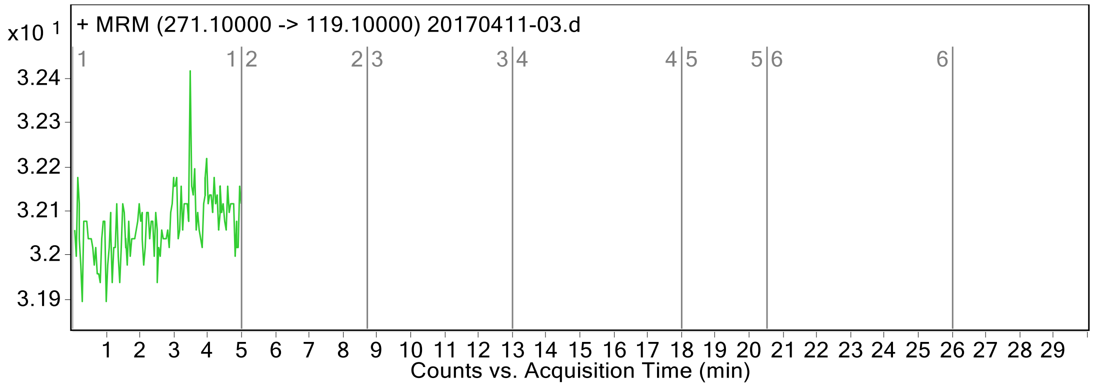
x10 1
+ MRM (271.10000 -> 119.10000) 20170411-03.d
3.24
3.23
3.22
3.21
3.2
3.19
1 2 3 4 5 6 7 8 9 10 11 12 13 14 15 16 17 18 19 20 21 22 23 24 25 26 27 28 29
Counts vs. Acquisition Time (min)

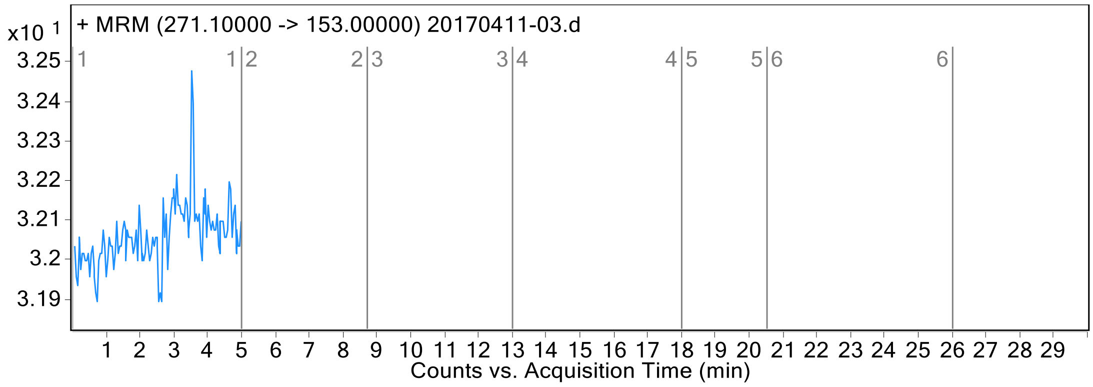
x10 1
+ MRM (271.10000 -> 153.00000) 20170411-03.d
3.25
3.24
3.23
3.22
3.21
3.2
3.19
1 2 3 4 5 6 7 8 9 10 11 12 13 14 15 16 17 18 19 20 21 22 23 24 25 26 27 28 29
Counts vs. Acquisition Time (min)

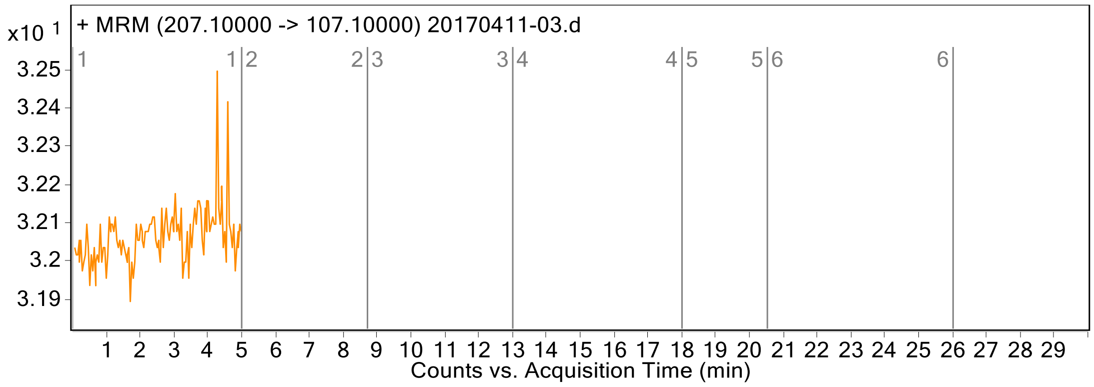
x10 1
+ MRM (207.10000 -> 107.10000) 20170411-03.d
Counts vs. Acquisition Time (min)

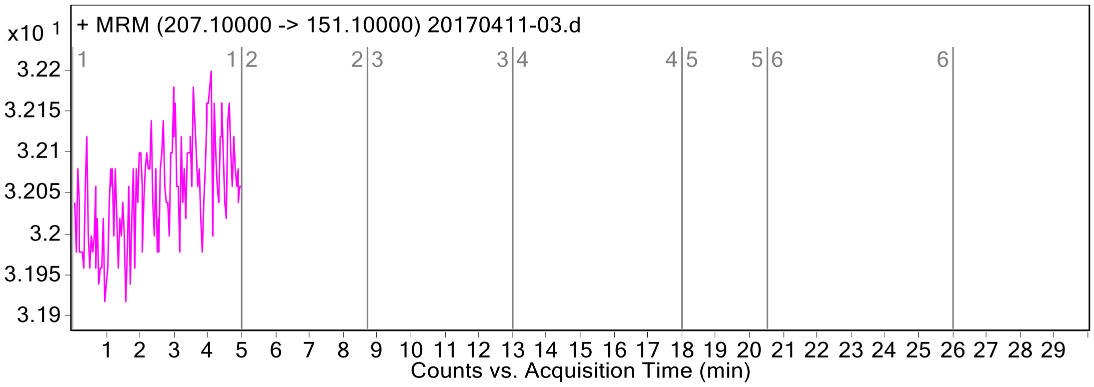
x10 1
+ MRM (207.10000 -> 151.10000) 20170411-03.d
Counts vs. Acquisition Time (min)

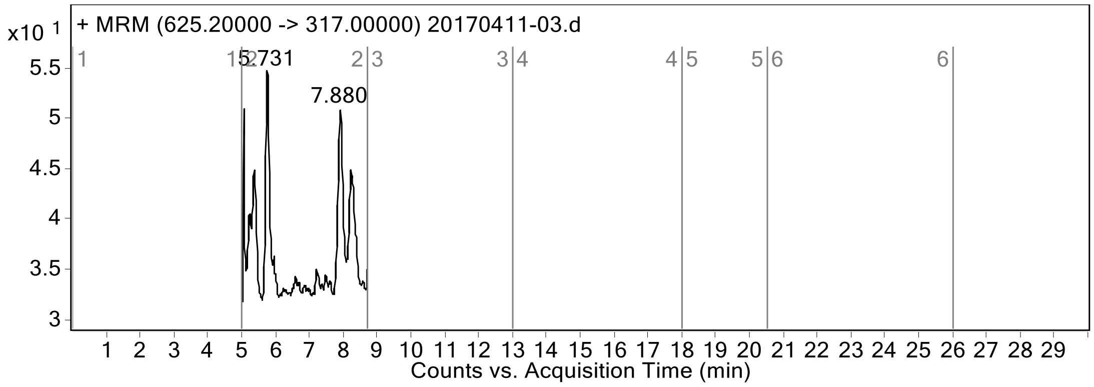
x10 1
+ MRM (625.20000 -> 317.00000) 20170411-03.d
5.731
7.880
Counts vs. Acquisition Time (min)

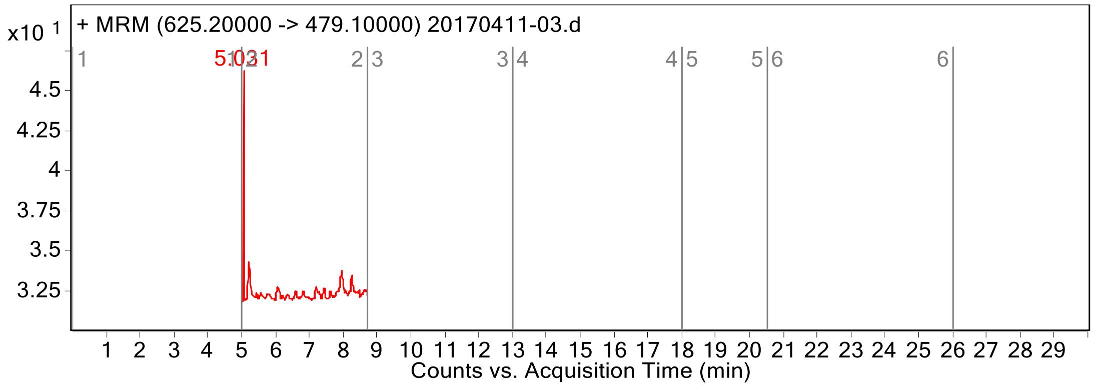
x10 1
+ MRM (625.20000 -> 479.10000) 20170411-03.d
5.031
Counts vs. Acquisition Time (min)

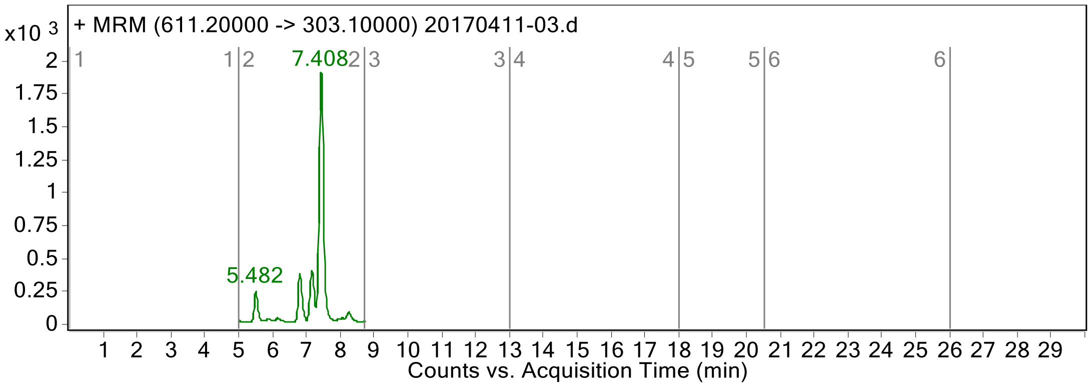
x10 3
+ MRM (611.20000 -> 303.10000) 20170411-03.d
7.408
5.482
Counts vs. Acquisition Time (min)

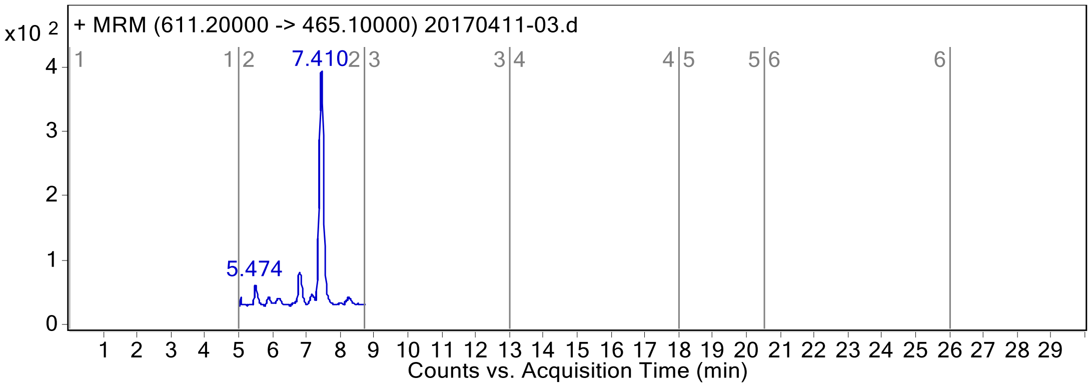
x10 2
+ MRM (611.20000 -> 465.10000) 20170411-03.d
7.410
5.474
Counts vs. Acquisition Time (min)

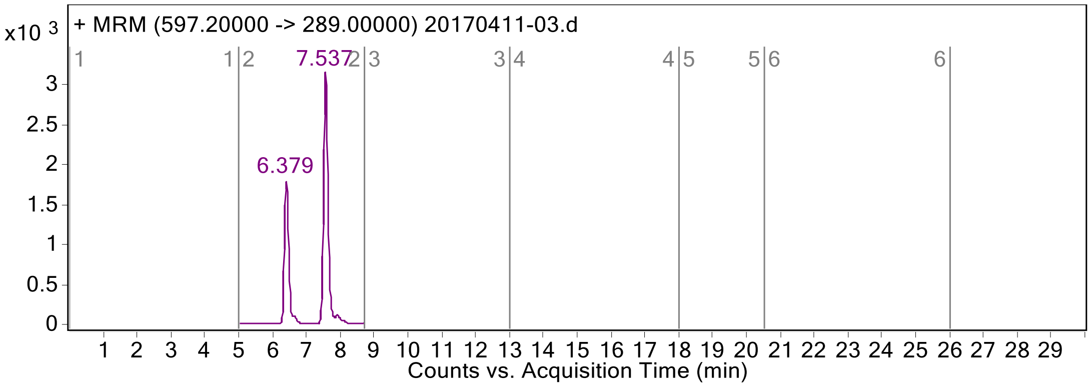
x10 3
+ MRM (597.20000 -> 289.00000) 20170411-03.d
7.537
6.379
Counts vs. Acquisition Time (min)

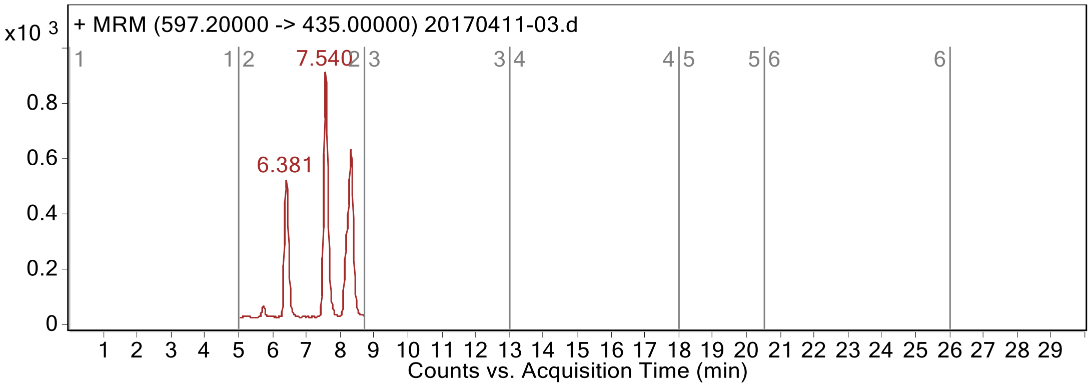
x10 3
+ MRM (597.20000 -> 435.00000) 20170411-03.d
7.540
6.381
Counts vs. Acquisition Time (min)

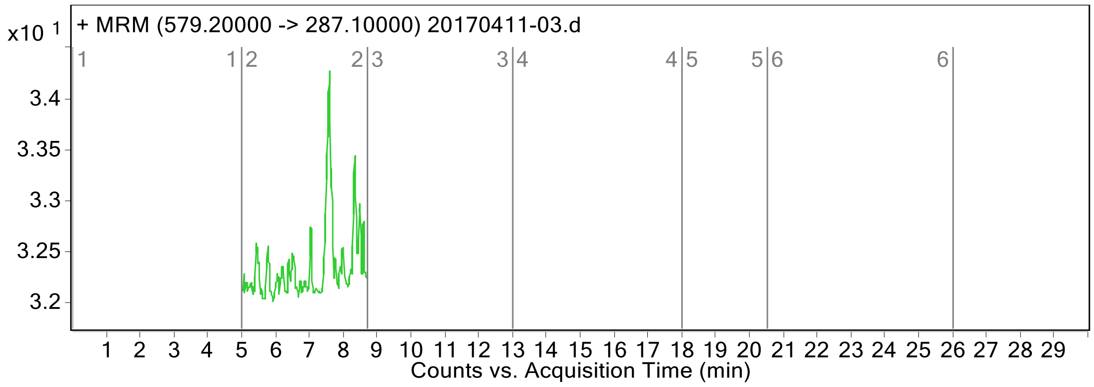
+ MRM (579.20000 -> 287.10000) 20170411-03.d
x10 1
Counts vs. Acquisition Time (min)

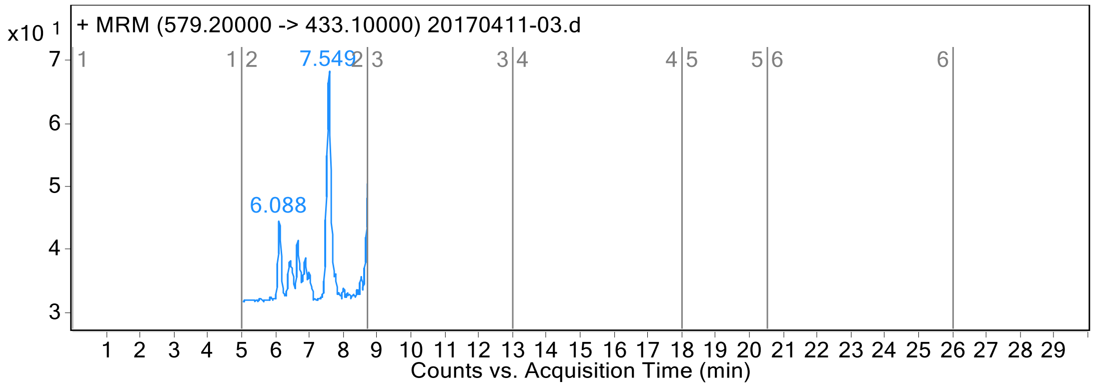
+ MRM (579.20000 -> 433.10000) 20170411-03.d
x10 1
7.549
6.088
Counts vs. Acquisition Time (min)

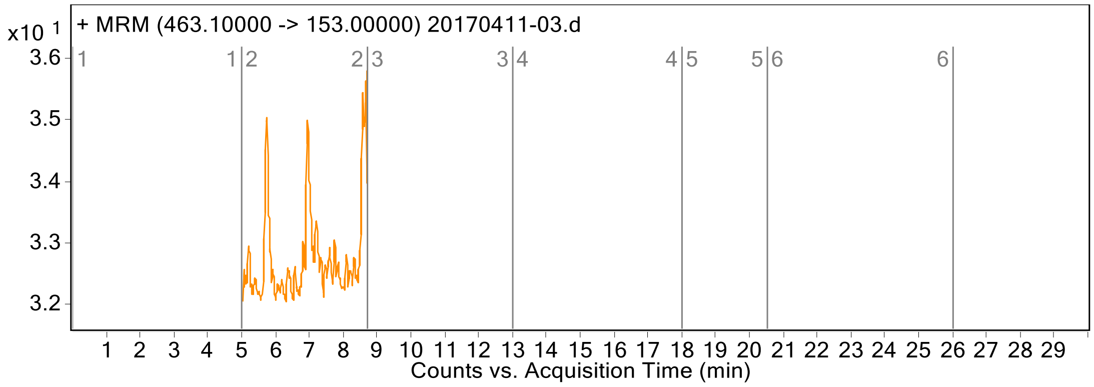
+ MRM (463.10000 -> 153.00000) 20170411-03.d
x10 1
Counts vs. Acquisition Time (min)

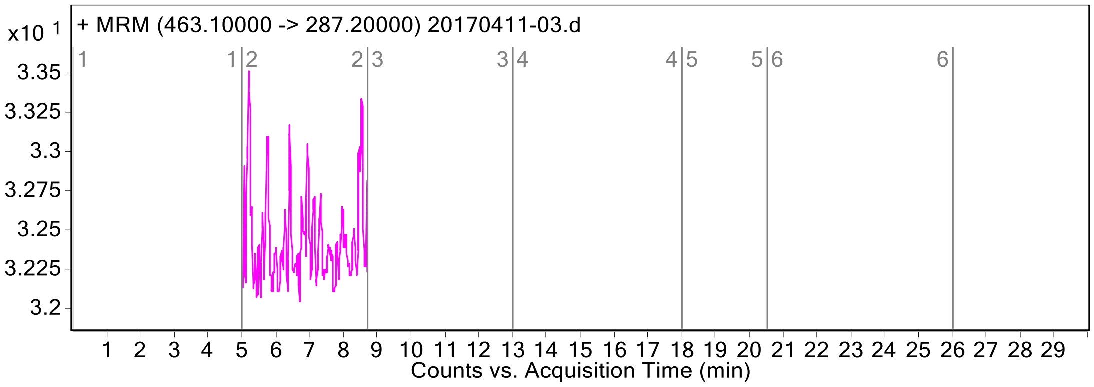
+ MRM (463.10000 -> 287.20000) 20170411-03.d
x10 1
Counts vs. Acquisition Time (min)

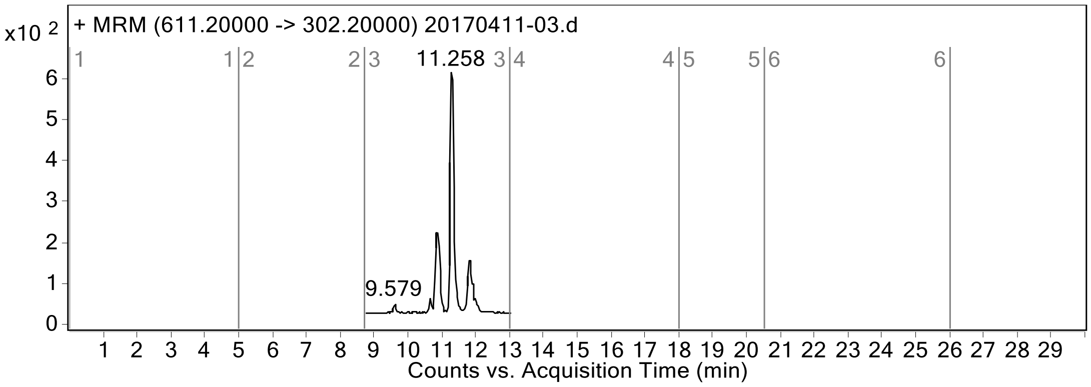

+ MRM (611.20000 -> 302.20000) 20170411-03.d
x10 2
11.258
9.579
Counts vs. Acquisition Time (min)

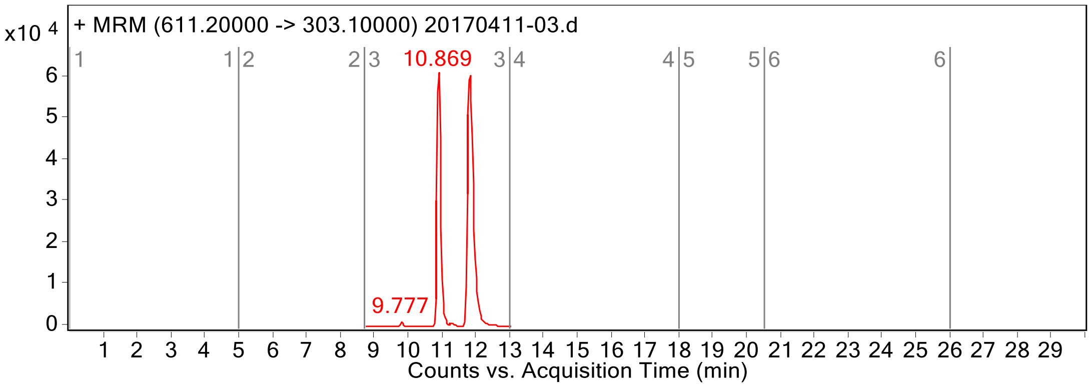

+ MRM (611.20000 -> 303.10000) 20170411-03.d
x10 4
10.869
9.777
Counts vs. Acquisition Time (min)

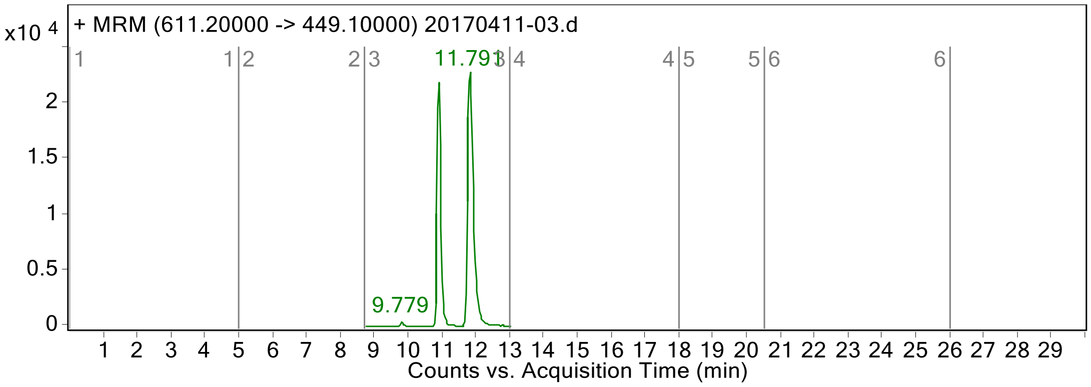

+ MRM (611.20000 -> 449.10000) 20170411-03.d
x10 4
11.793
9.779
Counts vs. Acquisition Time (min)

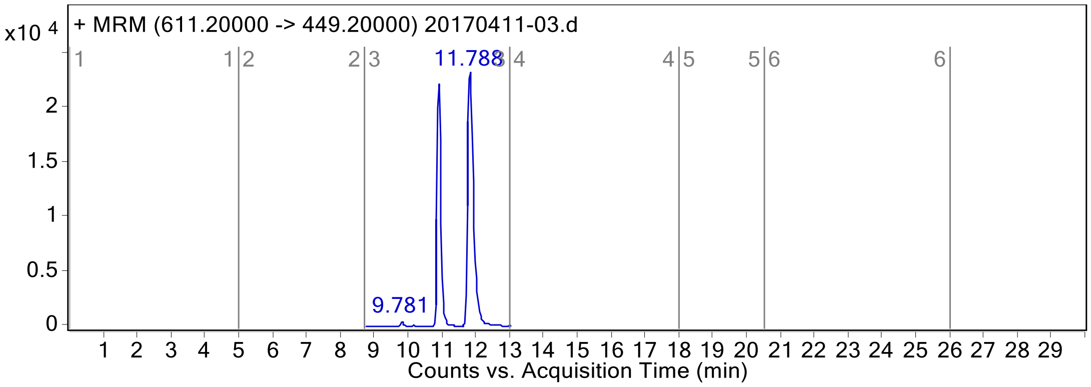

+ MRM (611.20000 -> 449.20000) 20170411-03.d
x10 4
11.788
9.781
Counts vs. Acquisition Time (min)

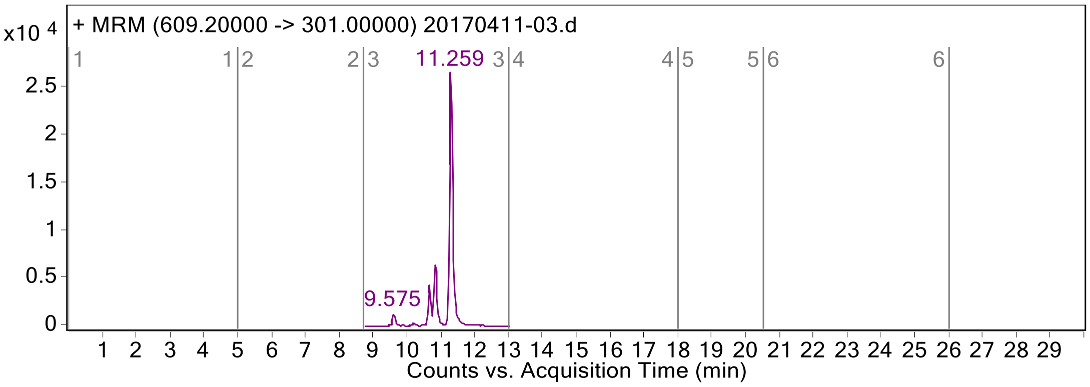
x10 4
+ MRM (609.20000 -> 301.00000) 20170411-03.d
11.259
9.575
Counts vs. Acquisition Time (min)

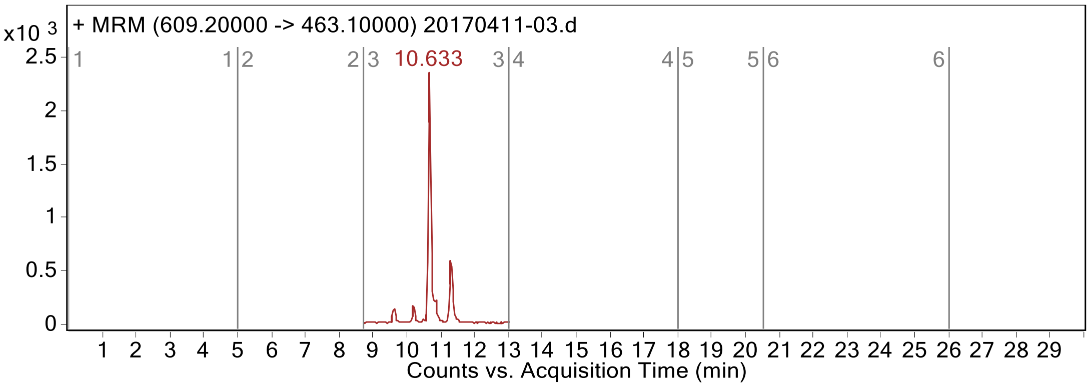
x10 3
+ MRM (609.20000 -> 463.10000) 20170411-03.d
10.633
Counts vs. Acquisition Time (min)

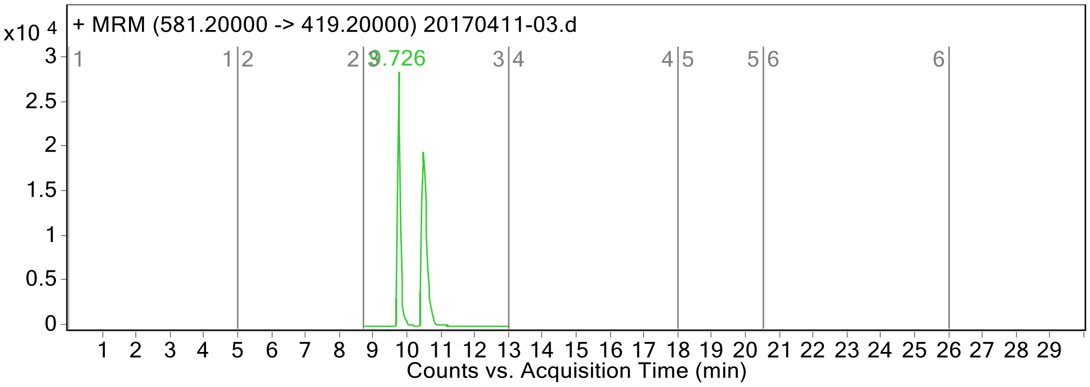
x10 4
+ MRM (581.20000 -> 419.20000) 20170411-03.d
9.726
Counts vs. Acquisition Time (min)

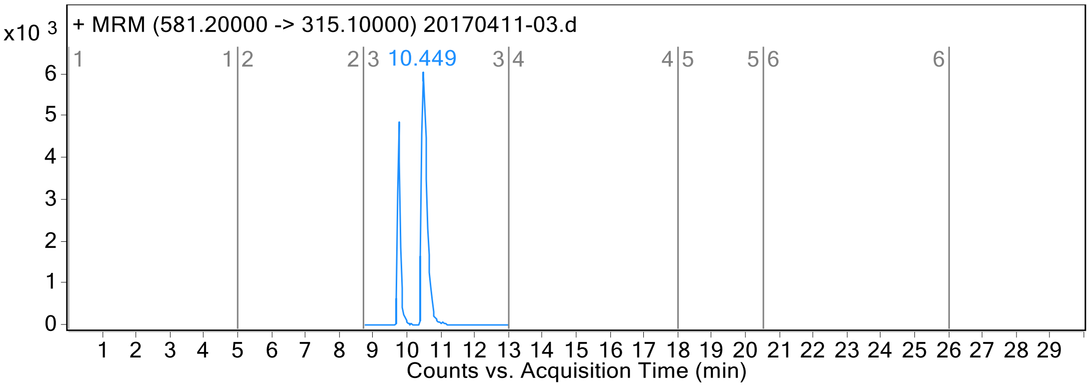
x10 3
+ MRM (581.20000 -> 315.10000) 20170411-03.d
10.449
Counts vs. Acquisition Time (min)

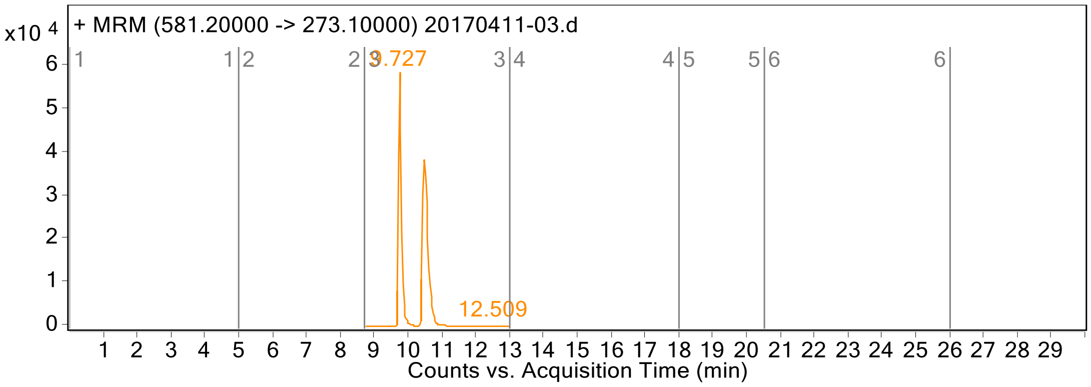
+ MRM (581.20000 -> 273.10000) 20170411-03.d
x10 4
9.727
12.509
Counts vs. Acquisition Time (min)

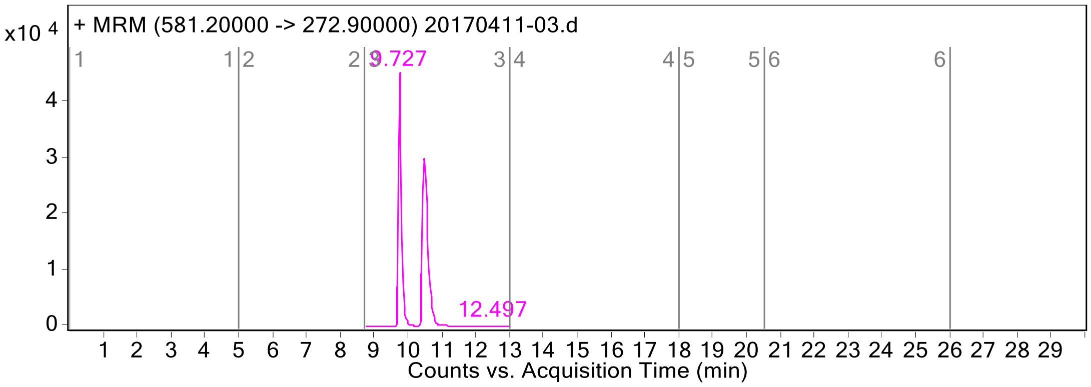
+ MRM (581.20000 -> 272.90000) 20170411-03.d
x10 4
9.727
12.497
Counts vs. Acquisition Time (min)

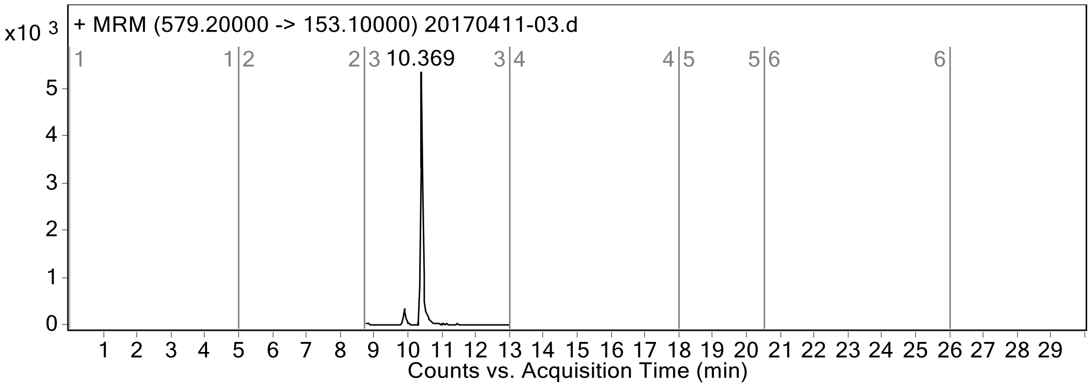
+ MRM (579.20000 -> 153.10000) 20170411-03.d
x10 3
10.369
Counts vs. Acquisition Time (min)

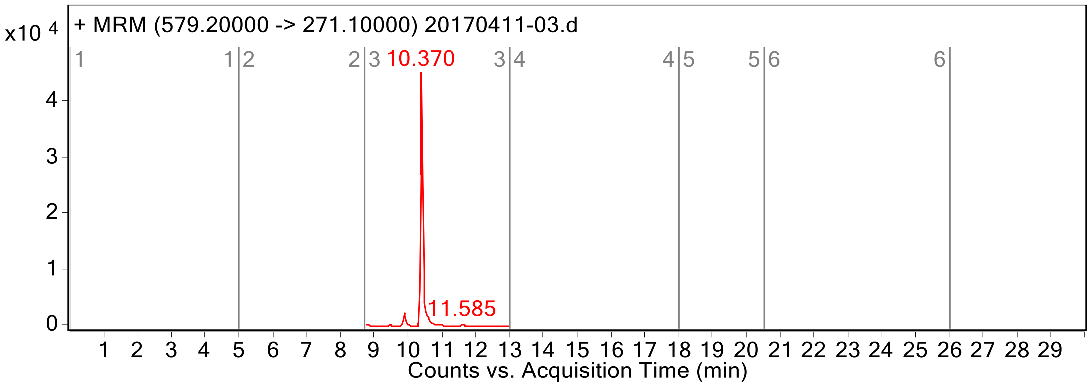
+ MRM (579.20000 -> 271.10000) 20170411-03.d
x10 4
10.370
11.585
Counts vs. Acquisition Time (min)

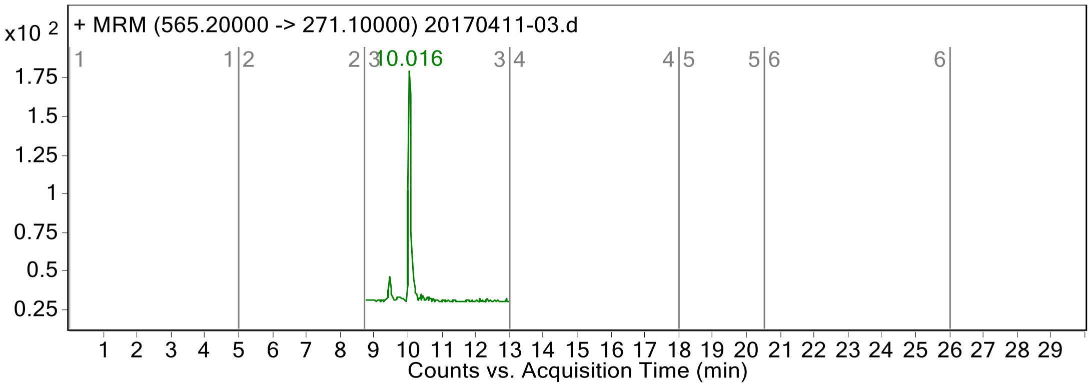

x10 2
+ MRM (565.20000 -> 271.10000) 20170411-03.d
10.016
Counts vs. Acquisition Time (min)

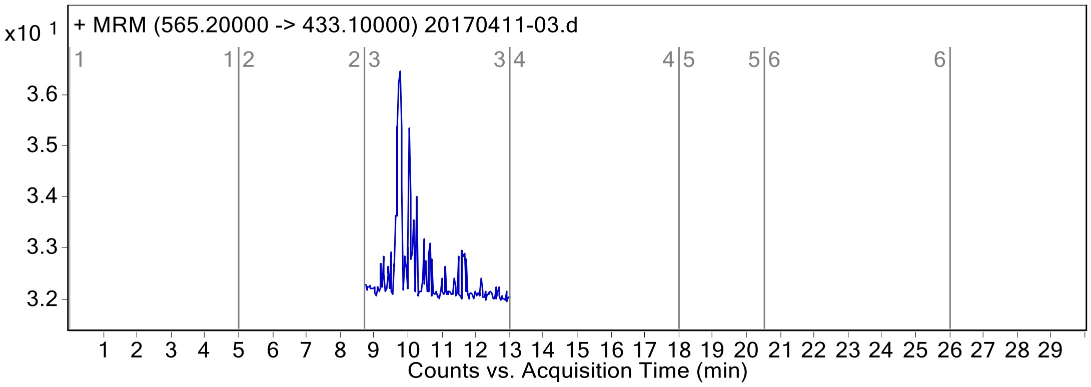

x10 1
+ MRM (565.20000 -> 433.10000) 20170411-03.d
Counts vs. Acquisition Time (min)

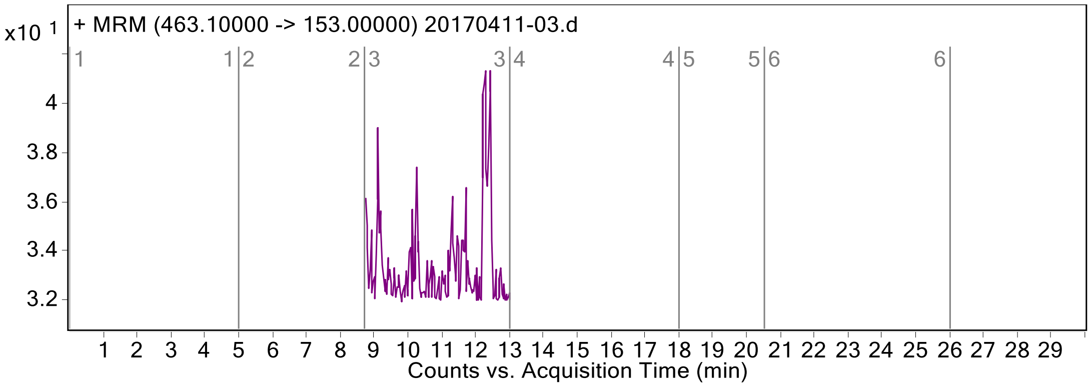

x10 1
+ MRM (463.10000 -> 153.00000) 20170411-03.d
Counts vs. Acquisition Time (min)

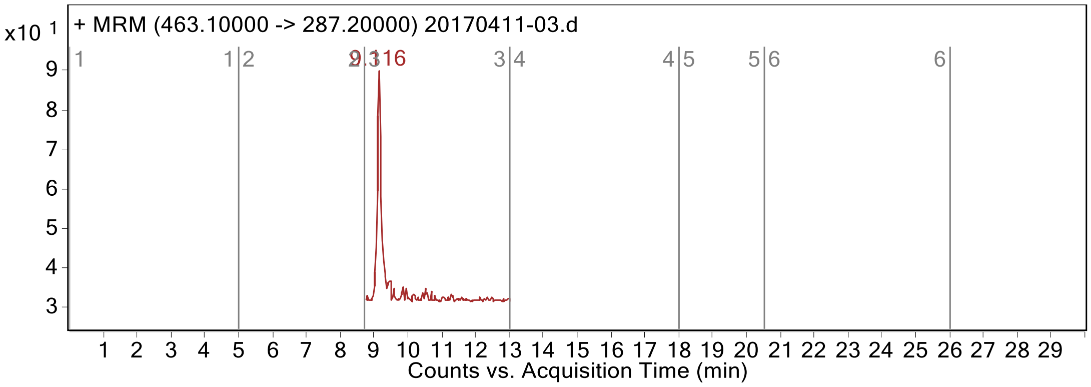

x10 1
+ MRM (463.10000 -> 287.20000) 20170411-03.d
9.316
Counts vs. Acquisition Time (min)

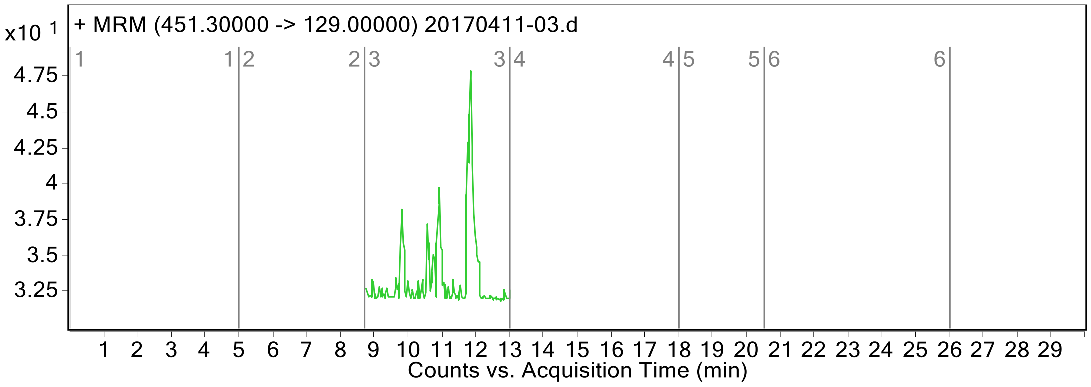
x10 1
+ MRM (451.30000 -> 129.00000) 20170411-03.d
4.75
4.5
4.25
4
3.75
3.5
3.25
1 2 3 4 5 6 7 8 9 10 11 12 13 14 15 16 17 18 19 20 21 22 23 24 25 26 27 28 29
Counts vs. Acquisition Time (min)

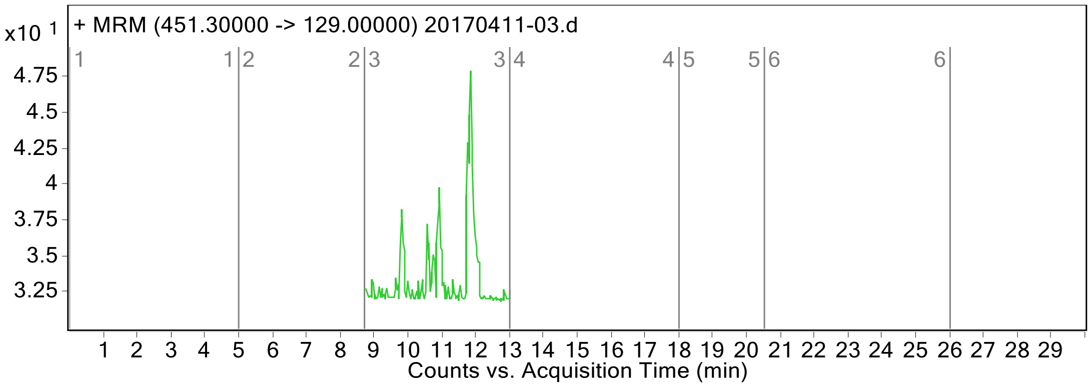
x10 1
+ MRM (451.30000 -> 129.00000) 20170411-03.d
4.75
4.5
4.25
4
3.75
3.5
3.25
1 2 3 4 5 6 7 8 9 10 11 12 13 14 15 16 17 18 19 20 21 22 23 24 25 26 27 28 29
Counts vs. Acquisition Time (min)

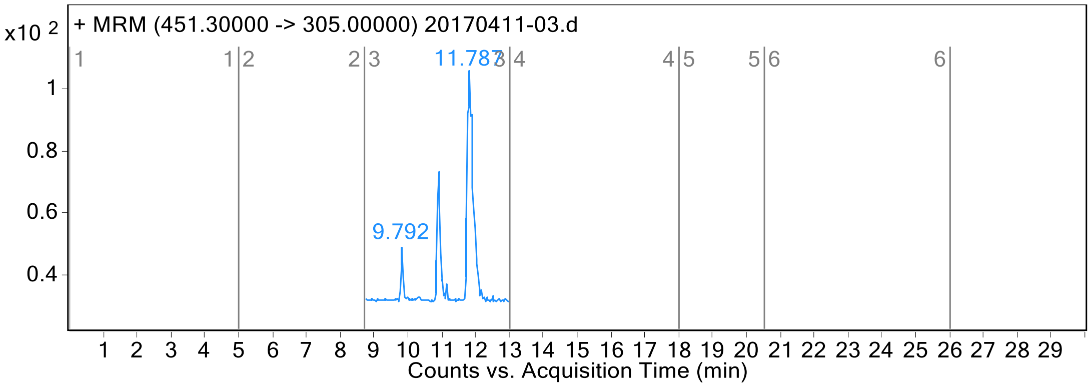
x10 2
+ MRM (451.30000 -> 305.00000) 20170411-03.d
11.787
9.792
1
0.8
0.6
0.4
1 2 3 4 5 6 7 8 9 10 11 12 13 14 15 16 17 18 19 20 21 22 23 24 25 26 27 28 29
Counts vs. Acquisition Time (min)

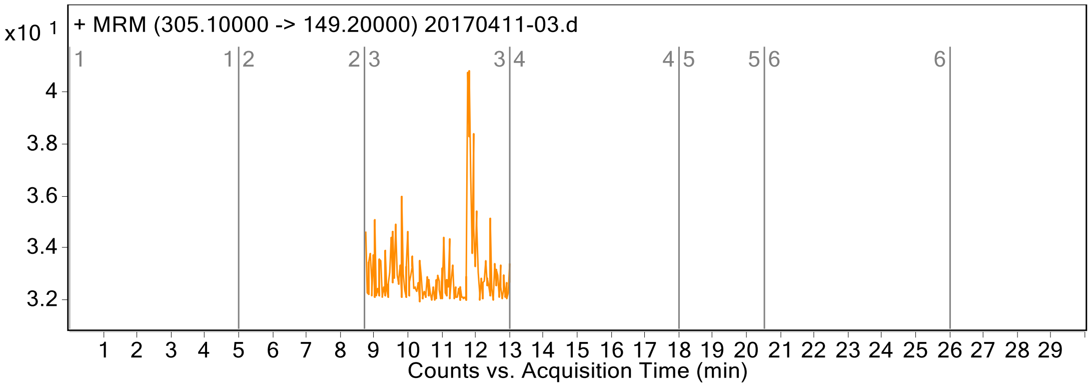
x10 1
+ MRM (305.10000 -> 149.20000) 20170411-03.d
4
3.8
3.6
3.4
3.2
1 2 3 4 5 6 7 8 9 10 11 12 13 14 15 16 17 18 19 20 21 22 23 24 25 26 27 28 29
Counts vs. Acquisition Time (min)

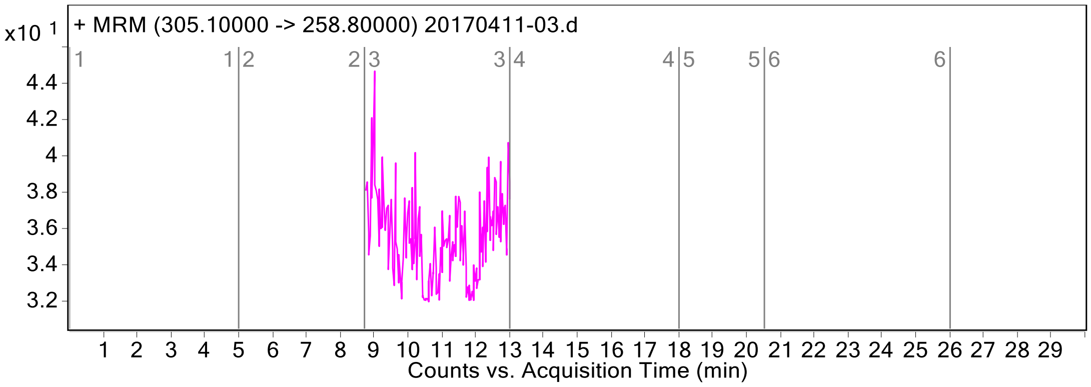
+ MRM (305.10000 -> 258.80000) 20170411-03.d
x10 1
Counts vs. Acquisition Time (min)

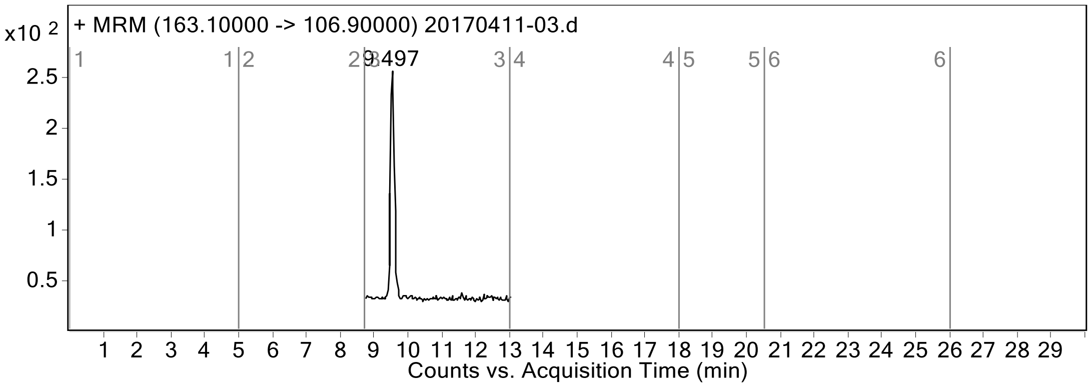
+ MRM (163.10000 -> 106.90000) 20170411-03.d
x10 2
9.497
Counts vs. Acquisition Time (min)

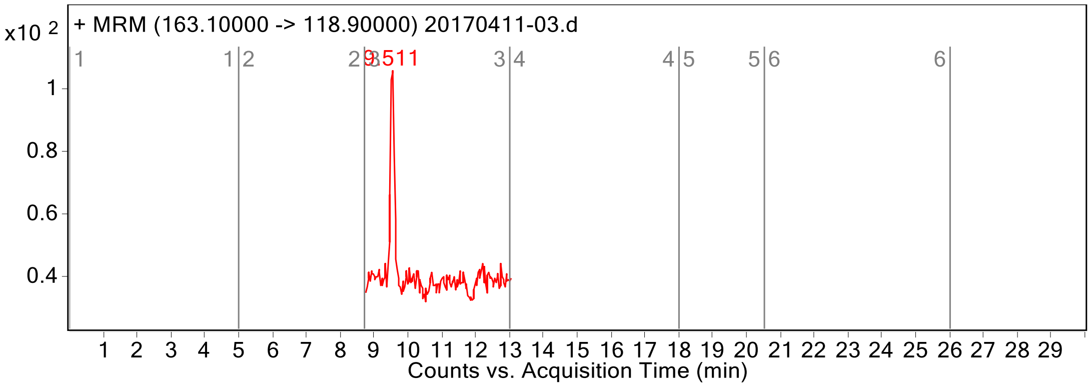
+ MRM (163.10000 -> 118.90000) 20170411-03.d
x10 2
9.511
Counts vs. Acquisition Time (min)

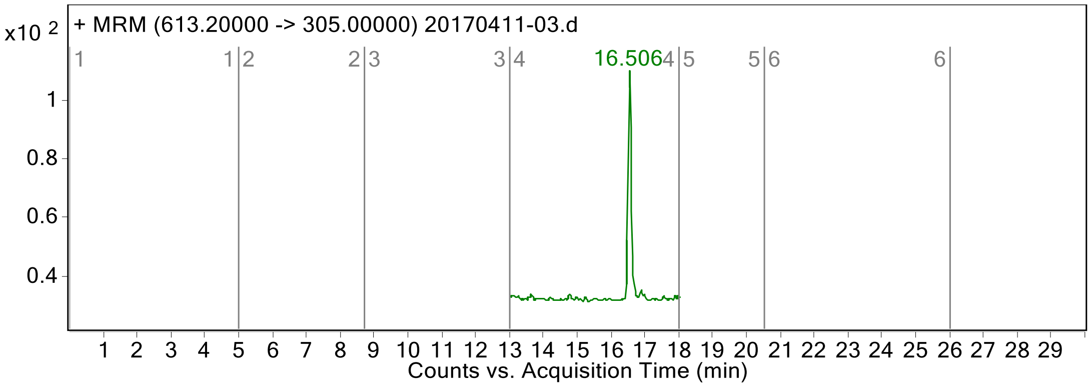
+ MRM (613.20000 -> 305.00000) 20170411-03.d
x10 2
16.506
Counts vs. Acquisition Time (min)

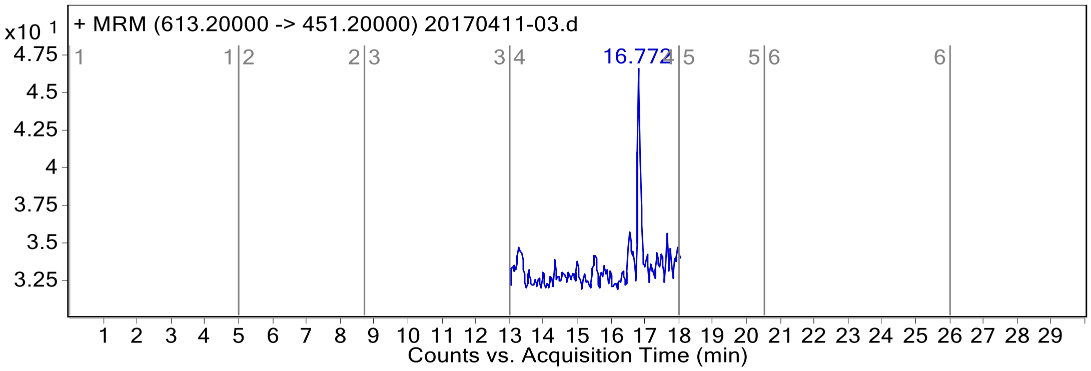
x10 1
+ MRM (613.20000 -> 451.20000) 20170411-03.d
16.772
Counts vs. Acquisition Time (min)

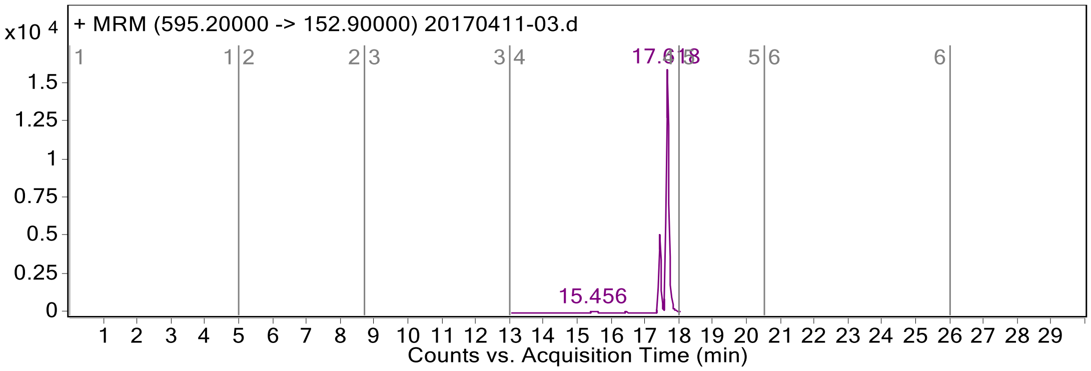
x10 4
+ MRM (595.20000 -> 152.90000) 20170411-03.d
17.618
15.456
Counts vs. Acquisition Time (min)

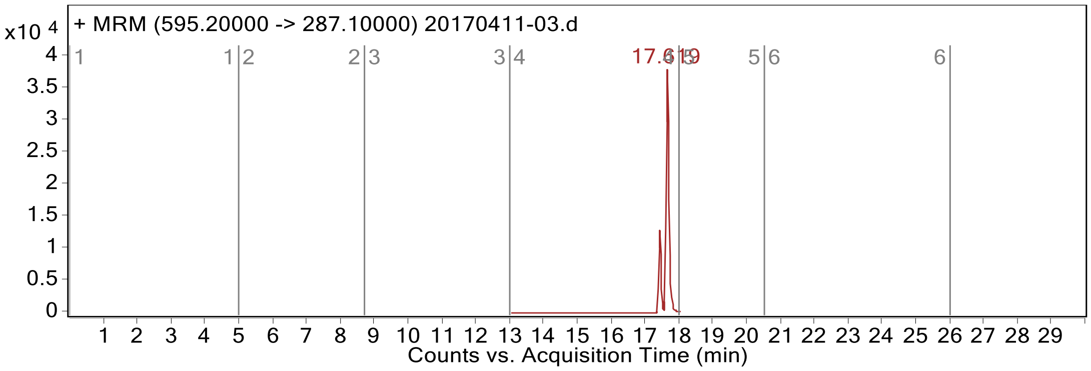
x10 4
+ MRM (595.20000 -> 287.10000) 20170411-03.d
17.619
Counts vs. Acquisition Time (min)

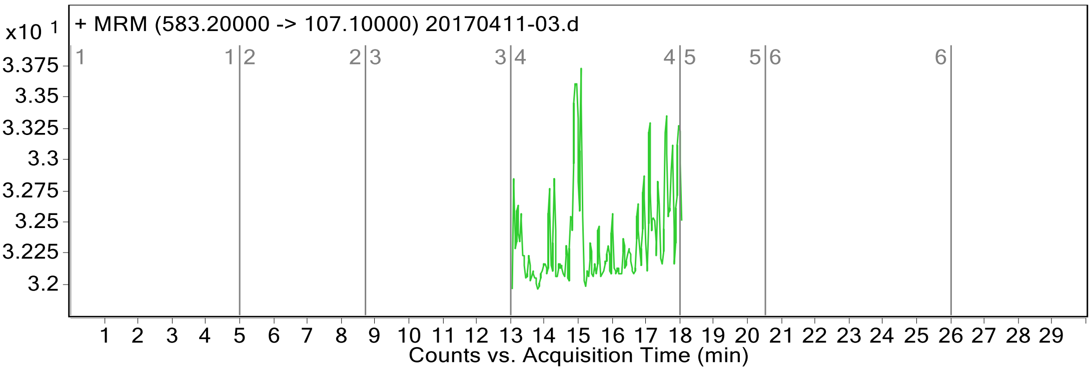
x10 1
+ MRM (583.20000 -> 107.10000) 20170411-03.d
Counts vs. Acquisition Time (min)

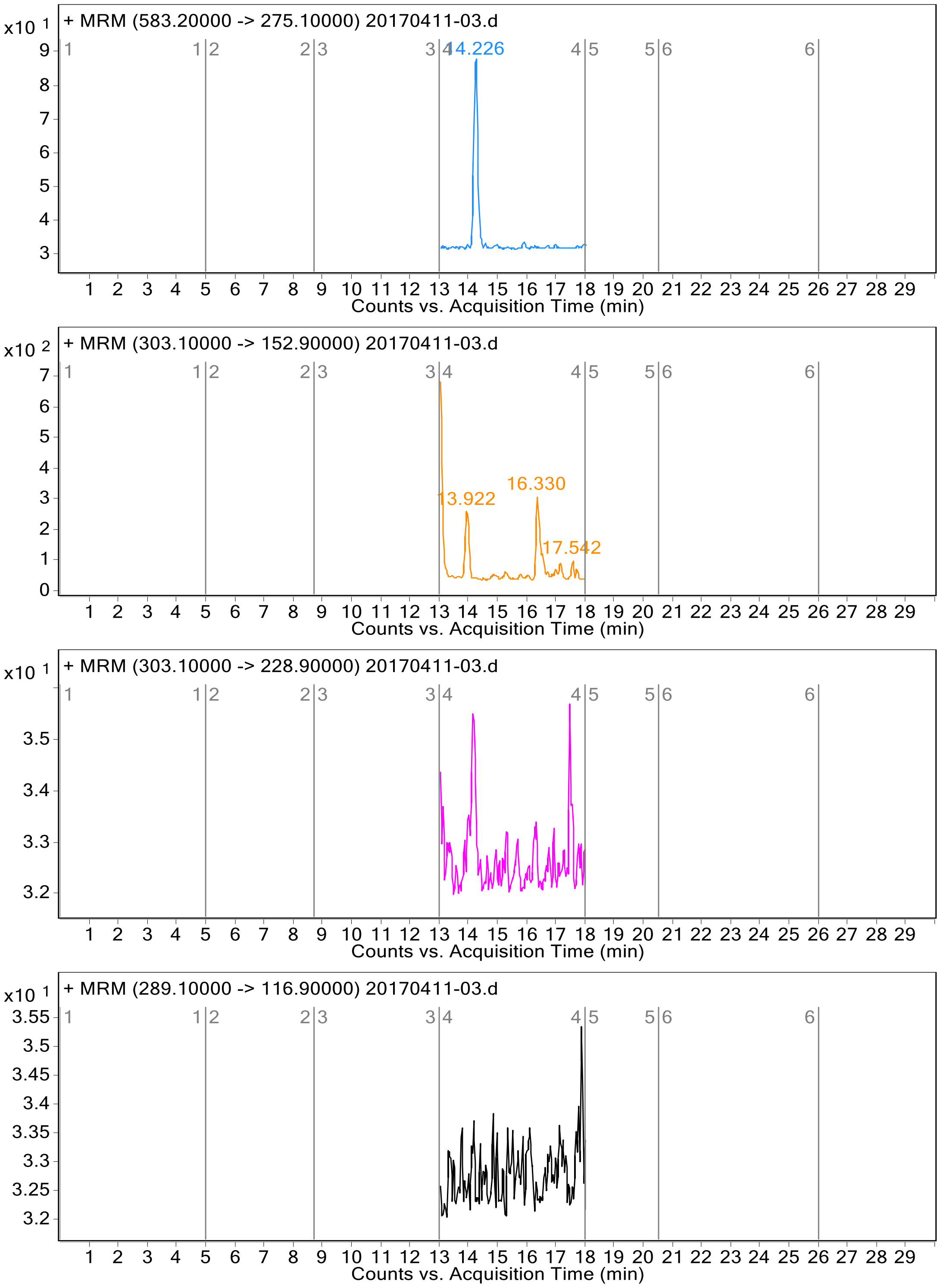

+ MRM (583.20000 -> 275.10000) 20170411-03.d
x10 1
14.226
Counts vs. Acquisition Time (min)
+ MRM (303.10000 -> 152.90000) 20170411-03.d
x10 2
13.922
16.330
17.542
Counts vs. Acquisition Time (min)
+ MRM (303.10000 -> 228.90000) 20170411-03.d
x10 1
Counts vs. Acquisition Time (min)
+ MRM (289.10000 -> 116.90000) 20170411-03.d
x10 1
Counts vs. Acquisition Time (min)

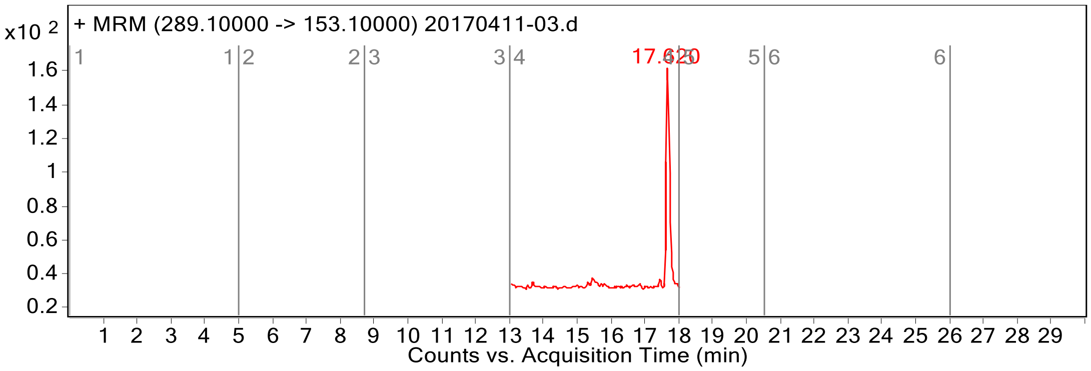
x10 2
+ MRM (289.10000 -> 153.10000) 20170411-03.d
17.620
Counts vs. Acquisition Time (min)

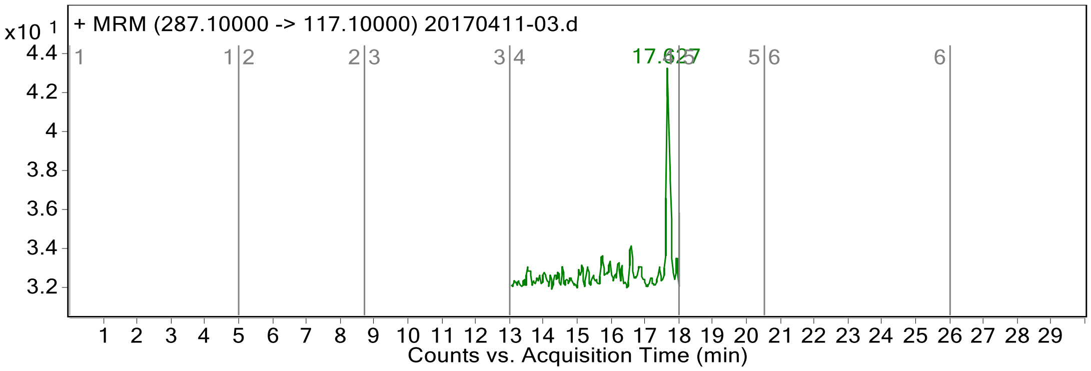
x10 1
+ MRM (287.10000 -> 117.10000) 20170411-03.d
17.627
Counts vs. Acquisition Time (min)

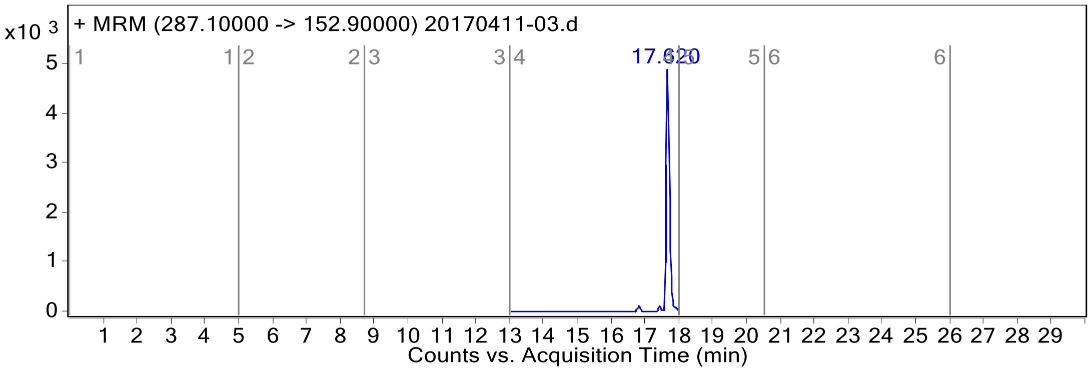
x10 3
+ MRM (287.10000 -> 152.90000) 20170411-03.d
17.620
Counts vs. Acquisition Time (min)

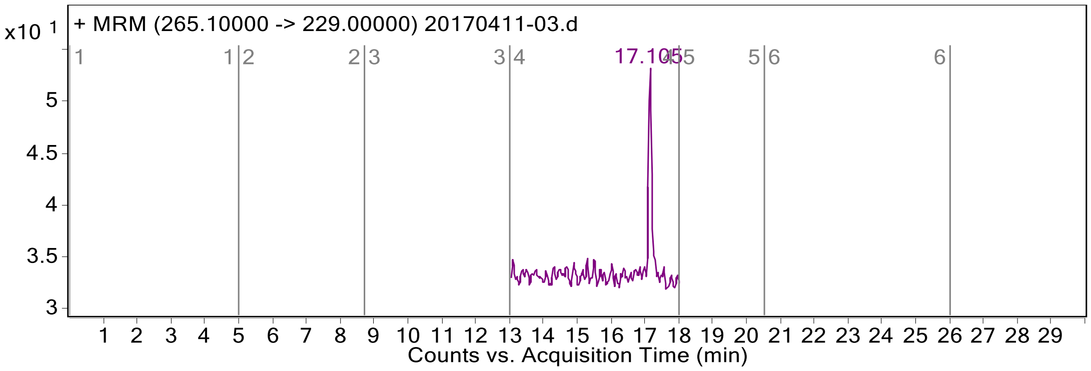
x10 1
+ MRM (265.10000 -> 229.00000) 20170411-03.d
17.105
Counts vs. Acquisition Time (min)

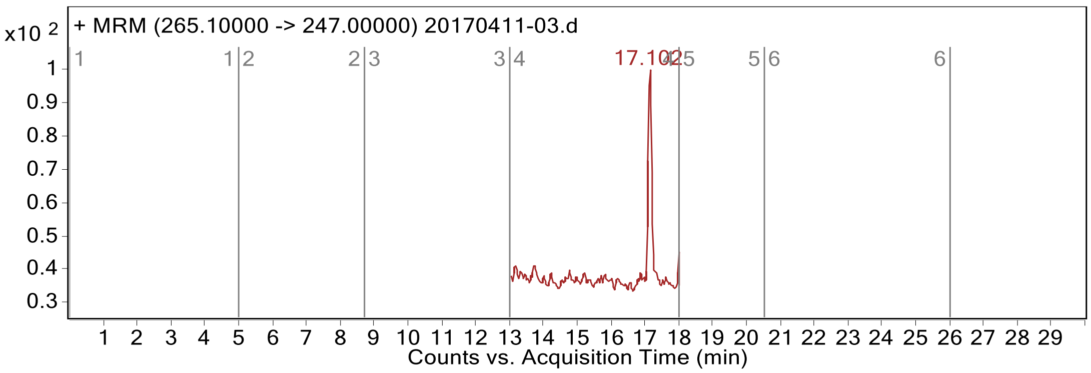
+ MRM (265.10000 -> 247.00000) 20170411-03.d
x10 2
17.102
Counts vs. Acquisition Time (min)

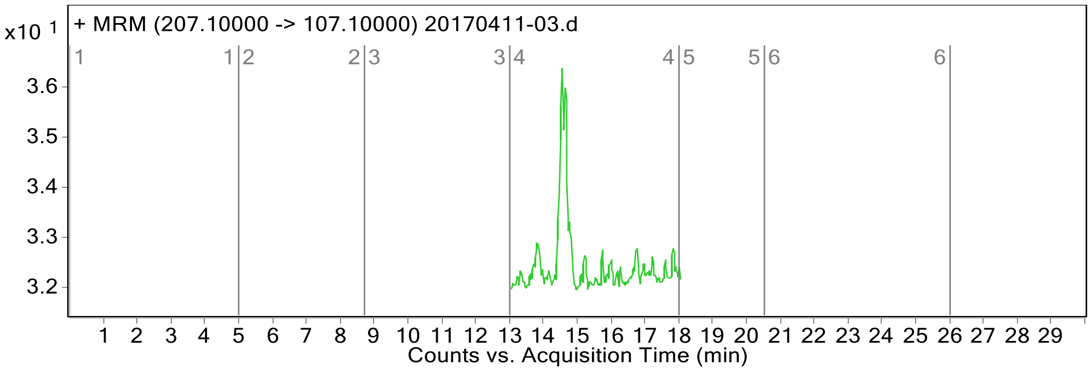
+ MRM (207.10000 -> 107.10000) 20170411-03.d
x10 1
Counts vs. Acquisition Time (min)

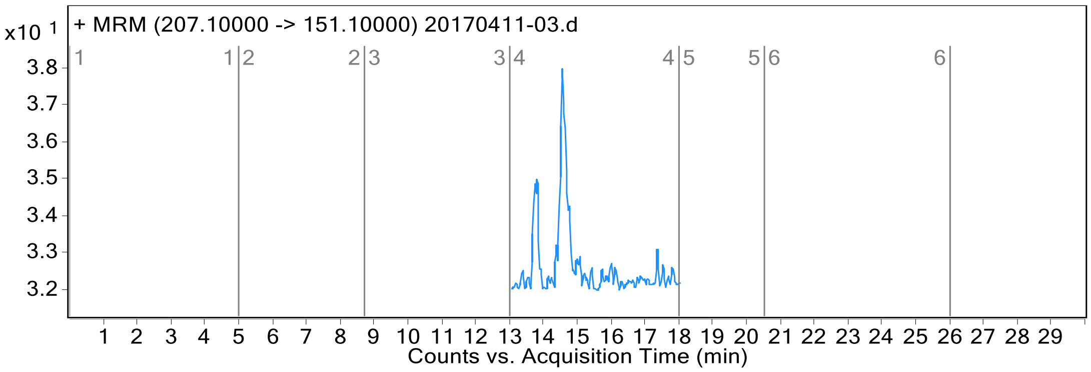
+ MRM (207.10000 -> 151.10000) 20170411-03.d
x10 1
Counts vs. Acquisition Time (min)

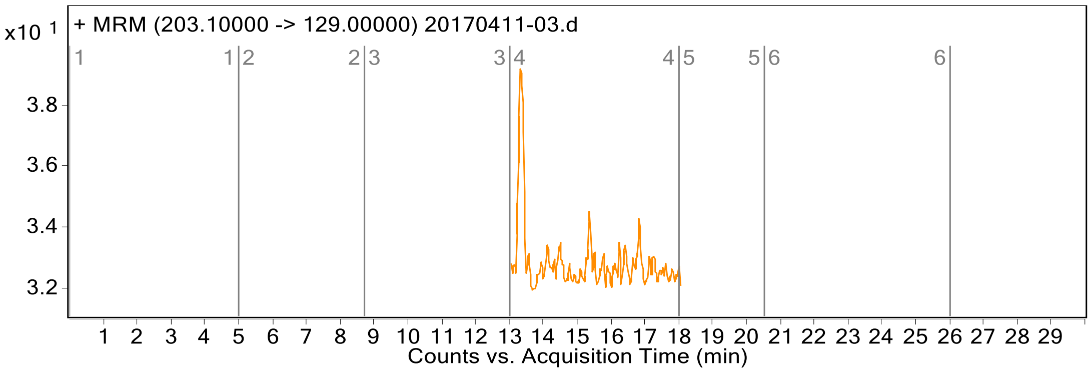
+ MRM (203.10000 -> 129.00000) 20170411-03.d
x10 1
Counts vs. Acquisition Time (min)

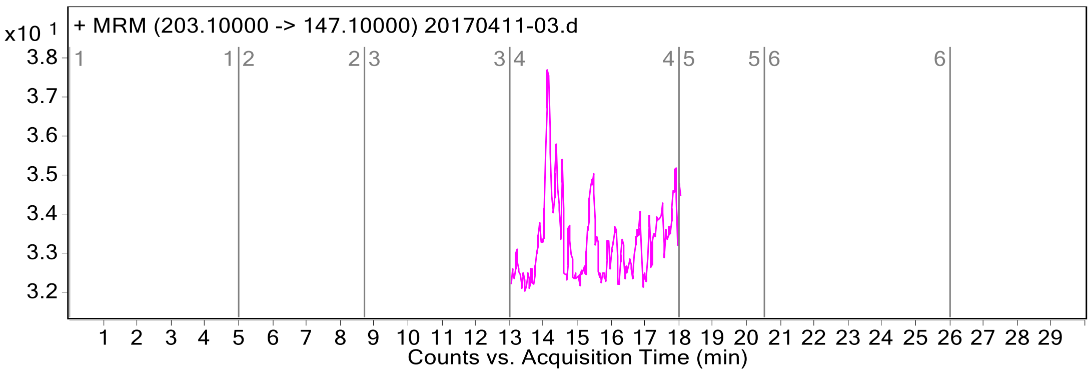
x10 1
+ MRM (203.10000 -> 147.10000) 20170411-03.d
Counts vs. Acquisition Time (min)

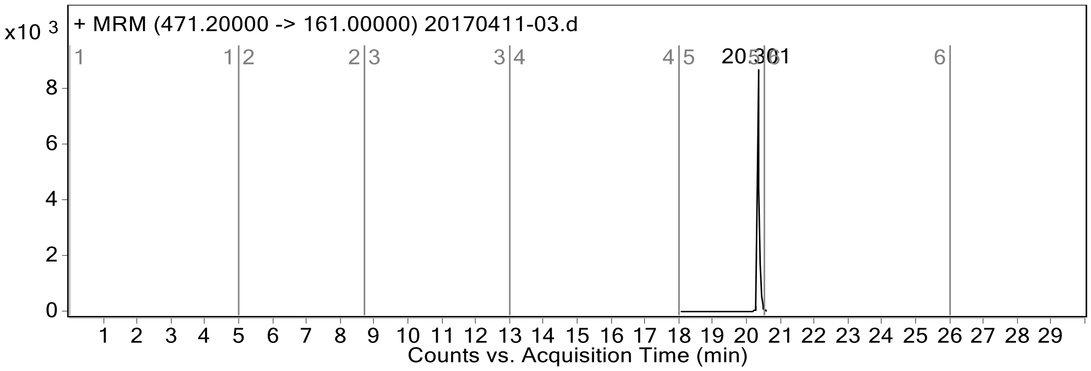
x10 3
+ MRM (471.20000 -> 161.00000) 20170411-03.d
Counts vs. Acquisition Time (min)

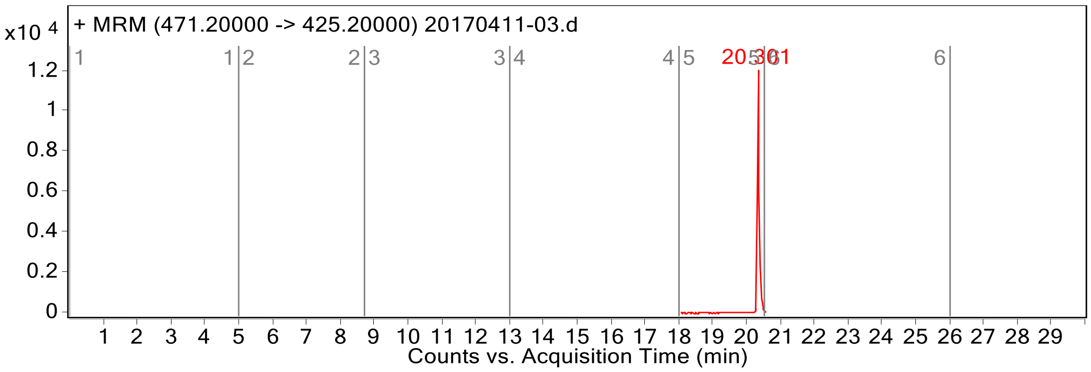
x10 4
+ MRM (471.20000 -> 425.20000) 20170411-03.d
Counts vs. Acquisition Time (min)

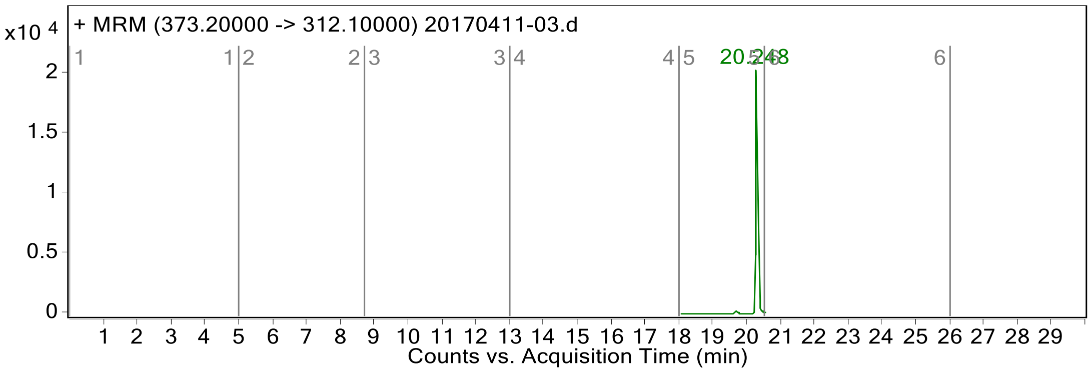
x10 4
+ MRM (373.20000 -> 312.10000) 20170411-03.d
Counts vs. Acquisition Time (min)

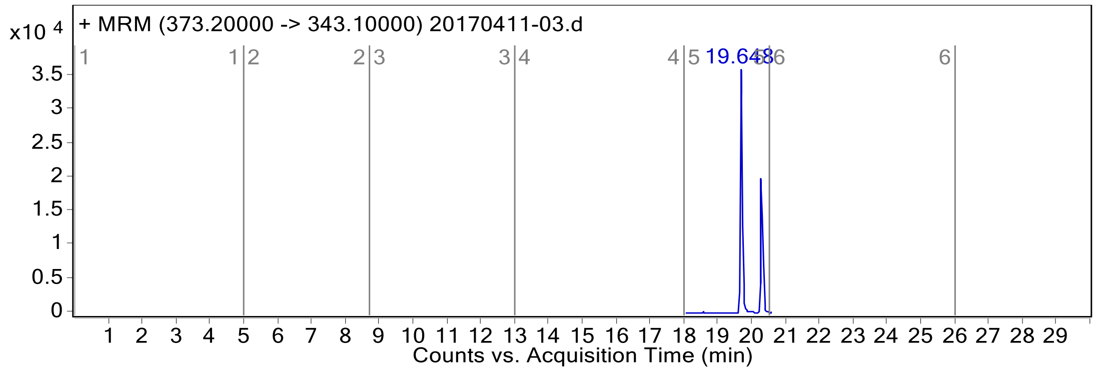
x10 4
+ MRM (373.20000 -> 343.10000) 20170411-03.d
19.648
Counts vs. Acquisition Time (min)

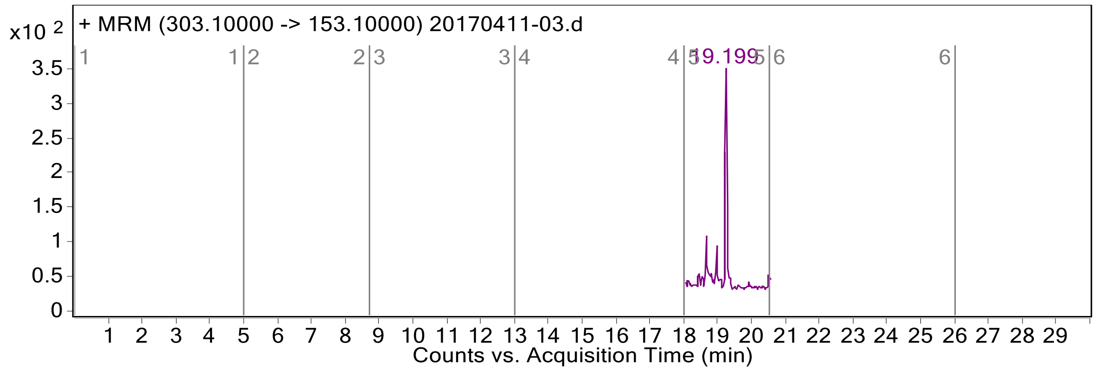
x10 2
+ MRM (303.10000 -> 153.10000) 20170411-03.d
19.199
Counts vs. Acquisition Time (min)

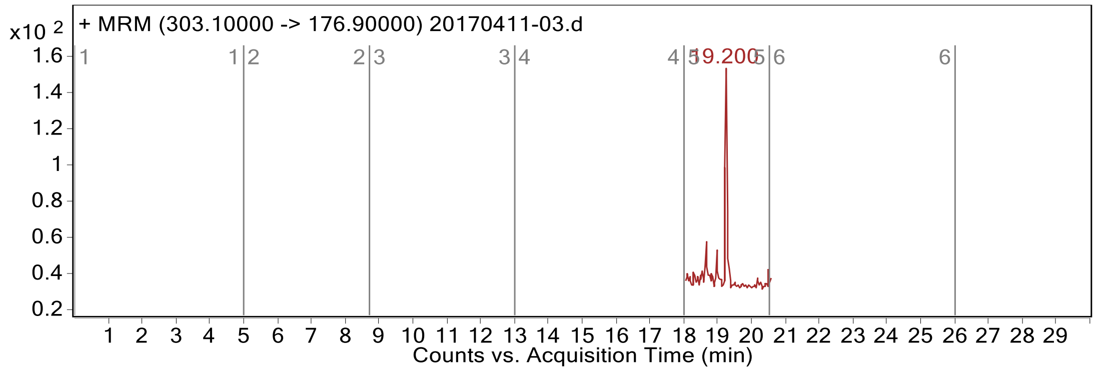
x10 2
+ MRM (303.10000 -> 176.90000) 20170411-03.d
19.200
Counts vs. Acquisition Time (min)

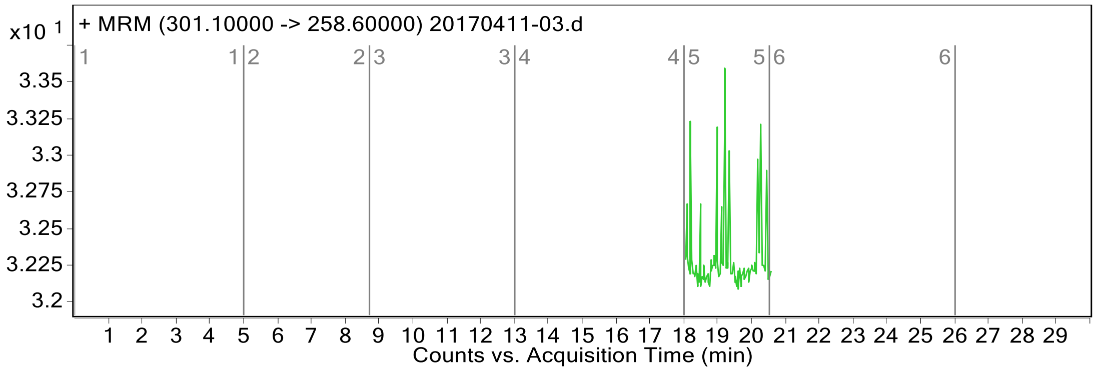
x10 1
+ MRM (301.10000 -> 258.60000) 20170411-03.d
Counts vs. Acquisition Time (min)

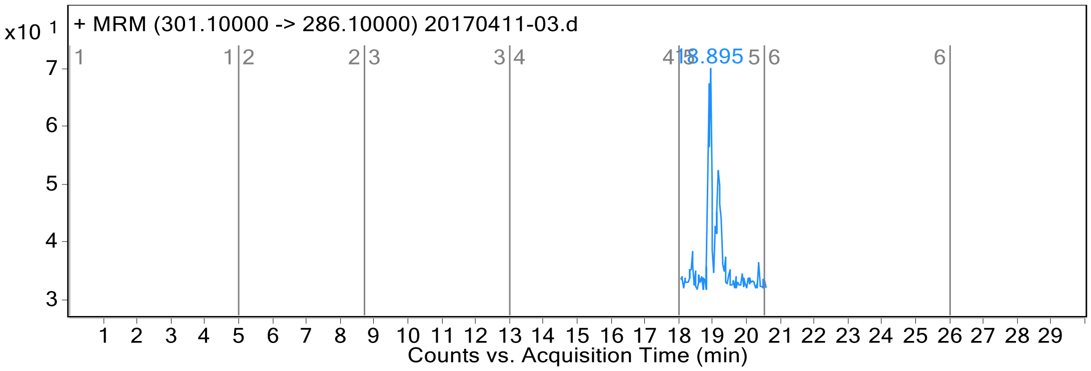
x10 1
+ MRM (301.10000 -> 286.10000) 20170411-03.d
18.895
Counts vs. Acquisition Time (min)

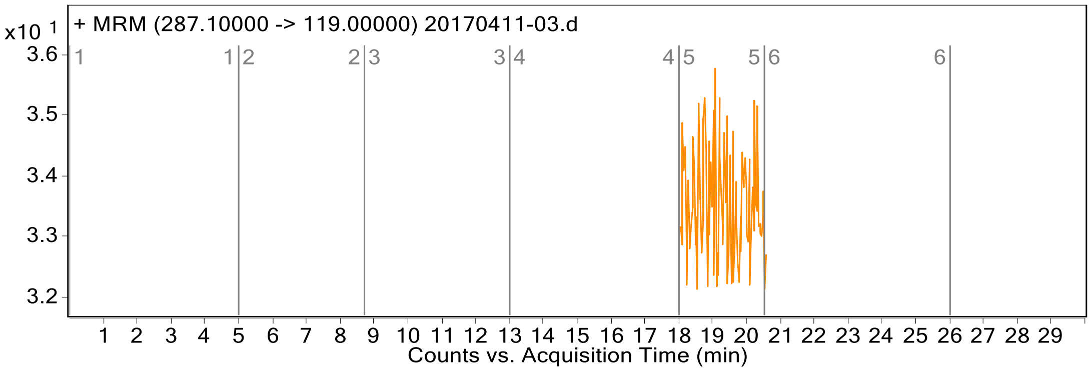
x10 1
+ MRM (287.10000 -> 119.00000) 20170411-03.d
Counts vs. Acquisition Time (min)

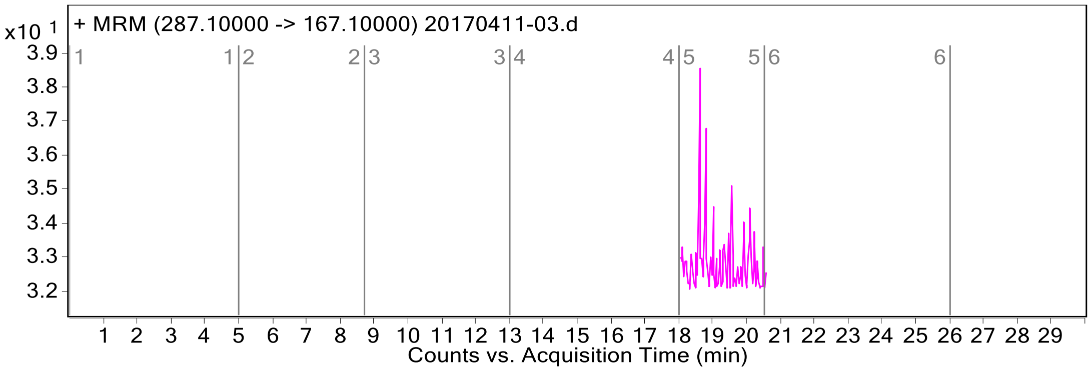
x10 1
+ MRM (287.10000 -> 167.10000) 20170411-03.d
Counts vs. Acquisition Time (min)

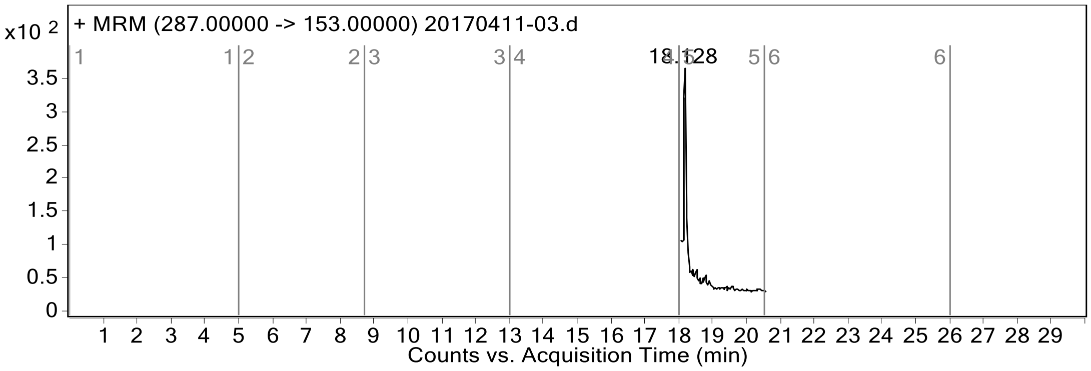
x10 2
+ MRM (287.00000 -> 153.00000) 20170411-03.d
18.128
Counts vs. Acquisition Time (min)

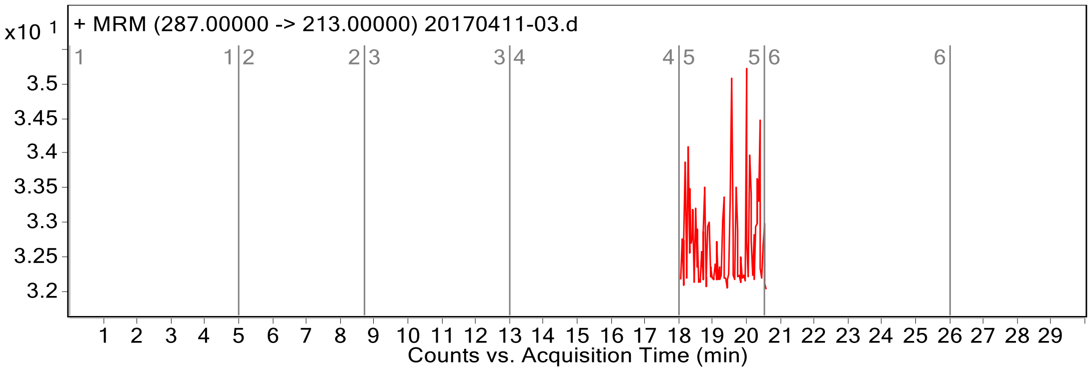
x10 1
+ MRM (287.00000 -> 213.00000) 20170411-03.d
Counts vs. Acquisition Time (min)

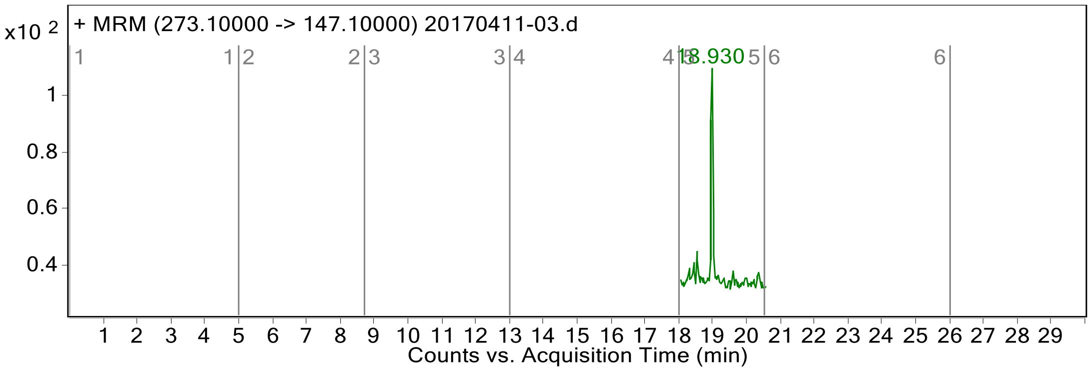
x10 2
+ MRM (273.10000 -> 147.10000) 20170411-03.d
18.930
Counts vs. Acquisition Time (min)

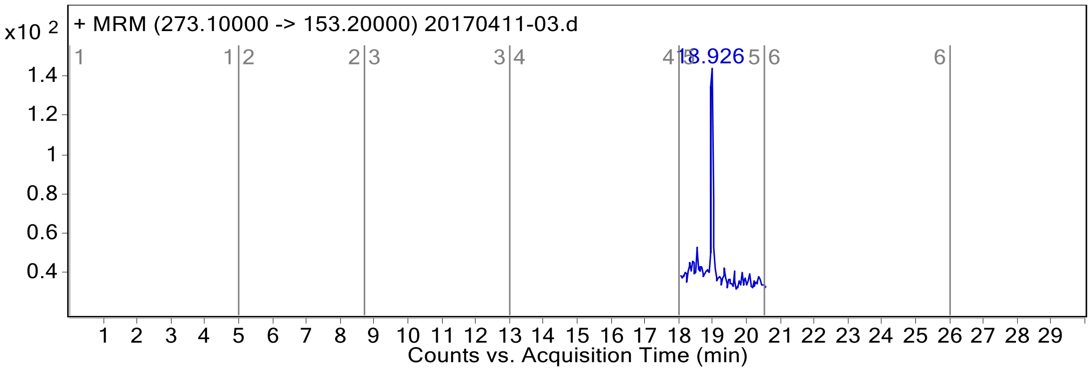
x10 2
+ MRM (273.10000 -> 153.20000) 20170411-03.d
18.926
Counts vs. Acquisition Time (min)

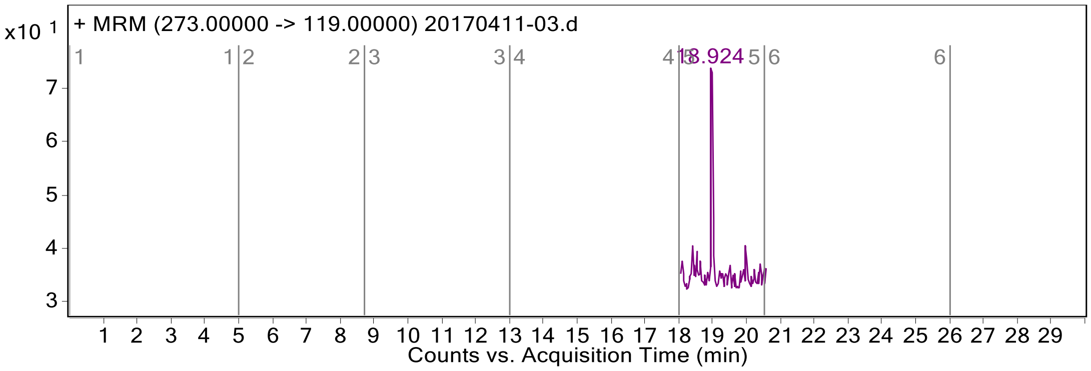
x10 1
+ MRM (273.00000 -> 119.00000) 20170411-03.d
18.924
Counts vs. Acquisition Time (min)

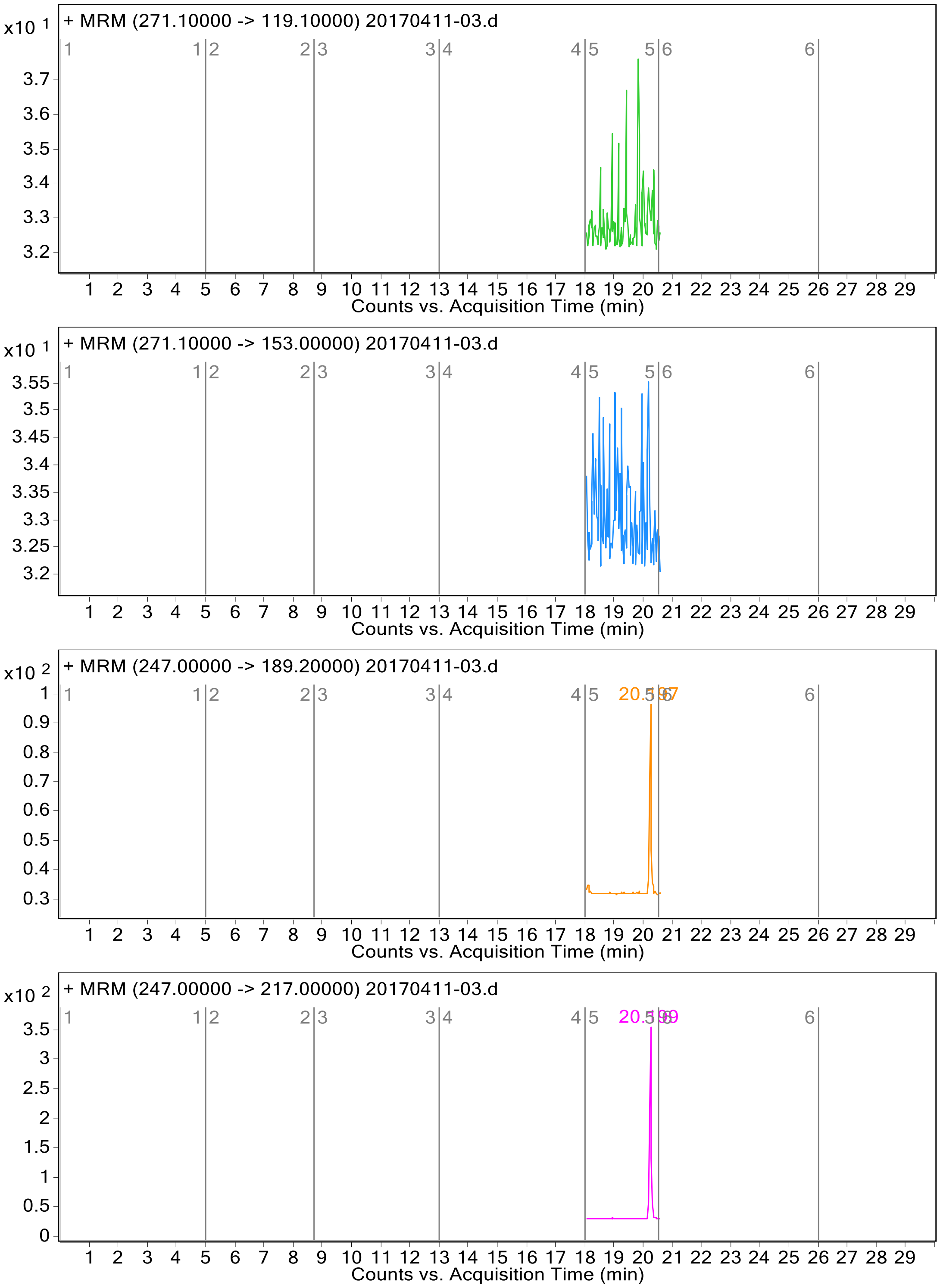

x10 1
+ MRM (271.10000 -> 119.10000) 20170411-03.d
Counts vs. Acquisition Time (min)
x10 1
+ MRM (271.10000 -> 153.00000) 20170411-03.d
Counts vs. Acquisition Time (min)
x10 2
+ MRM (247.00000 -> 189.20000) 20170411-03.d
20.567
Counts vs. Acquisition Time (min)
x10 2
+ MRM (247.00000 -> 217.00000) 20170411-03.d
20.569
Counts vs. Acquisition Time (min)

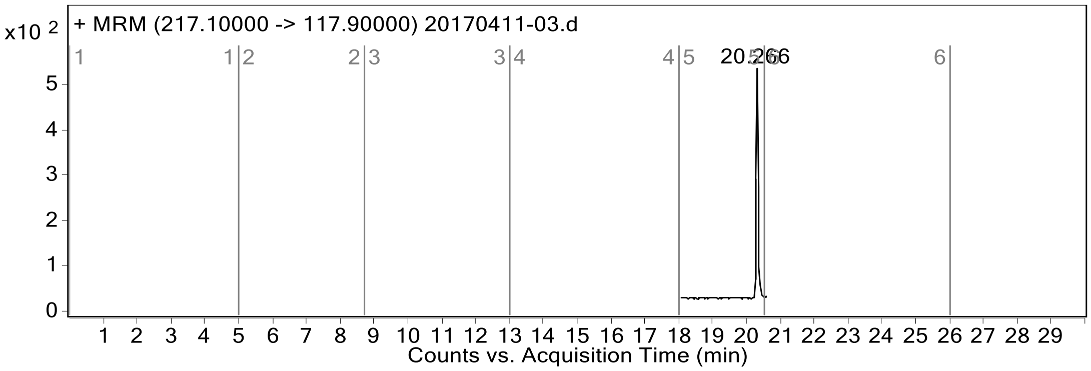
x10 2
+ MRM (217.10000 -> 117.90000) 20170411-03.d
20.266
Counts vs. Acquisition Time (min)

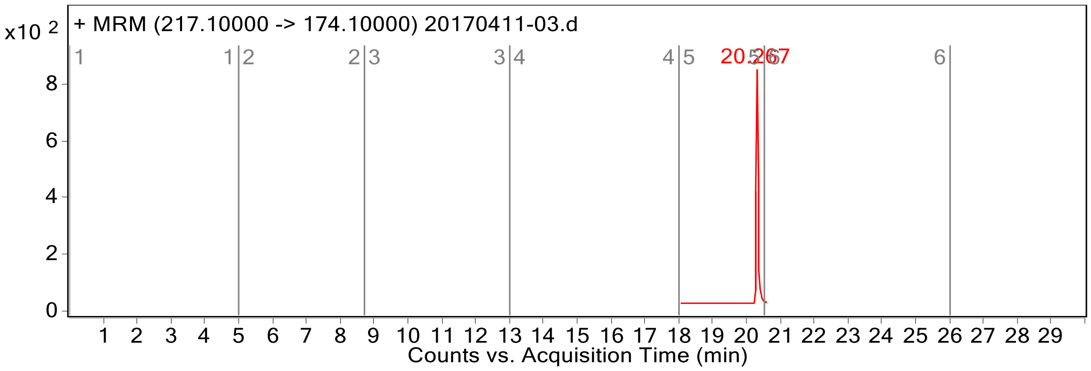
x10 2
+ MRM (217.10000 -> 174.10000) 20170411-03.d
20.267
Counts vs. Acquisition Time (min)

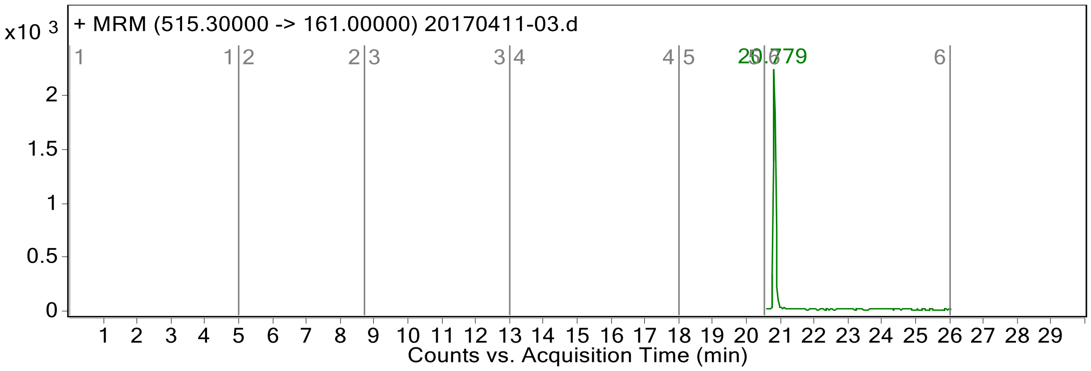
x10 3
+ MRM (515.30000 -> 161.00000) 20170411-03.d
20.779
Counts vs. Acquisition Time (min)

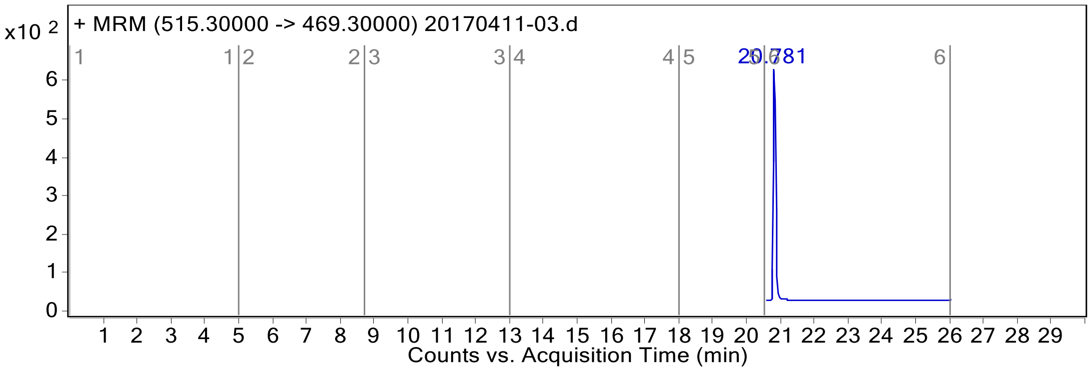
x10 2
+ MRM (515.30000 -> 469.30000) 20170411-03.d
20.781
Counts vs. Acquisition Time (min)

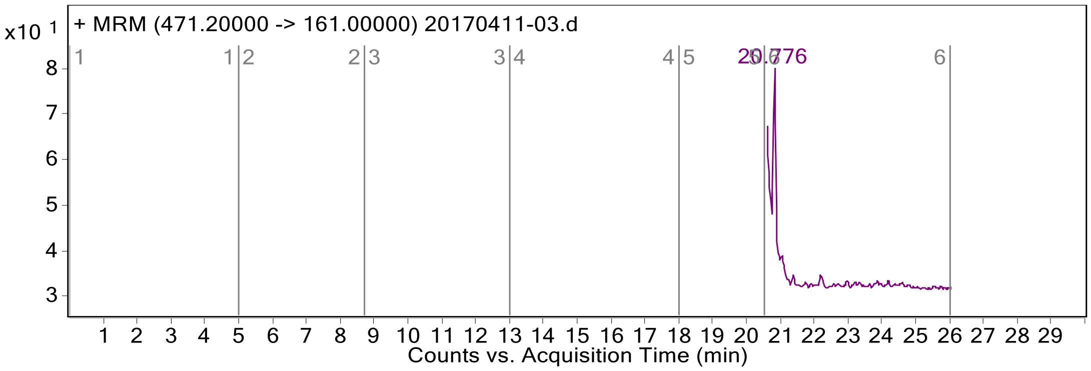
+ MRM (471.20000 -> 161.00000) 20170411-03.d
x10 1
20.776
Counts vs. Acquisition Time (min)

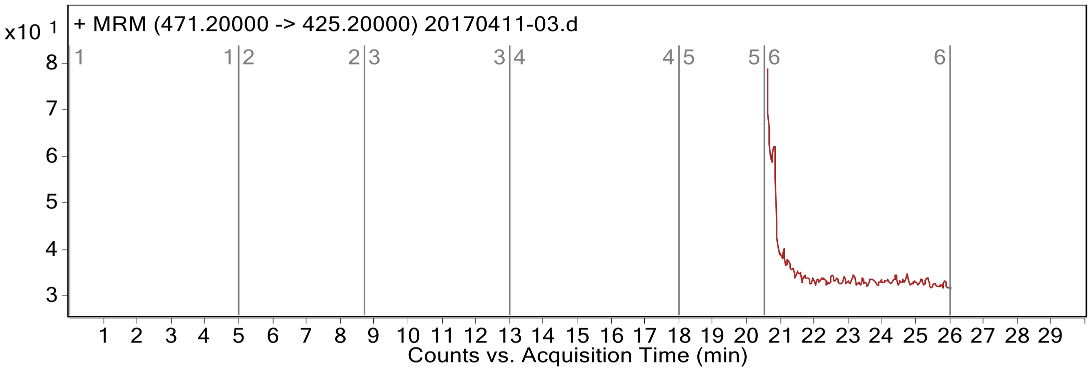
+ MRM (471.20000 -> 425.20000) 20170411-03.d
x10 1
Counts vs. Acquisition Time (min)

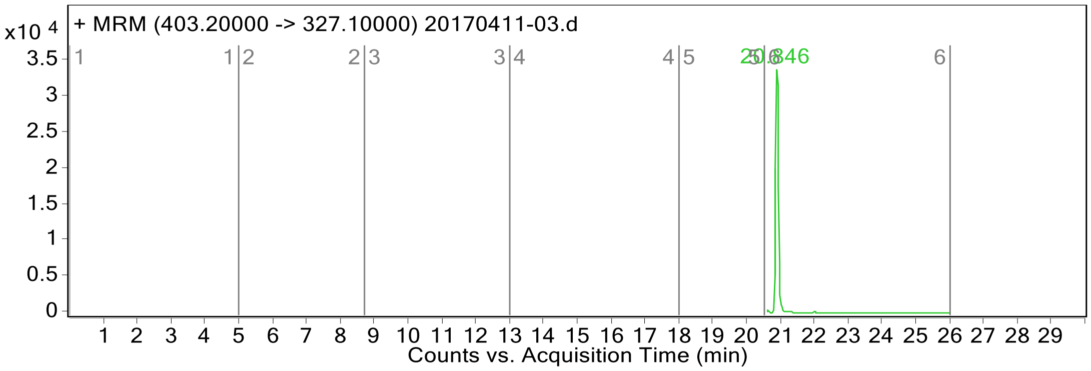
+ MRM (403.20000 -> 327.10000) 20170411-03.d
x10 4
20.846
Counts vs. Acquisition Time (min)

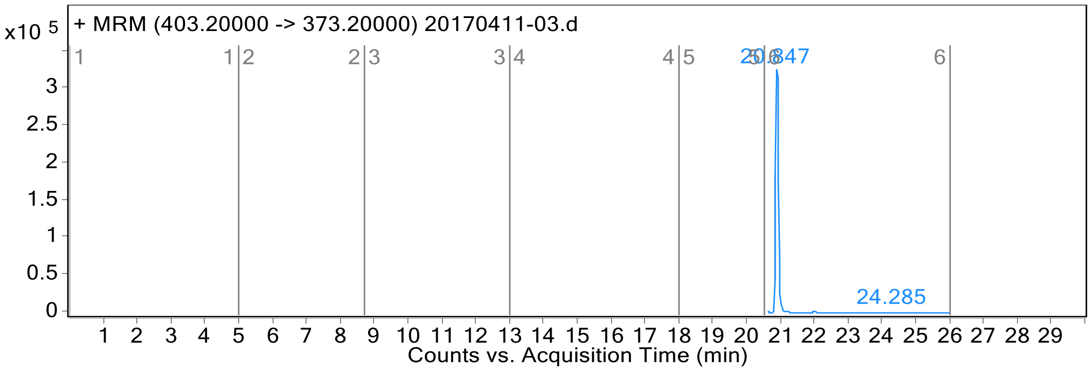
+ MRM (403.20000 -> 373.20000) 20170411-03.d
x10 5
20.847
24.285
Counts vs. Acquisition Time (min)

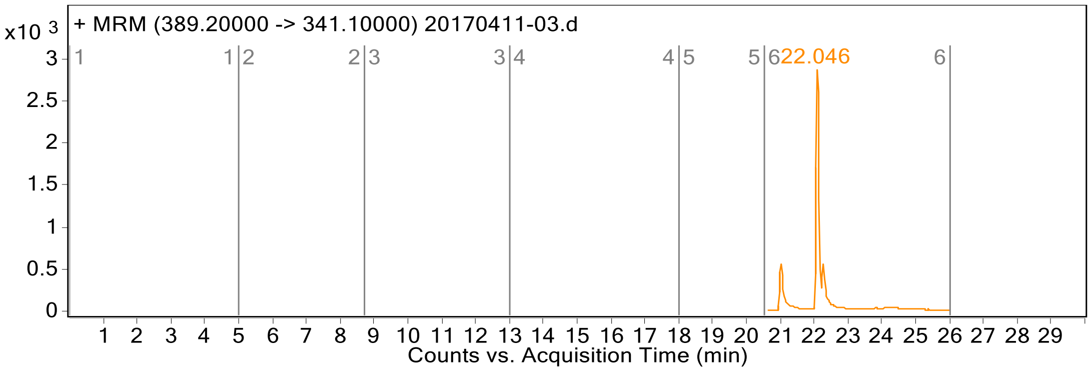
x10 3
+ MRM (389.20000 -> 341.10000) 20170411-03.d
22.046
Counts vs. Acquisition Time (min)

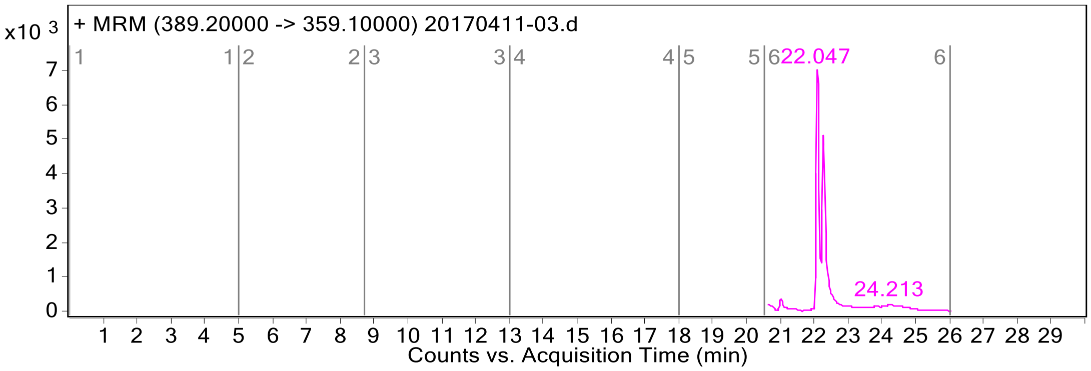
x10 3
+ MRM (389.20000 -> 359.10000) 20170411-03.d
22.047
24.213
Counts vs. Acquisition Time (min)

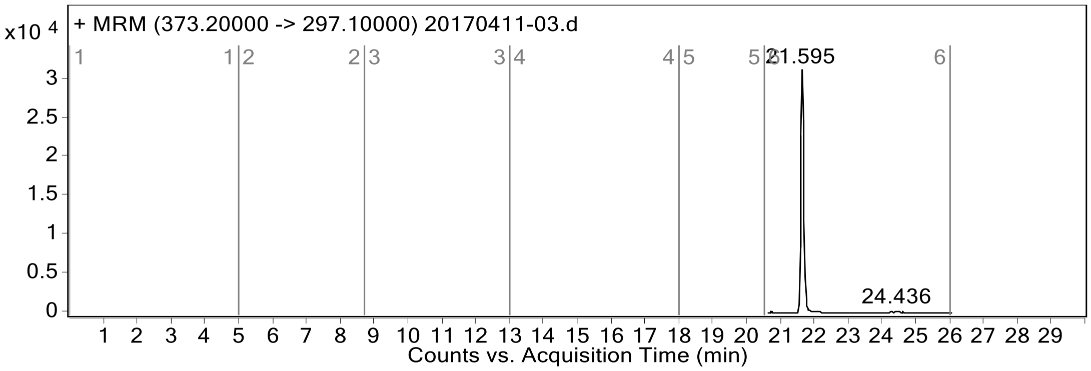
x10 4
+ MRM (373.20000 -> 297.10000) 20170411-03.d
21.595
24.436
Counts vs. Acquisition Time (min)

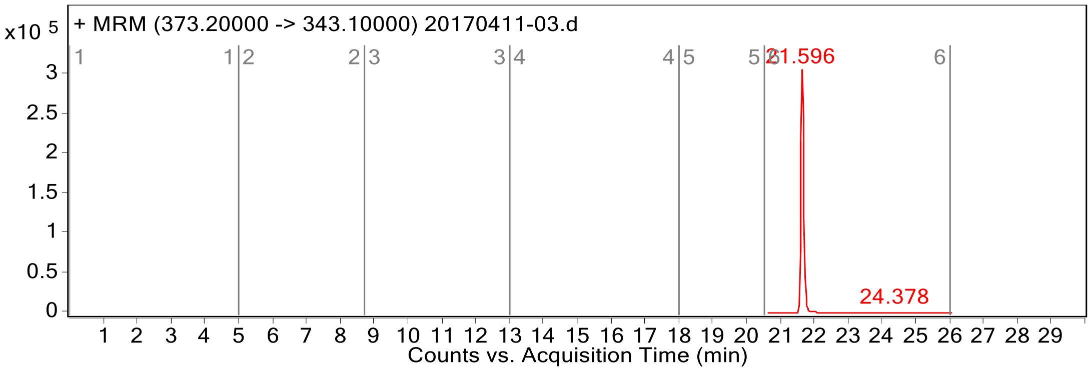
x10 5
+ MRM (373.20000 -> 343.10000) 20170411-03.d
21.596
24.378
Counts vs. Acquisition Time (min)

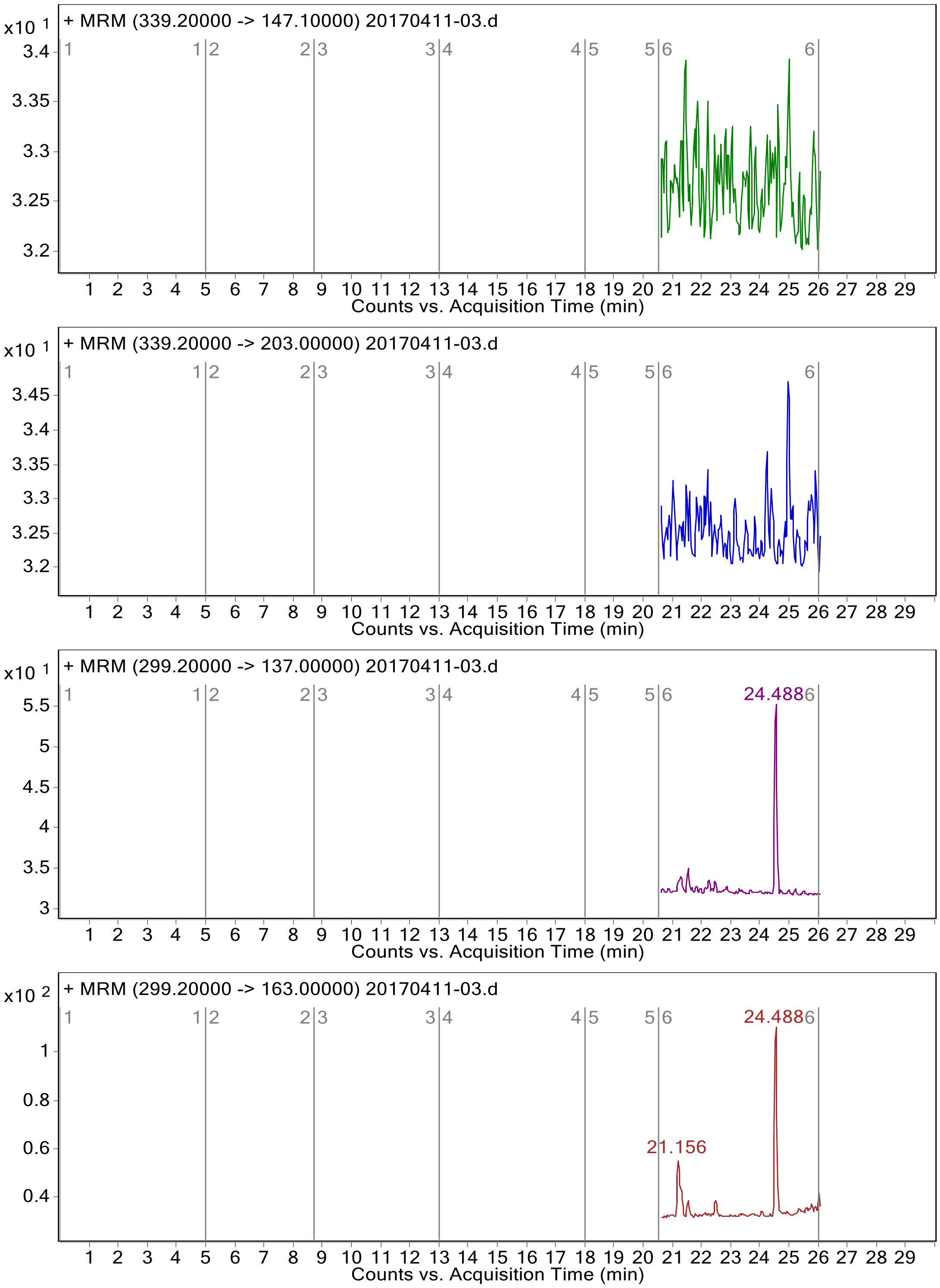
x10 1
+ MRM (339.20000 -> 147.10000) 20170411-03.d
Counts vs. Acquisition Time (min)
x10 1
+ MRM (339.20000 -> 203.00000) 20170411-03.d
Counts vs. Acquisition Time (min)
x10 1
+ MRM (299.20000 -> 137.00000) 20170411-03.d
24.4886
Counts vs. Acquisition Time (min)
x10 2
+ MRM (299.20000 -> 163.00000) 20170411-03.d
21.156
24.4886
Counts vs. Acquisition Time (min)

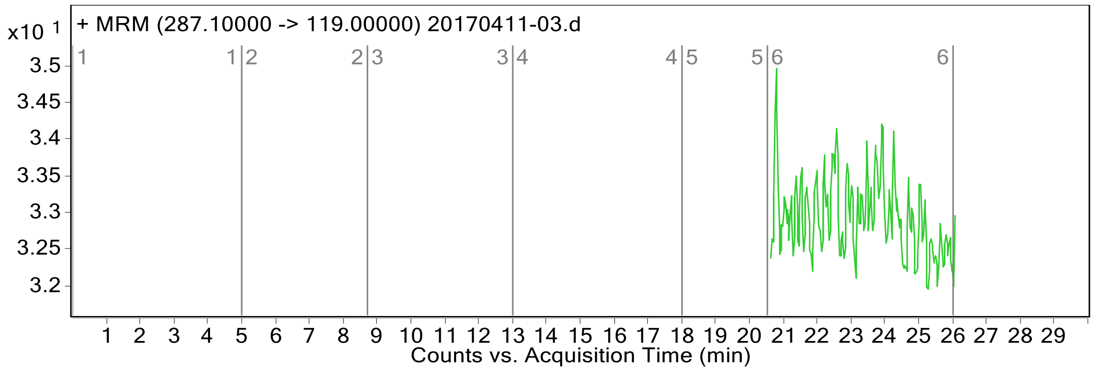
x10 1
+ MRM (287.10000 -> 119.00000) 20170411-03.d
3.5
3.45
3.4
3.35
3.3
3.25
3.2
1 1|2 2|3 3|4 4|5 5|6 6
1 2 3 4 5 6 7 8 9 10 11 12 13 14 15 16 17 18 19 20 21 22 23 24 25 26 27 28 29
Counts vs. Acquisition Time (min)

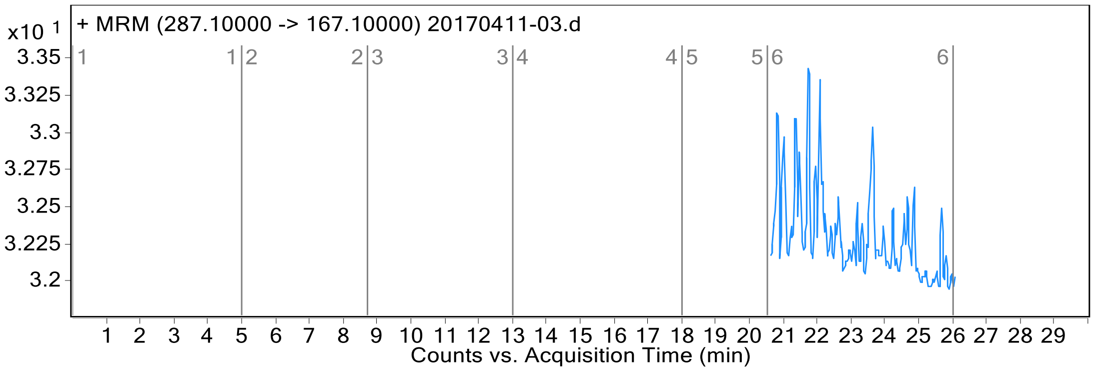
x10 1
+ MRM (287.10000 -> 167.10000) 20170411-03.d
3.35
3.325
3.3
3.275
3.25
3.225
3.2
1 1|2 2|3 3|4 4|5 5|6 6
1 2 3 4 5 6 7 8 9 10 11 12 13 14 15 16 17 18 19 20 21 22 23 24 25 26 27 28 29
Counts vs. Acquisition Time (min)

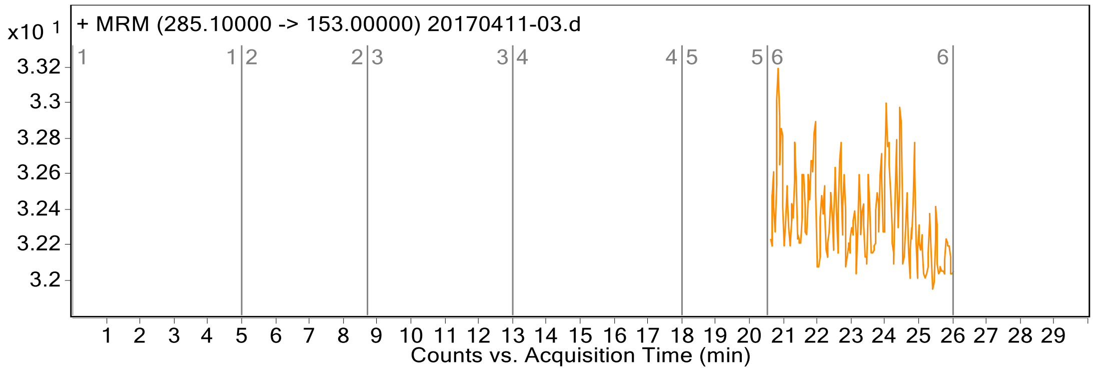
x10 1
+ MRM (285.10000 -> 153.00000) 20170411-03.d
3.32
3.3
3.28
3.26
3.24
3.22
3.2
1 1|2 2|3 3|4 4|5 5|6 6
1 2 3 4 5 6 7 8 9 10 11 12 13 14 15 16 17 18 19 20 21 22 23 24 25 26 27 28 29
Counts vs. Acquisition Time (min)

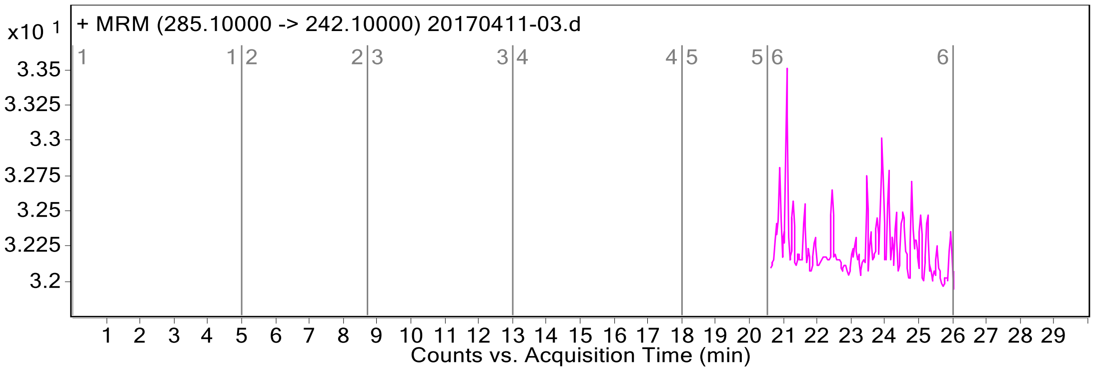
x10 1
+ MRM (285.10000 -> 242.10000) 20170411-03.d
3.35
3.325
3.3
3.275
3.25
3.225
3.2
1 1|2 2|3 3|4 4|5 5|6 6
1 2 3 4 5 6 7 8 9 10 11 12 13 14 15 16 17 18 19 20 21 22 23 24 25 26 27 28 29
Counts vs. Acquisition Time (min)

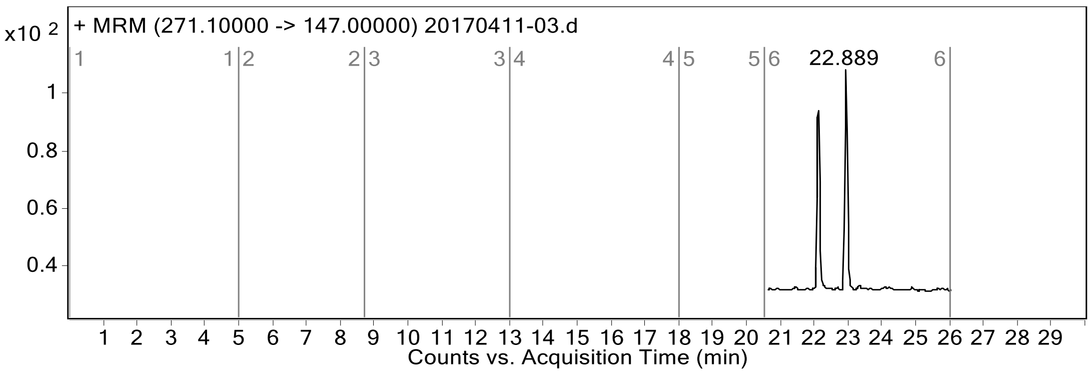

x10 2
+ MRM (271.10000 -> 147.00000) 20170411-03.d
22.889
Counts vs. Acquisition Time (min)

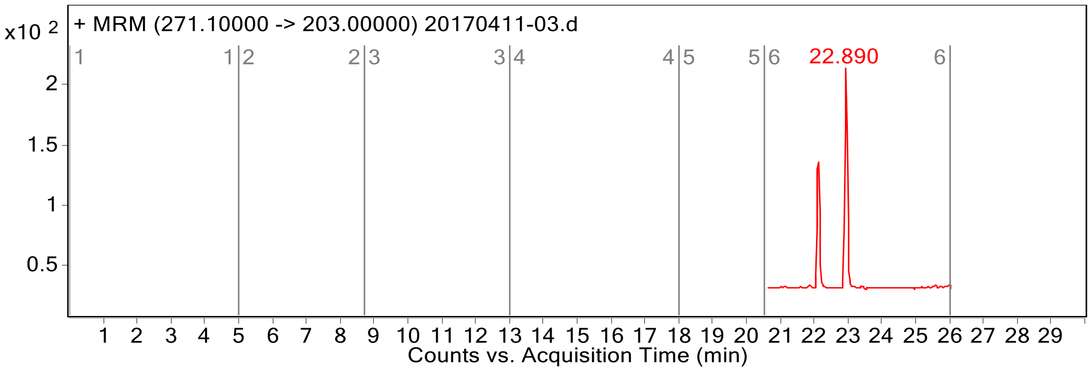

x10 2
+ MRM (271.10000 -> 203.00000) 20170411-03.d
22.890
Counts vs. Acquisition Time (min)

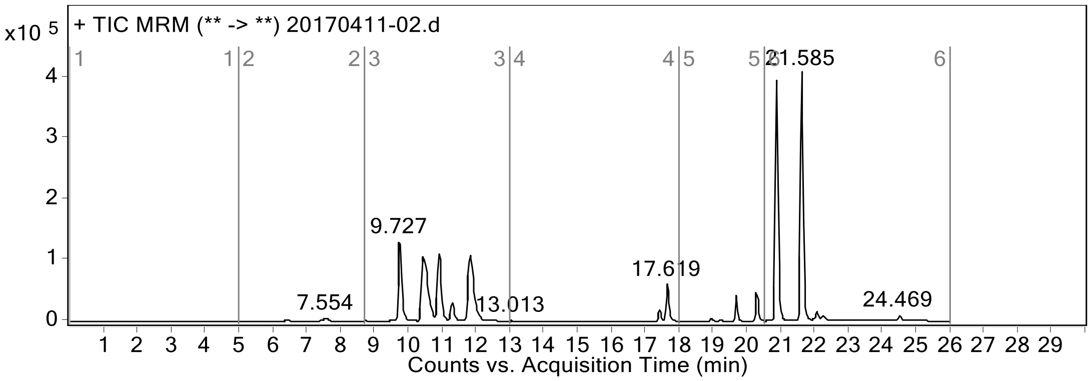

x10 5
+ TIC MRM (** -> **) 20170411-02.d
21.585
9.727
17.619
7.554
13.013
24.469
Counts vs. Acquisition Time (min)

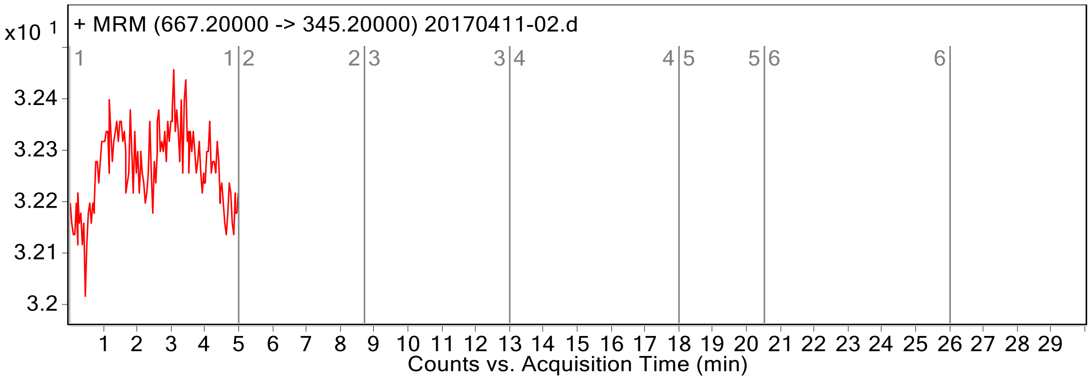

x10 1
+ MRM (667.20000 -> 345.20000) 20170411-02.d
Counts vs. Acquisition Time (min)

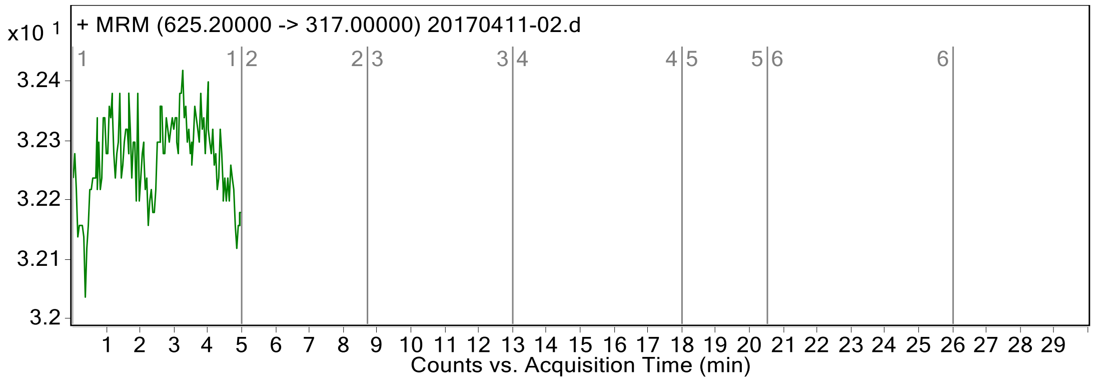
x10 1
+ MRM (625.20000 -> 317.00000) 20170411-02.d
3.24
3.23
3.22
3.21
3.2
1 2 3 4 5 6 7 8 9 10 11 12 13 14 15 16 17 18 19 20 21 22 23 24 25 26 27 28 29
Counts vs. Acquisition Time (min)

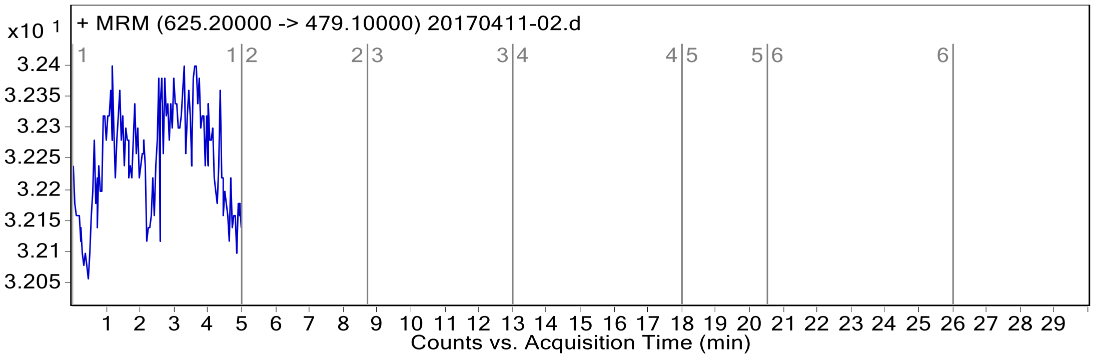
x10 1
+ MRM (625.20000 -> 479.10000) 20170411-02.d
3.24
3.235
3.23
3.225
3.22
3.215
3.21
3.205
1 2 3 4 5 6 7 8 9 10 11 12 13 14 15 16 17 18 19 20 21 22 23 24 25 26 27 28 29
Counts vs. Acquisition Time (min)

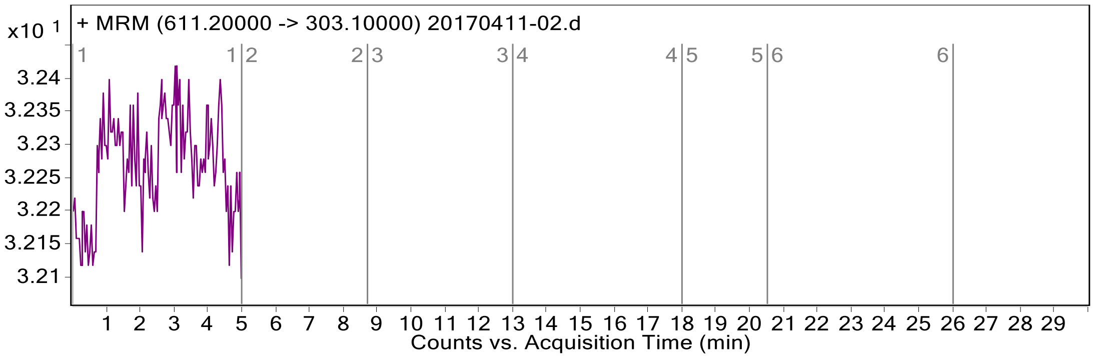
x10 1
+ MRM (611.20000 -> 303.10000) 20170411-02.d
3.24
3.235
3.23
3.225
3.22
3.215
3.21
1 2 3 4 5 6 7 8 9 10 11 12 13 14 15 16 17 18 19 20 21 22 23 24 25 26 27 28 29
Counts vs. Acquisition Time (min)

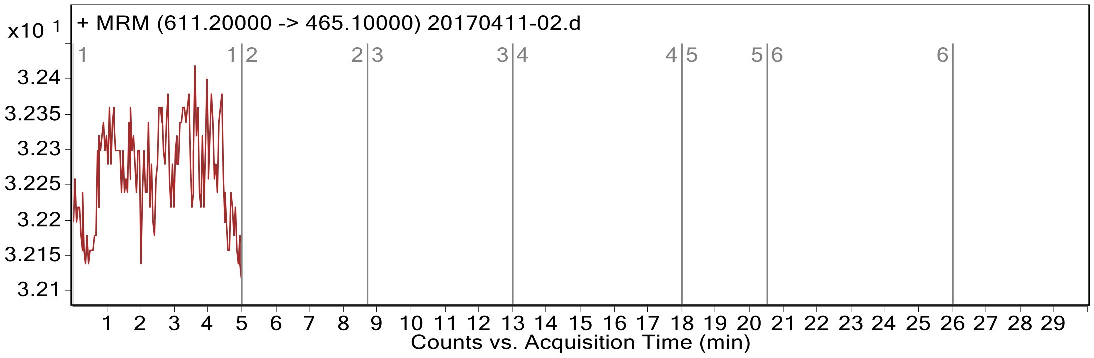
x10 1
+ MRM (611.20000 -> 465.10000) 20170411-02.d
3.24
3.235
3.23
3.225
3.22
3.215
3.21
1 2 3 4 5 6 7 8 9 10 11 12 13 14 15 16 17 18 19 20 21 22 23 24 25 26 27 28 29
Counts vs. Acquisition Time (min)

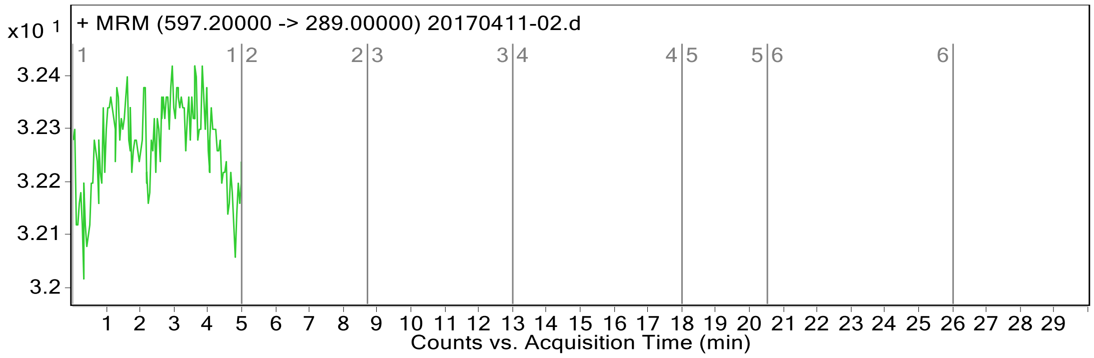
x10 1
+ MRM (597.20000 -> 289.00000) 20170411-02.d
3.24
3.23
3.22
3.21
3.2
1 2 3 4 5 6 7 8 9 10 11 12 13 14 15 16 17 18 19 20 21 22 23 24 25 26 27 28 29
Counts vs. Acquisition Time (min)

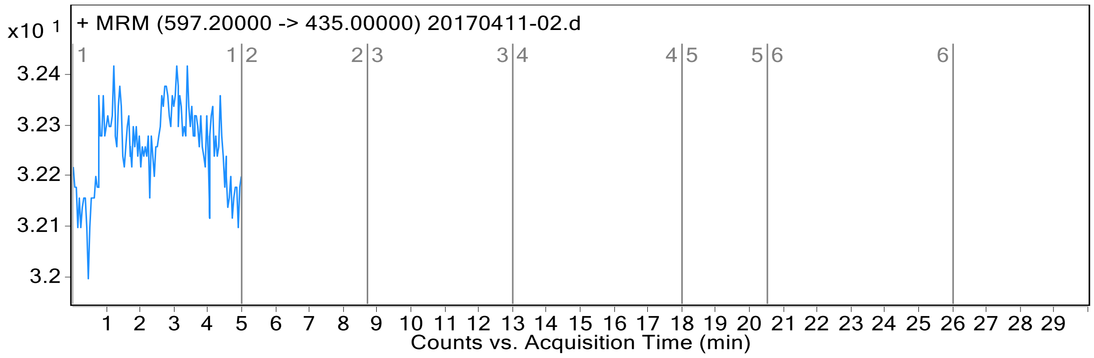
x10 1
+ MRM (597.20000 -> 435.00000) 20170411-02.d
3.24
3.23
3.22
3.21
3.2
1 2 3 4 5 6 7 8 9 10 11 12 13 14 15 16 17 18 19 20 21 22 23 24 25 26 27 28 29
Counts vs. Acquisition Time (min)

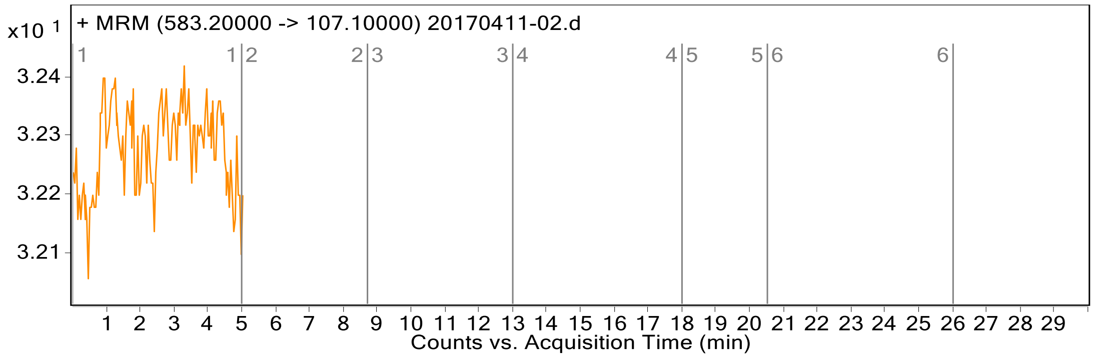
x10 1
+ MRM (583.20000 -> 107.10000) 20170411-02.d
3.24
3.23
3.22
3.21
1 2 3 4 5 6 7 8 9 10 11 12 13 14 15 16 17 18 19 20 21 22 23 24 25 26 27 28 29
Counts vs. Acquisition Time (min)

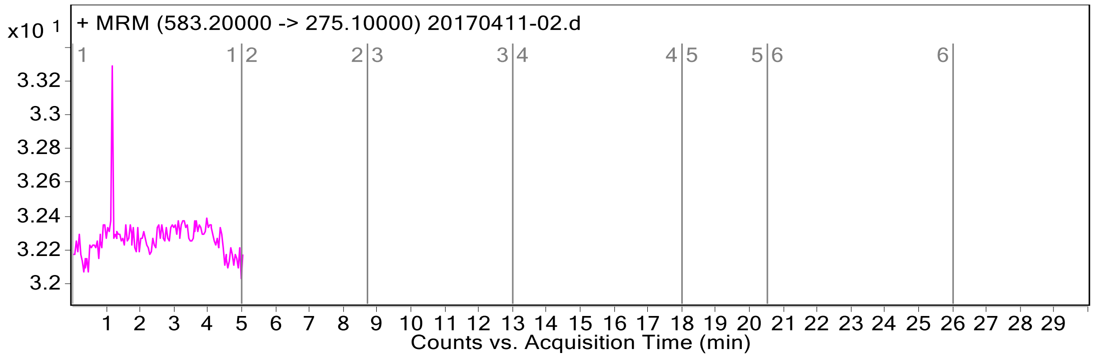
x10 1
+ MRM (583.20000 -> 275.10000) 20170411-02.d
3.32
3.3
3.28
3.26
3.24
3.22
3.2
1 2 3 4 5 6 7 8 9 10 11 12 13 14 15 16 17 18 19 20 21 22 23 24 25 26 27 28 29
Counts vs. Acquisition Time (min)

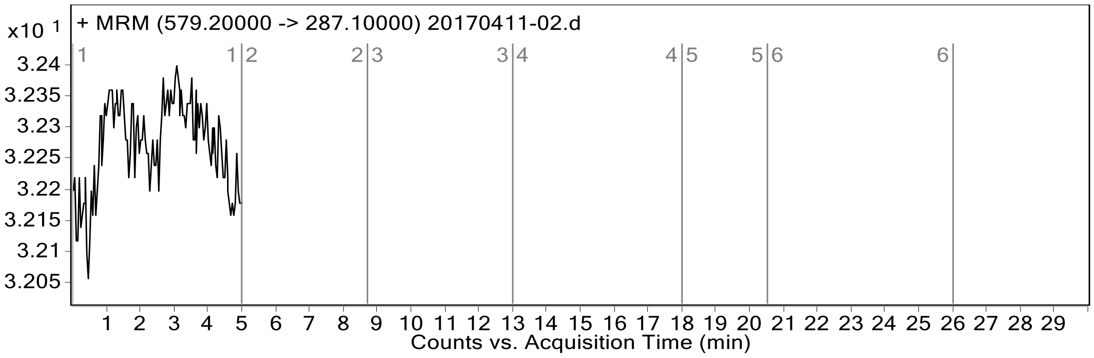
x10 1
+ MRM (579.20000 -> 287.10000) 20170411-02.d
3.24
3.235
3.23
3.225
3.22
3.215
3.21
3.205
1 2 3 4 5 6 7 8 9 10 11 12 13 14 15 16 17 18 19 20 21 22 23 24 25 26 27 28 29
Counts vs. Acquisition Time (min)

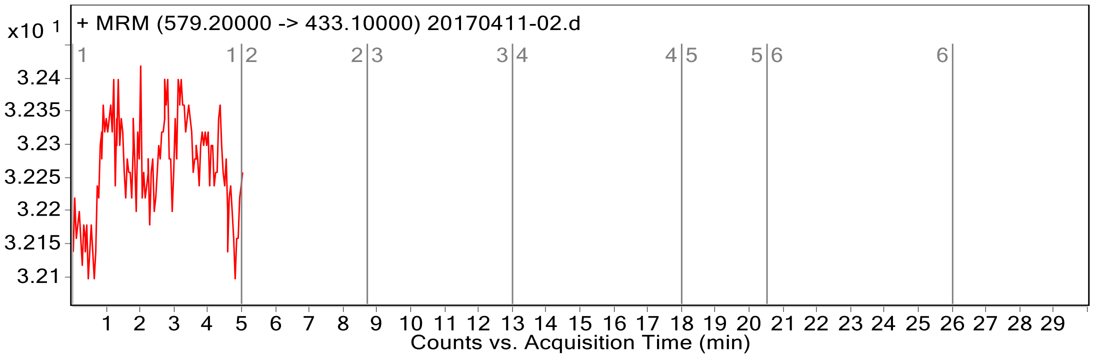
x10 1
+ MRM (579.20000 -> 433.10000) 20170411-02.d
1 1 2 2 3 3 4 4 5 5 6 6
3.24
3.235
3.23
3.225
3.22
3.215
3.21
1 2 3 4 5 6 7 8 9 10 11 12 13 14 15 16 17 18 19 20 21 22 23 24 25 26 27 28 29
Counts vs. Acquisition Time (min)

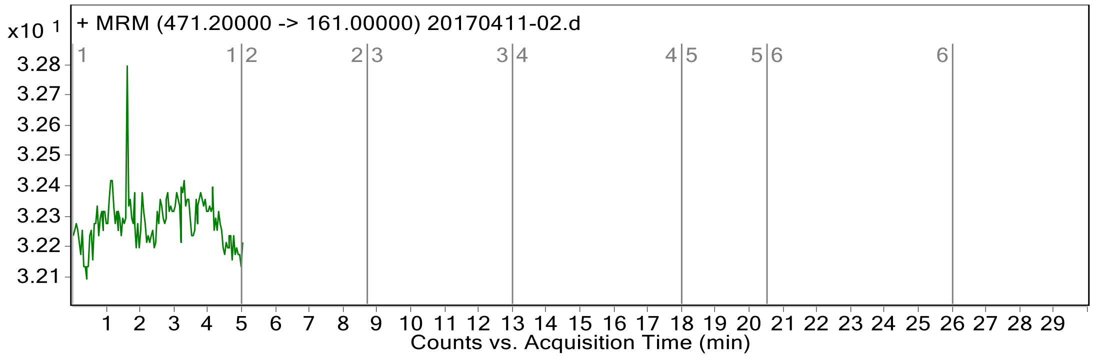
x10 1
+ MRM (471.20000 -> 161.00000) 20170411-02.d
1 1 2 2 3 3 4 4 5 5 6 6
3.28
3.27
3.26
3.25
3.24
3.23
3.22
3.21
1 2 3 4 5 6 7 8 9 10 11 12 13 14 15 16 17 18 19 20 21 22 23 24 25 26 27 28 29
Counts vs. Acquisition Time (min)

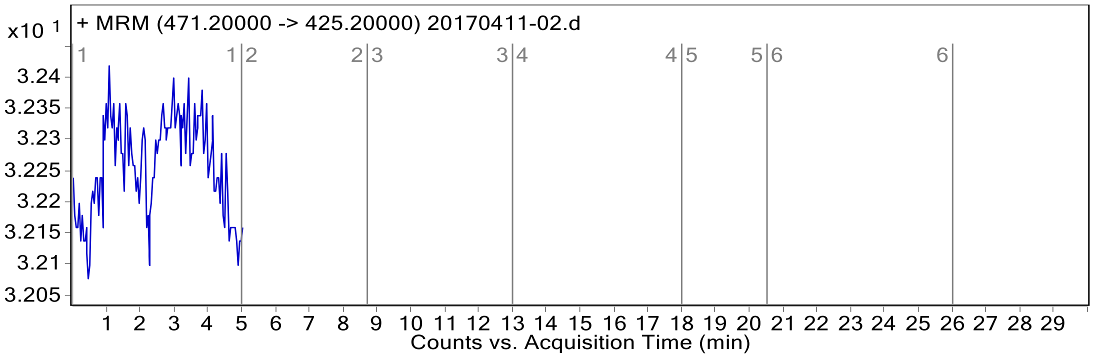
x10 1
+ MRM (471.20000 -> 425.20000) 20170411-02.d
1 1 2 2 3 3 4 4 5 5 6 6
3.24
3.235
3.23
3.225
3.22
3.215
3.21
3.205
1 2 3 4 5 6 7 8 9 10 11 12 13 14 15 16 17 18 19 20 21 22 23 24 25 26 27 28 29
Counts vs. Acquisition Time (min)

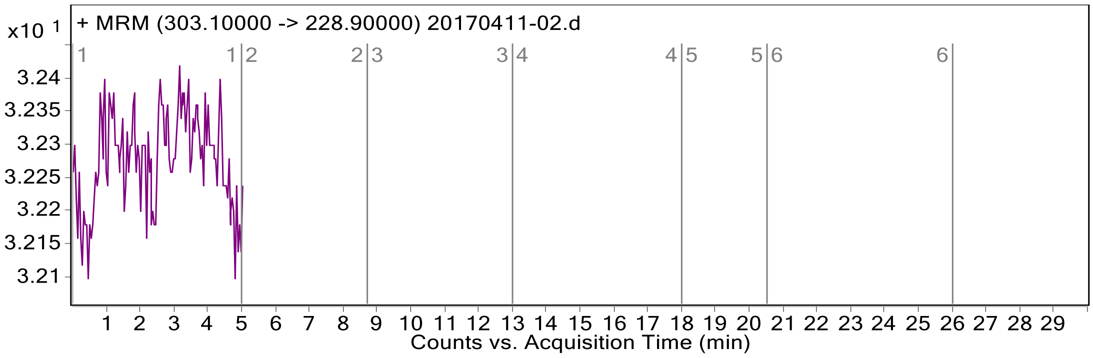
x10 1
+ MRM (303.10000 -> 228.90000) 20170411-02.d
1 1 2 2 3 3 4 4 5 5 6 6
3.24
3.235
3.23
3.225
3.22
3.215
3.21
1 2 3 4 5 6 7 8 9 10 11 12 13 14 15 16 17 18 19 20 21 22 23 24 25 26 27 28 29
Counts vs. Acquisition Time (min)

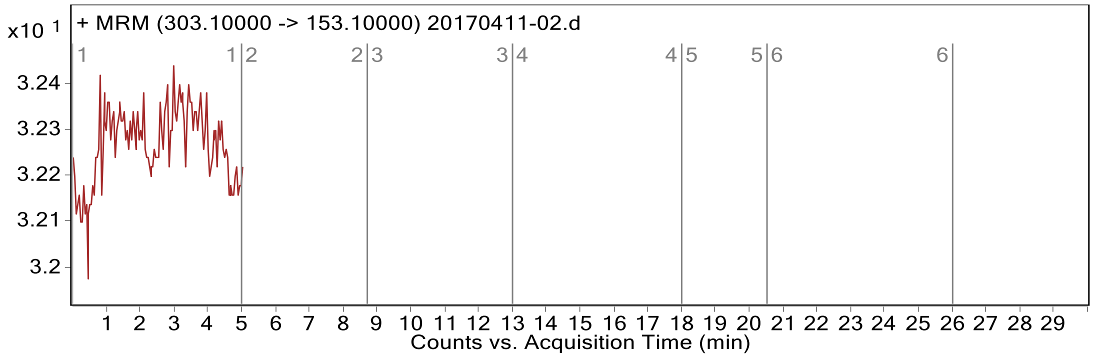
x10 1
+ MRM (303.10000 -> 153.10000) 20170411-02.d
1 1 2 2 3 3 4 4 5 5 6 6
3.24
3.23
3.22
3.21
3.2
1 2 3 4 5 6 7 8 9 10 11 12 13 14 15 16 17 18 19 20 21 22 23 24 25 26 27 28 29
Counts vs. Acquisition Time (min)

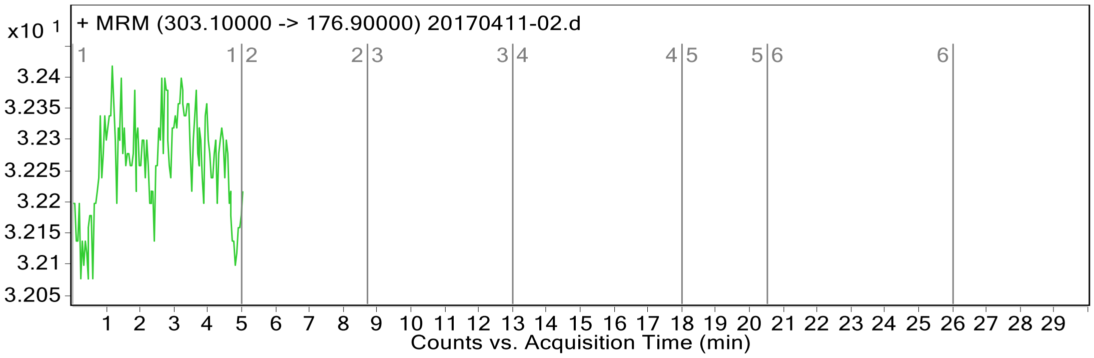
x10 1
+ MRM (303.10000 -> 176.90000) 20170411-02.d
3.24
3.235
3.23
3.225
3.22
3.215
3.21
3.205
1 2 3 4 5 6 7 8 9 10 11 12 13 14 15 16 17 18 19 20 21 22 23 24 25 26 27 28 29
Counts vs. Acquisition Time (min)

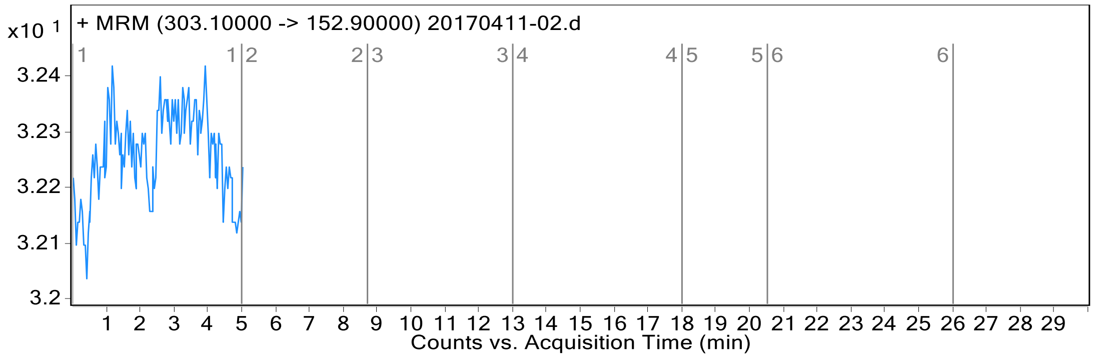
x10 1
+ MRM (303.10000 -> 152.90000) 20170411-02.d
3.24
3.23
3.22
3.21
3.2
1 2 3 4 5 6 7 8 9 10 11 12 13 14 15 16 17 18 19 20 21 22 23 24 25 26 27 28 29
Counts vs. Acquisition Time (min)

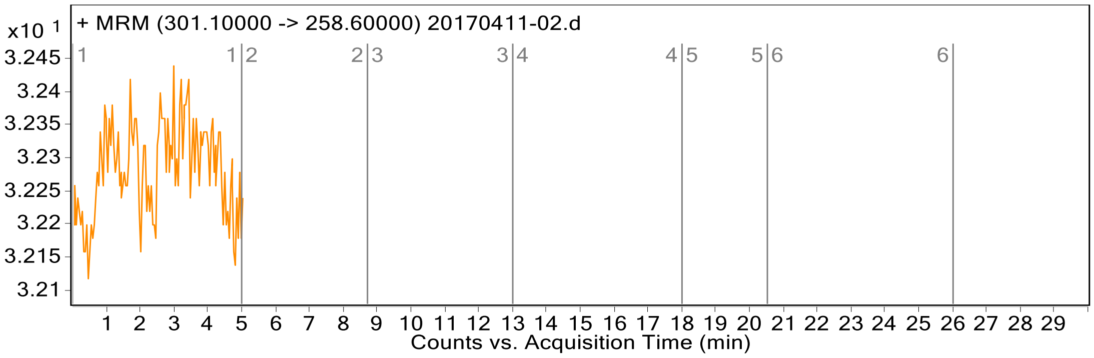
x10 1
+ MRM (301.10000 -> 258.60000) 20170411-02.d
3.245
3.24
3.235
3.23
3.225
3.22
3.215
3.21
1 2 3 4 5 6 7 8 9 10 11 12 13 14 15 16 17 18 19 20 21 22 23 24 25 26 27 28 29
Counts vs. Acquisition Time (min)

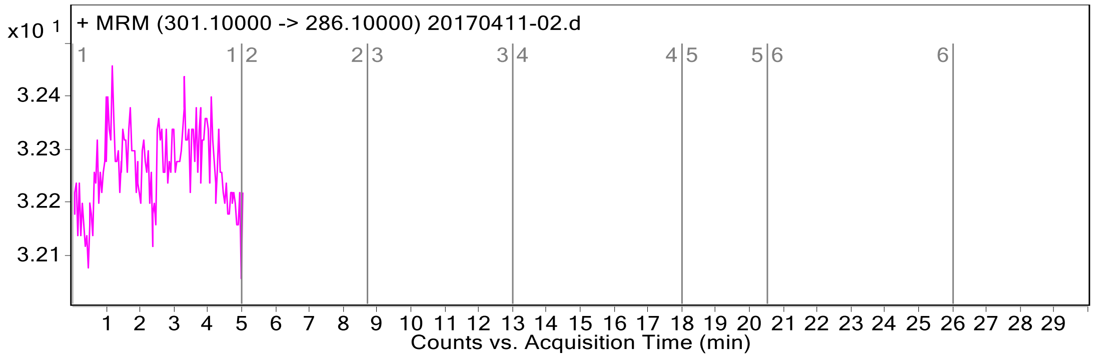
x10 1
+ MRM (301.10000 -> 286.10000) 20170411-02.d
3.24
3.23
3.22
3.21
1 2 3 4 5 6 7 8 9 10 11 12 13 14 15 16 17 18 19 20 21 22 23 24 25 26 27 28 29
Counts vs. Acquisition Time (min)

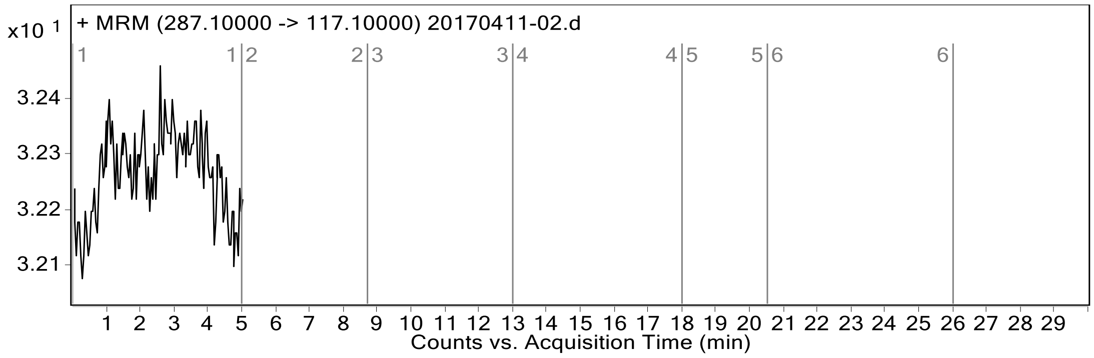
x10 1
+ MRM (287.10000 -> 117.10000) 20170411-02.d
3.24
3.23
3.22
3.21
1 2 3 4 5 6 7 8 9 10 11 12 13 14 15 16 17 18 19 20 21 22 23 24 25 26 27 28 29
Counts vs. Acquisition Time (min)

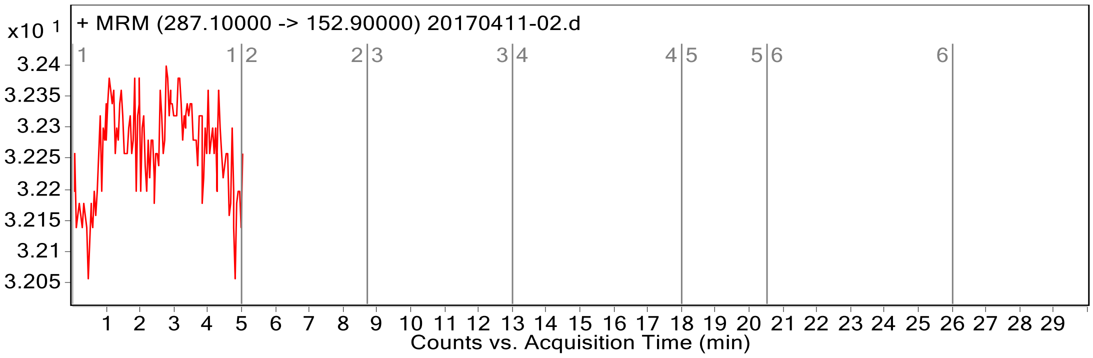
x10 1
+ MRM (287.10000 -> 152.90000) 20170411-02.d
3.24
3.235
3.23
3.225
3.22
3.215
3.21
3.205
1 2 3 4 5 6 7 8 9 10 11 12 13 14 15 16 17 18 19 20 21 22 23 24 25 26 27 28 29
Counts vs. Acquisition Time (min)

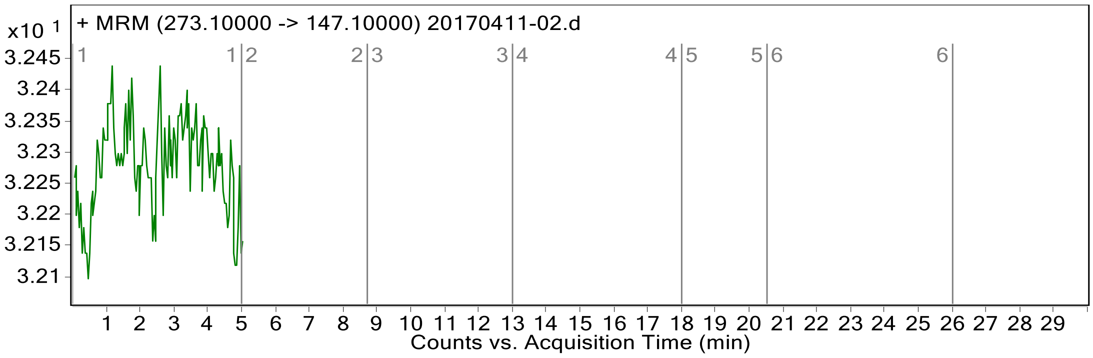
x10 1
+ MRM (273.10000 -> 147.10000) 20170411-02.d
3.245
3.24
3.235
3.23
3.225
3.22
3.215
3.21
1 2 3 4 5 6 7 8 9 10 11 12 13 14 15 16 17 18 19 20 21 22 23 24 25 26 27 28 29
Counts vs. Acquisition Time (min)

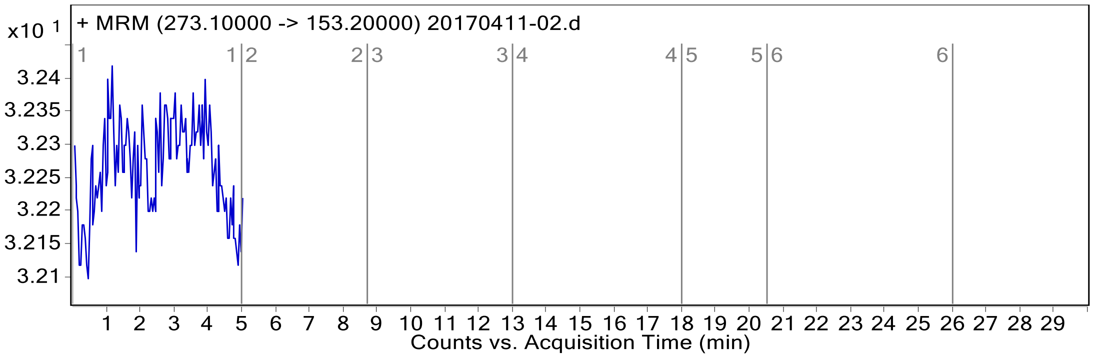
x10 1
+ MRM (273.10000 -> 153.20000) 20170411-02.d
3.24
3.235
3.23
3.225
3.22
3.215
3.21
1 2 3 4 5 6 7 8 9 10 11 12 13 14 15 16 17 18 19 20 21 22 23 24 25 26 27 28 29
Counts vs. Acquisition Time (min)

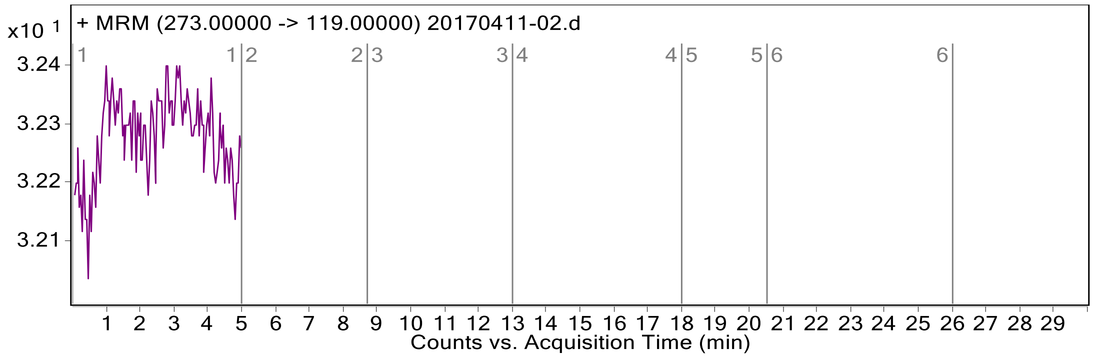
x10 1
+ MRM (273.00000 -> 119.00000) 20170411-02.d
3.24
3.23
3.22
3.21
1 2 3 4 5 6 7 8 9 10 11 12 13 14 15 16 17 18 19 20 21 22 23 24 25 26 27 28 29
Counts vs. Acquisition Time (min)

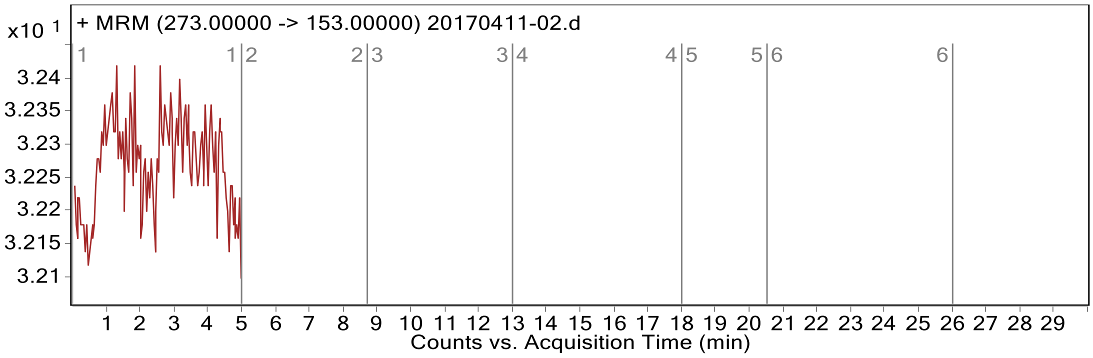
x10 1
+ MRM (273.00000 -> 153.00000) 20170411-02.d
3.24
3.235
3.23
3.225
3.22
3.215
3.21
1 2 3 4 5 6 7 8 9 10 11 12 13 14 15 16 17 18 19 20 21 22 23 24 25 26 27 28 29
Counts vs. Acquisition Time (min)

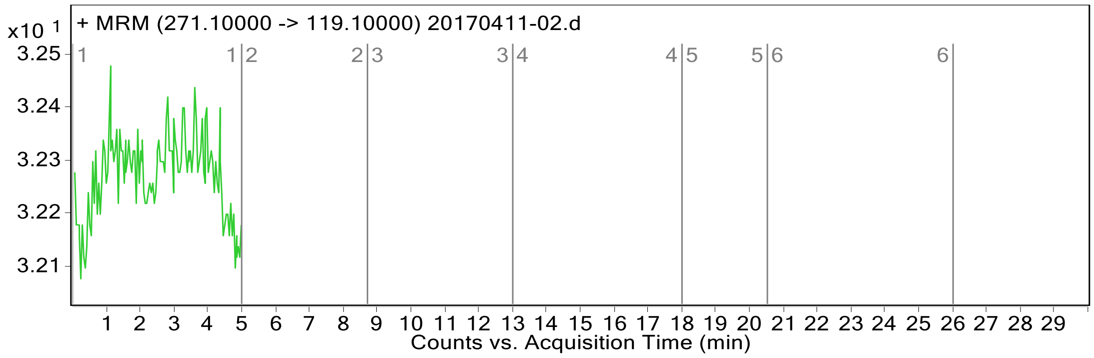

+ MRM (271.10000 -> 119.10000) 20170411-02.d
x10 1
3.25
3.24
3.23
3.22
3.21
1 2 3 4 5 6 7 8 9 10 11 12 13 14 15 16 17 18 19 20 21 22 23 24 25 26 27 28 29
Counts vs. Acquisition Time (min)

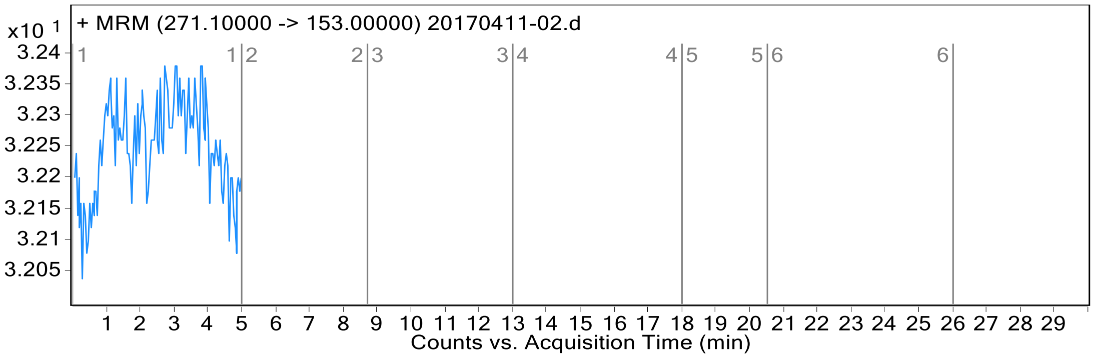

+ MRM (271.10000 -> 153.00000) 20170411-02.d
x10 1
3.24
3.235
3.23
3.225
3.22
3.215
3.21
3.205
1 2 3 4 5 6 7 8 9 10 11 12 13 14 15 16 17 18 19 20 21 22 23 24 25 26 27 28 29
Counts vs. Acquisition Time (min)

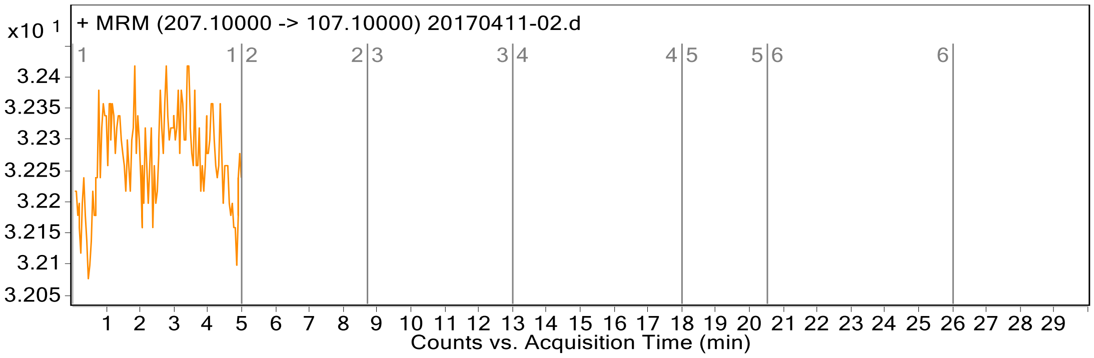

+ MRM (207.10000 -> 107.10000) 20170411-02.d
x10 1
3.24
3.235
3.23
3.225
3.22
3.215
3.21
3.205
1 2 3 4 5 6 7 8 9 10 11 12 13 14 15 16 17 18 19 20 21 22 23 24 25 26 27 28 29
Counts vs. Acquisition Time (min)

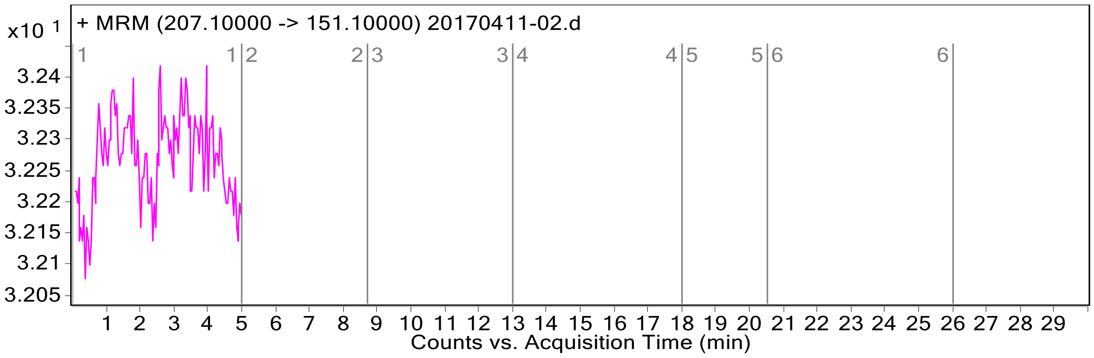

+ MRM (207.10000 -> 151.10000) 20170411-02.d
x10 1
3.24
3.235
3.23
3.225
3.22
3.215
3.21
3.205
1 2 3 4 5 6 7 8 9 10 11 12 13 14 15 16 17 18 19 20 21 22 23 24 25 26 27 28 29
Counts vs. Acquisition Time (min)

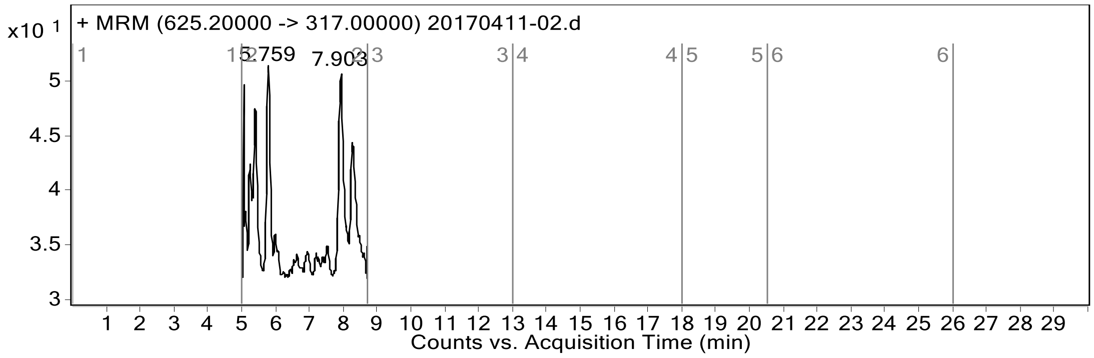

+ MRM (625.20000 -> 317.00000) 20170411-02.d
x10 1
5.759
7.903
5
4.5
4
3.5
3
1 2 3 4 5 6 7 8 9 10 11 12 13 14 15 16 17 18 19 20 21 22 23 24 25 26 27 28 29
Counts vs. Acquisition Time (min)

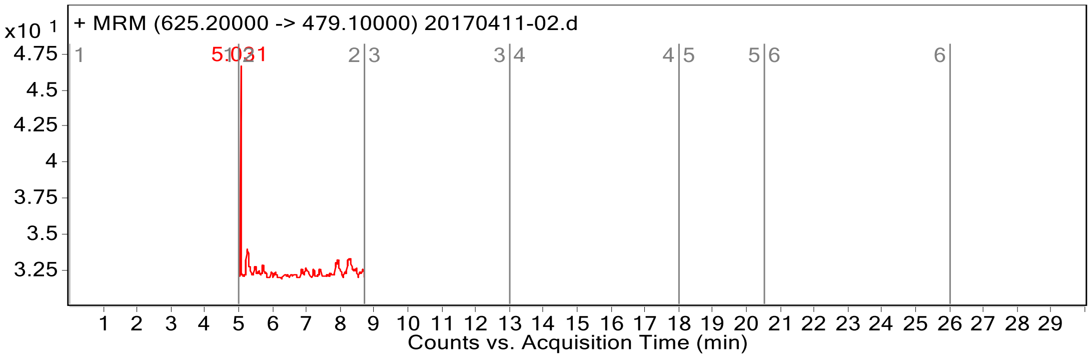
+ MRM (625.20000 -> 479.10000) 20170411-02.d
x10 1
5.031
Counts vs. Acquisition Time (min)

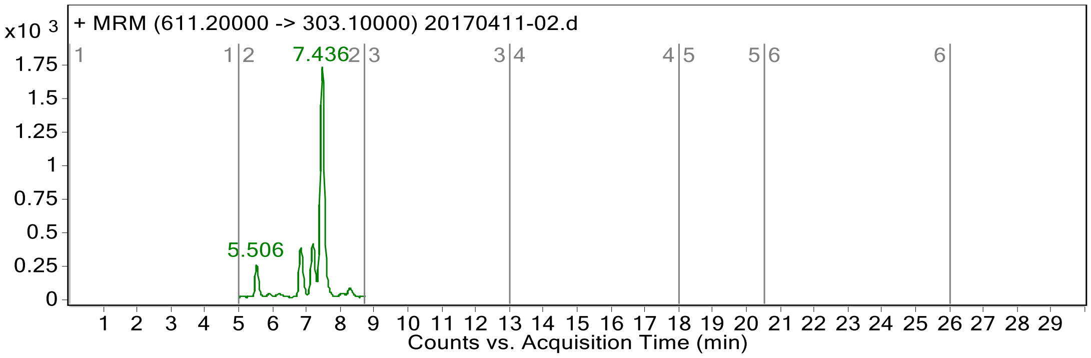
+ MRM (611.20000 -> 303.10000) 20170411-02.d
x10 3
7.436
5.506
Counts vs. Acquisition Time (min)

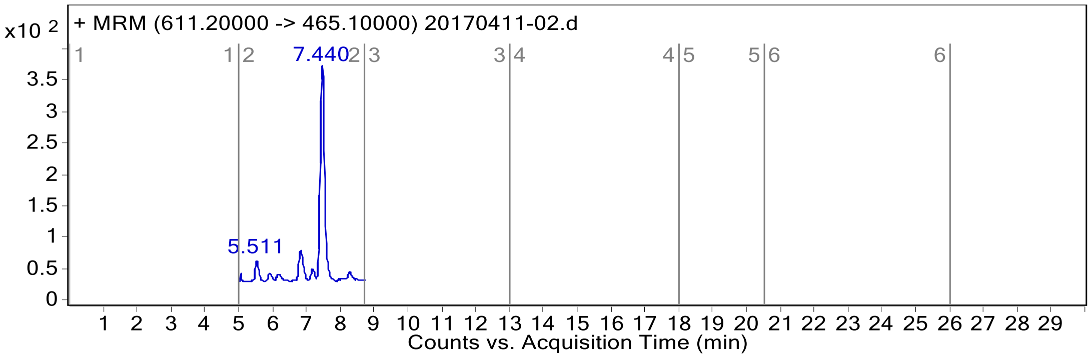
+ MRM (611.20000 -> 465.10000) 20170411-02.d
x10 2
7.440
5.511
Counts vs. Acquisition Time (min)

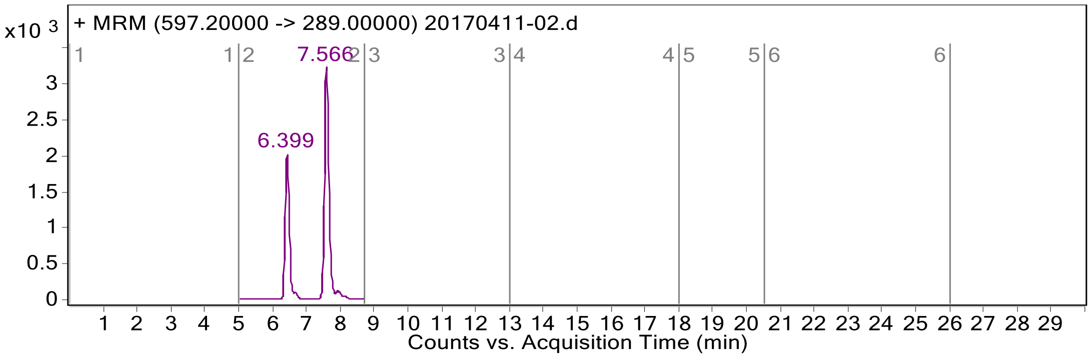
+ MRM (597.20000 -> 289.00000) 20170411-02.d
x10 3
7.566
6.399
Counts vs. Acquisition Time (min)

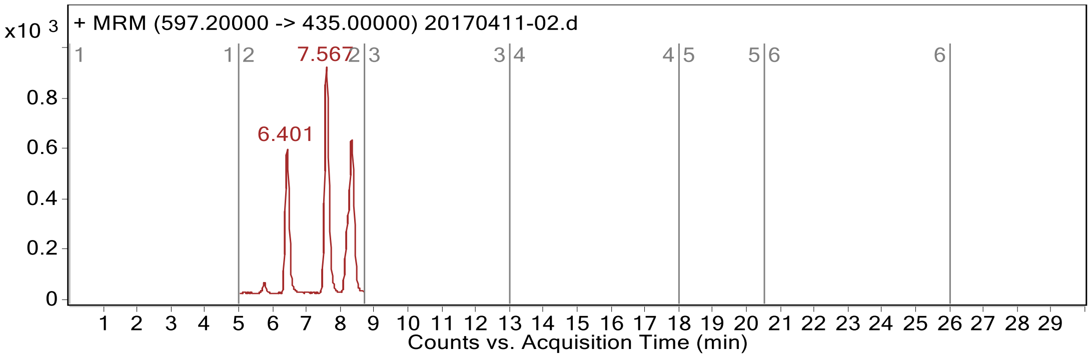
+ MRM (597.20000 -> 435.00000) 20170411-02.d
x10 3
7.567
6.401
Counts vs. Acquisition Time (min)

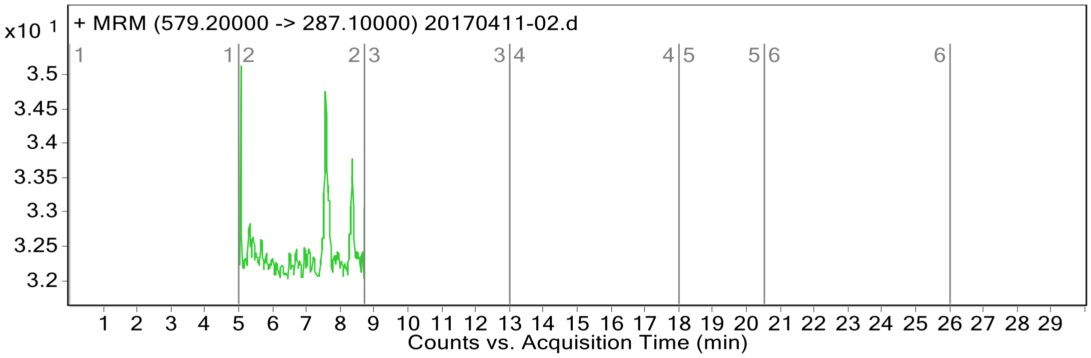
+ MRM (579.20000 -> 287.10000) 20170411-02.d
x10 1
Counts vs. Acquisition Time (min)

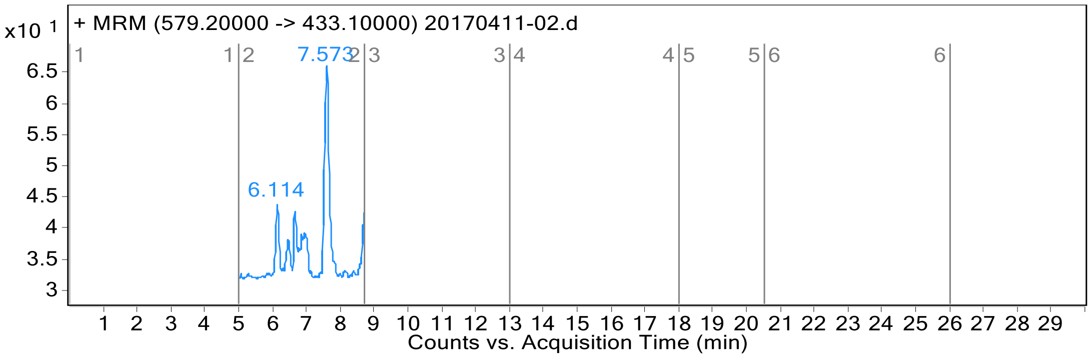
+ MRM (579.20000 -> 433.10000) 20170411-02.d
x10 1
7.573
6.114
Counts vs. Acquisition Time (min)

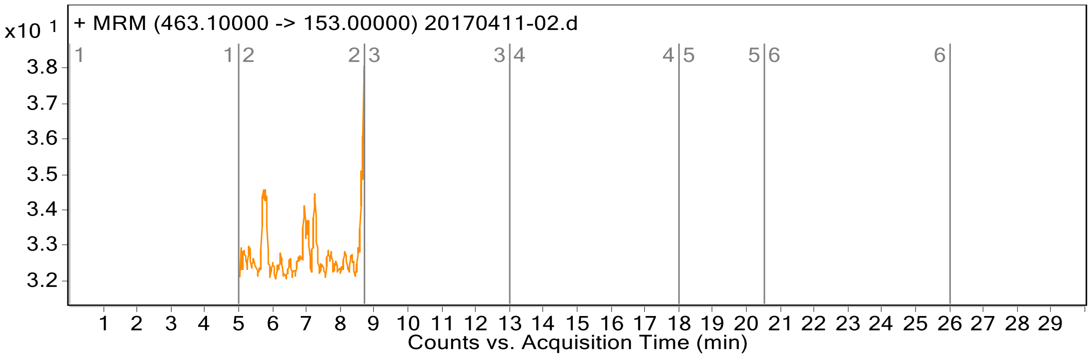
+ MRM (463.10000 -> 153.00000) 20170411-02.d
x10 1
Counts vs. Acquisition Time (min)

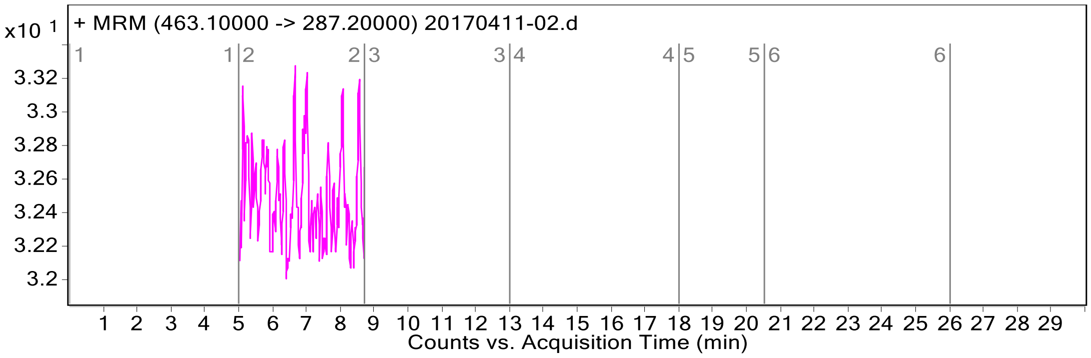
+ MRM (463.10000 -> 287.20000) 20170411-02.d
x10 1
Counts vs. Acquisition Time (min)

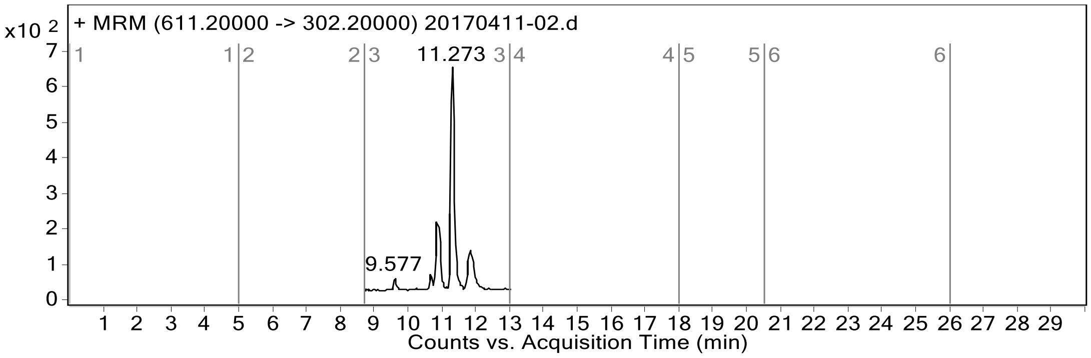
+ MRM (611.20000 -> 302.20000) 20170411-02.d
x10 2
11.273
9.577
Counts vs. Acquisition Time (min)

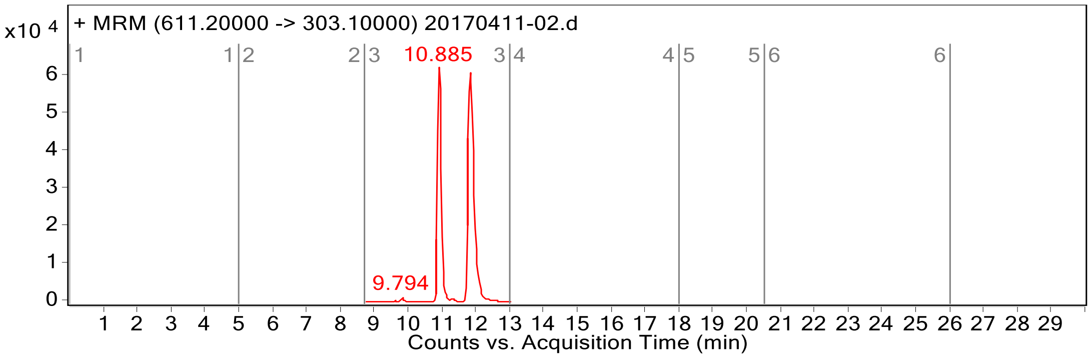
x10 4
+ MRM (611.20000 -> 303.10000) 20170411-02.d
10.885
9.794
Counts vs. Acquisition Time (min)

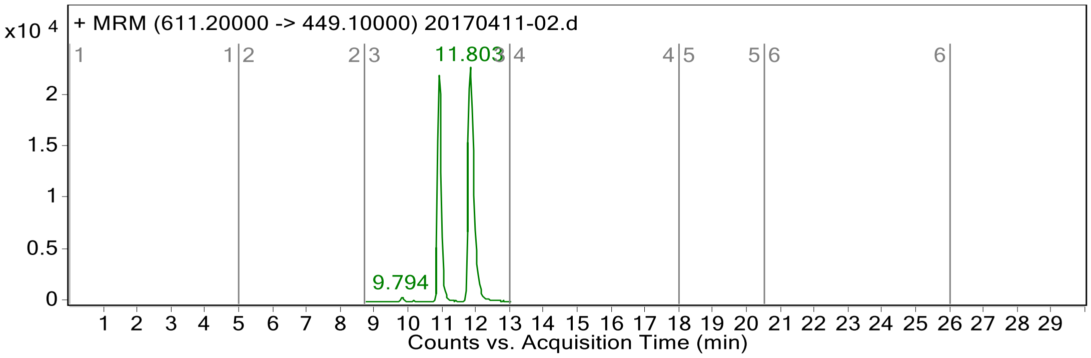
x10 4
+ MRM (611.20000 -> 449.10000) 20170411-02.d
11.803
9.794
Counts vs. Acquisition Time (min)

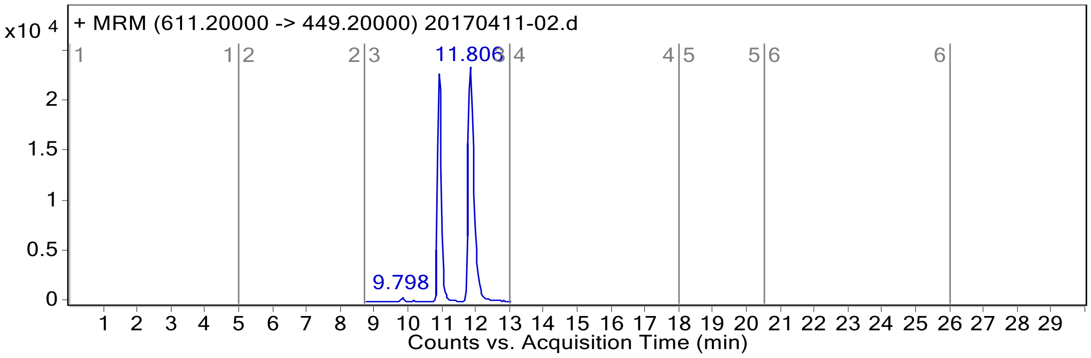
x10 4
+ MRM (611.20000 -> 449.20000) 20170411-02.d
11.806
9.798
Counts vs. Acquisition Time (min)

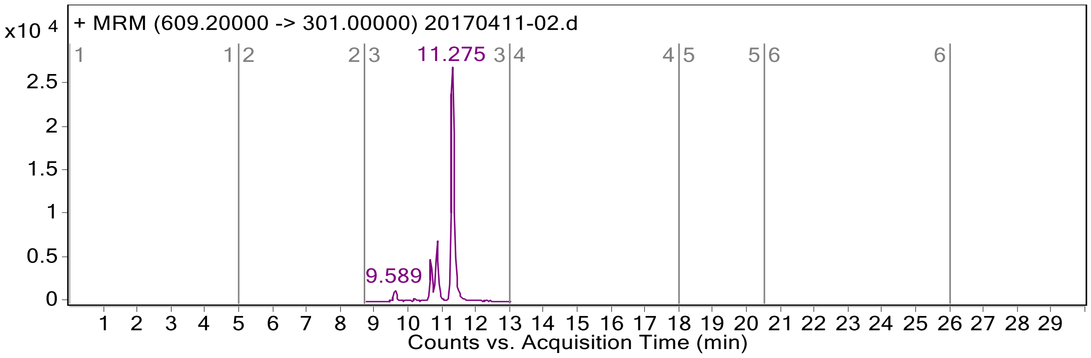
x10 4
+ MRM (609.20000 -> 301.00000) 20170411-02.d
11.275
9.589
Counts vs. Acquisition Time (min)

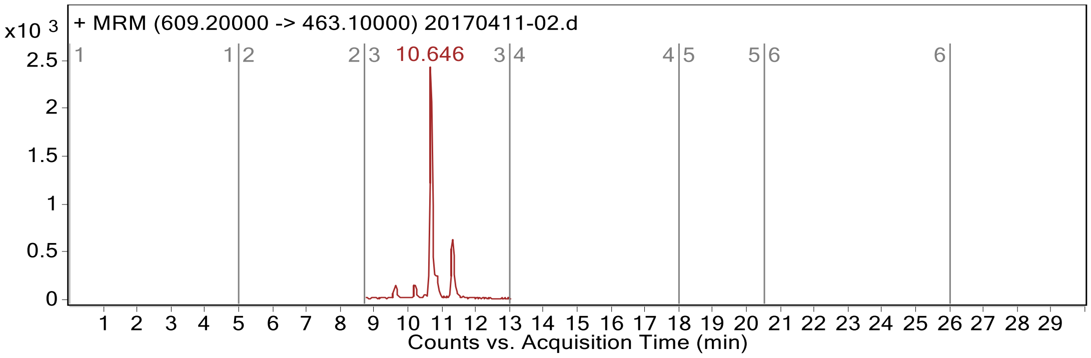
x10 3
+ MRM (609.20000 -> 463.10000) 20170411-02.d
10.646
Counts vs. Acquisition Time (min)

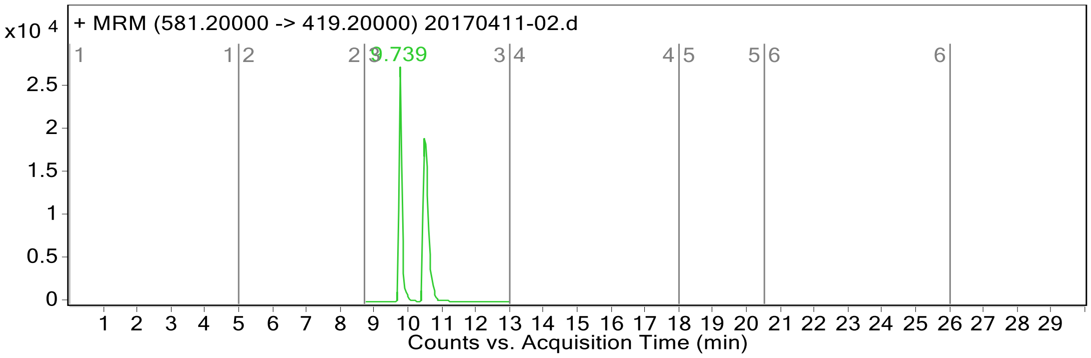
+ MRM (581.20000 -> 419.20000) 20170411-02.d
x10 4
9.739
Counts vs. Acquisition Time (min)

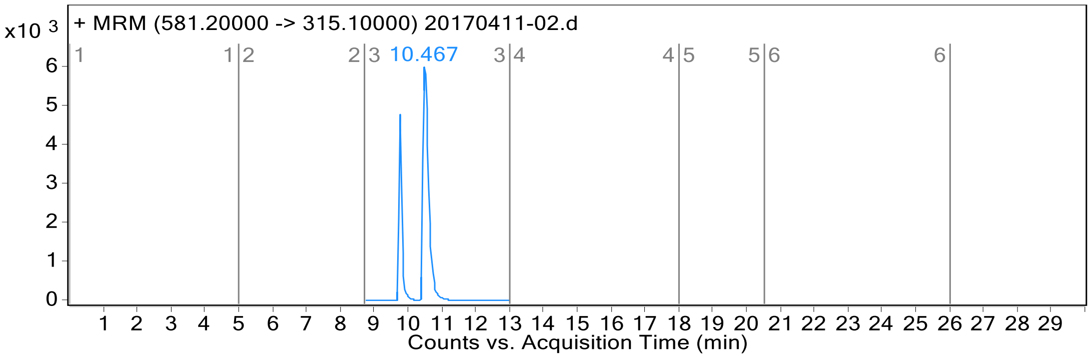
+ MRM (581.20000 -> 315.10000) 20170411-02.d
x10 3
10.467
Counts vs. Acquisition Time (min)

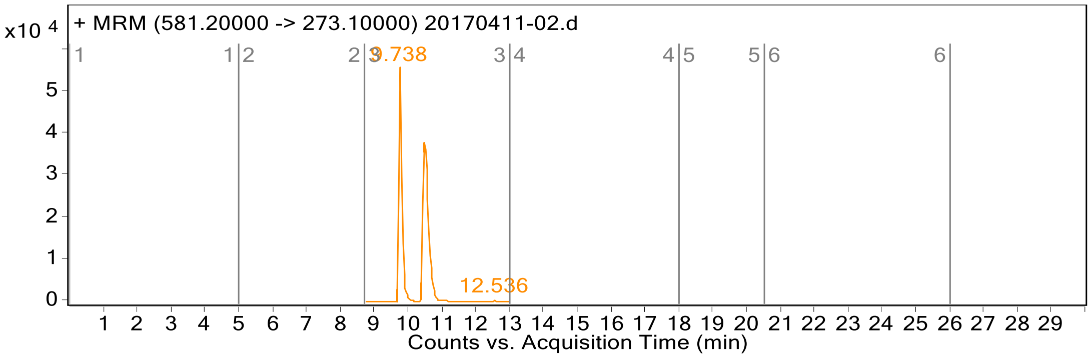
+ MRM (581.20000 -> 273.10000) 20170411-02.d
x10 4
9.738
12.536
Counts vs. Acquisition Time (min)

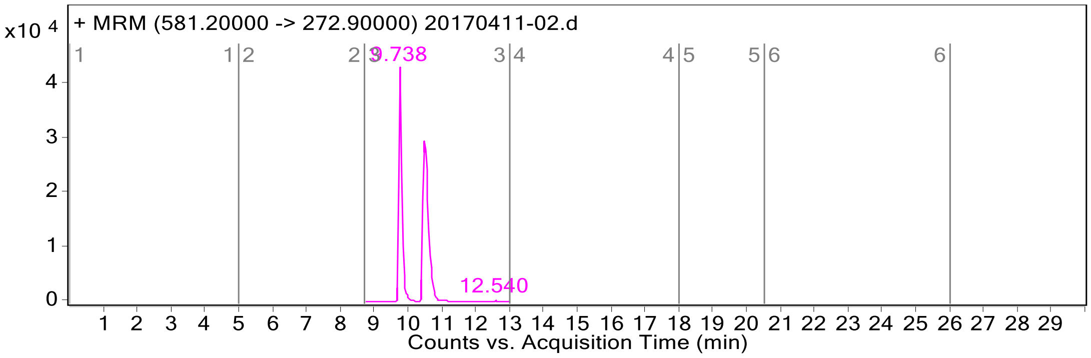
+ MRM (581.20000 -> 272.90000) 20170411-02.d
x10 4
9.738
12.540
Counts vs. Acquisition Time (min)

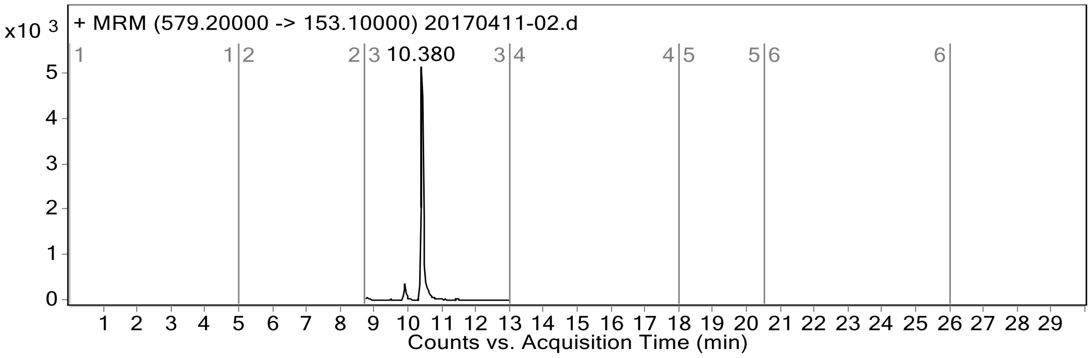
+ MRM (579.20000 -> 153.10000) 20170411-02.d
x10 3
10.380
Counts vs. Acquisition Time (min)

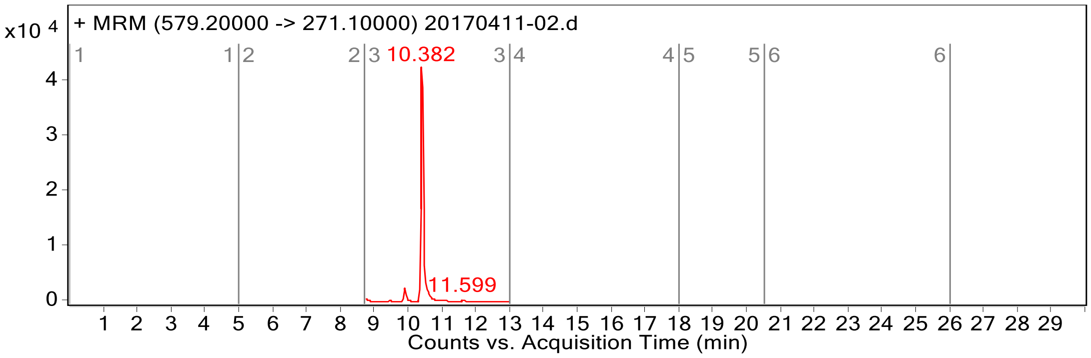
x10 4
+ MRM (579.20000 -> 271.10000) 20170411-02.d
10.382
11.599
Counts vs. Acquisition Time (min)

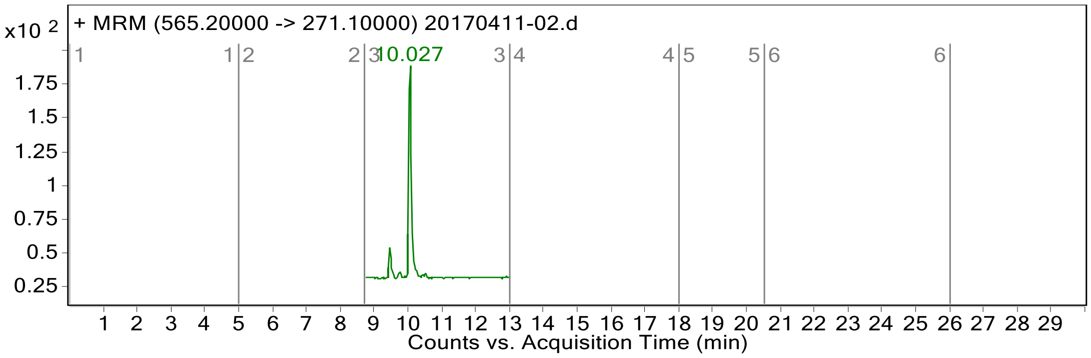
x10 2
+ MRM (565.20000 -> 271.10000) 20170411-02.d
10.027
Counts vs. Acquisition Time (min)

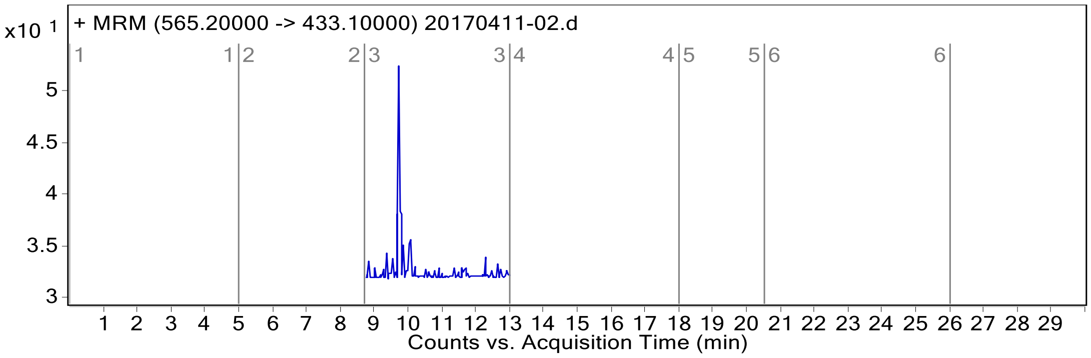
x10 1
+ MRM (565.20000 -> 433.10000) 20170411-02.d
Counts vs. Acquisition Time (min)

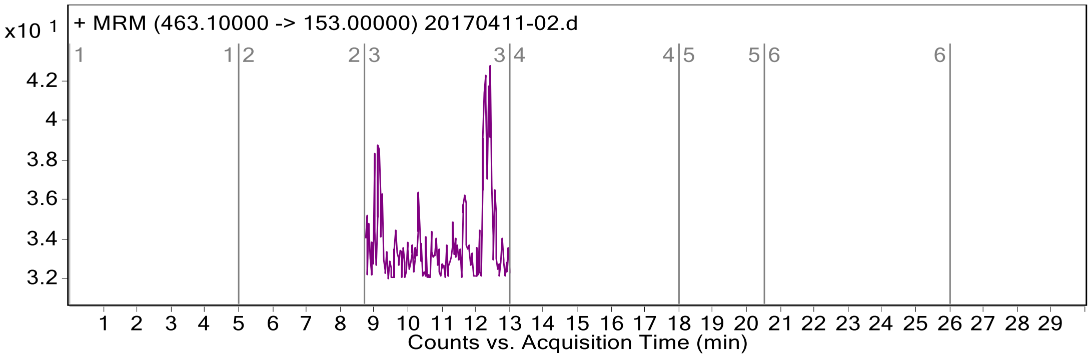
x10 1
+ MRM (463.10000 -> 153.00000) 20170411-02.d
Counts vs. Acquisition Time (min)

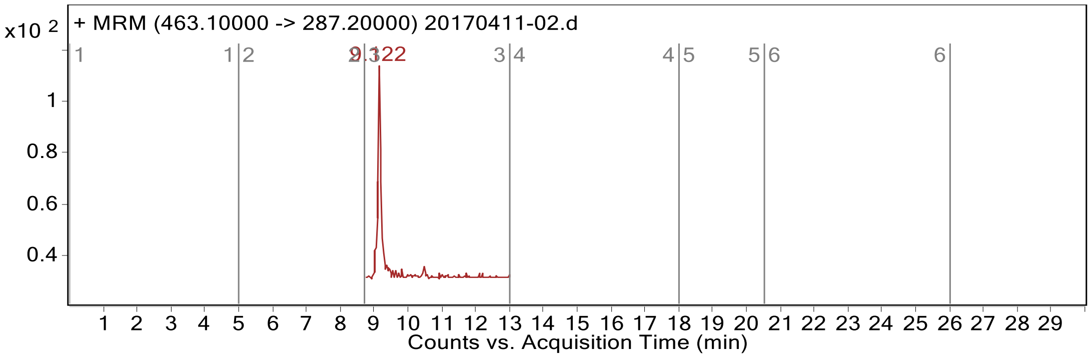
x10 2
+ MRM (463.10000 -> 287.20000) 20170411-02.d
9.122
Counts vs. Acquisition Time (min)

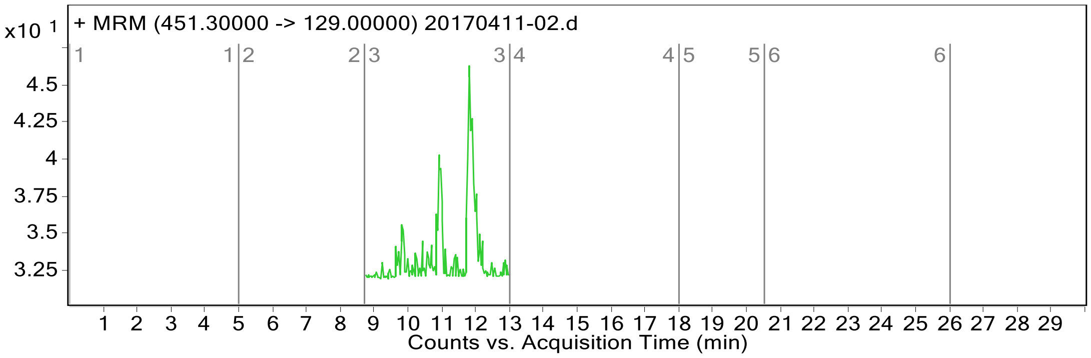
x10 1
+ MRM (451.30000 -> 129.00000) 20170411-02.d
Counts vs. Acquisition Time (min)

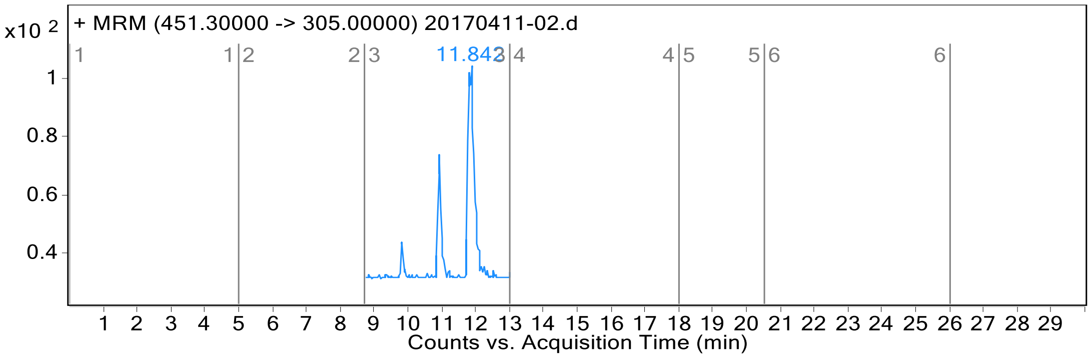
x10 2
+ MRM (451.30000 -> 305.00000) 20170411-02.d
11.842
Counts vs. Acquisition Time (min)

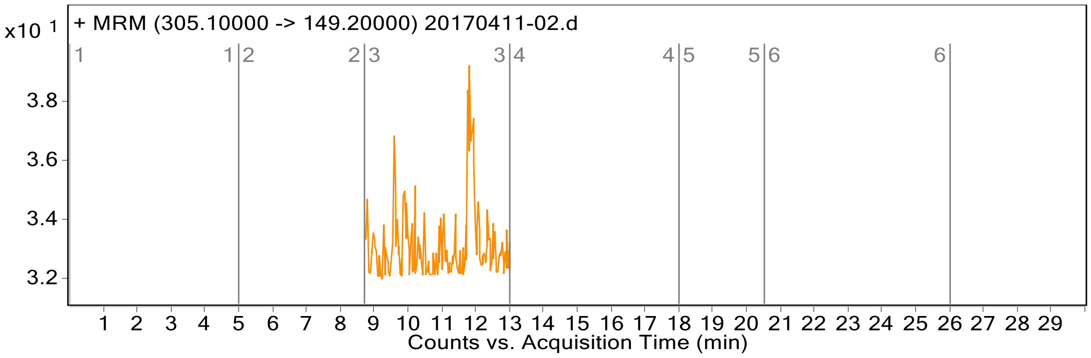
x10 1
+ MRM (305.10000 -> 149.20000) 20170411-02.d
Counts vs. Acquisition Time (min)

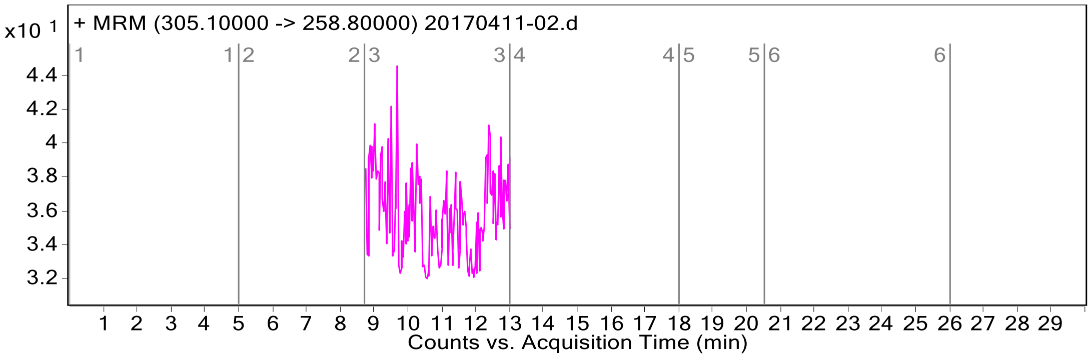
x10 1
+ MRM (305.10000 -> 258.80000) 20170411-02.d
Counts vs. Acquisition Time (min)

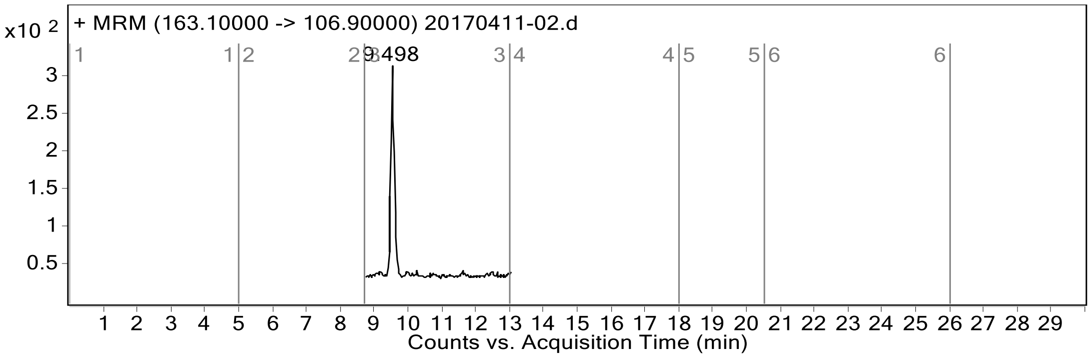
x10 2
+ MRM (163.10000 -> 106.90000) 20170411-02.d
9.498
Counts vs. Acquisition Time (min)

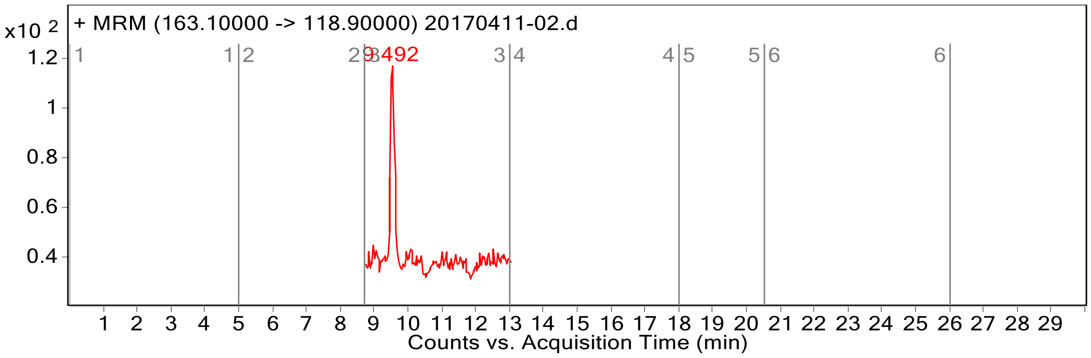
+ MRM (163.10000 -> 118.90000) 20170411-02.d
Counts vs. Acquisition Time (min)

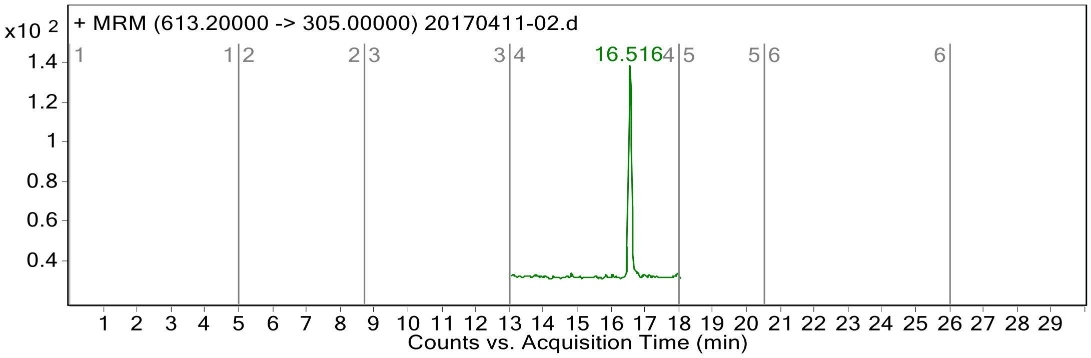
+ MRM (613.20000 -> 305.00000) 20170411-02.d
16.516
Counts vs. Acquisition Time (min)

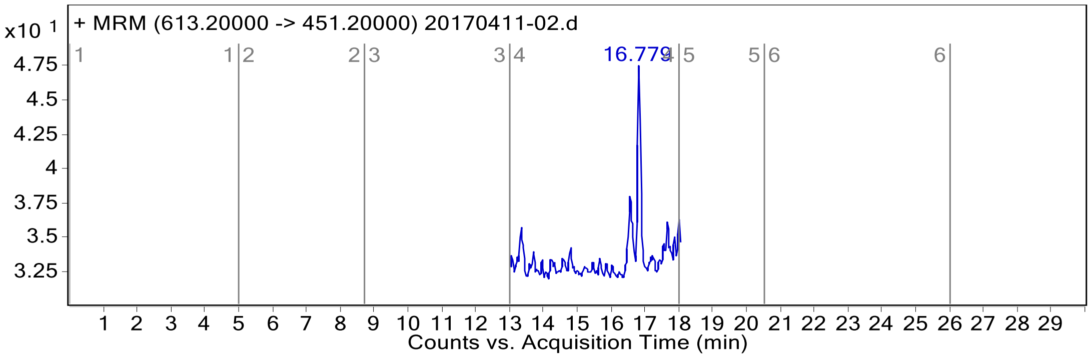
+ MRM (613.20000 -> 451.20000) 20170411-02.d
16.779
Counts vs. Acquisition Time (min)

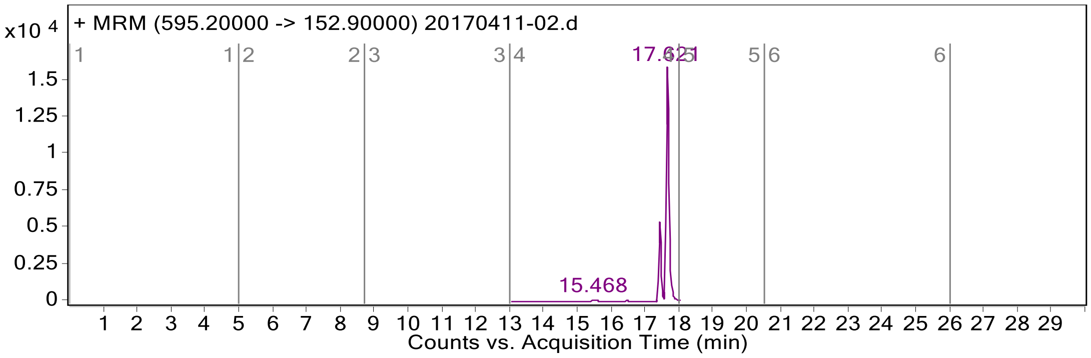
+ MRM (595.20000 -> 152.90000) 20170411-02.d
15.468
Counts vs. Acquisition Time (min)

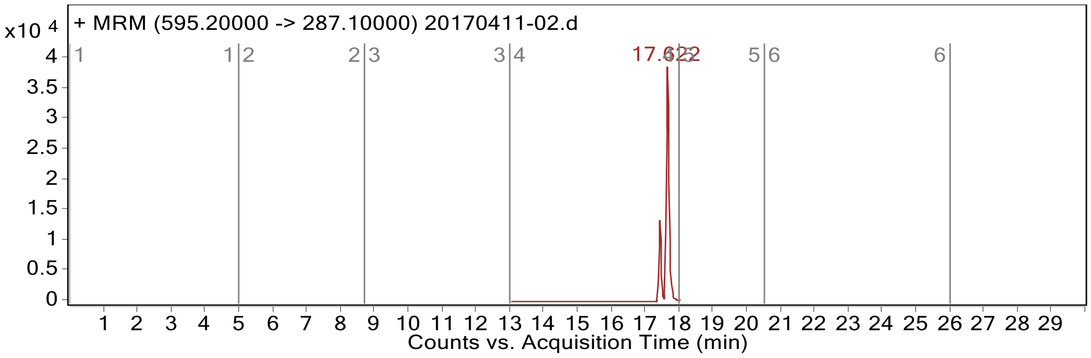
+ MRM (595.20000 -> 287.10000) 20170411-02.d
Counts vs. Acquisition Time (min)

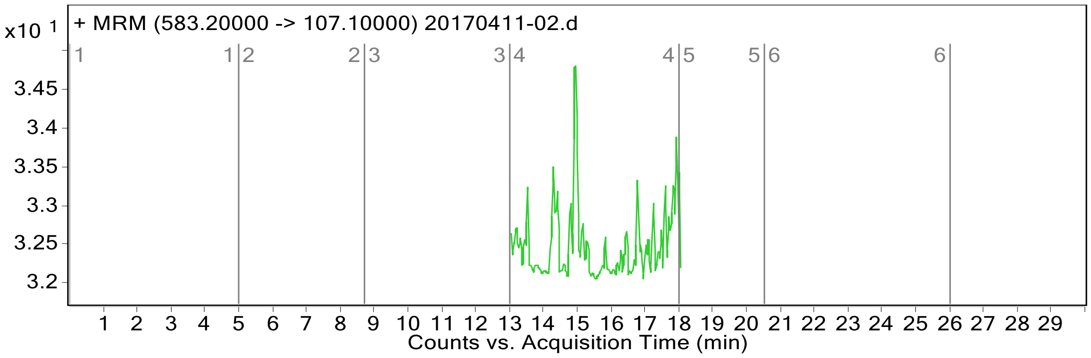
x10 1
+ MRM (583.20000 -> 107.10000) 20170411-02.d
Counts vs. Acquisition Time (min)

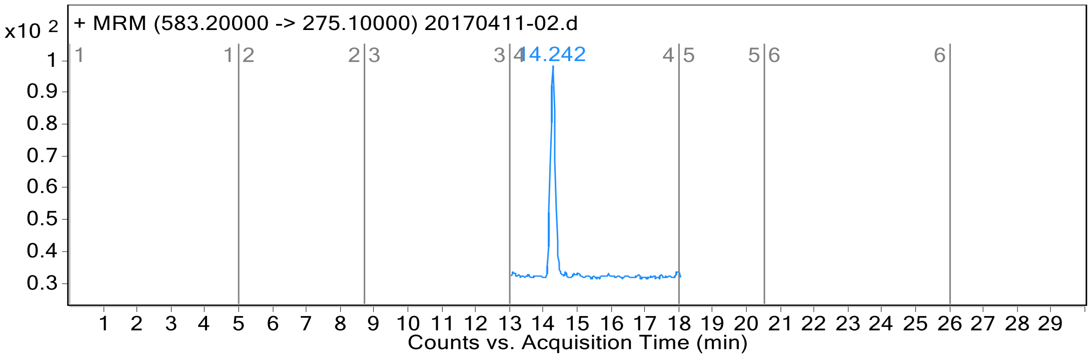
x10 2
+ MRM (583.20000 -> 275.10000) 20170411-02.d
14.242
Counts vs. Acquisition Time (min)

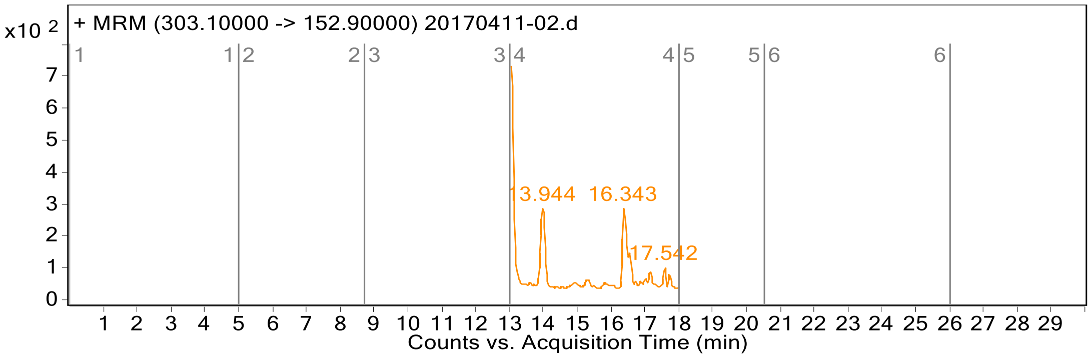
x10 2
+ MRM (303.10000 -> 152.90000) 20170411-02.d
13.944
16.343
17.542
Counts vs. Acquisition Time (min)

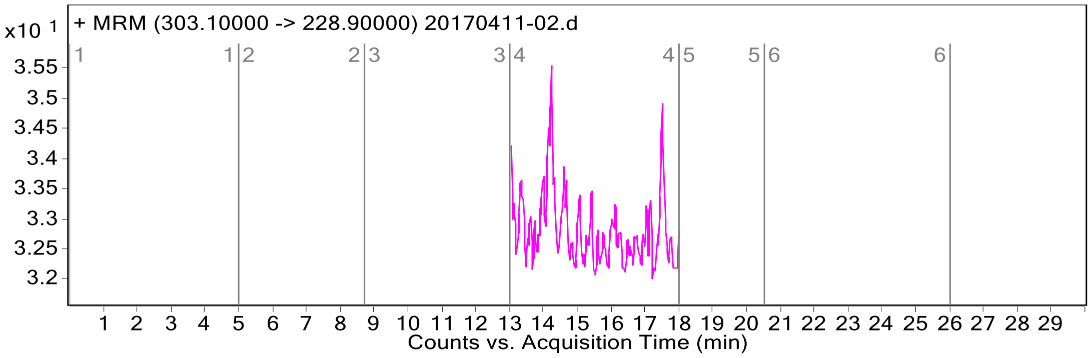
x10 1
+ MRM (303.10000 -> 228.90000) 20170411-02.d
Counts vs. Acquisition Time (min)

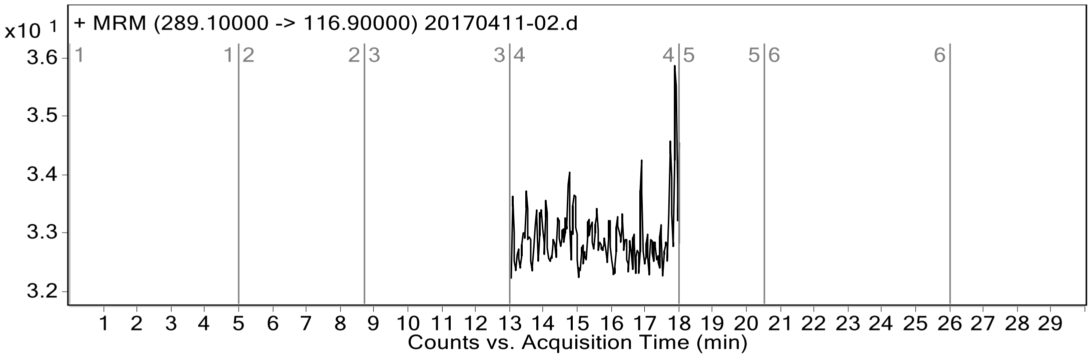
x10 1
+ MRM (289.10000 -> 116.90000) 20170411-02.d
Counts vs. Acquisition Time (min)

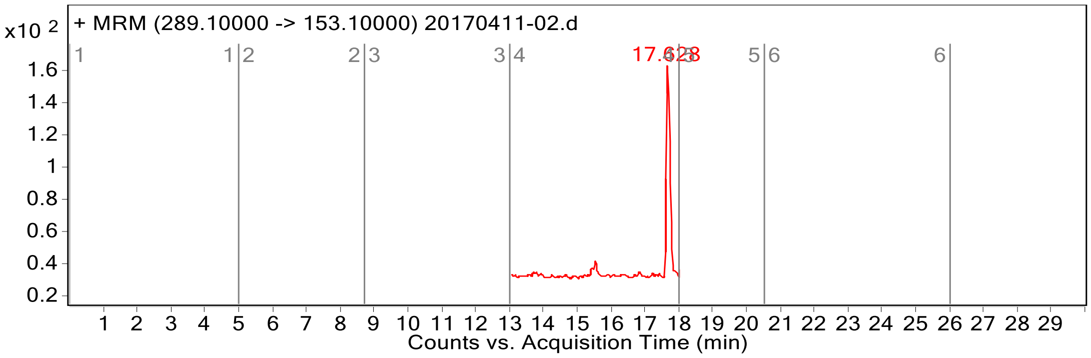

x10 2
+ MRM (289.10000 -> 153.10000) 20170411-02.d
Counts vs. Acquisition Time (min)

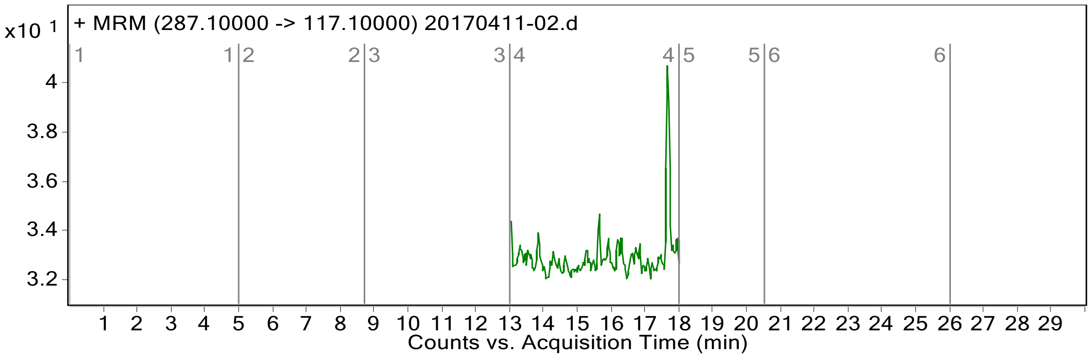

x10 1
+ MRM (287.10000 -> 117.10000) 20170411-02.d
Counts vs. Acquisition Time (min)

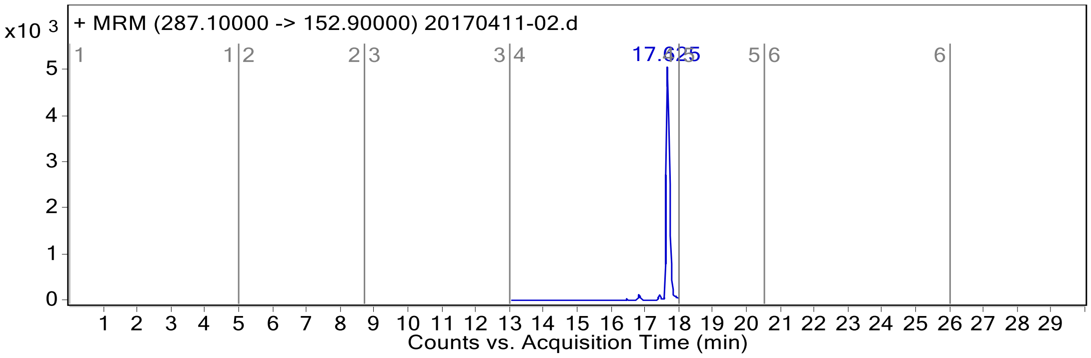

x10 3
+ MRM (287.10000 -> 152.90000) 20170411-02.d
Counts vs. Acquisition Time (min)

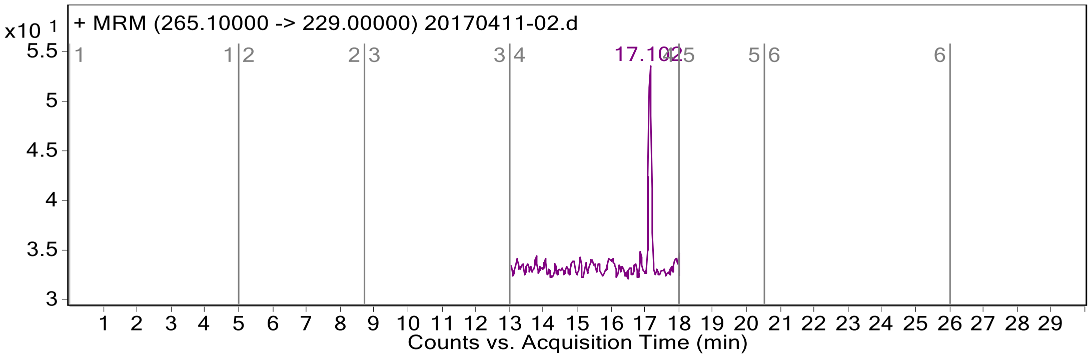

x10 1
+ MRM (265.10000 -> 229.00000) 20170411-02.d
Counts vs. Acquisition Time (min)

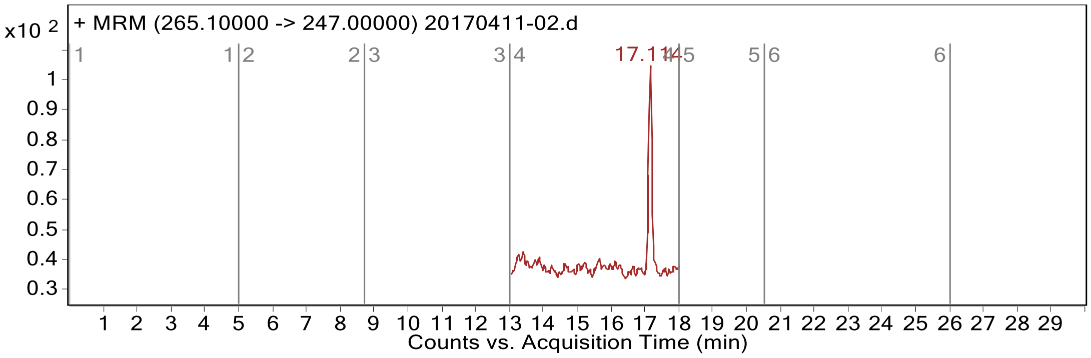

x10 2
+ MRM (265.10000 -> 247.00000) 20170411-02.d
Counts vs. Acquisition Time (min)

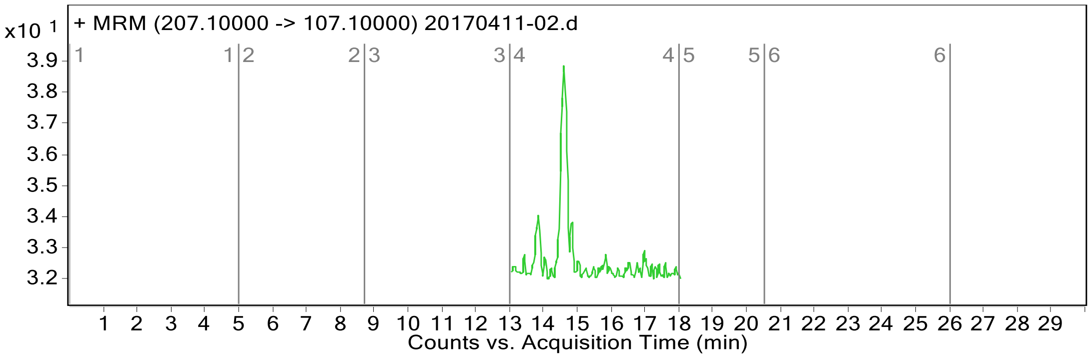
+ MRM (207.10000 -> 107.10000) 20170411-02.d
x10 1
Counts vs. Acquisition Time (min)

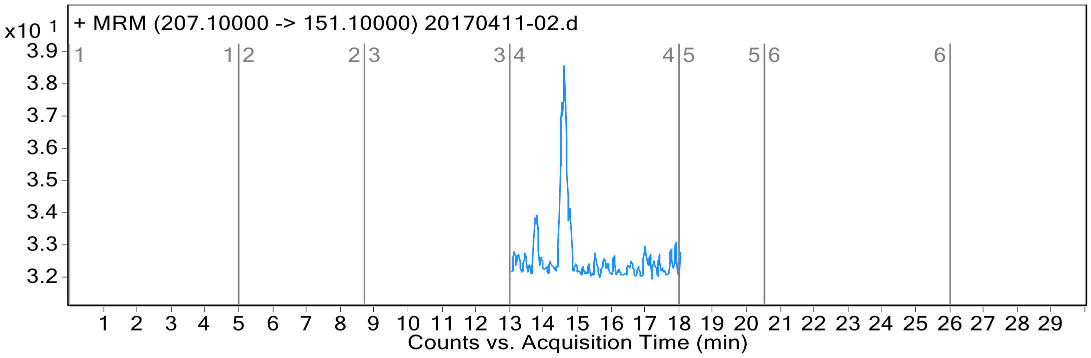
+ MRM (207.10000 -> 151.10000) 20170411-02.d
x10 1
Counts vs. Acquisition Time (min)

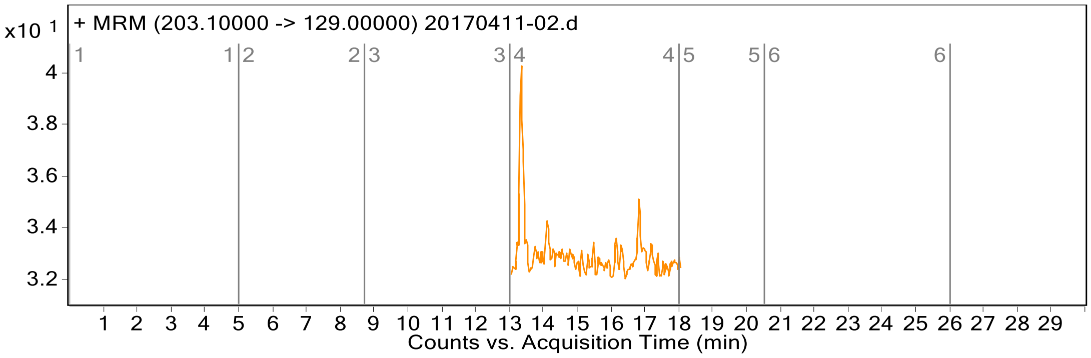
+ MRM (203.10000 -> 129.00000) 20170411-02.d
x10 1
Counts vs. Acquisition Time (min)

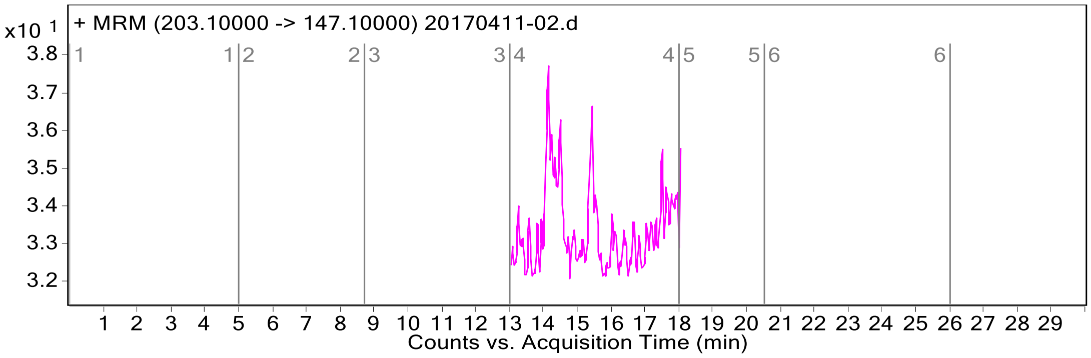
+ MRM (203.10000 -> 147.10000) 20170411-02.d
x10 1
Counts vs. Acquisition Time (min)

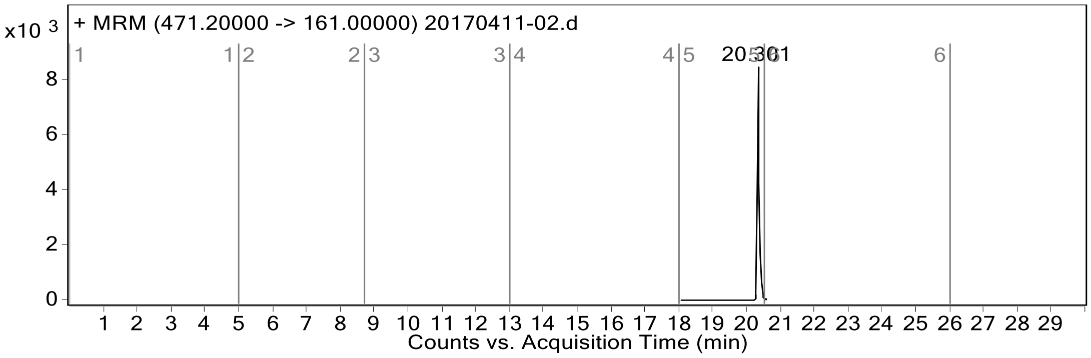
+ MRM (471.20000 -> 161.00000) 20170411-02.d
x10 3
Counts vs. Acquisition Time (min)

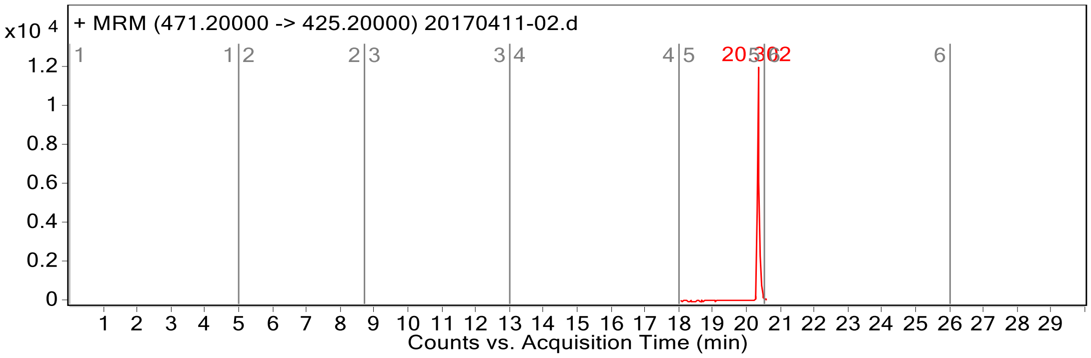
+ MRM (471.20000 -> 425.20000) 20170411-02.d
x10 4
Counts vs. Acquisition Time (min)

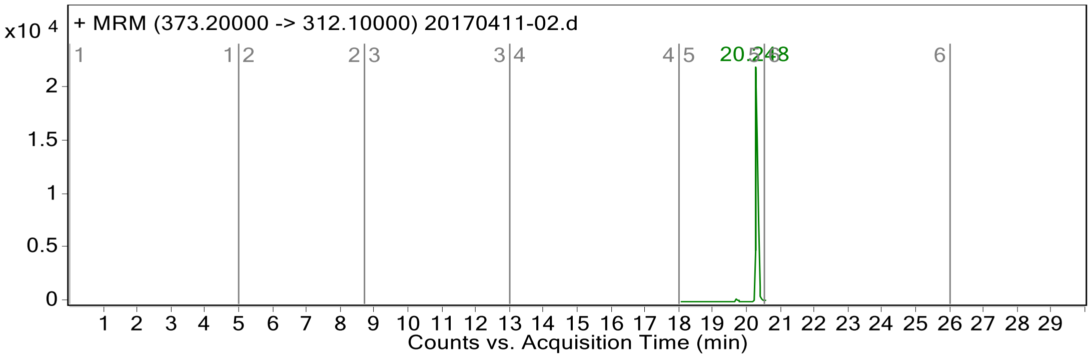
+ MRM (373.20000 -> 312.10000) 20170411-02.d
x10 4
Counts vs. Acquisition Time (min)

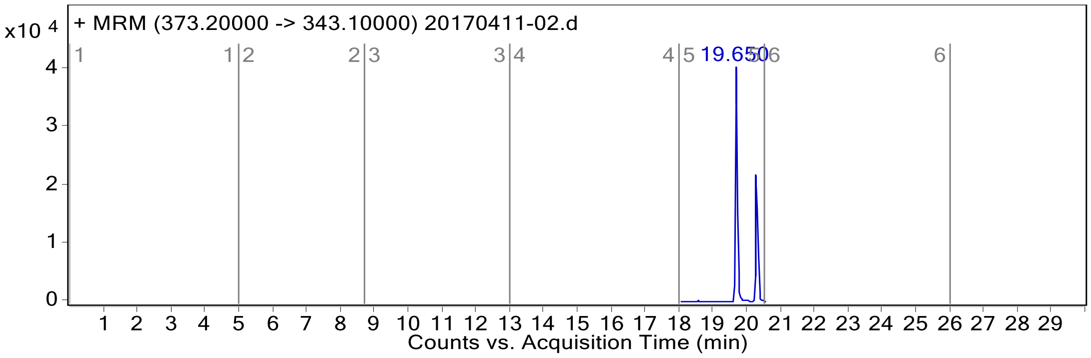
+ MRM (373.20000 -> 343.10000) 20170411-02.d
x10 4
19.650
Counts vs. Acquisition Time (min)

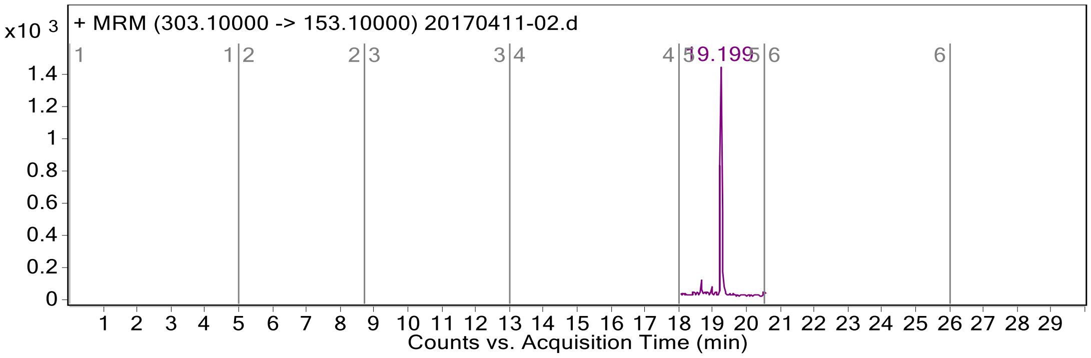
+ MRM (303.10000 -> 153.10000) 20170411-02.d
x10 3
19.199
Counts vs. Acquisition Time (min)

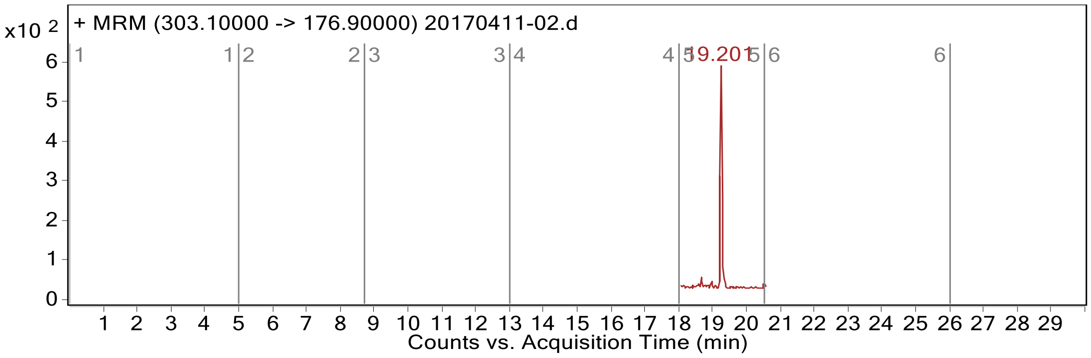
+ MRM (303.10000 -> 176.90000) 20170411-02.d
x10 2
19.201
Counts vs. Acquisition Time (min)

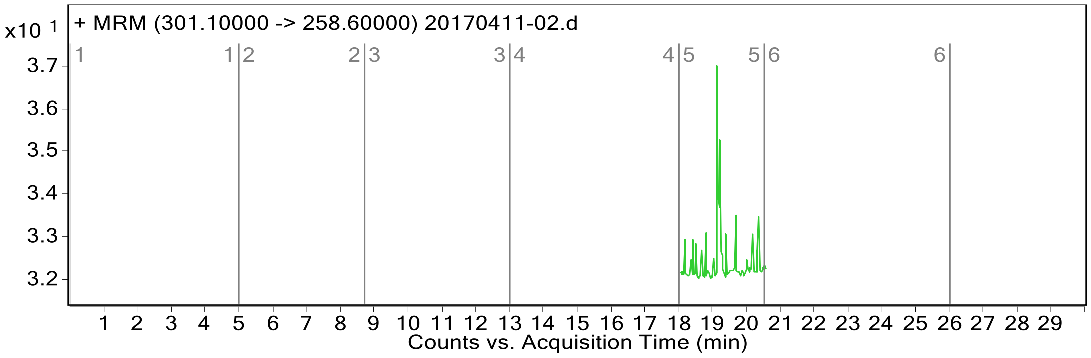

+ MRM (301.10000 -> 258.60000) 20170411-02.d
x10 1
Counts vs. Acquisition Time (min)

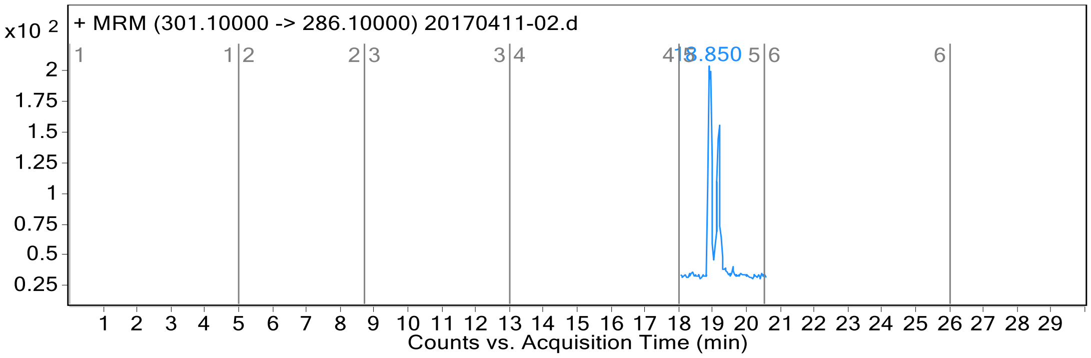

+ MRM (301.10000 -> 286.10000) 20170411-02.d
x10 2
18.850
Counts vs. Acquisition Time (min)

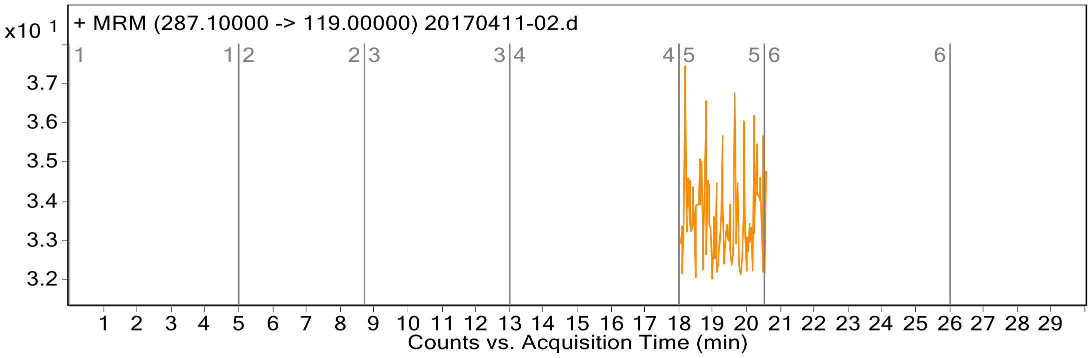

+ MRM (287.10000 -> 119.00000) 20170411-02.d
x10 1
Counts vs. Acquisition Time (min)

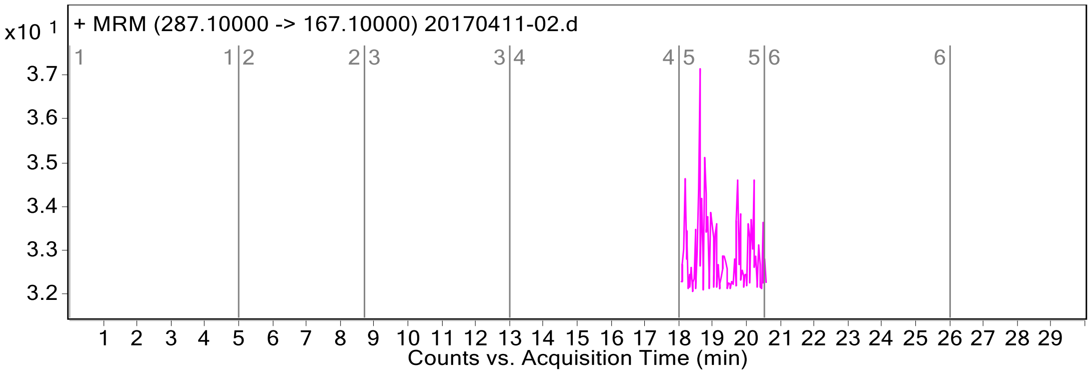

+ MRM (287.10000 -> 167.10000) 20170411-02.d
x10 1
Counts vs. Acquisition Time (min)

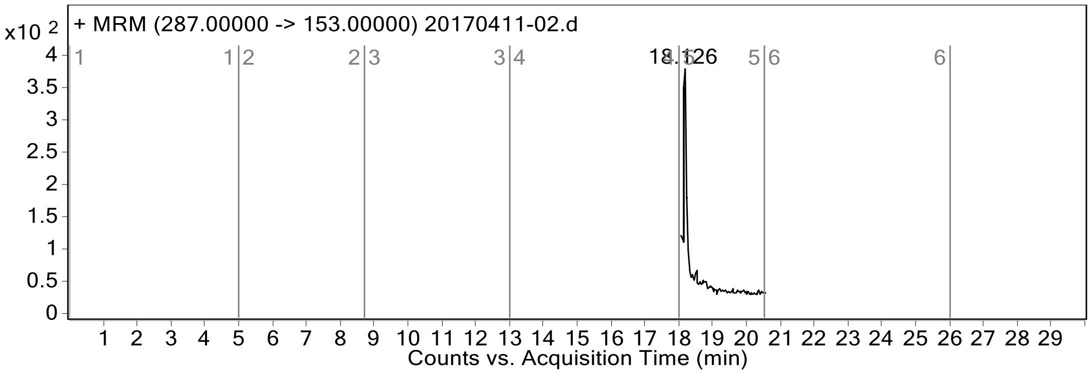
x10 2
+ MRM (287.00000 -> 153.00000) 20170411-02.d
18.126
Counts vs. Acquisition Time (min)

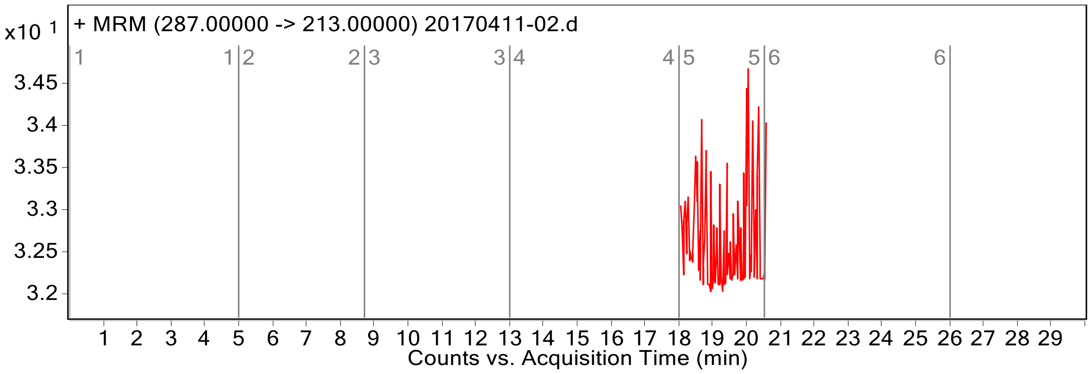
x10 1
+ MRM (287.00000 -> 213.00000) 20170411-02.d
Counts vs. Acquisition Time (min)

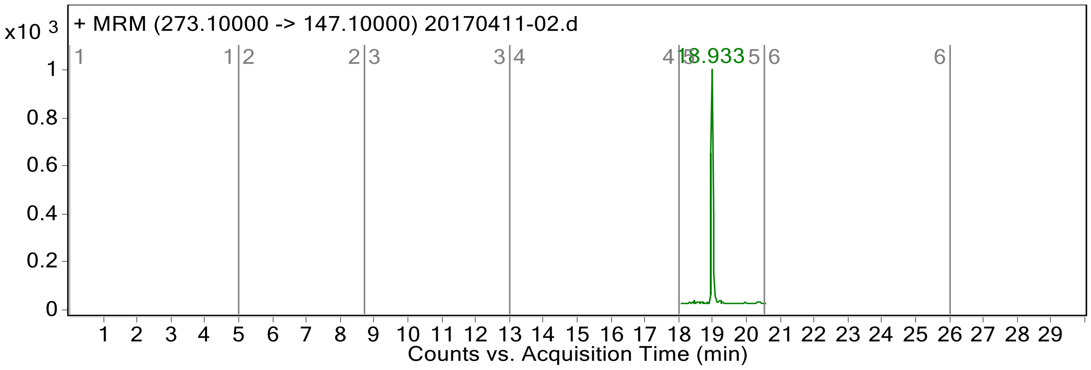
x10 3
+ MRM (273.10000 -> 147.10000) 20170411-02.d
18.933
Counts vs. Acquisition Time (min)

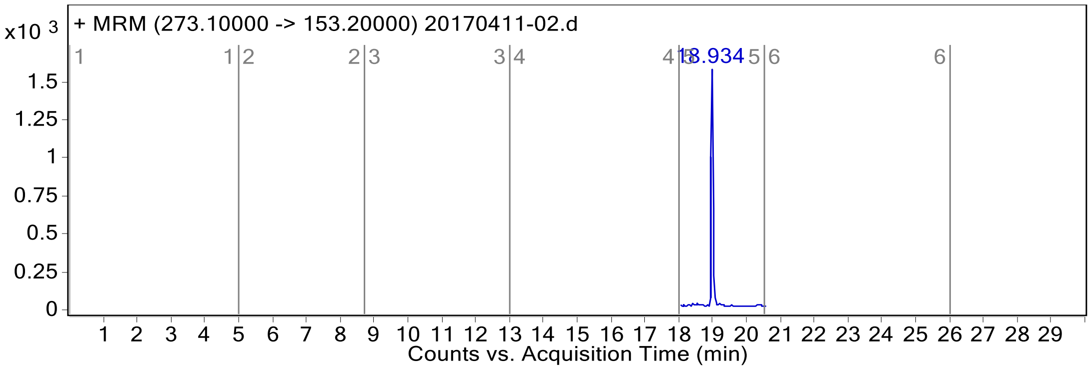
x10 3
+ MRM (273.10000 -> 153.20000) 20170411-02.d
18.934
Counts vs. Acquisition Time (min)

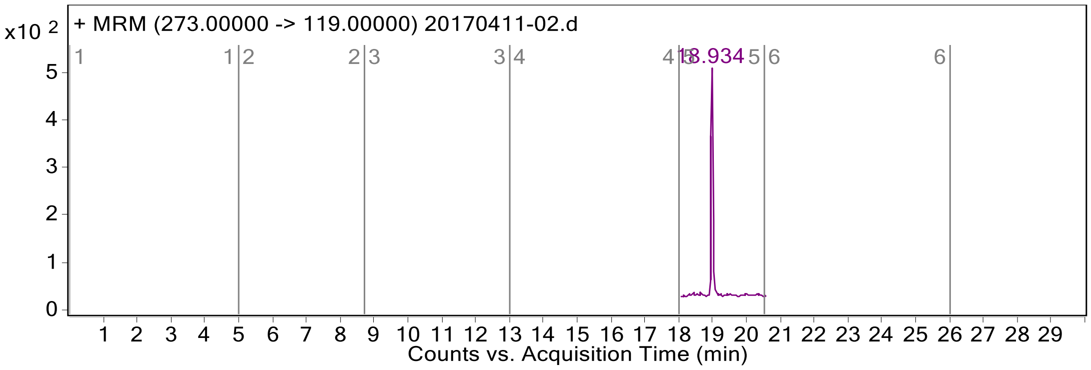
x10 2
+ MRM (273.00000 -> 119.00000) 20170411-02.d
18.934
Counts vs. Acquisition Time (min)

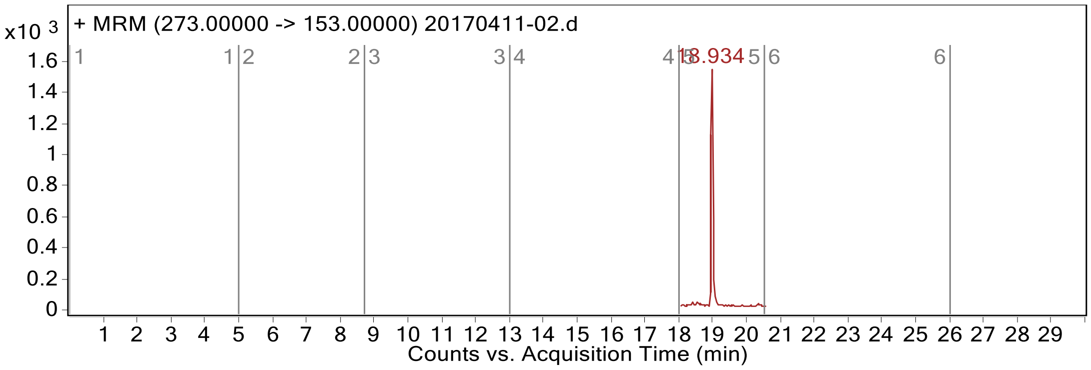
x10 3
+ MRM (273.00000 -> 153.00000) 20170411-02.d
18.934
Counts vs. Acquisition Time (min)

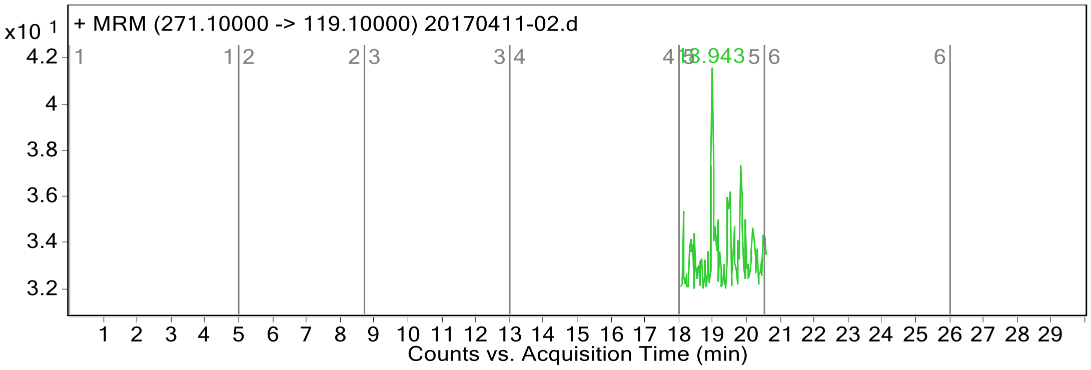
x10 1
+ MRM (271.10000 -> 119.10000) 20170411-02.d
18.943
Counts vs. Acquisition Time (min)

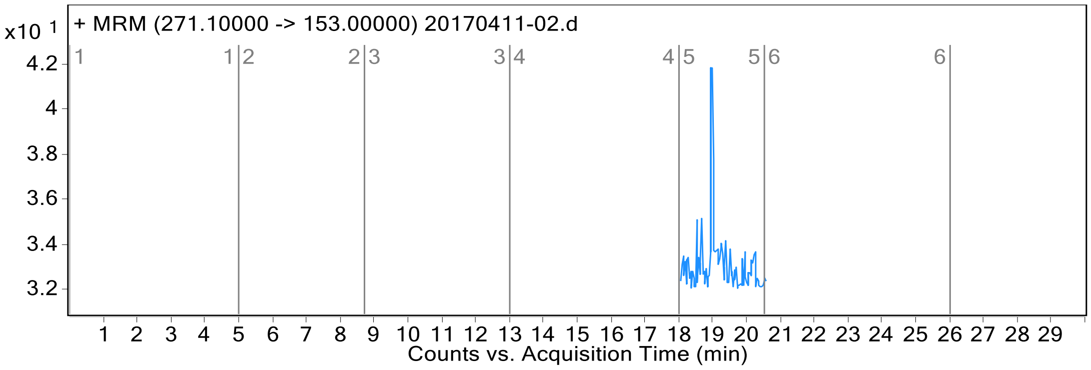
x10 1
+ MRM (271.10000 -> 153.00000) 20170411-02.d
Counts vs. Acquisition Time (min)

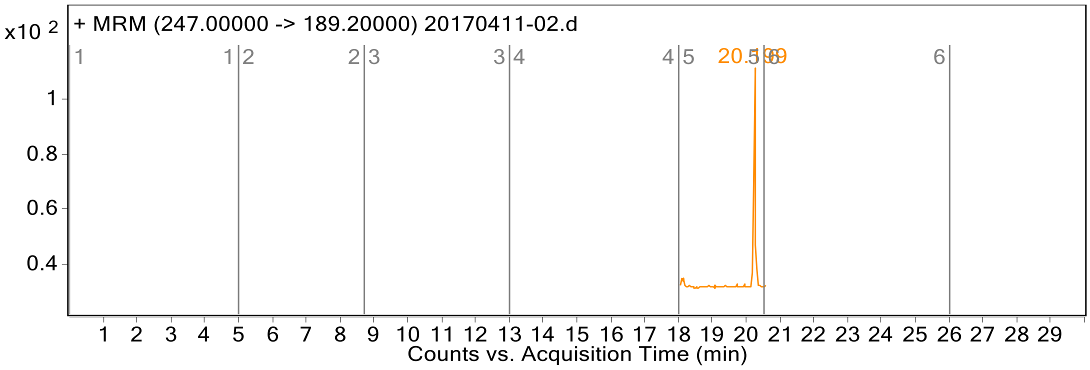
x10 2
+ MRM (247.00000 -> 189.20000) 20170411-02.d
Counts vs. Acquisition Time (min)

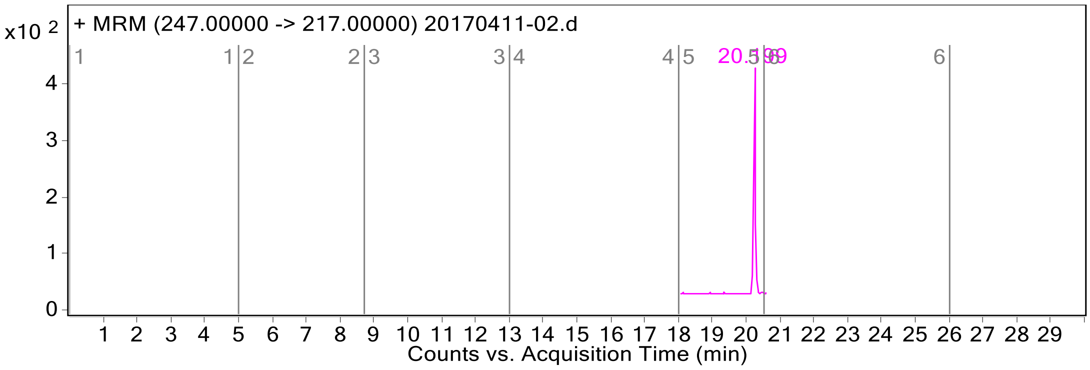
x10 2
+ MRM (247.00000 -> 217.00000) 20170411-02.d
Counts vs. Acquisition Time (min)

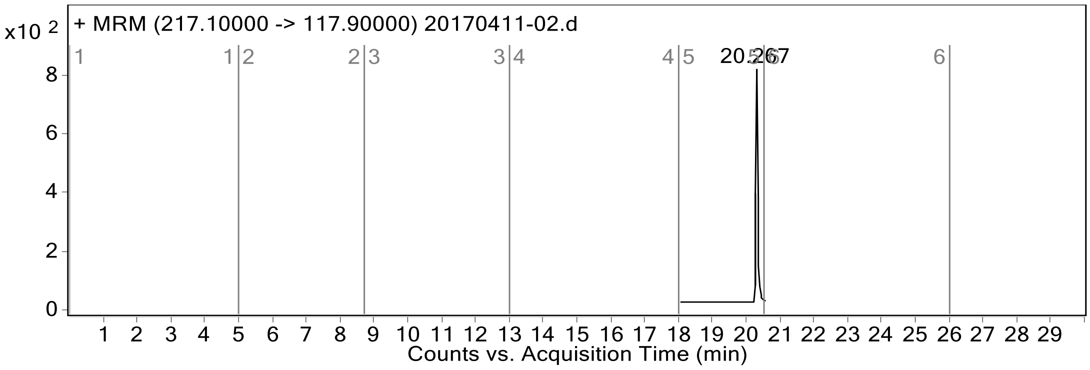
x10 2
+ MRM (217.10000 -> 117.90000) 20170411-02.d
Counts vs. Acquisition Time (min)

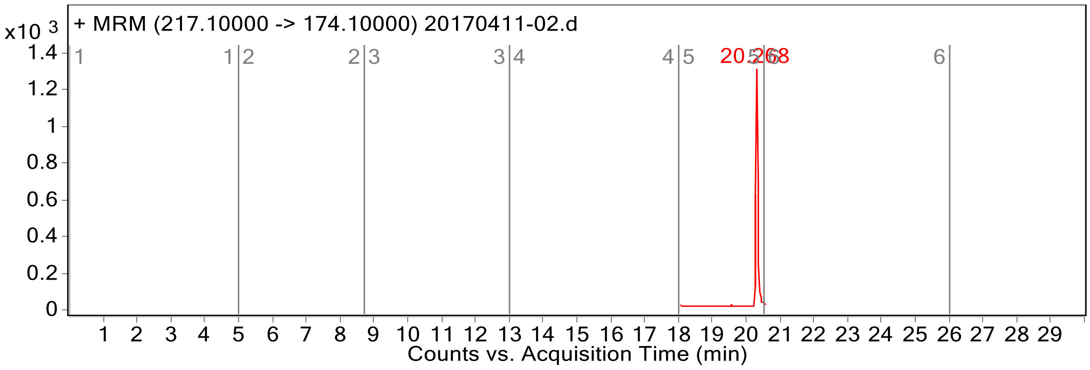
x10 3
+ MRM (217.10000 -> 174.10000) 20170411-02.d
Counts vs. Acquisition Time (min)

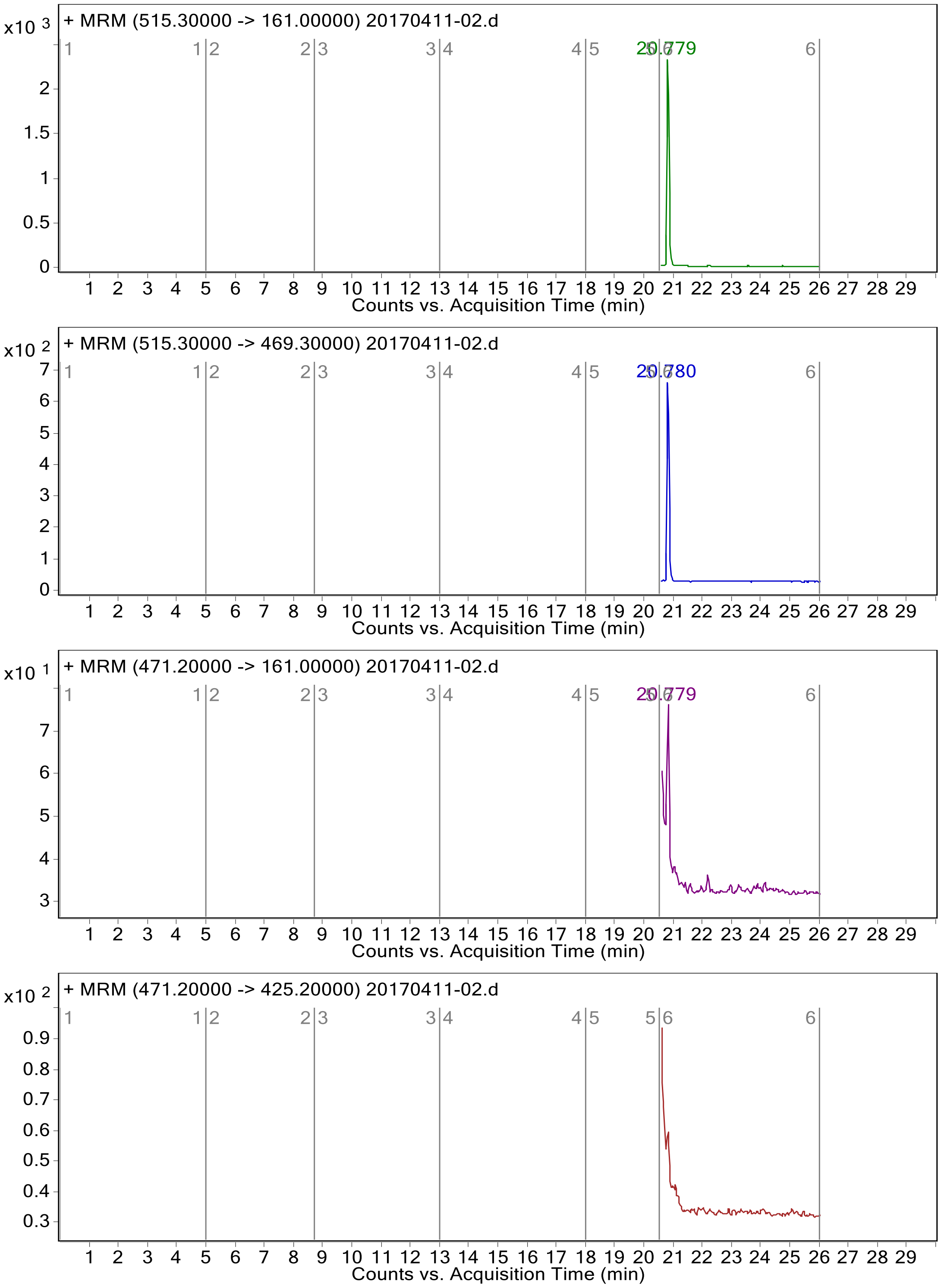

x10 3
+ MRM (515.30000 -> 161.00000) 20170411-02.d
20.779
Counts vs. Acquisition Time (min)
x10 2
+ MRM (515.30000 -> 469.30000) 20170411-02.d
20.780
Counts vs. Acquisition Time (min)
x10 1
+ MRM (471.20000 -> 161.00000) 20170411-02.d
20.779
Counts vs. Acquisition Time (min)
x10 2
+ MRM (471.20000 -> 425.20000) 20170411-02.d
Counts vs. Acquisition Time (min)

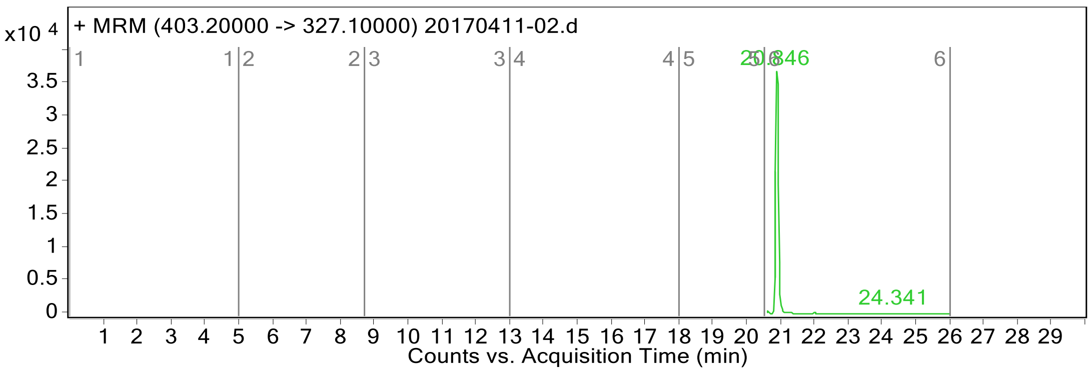

+ MRM (403.20000 -> 327.10000) 20170411-02.d
x10 4
20.846
24.341
Counts vs. Acquisition Time (min)

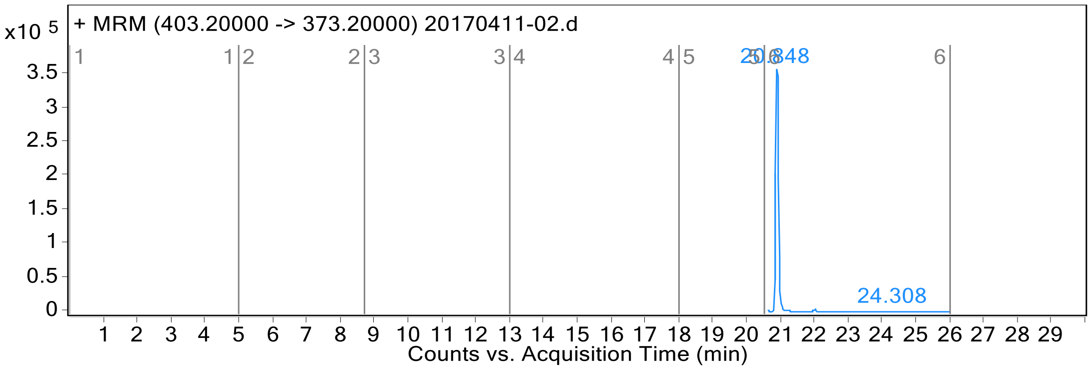

+ MRM (403.20000 -> 373.20000) 20170411-02.d
x10 5
20.848
24.308
Counts vs. Acquisition Time (min)

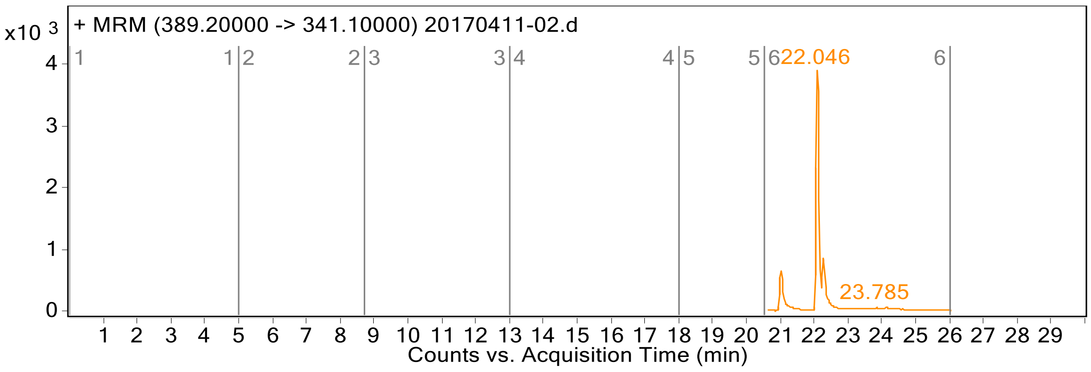

+ MRM (389.20000 -> 341.10000) 20170411-02.d
x10 3
22.046
23.785
Counts vs. Acquisition Time (min)

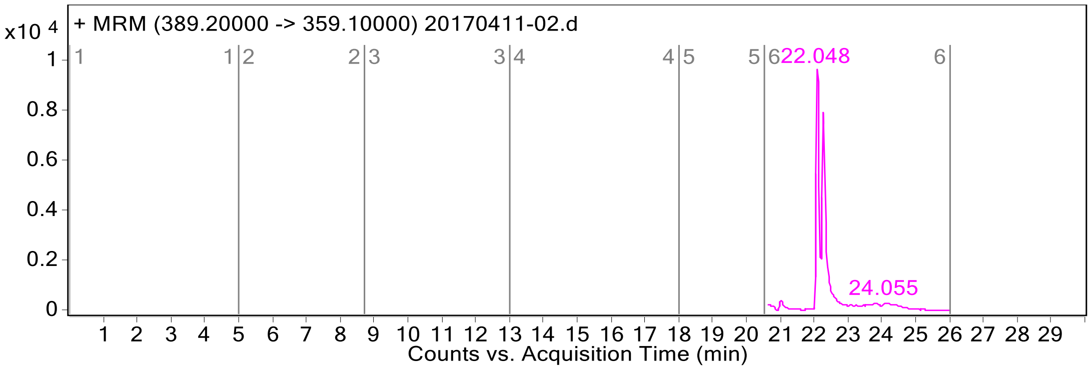

+ MRM (389.20000 -> 359.10000) 20170411-02.d
x10 4
22.048
24.055
Counts vs. Acquisition Time (min)

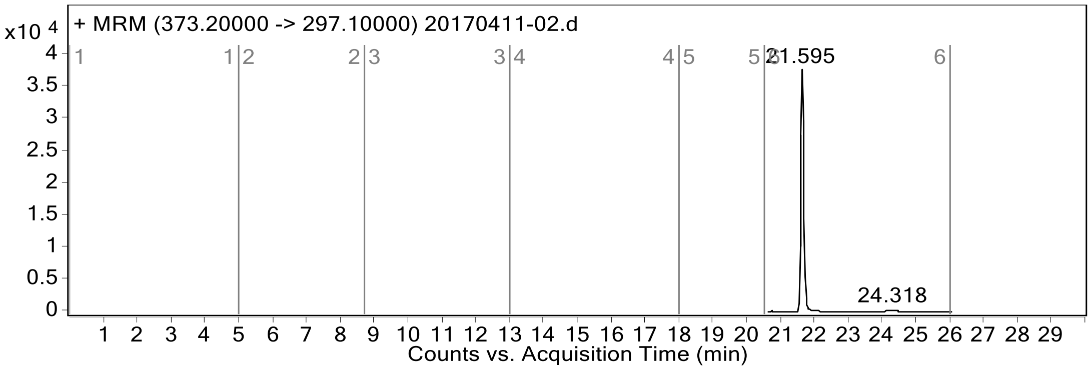
x10 4
+ MRM (373.20000 -> 297.10000) 20170411-02.d
21.595
24.318
Counts vs. Acquisition Time (min)

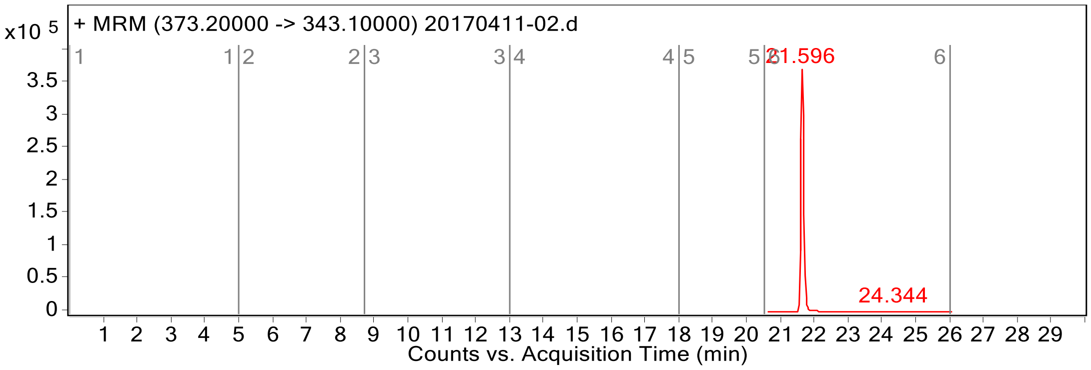
x10 5
+ MRM (373.20000 -> 343.10000) 20170411-02.d
21.596
24.344
Counts vs. Acquisition Time (min)

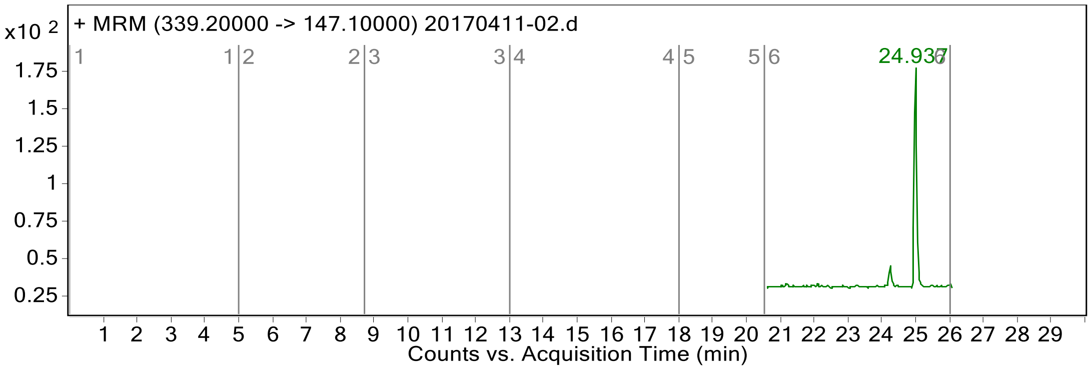
x10 2
+ MRM (339.20000 -> 147.10000) 20170411-02.d
24.937
Counts vs. Acquisition Time (min)

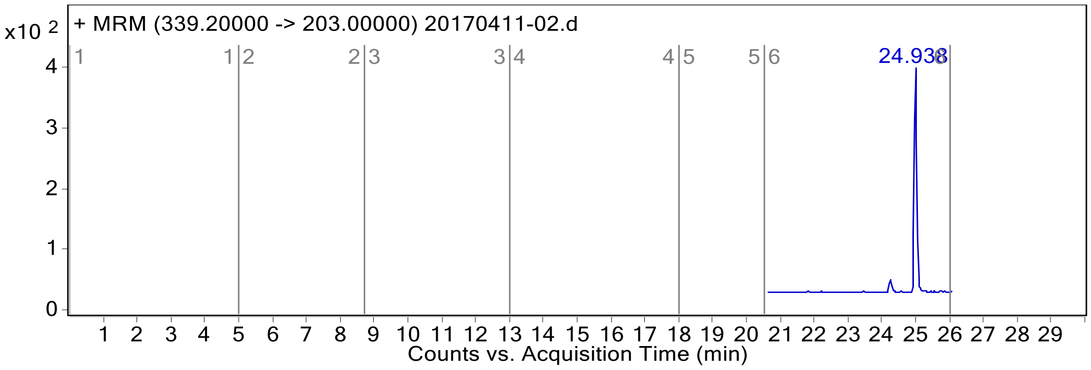
x10 2
+ MRM (339.20000 -> 203.00000) 20170411-02.d
24.938
Counts vs. Acquisition Time (min)

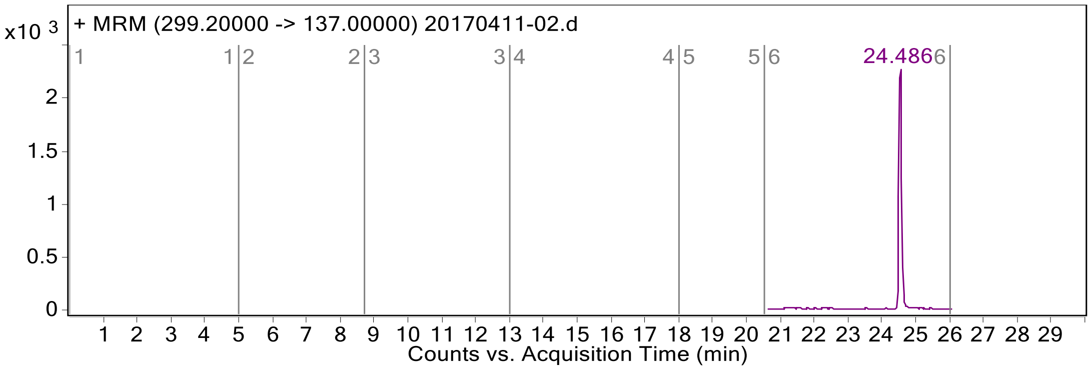
x10 3
+ MRM (299.20000 -> 137.00000) 20170411-02.d
24.4866
Counts vs. Acquisition Time (min)

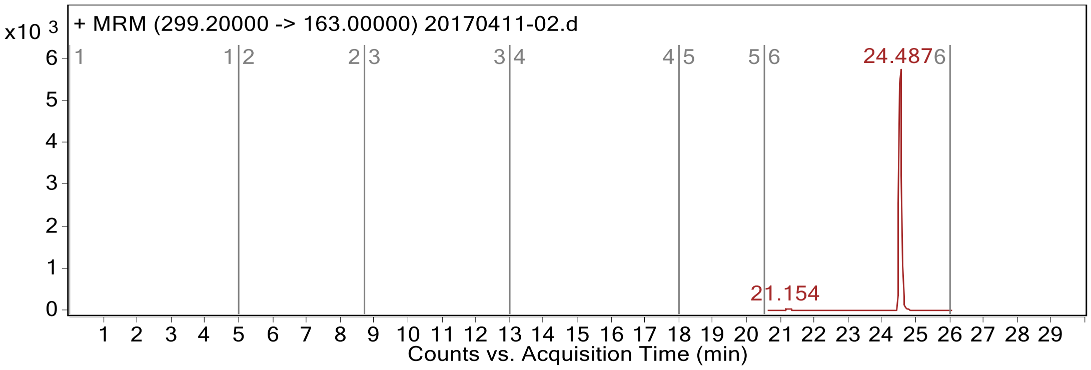
x10 3
+ MRM (299.20000 -> 163.00000) 20170411-02.d
24.4876
21.154
Counts vs. Acquisition Time (min)

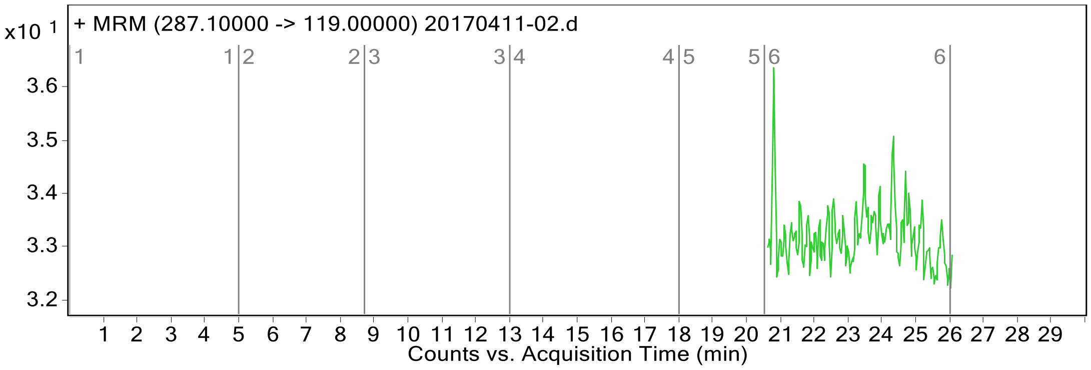
x10 1
+ MRM (287.10000 -> 119.00000) 20170411-02.d
Counts vs. Acquisition Time (min)

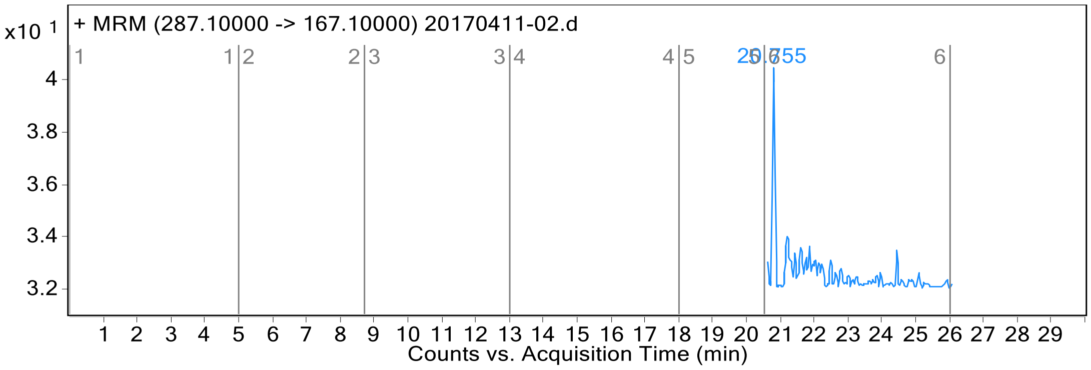
x10 1
+ MRM (287.10000 -> 167.10000) 20170411-02.d
Counts vs. Acquisition Time (min)

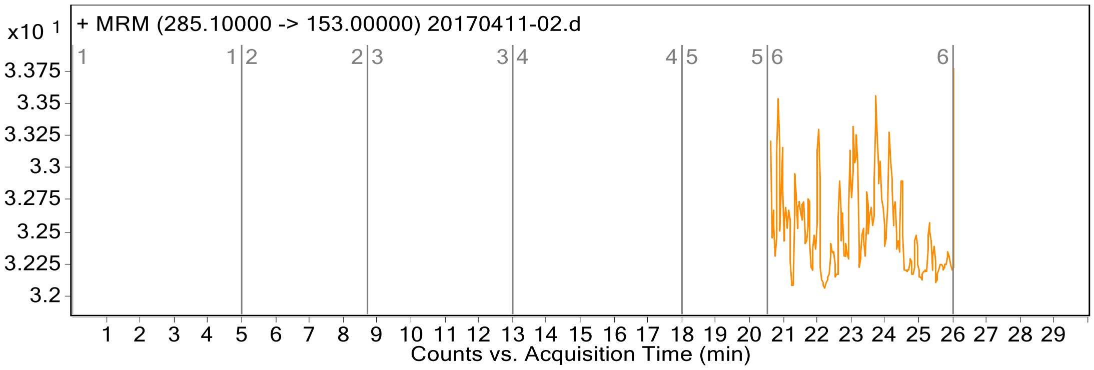
x10 1
+ MRM (285.10000 -> 153.00000) 20170411-02.d
Counts vs. Acquisition Time (min)

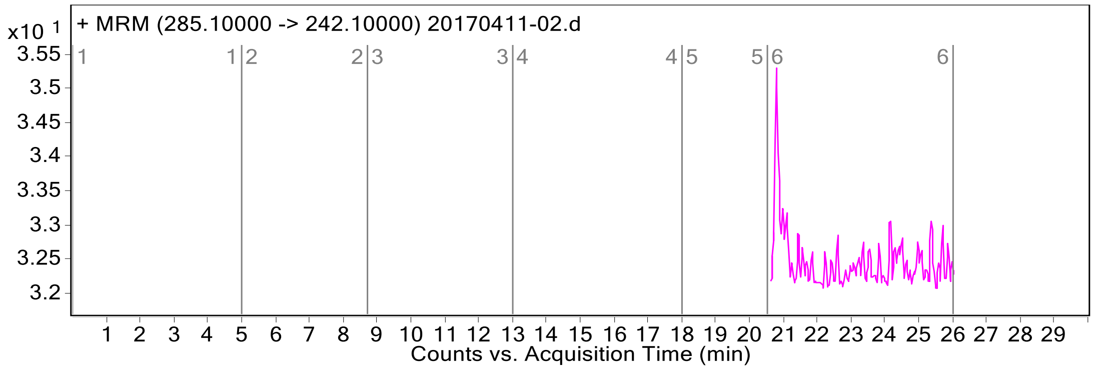
x10 1
+ MRM (285.10000 -> 242.10000) 20170411-02.d
Counts vs. Acquisition Time (min)

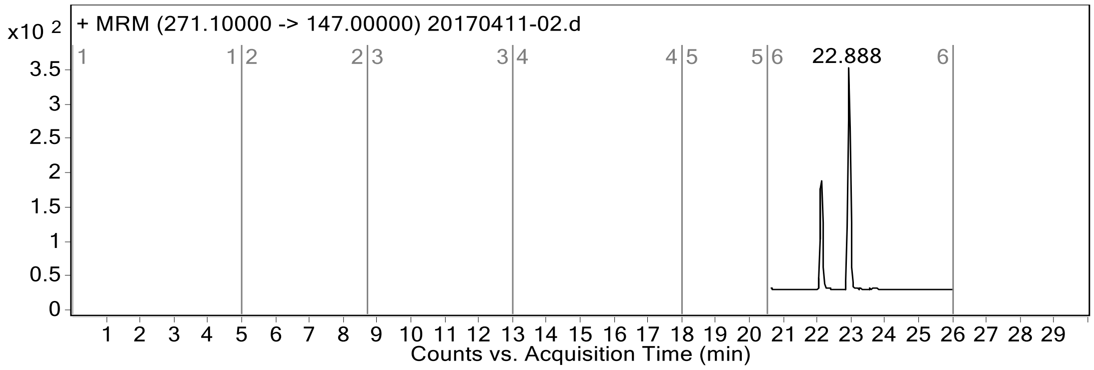
x10 2
+ MRM (271.10000 -> 147.00000) 20170411-02.d
22.888
Counts vs. Acquisition Time (min)

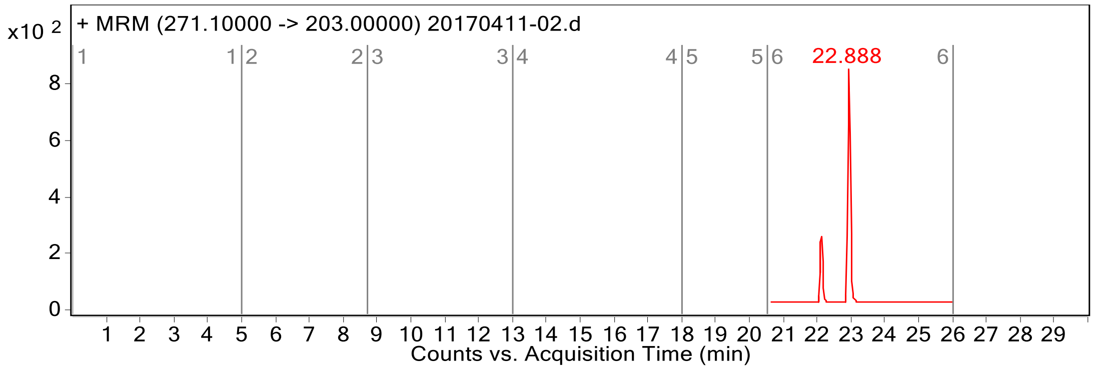
x10 2
+ MRM (271.10000 -> 203.00000) 20170411-02.d
22.888
Counts vs. Acquisition Time (min)

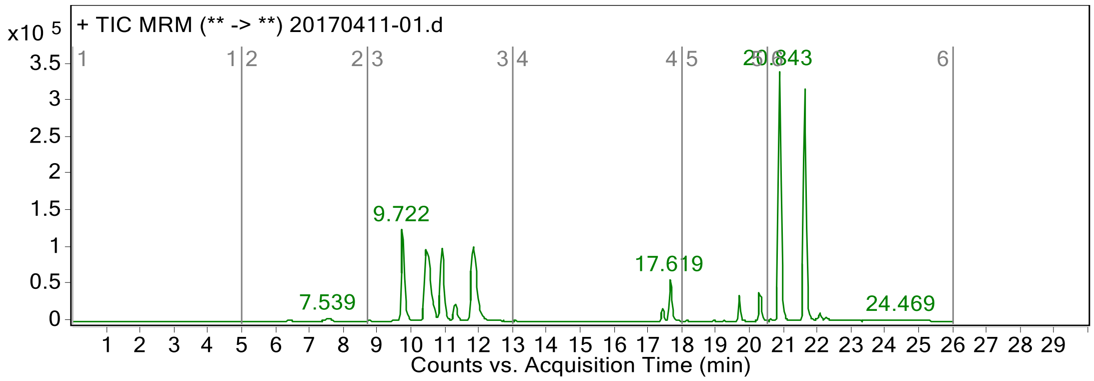

x10 5
+ TIC MRM (** -> **) 20170411-01.d
9.722
17.619
7.539
24.469
Counts vs. Acquisition Time (min)

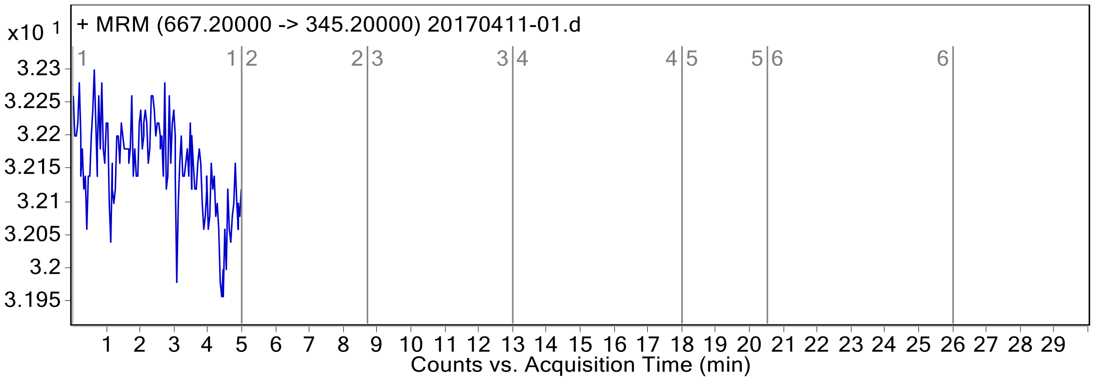

x10 1
+ MRM (667.20000 -> 345.20000) 20170411-01.d
Counts vs. Acquisition Time (min)

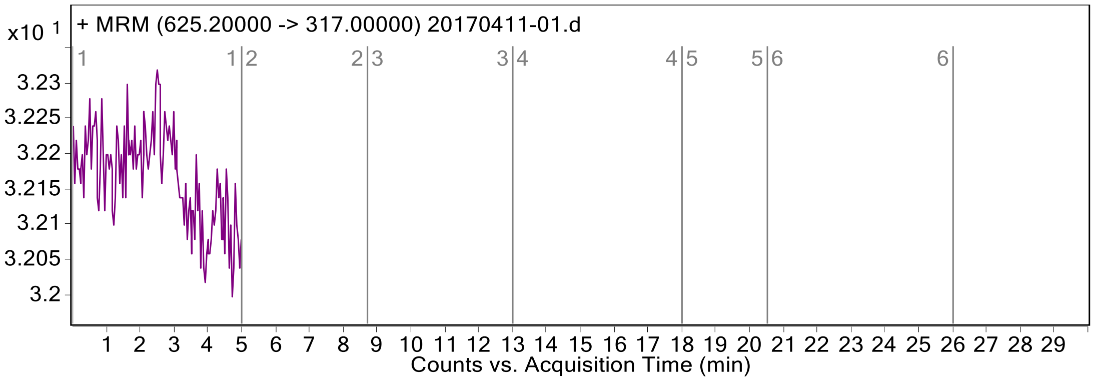

x10 1
+ MRM (625.20000 -> 317.00000) 20170411-01.d
Counts vs. Acquisition Time (min)

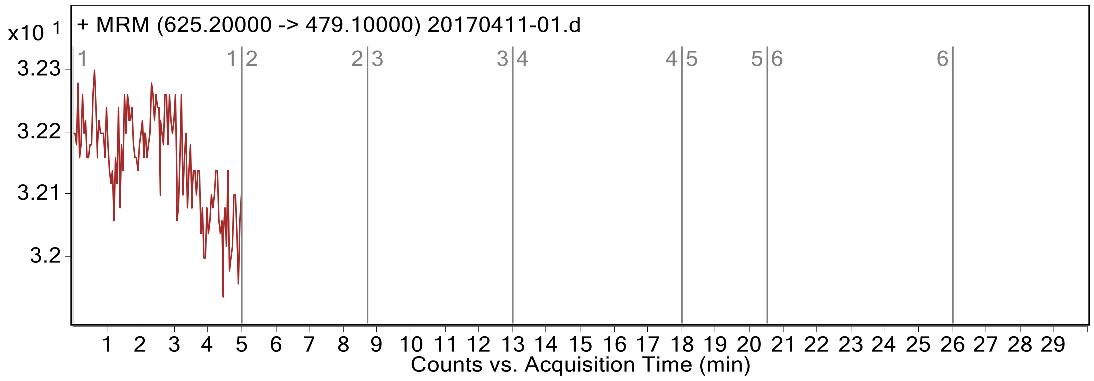

x10 1
+ MRM (625.20000 -> 479.10000) 20170411-01.d
Counts vs. Acquisition Time (min)

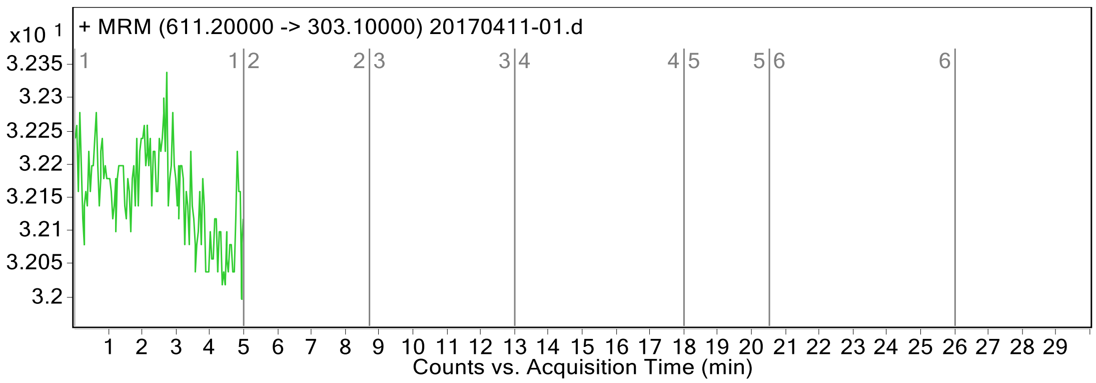

x10 1
+ MRM (611.20000 -> 303.10000) 20170411-01.d
Counts vs. Acquisition Time (min)

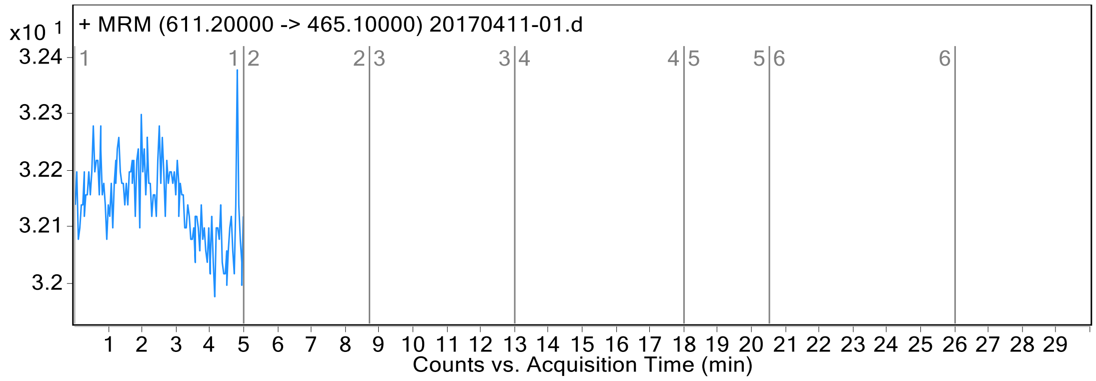

x10 1
+ MRM (611.20000 -> 465.10000) 20170411-01.d
Counts vs. Acquisition Time (min)

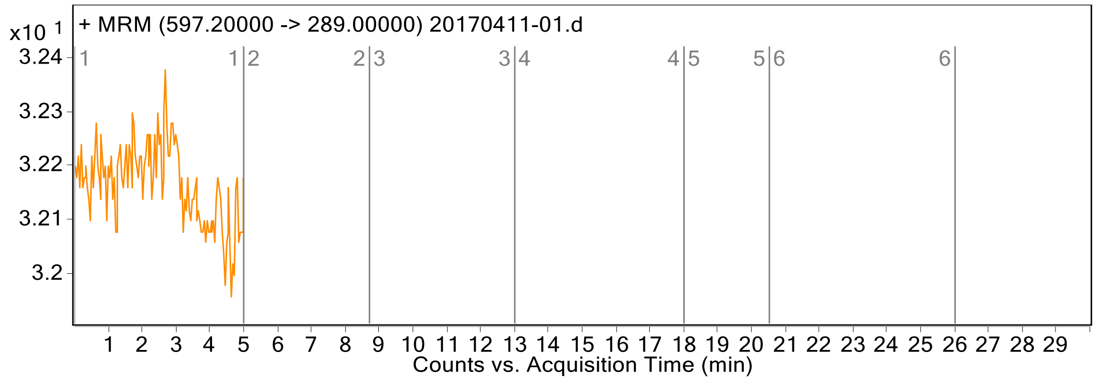

x10 1
+ MRM (597.20000 -> 289.00000) 20170411-01.d
Counts vs. Acquisition Time (min)

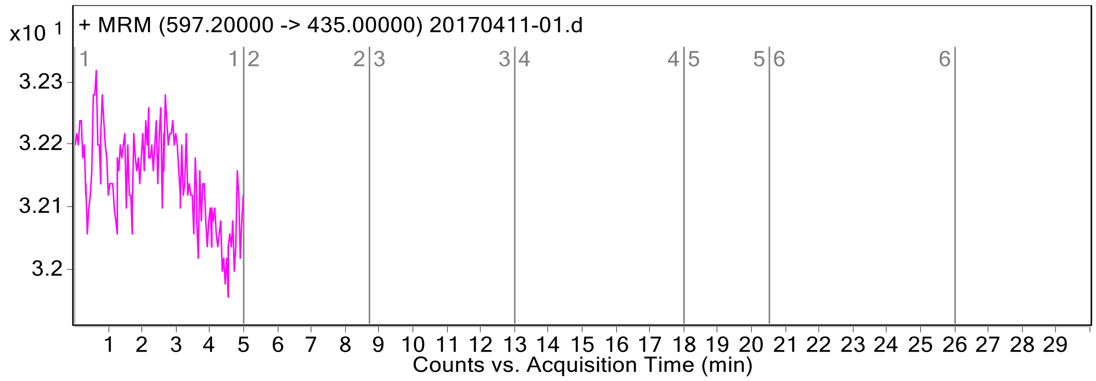

x10 1
+ MRM (597.20000 -> 435.00000) 20170411-01.d
Counts vs. Acquisition Time (min)

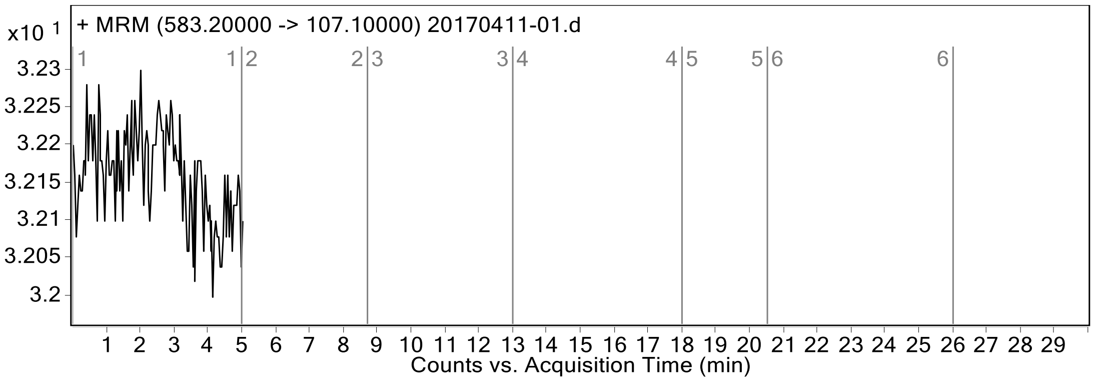

x10 1
+ MRM (583.20000 -> 107.10000) 20170411-01.d
Counts vs. Acquisition Time (min)

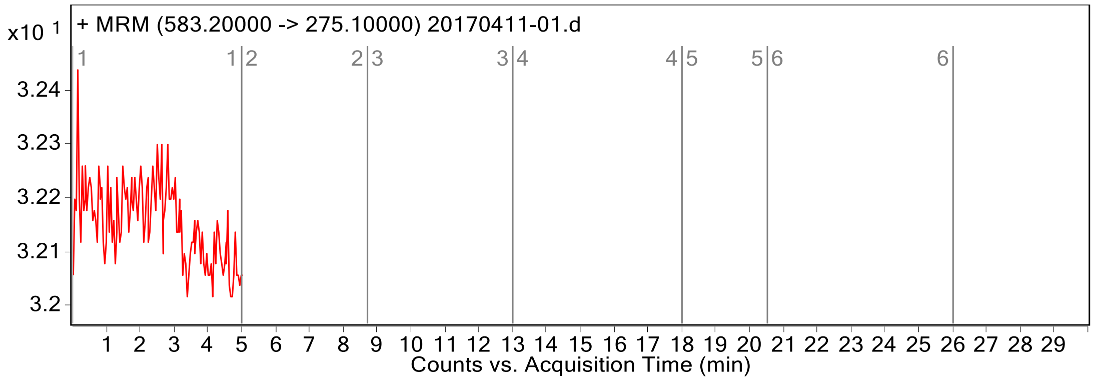

x10 1
+ MRM (583.20000 -> 275.10000) 20170411-01.d
Counts vs. Acquisition Time (min)

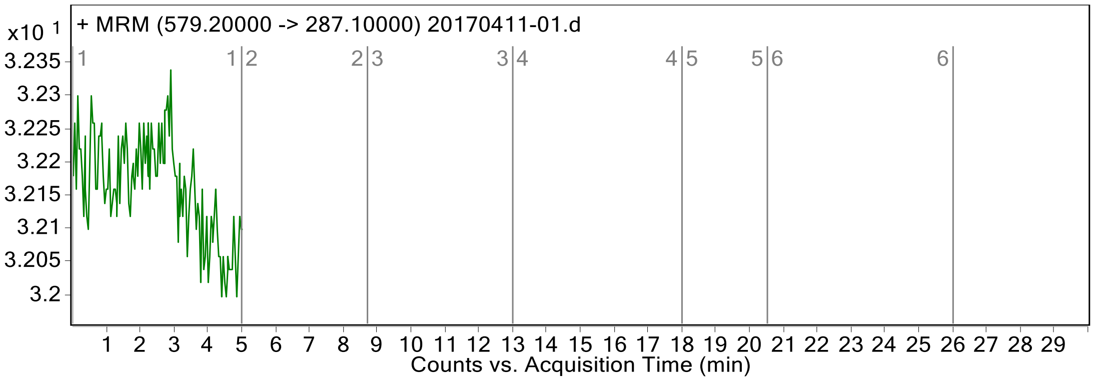

x10 1
+ MRM (579.20000 -> 287.10000) 20170411-01.d
Counts vs. Acquisition Time (min)

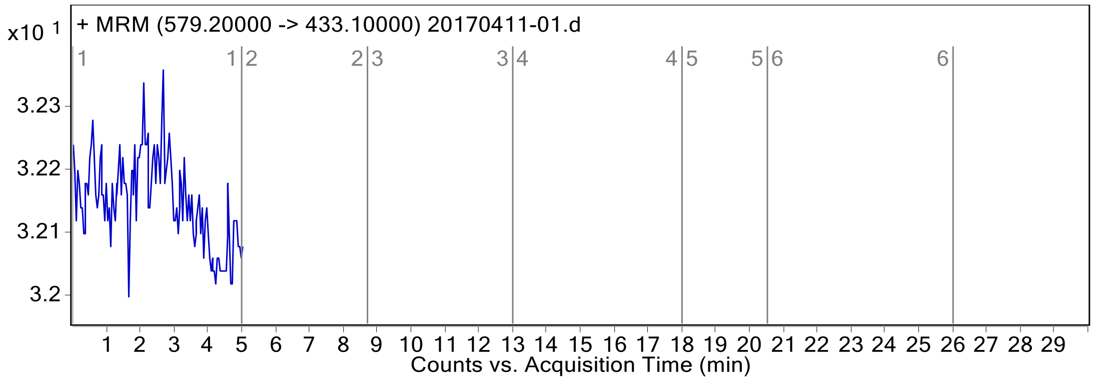

x10 1
+ MRM (579.20000 -> 433.10000) 20170411-01.d
Counts vs. Acquisition Time (min)

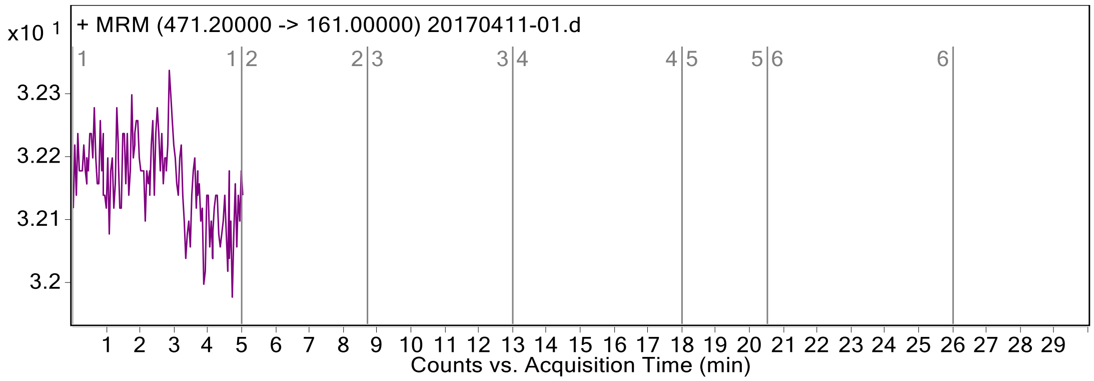
+ MRM (471.20000 -> 161.00000) 20170411-01.d
x10 1
3.23
3.22
3.21
3.2
1 2 3 4 5 6 7 8 9 10 11 12 13 14 15 16 17 18 19 20 21 22 23 24 25 26 27 28 29
Counts vs. Acquisition Time (min)

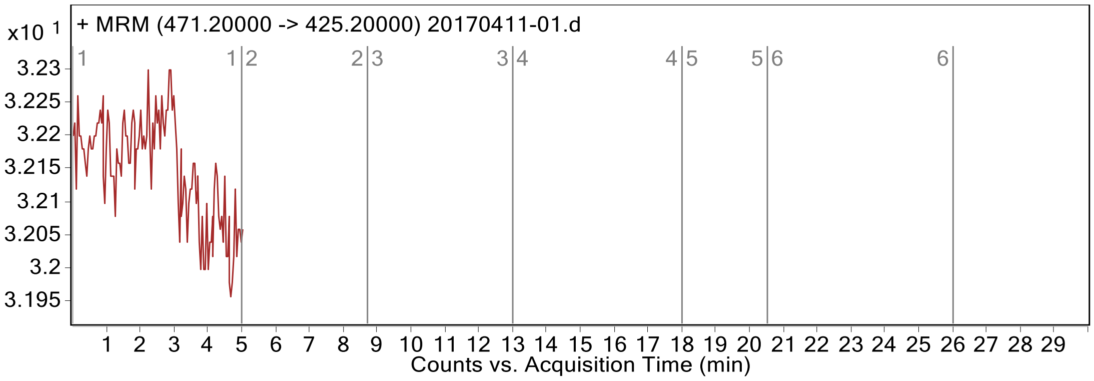
+ MRM (471.20000 -> 425.20000) 20170411-01.d
x10 1
3.23
3.225
3.22
3.215
3.21
3.205
3.2
3.195
1 2 3 4 5 6 7 8 9 10 11 12 13 14 15 16 17 18 19 20 21 22 23 24 25 26 27 28 29
Counts vs. Acquisition Time (min)

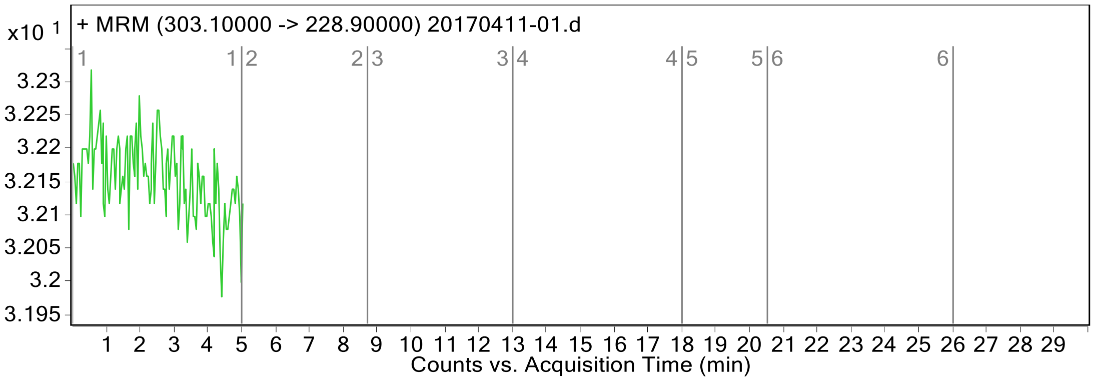
+ MRM (303.10000 -> 228.90000) 20170411-01.d
x10 1
3.23
3.225
3.22
3.215
3.21
3.205
3.2
3.195
1 2 3 4 5 6 7 8 9 10 11 12 13 14 15 16 17 18 19 20 21 22 23 24 25 26 27 28 29
Counts vs. Acquisition Time (min)

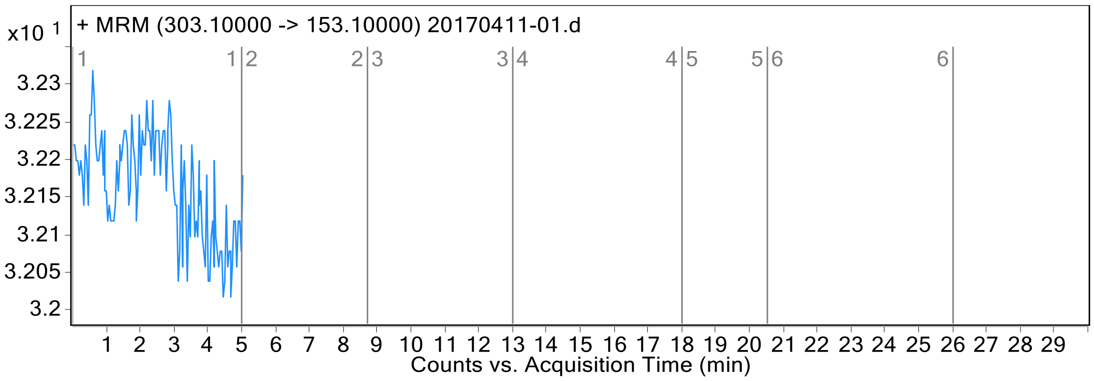
+ MRM (303.10000 -> 153.10000) 20170411-01.d
x10 1
3.23
3.225
3.22
3.215
3.21
3.205
3.2
1 2 3 4 5 6 7 8 9 10 11 12 13 14 15 16 17 18 19 20 21 22 23 24 25 26 27 28 29
Counts vs. Acquisition Time (min)

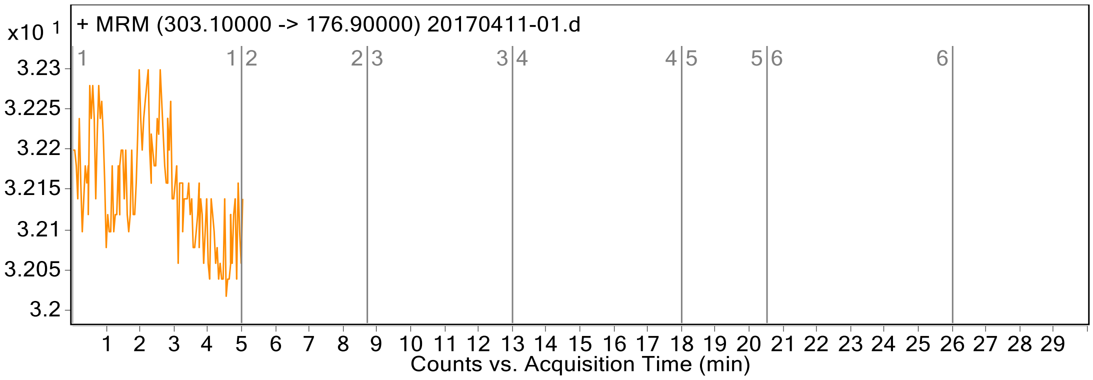
+ MRM (303.10000 -> 176.90000) 20170411-01.d
x10 1
3.23
3.225
3.22
3.215
3.21
3.205
3.2
1 2 3 4 5 6 7 8 9 10 11 12 13 14 15 16 17 18 19 20 21 22 23 24 25 26 27 28 29
Counts vs. Acquisition Time (min)

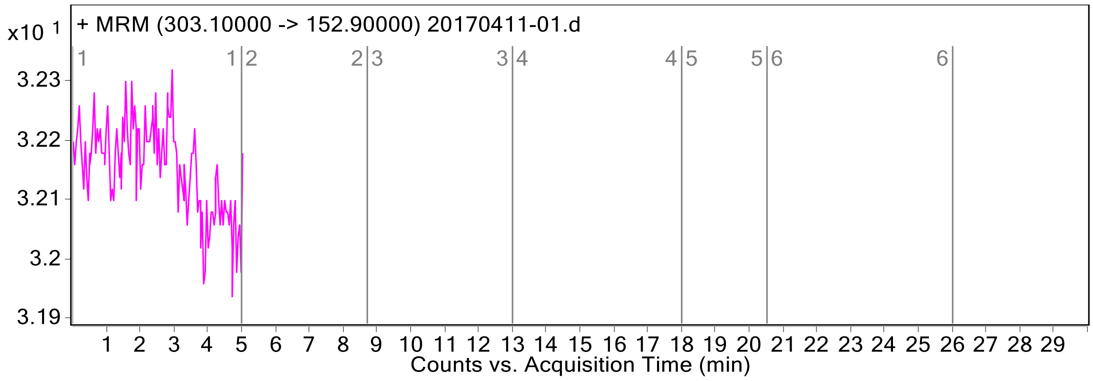
+ MRM (303.10000 -> 152.90000) 20170411-01.d
x10 1
3.23
3.22
3.21
3.2
3.19
1 2 3 4 5 6 7 8 9 10 11 12 13 14 15 16 17 18 19 20 21 22 23 24 25 26 27 28 29
Counts vs. Acquisition Time (min)

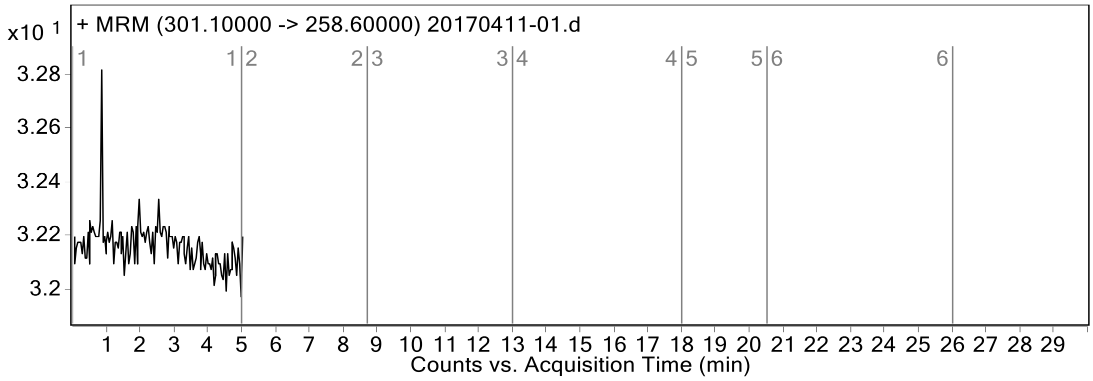
+ MRM (301.10000 -> 258.60000) 20170411-01.d
x10 1
3.28
3.26
3.24
3.22
3.2
1 2 3 4 5 6 7 8 9 10 11 12 13 14 15 16 17 18 19 20 21 22 23 24 25 26 27 28 29
Counts vs. Acquisition Time (min)

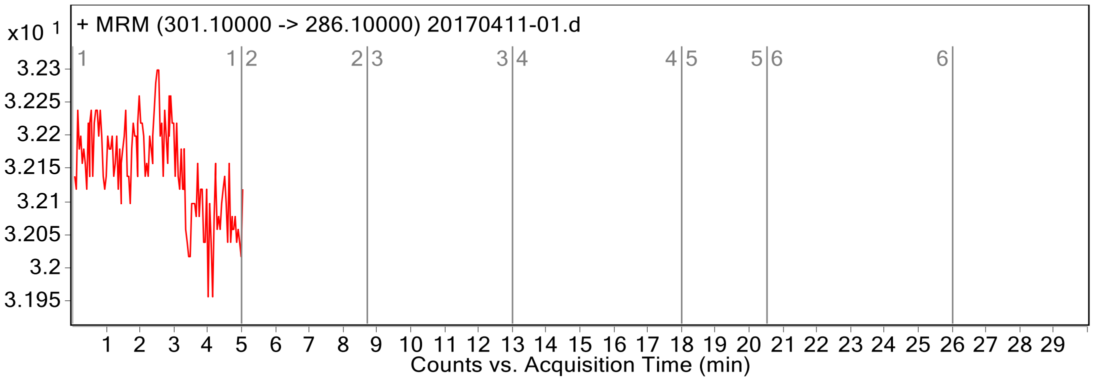
+ MRM (301.10000 -> 286.10000) 20170411-01.d
x10 1
3.23
3.225
3.22
3.215
3.21
3.205
3.2
3.195
1 2 3 4 5 6 7 8 9 10 11 12 13 14 15 16 17 18 19 20 21 22 23 24 25 26 27 28 29
Counts vs. Acquisition Time (min)

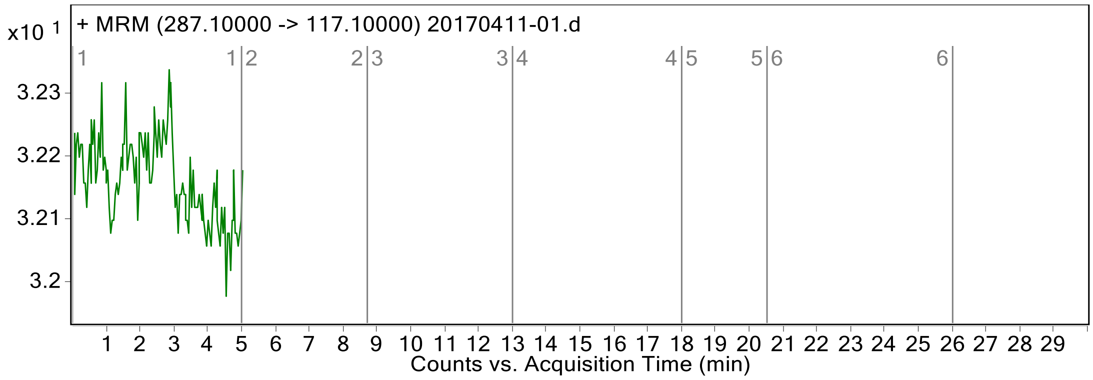
x10 1
+ MRM (287.10000 -> 117.10000) 20170411-01.d
3.23
3.22
3.21
3.2
1 2 3 4 5 6 7 8 9 10 11 12 13 14 15 16 17 18 19 20 21 22 23 24 25 26 27 28 29
Counts vs. Acquisition Time (min)

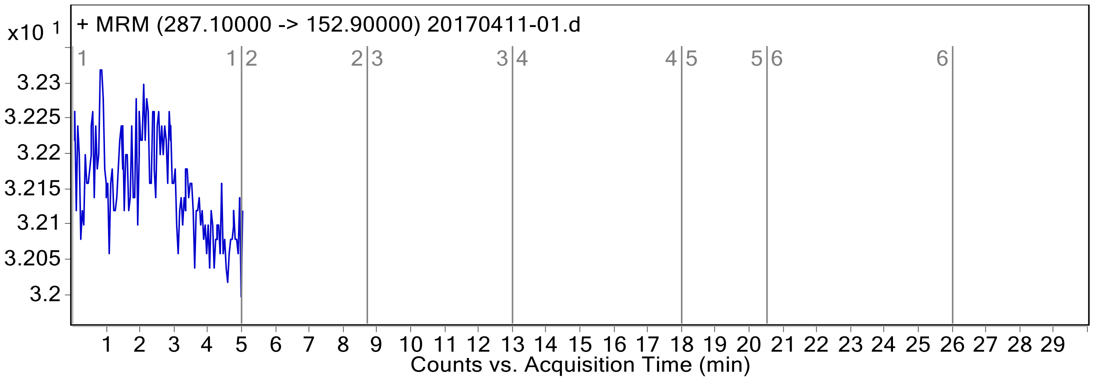
x10 1
+ MRM (287.10000 -> 152.90000) 20170411-01.d
3.23
3.225
3.22
3.215
3.21
3.205
3.2
1 2 3 4 5 6 7 8 9 10 11 12 13 14 15 16 17 18 19 20 21 22 23 24 25 26 27 28 29
Counts vs. Acquisition Time (min)

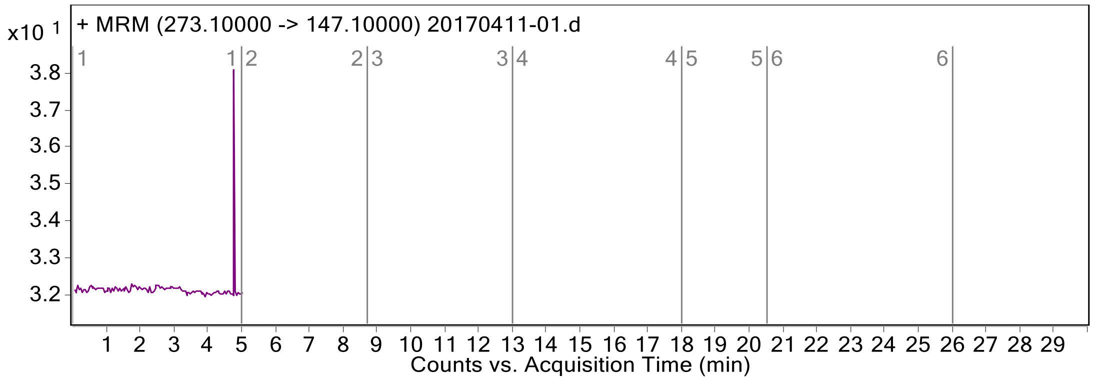
x10 1
+ MRM (273.10000 -> 147.10000) 20170411-01.d
3.8
3.7
3.6
3.5
3.4
3.3
3.2
1 2 3 4 5 6 7 8 9 10 11 12 13 14 15 16 17 18 19 20 21 22 23 24 25 26 27 28 29
Counts vs. Acquisition Time (min)

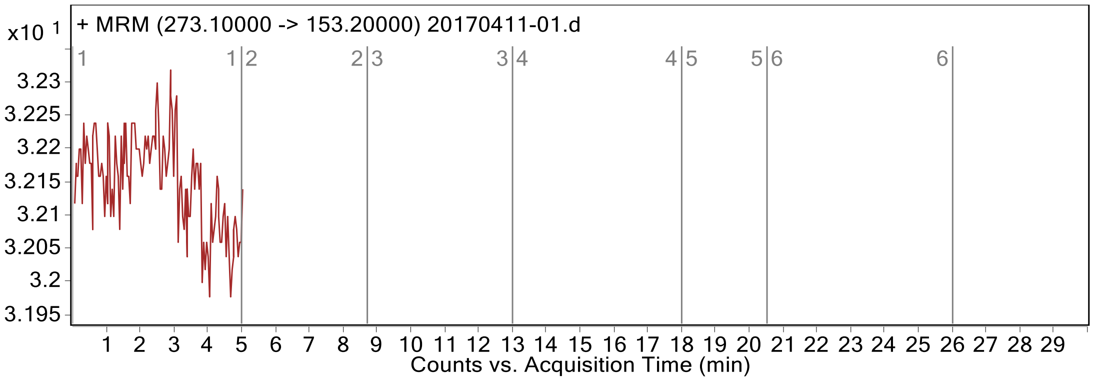
x10 1
+ MRM (273.10000 -> 153.20000) 20170411-01.d
3.23
3.225
3.22
3.215
3.21
3.205
3.2
3.195
1 2 3 4 5 6 7 8 9 10 11 12 13 14 15 16 17 18 19 20 21 22 23 24 25 26 27 28 29
Counts vs. Acquisition Time (min)

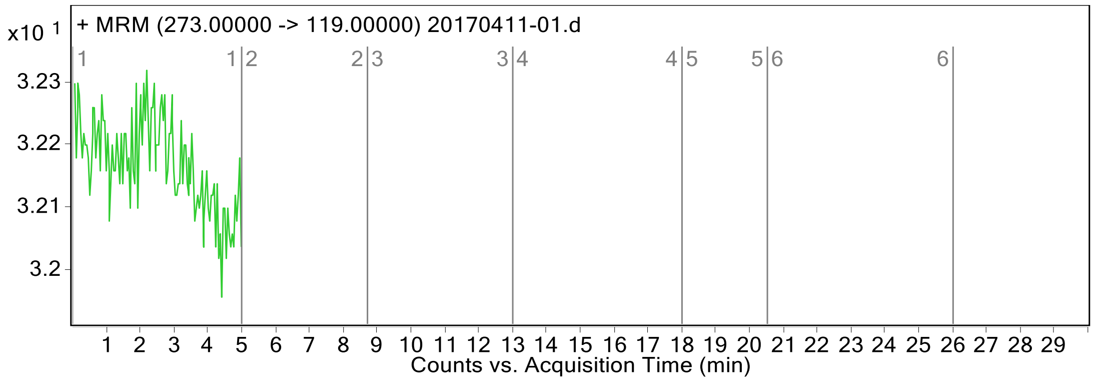

x10 1
+ MRM (273.00000 -> 119.00000) 20170411-01.d
3.23
3.22
3.21
3.2
1 2 3 4 5 6 7 8 9 10 11 12 13 14 15 16 17 18 19 20 21 22 23 24 25 26 27 28 29
Counts vs. Acquisition Time (min)

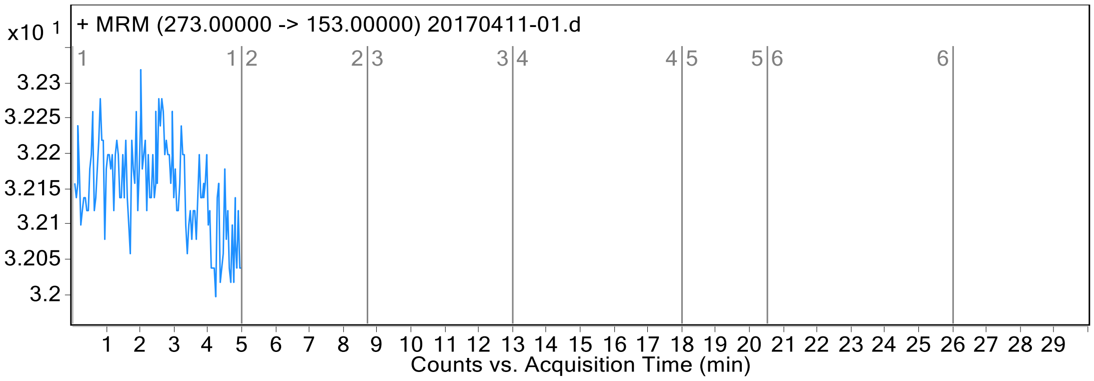

x10 1
+ MRM (273.00000 -> 153.00000) 20170411-01.d
3.23
3.225
3.22
3.215
3.21
3.205
3.2
1 2 3 4 5 6 7 8 9 10 11 12 13 14 15 16 17 18 19 20 21 22 23 24 25 26 27 28 29
Counts vs. Acquisition Time (min)

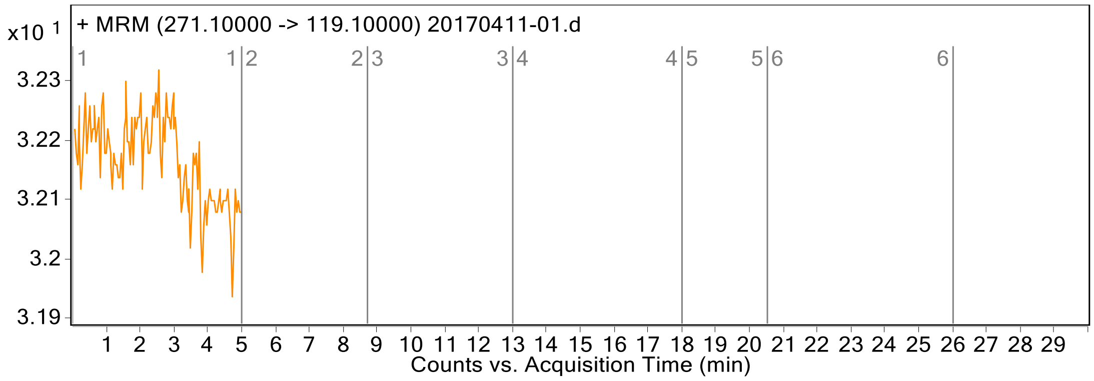

x10 1
+ MRM (271.10000 -> 119.10000) 20170411-01.d
3.23
3.22
3.21
3.2
3.19
1 2 3 4 5 6 7 8 9 10 11 12 13 14 15 16 17 18 19 20 21 22 23 24 25 26 27 28 29
Counts vs. Acquisition Time (min)

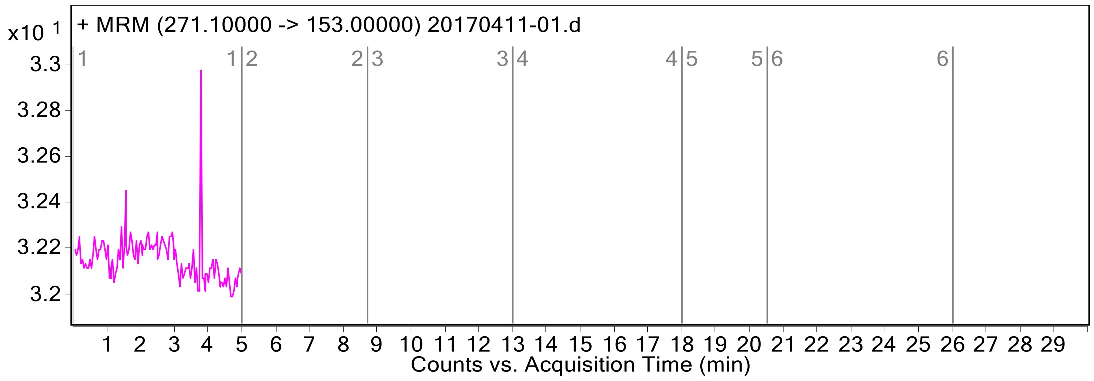

x10 1
+ MRM (271.10000 -> 153.00000) 20170411-01.d
3.3
3.28
3.26
3.24
3.22
3.2
1 2 3 4 5 6 7 8 9 10 11 12 13 14 15 16 17 18 19 20 21 22 23 24 25 26 27 28 29
Counts vs. Acquisition Time (min)

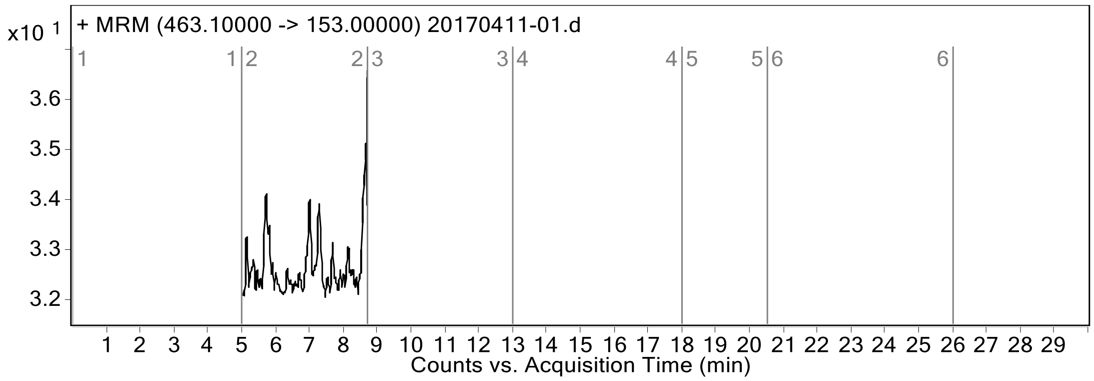
x10 1
+ MRM (463.10000 -> 153.00000) 20170411-01.d
Counts vs. Acquisition Time (min)

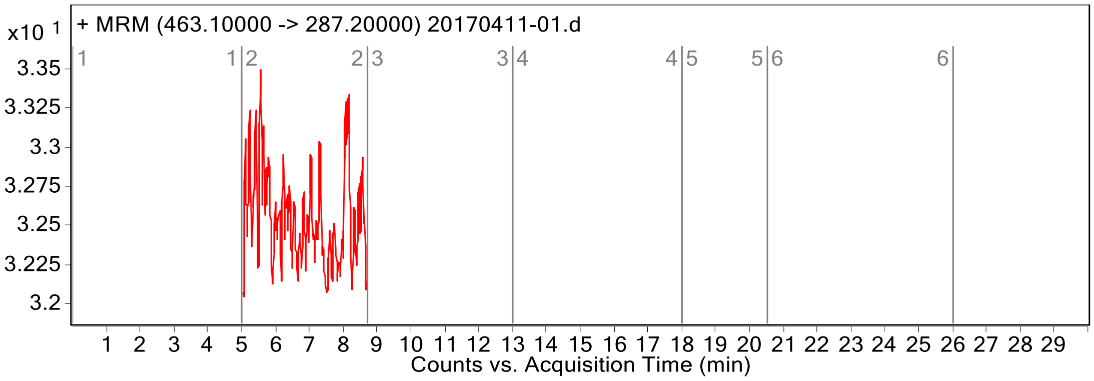
x10 1
+ MRM (463.10000 -> 287.20000) 20170411-01.d
Counts vs. Acquisition Time (min)

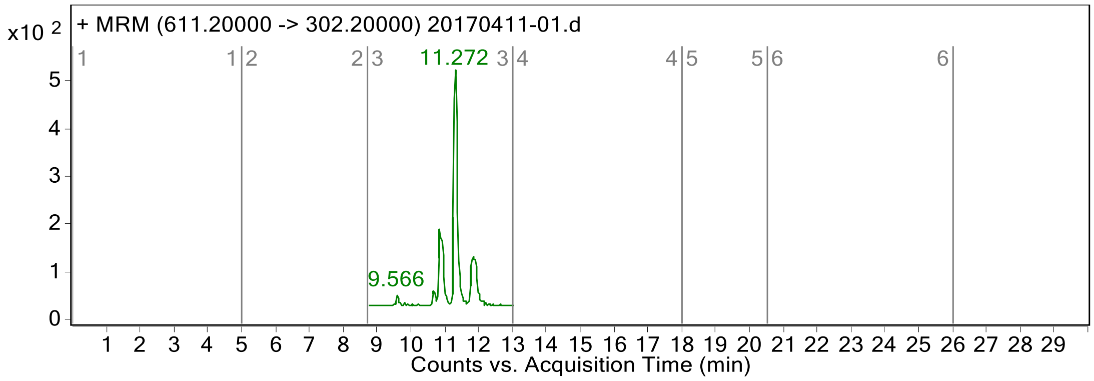
x10 2
+ MRM (611.20000 -> 302.20000) 20170411-01.d
11.272
9.566
Counts vs. Acquisition Time (min)

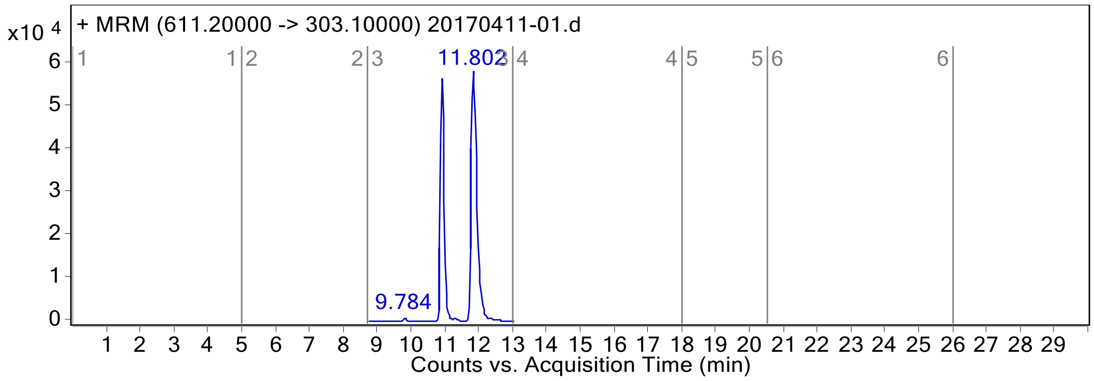
x10 4
+ MRM (611.20000 -> 303.10000) 20170411-01.d
11.802
9.784
Counts vs. Acquisition Time (min)

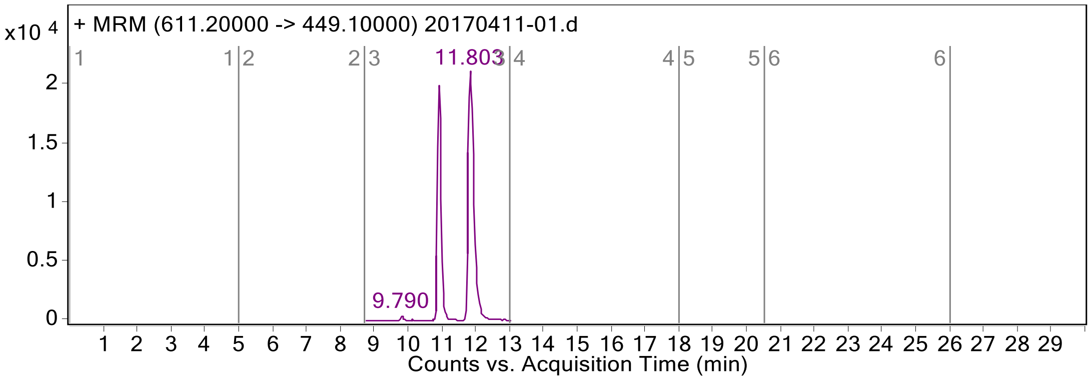
x10 4
+ MRM (611.20000 -> 449.10000) 20170411-01.d
11.803
9.790
Counts vs. Acquisition Time (min)

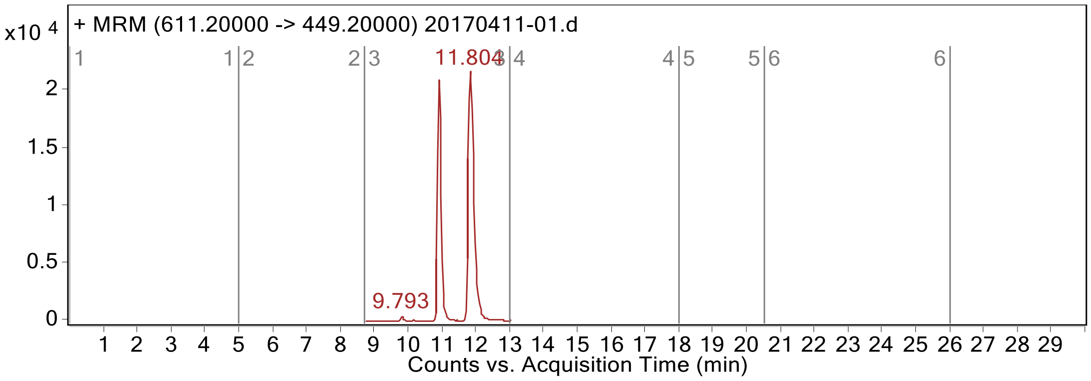
x10 4
+ MRM (611.20000 -> 449.20000) 20170411-01.d
11.804
9.793
Counts vs. Acquisition Time (min)

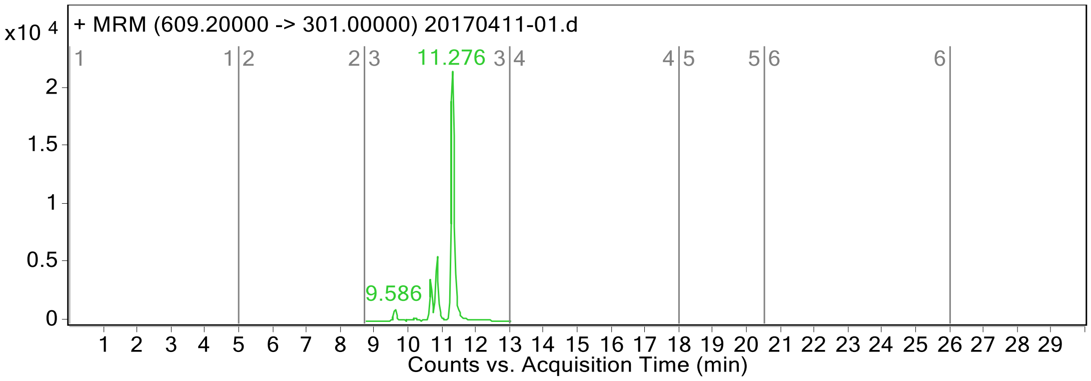
x10 4
+ MRM (609.20000 -> 301.00000) 20170411-01.d
11.276
9.586
Counts vs. Acquisition Time (min)

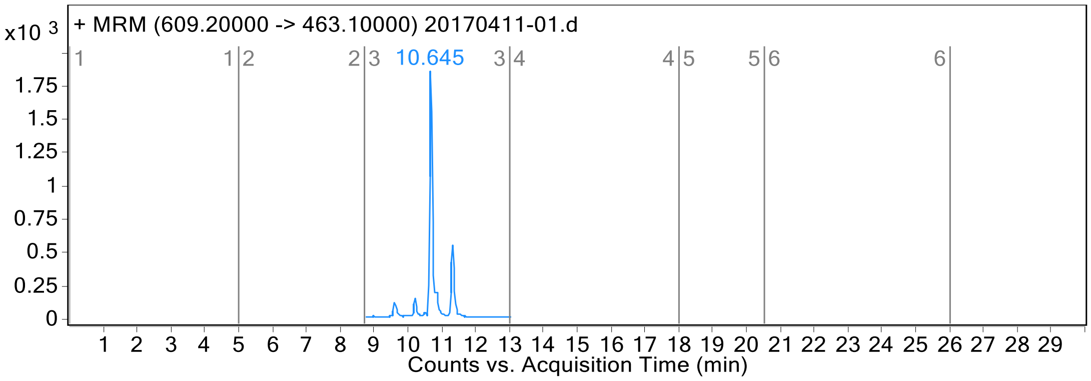
x10 3
+ MRM (609.20000 -> 463.10000) 20170411-01.d
10.645
Counts vs. Acquisition Time (min)

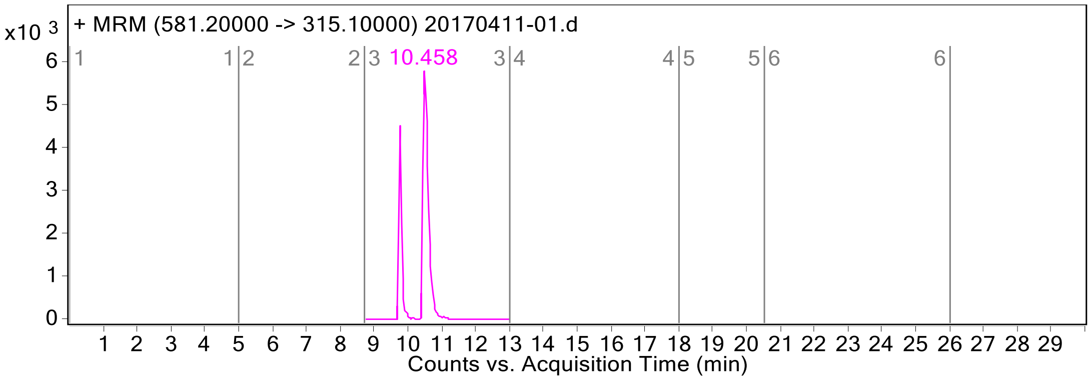
x10 3
+ MRM (581.20000 -> 315.10000) 20170411-01.d
10.458
Counts vs. Acquisition Time (min)

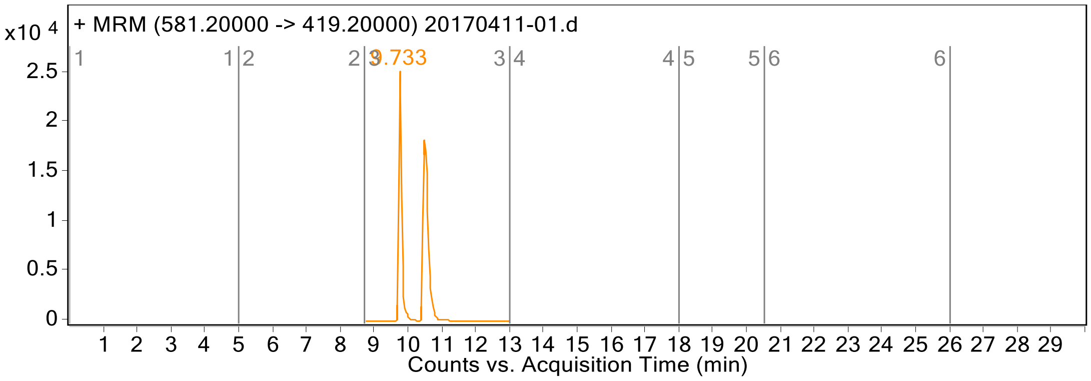
x10 4
+ MRM (581.20000 -> 419.20000) 20170411-01.d
9.733
Counts vs. Acquisition Time (min)

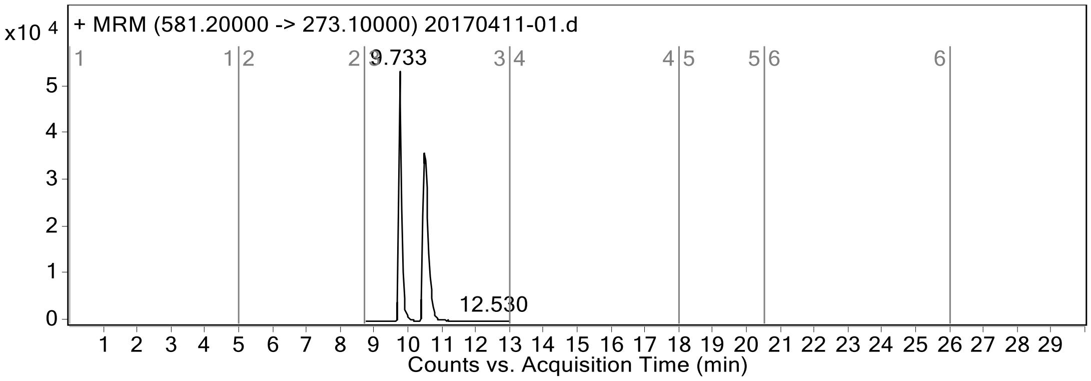
x10 4
+ MRM (581.20000 -> 273.10000) 20170411-01.d
9.733
12.530
Counts vs. Acquisition Time (min)

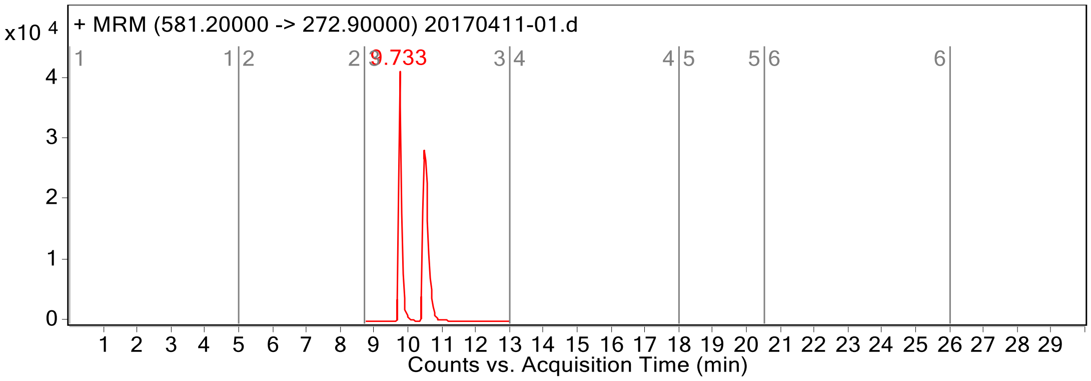
x10 4
+ MRM (581.20000 -> 272.90000) 20170411-01.d
9.733
Counts vs. Acquisition Time (min)

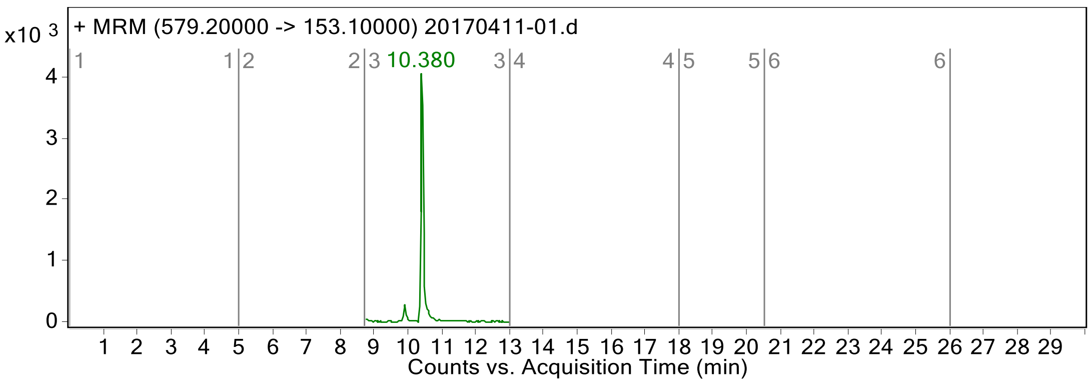
+ MRM (579.20000 -> 153.10000) 20170411-01.d
x10 3
10.380
Counts vs. Acquisition Time (min)

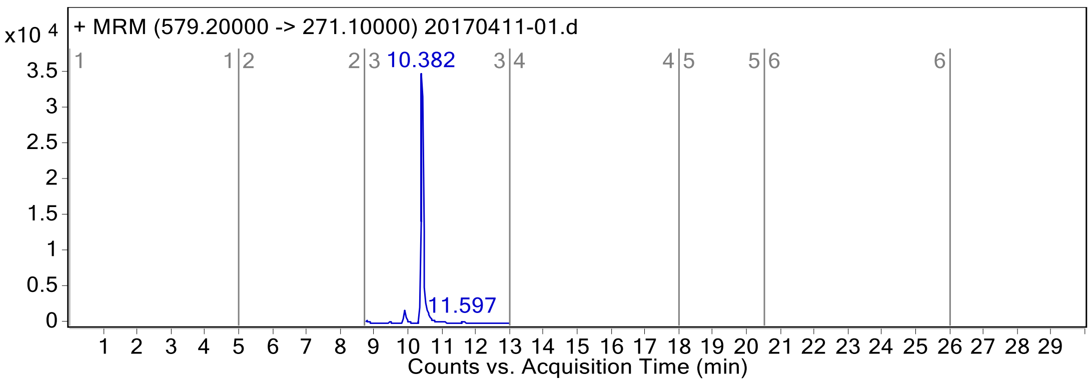
+ MRM (579.20000 -> 271.10000) 20170411-01.d
x10 4
10.382
11.597
Counts vs. Acquisition Time (min)

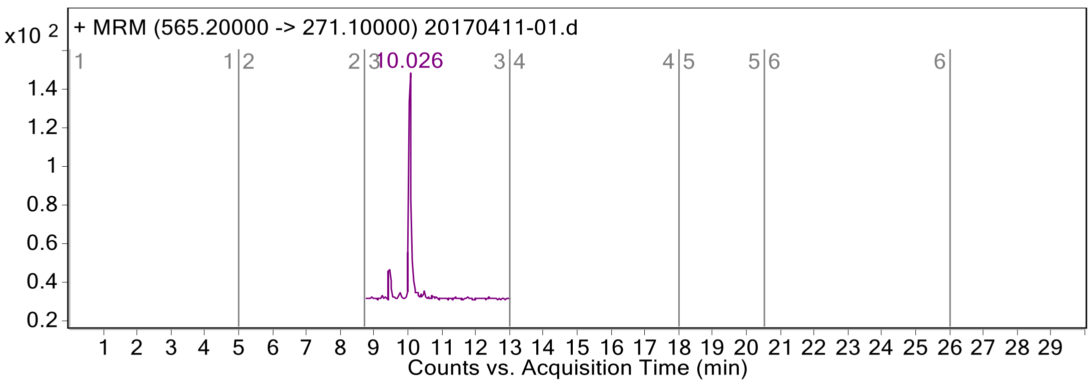
+ MRM (565.20000 -> 271.10000) 20170411-01.d
x10 2
10.026
Counts vs. Acquisition Time (min)

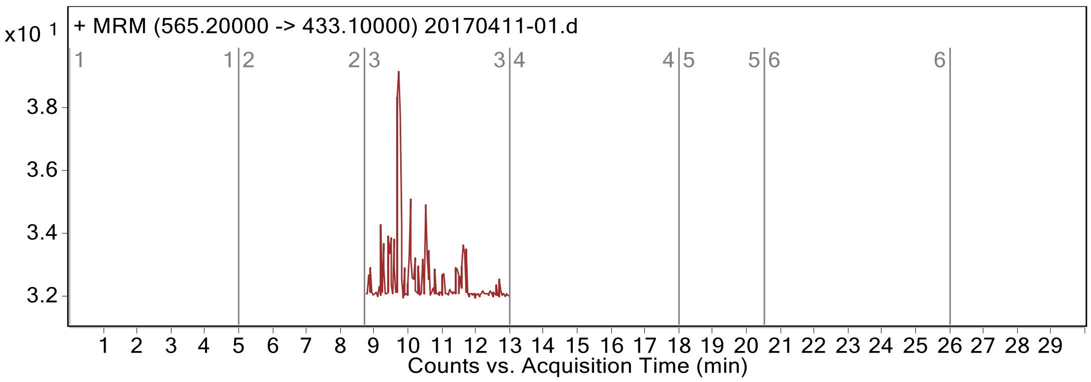
+ MRM (565.20000 -> 433.10000) 20170411-01.d
x10 1
Counts vs. Acquisition Time (min)

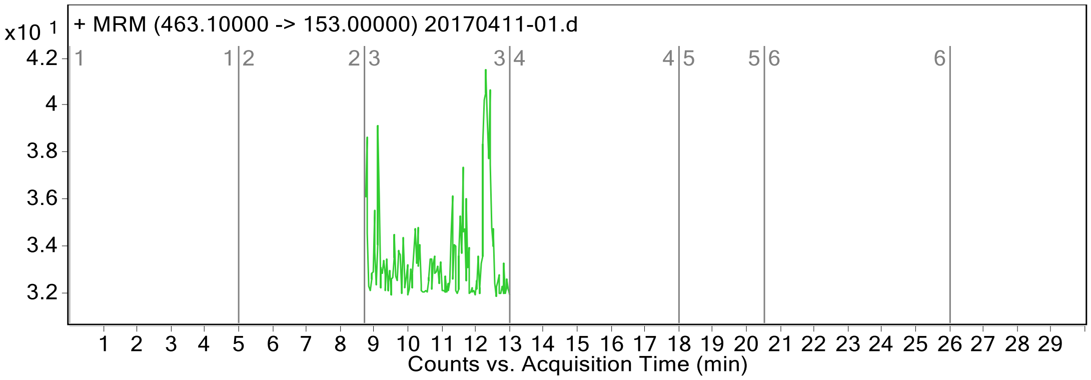
+ MRM (463.10000 -> 153.00000) 20170411-01.d
x10 1
Counts vs. Acquisition Time (min)

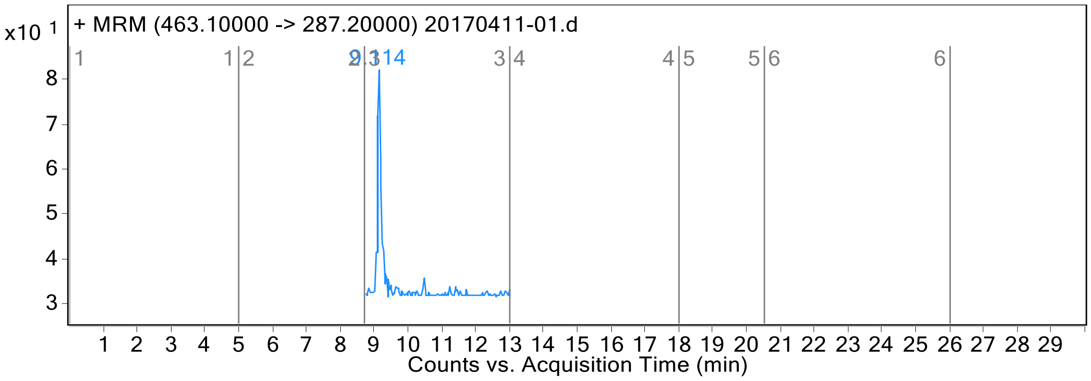
+ MRM (463.10000 -> 287.20000) 20170411-01.d
x10 1
9.314
Counts vs. Acquisition Time (min)

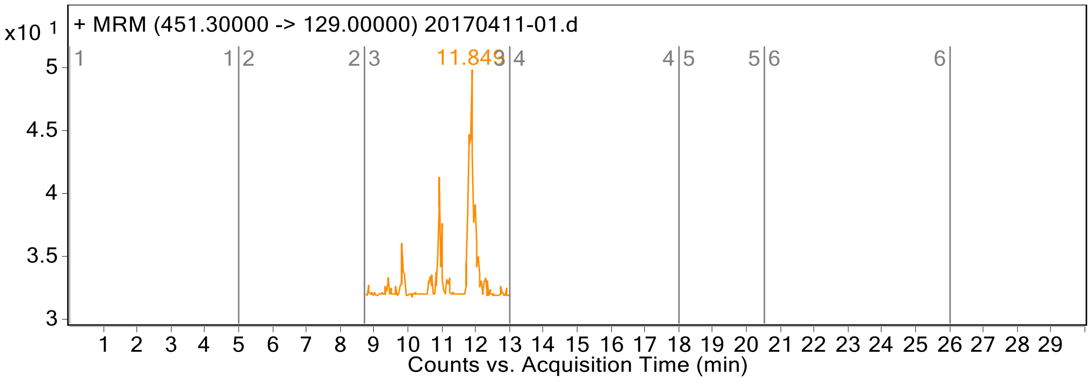
+ MRM (451.30000 -> 129.00000) 20170411-01.d
x10 1
11.849
Counts vs. Acquisition Time (min)

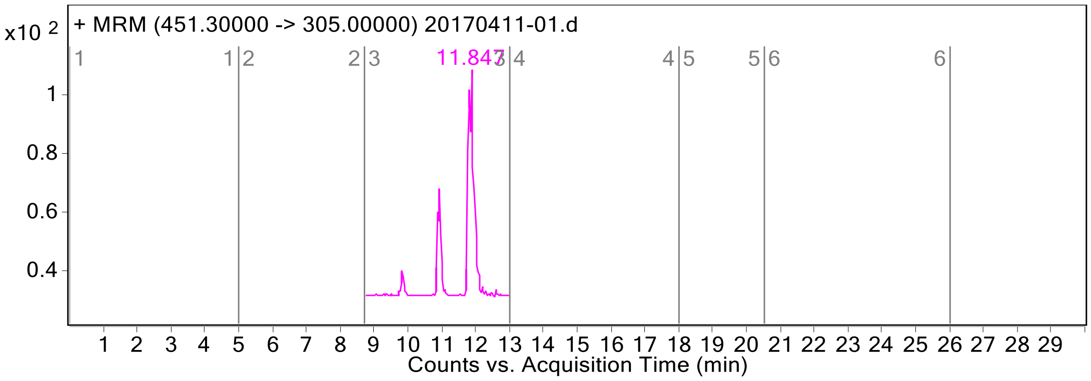
+ MRM (451.30000 -> 305.00000) 20170411-01.d
x10 2
11.847
Counts vs. Acquisition Time (min)

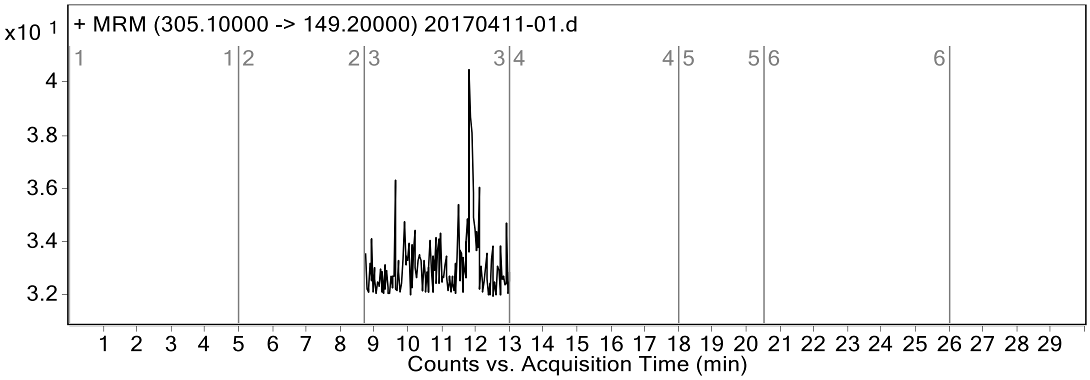
x10 1
+ MRM (305.10000 -> 149.20000) 20170411-01.d
Counts vs. Acquisition Time (min)

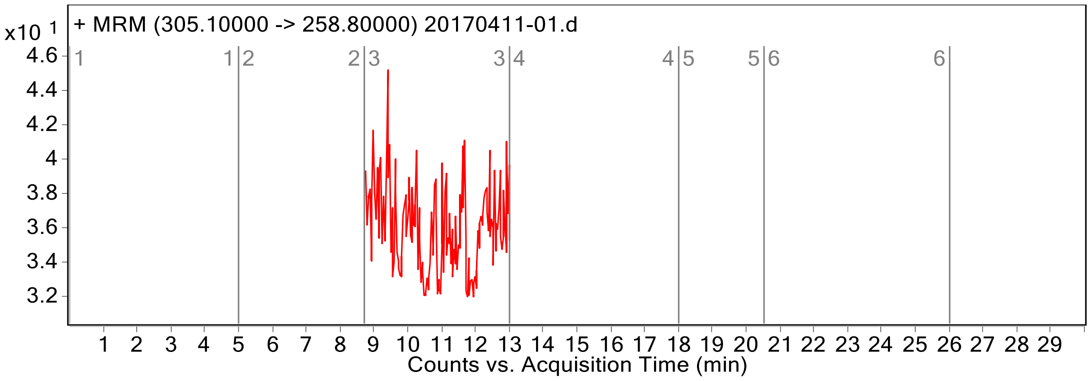
x10 1
+ MRM (305.10000 -> 258.80000) 20170411-01.d
Counts vs. Acquisition Time (min)

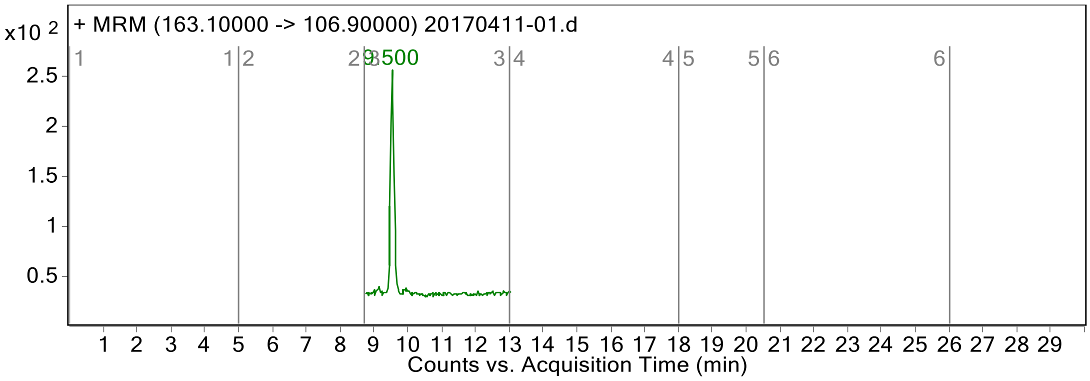
x10 2
+ MRM (163.10000 -> 106.90000) 20170411-01.d
9.500
Counts vs. Acquisition Time (min)

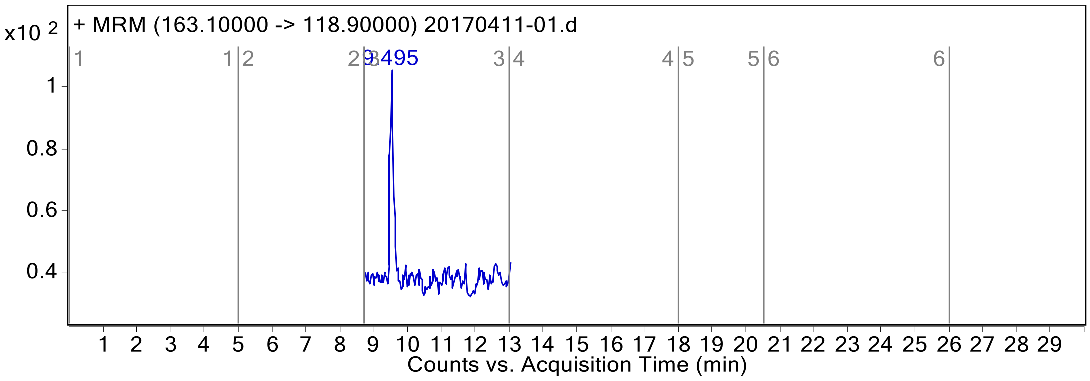
x10 2
+ MRM (163.10000 -> 118.90000) 20170411-01.d
9.495
Counts vs. Acquisition Time (min)

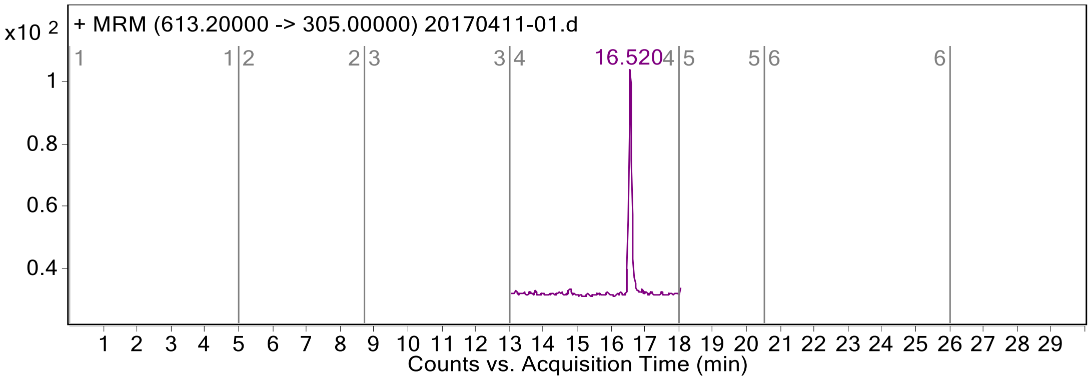

x10 2
+ MRM (613.20000 -> 305.00000) 20170411-01.d
16.520
Counts vs. Acquisition Time (min)

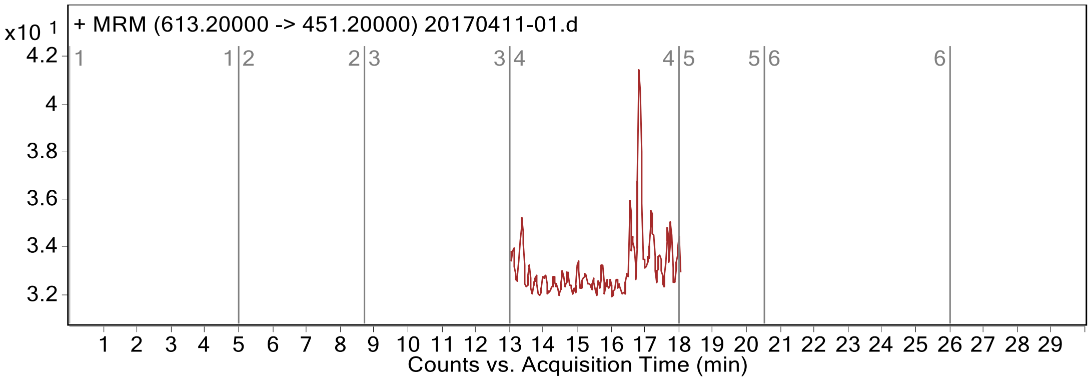

x10 1
+ MRM (613.20000 -> 451.20000) 20170411-01.d
Counts vs. Acquisition Time (min)

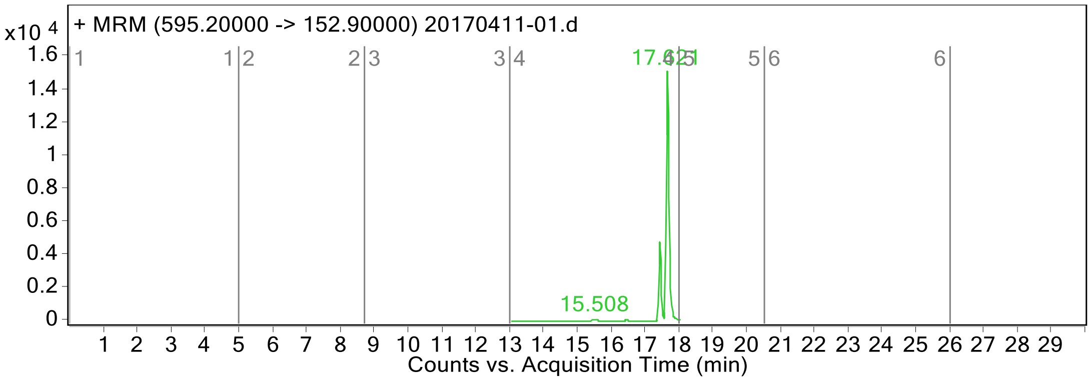

x10 4
+ MRM (595.20000 -> 152.90000) 20170411-01.d
15.508
Counts vs. Acquisition Time (min)

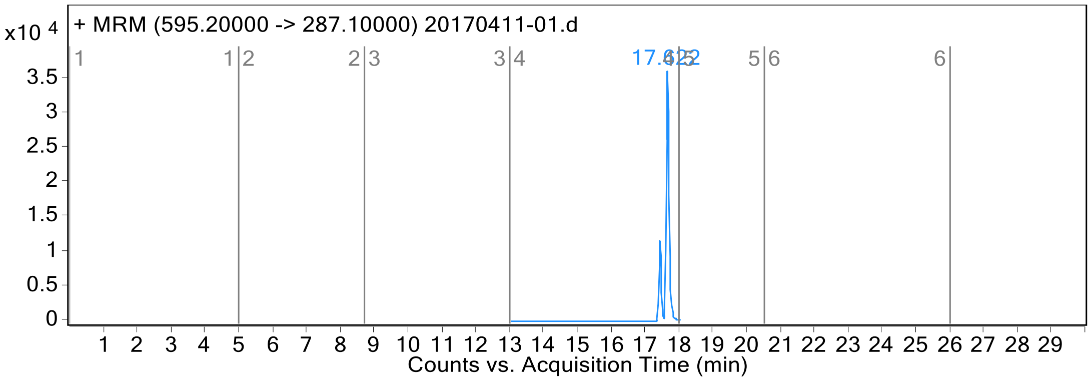

x10 4
+ MRM (595.20000 -> 287.10000) 20170411-01.d
Counts vs. Acquisition Time (min)

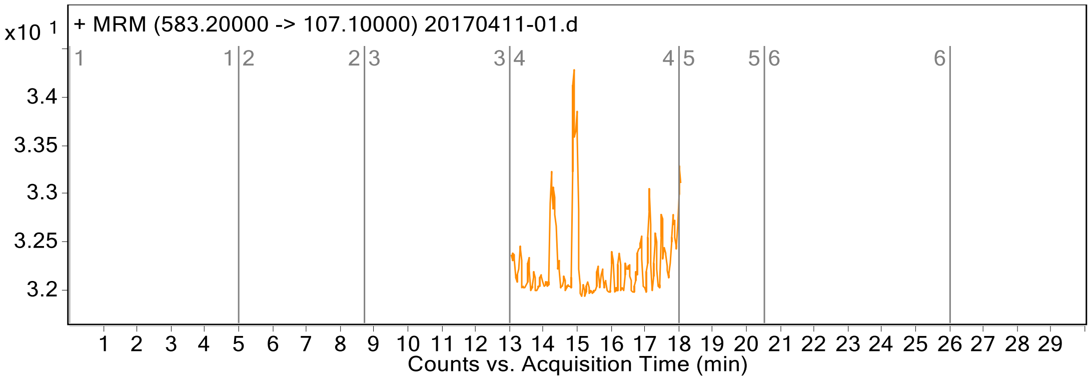

x10 1
+ MRM (583.20000 -> 107.10000) 20170411-01.d
Counts vs. Acquisition Time (min)

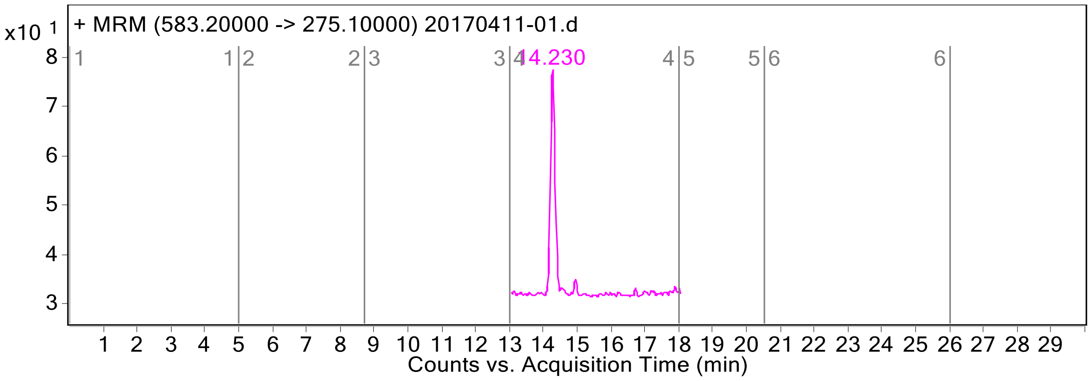

x10 1
+ MRM (583.20000 -> 275.10000) 20170411-01.d
14.230
Counts vs. Acquisition Time (min)

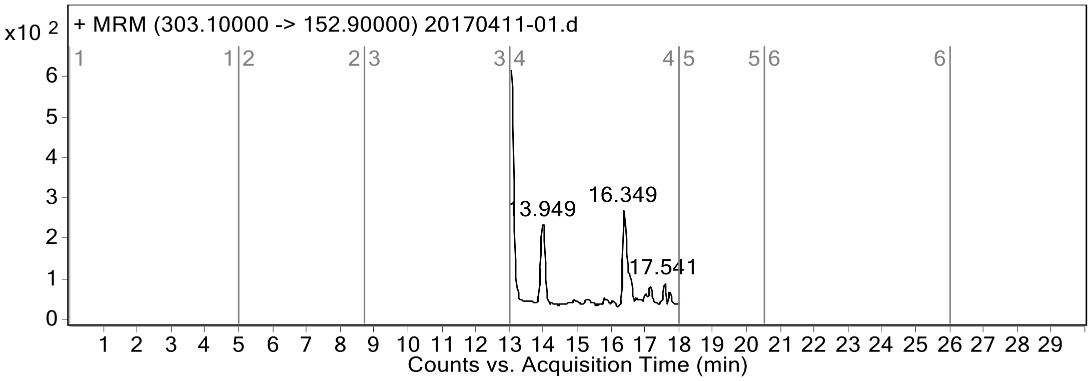

x10 2
+ MRM (303.10000 -> 152.90000) 20170411-01.d
13.949
16.349
17.541
Counts vs. Acquisition Time (min)

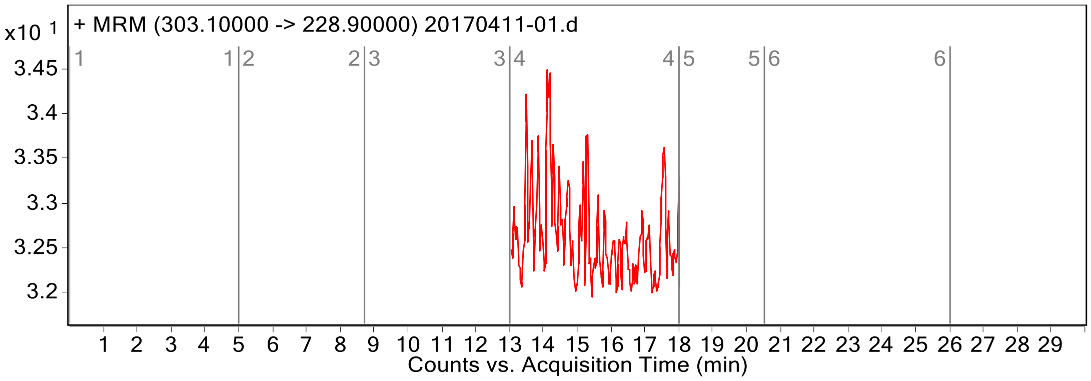

x10 1
+ MRM (303.10000 -> 228.90000) 20170411-01.d
Counts vs. Acquisition Time (min)

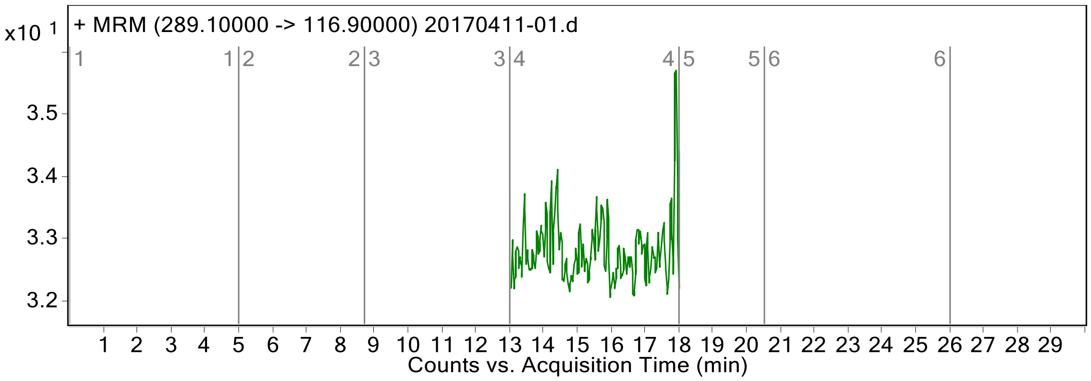
x10 1
+ MRM (289.10000 -> 116.90000) 20170411-01.d
Counts vs. Acquisition Time (min)

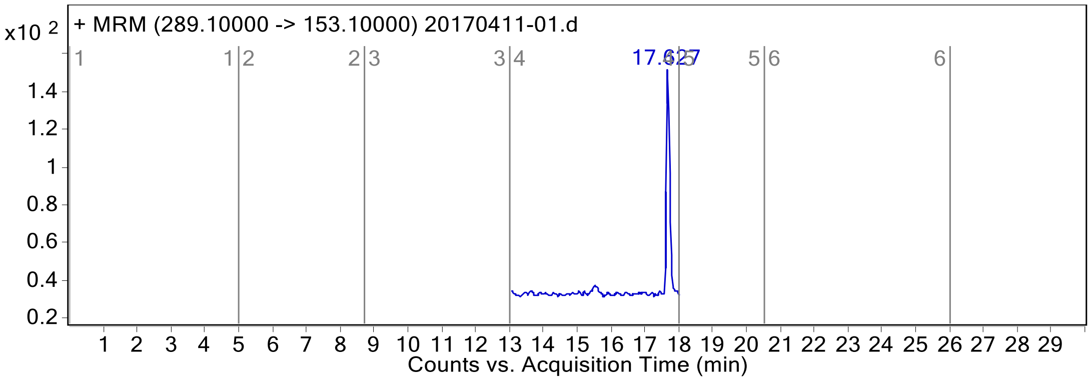
x10 2
+ MRM (289.10000 -> 153.10000) 20170411-01.d
17.627
Counts vs. Acquisition Time (min)

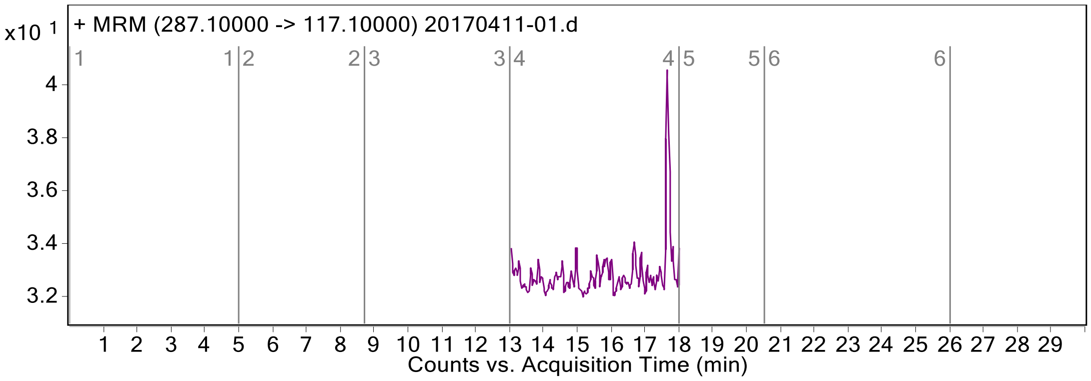
x10 1
+ MRM (287.10000 -> 117.10000) 20170411-01.d
Counts vs. Acquisition Time (min)

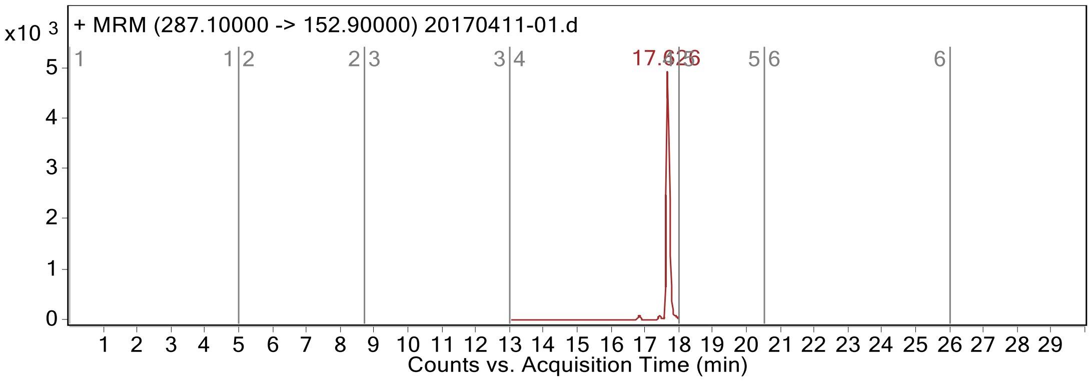
x10 3
+ MRM (287.10000 -> 152.90000) 20170411-01.d
17.626
Counts vs. Acquisition Time (min)

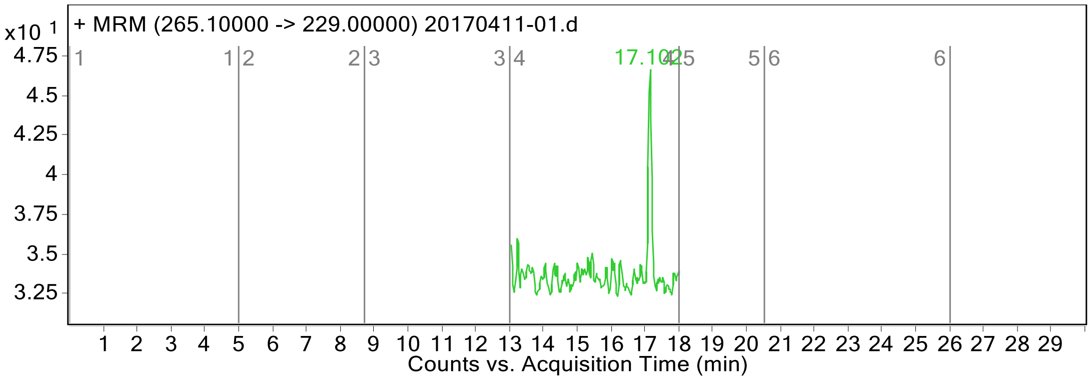

x10 1
+ MRM (265.10000 -> 229.00000) 20170411-01.d
17.102
Counts vs. Acquisition Time (min)

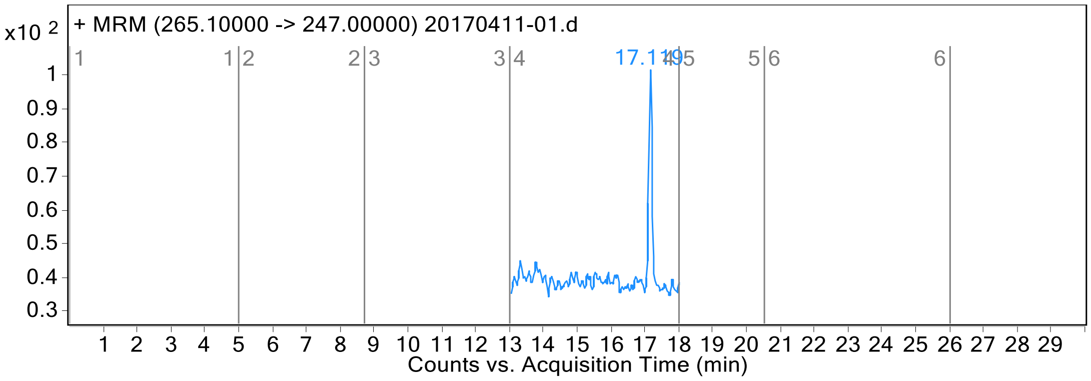

x10 2
+ MRM (265.10000 -> 247.00000) 20170411-01.d
17.119
Counts vs. Acquisition Time (min)

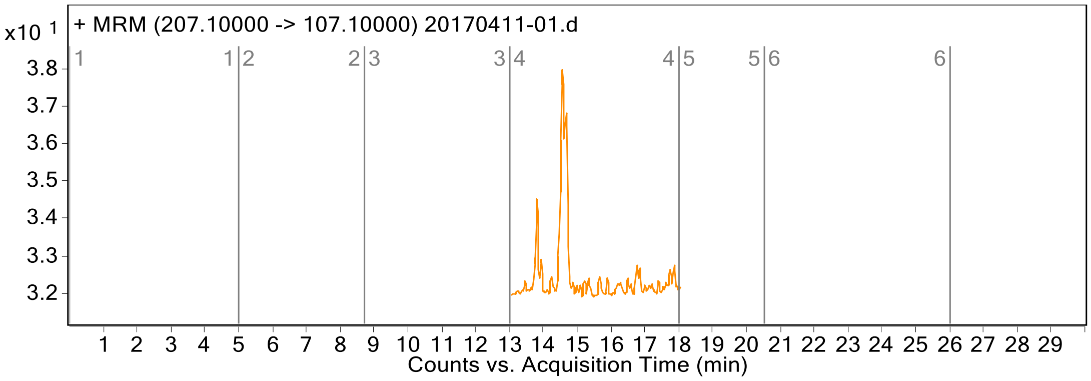

x10 1
+ MRM (207.10000 -> 107.10000) 20170411-01.d
Counts vs. Acquisition Time (min)

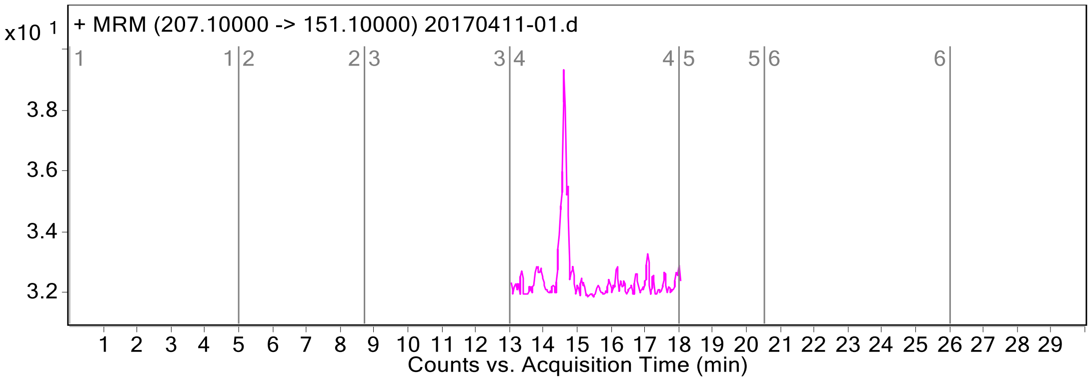

x10 1
+ MRM (207.10000 -> 151.10000) 20170411-01.d
Counts vs. Acquisition Time (min)

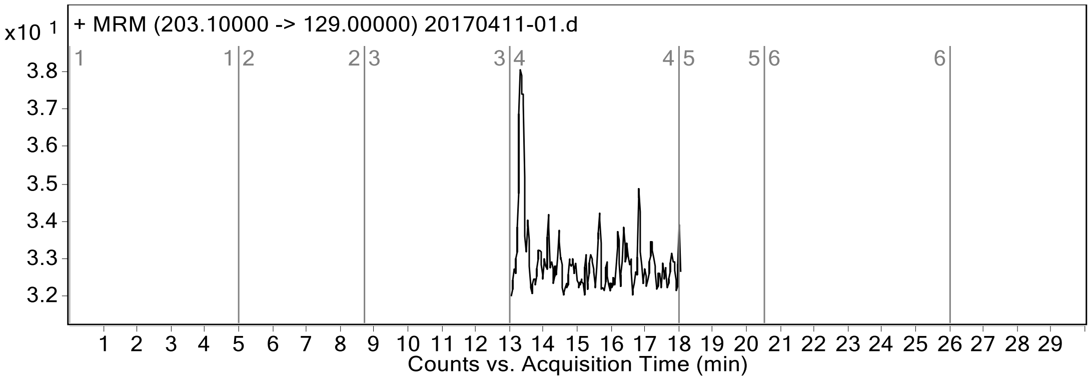
+ MRM (203.10000 -> 129.00000) 20170411-01.d
x10 1
Counts vs. Acquisition Time (min)

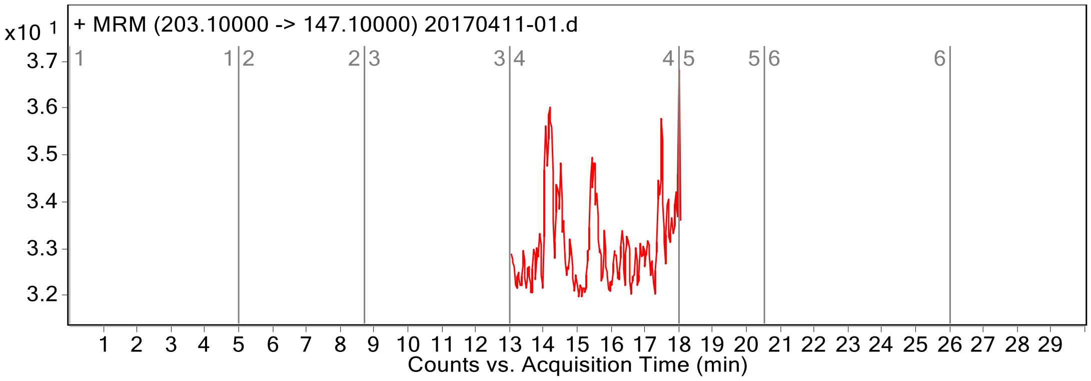
+ MRM (203.10000 -> 147.10000) 20170411-01.d
x10 1
Counts vs. Acquisition Time (min)

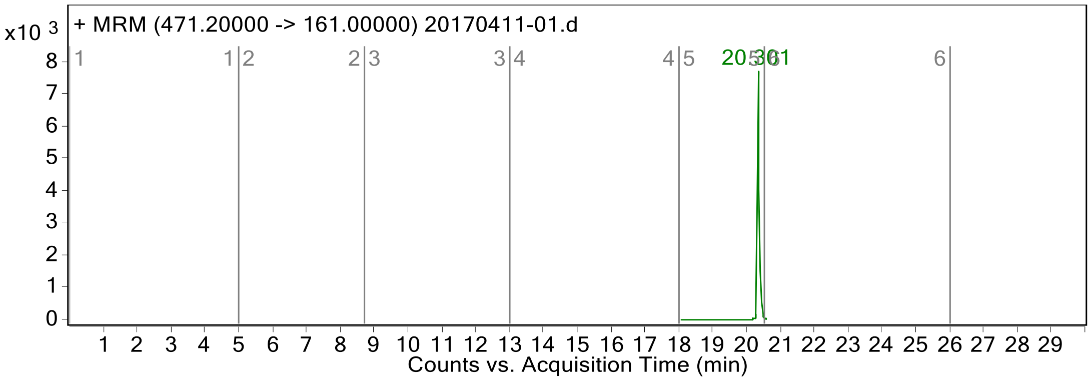
+ MRM (471.20000 -> 161.00000) 20170411-01.d
x10 3
Counts vs. Acquisition Time (min)

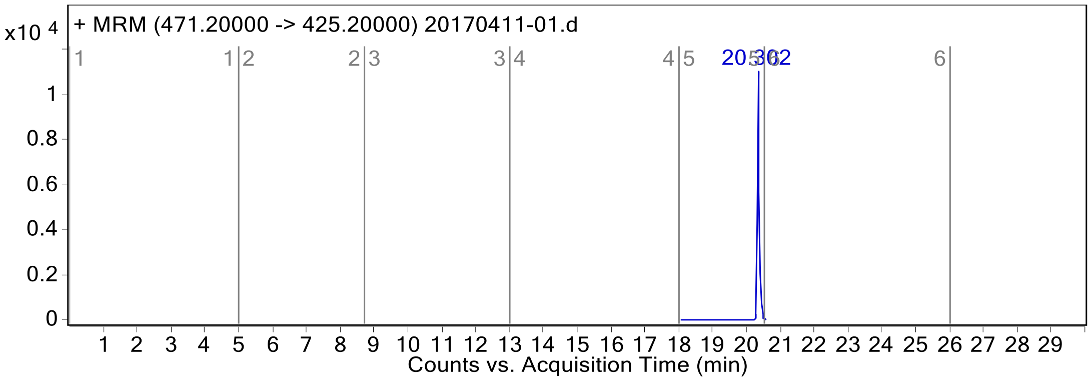
+ MRM (471.20000 -> 425.20000) 20170411-01.d
x10 4
Counts vs. Acquisition Time (min)

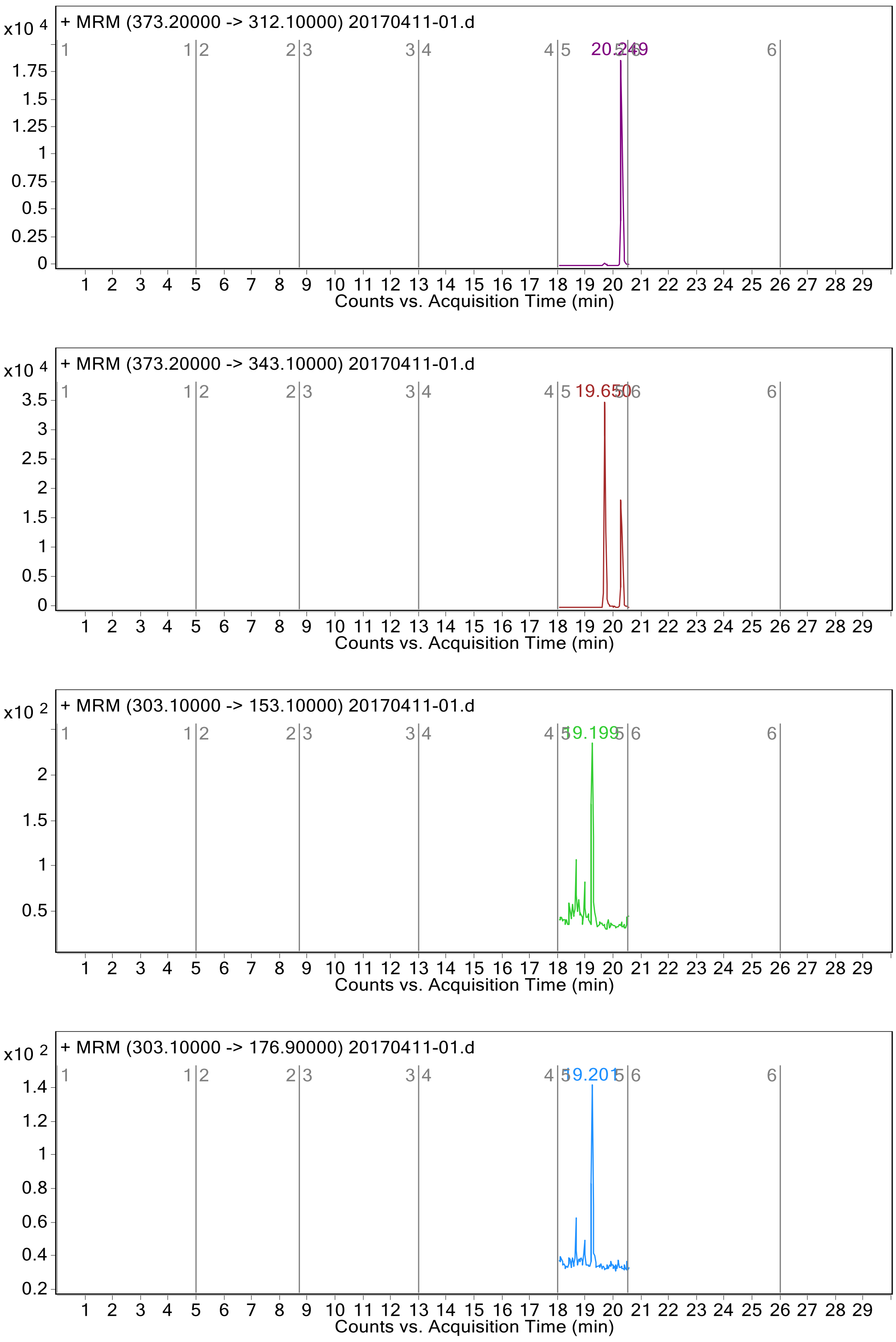

+ MRM (373.20000 -> 312.10000) 20170411-01.d
20.249
Counts vs. Acquisition Time (min)
+ MRM (373.20000 -> 343.10000) 20170411-01.d
19.650
Counts vs. Acquisition Time (min)
+ MRM (303.10000 -> 153.10000) 20170411-01.d
19.199
Counts vs. Acquisition Time (min)
+ MRM (303.10000 -> 176.90000) 20170411-01.d
19.201
Counts vs. Acquisition Time (min)

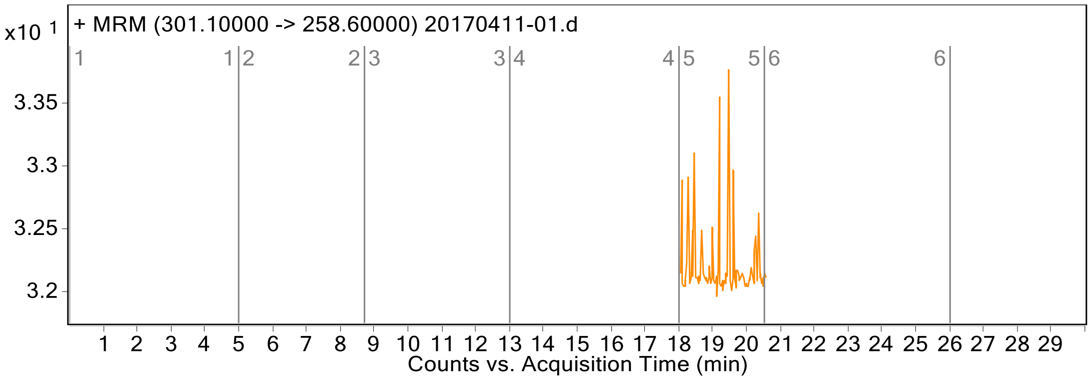
x10 1
+ MRM (301.10000 -> 258.60000) 20170411-01.d
3.35
3.3
3.25
3.2
1 2 3 4 5 6 7 8 9 10 11 12 13 14 15 16 17 18 19 20 21 22 23 24 25 26 27 28 29
Counts vs. Acquisition Time (min)

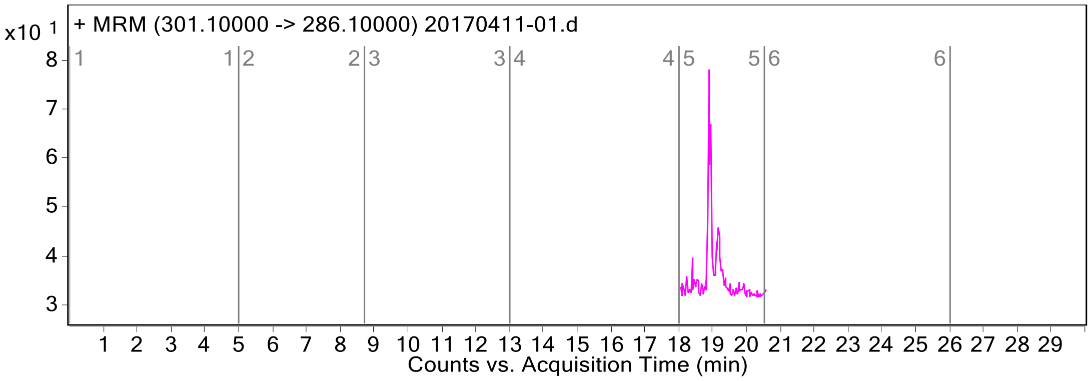
x10 1
+ MRM (301.10000 -> 286.10000) 20170411-01.d
8
7
6
5
4
3
1 2 3 4 5 6 7 8 9 10 11 12 13 14 15 16 17 18 19 20 21 22 23 24 25 26 27 28 29
Counts vs. Acquisition Time (min)

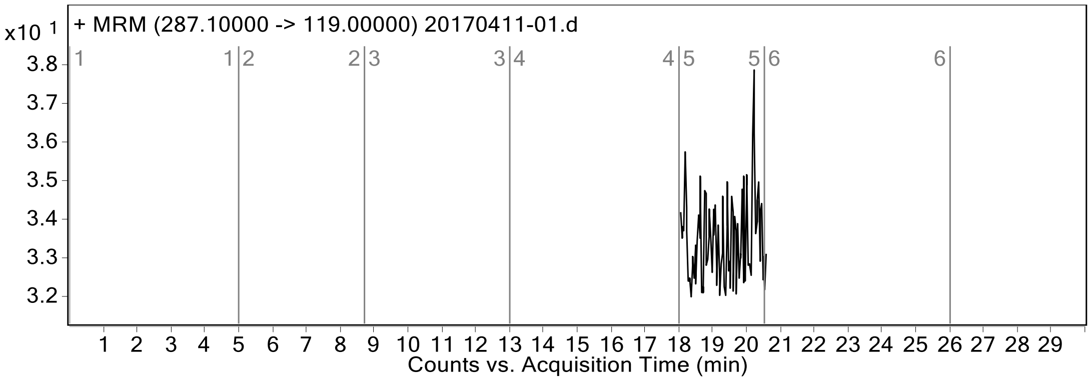
x10 1
+ MRM (287.10000 -> 119.00000) 20170411-01.d
3.8
3.7
3.6
3.5
3.4
3.3
3.2
1 2 3 4 5 6 7 8 9 10 11 12 13 14 15 16 17 18 19 20 21 22 23 24 25 26 27 28 29
Counts vs. Acquisition Time (min)

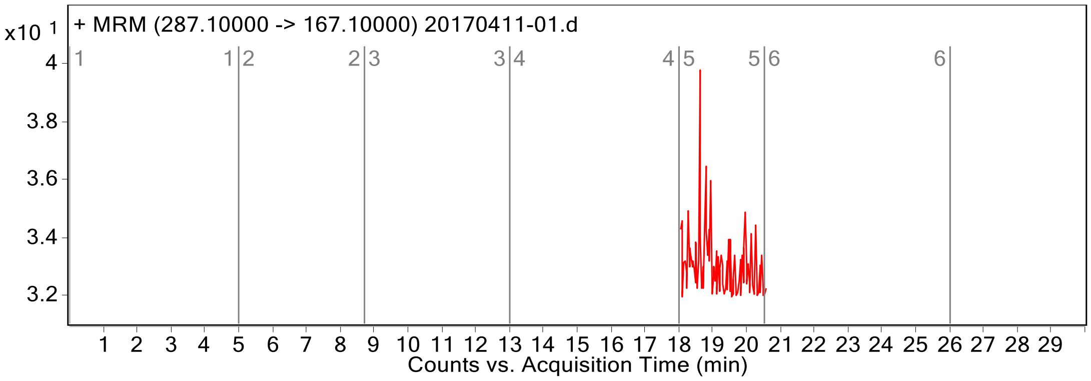
x10 1
+ MRM (287.10000 -> 167.10000) 20170411-01.d
4
3.8
3.6
3.4
3.2
1 2 3 4 5 6 7 8 9 10 11 12 13 14 15 16 17 18 19 20 21 22 23 24 25 26 27 28 29
Counts vs. Acquisition Time (min)

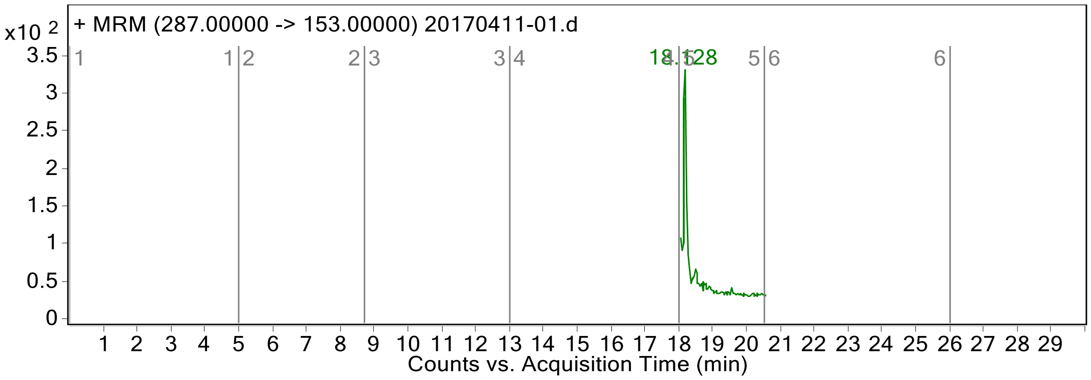
+ MRM (287.00000 -> 153.00000) 20170411-01.d
x10 2
18.128
Counts vs. Acquisition Time (min)

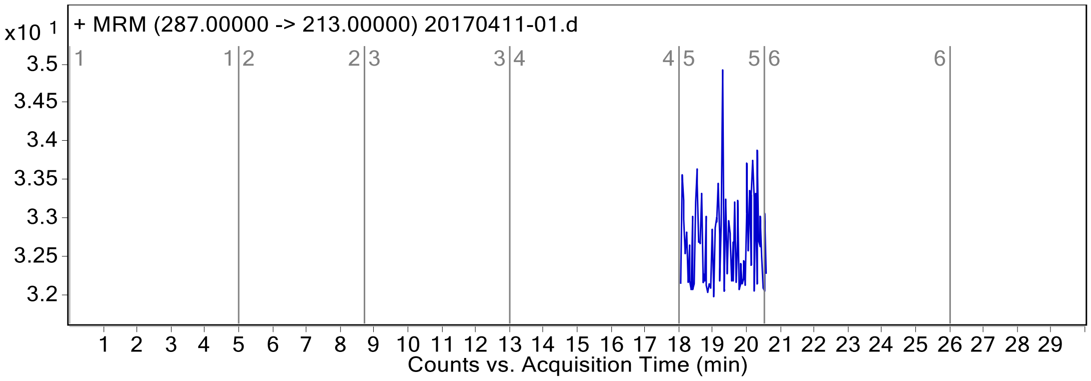
+ MRM (287.00000 -> 213.00000) 20170411-01.d
x10 1
Counts vs. Acquisition Time (min)

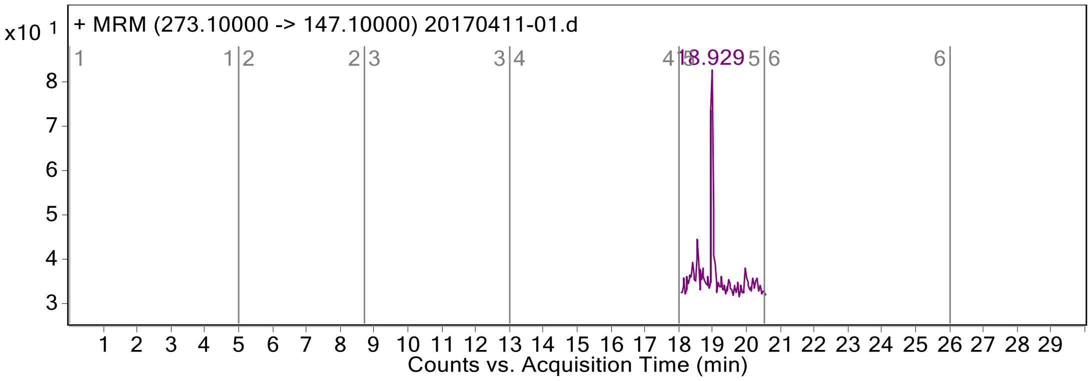
+ MRM (273.10000 -> 147.10000) 20170411-01.d
x10 1
18.929
Counts vs. Acquisition Time (min)

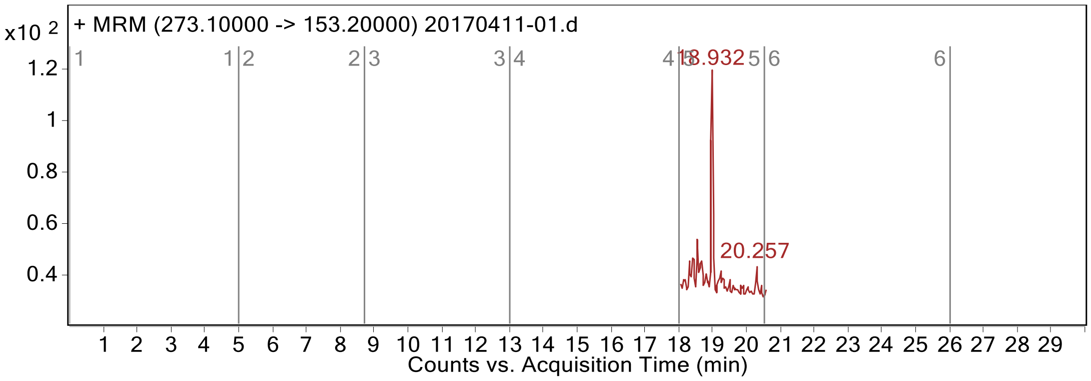
+ MRM (273.10000 -> 153.20000) 20170411-01.d
x10 2
18.932
20.257
Counts vs. Acquisition Time (min)

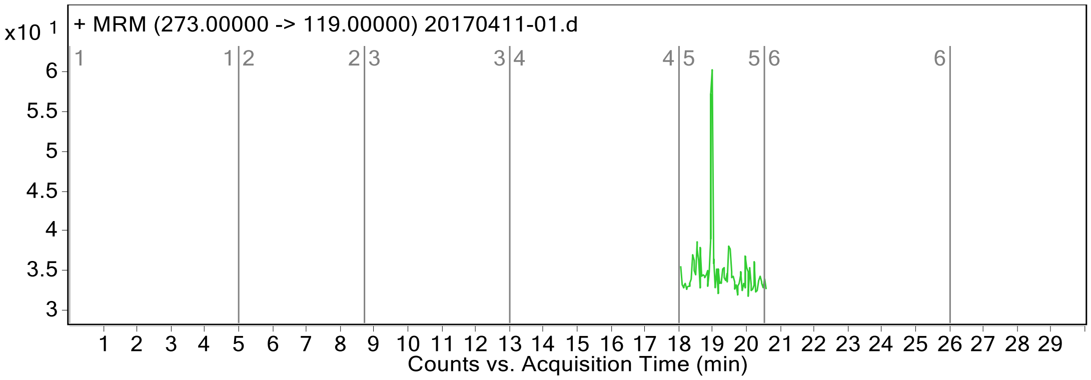
+ MRM (273.00000 -> 119.00000) 20170411-01.d
x10 1
Counts vs. Acquisition Time (min)

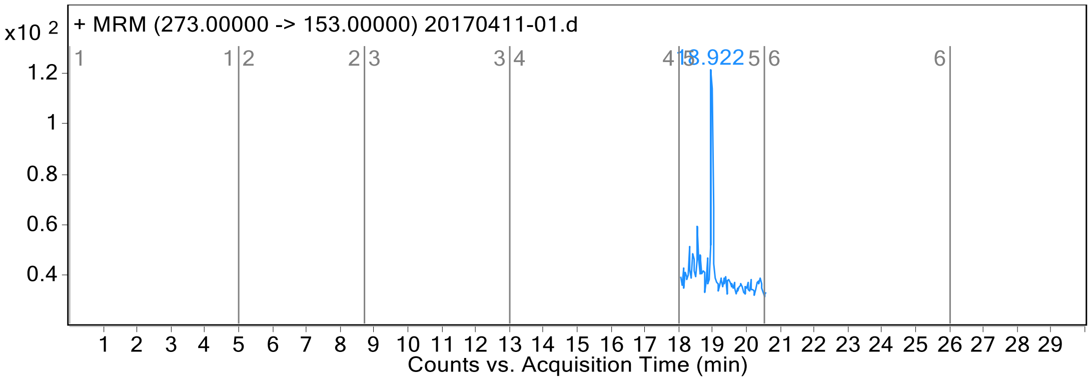
+ MRM (273.00000 -> 153.00000) 20170411-01.d
x10 2
18.922
Counts vs. Acquisition Time (min)

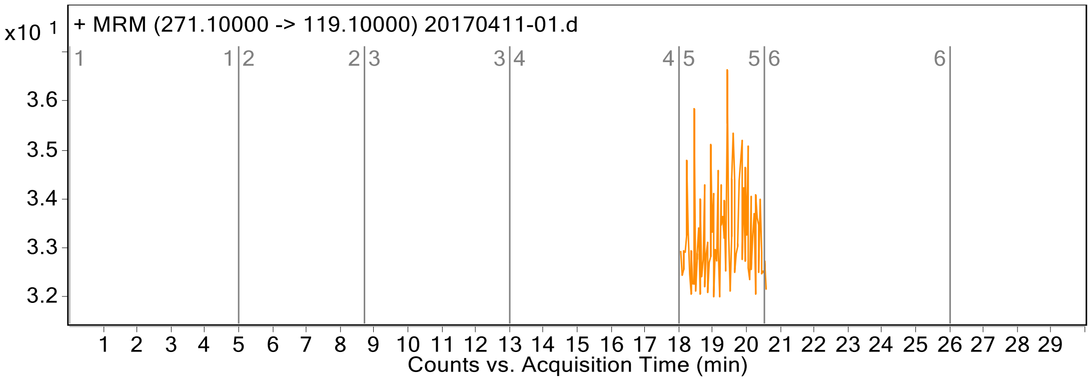
+ MRM (271.10000 -> 119.10000) 20170411-01.d
x10 1
Counts vs. Acquisition Time (min)

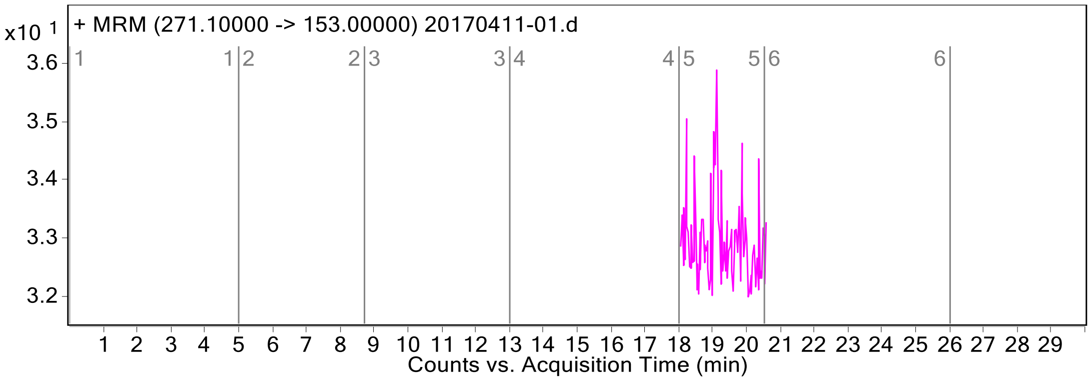
+ MRM (271.10000 -> 153.00000) 20170411-01.d
x10 1
Counts vs. Acquisition Time (min)

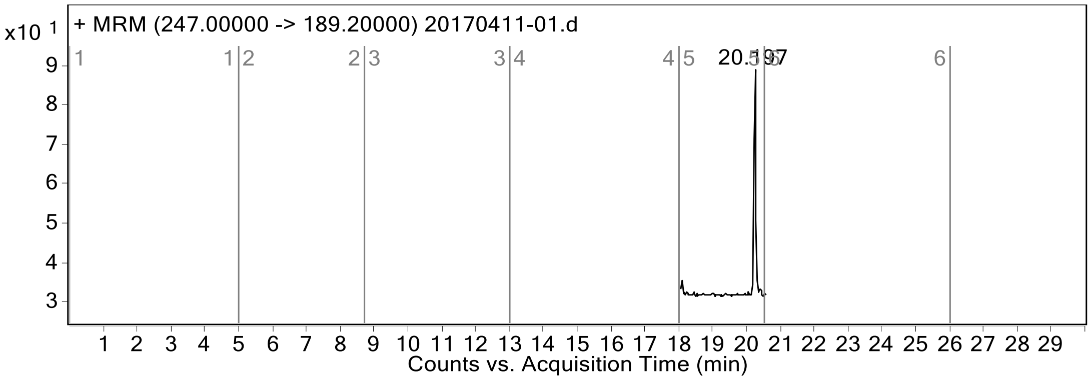
+ MRM (247.00000 -> 189.20000) 20170411-01.d
x10 1
Counts vs. Acquisition Time (min)

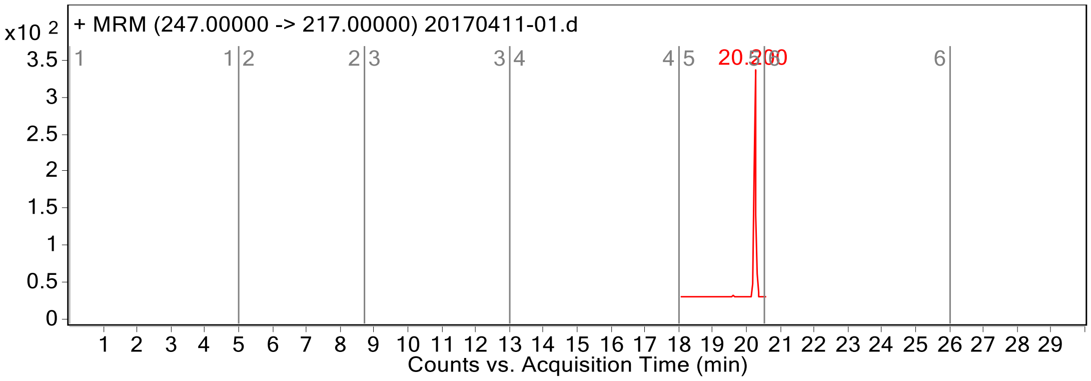
+ MRM (247.00000 -> 217.00000) 20170411-01.d
x10 2
Counts vs. Acquisition Time (min)

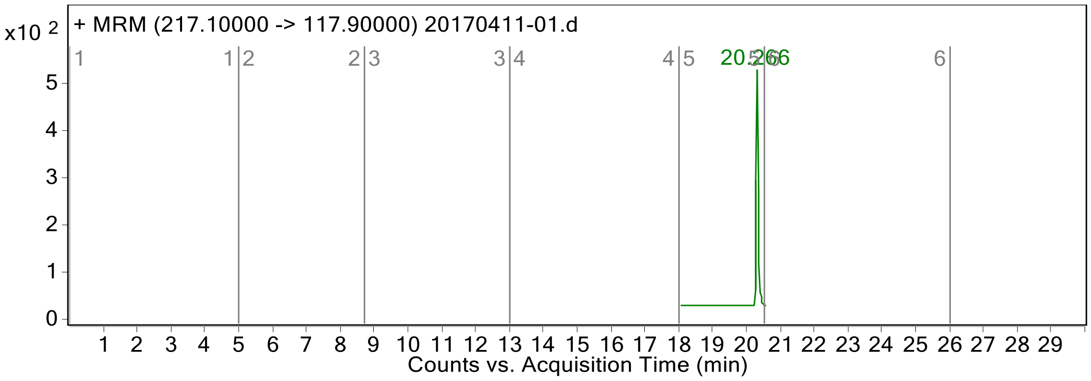
+ MRM (217.10000 -> 117.90000) 20170411-01.d
x10 2
Counts vs. Acquisition Time (min)

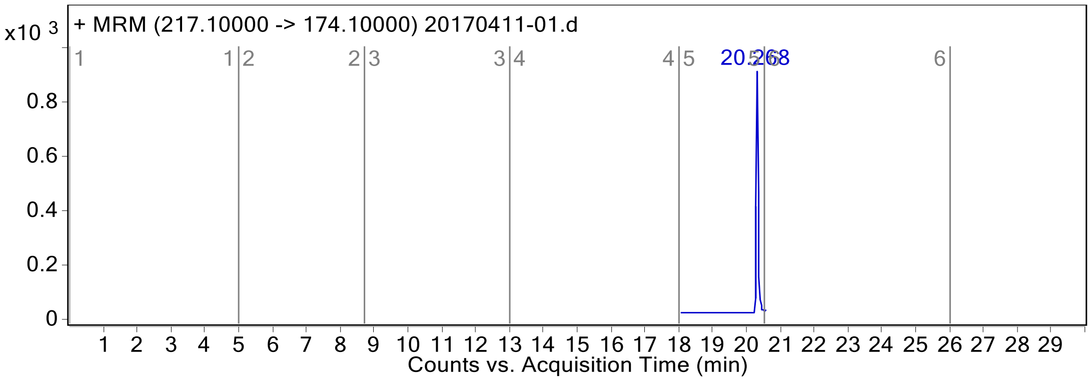
+ MRM (217.10000 -> 174.10000) 20170411-01.d
x10 3
Counts vs. Acquisition Time (min)

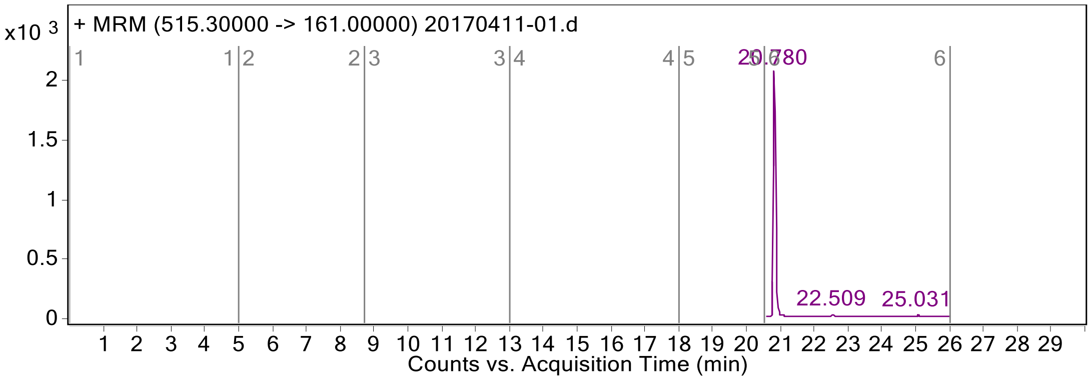
x10 3
+ MRM (515.30000 -> 161.00000) 20170411-01.d
20.780
22.509
25.031
Counts vs. Acquisition Time (min)

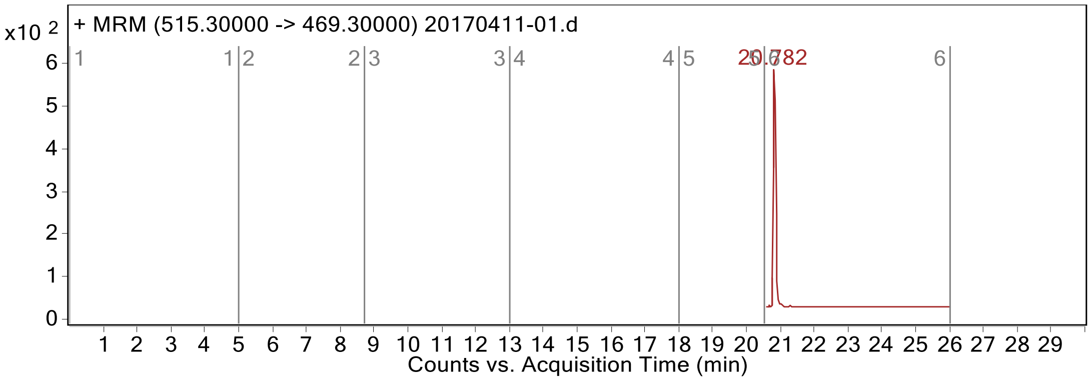
x10 2
+ MRM (515.30000 -> 469.30000) 20170411-01.d
20.782
Counts vs. Acquisition Time (min)

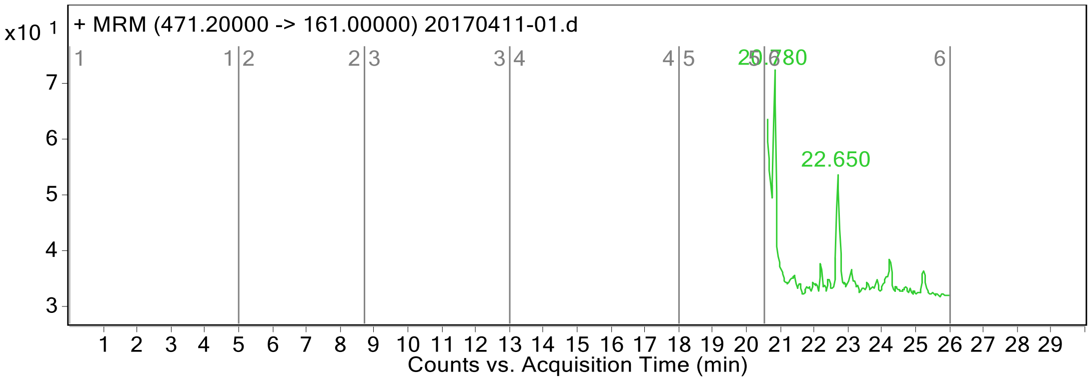
x10 1
+ MRM (471.20000 -> 161.00000) 20170411-01.d
20.780
22.650
Counts vs. Acquisition Time (min)

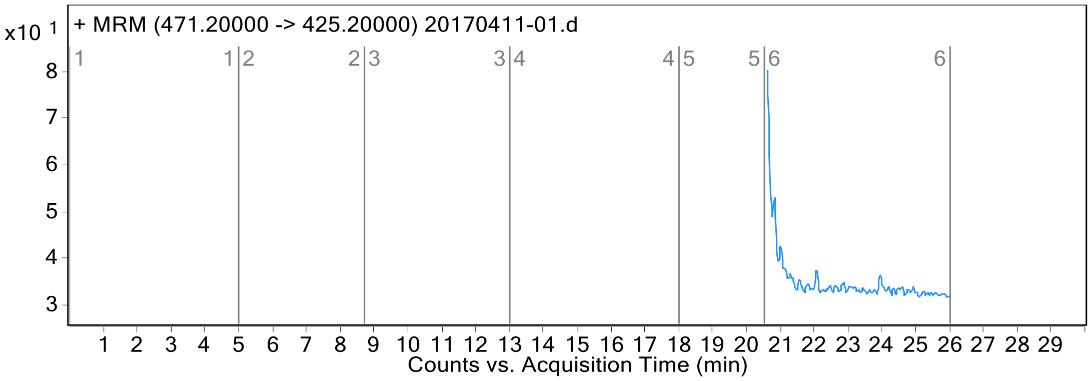
x10 1
+ MRM (471.20000 -> 425.20000) 20170411-01.d
Counts vs. Acquisition Time (min)

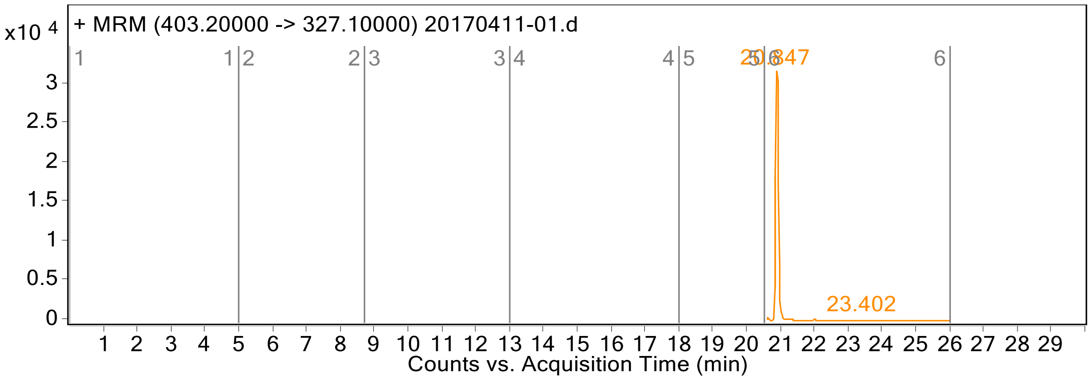
x10 4
+ MRM (403.20000 -> 327.10000) 20170411-01.d
20.847
23.402
Counts vs. Acquisition Time (min)

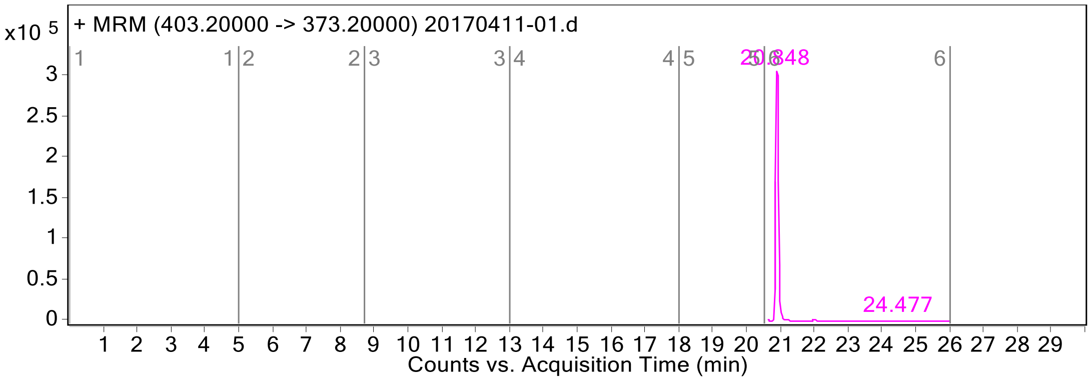
x10 5
+ MRM (403.20000 -> 373.20000) 20170411-01.d
20.848
24.477
Counts vs. Acquisition Time (min)

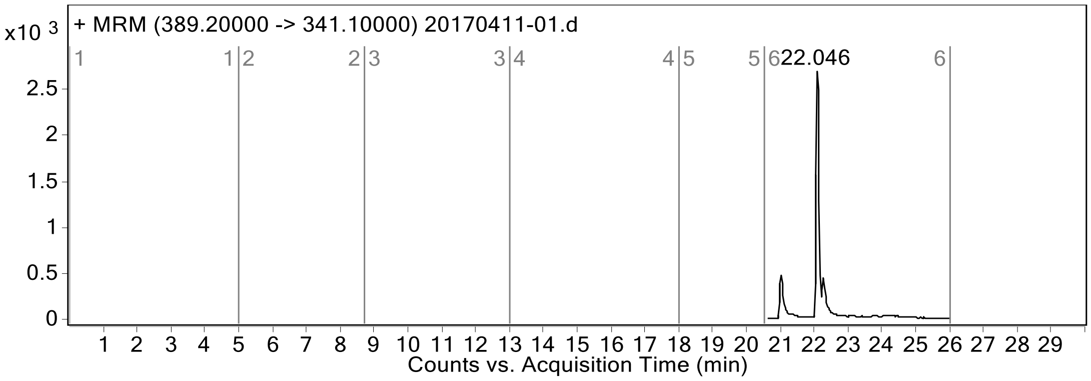
x10 3
+ MRM (389.20000 -> 341.10000) 20170411-01.d
22.046
Counts vs. Acquisition Time (min)

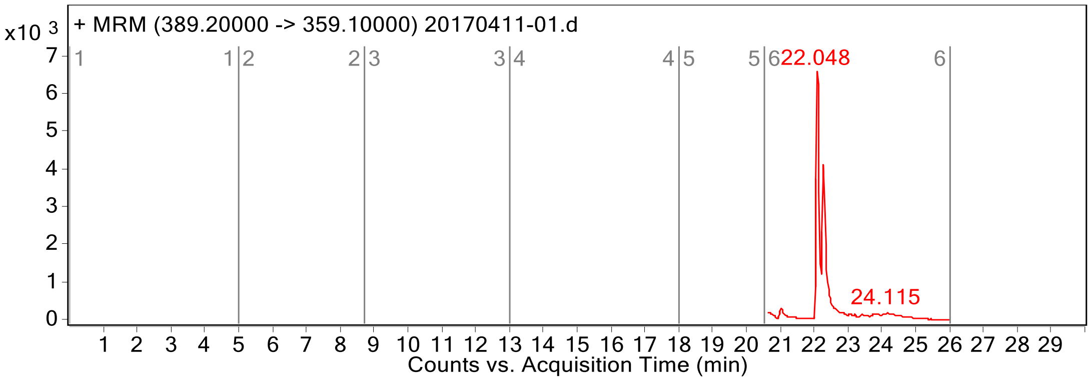
x10 3
+ MRM (389.20000 -> 359.10000) 20170411-01.d
22.048
24.115
Counts vs. Acquisition Time (min)

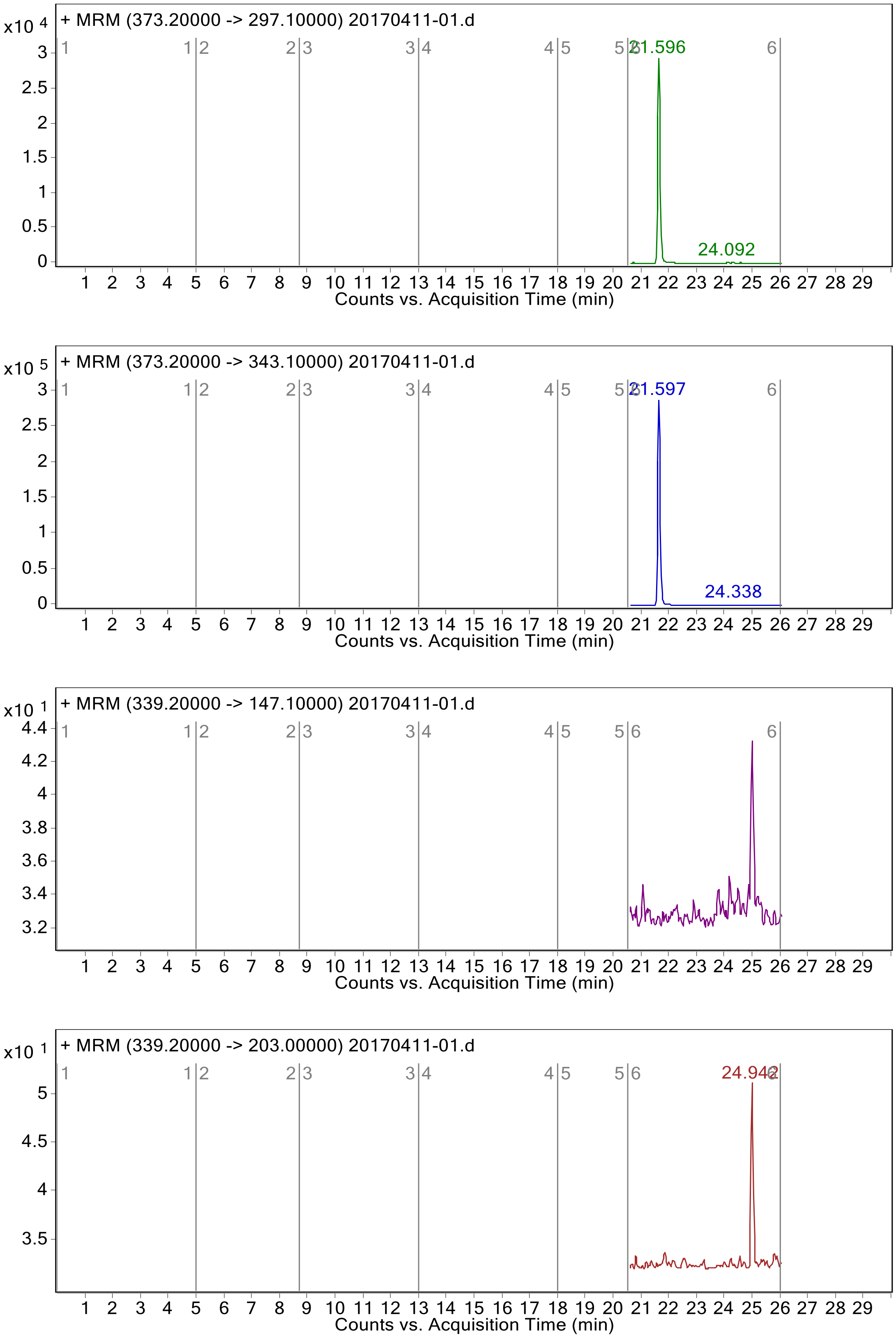
+ MRM (373.20000 -> 297.10000) 20170411-01.d
x10 4
21.596
24.092
Counts vs. Acquisition Time (min)
+ MRM (373.20000 -> 343.10000) 20170411-01.d
x10 5
21.597
24.338
Counts vs. Acquisition Time (min)
+ MRM (339.20000 -> 147.10000) 20170411-01.d
x10 1
Counts vs. Acquisition Time (min)
+ MRM (339.20000 -> 203.00000) 20170411-01.d
x10 1
24.942
Counts vs. Acquisition Time (min)

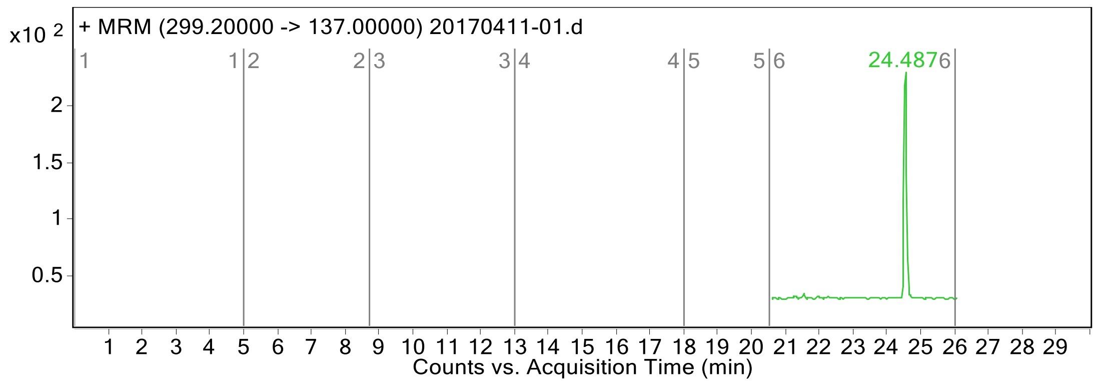
x10 2
+ MRM (299.20000 -> 137.00000) 20170411-01.d
24.4876
Counts vs. Acquisition Time (min)

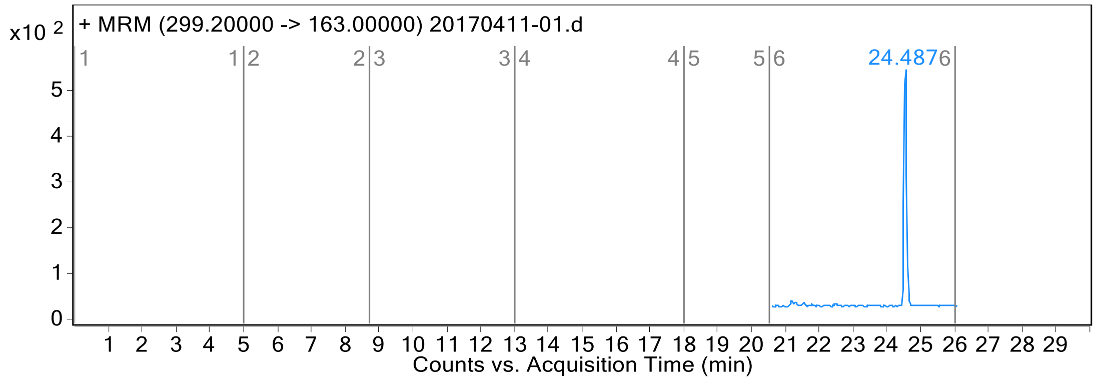
x10 2
+ MRM (299.20000 -> 163.00000) 20170411-01.d
24.4876
Counts vs. Acquisition Time (min)

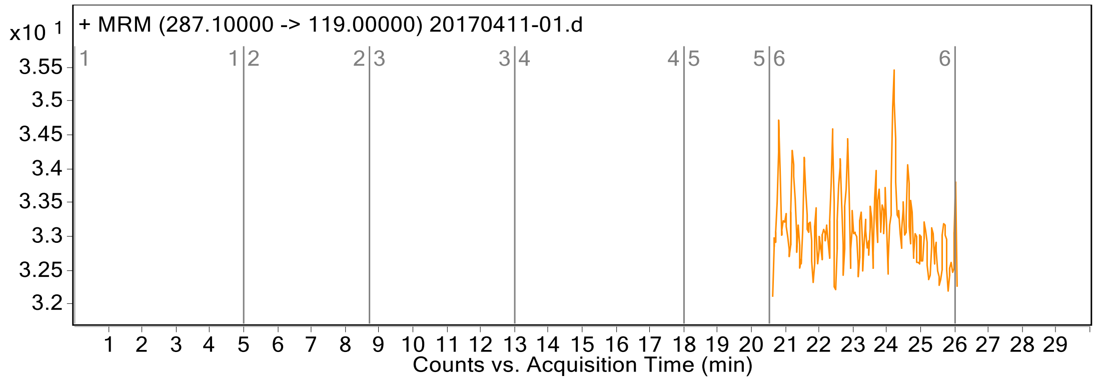
x10 1
+ MRM (287.10000 -> 119.00000) 20170411-01.d
Counts vs. Acquisition Time (min)

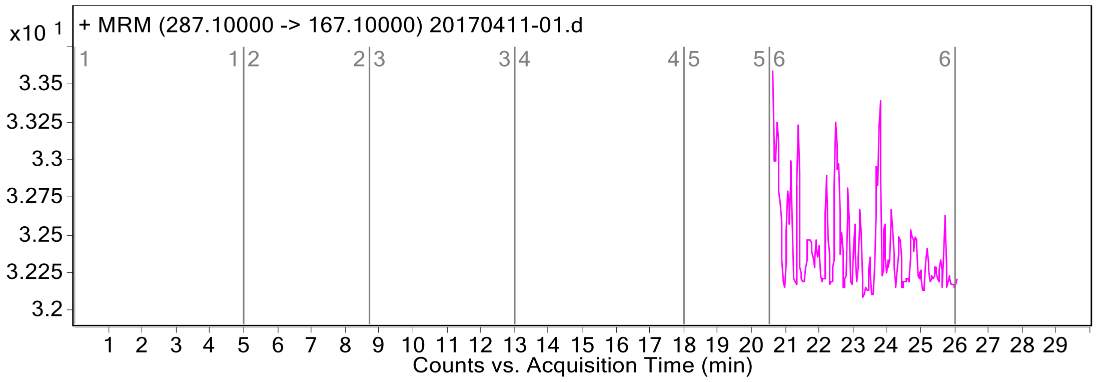
x10 1
+ MRM (287.10000 -> 167.10000) 20170411-01.d
Counts vs. Acquisition Time (min)

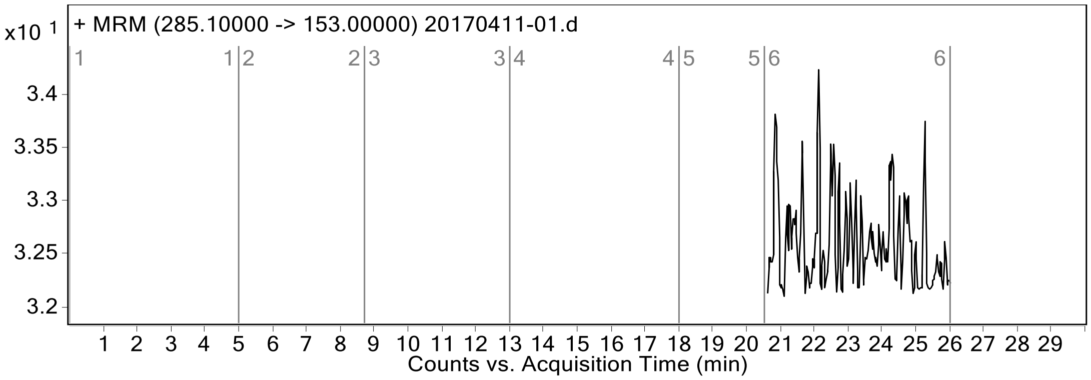

x10 1
+ MRM (285.10000 -> 153.00000) 20170411-01.d
3.4
3.35
3.3
3.25
3.2
1 2 3 4 5 6 7 8 9 10 11 12 13 14 15 16 17 18 19 20 21 22 23 24 25 26 27 28 29
Counts vs. Acquisition Time (min)

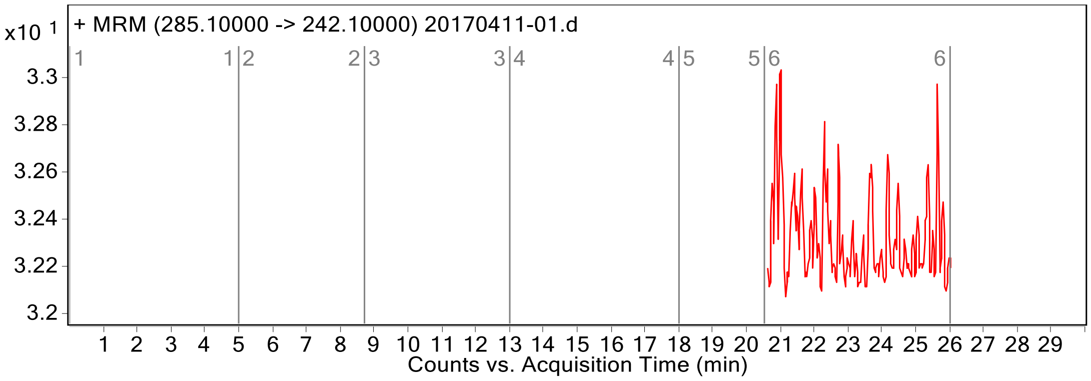

x10 1
+ MRM (285.10000 -> 242.10000) 20170411-01.d
3.3
3.28
3.26
3.24
3.22
3.2
1 2 3 4 5 6 7 8 9 10 11 12 13 14 15 16 17 18 19 20 21 22 23 24 25 26 27 28 29
Counts vs. Acquisition Time (min)

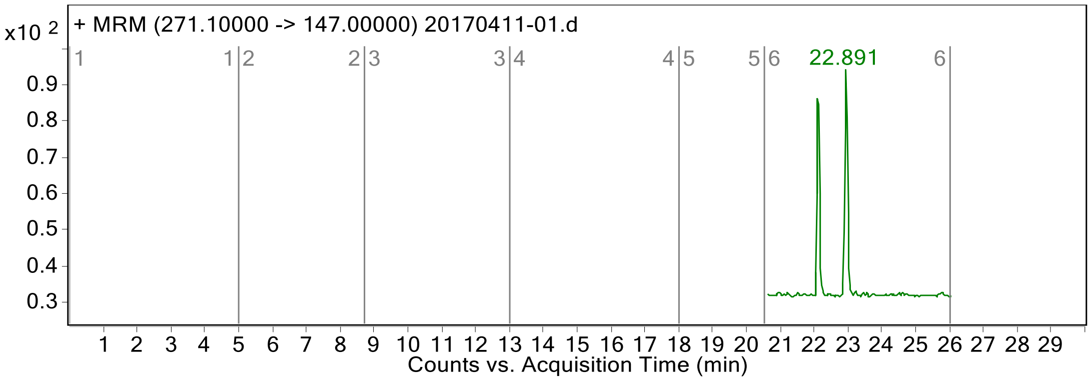

x10 2
+ MRM (271.10000 -> 147.00000) 20170411-01.d
22.891
0.9
0.8
0.7
0.6
0.5
0.4
0.3
1 2 3 4 5 6 7 8 9 10 11 12 13 14 15 16 17 18 19 20 21 22 23 24 25 26 27 28 29
Counts vs. Acquisition Time (min)

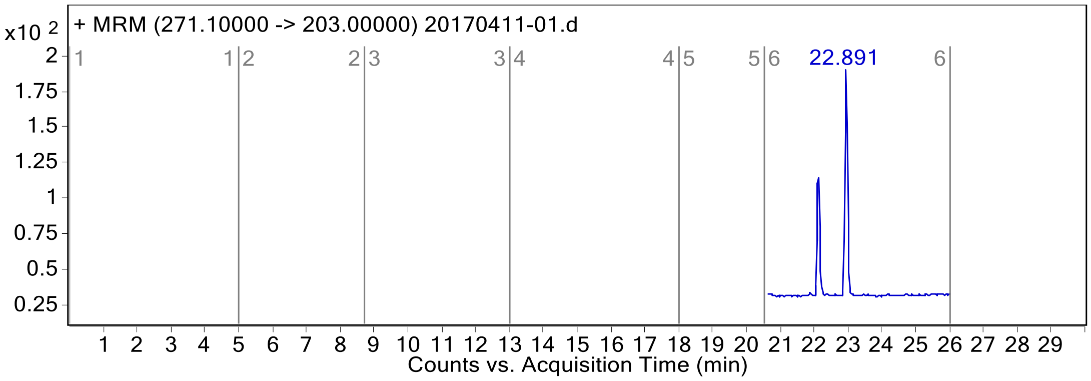

x10 2
+ MRM (271.10000 -> 203.00000) 20170411-01.d
22.891
2
1.75
1.5
1.25
1
0.75
0.5
0.25
1 2 3 4 5 6 7 8 9 10 11 12 13 14 15 16 17 18 19 20 21 22 23 24 25 26 27 28 29
Counts vs. Acquisition Time (min)

UPLC-Q-TOF-MS spectrum of Zhishi extract

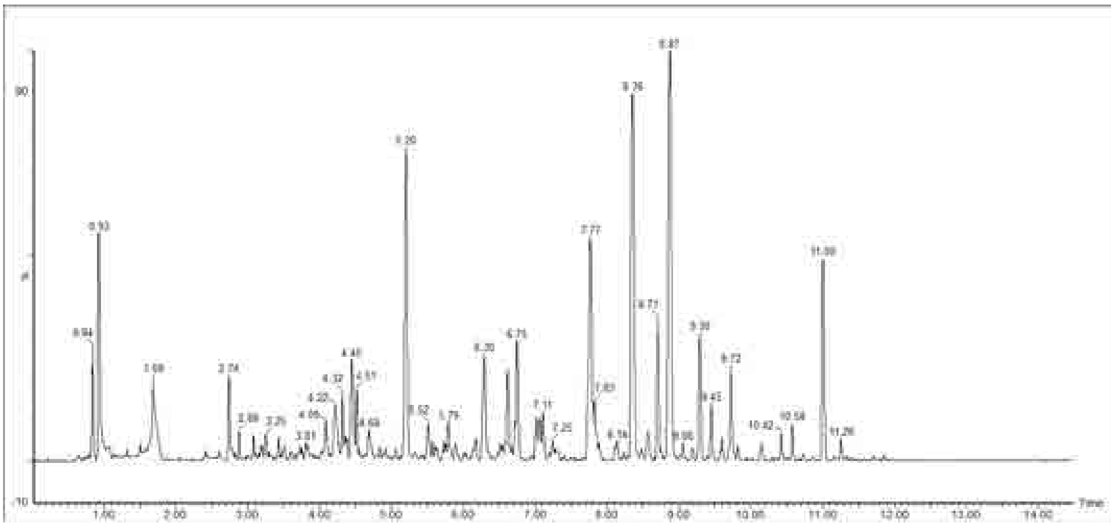

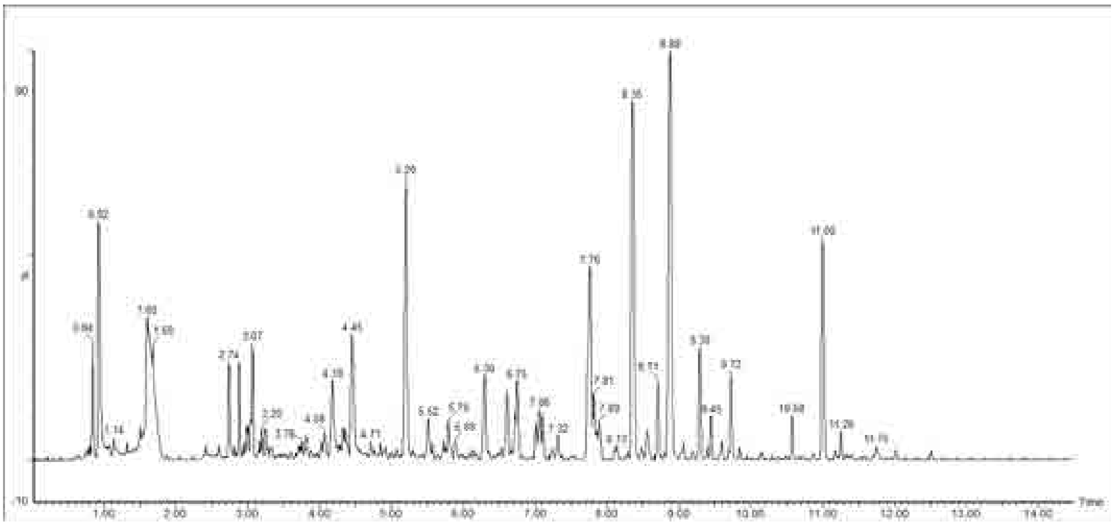

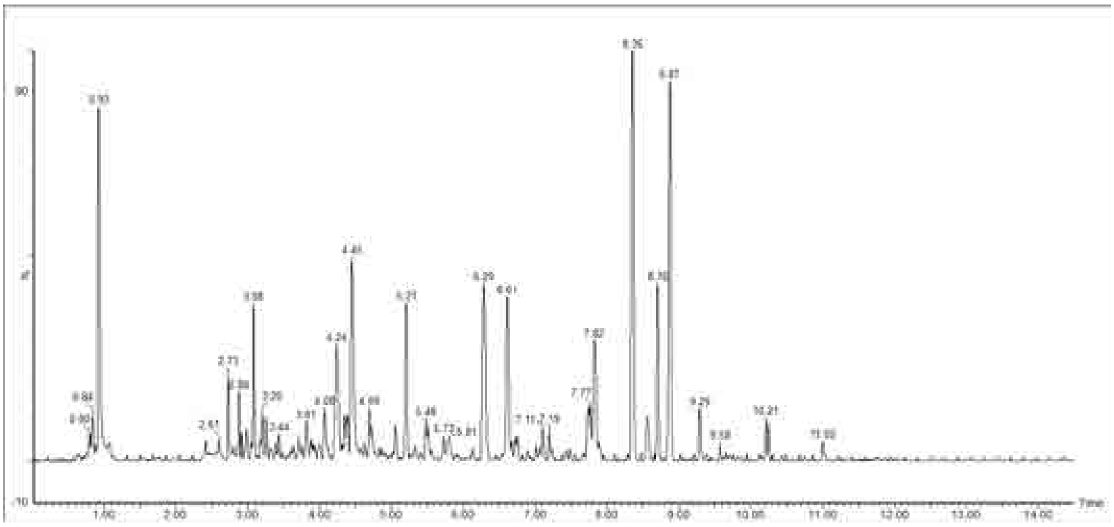

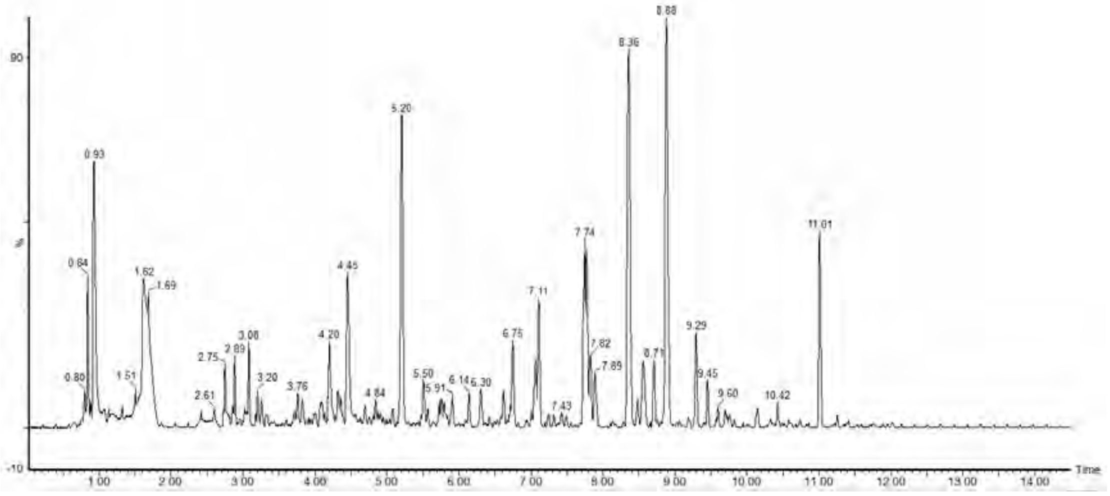
90
%
-10
Time
1.00
2.00
3.00
4.00
5.00
6.00
7.00
8.00
9.00
10.00
11.00
12.00
13.00
14.00
0.93
5.20
8.36
8.88
7.74
11.01
4.45
7.11
9.29

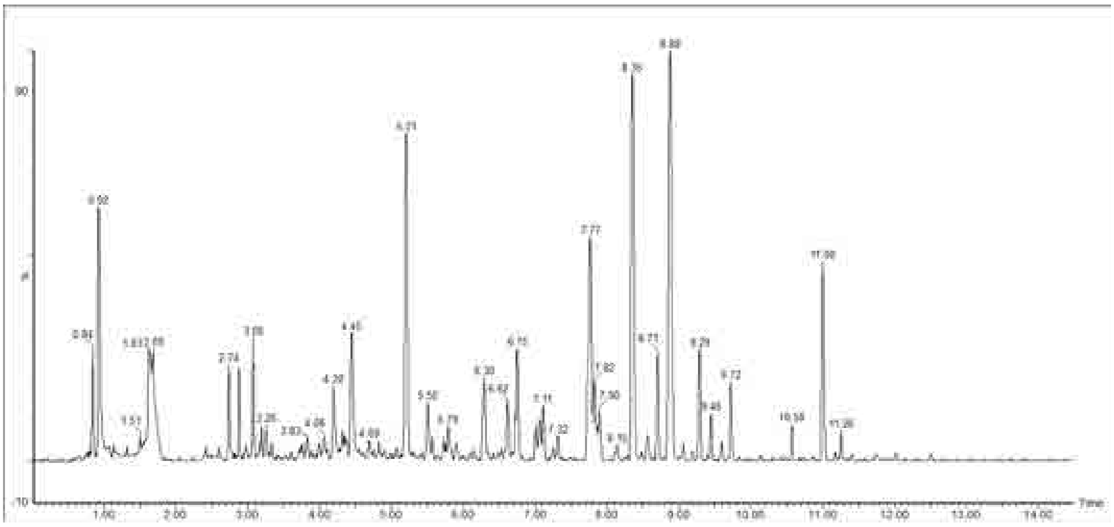
90
%
-10
Time
1.00
2.00
3.00
4.00
5.00
6.00
7.00
8.00
9.00
10.00
11.00
12.00
13.00
14.00
0.92
8.88
7.77
4.45
9.29
